# Exploiting decarbonylation and dehydrogenation of formamides for the synthesis of ureas, polyureas, and poly(urea-urethanes)†

**DOI:** 10.1039/d4sc03948c

**Published:** 2024-09-05

**Authors:** James Luk, Alister S. Goodfellow, Nachiket Deepak More, Michael Bühl, Amit Kumar

**Affiliations:** a EaStCHEM, School of Chemistry, University of St. Andrews North Haugh, St. Andrews KY16 9ST UK ak336@st-andrews.ac.uk mb105@st-andrews.ac.uk

## Abstract

Urea derivatives, polyureas, and poly(urea-urethanes) are materials of great interest. However, their current methods of synthesis involve toxic feedstocks – isocyanate and phosgene gas. There is significant interest in developing alternative methodologies for their synthesis from safer feedstocks. We report here new methods for the synthesis of urea derivatives, polyureas, and poly(urea-urethane) using a ruthenium pincer catalyst. In this approach, urea derivatives and polyureas are synthesized from the self-coupling of formamides and diformamides, respectively, whereas poly(urea-urethanes) are synthesized from the coupling of diformamides and diols. CO and H_2_ gases are eliminated in all these processes. Decarbonylation of formamides using such organometallic catalysts has not been reported before and therefore mechanistic insights have been provided using experiments and DFT computation to shed light on pathways of these processes.

## Abstract 2

We report here new methods to make non-isocyanates polyureas and poly(urea-urethanes) from diformamides feedstock with the extrusion of H_2_ and CO.

## Graphical abstract

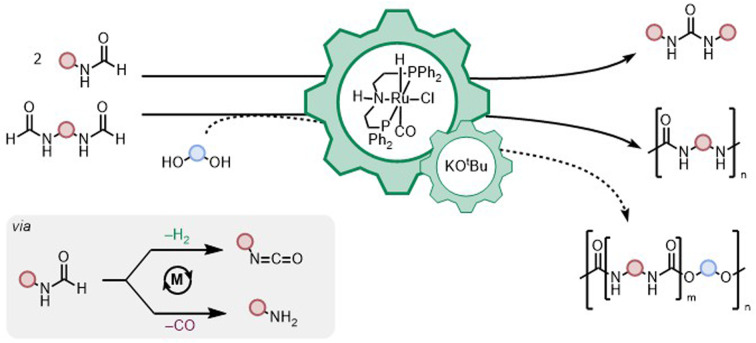

## Introduction

**Fig. 1:**
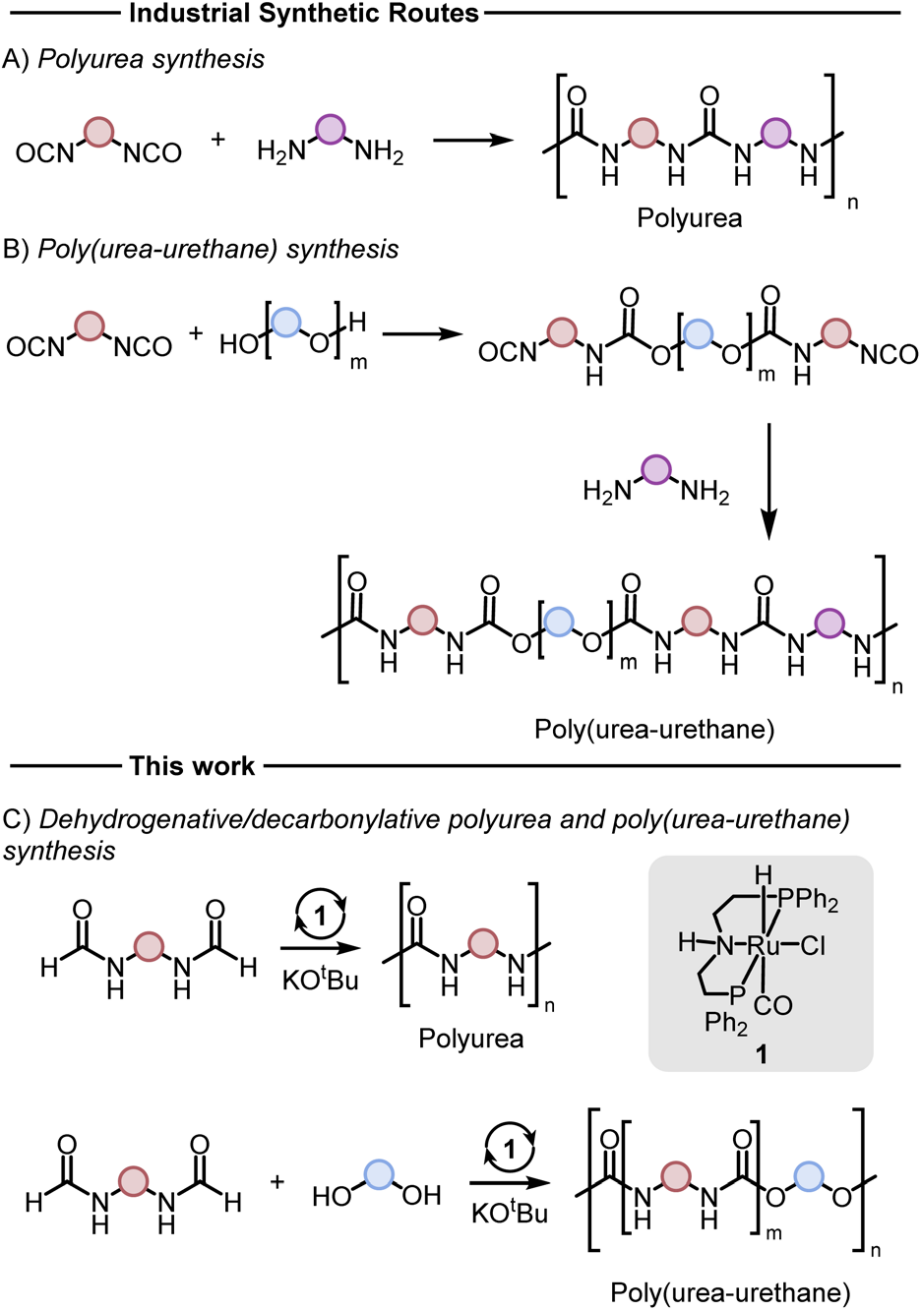
Conventional methods for the synthesis of polyureas (A) and poly(urea-urethane) (B), and the methods disclosed herein (C).

Polyureas,^1^ and poly(urea-urethanes)^2^ are important classes of plastics with various applications ranging from construction materials, foams, coatings, adhesives, and biomedical industries. Although the production technologies of these polymers and their markets are very well-established, these polymers are produced from diisocyanates which can be toxic (Fig. 1A and B). The number of new occupational diseases caused by diisocyanates is exceptionally high and is estimated to be ∼6000 per year.^3^ This has resulted in strict restrictions on the use of this feedstock. For example, according to a recent REACH regulation of the European Union, since August 24, 2023, mandatory adequate training is required for all professional users to handle diisocyanates (on their own or constituent in other substances) in a concentration of more than 0.1% by weight.^3^ Additionally, the precursor to make diisocyanates is phosgene gas which is even more toxic and hazardous to human health and the environment. These have led to increased interest in the development of new methods to substitute diisocyanate with safer feedstocks in the polymer industry.

Various methods for the synthesis of polyureas without using diisocyanates have been reported in the literature, however, they suffer from drawbacks such as harsh reaction conditions, use of expensive reagents/catalysts, and limited substrate scope providing opportunities for the development of new methods.^4–12^ Poly(urea-urethanes) are prepared from the polyaddition reaction of diols and diamines with diisocyanates (Fig. 1B). This is usually achieved in two stages where first a diisocyanate is added to a diol to make a prepolymer with end-capped isocyanate to which a diamine is added to make the poly(urea-urethane).

Reactions based on catalytic dehydrogenation are an atom-economic approach for the synthesis of organic compounds such as ketones, esters, amides, carboxylic acids, and urea derivatives.^13–17^ The approach has also been utilized for the synthesis of polymers. For example, high molecular weight polyesters can be synthesized from the acceptorless dehydrogenative coupling of diols using Milstein's ruthenium catalysts.^18^ The synthesis of polyamides has been reported from the dehydrogenative coupling of diols and diamines by Guan^19^ and Milstein.^20^ We,^21–23^ Robertson,^24^ and Liu^25^ have independently reported on the synthesis of polyureas from the coupling of diamines with methanol or diformamides using ruthenium or manganese pincer catalysts. We have also recently reported the synthesis of polyethyleneimine derivatives from the manganese catalysed coupling of ethylene glycol and ethylenediamine.^26^ We now report new methodologies for the synthesis of polyureas, and poly(urea-urethane) from diformamide and diol feedstock using pincer catalysts. Our strategy is inspired by the previous work on the pincer complex catalysed dehydrogenative reactions to form urea derivatives, and organic carbamates by Hong,^27^ Gunanathan,^28^ Milstein,^29^ Hazari,^30,31^ Bernskoetter,^30,31^ and Sanford.^32^

## Results and discussion

**Scheme 1:**
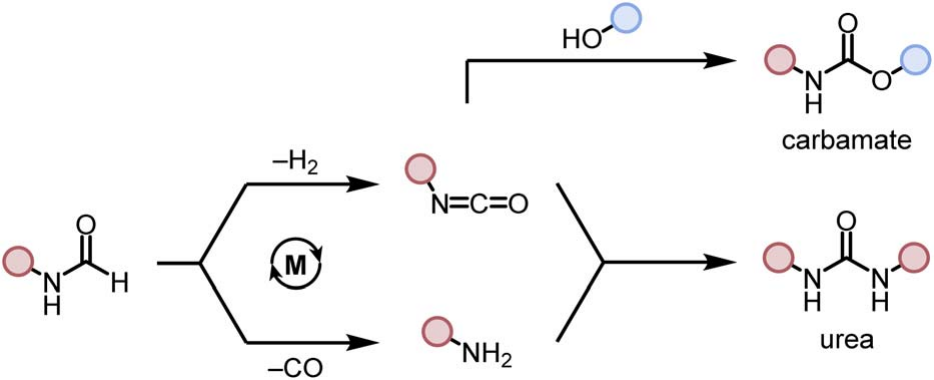
Dehydrogenative and decarbonylative pathways for the formation of carbamate and urea derivative.

We started our investigation by studying the effects of catalytic conditions on the reaction of *N*-cyclohexylformamide with cyclohexanol. The Ru-MACHO^PPh_2_^ complex 1 was used as a precatalyst as it has been used in the past for the synthesis of (poly)urea derivatives from the coupling of (di)formamides and (di)amines.^24^ Inspired by previous studies on the dehydrogenative catalysis, the initial study was performed using 1 mol% complex 1, 4 mol% KO^*t*^Bu, at 150 °C for 24 h in a sealed J. Young's flask (Table 1). Analysis of the reaction mixture by ^1^H NMR spectroscopy and GC-MS showed 89% conversion of formamide. Interestingly, a mixture of three products – carbamate (58%), *N*,*N*′-dicyclohexylurea (22%), and *N*-cyclohexylamine (9%) was obtained (Table 1, entry 1). Based on previous studies,^32^ we suggest that the formation of carbamate occurs through the dehydrogenation of formamide to isocyanate and its subsequent reaction with the alcohol nucleophile to form the carbamate derivative. Additionally, we speculate that under these reaction conditions, formamide undergoes decarbonylation to form an amine that can react with the isocyanate intermediate to form the corresponding urea derivative (Scheme 1). This was confirmed by a GC-TCD (Gas Chromatography Thermal Conductivity Detector) analysis of the gas produced in the overhead space of the reaction flask that showed the presence of H_2_ and CO (ESI, Fig. S48†). We hypothesize that the relative rate of dehydrogenation *vs.* decarbonylation could affect the selectivity towards the formation of carbamate and urea derivatives. For the selective formation of carbamate, the decarbonylation process needs to be avoided completely and for that of urea derivatives, the rate of dehydrogenation and decarbonylation should be similar (Scheme 1). We therefore studied the effects of various catalytic conditions on the selectivity of the products. Changing the precatalyst from Ru-MACHO^PPh_2_^ to the ^i^Pr analogue (complex 2) led to a higher conversion (91%) as well as a higher selectivity towards carbamate (81% yield, entry 2). Interestingly, when the Mn-MACHO^PPh_2_^ complex 3 was used under the same condition, a lower conversion of formamide was obtained (62%) and the carbamate was obtained in 35% yield (entry 3). Again, changing the precatalyst from Mn-MACHO^PPh_2_^ to the ^i^Pr analogue (complex 4) led to a higher conversion (86%) as well as a higher selectivity towards carbamate (80% yield, entry 4). Considering the advantages associated with the catalysts based on earth-abundant metals,^33^ we conducted a few more optimization studies using the Mn-MACHO^iPr_2_^ complex 4. Changing the reaction solvent from toluene to THF did not have much effect on the conversion and selectivity (entry 5) whereas lowering the temperature from 150 to 130 °C dropped the conversion of formamide (63%) and yield of the carbamate (48%, entry 6). Interestingly, changing the amount of base had a more drastic effect on the reaction outcome. When the KO^*t*^Bu loading was reduced to 1 mol%, keeping the remaining conditions the same, only 31% conversion of formamide was obtained (entry 7) but when the KO^*t*^Bu loading was increased to 10 mol%, almost complete conversion of formamide was obtained but the yield of carbamate was reduced to 34% and the remaining products were found to be urea and amine (entry 8). Changing the base to KOH, K_2_CO_3_, and NaO^*t*^Bu led to excellent conversion of formamide, but poor selectivity of carbamate was obtained (entries 9–11). A few examples of base-mediated carbonylation of amines to formamides have been reported in the literature and therefore it is likely that base could be involved in the reverse reaction *i.e.* the decarbonylation of formamide to amines.^34,35^ Doubling the concentration of the reaction mixture reduced the selectivity of carbamate (entry 12) and when the concentration was reduced to half, the selectivity of carbamate was increased with no formation of *N*,*N*′-dicyclohexylurea. However, a relatively lower conversion of formamide was obtained (entry 13). When the reaction was performed without using any base (entry 14), no consumption of the formamide was observed suggesting the crucial role of base. To further test the role of base, the deprotonated complex (5) was independently synthesized and tested in catalysis. Interestingly, the selectivity of the reaction towards carbamate was almost 100% and the formation of urea derivative was not detected. However, the conversion of formamide and yield of carbamate was very low −33%, and 28%, respectively (entry 15).

**Table 1:** Optimization of catalytic conditions for the dehydrogenative coupling of formamide and cyclohexanol^a^

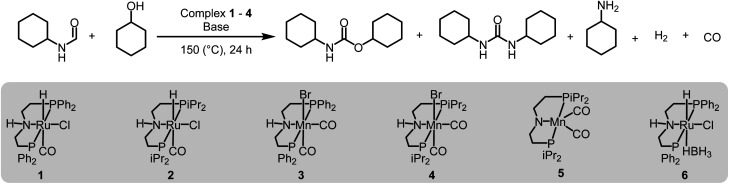						
Entry	Complex	Base	Solvent	Formamide conversion (%)	Carbamate yield^b^ (%)	Urea yield^b^ (%)
1	1	KO^*t*^Bu (4 mol%)	Toluene	89	58	22
2	2	KO^*t*^Bu (4 mol%)	Toluene	91	81	8
3	3	KO^*t*^Bu (4 mol%)	Toluene	62	35	1
4	4	KO^*t*^Bu (4 mol%)	Toluene	86	80	2
5	4	KO^*t*^Bu (4 mol%)	THF	88	78	8
6^c^	4	KO^*t*^Bu (4 mol%)	Toluene	63	48	0
7	4	KO^*t*^Bu (1 mol%)	Toluene	31	26	0
8	4	KO^*t*^Bu (10 mol%)	Toluene	98	34	10
9	4	KOH (4 mol%)	Toluene	89	53	9
10	4	K_2_CO_3_ (4 mol%)	Toluene	78	54	0
11	4	NaO^*t*^Bu (4 mol%)	Toluene	95	75	15
12	1	KO^*t*^Bu (4 mol%)	Toluene (1 mL)	97	44	17
13	1	KO^*t*^Bu (4 mol%)	Toluene (4 mL)	72	61	0
14	4	—	Toluene	0	0	0
15	5	—	Toluene	33	28	0

a Catalytic conditions: formamide (1 mmol), alcohol (1 mmol), solvent (2 mL), 150 °C, 24 h; reactions were carried out in a sealed J. Young's flask. The remaining product (other than carbamate and urea) was detected to be amine by GC-MS.

b Yield was determined by ^1^H NMR spectroscopy using 1,1-diphenylethylene as an internal standard.

c The reaction was carried out at 130 °C.

To probe the effect of gas present in the headspace on selectivity, two experiments were performed – one using 1 atm CO and another one using 1 atm of H_2_ using conditions described in Table 1, entry 4. It was found that both reactions led to a lower conversion of formamide and a lower yield of carbamate. The reaction in the presence of 1 atm of CO led to 24% conversion of formamide and 12% yield of carbamate whereas that in the presence of 1 atm H_2_ led to 12% conversion of formamide and 6% yield of carbamate (Table S1, entries 17 and 18†). No urea derivative was observed in either case.

These optimization studies suggest the following: (a) the selectivity of the reaction depends on various factors – catalyst, base (amount and type), and solvent (type, amount). (b) ^i^Pr substituents on phosphine in case of Ru-MACHO or Mn-MACHO leads to higher selectivity of carbamate derivatives. (c) Increasing the amount of base (KO^*t*^Bu) and using ruthenium-based pincer catalyst instead of manganese analogues favors the decarbonylation process.

We envisioned that the knowledge and understanding gained from the aforementioned catalytic studies could be utilized to develop new methodologies to make ureas, polyureas, and poly(urea-urethanes) from (di)formamides and diol feedstocks. Thus, a range of formamide substrates were studied for the synthesis of urea derivatives directly from formamides. Based on our optimisation reactions (Table S2, see ESI†), the conditions used for this set of reactions correspond to that of Table 1, entry 1, which showed a high conversion of formamide and the best selectivity towards urea production among all the studied conditions (Table 1). The use of *N*-octylformamide resulted in a moderate yield of dioctylurea (53%, Table 2, entry 1). Following this, *N*-cyclohexylformamide (entry 2) and formanilide were used as substrates, the former resulting in a yield of 37% (entry 3) and the latter only 5% yield of the corresponding urea derivative. In the case of formanilide, the only other major product that is observed in the GC-MS and NMR is aniline, suggesting that aromatic formamides are more prone to decarbonylation. Furthermore, *N*-benzylformamide (entry 4), 4-fluorobenzylformamide (entry 5), 1-phenylethylformamide (entry 6) and *N*-methylformamide (entry 7) were used as substrates, all resulting in moderate to high yields of corresponding urea derivatives as described in Table 2. Interestingly, performing a control experiment by heating formanilide in the presence of 50 mol% KO^*t*^Bu (without using any metal catalyst) did not lead to the formation of any urea derivative but did lead to a 32% conversion of formanilide to aniline. This suggests that the metal catalyst is needed for the dehydrogenation of formamide which will eventually lead to the formation of a urea derivative. Furthermore, when a control reaction was performed by heating *N*-benzylformamide without any metal catalyst or base, no conversion was observed and *N*-benzylformamide was completely recovered (ESI, Fig. S176b†) suggesting the significance of transition-metal catalyst and base in the transformation. However, when using complex 5, under base-free reaction conditions without using any alcohol, *N*-benzylformamide, and formanilide led to the formation of *N*,*N*′-dibenzylurea (17% yield) and *N*,*N*′-diphenylurea (16%), respectively suggesting the important role of base in yield and selectivity (ESI, Fig. S176c and d,† respectively).

**Table 2:** Synthesis of urea derivatives from formamides^a^

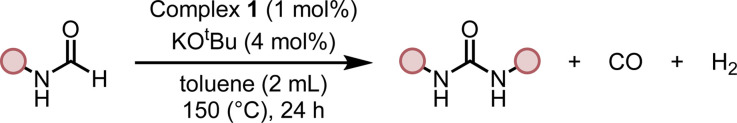			
Entry	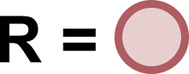	Conversion (%)	Yield (%)
1	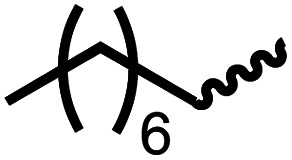	65	53 (51)
2	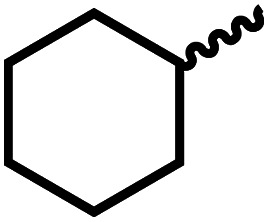	39	37
3^b^	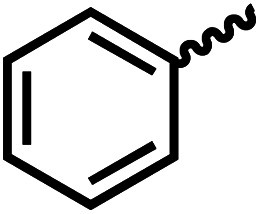	33	5
4	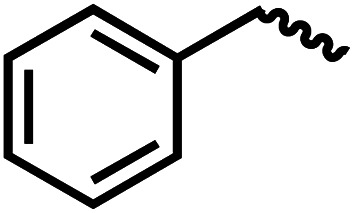	84	74 (68)
5	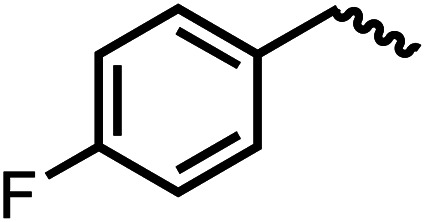	97	88 (83)
6	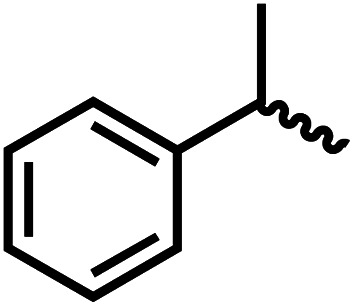	74	70 (63)
7	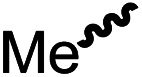	54	46
8^c^	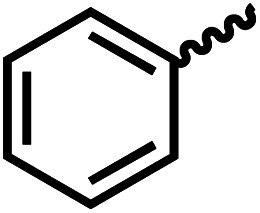	32	0

a Catalytic conditions: formamide (1 mmol), complex 1 (0.01 mmol), KO^*t*^Bu (0.04 mmol), toluene (2 mL), 24 hours, 150 °C. Conversion and yields are estimated by ^1^H NMR spectroscopy using 1,3,5-trimethoxybenzene (0.33 mmol) as an internal standard. Numbers in parentheses are isolated yields.

b The major product was determined to be aniline.

c The reaction was conducted in the presence of 50 mol% KO^*t*^Bu without using ruthenium catalyst, the only product detected was found to be aniline.

Following the successful dehydrogenative (and decarbonlyative) coupling of a variety of aliphatic formamides, the same process was applied to various diformamides to form polyureas. Similar to the urea derivatives, aliphatic diformamides were successfully coupled to form polyureas. However, no formation of polyurea was observed in the case of 1,4-phenylenediformamide. When *N*,*N*-(1,8-octanediyl)-diformamide was used as a substrate, 83% of a solid product was isolated (Table 3, entry 1) which was found to have a *M*_n_ (number average molecular weight) of 2949 Da and a PDI (polydispersity index) of 1.5 as measured by the gel permeation chromatography. Further characterisation by NMR and IR spectroscopy, as well as MALDI-TOF mass spectrometry confirmed that the formed polymer is a polyurea as mentioned in Table 3. The melting temperature and glass transition temperatures were found to be 222 °C and 75 °C as estimated by the Differential Scanning Calorimetry (DSC, Table 3, entry 1). The decomposition temperatures (*T*_d_) was estimated using TGA analysis as the temperature of 5% weight loss and was found to be 298 °C. Comparatively, *N*,*N*-cyclohexyldiformamide (entry 2) resulted in a polyurea in a much lower yield (26%, *M*_n_ = 1874 Da), in concordance with the mono-formamide analogues of these diformamides in Table 2 (entries 1 and 2). We suspect that the low molecular weight of the polymer in these cases could be a result of poor solubility of polyurea in toluene (that is the reaction solvent) resulting in early product precipitation at a smaller chain length. We therefore used diformamide made from 4,7,10-trioxa-1,13-tridecanediamine which could have higher solubility due to the presence of ethylene glycol linkages (Table 3, entry 3). Indeed, this resulted in a polymer of much larger *M*_n_ (17 384 Da) however the PDI was also found to be high (4.2). Similarly, the diformamide made from 4,9-dioxa-1,12-dodecanediamine (Table 3, entry 4) led to the formation of a polyurea in 95% yield and of high *M*_n_ (15 470 Da) but again the PDI was found to be quite high (7.9). We speculate that the large PDI in the latter two cases is likely because the polymerisation process occurs *via* step growth polymerisation commonly known for condensation polymerisation processes for which higher PDI are a characteristic. The relatively lower PDI in the first two cases is presumably because of polymer precipitation at early stage when the solubility limit reaches. We further expanded the substrate scope using diformamides made from *m*-xylenedimaine, and other aliphatic diamine as described in Table 2, entries 5–8. Of interest is a polyurea made from diformamide that was made from Priamine which is considered a renewable diamine. Polyurea of a high molecular weight, 674 kDa which was higher than our calibration curve (up to 55 000 Da) was obtained in this case (entry 8).

**Table 3:** Synthesis of polyureas from diformamides^a^

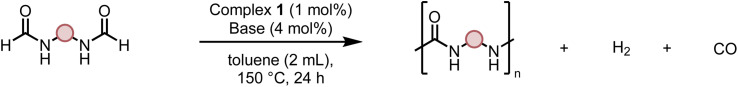							
Entry	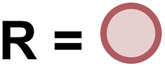	Yield (%)	*M*_n_ (Da)^b^	PDI	*T*_m_ (°C)	*T*_g_ (°C)	*T*_d_ (°C)
1	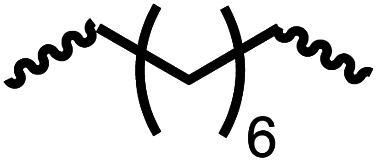	83	2949	1.5	222	75	298
2	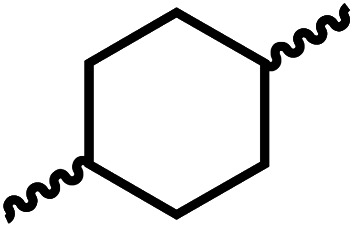	26	1874	1.4	NA	44	258
3	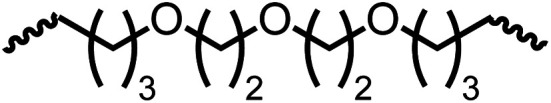	52	17 384	4.2	114	−12	299
4	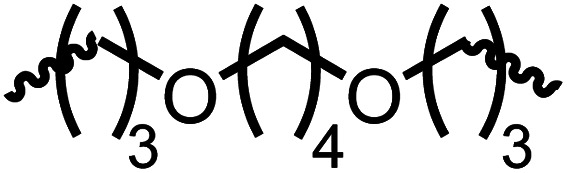	95	15 470	7.9	134	39	267
5^c^	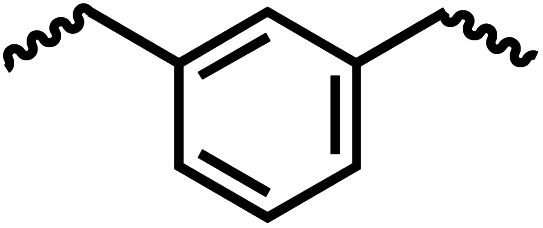	83	597	—	234	NA	228
6^c^	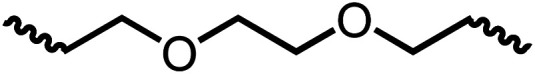	88	23 063	—	94	−3.8	236
7^c^	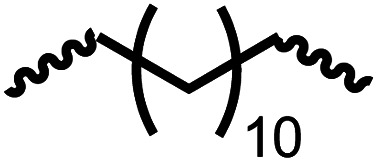	93	1086	—	193	NA	311
8^d^	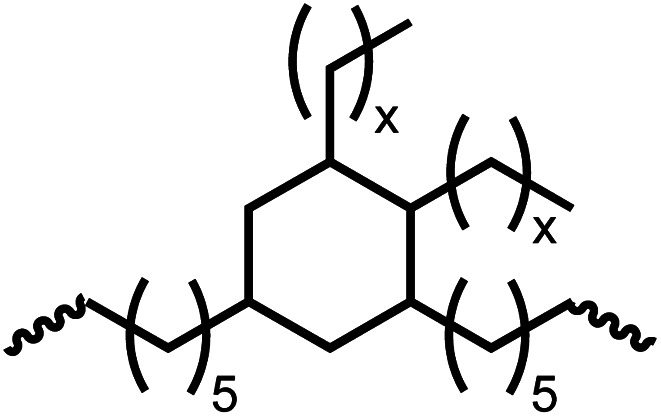	38	>55 000^e^	1.28	NA	NA	291

a Catalytic conditions: diformamide (1 mmol), catalyst (0.01 mmol), KO^*t*^Bu (0.04 mmol), toluene (2 mL), 24 hours, 150 °C.

b Yields are isolated yields. *M*_n_ and PDI were estimated by the GPC. *T*_m_ = melting temperature, *T*_g_ = glass transition temperature, *T*_d_ = decomposition temperature.

c The *M*_n_ for entries 5–7 were calculated by end-group analysis using ^1^H NMR spectroscopy.

d Due to difficulty in separating starting material from product, at the end of the 24 h reaction, the starting material and product were isolated then allowed to react under standard reaction conditions for 48 h.

e *M*_n_ of entry 8 is beyond the range of the calibration curved (estimated to be 674 034 Da).

Having demonstrated the synthesis of polyureas from the self-coupling of diformamides, we paid attention to the coupling of diformamides and diols aiming to make polyurethanes. However, coupling of *N*,*N*-(1,8-octanediyl)diformamide with 1,4-cyclohexanediol using Ru-MACHO complex 1 (1 mol%), and KO^*t*^Bu (4 mol%) in toluene at 150 °C for 24 h led to the isolation of a material in 72% yield that contained both urea and urethane linkages in 84 : 16 ratio as per the ^1^H NMR spectroscopy (Table 4, entry 1). Further characterisation by NMR, and IR spectroscopy as well as MALDI-TOF mass spectrometry confirmed the isolated material to be a poly(urea-urethane). Analysis of the polymer by GPC showed the molecular weight (*M*_n_) to be 2827 Da and PDI to be 1.7. As described in Table 1 (entry 1 and 4), the manganese MACHO^iPr^ complex 4 was more selective to the formation of carbamate than urea. We therefore studied the coupling of *N*,*N*-(1,8-octanediyl)diformamide with 1,4-cyclohexanediol using Mn-MACHO^iPr^ complex 4 instead of complex 1 keeping the remaining conditions the same. However, a similar polymer formed (in terms of urea/urethane linkages as well as its molecular weight and thermal properties) although in a relatively lower yield (Table 4, entry 2). Testing this methodology for the coupling of *N*,*N*′-(cyclohexane-1,4-diyl)diformamide and 1,4-cyclohexanediol using complex 1 also led to the formation of poly(urea-urethane) of *M*_n_ 1316 Da and PDI 1.1 in 70% yield (Table 4, entry 3). The glass transition temperatures (*T*_g_) of these polymers were found to be in the range of 37–44 °C whereas the decomposition temperatures (*T*_d_) were found to be in the range of 285–298 °C.

**Table 4:** Synthesis of poly(urea-urethanes) from diformamides and diols^a^

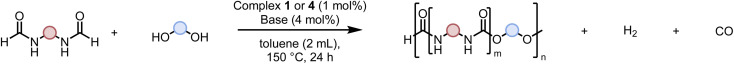										
Entry	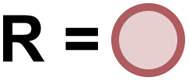	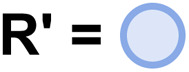	Catalyst	Yield (%)	R : R′	*M*_n_ (Da)	PDI	*T*_m_ (°C)	*T*_g_ (°C)	*T*_d_ (°C)
1^c^	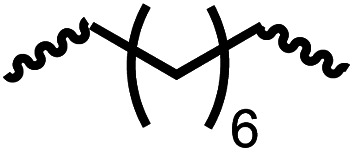	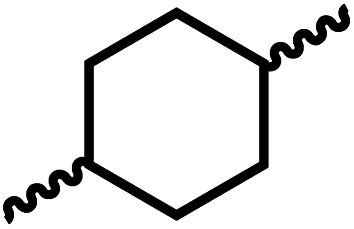	1	72	84 : 16	2827	1.7	192	37	290
2^d^	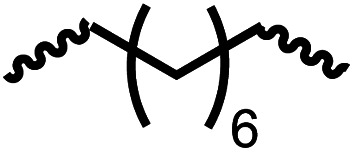	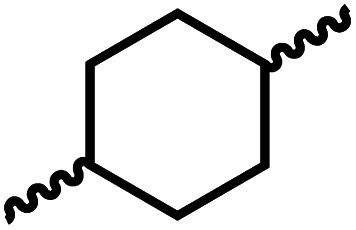	4	46	88 : 12	1776	1.3	187	40	285
3	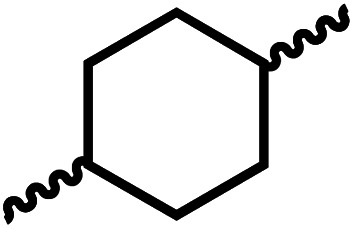	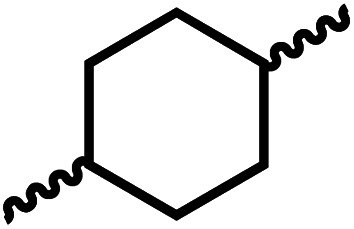	1	70	85 : 15	1316	1.1	NA	44	298
4^b^	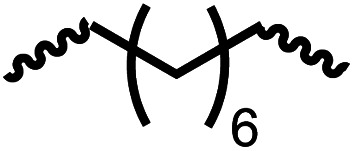	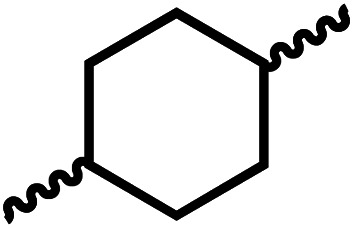	4	37	74 : 26	1669	—	72	—	181

a Catalytic conditions: diformamide (1 mmol), catalyst (0.01 mmol), KO^*t*^Bu (0.04 mmol), toluene (2 mL), 24 hours, 150 °C.

b Yields are all given as isolated yields. *M*_n_ and PDI were estimated by the GPC. *T*_m_ = melting temperature, *T*_g_ = glass transition temperature, *T*_d_ = decomposition temperature. R : R′ represent the ratio of urea/carbamate linkages.

c The reaction was carried out under 1 bar H_2_.

d The *M*_n_ was determined by end group analysis using ^1^H NMR spectroscopy.

We envisioned that since the polymer precipitates out at the end of the reaction whereas the catalyst presumably remains soluble in the reaction solvent, presents opportunities for catalyst recycling studies. Indeed, transferring the soluble part of the reaction mixture at the end of the catalytic coupling of *N*,*N*-(1,8-octanediyl)-diformamide conducted under the standard reaction condition (Table 3, entry 1) to another J. Young's flask containing *N*,*N*-(1,8-octanediyl)-diformamide (1 mmol) and KO^*t*^Bu (0.04 mmol, 4 mol%) led to the isolation of a similar polyurea (characterised by NMR and IR spectroscopy) in 85% yield (Table 5). Repeating the same process for the second recycling step led to the formation of polyurea in 83% yield whereas the yield dropped to 36% when the catalyst recycling was conducted for the third time. Although the yield remained constant across the first three cycles, we surmised that the kinetics of the reaction were likely changing. Consequently, the reaction was repeated for a lower reaction time (6 h) and with double the amount of catalyst and base (2 mol% and 8 mol%, respectively) to observe if the reaction was slowing. It was found that this was indeed the case, with the yield of polyurea obtained at the end of each cycle dropping from 37% to 7% to 0% for each respective cycle.

**Table 5:** Catalyst recycling study for polyurea synthesis^a^

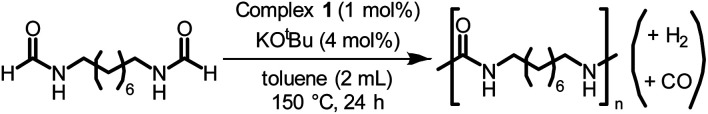				
Experiment	Standard	1st recycling	2nd recycling	3rd recycling
Yield^b^ (%)	84	85	83	36
Yield^b^^,^^c^ (%)	37	7	0	—

a Catalytic conditions: diformamide (1 mmol), catalyst (0.01 mmol), KO^*t*^Bu (0.04 mmol), toluene (2 mL), 150 °C and 24 h.

b Yields are given as isolated yields.

c The reaction was carried out with 0.02 mmol catalyst, 0.08 mmol KO^*t*^Bu and for 6 h.

**Fig. 2:**
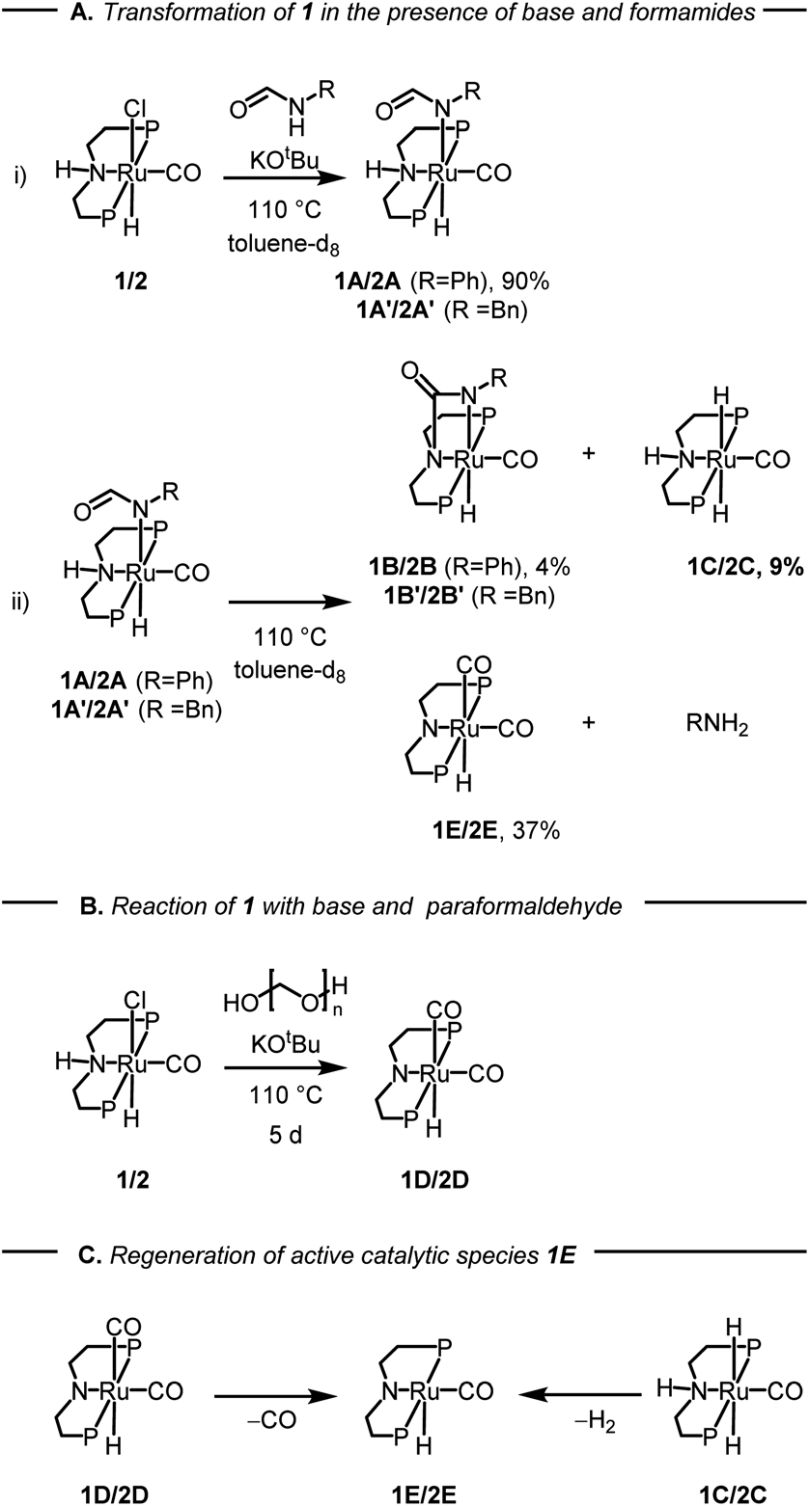
Studies on the reactivity of formamides with complexes 1 or 2 and KO^*t*^Bu, where P = PPh_2_ for complex 1 and P = P^i^Pr_2_ for complex 2 and related species. The yields given are in relation to complex 1A after 1 d in (i) and 7 d in (ii). Further yields can be found in the ESI (Table S3†).

**Fig. 3:**
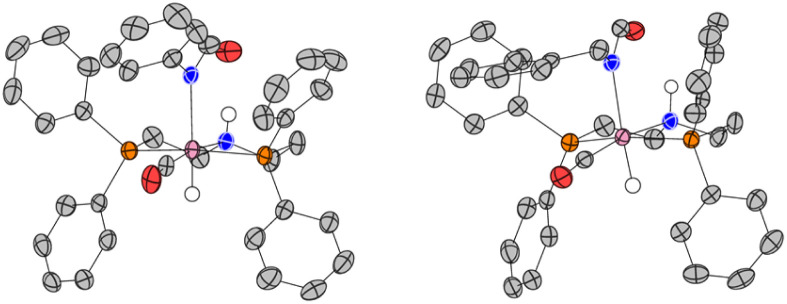
Single-crystal X-ray structures of the asymmetric unit cell of complex 1A (left), and 1A′ (right). ORTEP diagrams plotted at 50% probability level. Selected hydrogen atoms have been omitted for clarity.

After studying the catalytic reactions, we moved our attention towards understanding the mechanism of the dehydrogenation and decarbonylation processes. Although the dehydrogenation of formamide to isocyanate has been reported,^22,29^ decarbonylation of formamide using an organometallic catalyst is not known to the best of our awareness. The only example of the decarbonylation of formamide has been reported by Maron and Zhou using La[N(SiMe_3_)_2_]_3_ catalyst.^36^ We first studied the stoichiometric reaction of activated Ru-MACHO^PPh_2_^ complex with formamide to understand the organometallic transformations. Performing the reaction of complex 1 with KO^*t*^Bu (1.2 equivalents) and formanilide or *N*-benzylformamide (1.2 equivalents) in toluene-d_8_ at 110 °C led to the clean formation of new complexes which were characterised to be the N–H activated complexes of corresponding formamides 1A, and 1A′ (Fig. 2A(i)). The quantitative formation of 1A took only 10 minutes whereas the formation of 1A′ was relatively slower and took one day to achieve the quantitative yield. The complexes were characterised by NMR spectroscopy, mass spectrometry, and single-crystal X-ray diffraction (Fig. 3). Complex 1A co-crystallised with formanilide has also been reported by Nova and Bernskoetter.^37^ Further heating the toluene solution of the *in situ* formed complexes 1A at 110 °C led to its slow conversion to mainly three species two of which were identified to be complexes 1B^32^ which is an isocyanate-coordinated complex, and 1C^38^ which is a ruthenium dihydride complex based on previous reports.

The third one was identified to be the ruthenium dicarbonyl complex 1D based on a recent report by Leitner.^39^ Over time, the concentration of complexes 1A and 1B was found to decrease with complete consumption in four days whereas the concentration of complexes 1C and 1D were found to increase until seven days (see Section 4, ESI†). This reaction was repeated using complex 2 in comparison to complex 1 (ESI, Section 4 and Table S3†). Interestingly, complex 2 led to more dehydrogenation and less decarbonylation in comparison to complex 1 which is consistent with our optimisation studies where complex 1 is more selective towards the formation of urea.

Based on this experiment we suggest that under our reaction conditions, complex 1A gets dehydrogenated to form the coordinated isocyanate complex 1B and the ruthenium dihydride complex 1C (Fig. 2A(ii)). Concomitantly, complex 1A also gets decarbonylated to form the ruthenium dicarbonyl complex 1D and aniline (Fig. 2A(ii)). In support of the decarbonylation step, analysing the reaction mixture (after two days of heating) by GC-MS showed the formation of aniline alongside formanilide (see ESI, Fig. S166 and S167†). Additionally, analysis of the reaction over time by the ^1^H NMR spectroscopy showed the disappearance of formanilide (*δ* 8.62 ppm) and appearance of H_2_ (*δ* 4.54 ppm, see ESI Fig. S159†).

Having developed some understanding of the organometallic transformations, we carried out DFT computation to elucidate pathways of dehydrogenation and decarbonylation. Hydrogenation and dehydrogenation reactions involving MACHO-type complexes have been studied by DFT computation in much detail.^40–43^ We have previously carried out DFT computation where we proposed a pathway for the dehydrogenation of formamides to isocyanates catalysed by the Mn-MACHO^iPr_2_^ complex (4).^22^ Using the same level of DFT as in our previous studies,^44^ and informed by our experimental findings, we have computed key reaction steps leading to the main products shown in Fig. 2. We have used active catalysts derived from Ru-MACHO^Ph_2_^ (1) and Mn-MACHO^iPr_2_^ (4) along with *N*-methylformamide (NMF) as a model substrate. Selected steps have also been recalculated for formanilide (*N*-phenylformamide, NPF) as substrate (catalyst derived from 1 only).

**Fig. 4:**
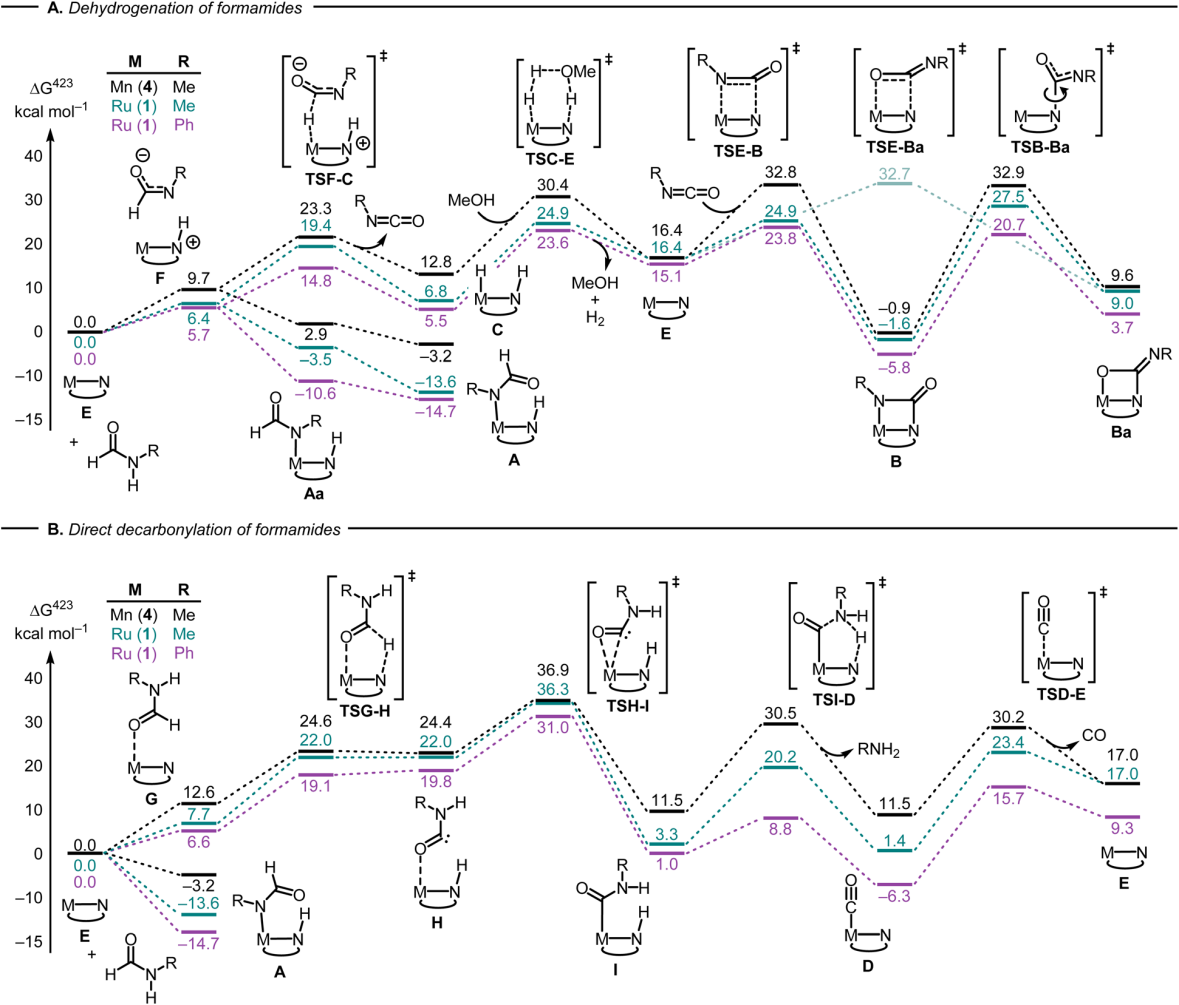
Selected steps involved in the dehydrogenation (A) and direct decarbonylation (B) of formamides using catalysts derived from 1 and 4 (PBE0-D3(BJ)_PCM(THF)_/def2-TZVP//RI-BP86_PCM(THF)_/def2-SVP level). For the ruthenium version, complexes A, C and E are identical with 1A, 1C, and 1E (Fig. 2) respectively.

The first step in the dehydrogenation of formamide through the active catalyst (labeled E in Fig. 4A) is the formation of an NH-activated zwitterionic intermediate (F in Fig. 4A). The second H-transfer *via*TSF-C releases the isocyanate and affords the hydrogenated catalyst (C in Fig. 4A). Under catalytic turnover conditions, the latter can either liberate H_2_ (*via* a solvent-or substrate-assisted transition state, TSC-E in Fig. 4A) or transfer it to other substrates, *e.g.* fresh formamide (see below), regenerating catalyst E. This can then add the previously produced isocyanate to form the metallacyclobutanone species B.

Alternatively, the zwitterionic intermediate F can rearrange to form the *N*-formamide complex A. In fact, “slippage” of the formamide nitrogen from the hydrogen bond with the N-protonated MACHO backbone in F to the metal centre, affording rotamer Aa, is so facile that the corresponding transition states, after applying the thermodynamic corrections (see ESI†) are lower in free energy than species F (not shown in Fig. 4A). Rotation about two single bonds in Aa (arguably very facile as well) affords A, which is stabilized by an intramolecular H-bond between the protonated MACHO-N atom and the carbonyl oxygen. A transition state leading from a rotamer of 1A to 1B (R = Ph) through H_2_ elimination could be located but was excessively high in energy (Δ*G*^423‡^ close to 60 kcal mol^−1^ relative to 1A, see Fig. S199 in the ESI†). Complexes B are computed to be accessible through decoordination of amide from A and subsequent dehydrogenation of the amide, with the highest barrier of, *e.g.* Δ*G*^423‡^ = 38.5 kcal mol^−1^ between 1A and 1TSE-B (numbers in pink in Fig. 4A).

Following recent findings from the Sanford group that isocyanates can add to Mn-MACHO complexes across both the C=N double bond (forming complexes B), and the C=O double bond,^32^ we have computed the resulting metallaoxetanes (Ba) and selected transition states leading to them. Metallacyles B and Ba can interconvert through rotation along the C–N(MACHO) bond *via* transition states TSB-Ba (Fig. 4A). Complexes Ba are significantly higher in energy than their isomers B as also suggested by Sanford,^32^ and the barriers leading from Ba to B are low (especially for the Ru complexes 1, Δ*G*^423‡^ = 17.0–18.5 kcal mol^−1^ between Ba and TSB-Ba). These results therefore rationalize why no traces of complexes Ba can be found under our reaction conditions.

We were also able to characterize a pathway for the direct decarbonylation of formamide substrates leading from the activated catalyst E to free amine RNH_2_ and the metal–CO complexes (such as 1D in Fig. 2). Formation of the latter is predicted to be mildly endergonic for Ru-MACHO^Ph_2_^ (1) and the *N*-methylformamide model (R = Me, Δ*G*^423^ = 1.4 kcal mol^−1^), but noticeably exergonic for formanilide (R = Ph, Δ*G*^423^ = −6.3 kcal mol^−1^), in accordance with the experimental detection of 1D (Fig. 2). This direct decarbonylation involves deprotonation of the aldehyde moiety and the formation of intermediate metal–carbamoyl complexes (I in Fig. 4B). Carbamoyl complexes are well known^45,46^ and some have also been reported to undergo decarbonylation.^46^ Overall barriers for this process relative to free activated catalyst would seem surmountable (*e.g.* Δ*G*^423‡^ = 31.0 kcal mol^−1^ between 1E and 1TSH-I in Fig. 4, R = Ph), however, the accessibility of the amide complexes A, which would be off-cycle intermediates under turnover conditions, would raise the overall energy span to unsurmountable values (*e.g.* to Δ*G*^423‡^ = 45.7 kcal mol^−1^ from 1A to TS1H-I in Fig. 4B, R = Ph).

**Scheme 2:**
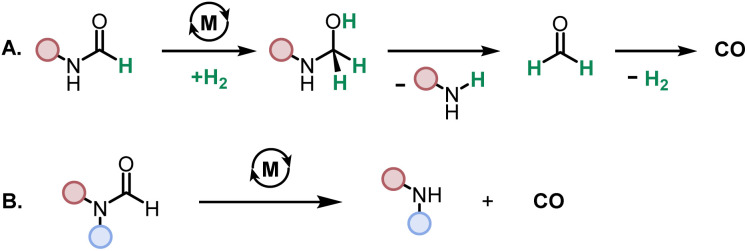
Proposed decarbonylation pathways for primary (A) and secondary (B) formamides.

While direct decarbonylation of primary formamides using MACHO complexes seems unlikely based on these results, decarbonylation of aldehydes, notably formaldehyde, could be possible. There is precedence for such decarbonylation reactions catalyzed by ruthenium complexes.^47^ We theorized that the hydrogenated MACHO catalysts such as 1C (Fig. 2), formed by the dehydrogenation of primary formamide substrates (Fig. 4), could hydrogenate a formamide to give a hemiaminal, which could then decompose into amines and formaldehyde (Scheme 2A). In support of this, monitoring the reaction of formanilide with complex 1A + KO^*t*^Bu as described in Fig. 2A by ^1^H NMR spectroscopy showed the formation of formaldehyde (*δ* 9.58 ppm, Fig. S156, see ESI†).

Hydrogenation of amides to hemiaminals enroute to amines and alcohols is well known using such pincer catalysts.^48,49^ In this study, the formation of a hemiaminal using Ru-MACHO precatalyst 1 is also computed to be facile (see Fig. S197 in the ESI†) and more favorable for formanilide than *N*-methylformamide. We have also traced two possible pathways for the decomposition of the hemiaminal – one assisted with a protic reagent (modelled through methanol) and the second one assisted with KO^*t*^Bu as also reported by Santaballa^50^ (see Fig. S198 in the ESI†). With an overall barrier of around 30 kcal mol^−1^ this decomposition should be readily possible under our reaction conditions. Of particular note is the computed ease of decomposition for the hemiaminal resulting from formanilide upon alcohol deprotonation, consistent with the observation that this substrate shows a high selectivity toward the formation of amine (Table 1, entry 16). The decomposition of hemiaminal to amine and aldehyde has also been proposed to be catalyzed by transition metal (an iron pincer complex) or formamide such as formanilide with a similar energy barrier.^48^

**Fig. 5:**
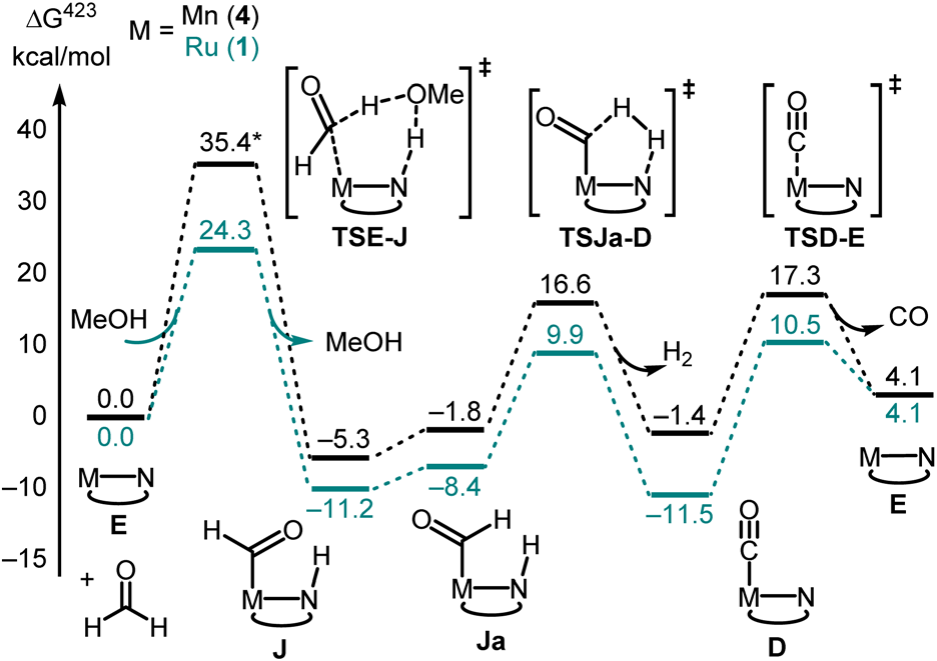
Selected steps involved in the decarbonylation of formaldehyde using catalysts derived from 1 and 4 (PBE0-D3(BJ)_PCM(THF)_/def2-TZVP//RI-BP86_PCM(THF)_/def2-SVP level). *The structure of TSE-J does not contain protic assistance from MeOH for Mn-MACHO (4) as this was not found to stabilise the TS.

Decarbonylation of formaldehyde with the Ru-MACHO catalyst 1 is predicted to be facile (Fig. 5), with an overall barrier as low as Δ*G*^423‡^ = 24.3 kcal mol^−1^ with assistance from a protic H-relay (1TSE-Ja in Fig. 5) and a large driving force for the formation of the observed carbonyl complex 1D (Δ*G*^423^ = −11.5 kcal mol^−1^). These predictions are borne out by a subsequent control experiment where complex 1D is formed from the reaction of complex 1/KO^*t*^Bu with paraformaldehyde (Fig. 2B). The analysis of gas by GC-TCD (Gas Chromatography-Thermal Conductivity Detector) produced in this reaction showed the presence of CO and H_2_ confirming that the precatalyst 1 is capable of decarbonylating formaldehyde under the reaction condition as also reported by Leitner.^39^

In a control experiment, we tested the reactivity of *N*-methylformanilide under the standard reaction condition using complex 1 (as mentioned in Table 2). Since the secondary formamide does not contain N–H proton and cannot dehydrogenate to make isocyanate as in the case of primary formamide (*e.g.* formanilide), there is no presence of metal-hydride species for the hydrogenation of formamide to make a hemiaminal intermediate and therefore the decarbonylation as per the proposal described in Scheme 2A and Fig. 5 should not be possible. However, we observed almost complete decarbonylation of *N*-methylformanilide and *N*-methyl aniline was observed in almost quantitative yield (ESI, Section 4.4 and Fig. S176g†). We suggest that the reaction in this case undergoes *via* the direct decarbonylation route (Scheme 2B) proposed in Fig. 4B. Since the secondary formamide cannot form complexes analogous to 1A, the barrier for the decarbonylation would be possible to achieve under the reaction condition (150 °C).

Overall, the computations afford plausible mechanistic pathways that are in agreement with the observed products. Comparing Mn and Ru complexes it appears that the latter are consistently lower in energy relative to the respective activated complex 1E than the former (relative to 4E). The slightly different phosphine ligands on the MACHO backbone (Ph *vs.*^i^Pr side chains) are not expected to be crucial for the energetics, arguably it is the fact that (apart from the pentacoordinate complexes E), Mn-MACHO species have a CO ligand trans to the bound substrates, whereas the Ru-MACHO congeners have a hydride ligand in that position (*cf.* the X-ray strictures of 1A and 1A′ in Fig. 3). The strong trans-effect and -influence of this carbonyl ligand is expected to destabilise the Mn-complexes. The extent of this destabilisation is variable, though (compare black and dark green profiles in Fig. 4 and 5), so that the effect on the overall rate-limiting barriers is hard to predict beforehand, and either type of catalyst can be active, depending on the particular reaction and the reaction conditions.

Using MeOH as a simple model for cyclohexanol, the overall reactions leading to the observed products (or their simplified models) are computed to be endergonic at our level (see Table S4 in the ESI†). It may thus be expected that the reaction is driven forward through the removal of gaseous byproducts (H_2_ and CO) from the equilibrium mixture, and that product distribution is kinetically controlled. The calculations show significant differences in the energetics between the two aliphatic and aromatic substrates along the pathways (compare for instance pink and green profiles in Fig. 4). Intermediates and transition states derived from formanilide tend to be lower in energy than their aliphatic counterparts. In view of the complicated reaction system with several interlinked catalytic cycles under turnover, however, quantitative prediction of selectivity based on these findings is difficult.

## Conclusion

**Fig. 6:**
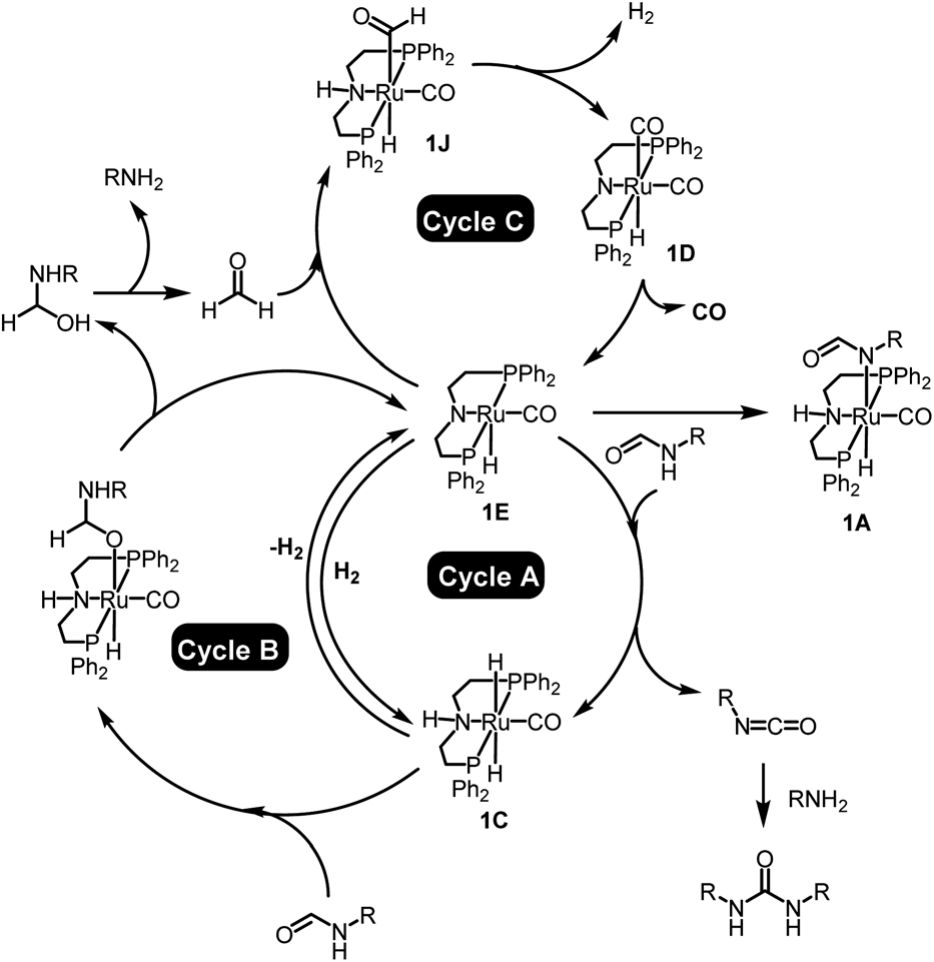
Summary of the mechanism for the dehydrogenation and decarbonylation of formamides catalysed by the Ru-MACHO pincer catalyst.

In conclusion, we have demonstrated new methods for the synthesis of urea derivatives, polyureas, and poly(urea-urethanes). Urea derivatives/polyureas can be synthesized from formamides/diformamides whereas poly(urea-urethanes) can be synthesized from the coupling of diformamides and diols using a Ru-MACHO pincer catalyst. All these processes undergo with the extrusion of H_2_ and CO which in principle can be easily separated/collected from the solution phase and used as a valuable feedstock *e.g.* for syngas. Mechanistic investigations have been conducted using experiments and DFT computation. According to our findings, the transformation of primary formamides to urea derivatives occurs *via* three catalytic cycles as outlined in Fig. 6. In the 1st cycle (A), the activated complex 1E dehydrogenates formamides to isocyanates with the concomitant formation of ruthenium dihydride complex 1C that can reversibly release H_2_ to regenerate the active species 1E. In cycle B, the ruthenium dihydride complex 1C can hydrogenate formamide to form a hemiaminal that can off-metal decompose to amine and formaldehyde. Amine thus formed in cycle B can react with the isocyanate formed in cycle A to form a urea derivative. Formaldehyde produced from cycle B can subsequently enter into cycle C by reacting with the active species 1E and forming a ruthenium formyl intermediate (1J) that can dehydrogenate to form the ruthenium dicarbonyl species 1D. Elimination of CO from complex 1D regenerates the active species 1E.

## Author contributions

A. K. conceived the study. The synthetic chemistry was carried out by J. L. and N. D. M. The DFT computation was performed by A. S. G. and M. B. The manuscript and ESI† were prepared by the contribution of all the authors. All authors agreed on the finalised version of the manuscript.

## Conflicts of interest

There are no conflicts to declare.

## Supplementary Material

SC-015-D4SC03948C-s001

SC-015-D4SC03948C-s002

## Data Availability

Crystallographic data for complex 1A and 1A′ have been deposited at the CCDC under CCDC 2356158 and CCDC 2356150,† respectively. The datasets supporting this article have been uploaded as part of the ESI.† The raw research data supporting this publication can be accessed at DOI: https://doi.org/10.17630/00c4273b-ec5d-45d0-998a-c6dec75789ae.
