# Supplementary material for: Exploiting decarbonylation and dehydrogenation of formamides for the synthesis of ureas, polyureas, and poly(urea-urethanes)
Source: Chem Sci. 2024 Sep 5;15(40):16594–604. doi: 10.1039/d4sc03948c (PMC11411599; doi:10.1039/d4sc03948c)
Supplement: SC-015-D4SC03948C-s001 [file SC-015-D4SC03948C-s001.pdf]

Supplementary Information (SI) for Chemical Science.
This journal is © The Royal Society of Chemistry 2024

Supplementary Information

Exploiting Decarbonylation and Dehydrogenation of Formamides for the Synthesis of Ureas, Polyureas, and Poly(urea-urethanes)

James Luk, Alister. S. Goodfellow, Nachiket Deepak More, Michael Bühl,* Amit Kumar*

EaStCHEM, School of Chemistry, University of St. Andrews, North Haugh, St. Andrews, KY169ST, UK.

Table of Contents

1. General information **...... 3**

2. General Procedures **...... 4**

2.1. General procedure for the synthesis of diformamide substrates[1] 4

2.2. General procedure for the synthesis of carbamates and urea derivatives 4

2.3. General procedure for synthesis of polyureas 4

2.4. General procedure for synthesis of polyurea(urethanes) 4

3. Characterisation data **...... 5**

3.1. Characterisation data for carbamate synthesis 5

3.1.1. ^{1}H NMR data for carbamate synthesis 5
3.1.2. GC-MS data for carbamate synthesis 15

3.2. Characterisation data for urea synthesis 16

3.2.1. Characterisation data for isolated urea compounds 16
3.2.2. Crude ^{1}H NMR spectra for the synthesis of urea derivatives 17
3.2.3. Crude GC-MS data for urea synthesis 22
3.2.4. GC-TCD data 29
3.2.5. ^{1}H and ^{13}C{^{1}H} NMR of isolated urea compounds 29
3.2.6. GC-MS spectra of isolated urea compounds 33
3.2.7. IR spectra of isolated urea compounds 35

3.3. Characterisation data for polyurea synthesis 37

3.3.1. Characterisation data for isolated polyurea compounds 37
3.3.2. ^{1}H NMR and ^{13}C{^{1}H} NMR spectra for polyurea synthesis 39
3.3.3. IR spectra for polyurea synthesis 47
3.3.4. DSC spectra for polyurea samples 51
3.3.5. TGA spectra for polyurea samples 55
3.3.6. MALDI TOF spectra for polyurea samples 59
3.3.7. GPC data of the isolated polyurea 61

3.4. Characterisation data for polyurea(urethane) synthesis 63

3.4.1. ^{1}H NMR and ^{13}C{^{1}H} NMR spectra for polyurea(urethane) synthesis 63
3.4.2. IR spectra for polyurea(urethane) synthesis 68
3.4.3. DSC data for polyurea(urethane) 69
3.4.4. TGA data for polyurea(urethane) 71
3.4.5. MALDI-TOF spectra for polyurea(urethane) 73
3.4.6. GPC traces of polyurea(urethane) 74

3.5. Characterisation data for polyurea from recycling studies 76

3.5.1. ^{1}H NMR and ^{13}C{^{1}H} NMR data for polyurea from catalyst recycling studies 76
3.5.2. IR data for polyurea from catalyst recycling studies 80

4. Mechanistic Investigations **...... 82**

4.1. Stoichiometric reaction of complex 1 with base and formamides 82

4.2. Synthesis of complexes 1a, 1b and 1d 89

4.3. Characterisation data for complexes 1a, 1a' and 1d 89

4.3.1. NMR data 89
4.3.2. Crystallographic data 92

4.4. Control Experiments 95

4.5. Characterisation data for control experiments 96

4.5.1. ^{1}H NMR data 96
4.5.2. GCMS data 99
4.5.3. GCTCD data 103

5. Computational Details *103*

5.1. General Considerations **103**

5.2. Driving forces of catalysed reactions **105**

5.3. Direct decarbonylation of amide **106**

5.4. Production and decomposition of hemiaminals **107**

5.5. Computational raw data **108**

6. References *226*

1. General information

All manipulations were carried out under an inert atmosphere of argon or nitrogen using standard Schlenk and glove-box techniques unless specified. All chemicals (e.g. diamines, diols, formic acid, etc) including complex **1** and **5** were purchased from Sigma-Aldrich, Thermofisher, TCI or Strem, unless stated otherwise, and used as received. N,N'-(1,8-octanediyl)diformamide, N,N'-(1,4-cyclohexyl)diformamide were synthesised according to literature procedure.[1] Complexes **2**,[2] **3**,[3] and **4**[3] were synthesised according to literature reports. THF and toluene were dried by a solvent purification system and degassed using freeze-pump-thaw technique.

All NMR spectroscopic experiments were carried out at 298 K using a Bruker Avance II 400 400 MHz or a Bruker AV-III HD 500 MHz, unless stated otherwise, and reported in ppm (δ). NMR spectroscopy abbreviations: b - broad, s – singlet, d – doublet, t – triplet, q – quartet, m – multiplet. NMR spectra of polymer samples were taken in *d*-TFA with a D_2O capillary inserted as a reference for ^1H and $^{13}C\{^1H\}$ NMR spectra. Error in the estimation of conversion and yields is ±5%.

IR spectra were recorded using a MIRacle™ single reflection horizontal ATR accessory from Pike (ZnSe single crystal). TGA was performed using an Stanton Redcroft STA-780 Series Thermal Analyser between 30–600°C at a heating rate of 10 °C/min under a flow of nitrogen gas (25 mL/min). DSC analyses were performed using a Netzsch DSC204 between –50–240 °C or –50–280 °C at a heating rate of 10 °C/min under a flow of nitrogen gas (20 mL/min) after an initial heat/cool cycle (25–120 °C at 10 °C/min with a 20 minute isothermal at 120 °C) to remove the thermal history of the sample.

GC-MS samples were prepared in HPLC grade DCM and run on an Agilent 8860 GC system coupled to an Agilent 5977B EI instrument. Gel permeation chromatography (GPC) was performed on an Agilent 1260 InfinityLab II GPC fitted with a refractive index (RI) detector (35 °C). The single (plus guard column) Agilent PolarGel column setup was contained within an oven (35 °C). Dichloroacetic acid/chloroform (20/80) was used as the eluent at a flow rate of 1.0 mL min^{-1}. Samples were dissolved in the eluent (2.0 mg mL^{-1}), filtered (0.2 μm pore size) and run immediately. The calibration was conducted using a series of monodisperse polystyrene (M_n = 578 - 457000 g mol^{-1}) standards obtained from Agilent Technologies.

For the preparation of the MALDI samples, polyureas were dissolved in neat TFA and further diluted with 0.1% TFA (prepared in HPLC grade water). 0.5 µL of the resulting solution was applied to a stainless steel MALDI target plate, 0.5 µL of the matrix was co-spotted and allowed to dry. Matrix was either 2,5- dihydroxybenzoic acid or alpha-cyano-4-hydroxycinnamic acid prepared at 10 mg/mL in 50:50 acetonitrile: 0.1% TFA. MALDI data was acquired using a 4800 MALDI TOF/TOF Analyser (Sciex) equipped with a Nd:YAG 355 nm laser. The sample was acquired in positive MS mode between 200 m/z and 4000 m/z in reflector mode or 3000m/z-10000m/z in linear mode. The instrument was externally calibrated in reflector mode using Sciex 6 peptide TOF/TOF calibration mix, and in linear mode with ubiquitin protein.

Single crystals were obtained by vapour diffusion of toluene/hexane. SCXRD was performed and data was obtained by Crystallography Service, School of Chemistry, University of St Andrews. Data was solved using SHELXT[4] direct methods and refined by full-matrix least-squares against F^2 using SHELXL.[5] Crystallographic data is summarised in Table S4. Further experimental and refinement details can be found in the corresponding CIF files. The

supplementary data for the crystallographic data can be found at CCDC 2356158 and CCDC 2356150.

2. General Procedures

2.1. General procedure for the synthesis of diformamide substrates[1]

The desired diamine (10 mmol) was added to ethyl formate (100 mmol) and allowed to stir at 70 °C for 24 h. The solvent was then removed under reduced pressure affording the pure product (>99%).

2.2. General procedure for the synthesis of carbamates and urea derivatives

Synthesis of Carbamates: The desired formamide (1 mmol), the metal complex (0.01 mmol, 1 mol%), and KO^{t}Bu (0.04 mmol, 4 mol%) were weighed (under air) in a J. Young flask containing a stirrer bar and then degassed using three vacuum/nitrogen cycles on a Schlenk line. Alcohol (1 mmol) was added followed by solvent (2 mL), both under nitrogen. The Young's flask was placed in an oil bath to reflux the reaction mixture at 150 °C. After reaction, the contents of the flask were cooled to room temperature, and the lid was slowly opened to measure the gas release. The solid precipitate (presumably consisting of urea was filtered) and 0.25 mmol of diphenylethylene was added to the filtrate as an internal standard to estimate the crude yields/conversion using ^{1}H NMR spectroscopy.

Synthesis of Urea derivatives: The same procedure (as described above) was followed for the synthesis of urea derivatives except no alcohol was added in this case.

2.3. General procedure for synthesis of polyureas

The desired diformamide (1 mmol), precatalyst (0.01 mmol) and KO^{t}Bu (0.04 mmol) were added to a flame-dried 250 mL J. Young flask before being vacuum cycled using argon 3 times. Under argon, degassed toluene (2 mL) was added, and the reaction was heated to the desired temperature (150 °C) and allowed to stir (24 h). The reaction was then allowed to cool to room temperature before the solid off-white precipitate was collected and washed with methanol and dried under vacuum to afford the product.

Procedure for catalyst recycling: The same procedure (as described above) was followed. However, upon completion of the 24 h reaction, the reaction mixture was allowed to cool to room temperature and the reaction solution was transferred by filter cannula to another J. Young flask containing diformamide (1 mmol) and KO^{t}Bu (0.03 mmol) under argon. The reaction was then heated to 150 °C and the same process as described above was repeated a further 2 times after 24 h.

2.4. General procedure for synthesis of polyurea(urethanes)

The desired diformamide (1 mmol), diol (1 mmol), precatalyst (0.01 mmol) and KO^{t}Bu (0.04 mmol) were added to a flame-dried 250 mL Young's flask before being vacuum cycled with argon 3 times. After degassing using vacuum/argon cycles, degassed toluene (2 mL) was added under argon and the reaction was heated to the desired temperature (150 °C) and allowed to stir (24 h). The reaction was then allowed to cool to room temperature from which the solid

off-white precipitate was collected and washed with methanol and dried under vacuum to afford the product.

3. Characterisation data

3.1. Characterisation data for carbamate synthesis

3.1.1. ^{1}H NMR data for carbamate synthesis

Table S1: Full table for optimization of catalytic conditions for the dehydrogenative coupling of formamide and cyclohexanol.[a]

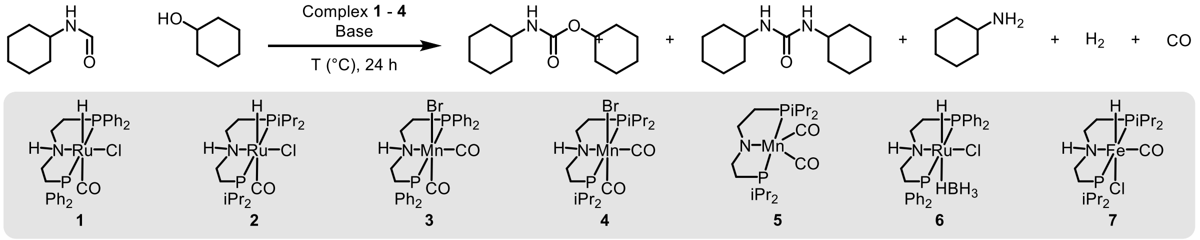

Entry	Complex	Base	Solvent	Formamide Conversion (%)	Carbamate Yield (%)[b]	Urea Yield (%)[b]
1	**1**	KO^{t}Bu (4 mol%)	toluene	89	58	22
2	**2**	KO^{t}Bu (4 mol%)	toluene	91	81	8
3	**3**	KO^{t}Bu (4 mol%)	toluene	62	35	1
4	**4**	KO^{t}Bu (4 mol%)	toluene	86	80	2
5	**4**	KO^{t}Bu (4 mol%)	THF	88	78	8
6[c]	**4**	KO^{t}Bu (4 mol%)	toluene	63	48	0
7	**4**	KO^{t}Bu (1 mol%)	toluene	31	26	0
8	**4**	KO^{t}Bu (10 mol%)	toluene	98	34	10
9	**4**	KOH (4 mol%)	toluene	89	53	9
10	**4**	K_2CO_3 (4 mol%)	toluene	78	54	0
11	**4**	NaO^{t}Bu (4 mol%)	toluene	95	75	15
12	**1**	KO^{t}Bu (4 mol%)	toluene (1 mL)	97	44	17
13	**1**	KO^{t}Bu (4 mol%)	toluene (4 mL)	72	61	0
14	**4**	-	toluene	14	0	0
15	**5**	-	toluene	33	28	0
16	**7**	KO^{t}Bu (4 mol%)	toluene	23	5	0
17[d]	**4**	KO^{t}Bu (4 mol%)	toluene	24	12	0
18[e]	**4**	KO^{t}Bu (4 mol%)	toluene	12	6	0

[a]Catalytic conditions: formamide (1 mmol), alcohol (1 mmol), solvent (2 mL), 150 °C, 24 h; reactions were carried out in a sealed J. Young's flask. The remaining product (other than carbamate and urea) was detected to be amine by GC-MS. [b]Yield was determined by ^{1}H NMR spectroscopy using 1,1-diphenylethylene as an internal standard. [c]The reaction was carried out at 130 °C. [d]The reaction was carried out under 1 bar CO pressure. [e]The reaction was carried out under 1 bar H_2 pressure.

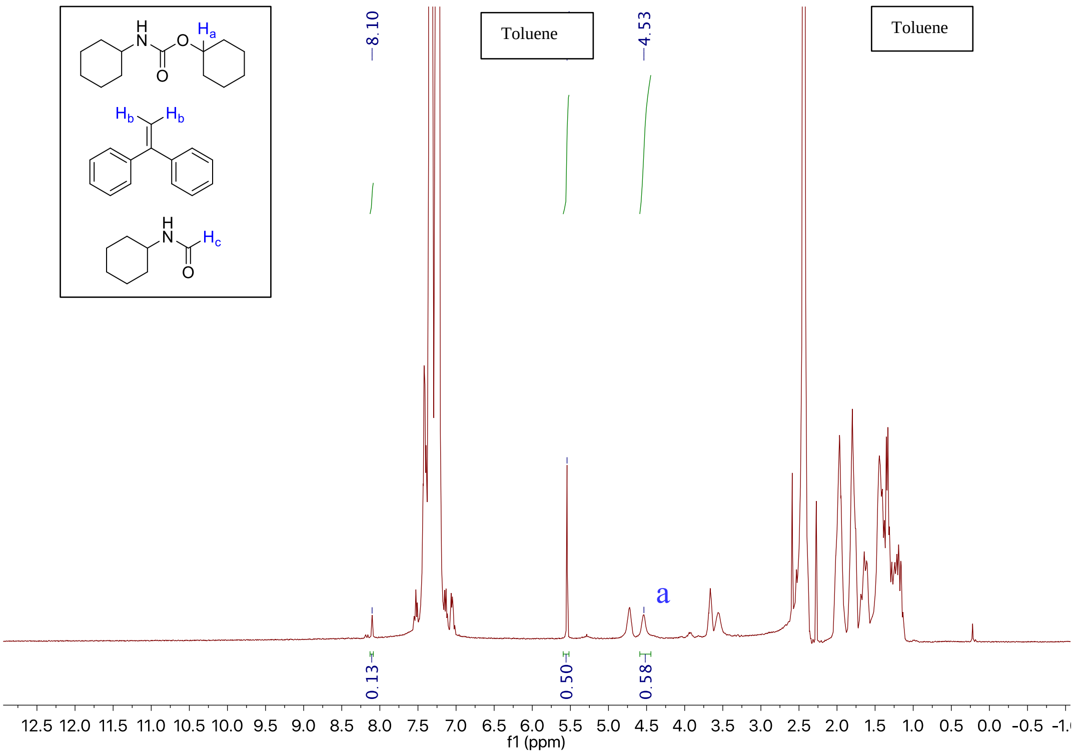

Figure S1: ^{1}H NMR spectrum ($CDCl_3$, 400 MHz, 298 K) of Table 1, entry 1.

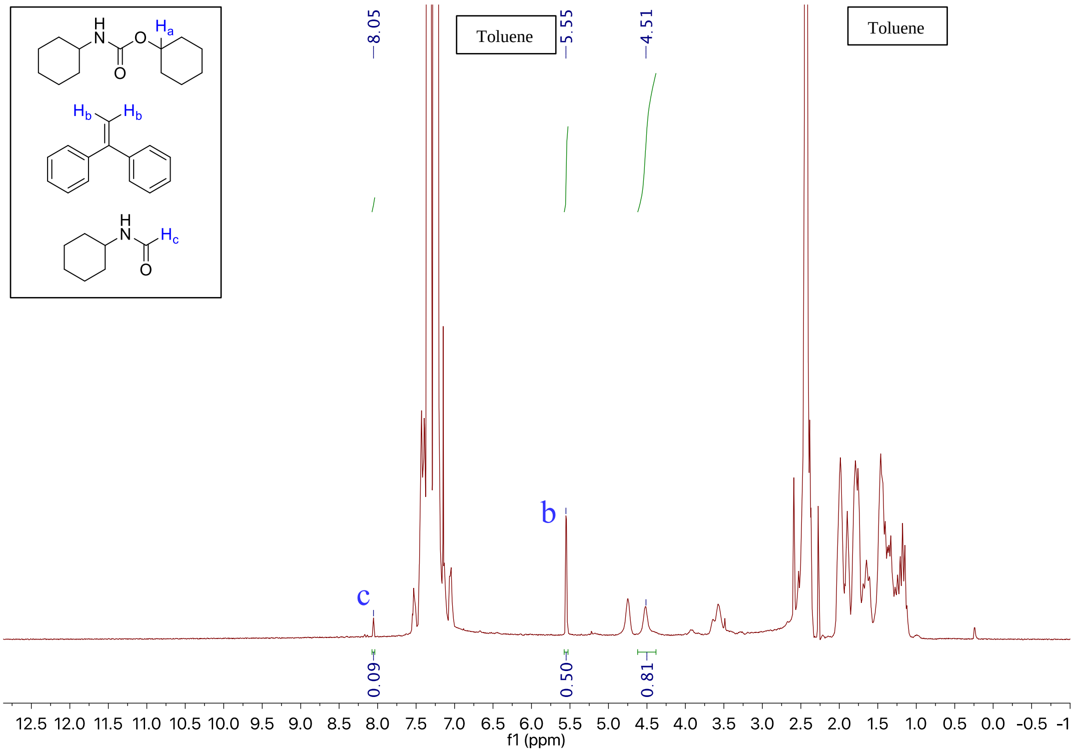

Figure S2: ^{1}H NMR spectrum ($CDCl_3$, 400 MHz, 298 K) of Table 1, entry 2.

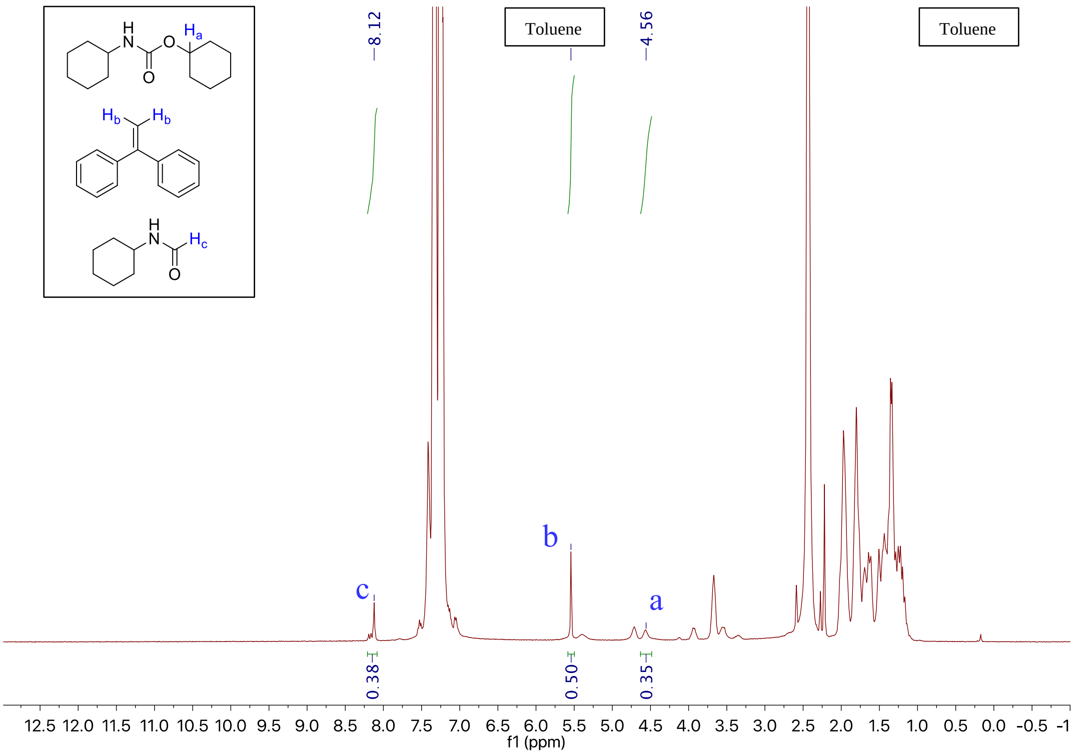

Figure S3: ^{1}H NMR spectrum ($CDCl_3$, 400 MHz, 298 K) Table 1, entry 3.

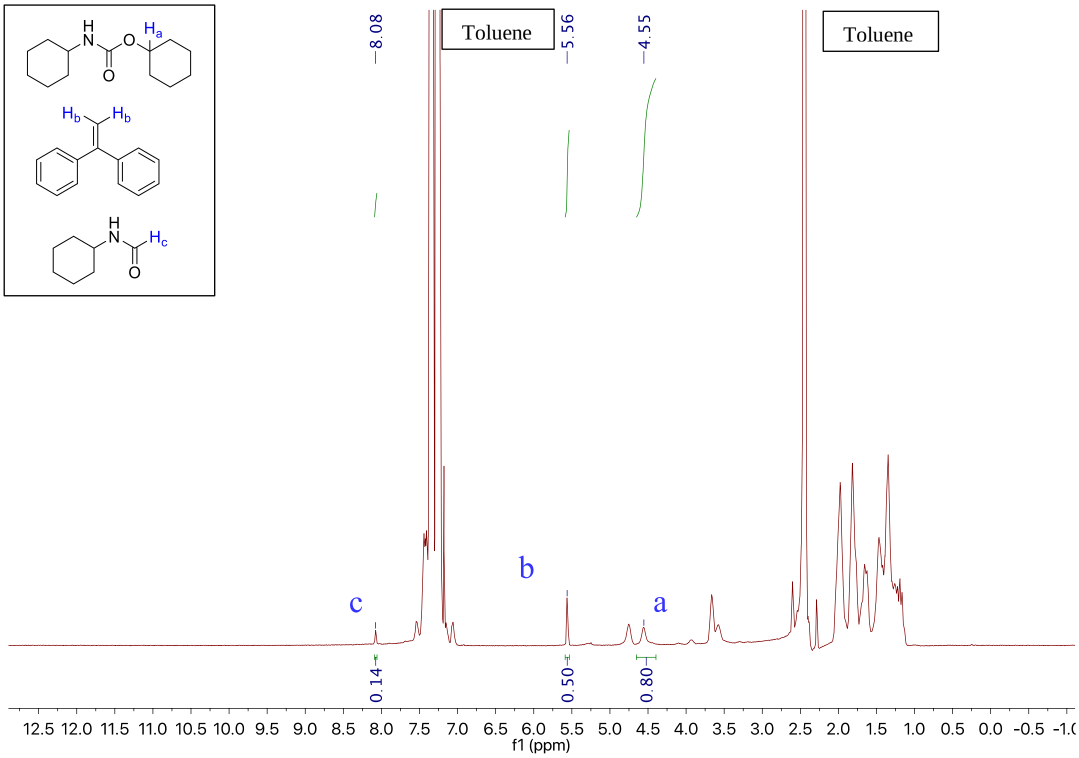

Figure S4: ^{1}H NMR spectrum ($CDCl_3$, 400 MHz, 298 K) of Table 1, entry 4.

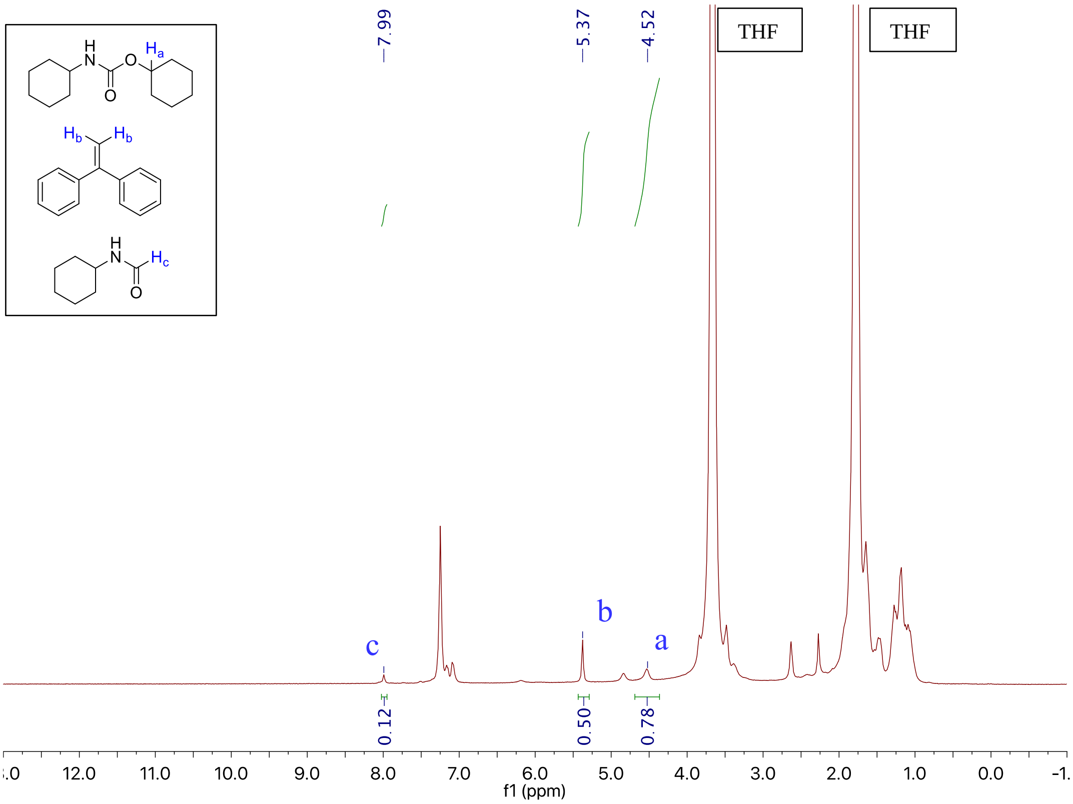

Figure S5: ¹H NMR spectrum ($CDCl_3$, 400 MHz, 298 K) of Table 1, entry 5.

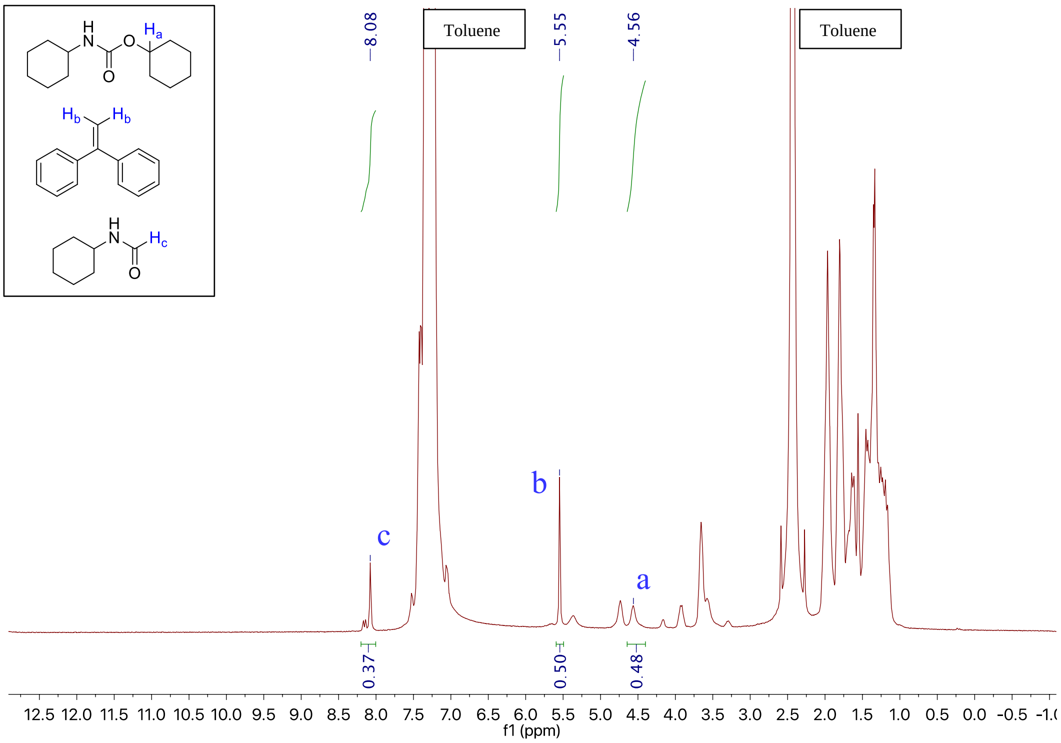

Figure S6: ¹H NMR spectrum ($CDCl_3$, 400 MHz, 298 K) of Table 1, entry 6.

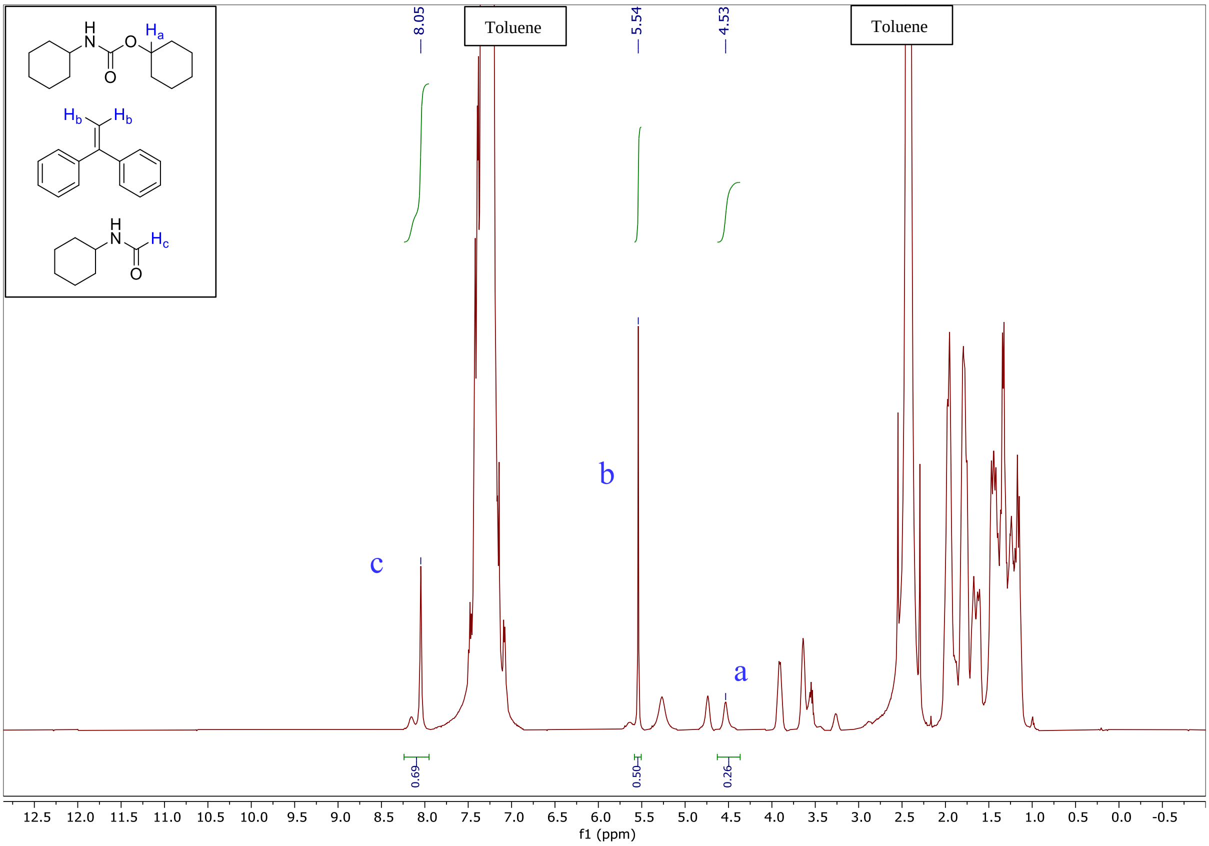

Figure S7: ^{1}H NMR spectrum ($CDCl_3$, 400 MHz, 298 K) of Table 1, entry 7.

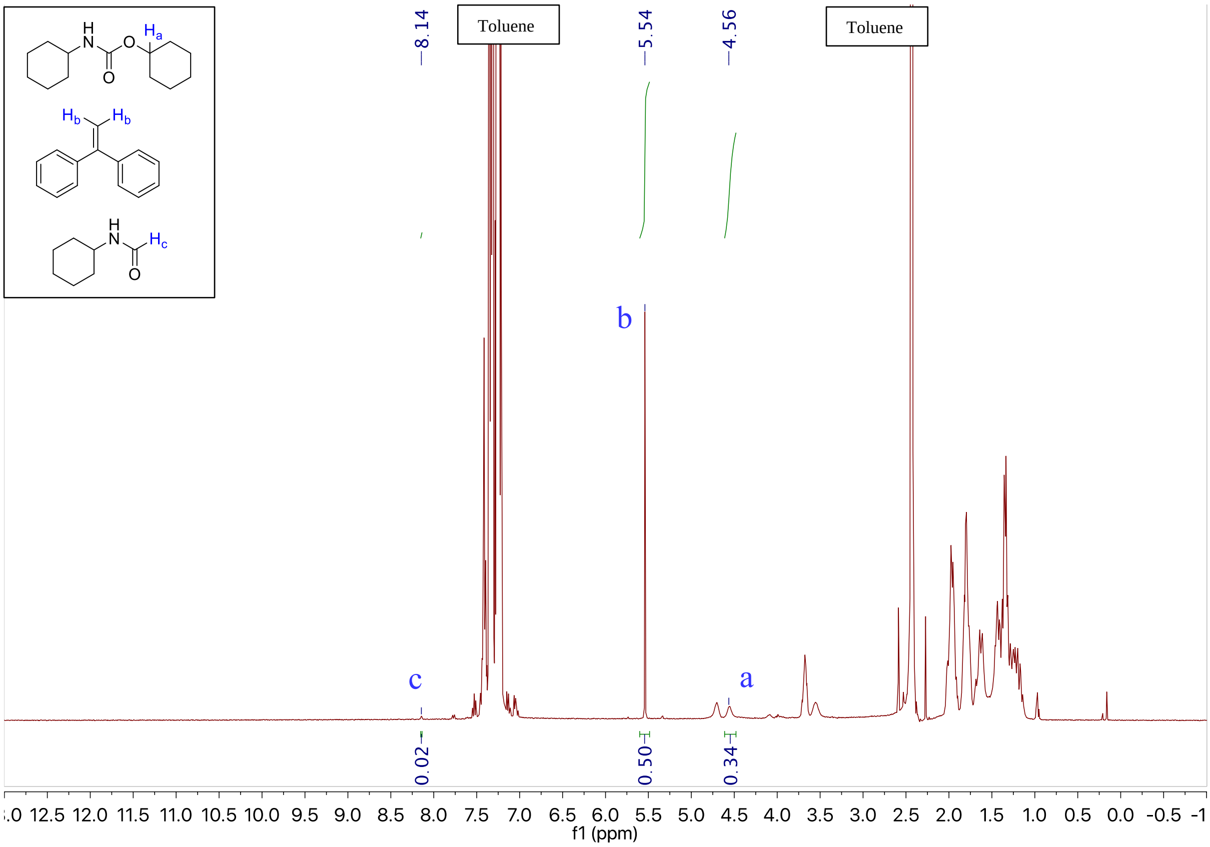

Figure S8: ^{1}H NMR spectrum ($CDCl_3$, 400 MHz, 298 K) of Table 1, entry 8.

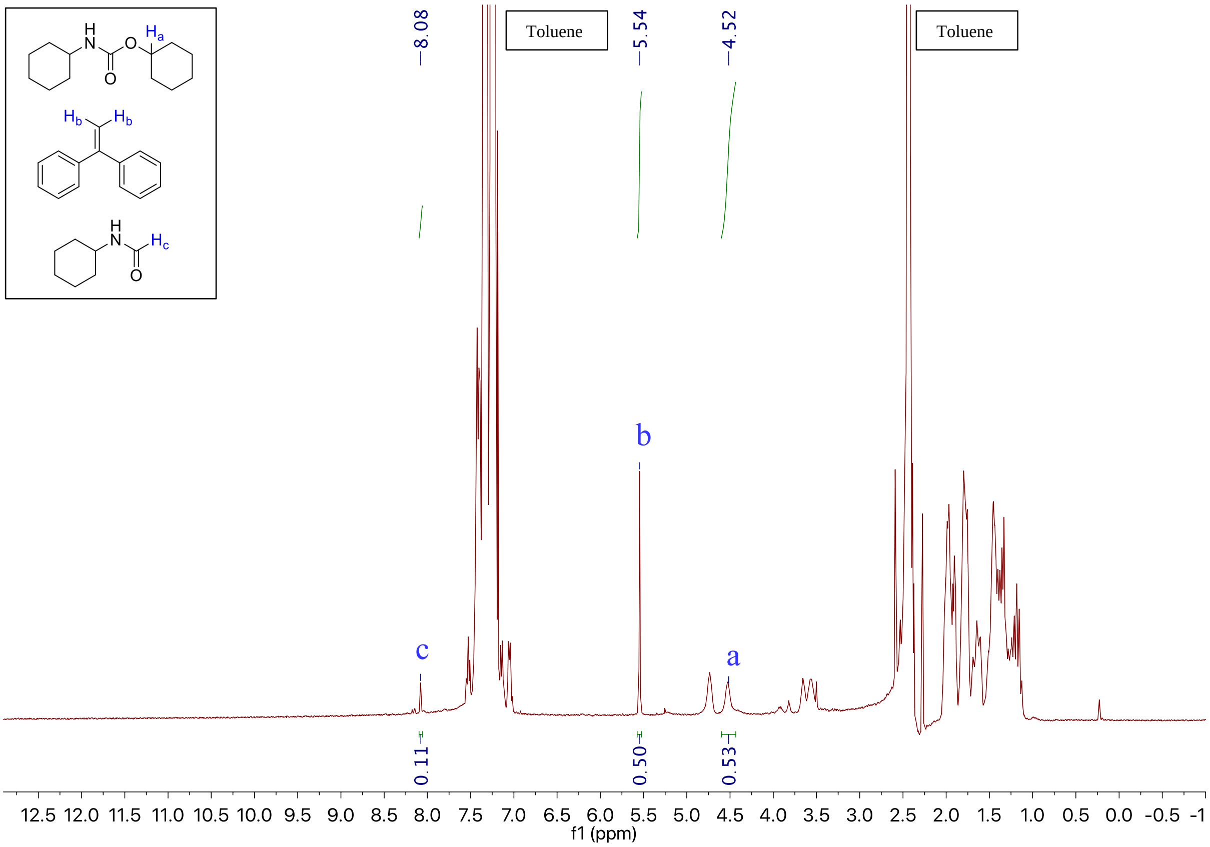

Figure S9: ^{1}H NMR spectrum ($CDCl_3$, 400 MHz, 298 K) Table 1, entry 9.

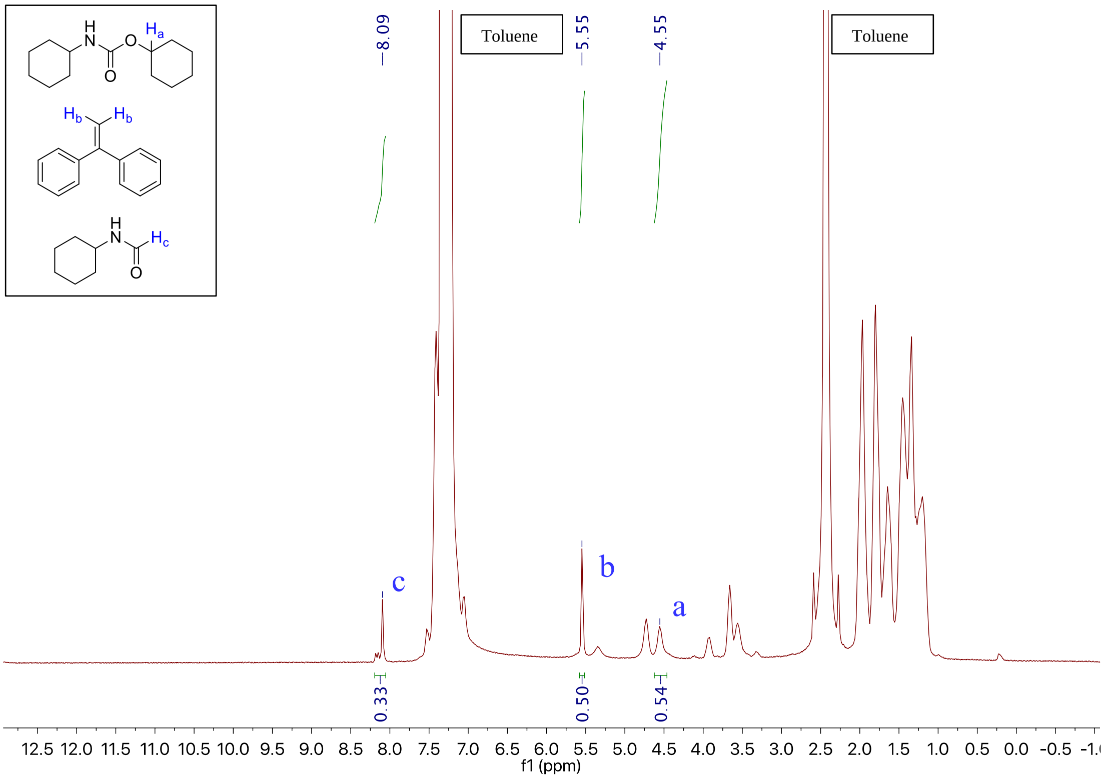

Figure S10: ^{1}H NMR spectrum ($CDCl_3$, 400 MHz, 298 K) of Table 1, entry 10.

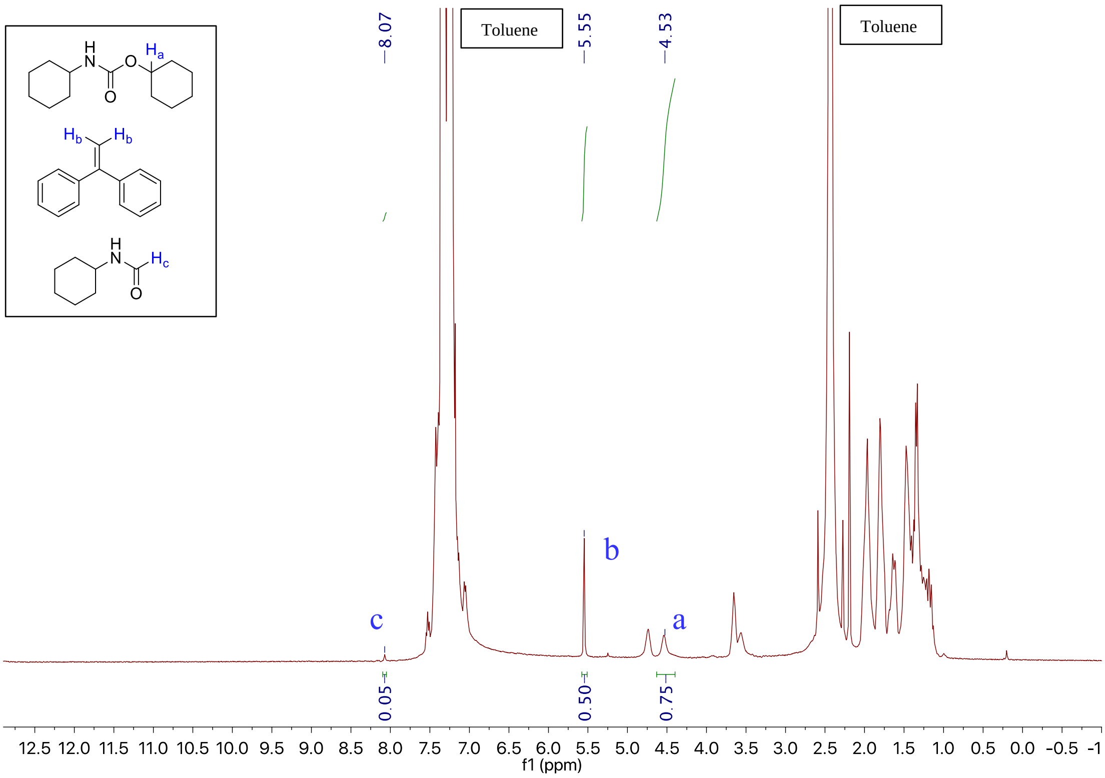

Figure S11: ^{1}H NMR spectrum ($CDCl_3$, 400 MHz, 298 K) of Table 1, entry 11.

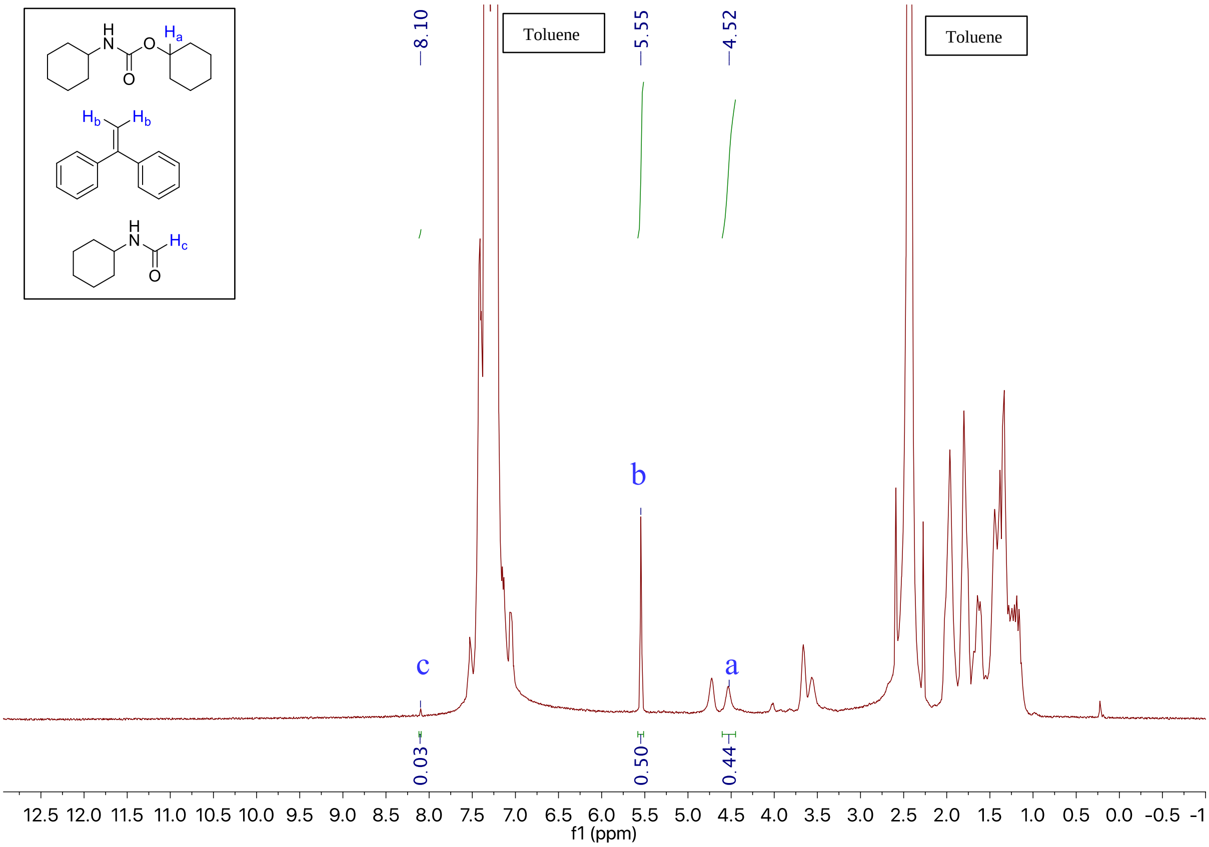

Figure S12: ^{1}H NMR spectrum ($CDCl_3$, 400 MHz, 298 K) of Table 1, entry 12.

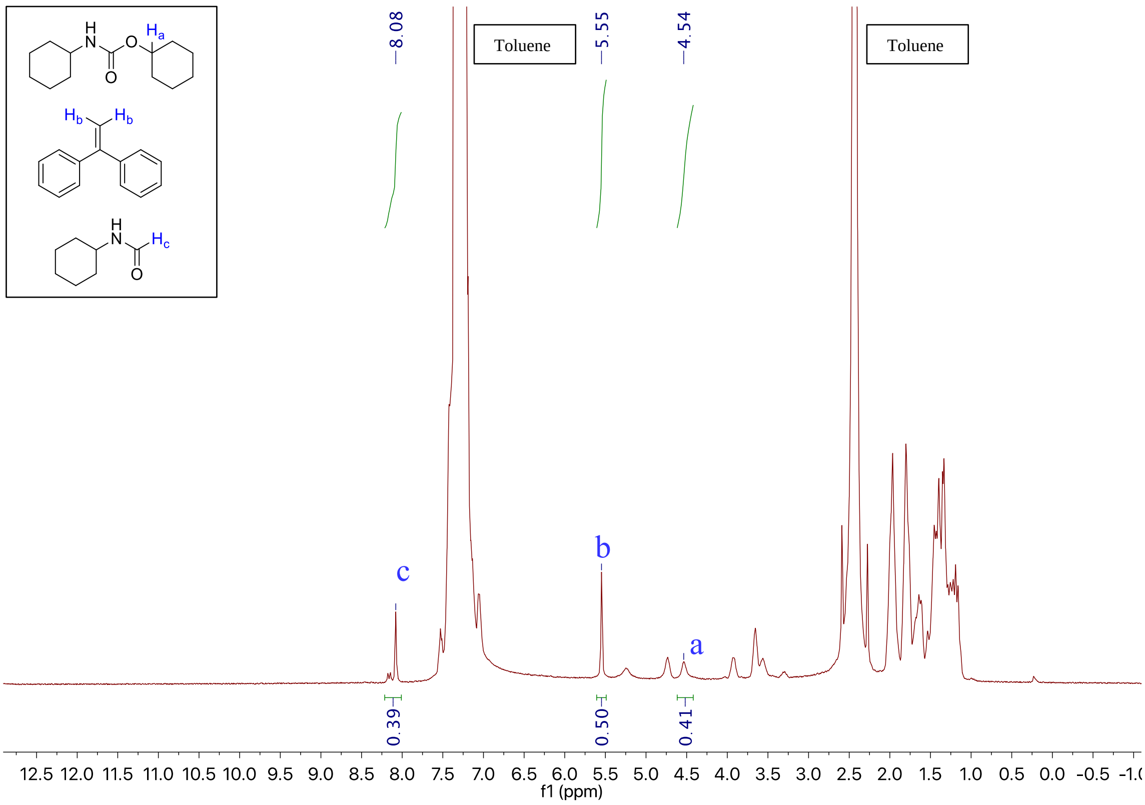

Figure S13: ^{1}H NMR spectrum ($CDCl_3$, 400 MHz, 298 K) Table 1, entry 13.

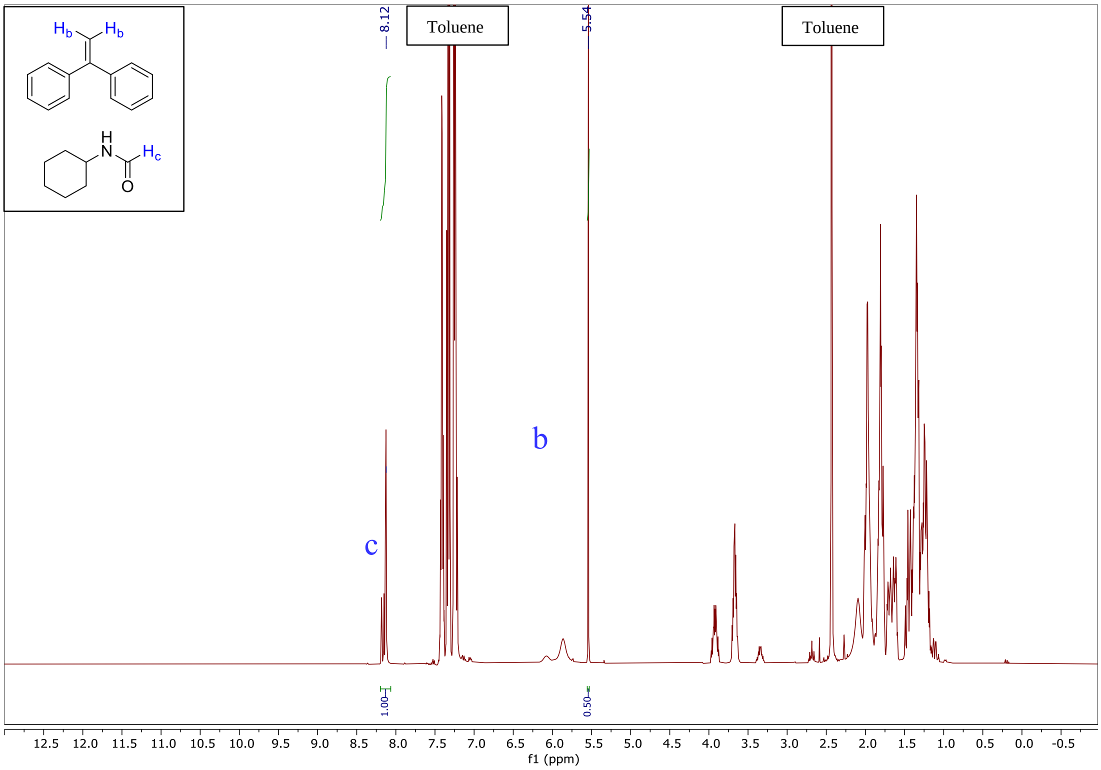

Figure S14: ^{1}H NMR spectrum ($CDCl_3$, 400 MHz, 298 K) of Table 1, entry 14.

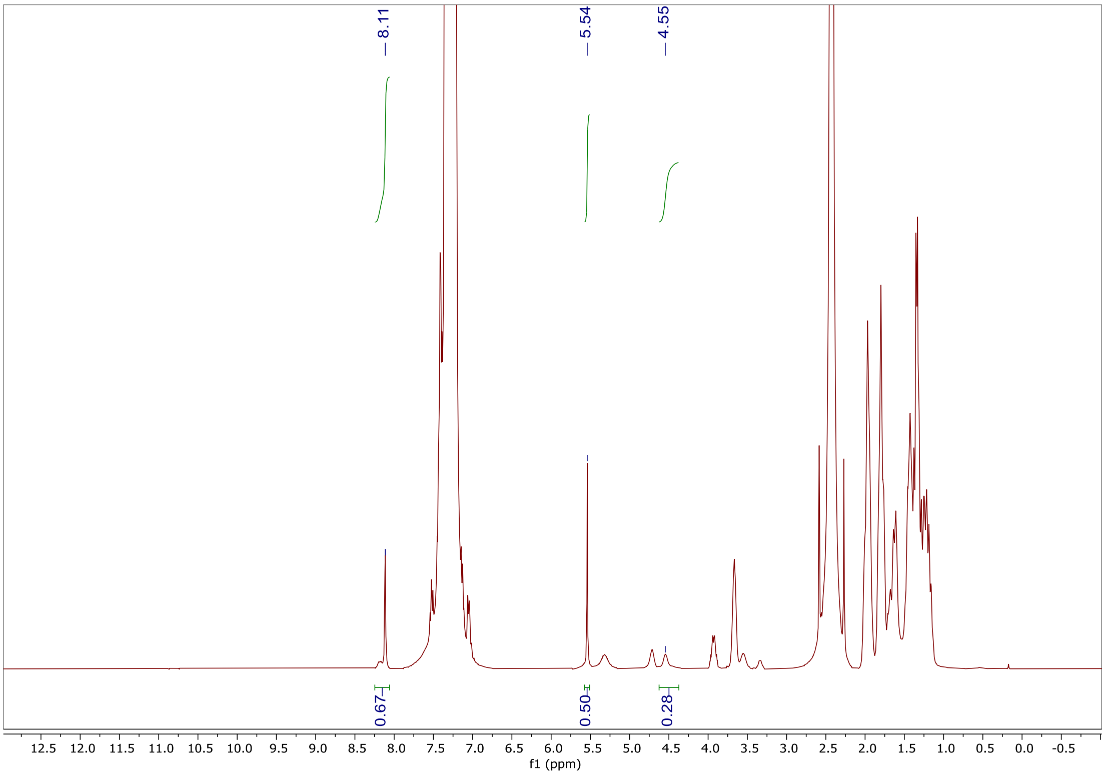

Figure S15: ^{1}H NMR spectrum ($CDCl_3$, 400 MHz, 298 K) of Table S1, entry 15.

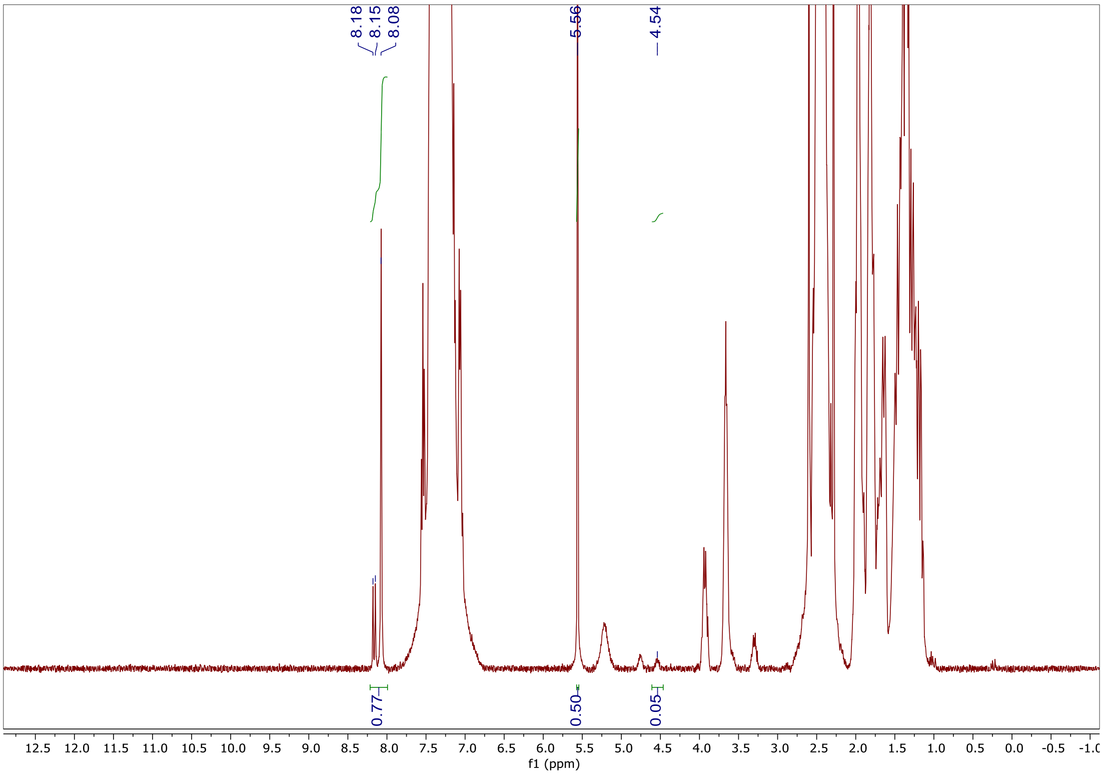

Figure S16: ^{1}H NMR spectrum ($CDCl_3$, 400 MHz, 298 K) of Table S1, entry 16.

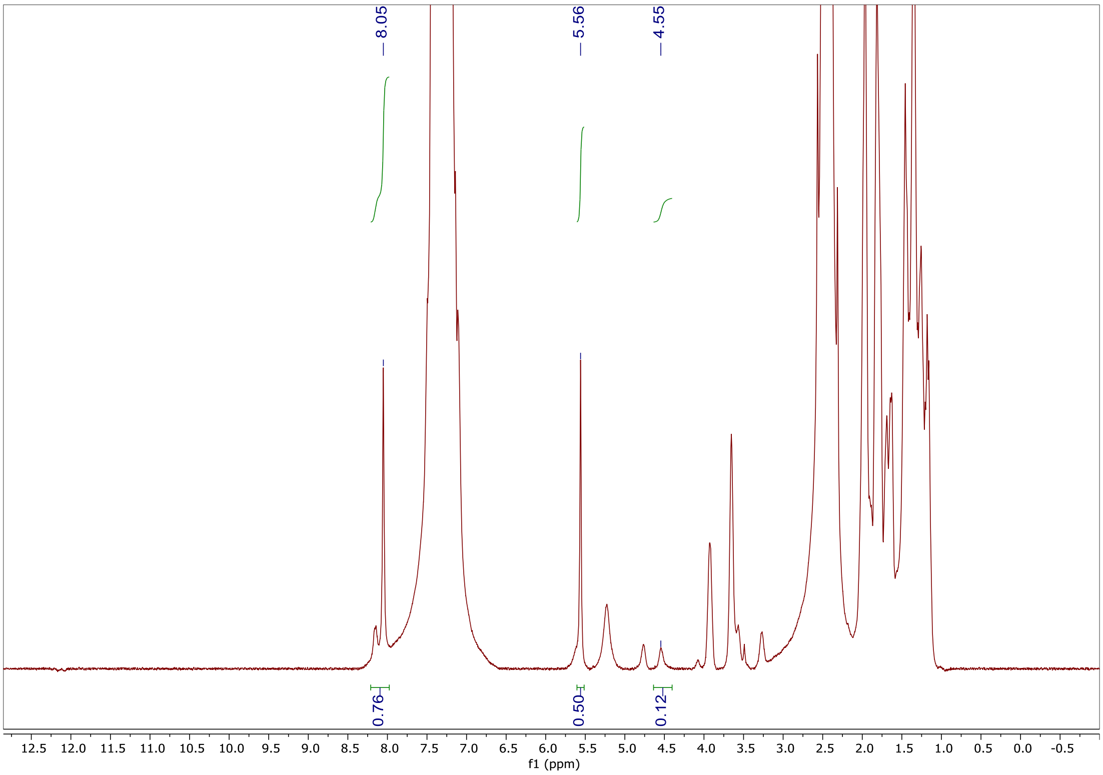

Figure S17: ^{1}H NMR spectrum ($CDCl_3$, 400 MHz, 298 K) of Table S1, entry 17.

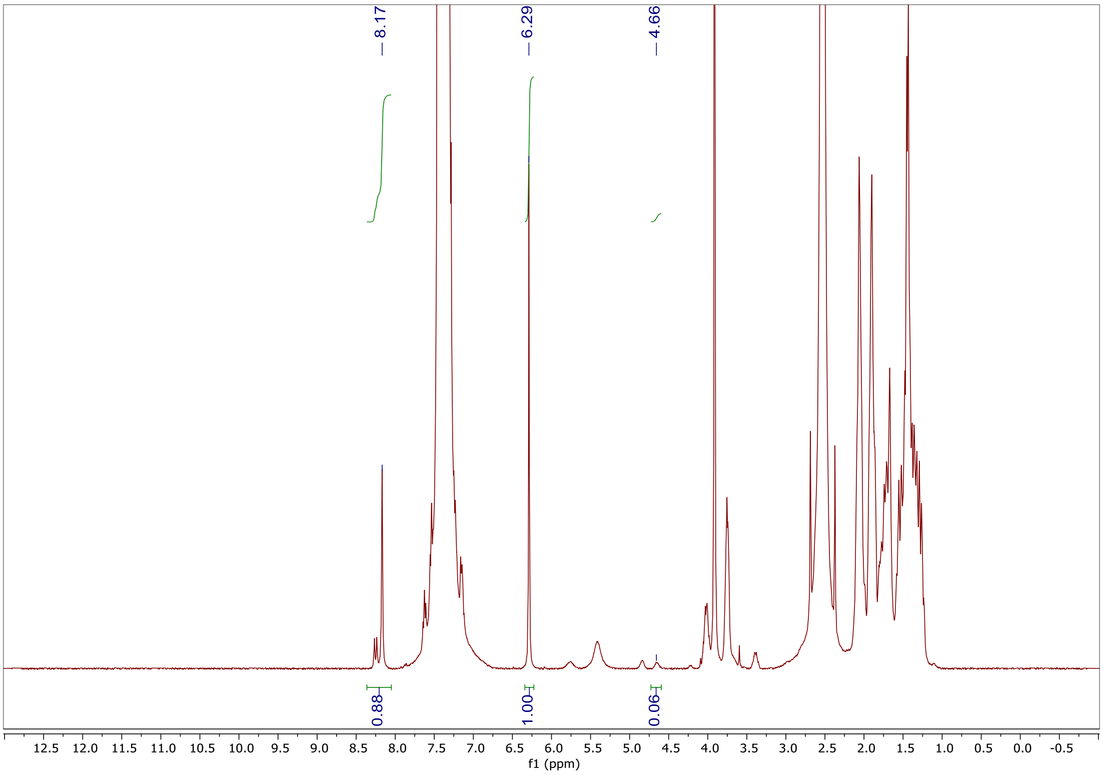

Figure S18: ^{1}H NMR spectrum ($CDCl_3$, 400 MHz, 298 K) of Table S1, entry 18.

3.1.2. GC-MS data for carbamate synthesis

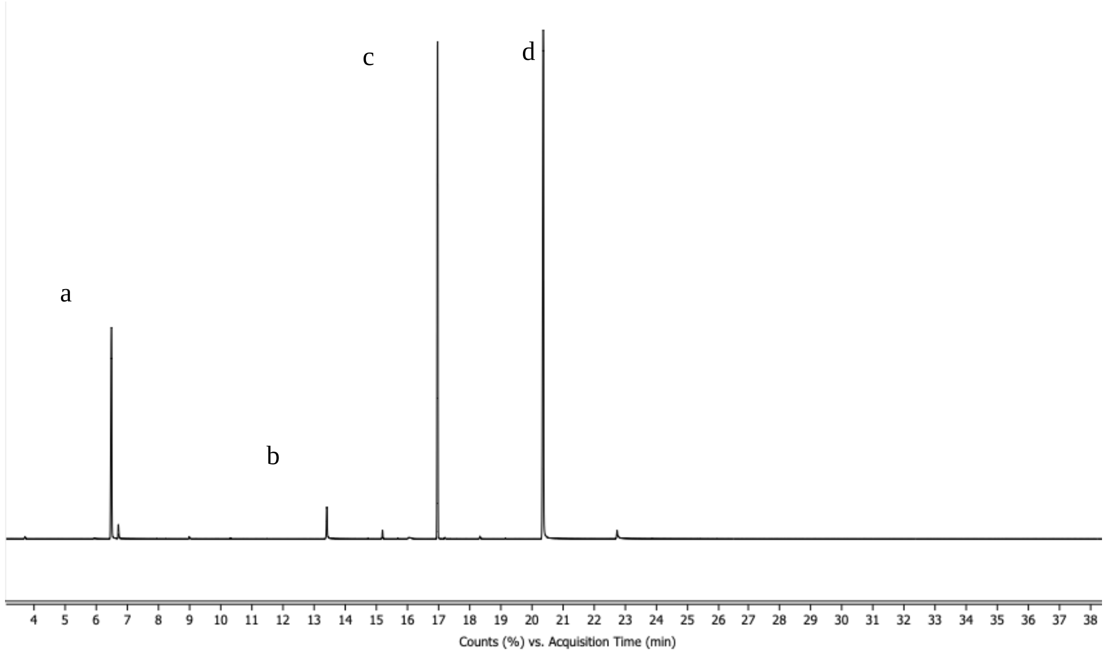

Figure S19: Representative gas chromatograph of the reaction mixture from the dehydrogenative coupling of N-cyclohexylformamide and cyclohexanol (Table 1, entry 4).

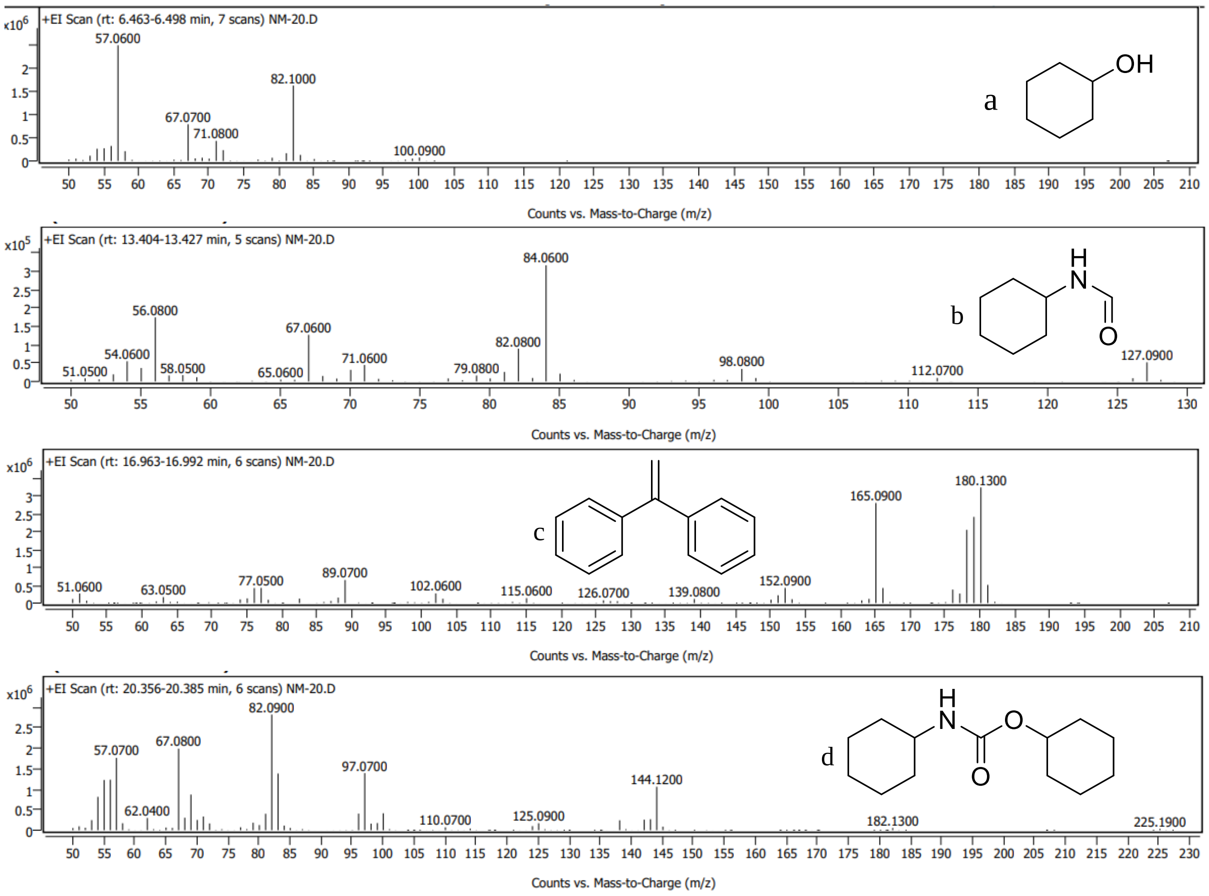

Figure S20: Mass spectra corresponding to gas chromatograph of the reaction mixture from the dehydrogenative coupling of N-cyclohexylformamide and cyclohexanol (Figure S15).

3.2. Characterisation data for urea synthesis

3.2.1. Characterisation data for isolated urea compounds

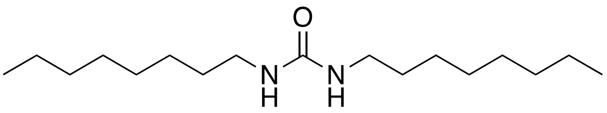

White crystals (72.3 mg, 51%).
^{1}H NMR (500 MHz, CD_3OD): δ_H 3.09 (4H, t, J = 7.0 Hz), 1.46 (4H, m), 1.32 (20H, m), 0.89 (6H, m).
^{13}C{^{1}H} NMR (126 MHz, CD_3OD): δ_C 161.4, 41.0, 33.0, 31.4, 30.5, 30.4, 28.0, 23.7, 14.4.
IR (ATR-FTIR, cm^{-1}): ν 3331m (NH), 2955m, 2924s and 2849m (CH), 1611s (C=O), 1568s (NH).
GC-MS (*m/z*): 284.26

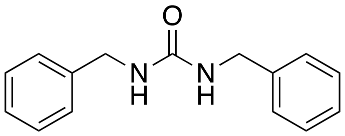

White crystals (81.2 mg, 68%).
^{1}H NMR (500 MHz, CD_3OD): δ_H 7.32-7.27 (8H, m), 7.24-7.21 (2H, m), 4.34 (4H, s).
^{13}C{^{1}H} NMR (126 MHz, CD_3OD): δ_C 161.1, 141.3, 129.5, 128.2, 128.0, 44.8.
IR (ATR-FTIR, cm^{-1}): ν 3319m (NH), 3028w and 2872w (CH), 1611s (C=O), 1568s (NH).
GC-MS (*m/z*): 240.10

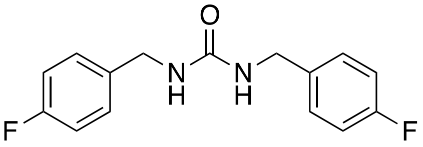

White crystals (114.3 mg, 83%).
^{1}H NMR (500 MHz, CD_3OD): δ_H 7.29 (4H, dd, J = 8.59, 5.46 Hz), 7.02 (4H, t, J = 8.79 Hz), 4.30 (4H, s).
^{13}C{^{1}H} NMR (126 MHz, CD_3OD): δ_C 163.4 (d, J = 243.3 Hz), 160.9, 137.4, 130.1, 116.0 (d, J = 21.6 Hz), 44.1.
IR (ATR-FTIR, cm^{-1}): ν 3317m (NH), 3041w and 2877w (CH), 1608m (C=O), 1558s (NH).
GC-MS (*m/z*): 276.08

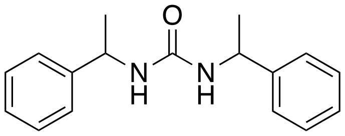

White crystals (84.5 mg, 63%).
^{1}H NMR (500 MHz, CD_3OD): δ_H 7.31 – 7.26 (8H, m), 7.22 – 7.19 (2H, m), 4.78 (2H, q, J = 6.94 Hz), 1.38 (6H, d, J = 6.97 Hz).
^{13}C{^{1}H} NMR (126 MHz, CD_3OD): δ_C 159.5, 146.4, 129.5, 127.9, 126.8, 50.7, 23.6.
IR (ATR-FTIR, cm^{-1}): ν 3319m (NH), 2978w and 2866w (CH), 1624s (C=O), 1568 (NH).
GC-MS (*m/z*): 268.12

3.2.2. Crude ^{1}H NMR spectra for the synthesis of urea derivatives

Table S2: Catalyst optimization for urea synthesis.[a]

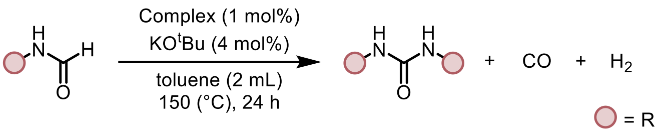

Entry	Complex	R	Conversion (%)	Yield (%)
1	**1**	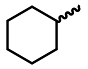	39	37
2	**2**	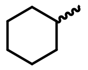	25	12
3	**3**	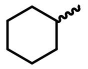	15	8
4	**4**	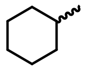	5	3

[a]Catalytic conditions: Formamide (1 mmol), complex 1 (0.01 mmol), KO^{t}Bu (0.04 mmol), toluene (2 mL), 24 hours, 150 ℃. Conversion and yields are estimated by ^{1}H NMR spectroscopy using 1,3,5-trimethoxybenzene (0.33 mmol) as an internal standard. Numbers in parentheses are isolated yields.

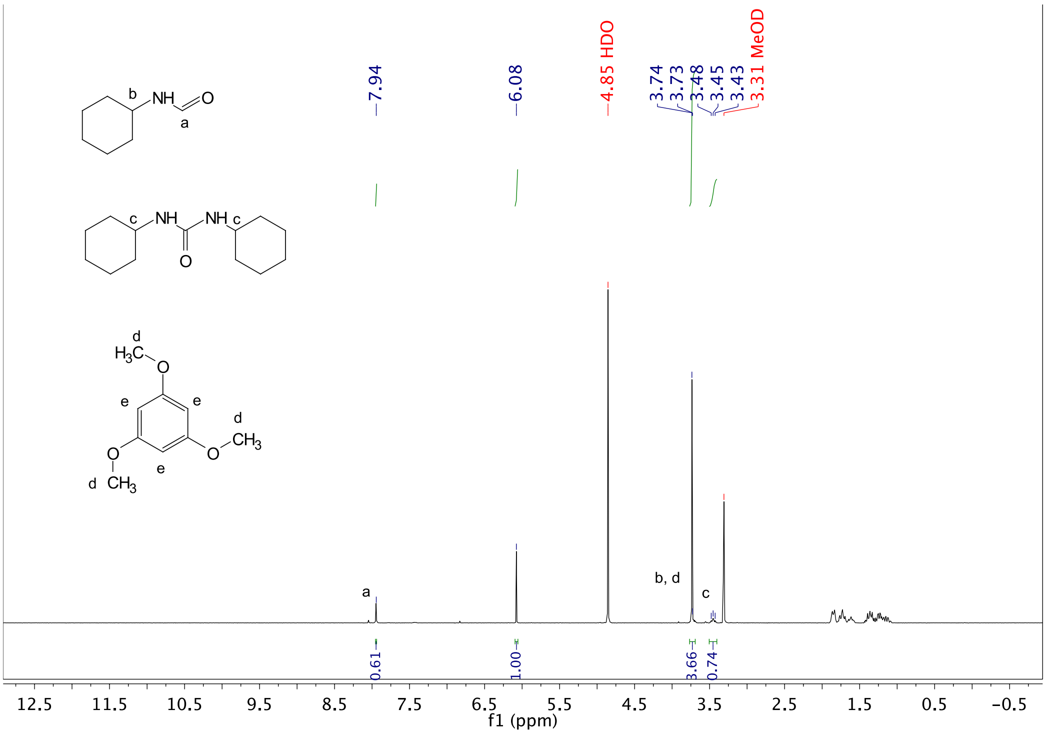

Figure S21: Crude ^{1}H NMR spectrum (CD_3OD, 400 MHz, 298K) for Table S2, entry 1 and Table 2, entry 2.

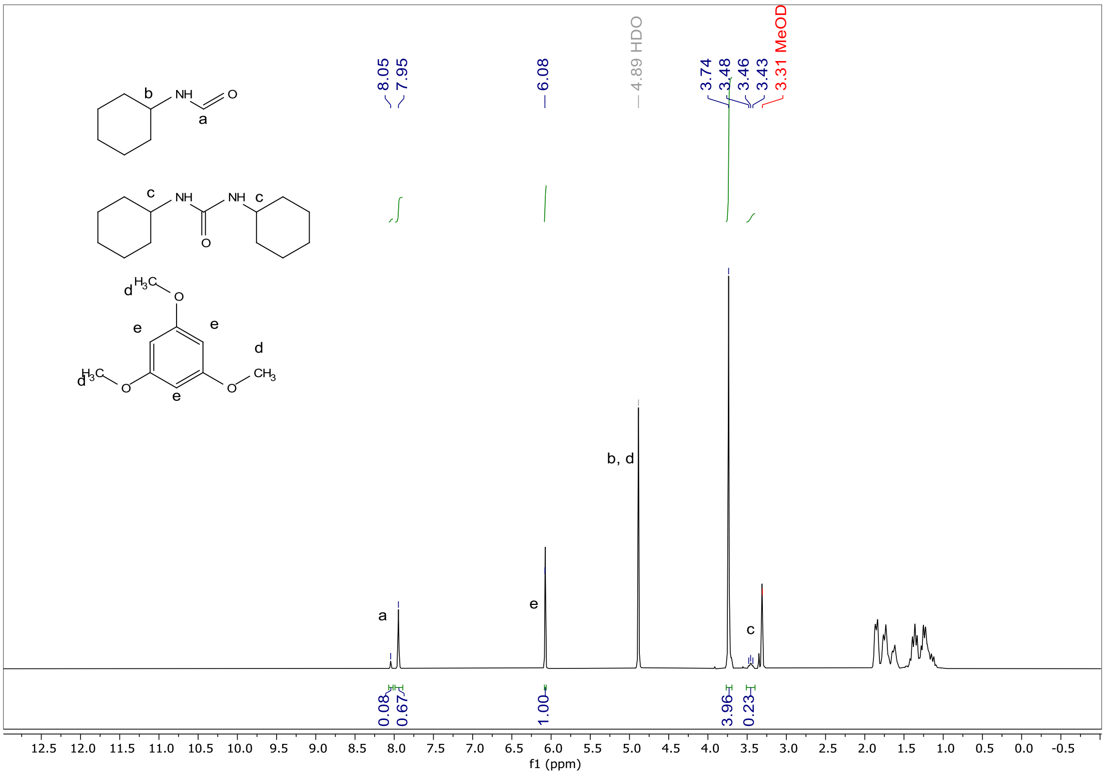

Figure S22: Crude ^{1}H NMR spectrum (CD_3OD, 400 MHz, 298K) for Table S2, entry 2.

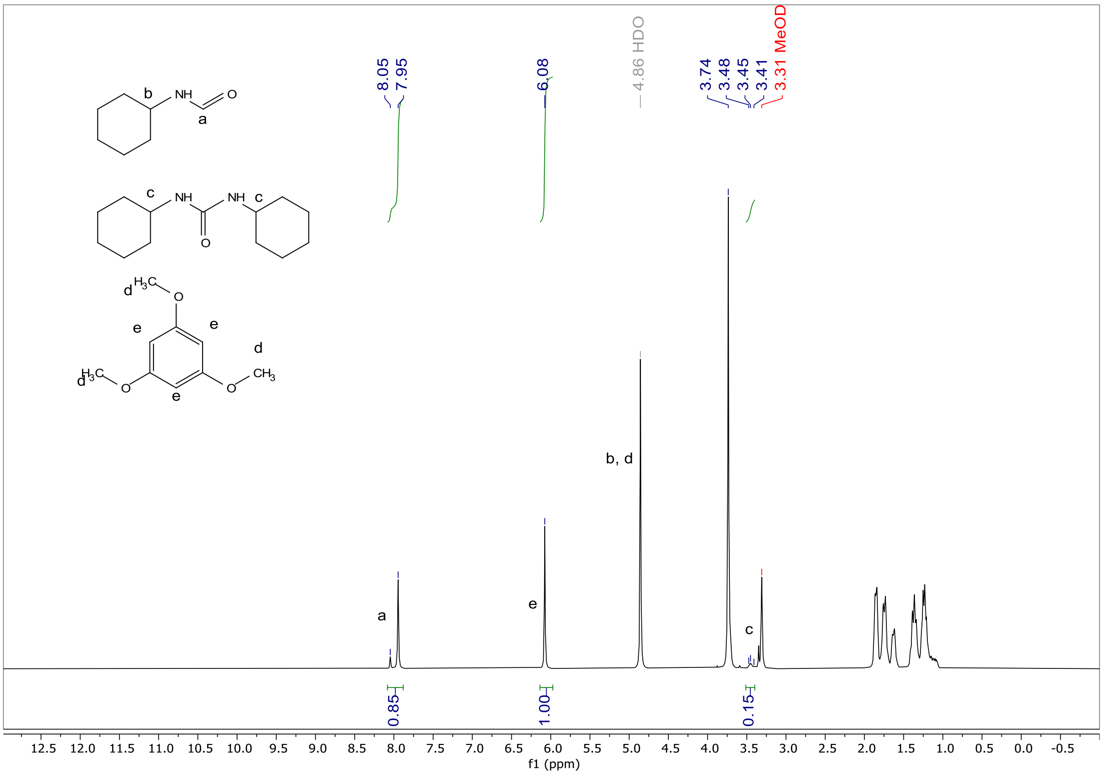

Figure S23: Crude ^{1}H NMR spectrum (CD_3OD, 400 MHz, 298K) for Table S2, entry 3.

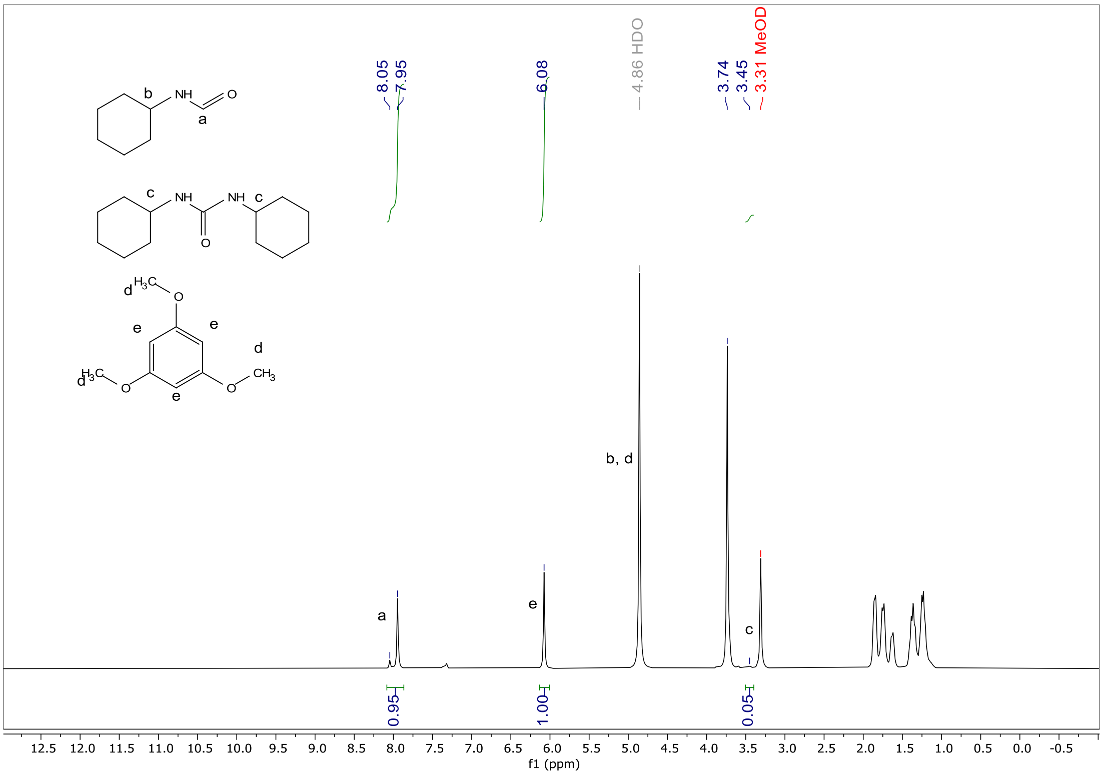

Figure S24: Crude ^{1}H NMR spectrum (CD_3OD, 400 MHz, 298K) for Table S2, entry 4.

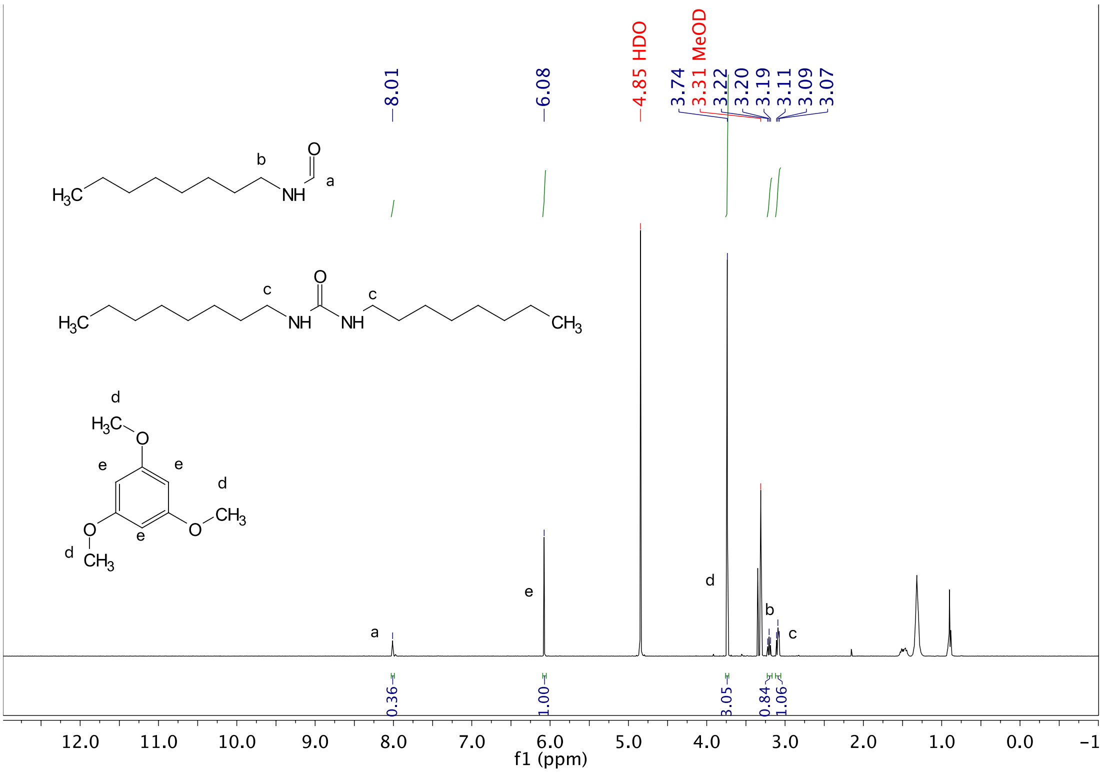

Figure S25: Crude ^{1}H NMR spectrum (CD_3OD, 400 MHz, 298K) for Table 2, entry 1.

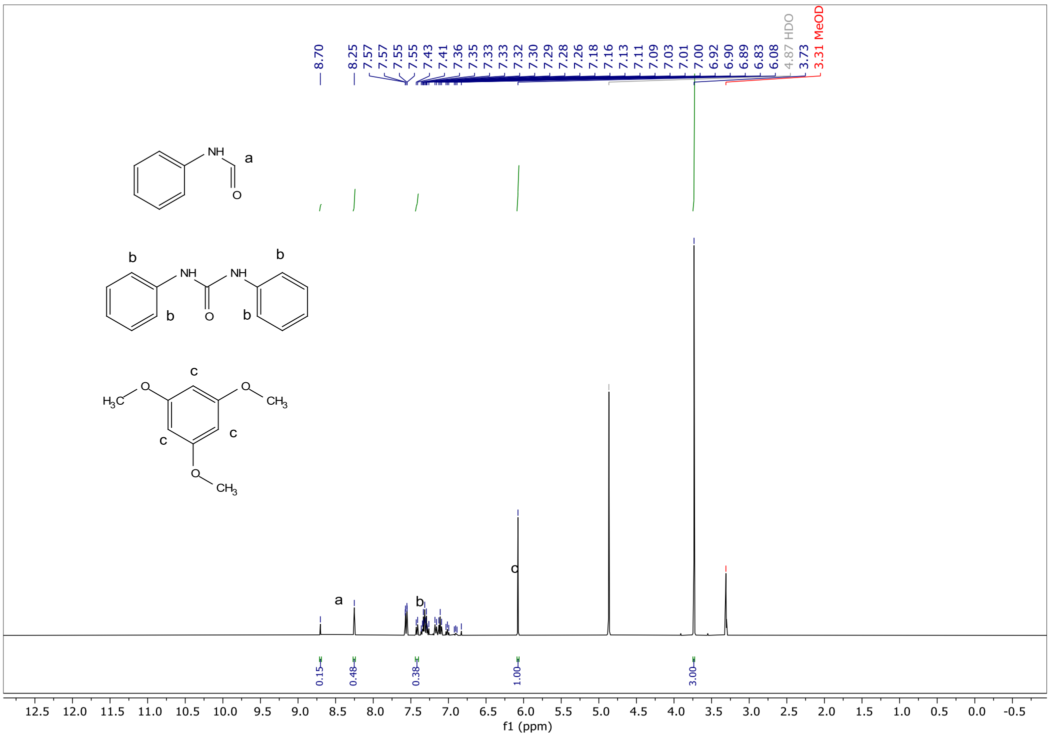

Figure S26: Crude ^{1}H NMR spectrum (CD_3OD, 400 MHz, 298K) for Table 2, entry 3.

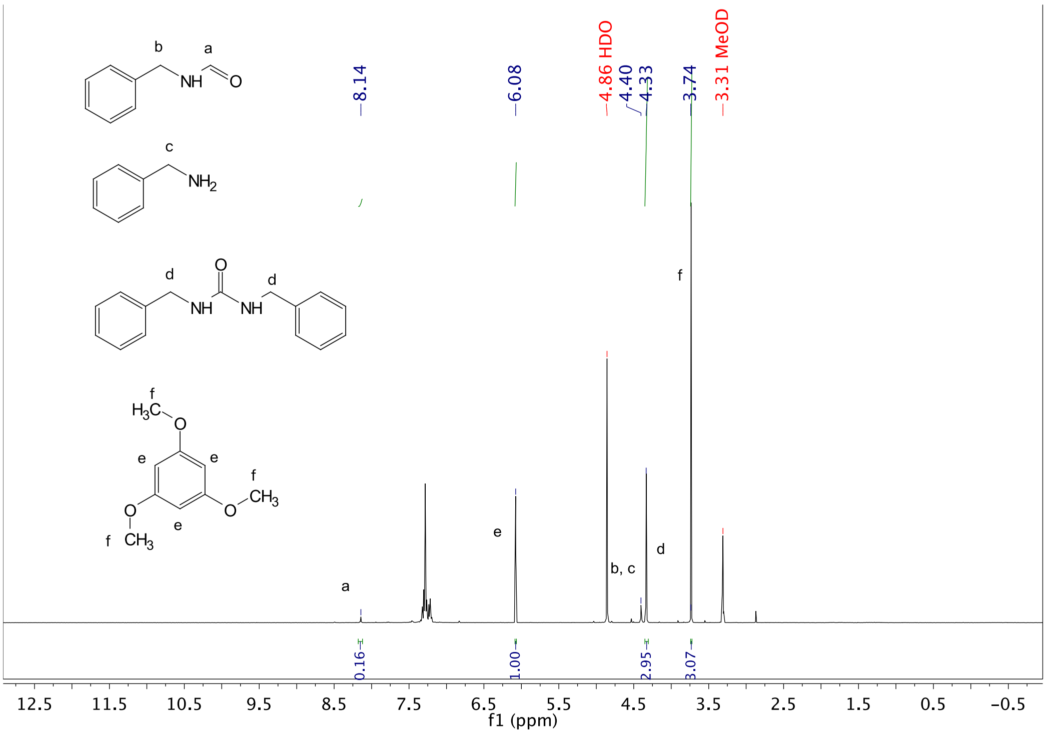

Figure S27: Crude ^{1}H NMR spectrum (CD_3OD, 400 MHz, 298K) Table 2, entry 4.

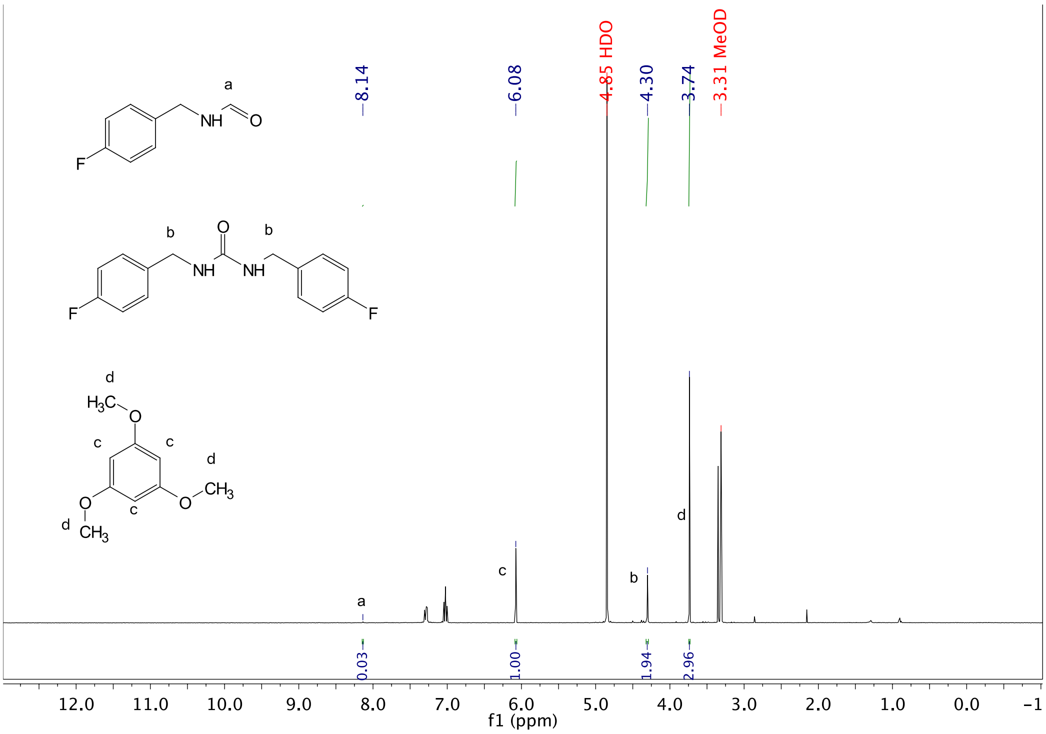

Figure S28: Crude ^{1}H NMR spectrum (CD_3OD, 400 MHz, 298K) Table 2, entry 5.

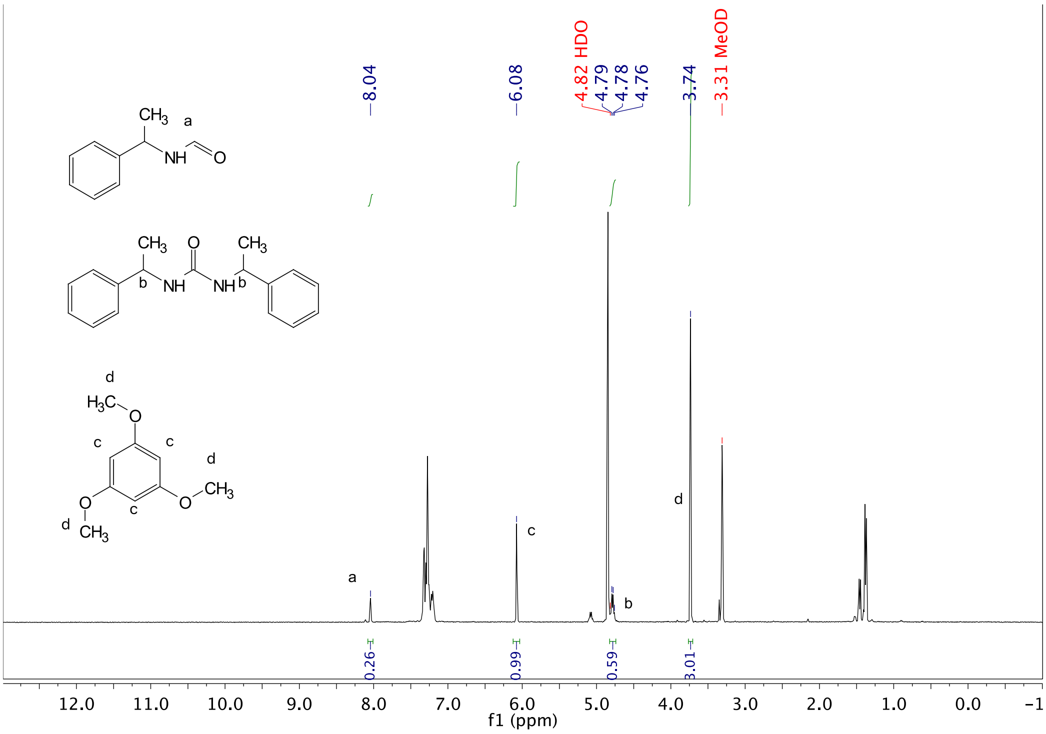

Figure S29: Crude ^{1}H NMR spectrum (CD_3OD, 400 MHz, 298K) for Table 2, entry 6.

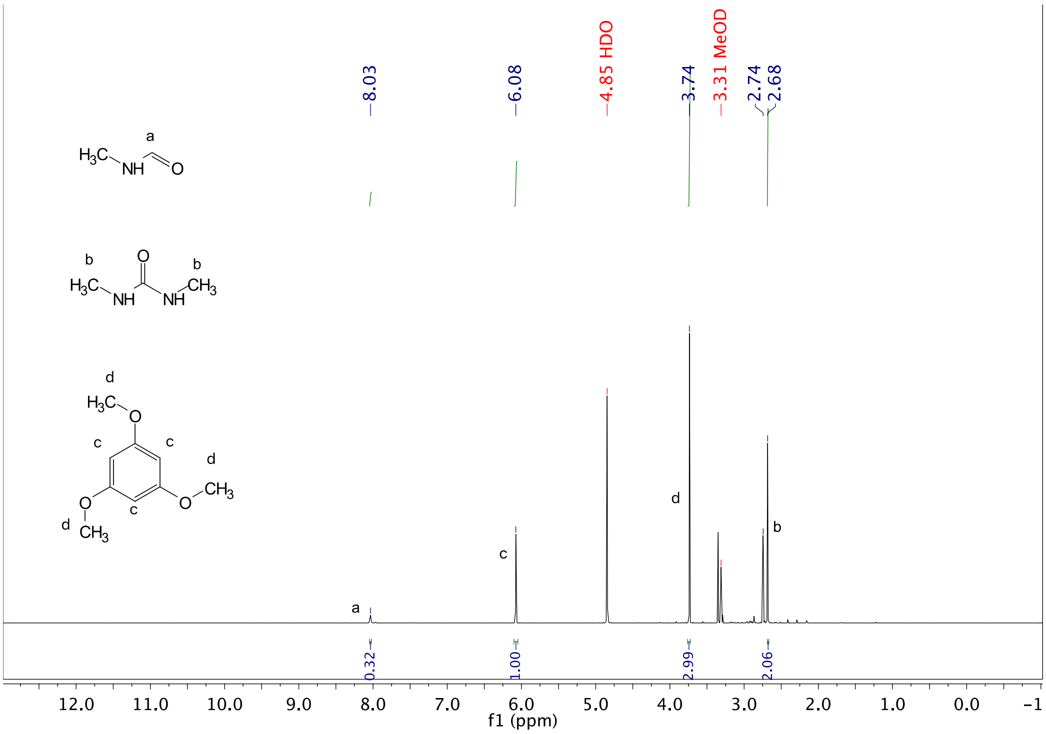

Figure S30: Crude ^{1}H NMR spectrum (CD_3OD, 400 MHz, 298K) for Table 2, entry 7.

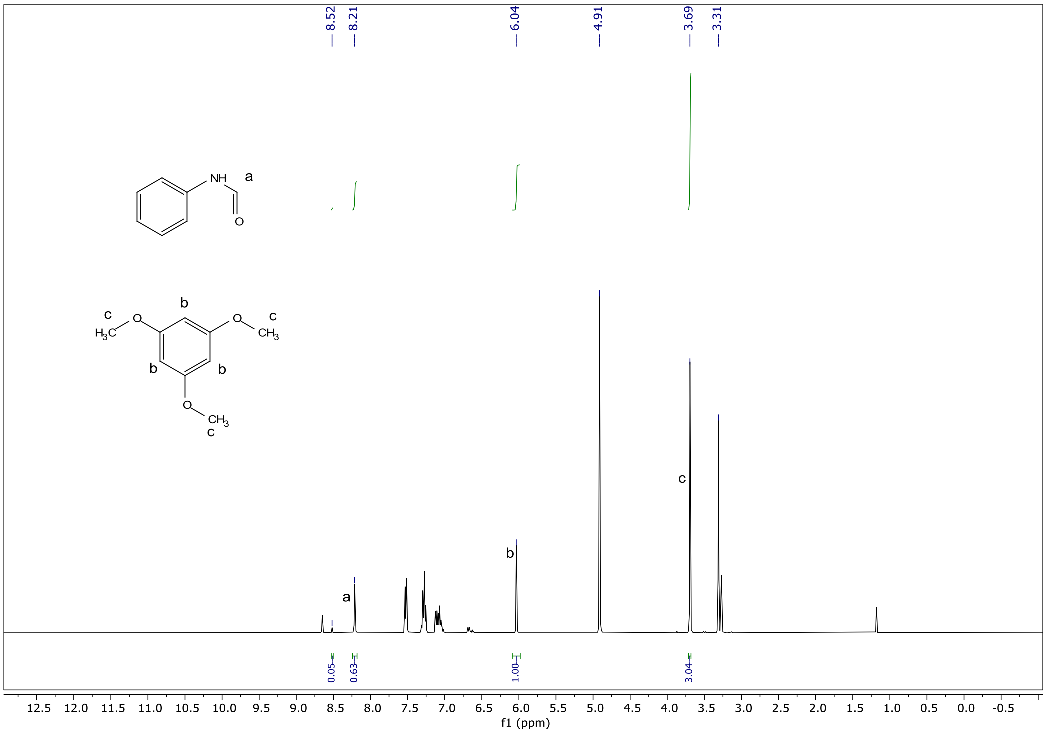

Figure S31: Crude ^{1}H NMR spectrum (CD_3OD, 400 MHz, 298K) for Table 2, entry 8.

3.2.3. Crude GC-MS data for urea synthesis

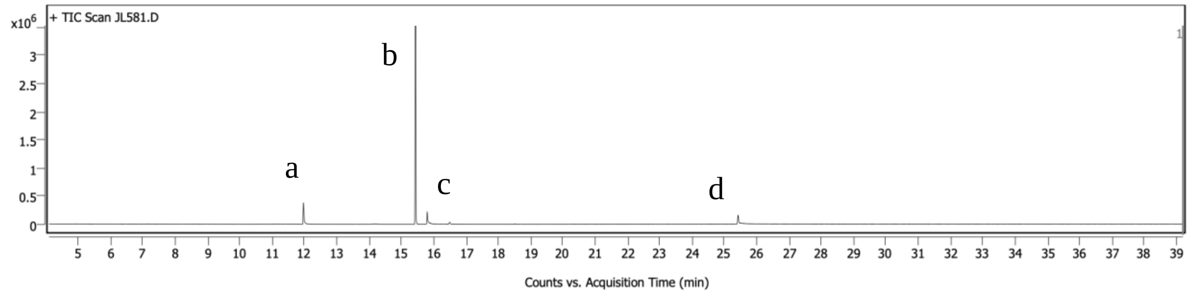

Figure S32: Gas chromatogram of crude reaction mixture from Table 2, entry 1.

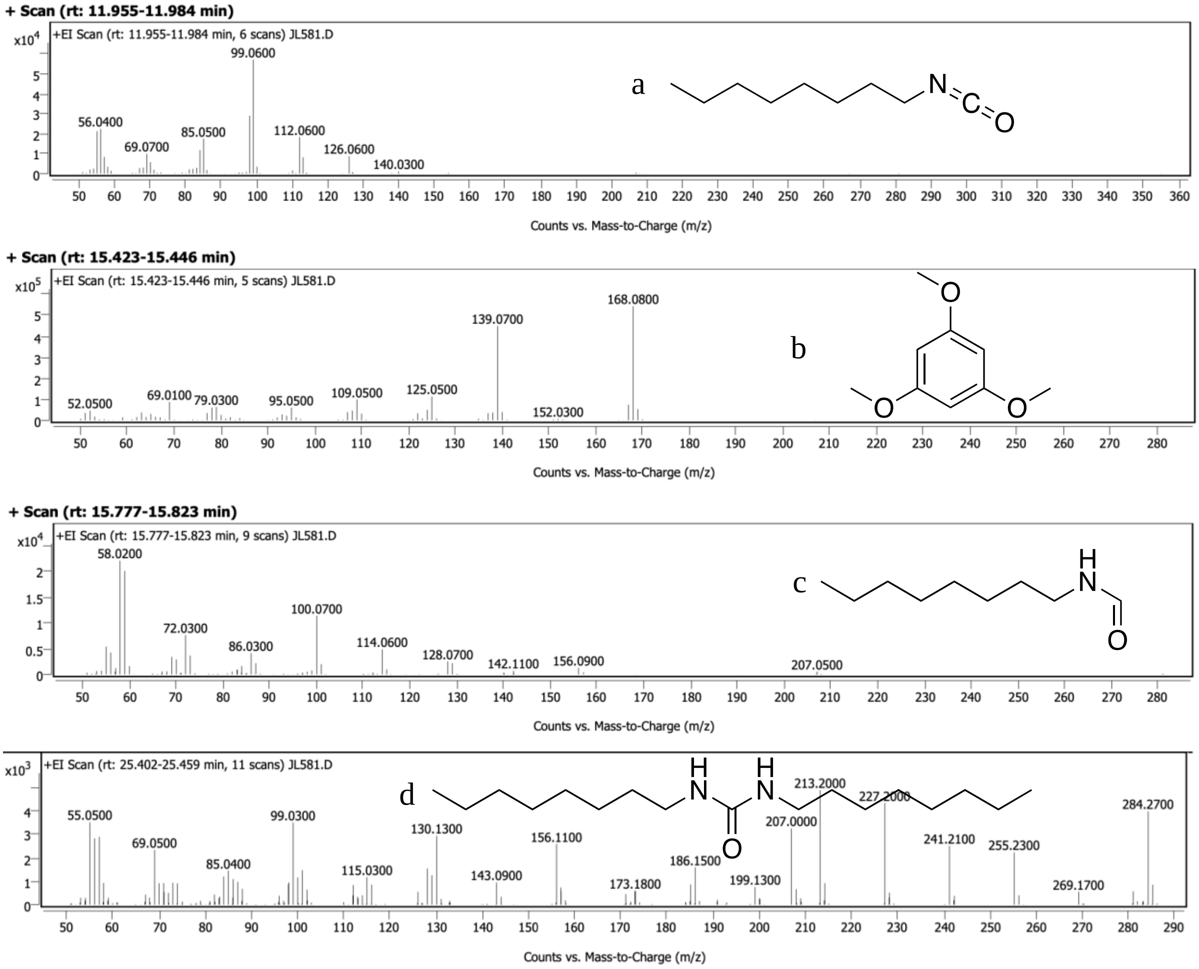

Figure S33: Mass spectra corresponding to gas chromatogram of crude reaction mixture from Table 2, entry 1 (Figure S25).

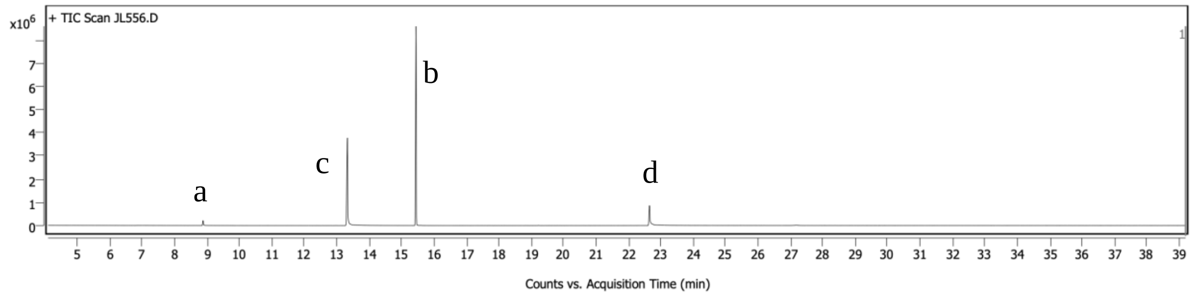

Figure S34: Gas chromatogram of crude reaction mixture from Table 2, entry 2.

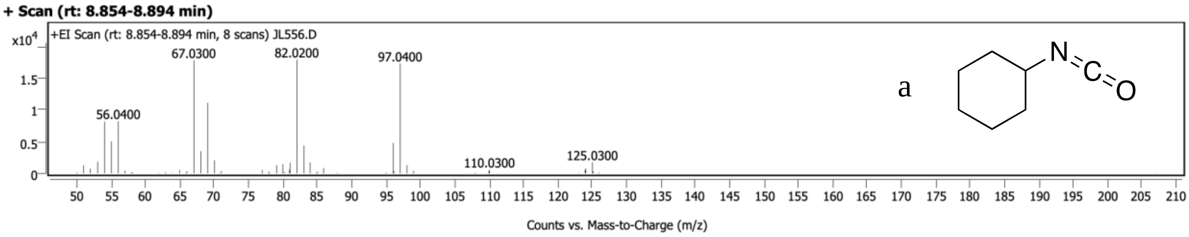

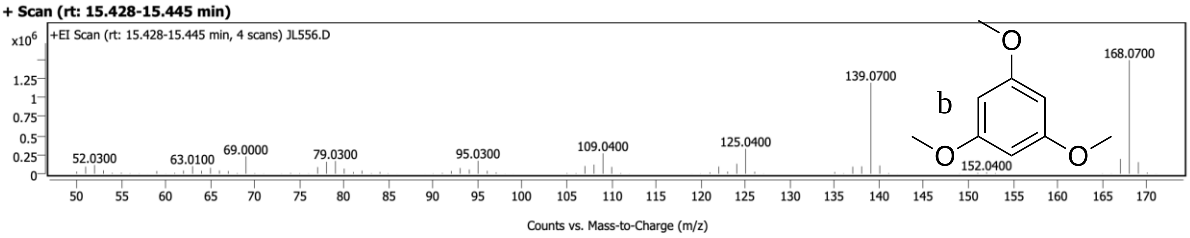

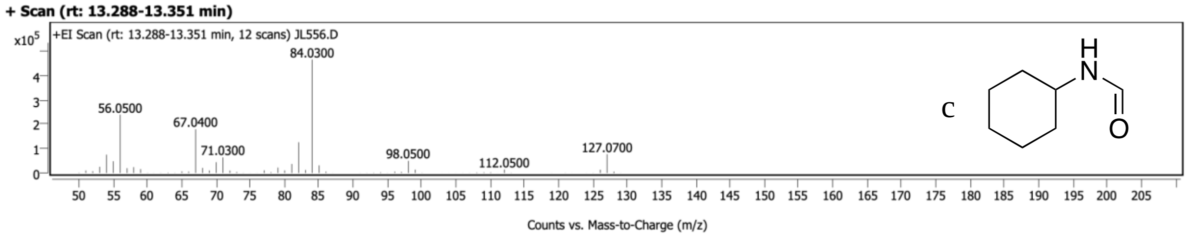

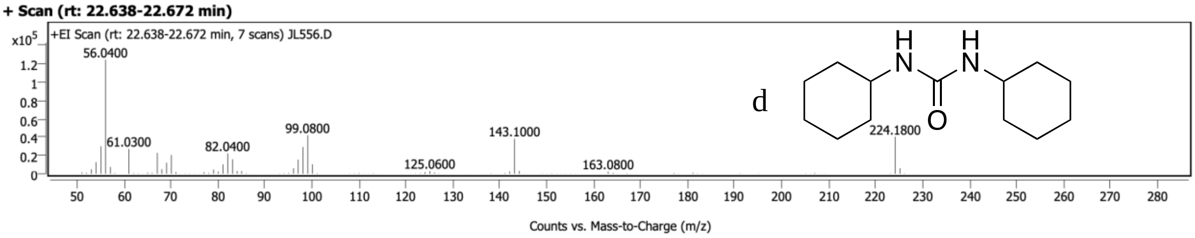

Figure S35: Mass spectra corresponding to gas chromatogram of crude reaction mixture from Table 2, entry 2 (Figure S27).

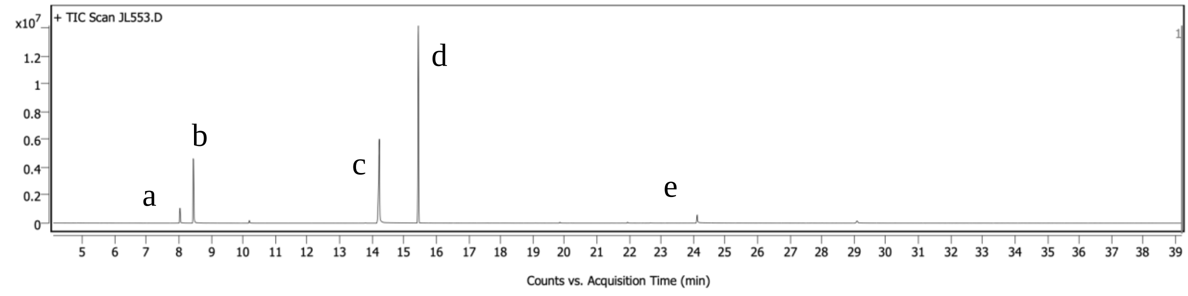

Figure S36: Gas chromatogram of crude reaction mixture from Table 2, entry 3.

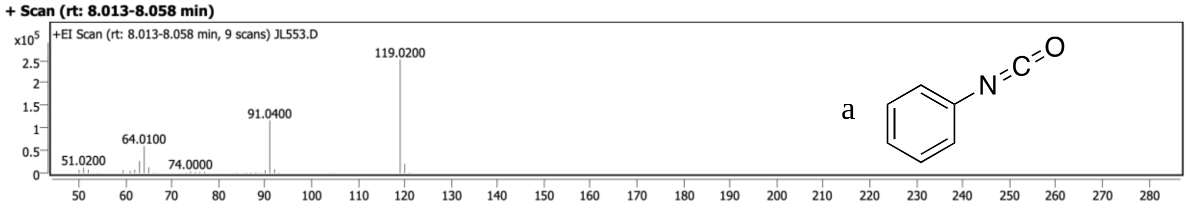

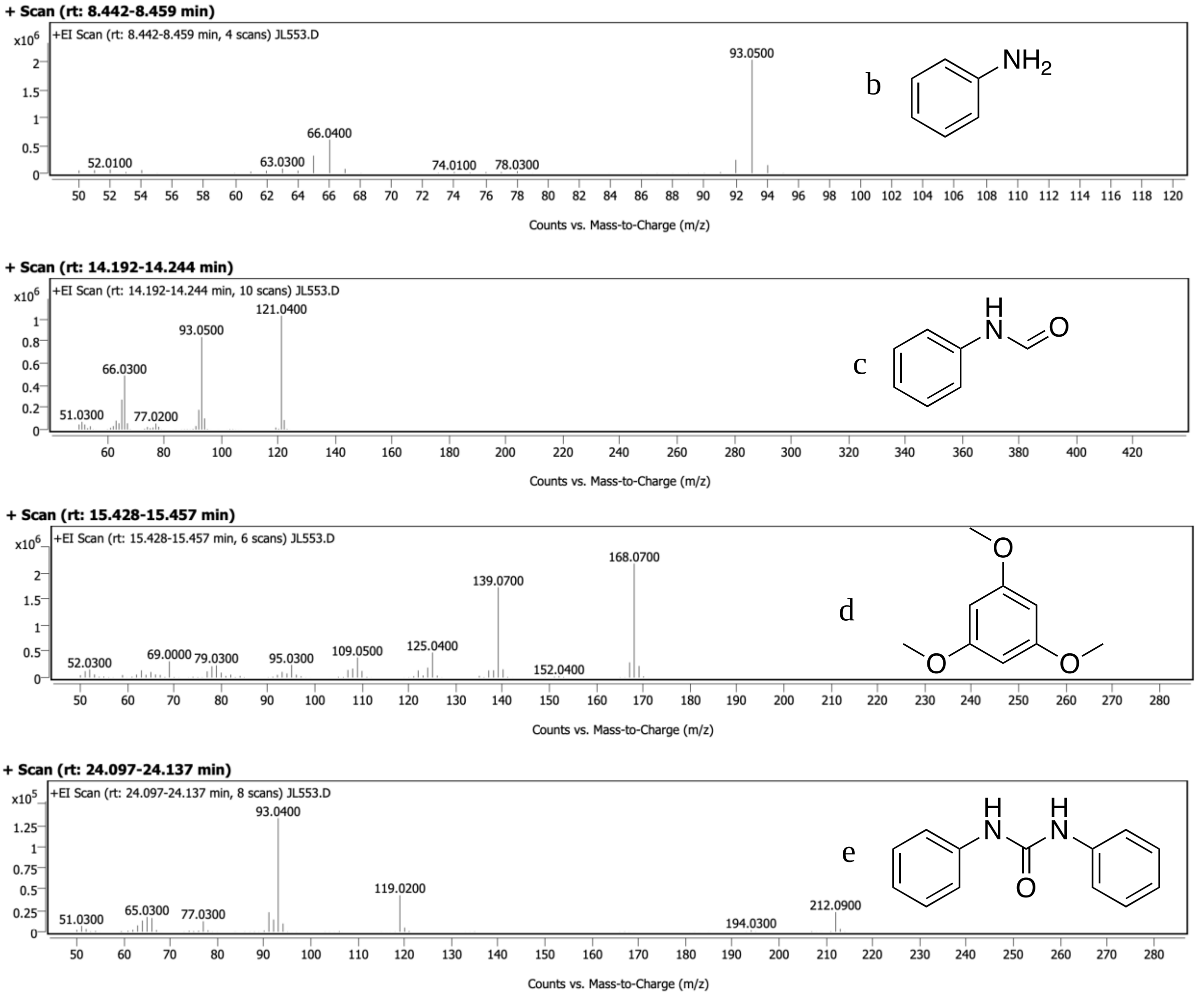

Figure S37: Mass spectra corresponding to gas chromatogram of crude reaction mixture from Table 2, entry 3 (Figure S29).

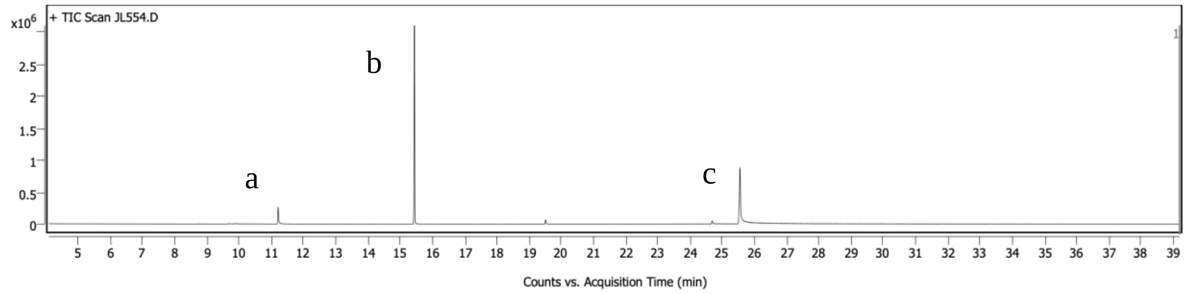

Figure S38: Gas chromatogram of crude reaction mixture from Table 2, entry 4.

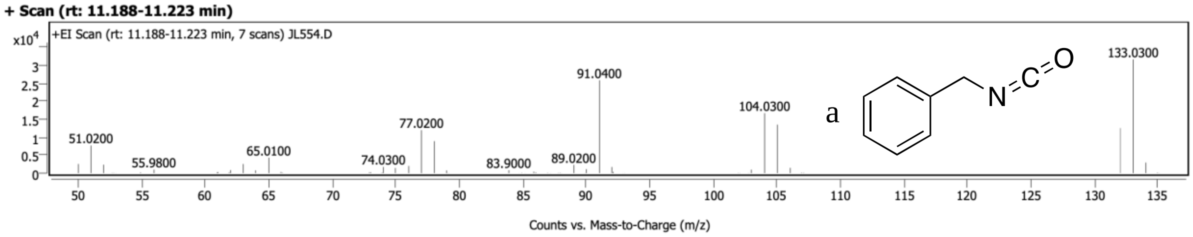

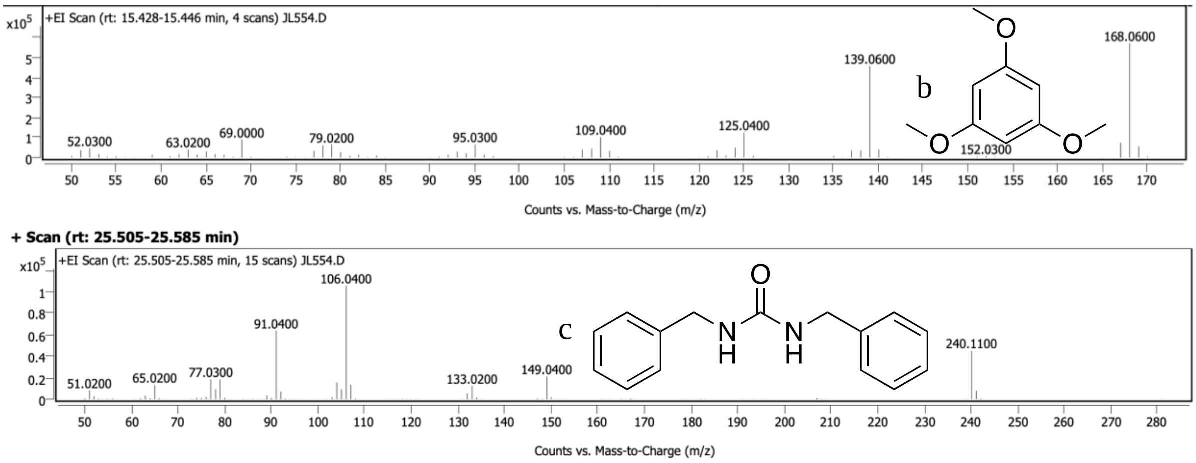

Figure S39: Mass spectra corresponding to gas chromatogram of crude reaction mixture from Table 2, entry 4 (Figure S31).

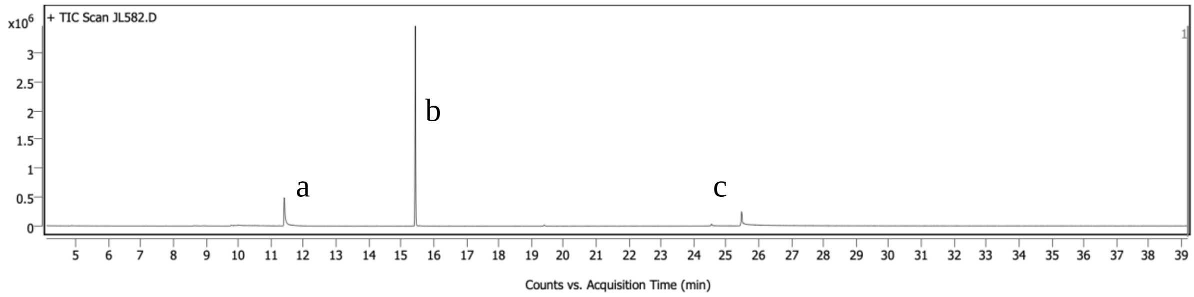

Figure S40: Gas chromatogram of crude reaction mixture from Table 2, entry 5.

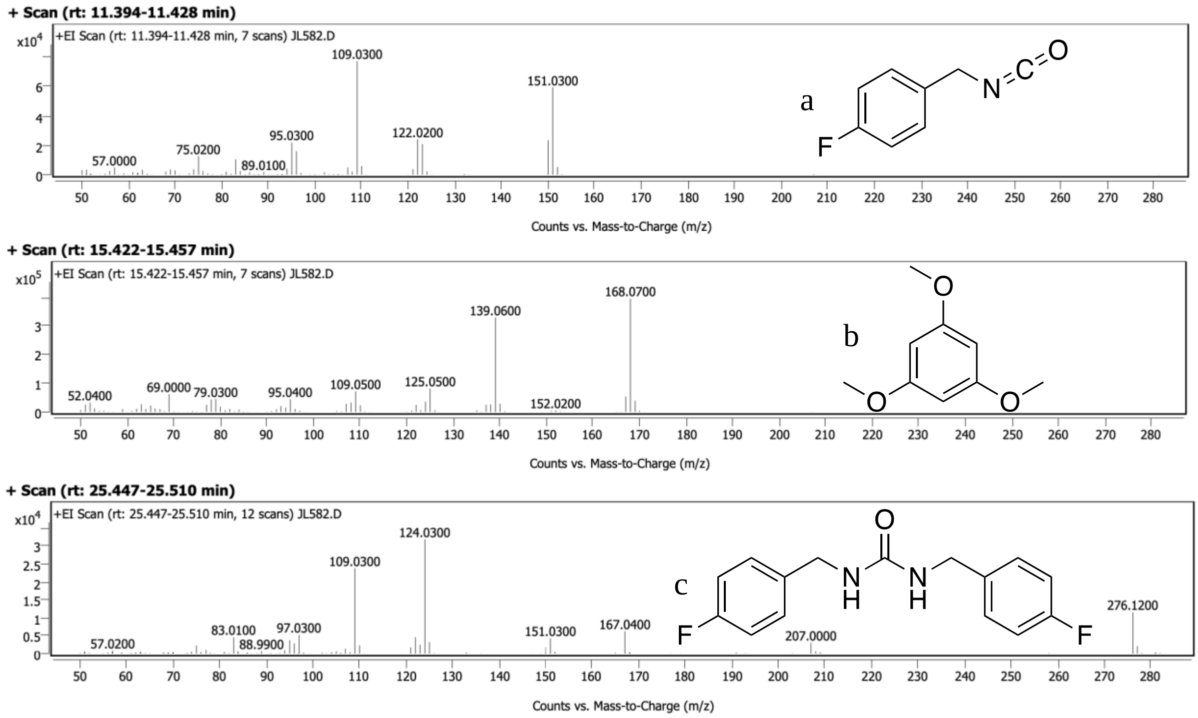

Figure S41: Mass spectra corresponding to gas chromatogram of crude reaction mixture from Table 2, entry 5 (Figure S33).

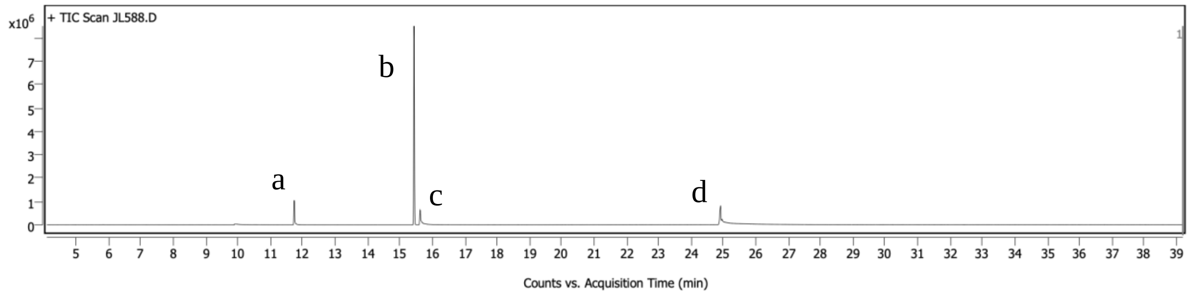

Figure S42: Gas chromatogram of the crude reaction mixture from Table 2, entry 6.

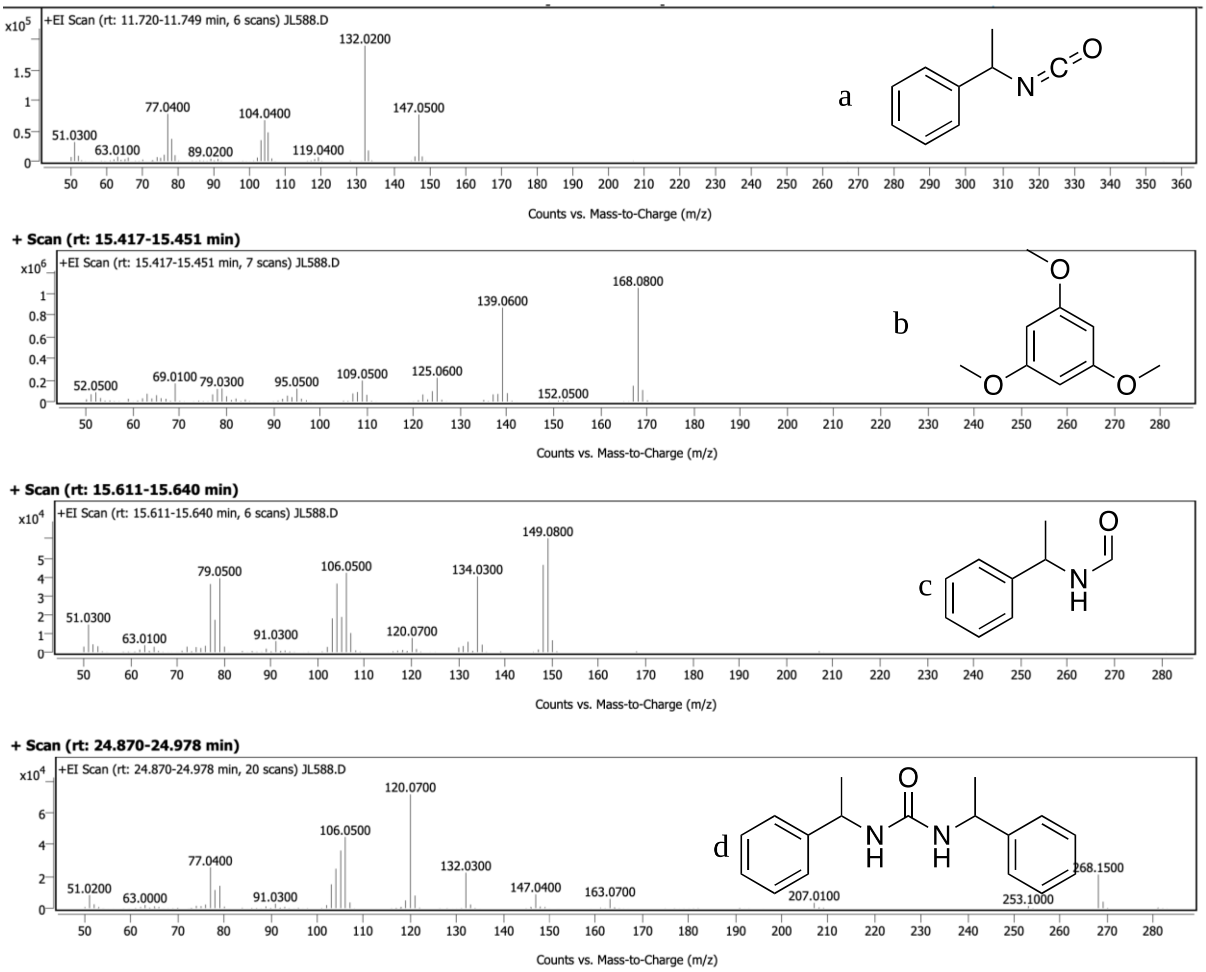

Figure S43: Mass spectra corresponding to gas chromatogram of the crude reaction mixture from Table 2, entry 6 (Figure S35).

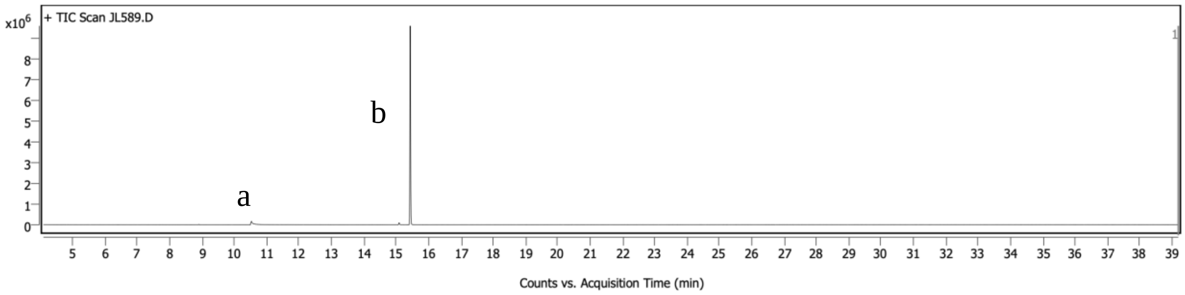

Figure S44: Gas chromatogram of the crude reaction mixture from Table 2, entry 7.

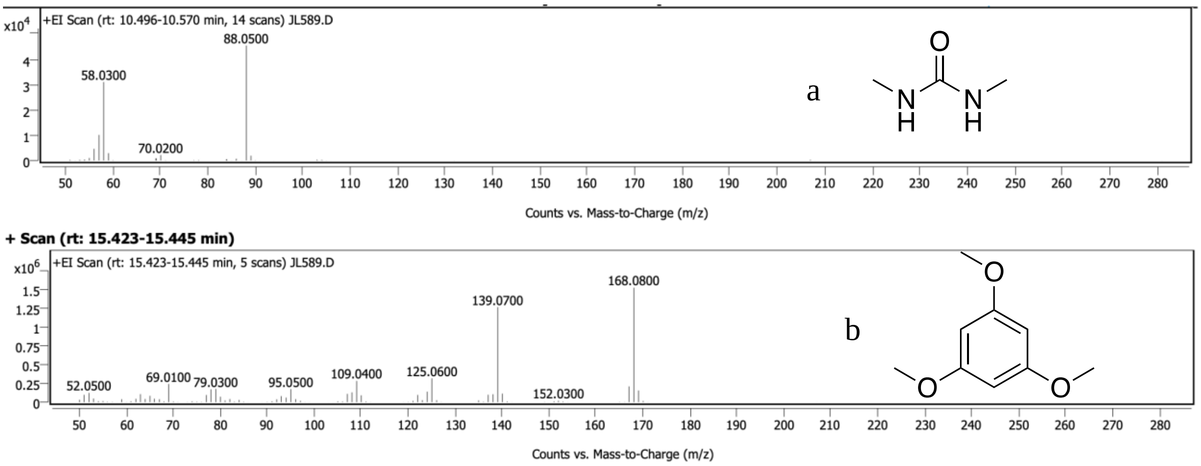

Figure S45: Mass spectra corresponding to the gas chromatogram of the crude reaction mixture from Table 2, entry 7 (Figure S37).

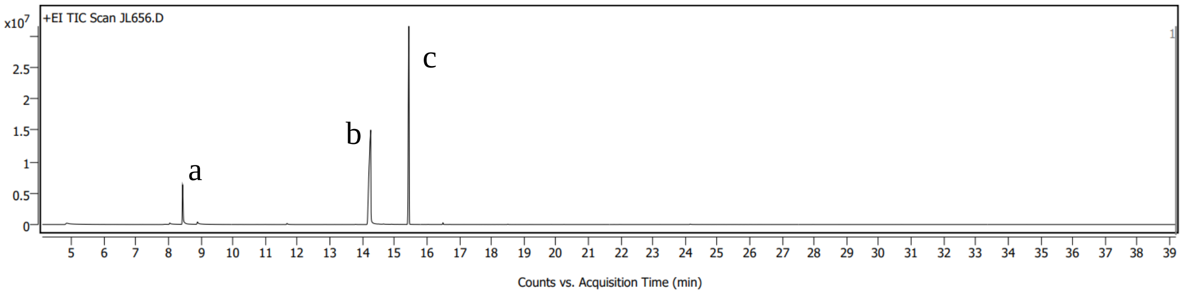

Figure S46: Gas chromatogram of the crude reaction mixture from Table 2, entry 7.

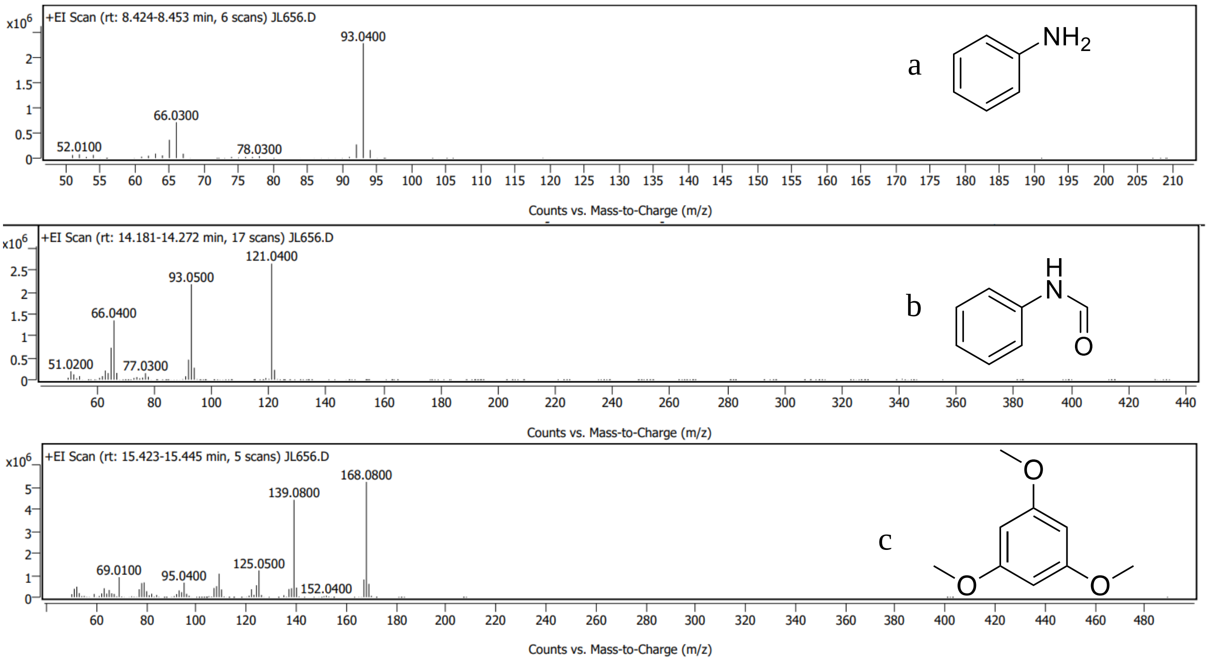

Figure S47: Mass spectra corresponding to gas chromatogram of the crude reaction mixture from Table 2, entry 6 (Figure S39).

3.2.4. GC-TCD data

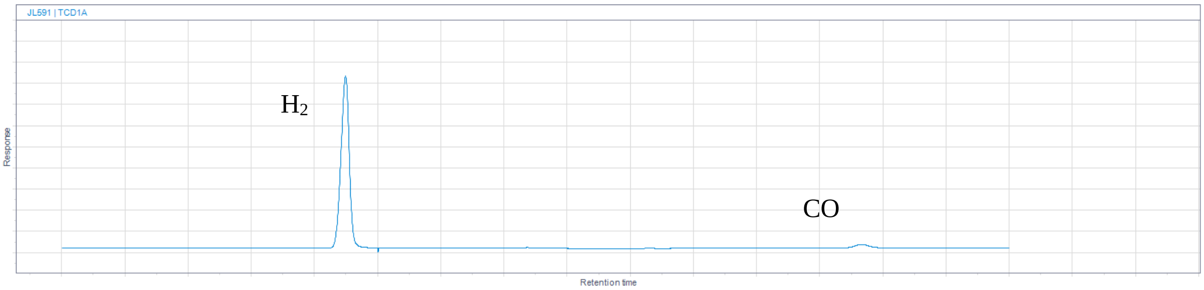

Figure S48: Representative gas chromatogram from the dehydrogenation/decarbonylation of formamide to form ureas (Table 2, entry 4).

3.2.5. ^1H and $^{13}C\{^1H\}$ NMR of isolated urea compounds

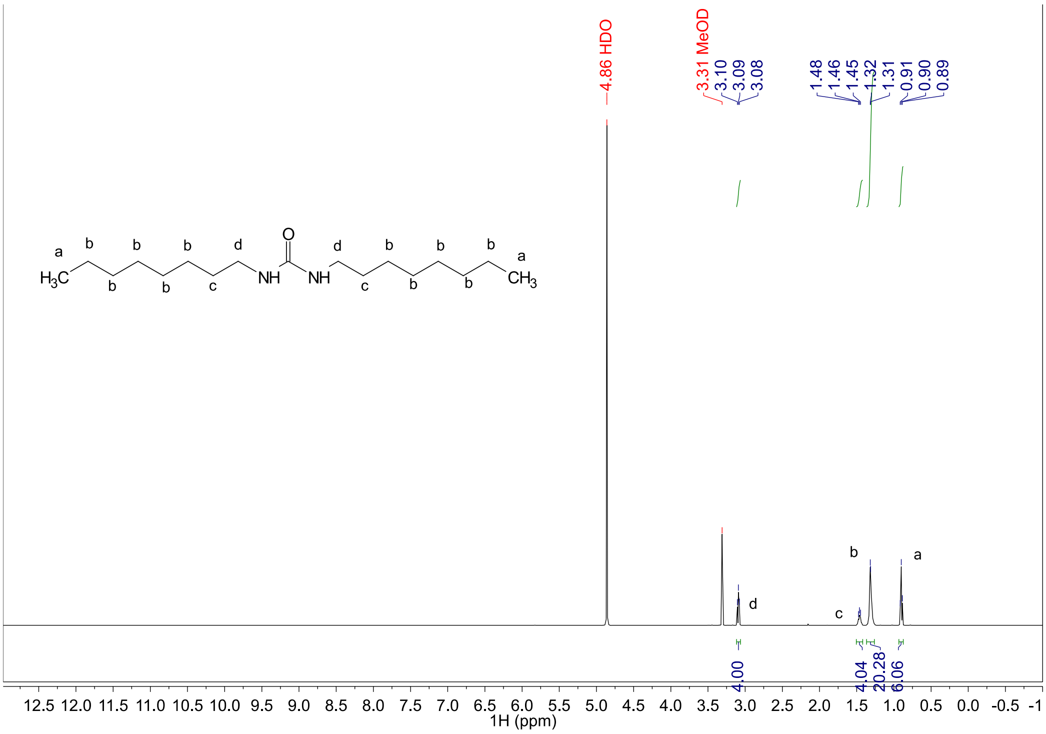

Figure S49: ^1H NMR spectrum (CD_3OD, 400 MHz, 298 K) of isolated N,N'-dioctylurea (Table 2, entry 1).

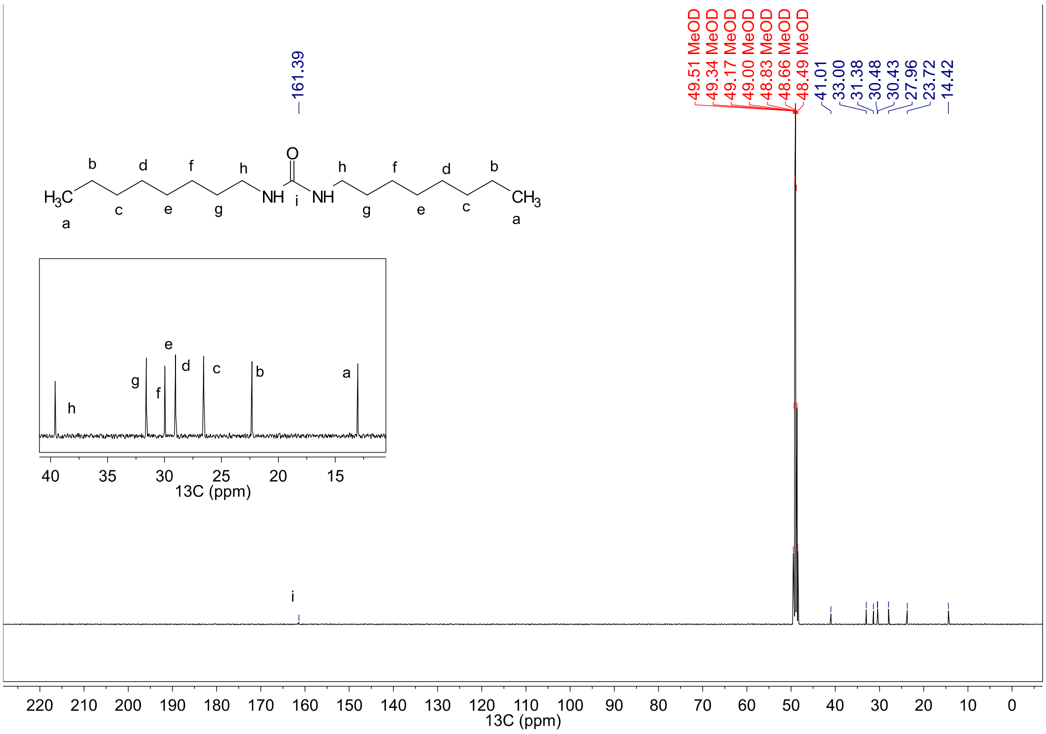

Figure S50: $^{13}C\{^{1}H\}$ NMR spectrum (CD_3OD, 126 MHz, 298 K) of isolated N,N'-dioctylurea (Table 2, entry 1).

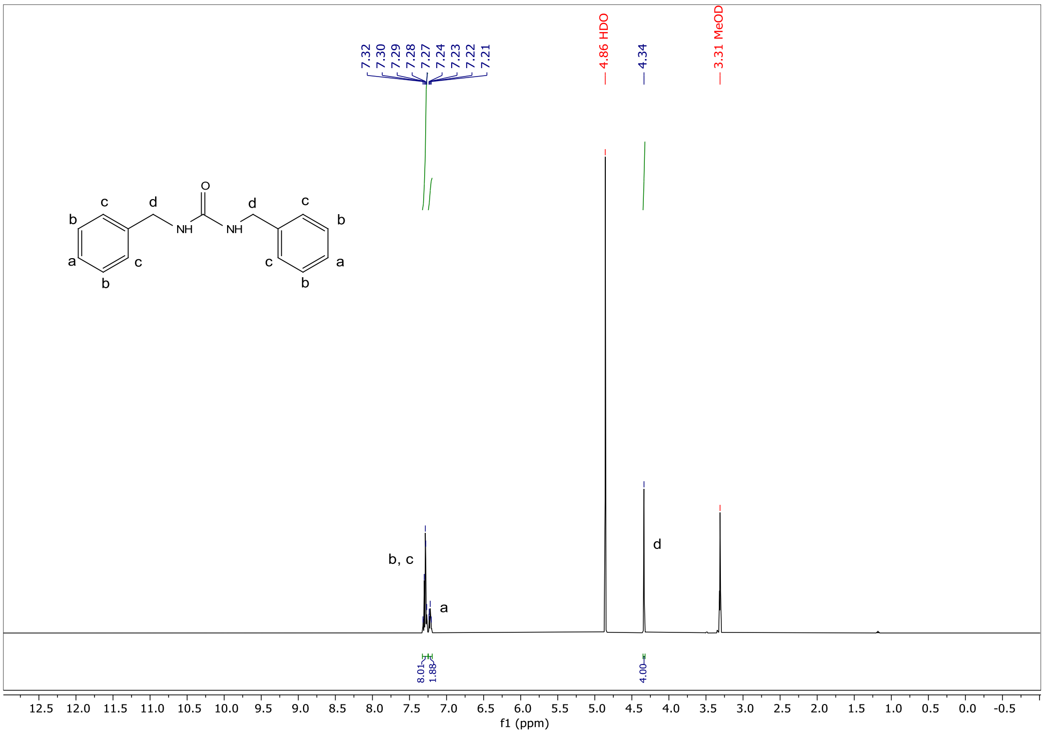

Figure S51: ^{1}H NMR spectrum (CD_3OD, 400 MHz, 298 K) of isolated 1,3-dibenzylurea (Table 2, entry 4).

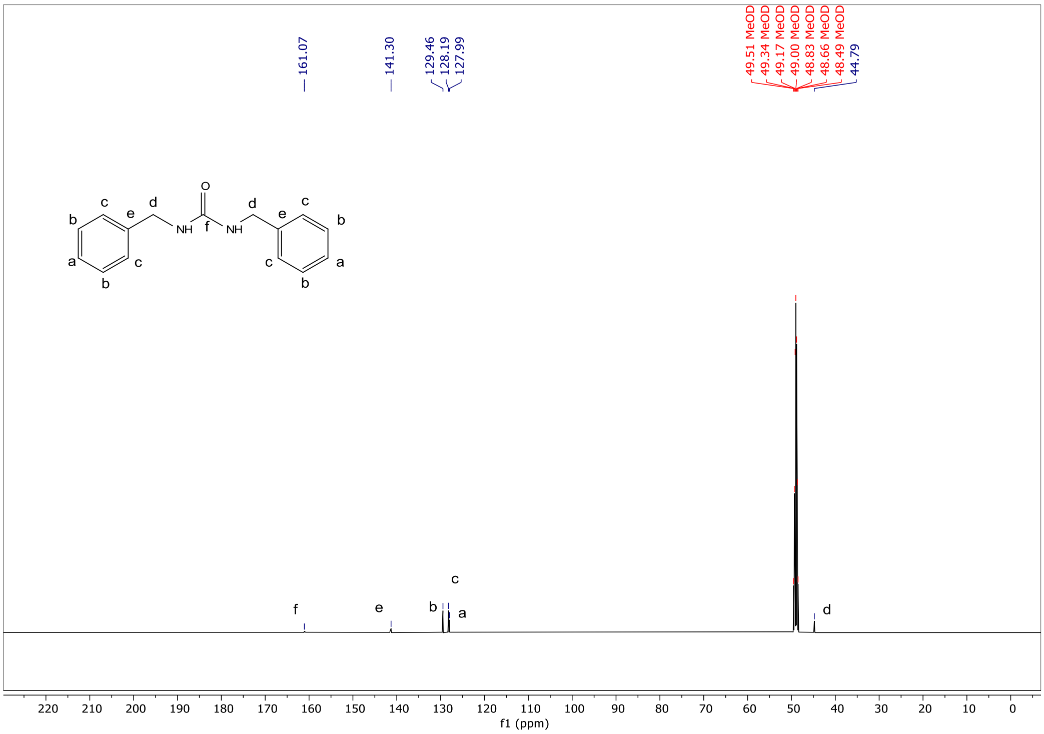

Figure S52: $^{13}C\{^{1}H\}$ NMR spectrum (CD_3OD, 126 MHz, 298 K) of isolated 1,3-dibenzylurea (Table 2, entry 4).

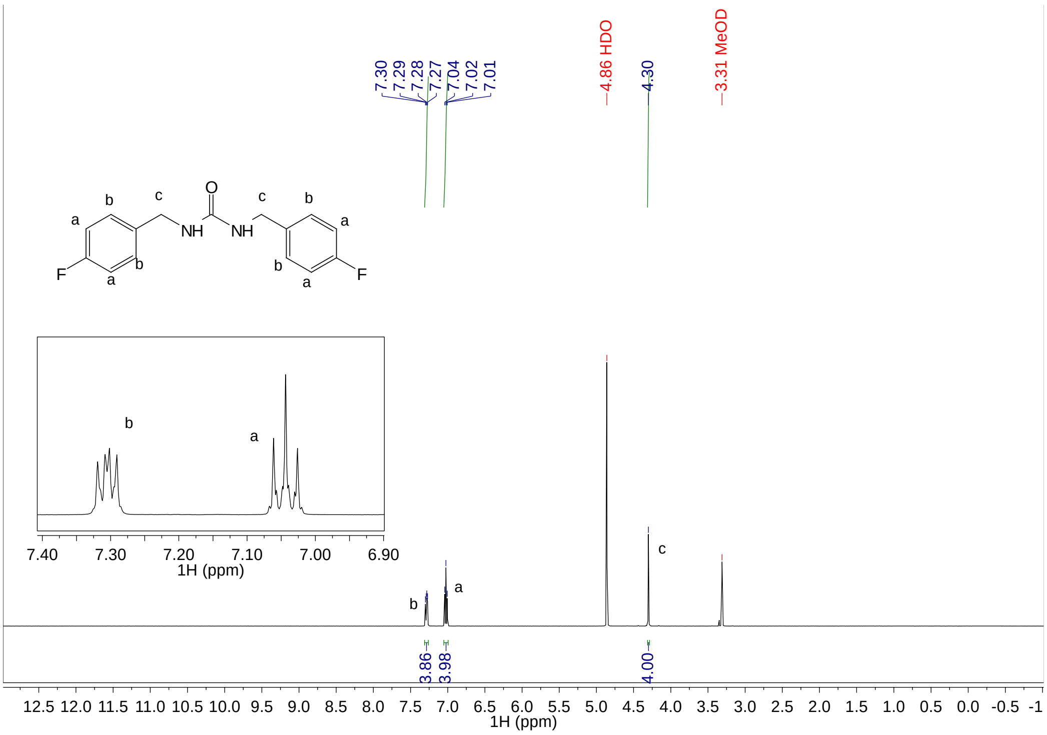

Figure S53: ^{1}H NMR spectrum (CD_3OD, 400 MHz, 298 K) of isolated 1,3-bis(4-fluorobenzyl)urea (Table 2, entry 5).

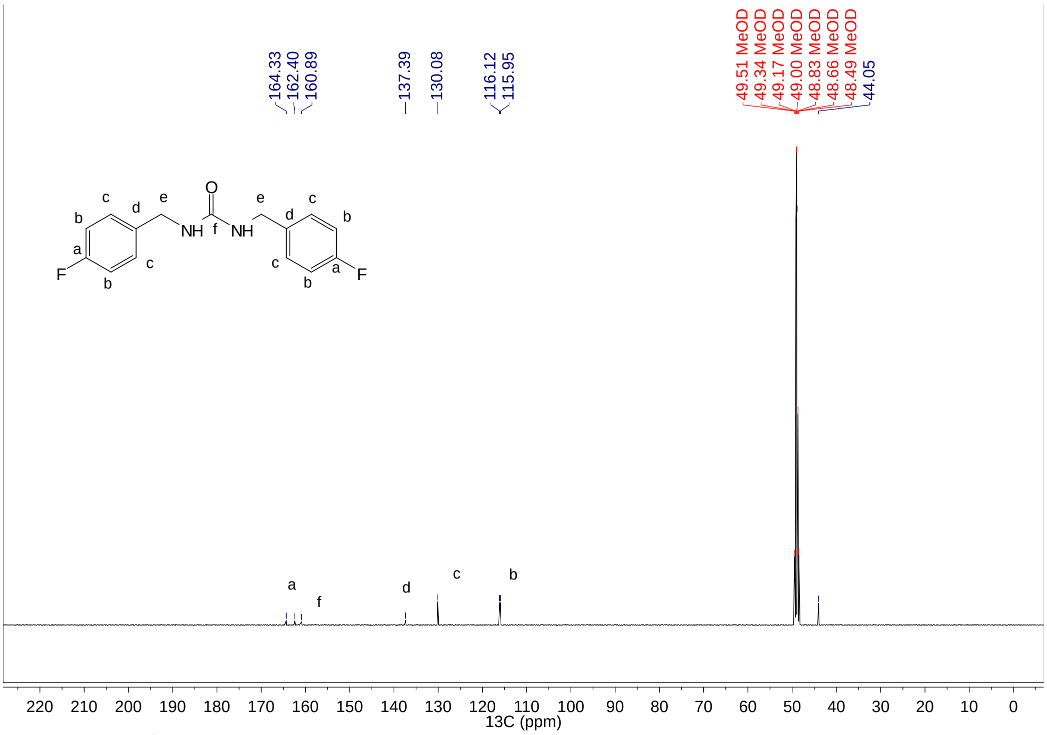

Figure S54: $^{13}C\{^{1}H\}$ NMR spectrum (CD_3OD, 126 MHz, 298 K) of isolated 1,3-bis(4-fluorobenzyl)urea (Table 2, entry 5).

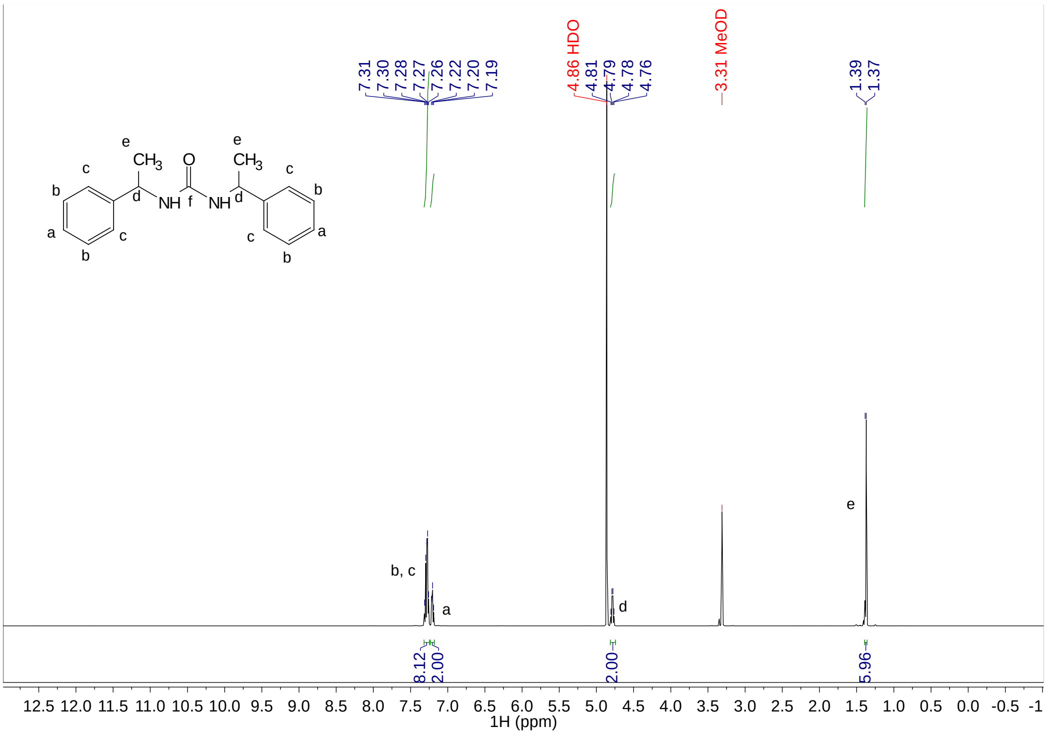

Figure S55: ^{1}H NMR spectrum (CD_3OD, 400 MHz, 298 K) of isolated 1,3-bis(1-phenylethyl)urea (Table 2, entry 6).

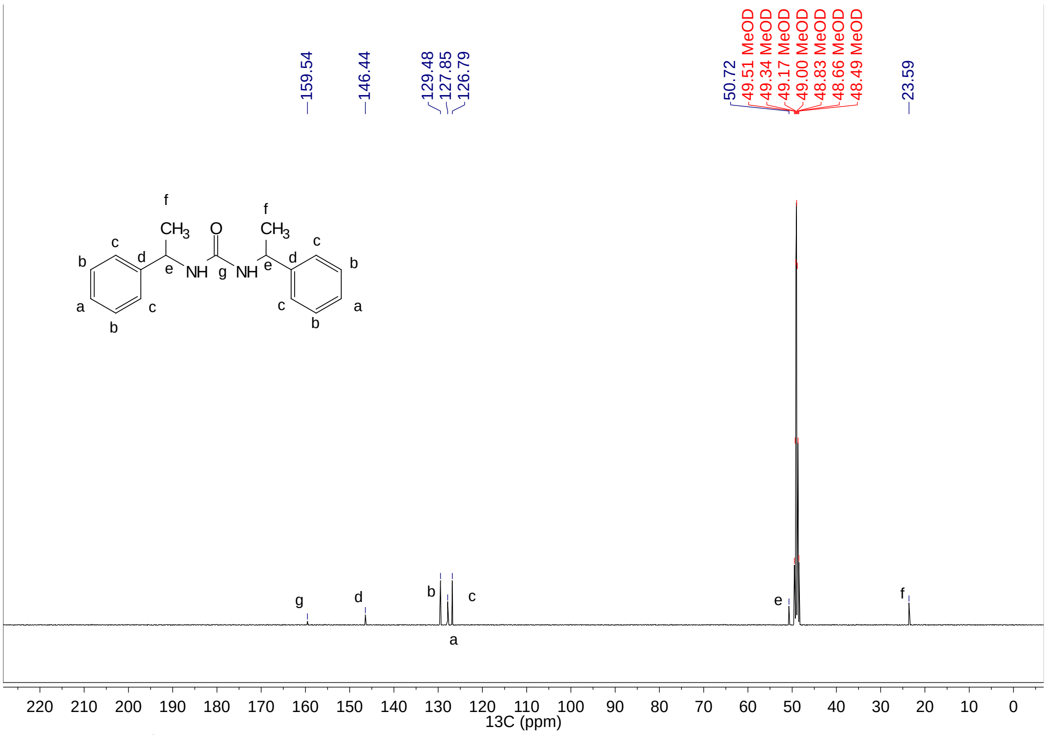

Figure S56: $^{13}C\{^{1}H\}$ NMR spectrum (CD_3OD, 126 MHz, 298 K) of isolated 1,3-bis(1-phenylethyl)urea (Table 2, entry 6).

3.2.6. GC-MS spectra of isolated urea compounds

Note: A signal resembling the corresponding isocyanate is present in the GC-MS spectra for the isolated ureas. It is expected that these signals arise from the cleavage of the urea bond in the GC-MS oven (425 °C).

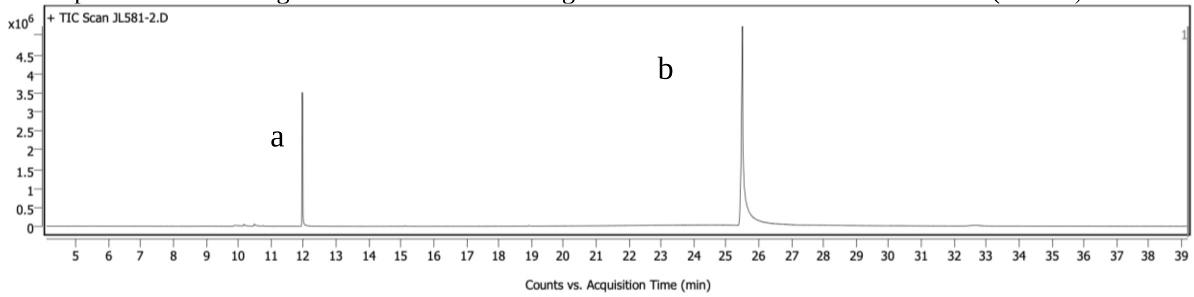

Figure S57: Gas chromatogram of isolated N,N'-dioctylurea (Table 2, entry 1).

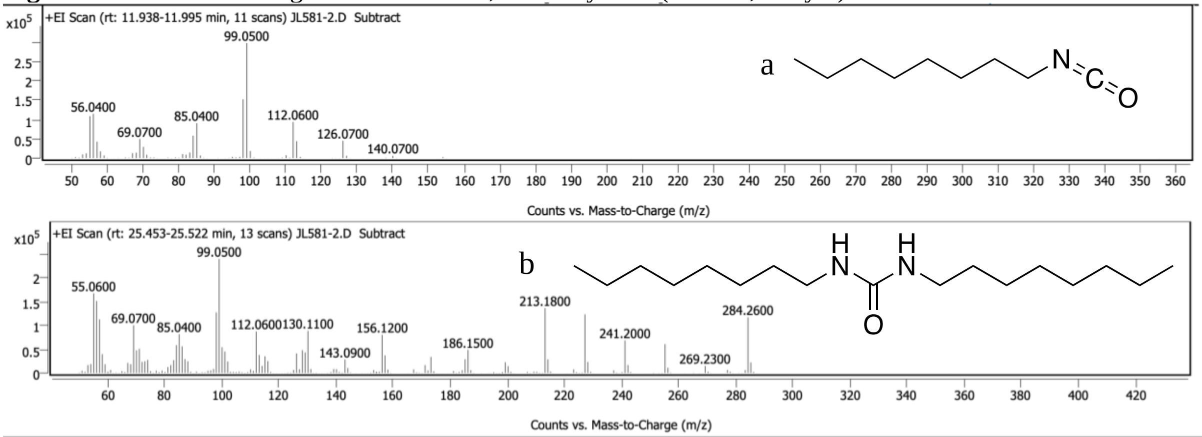

Figure S58: Mass spectra corresponding to the gas chromatogram of isolated N,N'-dioctylurea (Table 2, entry 1).

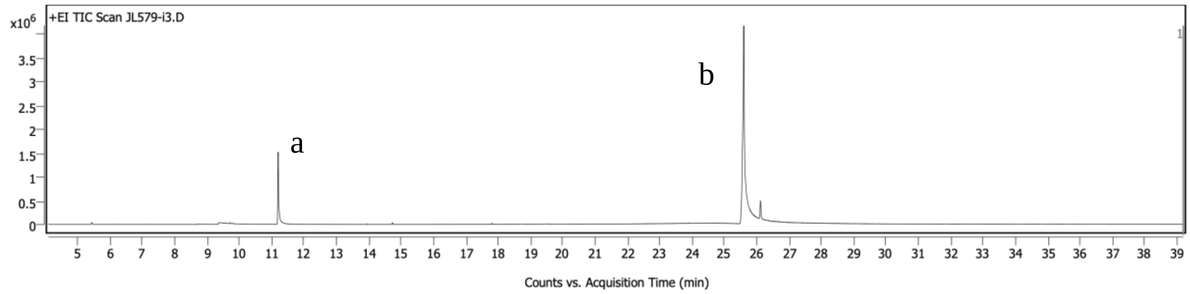

Figure S59: Gas chromatogram of isolated 1,3-dibenzylurea (Table 2, entry 4).

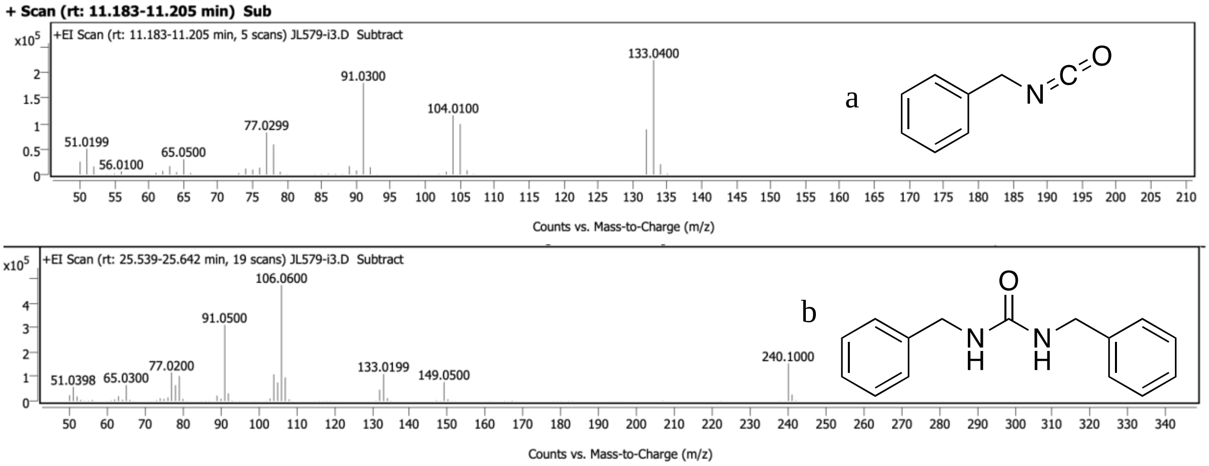

Figure S60: Mass spectra corresponding to gas chromatogram of isolated 1,3-dibenzylurea (Table 2, entry 4).

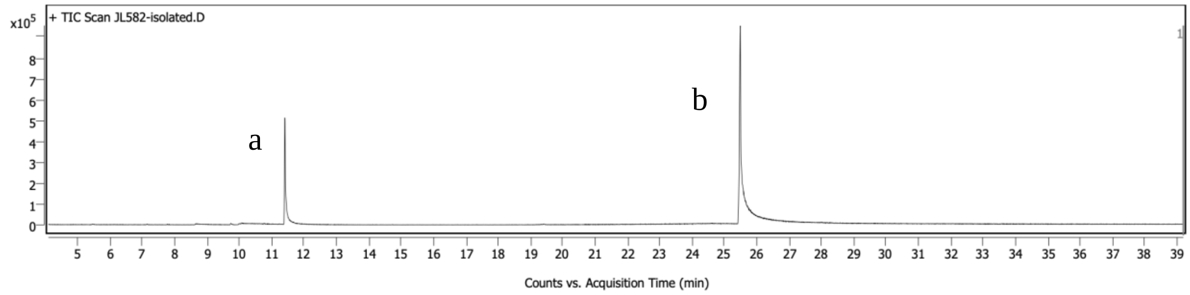

Figure S61: Gas chromatogram of isolated 1,3-bis(4-fluorobenzyl)urea (Table 2, entry 5).

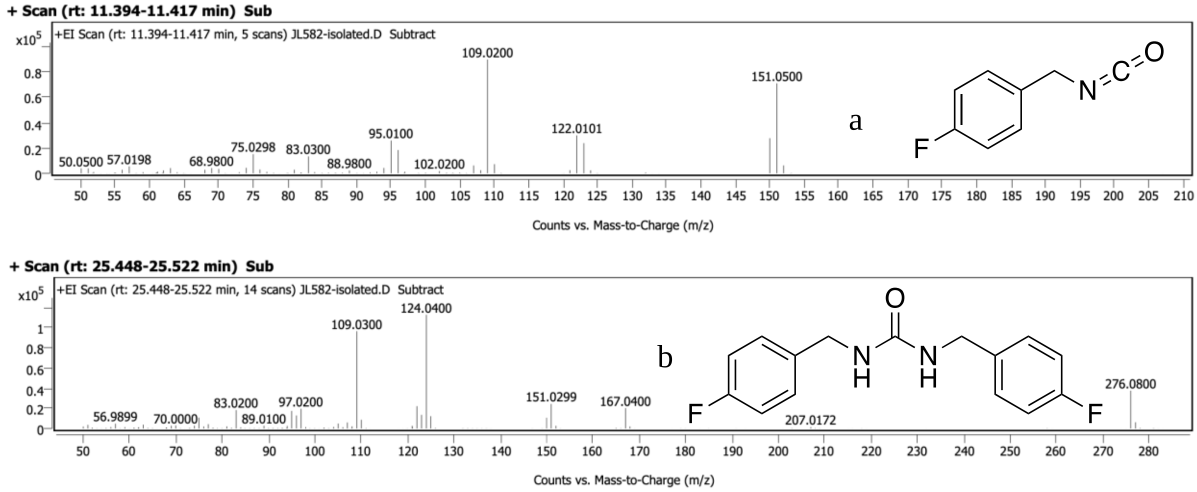

Figure S62: Mass spectra corresponding to gas chromatogram of isolated 1,3-bis(4-fluorobenzyl)urea corresponding to (Table 2, entry 5).

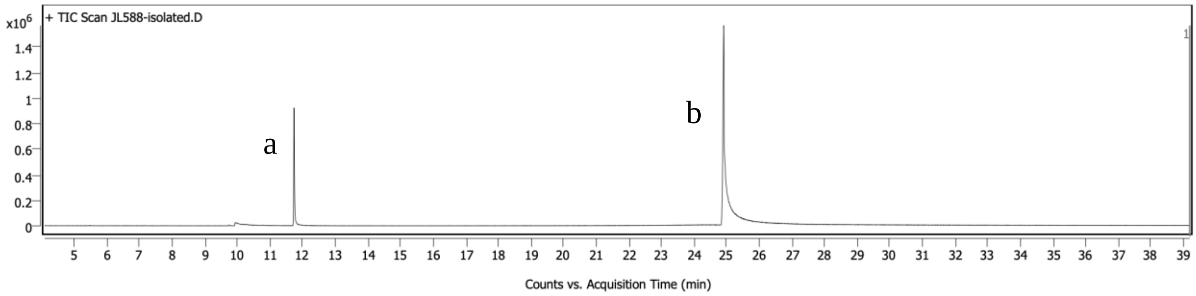

Figure S63: Gas chromatogram of isolated 1,3-bis(1-phenylethyl)urea (Table 2, entry 6)

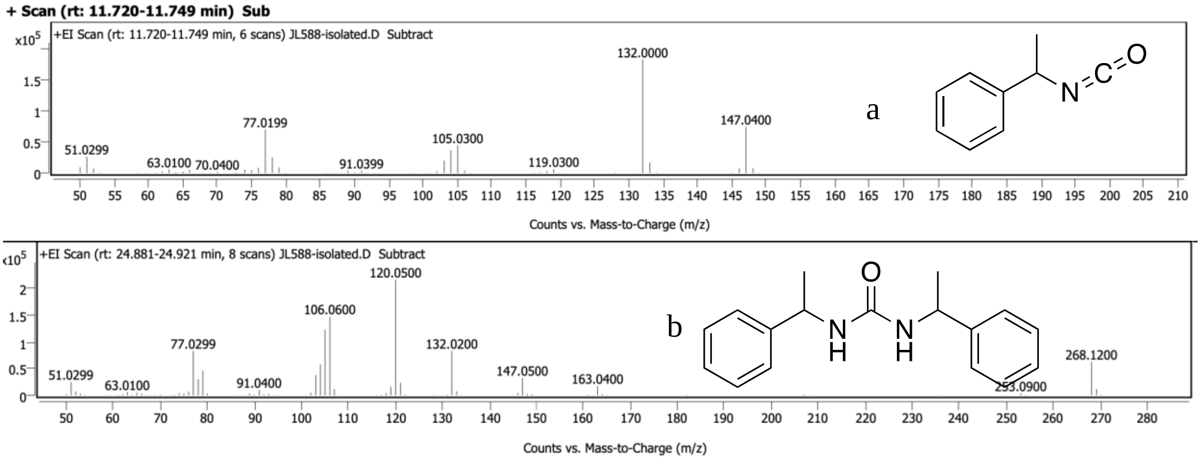

Figure S64: Mass spectra corresponding to gas chromatogram of isolated 1,3-bis(1,phenylethyl)urea (Table S1, entry 6).

3.2.7. IR spectra of isolated urea compounds

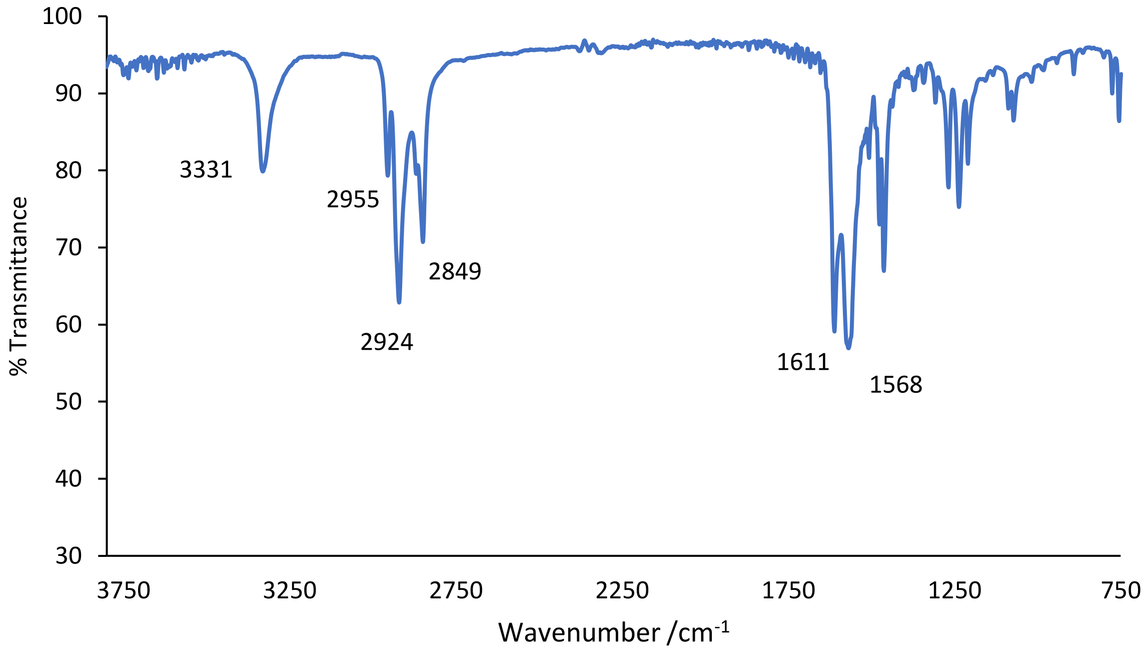

Figure S65: IR spectrum of isolated N,N'-dioctylurea (Table 2, entry 1).

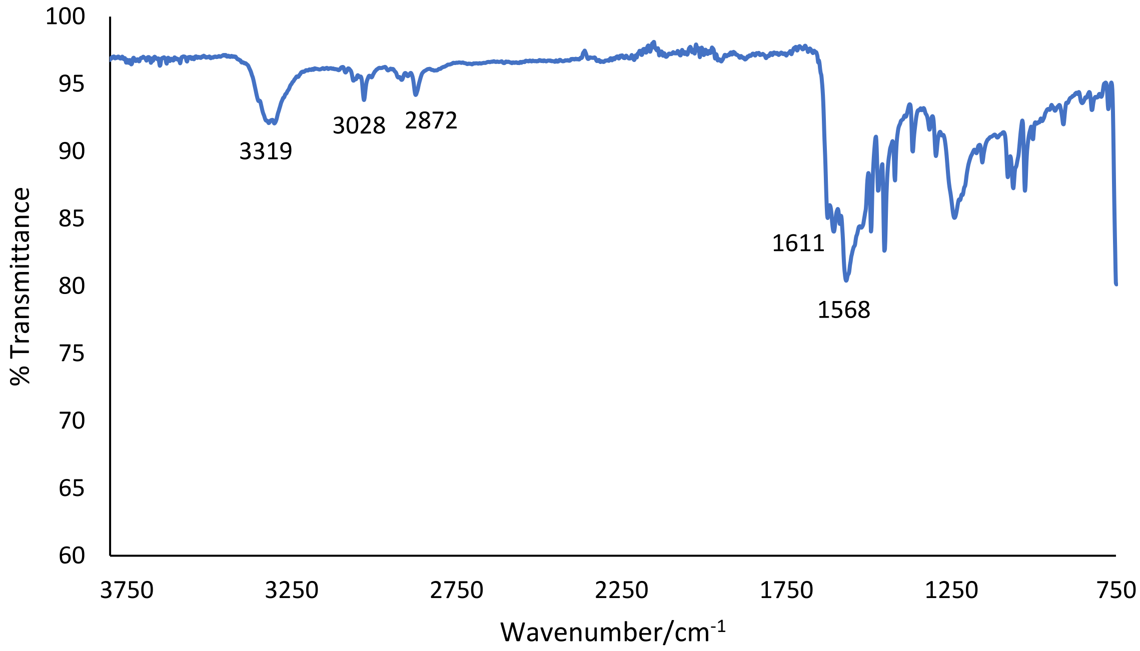

Figure S66: IR spectrum of isolated 1,3-dibenzylurea (Table S1, entry 4).

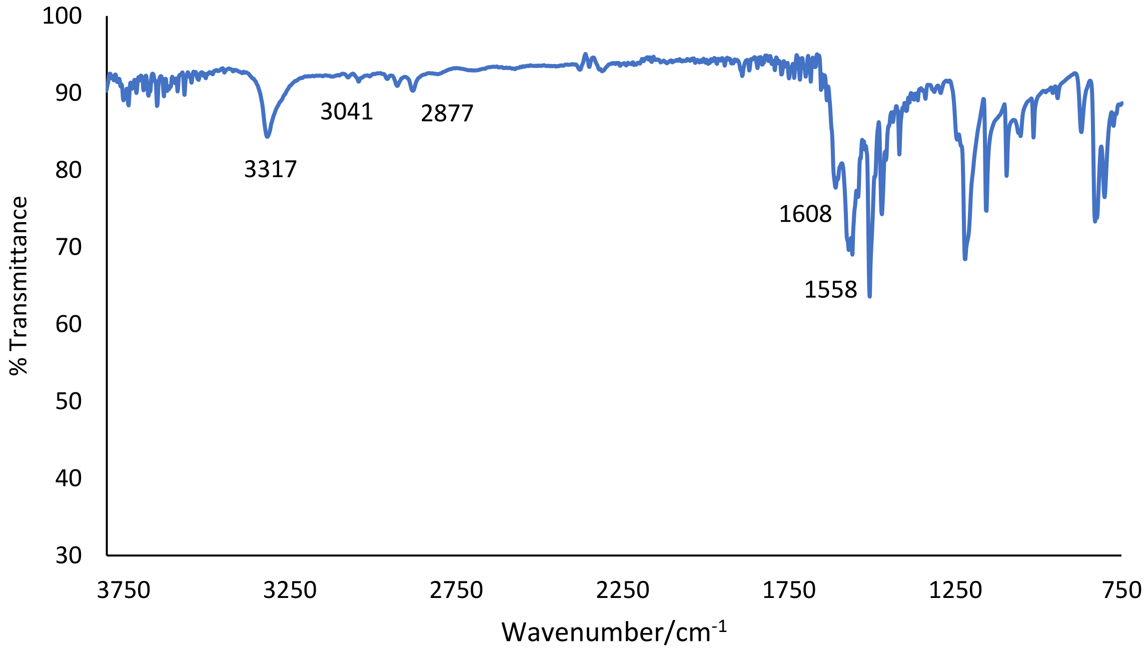

Figure S67: IR spectrum of isolated 1,3-bis(4-fluorobenzyl)urea (Table S1, entry 5).

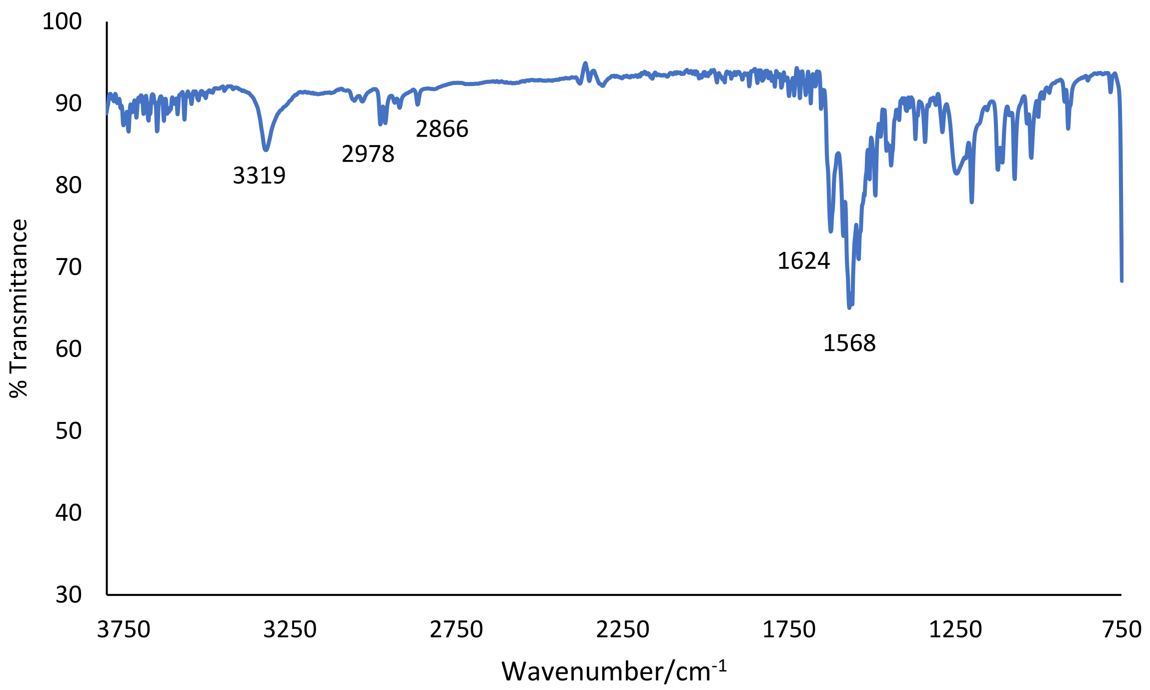

Figure S68: IR spectrum of 1,3-bis(1-phenylethyl)urea (Table S1, entry 6).

3.3. Characterisation data for polyurea synthesis

3.3.1. Characterisation data for isolated polyurea compounds

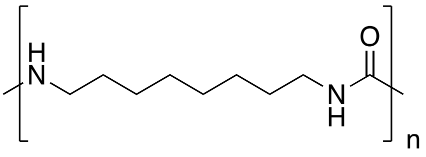

White solid (141.3 mg, 83%).
^{1}H NMR (500 MHz, *d*-TFA): δ_H 8.93 (0.17H, s), 8.64 (0.04H, s), 4.02 (0.36H, m), 3.91 (0.14H), 3.79 (3.58H, m), 2.11 (4H, m), 1.83 (8H, s).
^{13}C{^{1}H} NMR (126 MHz, *d*-TFA): δ_C 165.5, 158.9, 42.0, 41.3, 28.6, 28.3, 27.7, 26.1.
IR (ATR-FTIR, cm^{-1}): ν 3317 (NH), 2927 (CH), 2850 (CH), 1611 (C=O), 1568 (NH).

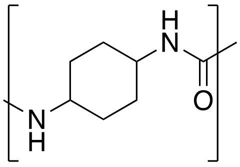

White solid (36 mg, 26%).
^{1}H NMR (500 MHz, *d*-TFA): δ_H 8.69-8.61 (2.98H, m), 4.33 (2.88H, m), 3.90 (1.05H, m), 2.49 (8H, m), 1.97-1.90 (8H, m).
^{13}C{^{1}H} NMR (126 MHz, *d*-TFA): δ_C 167.3, 164.9, 52.9, 49.2, 30.9, 29.3.
IR (ATR-FTIR, cm^{-1}): ν 3267 (NH), 3068 (CHO), 2933, 2856 (CH), 1647 (C=O), 1541 (NH).

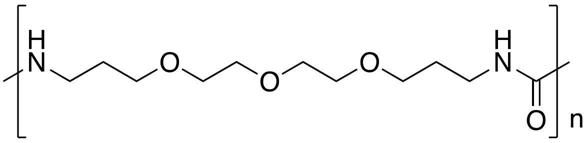

White solid (128.1 mg, 52%).
^{1}H NMR (500 MHz, *d*-TFA): δ_H 4.36-4.26 (12H, m), 3.96-3.94 (4H, m), 2.49-2.47 (4H, m).
^{13}C{^{1}H} NMR (126 MHz, *d*-TFA): δ_C 159.6, 69.5, 69.2, 68.6, 38.9, 27.5.
IR (ATR-FTIR, cm^{-1}): ν 3319 (NH), 2868 (CH), 1595 (C=O).

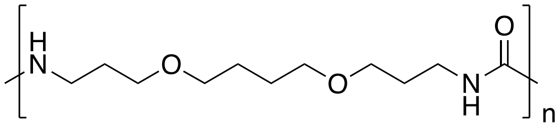

White solid (218.8 mg, 95%).
^{1}H NMR (500 MHz, *d*-TFA, δ_H): 8.76 (0.04H, s), 8.56 (0.01H, s), 4.15-4.08 (4H, m), 3.99 (0.05H, m), 3.93 (0.02H, m), 3.85-3.82 (2H, m), 2.38-2.36 (2H, m), 2.11 (2H, m).
^{13}C{^{1}H} NMR (126 MHz, *d*-TFA, δ_C): 165.8, 159.9, 71.1, 67.7, 38.6, 37.7, 27.6, 27.1, 24.7.
IR (ATR, ν_{max}/cm^{-1}): 3319 (NH), 2937, 2860, 2798 (CH), 1611 (C=O), 1568 (NH).

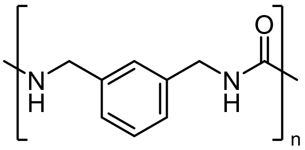

White solid (135 mg, 83%)
^{1}H NMR (500 MHz, *d*-TFA, δ_H): 8.94-8.77 (0.65H, m), 7.86-7.67 (4H, m), 5.12-5.04 (1.38H, m), 4.96-4.95 (2.54H, m).
^{13}C{^{1}H} NMR (126 MHz, *d*-DMSO, δ_C): 165.6, 159.5, 135.4, 130.0, 128.0, 127.6, 126.6, 45.2, 43.8.
IR (ATR, ν_{max}/cm^{-1}): 3312 (NH), 2916 (CH), 1616 (C=O), 1574 (NH).

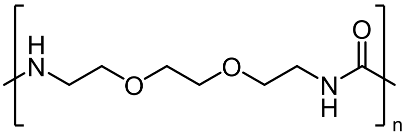

White solid (153 mg, 88%)
^{1}H NMR (500 MHz, *d*-TFA, δ_H): 8.85 (0.19H, s), 8.68 (0.08H, s), 4.37-4.32 (8H, m), 4.26-4.21 (0.52H, m), 4.09-4.08 (4H, m).
^{13}C{^{1}H} NMR (126 MHz, *d*-TFA, δ_C): 169.0, 166.1, 70.1, 69.8, 69.4, 68.5, 41.9, 41.6, 39.1.
IR (ATR, ν_{max}/cm^{-1}): 3358 (NH), 2884, 2864 (CH), 1613 (C=O), 1587 (NH).

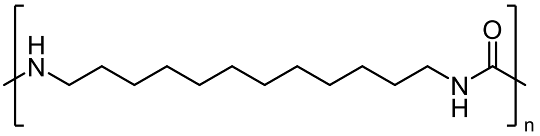

White solid (211 mg, 93%)
^{1}H NMR (500 MHz, *d*-TFA, δ_H): 8.89 (0.28H, s), 8.62 (0.08, s), 3.99 (0.90H, m), 3.88 (0.25H, m), 3.75 (2.76H, m), 2.13-2.07 (4H, m), 1.78-1.74 (16H, m).
^{13}C{^{1}H} NMR (126 MHz, *d*-TFA, δ_C): 1.67, 165.3, 158.8, 44.8, 42.0, 41.2, 29.5, 29.1, 29.1, 29.0, 28.7, 28.6, 28.4, 27.6, 26.2, 25.9.
IR (ATR, ν_{max}/cm^{-1}):3333 (NH), 2920, 2845 (CH), 1611 (C=O), 1574 (NH).

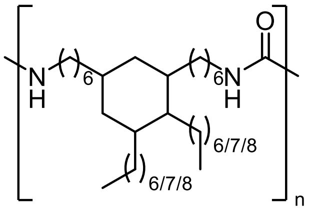

White solid (181 mg, 38%)
^{1}H NMR (500 MHz, *d*-DMSO, δ_H): 7.98 (0.99H, br s), 3.05 (1.67H, br s), 1.37-0.86 (33.13H br s).
^{13}C{^{1}H} NMR (126 MHz, *d*-DMSO, δ_C): 161.3, 37.5, 29.5, 26.9, 22.7, 14.2.
IR (ATR, ν_{max}/cm^{-1}): 3270 (NH), 2920, 2851 (CH), 1661 (C=O).

3.3.2. ^{1}H NMR and ^{13}C{^{1}H} NMR spectra for polyurea synthesis

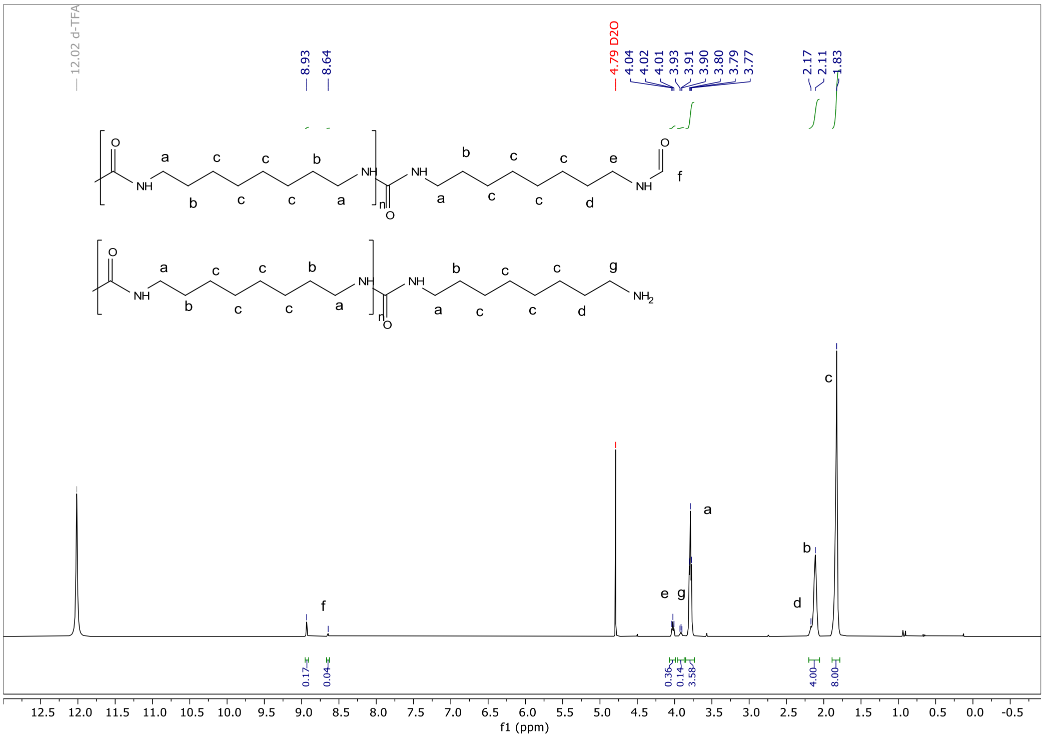

Figure S69: ^{1}H NMR spectrum (*d*-TFA, 500 MHz, 298 K) of Table 3, entry 1.

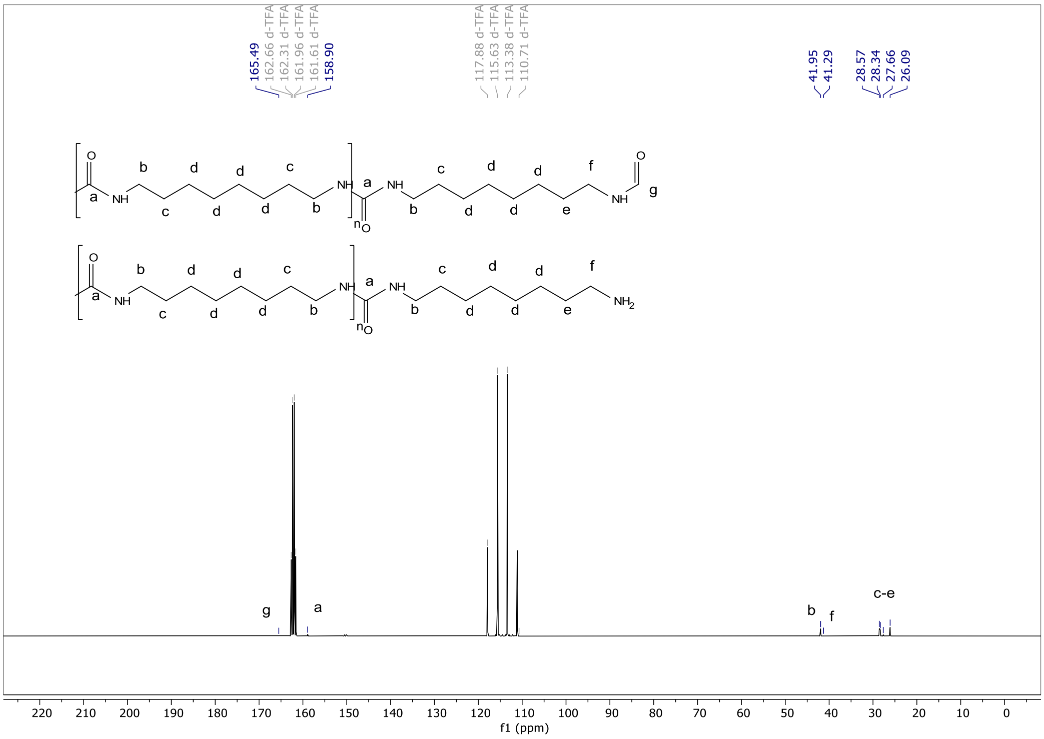

Figure S70: $^{13}C\{^{1}H\}$ NMR spectrum (*d*-TFA, 126 MHz, 298 K) of Table 3, entry 1.

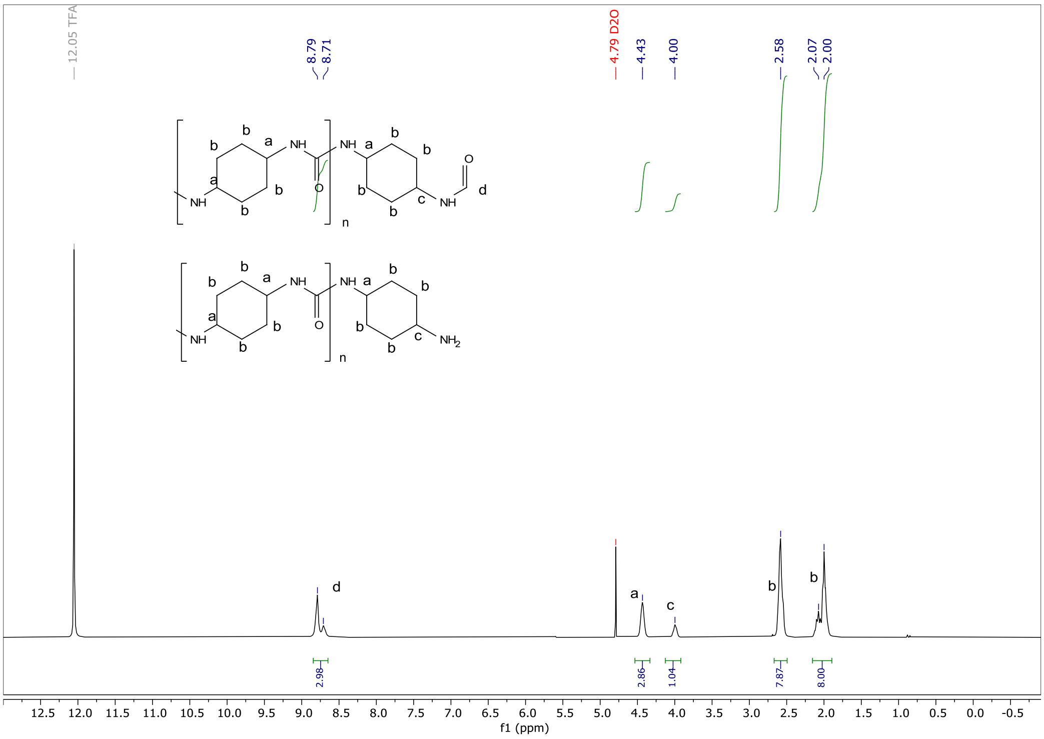

Figure S71: ^{1}H NMR spectrum (*d*-TFA, 500 MHz, 298 K) of Table 3, entry 2.

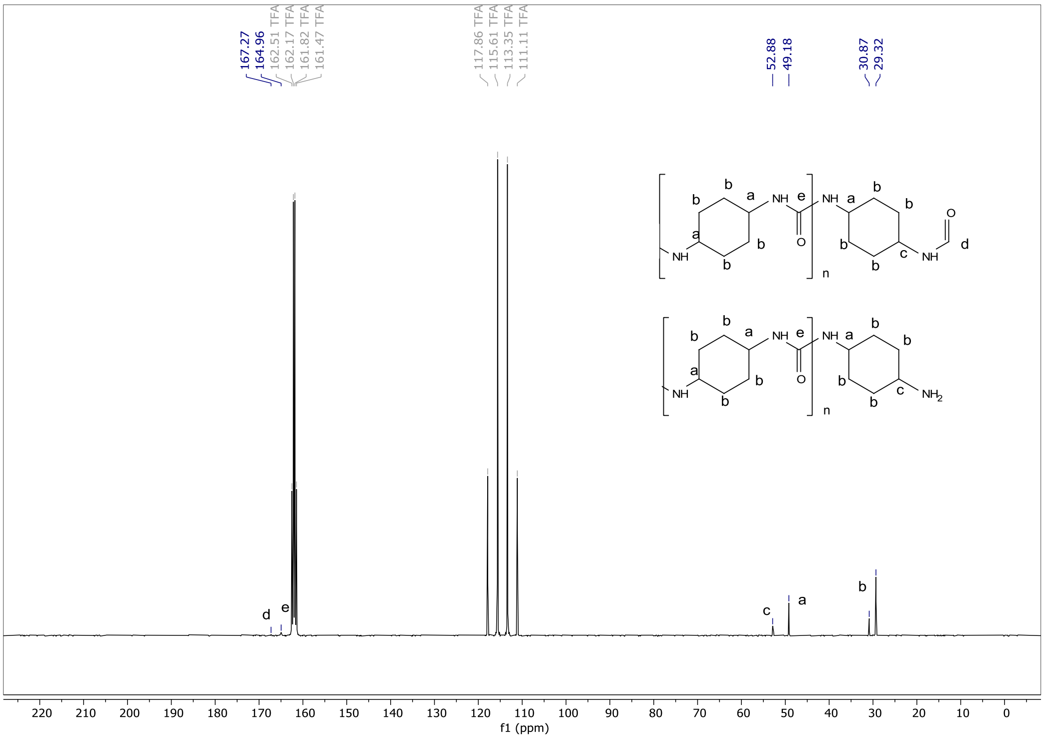

Figure S72: $^{13}C\{^1H\}$ NMR spectrum (*d*-TFA, 126 MHz, 298 K) of Table 3, entry 2.

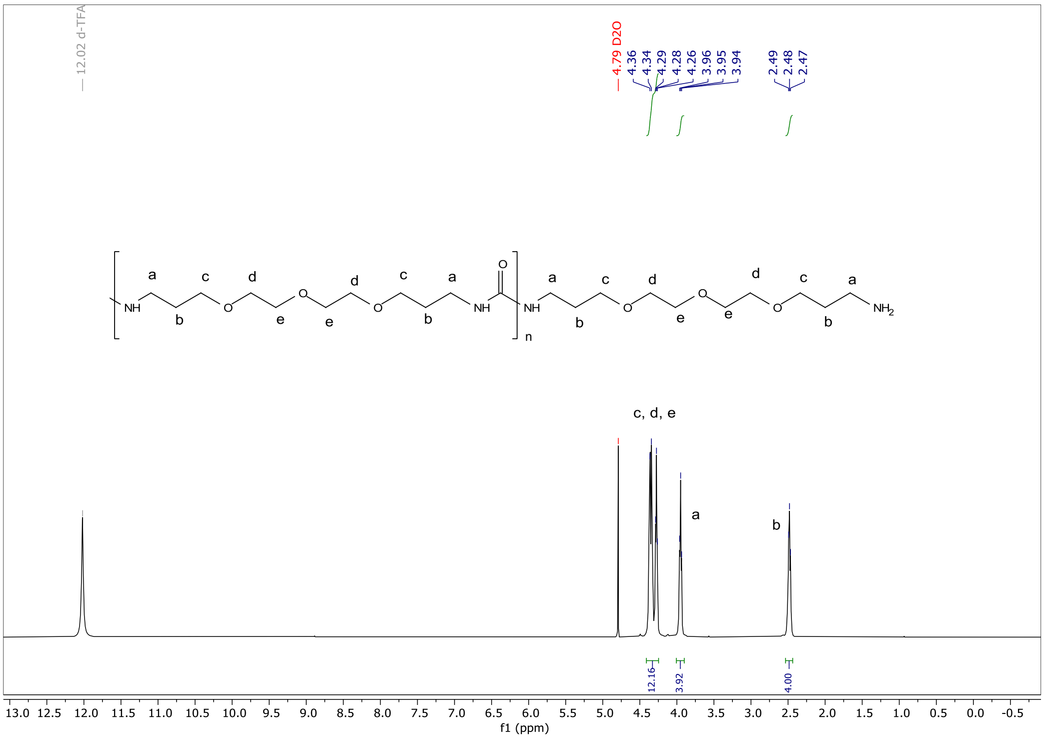

Figure S73: ^1H NMR spectrum (*d*-TFA, 500 MHz, 298 K) of Table 3, entry 3.

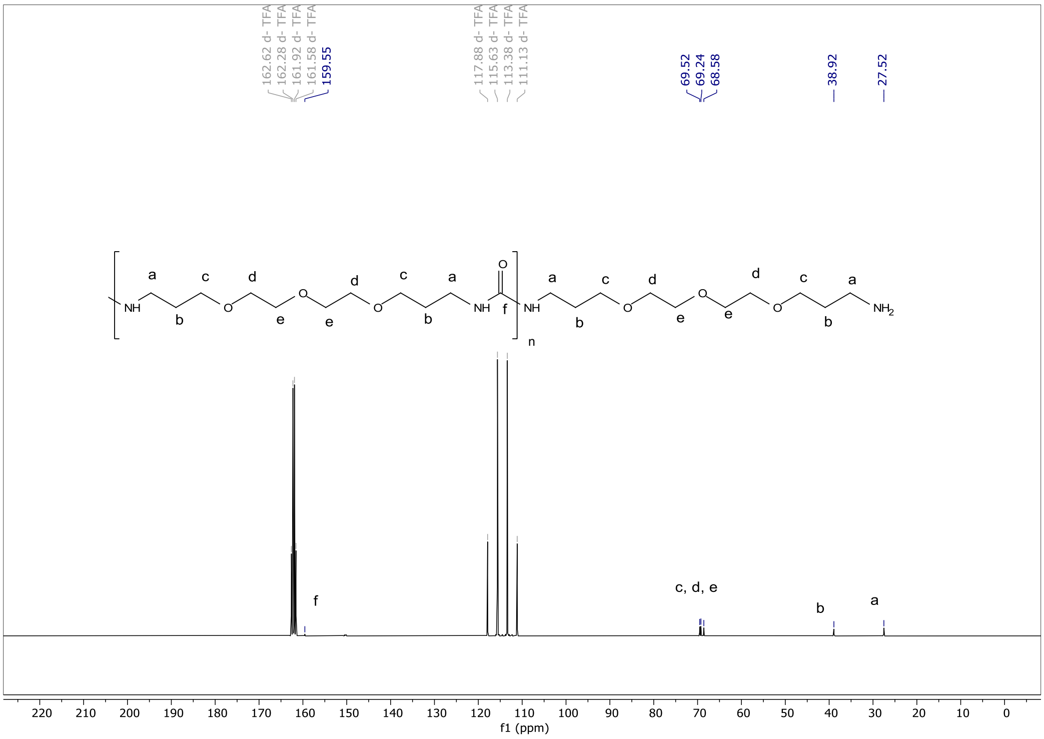

Figure S74: $^{13}C\{^1H\}$ NMR spectrum (*d*-TFA, 126 MHz, 298 K) of Table 3, entry 3.

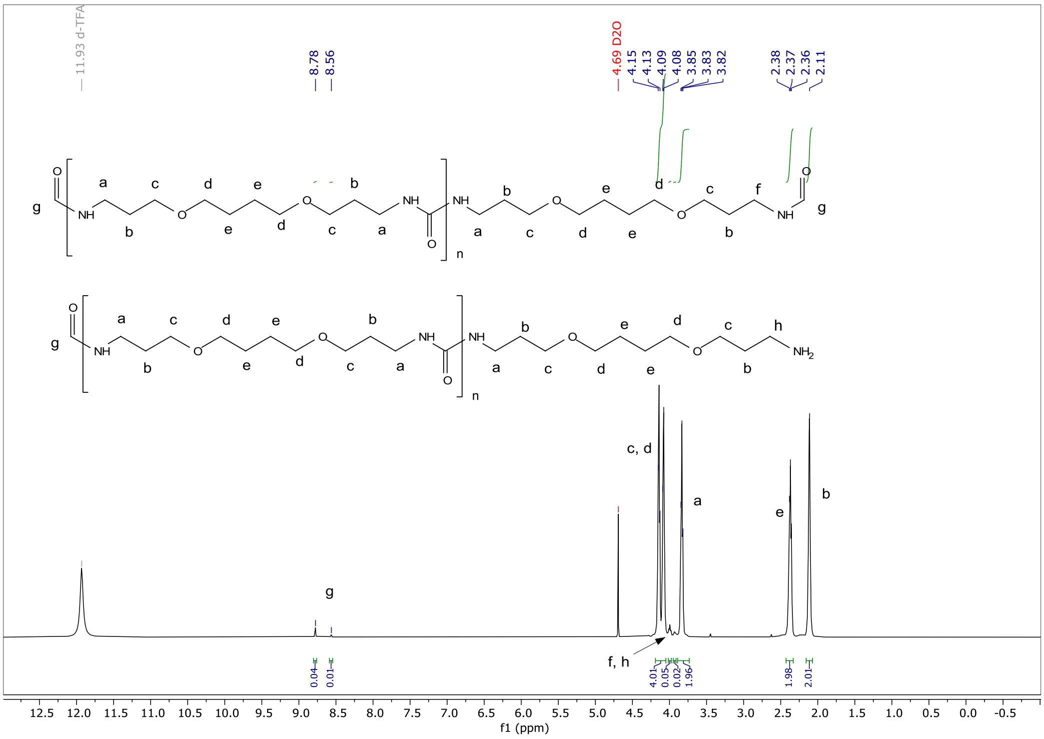

Figure S75: ^1H NMR spectrum (*d*-TFA, 500 MHz, 298 K) of Table 3, entry 4.

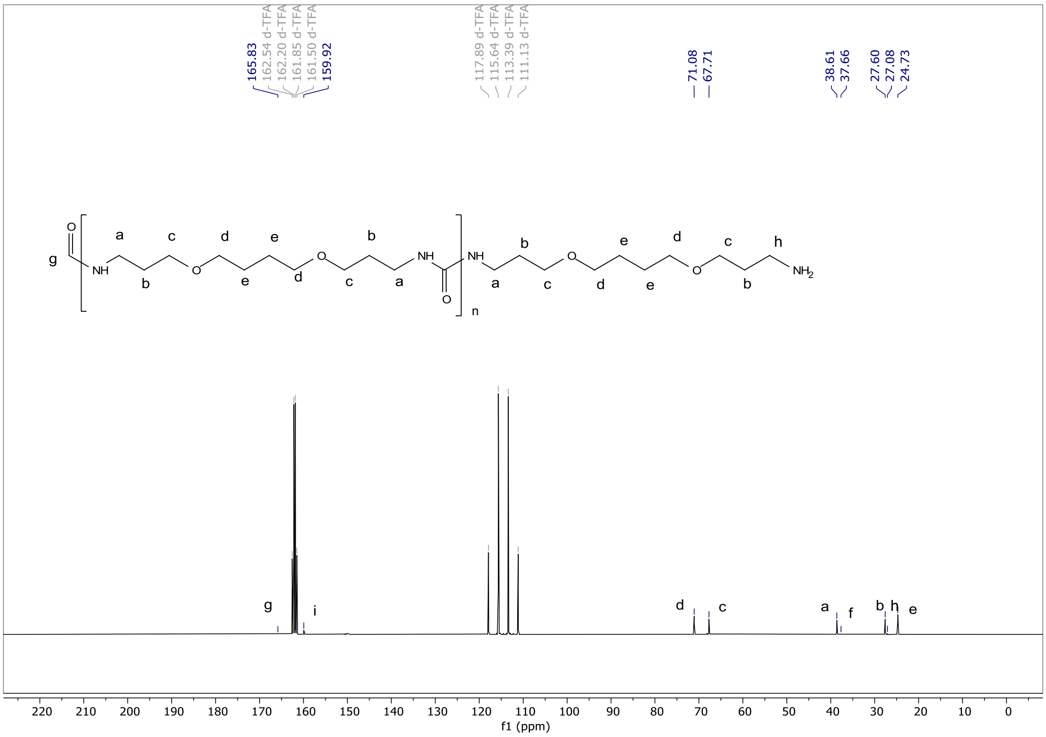

Figure S76: $^{13}C\{^1H\}$ NMR spectrum (*d*-TFA, 126 MHz, 298 K) of Table 3, entry 4.

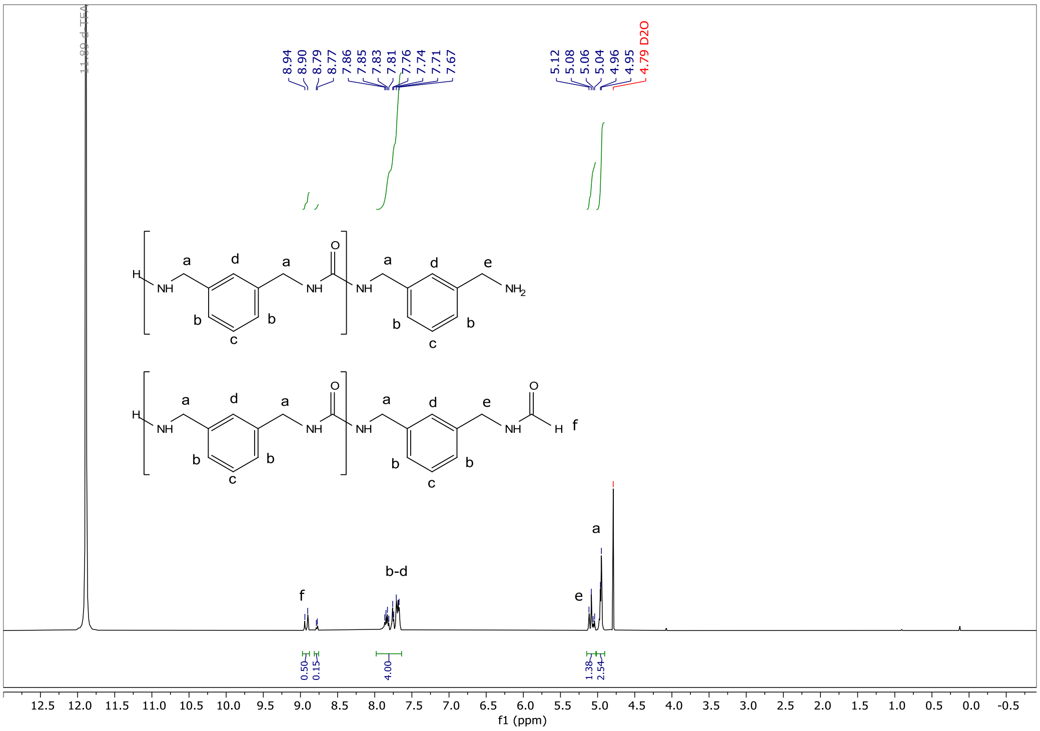

Figure S77: ^1H NMR spectrum (*d*-TFA, 500 MHz, 298 K) of Table 3, entry 5.

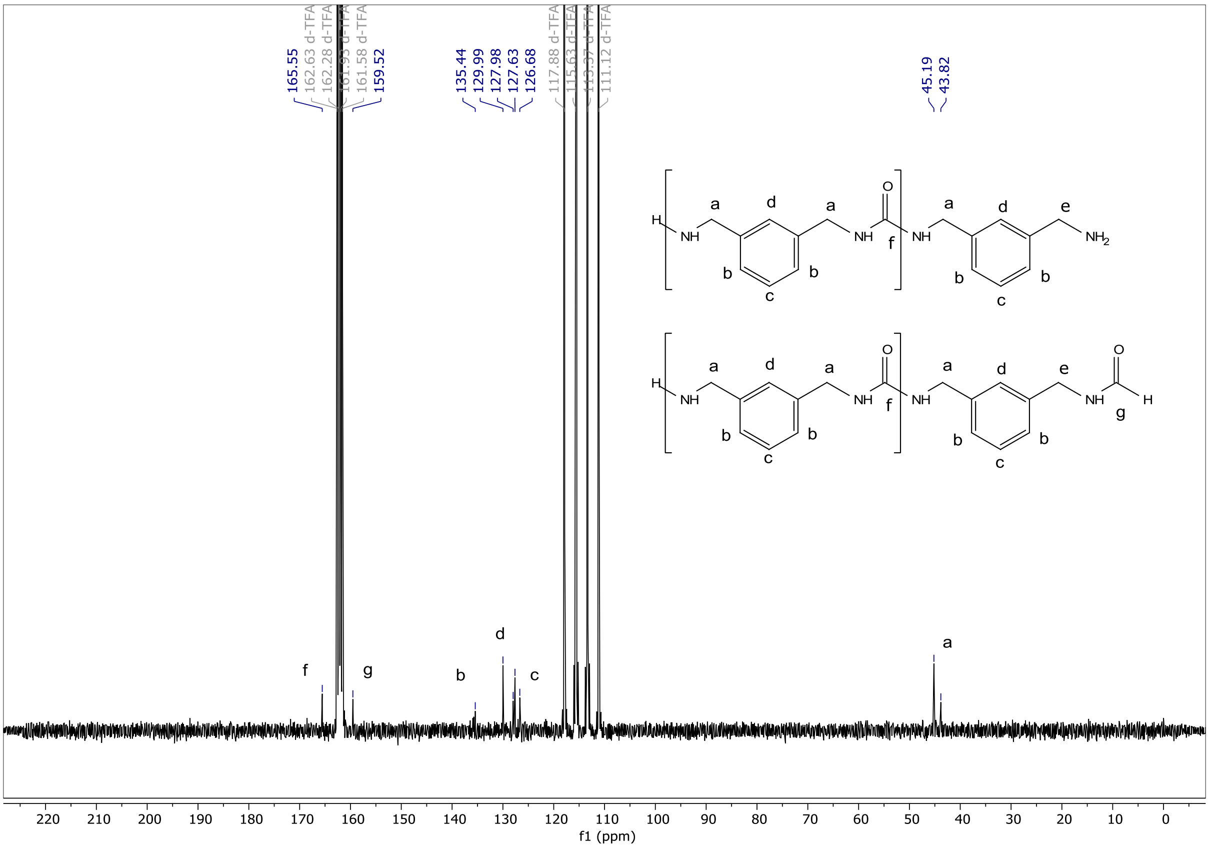

Figure S78: $^{13}C\{^{1}H\}$ NMR spectrum (*d*-TFA, 126 MHz, 298 K) of Table 3, entry 5.

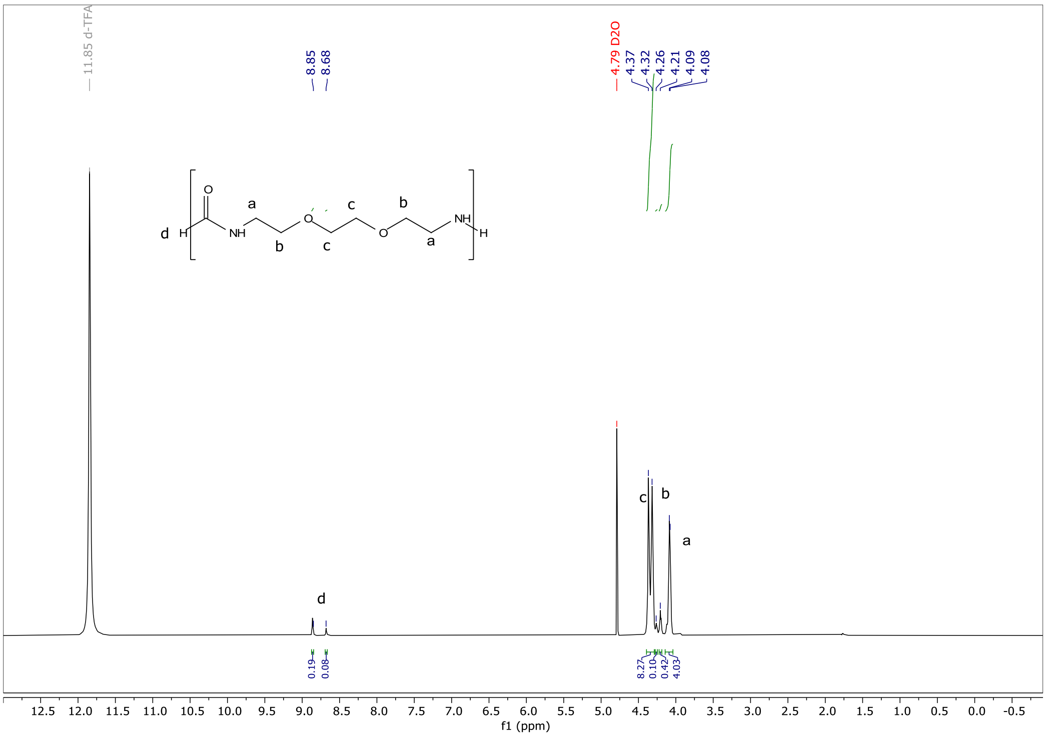

Figure S79: ^{1}H NMR spectrum (*d*-TFA, 500 MHz, 298 K) of Table 3, entry 6.

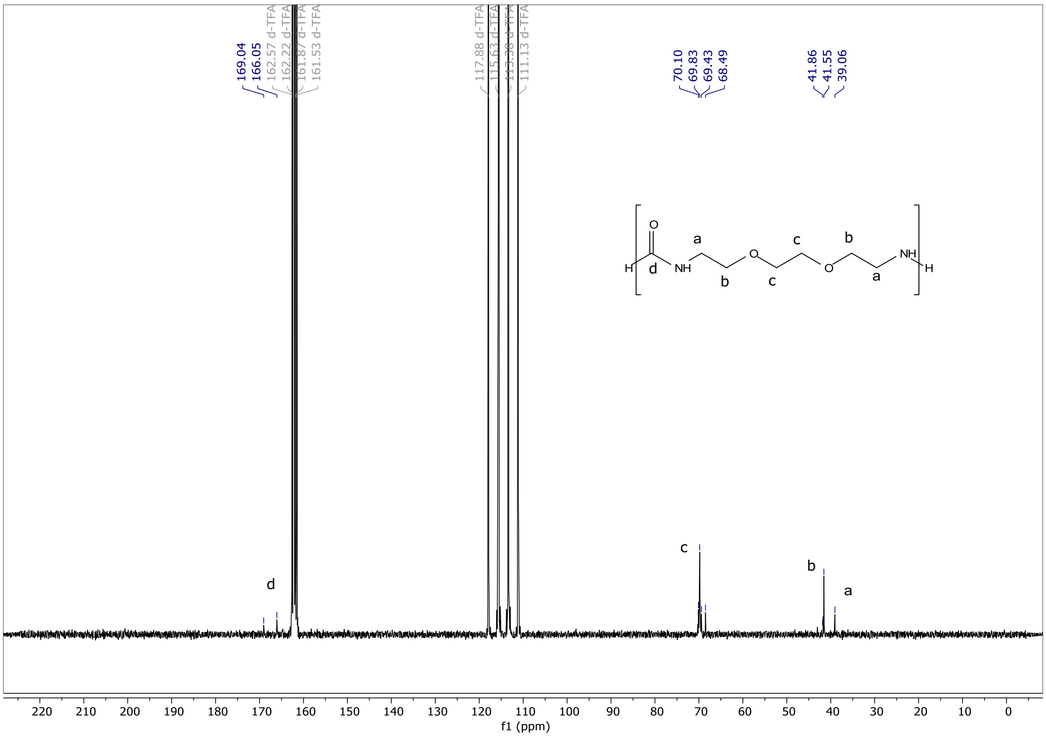

Figure S80: $^{13}C\{^{1}H\}$ NMR spectrum (*d*-TFA, 126 MHz, 298K) of Table 3, entry 6.

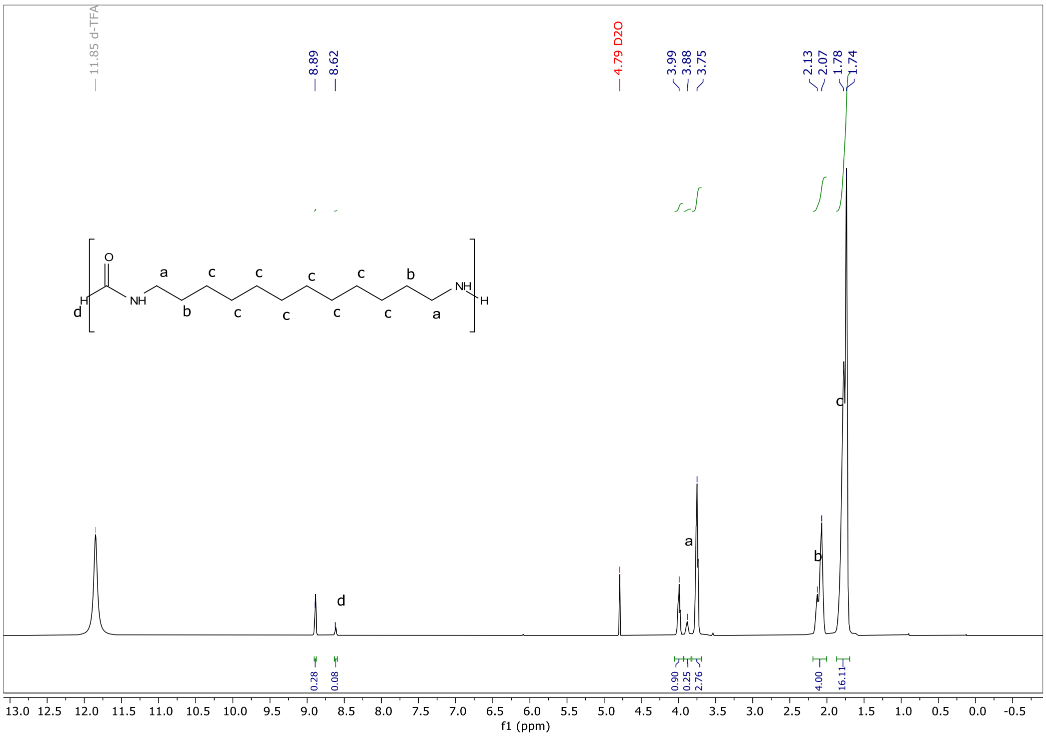

Figure S81: ^{1}H NMR spectrum (*d*-TFA, 500 MHz, 298K) of Table 3, entry 7.

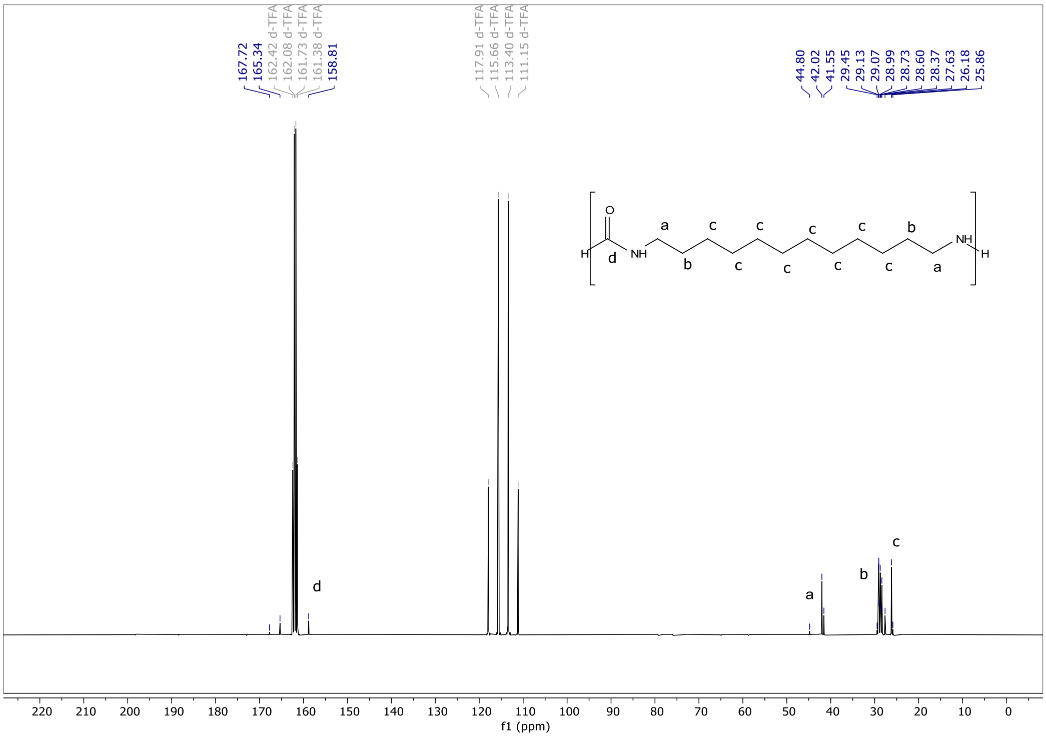

Figure S82: $^{13}C\{^1H\}$ NMR spectrum (*d*-TFA, 126 MHz, 298K) of Table 3, entry 7.

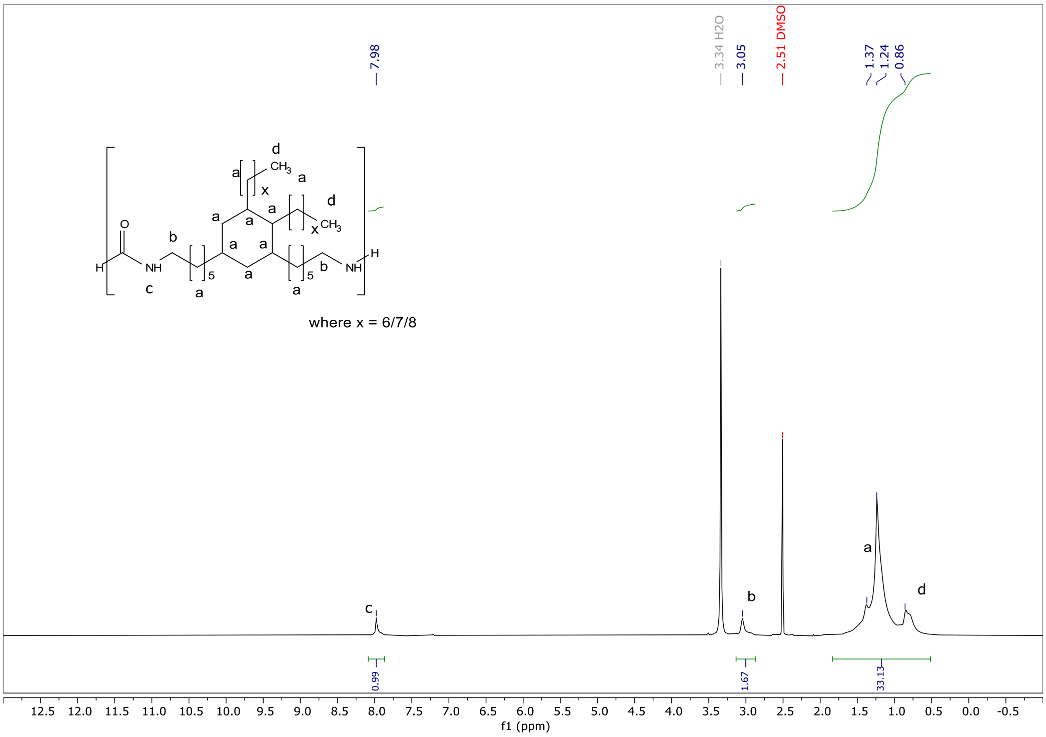

Figure S83: ^1H NMR spectrum (*d*-DMSO, 500 MHz, 298K) of Table 3, entry 8.

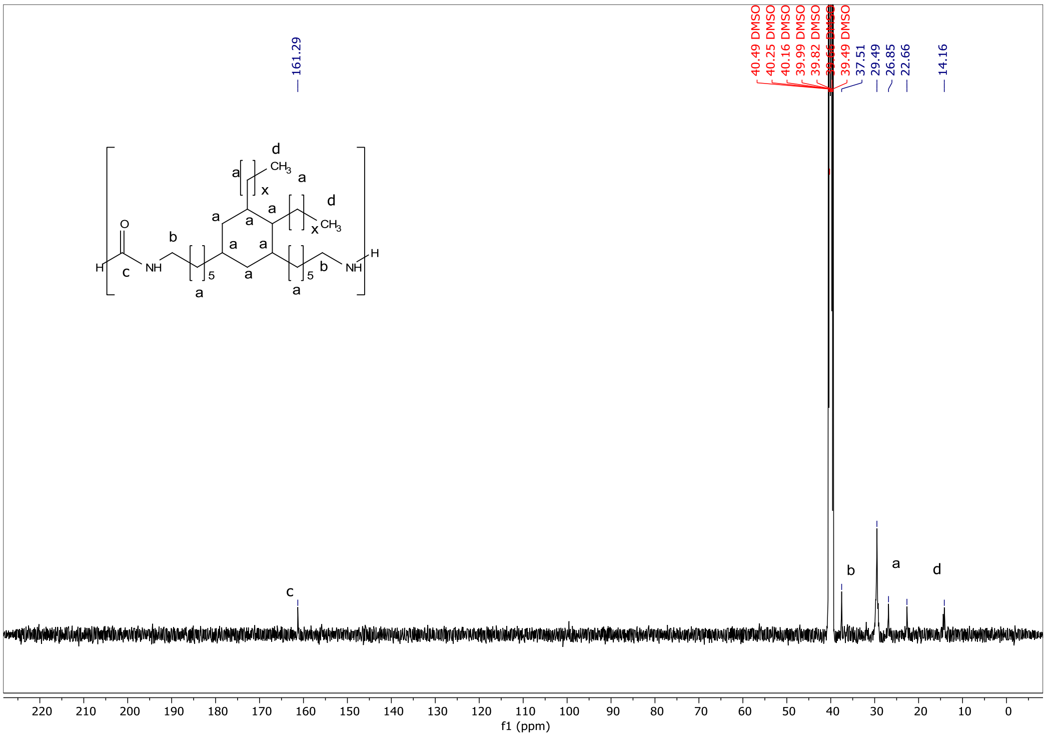

Figure S84: $^{13}C\{^1H\}$ NMR spectrum (*d*-DMSO, 126 MHz, 298K) of Table 3, entry 8.

3.3.3. IR spectra for polyurea synthesis

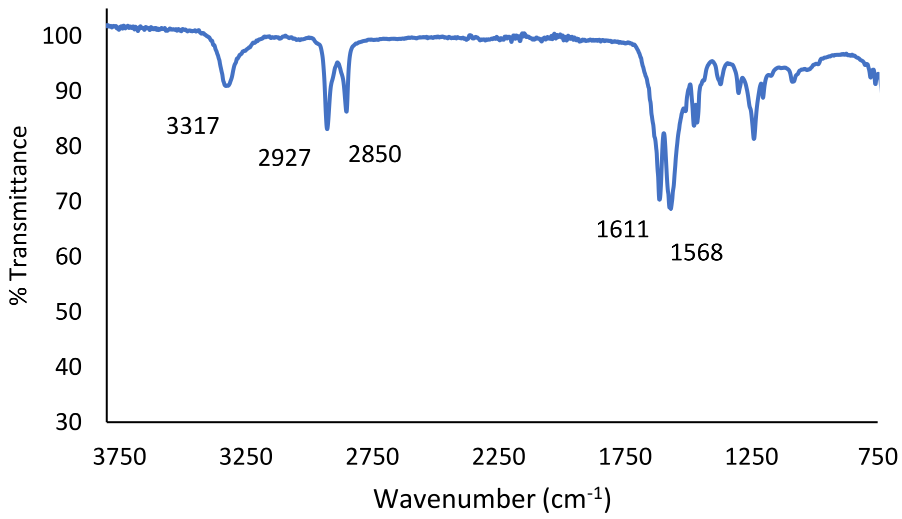

Figure S85: IR spectrum of the polyurea corresponding to Table 3, entry 1.

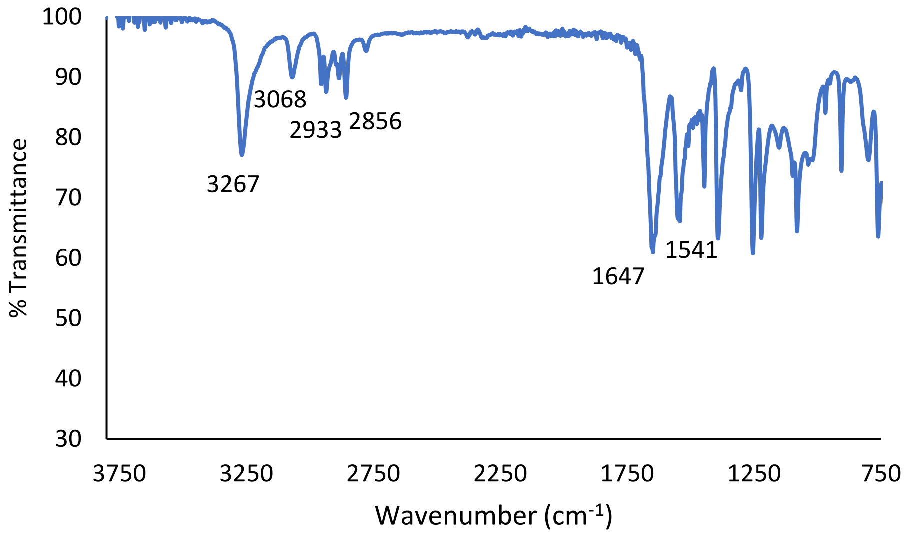

Figure S86: IR spectrum of the polyurea corresponding to Table 3, entry 2.

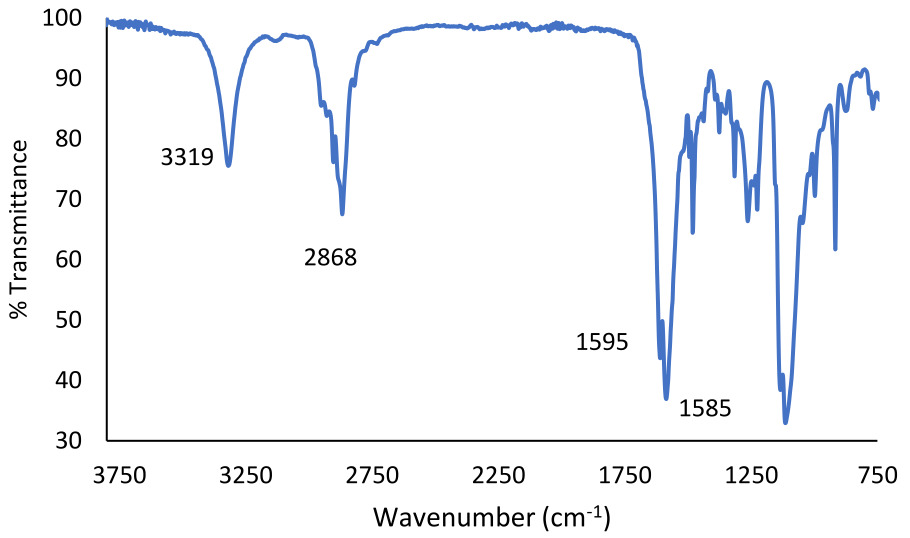

Figure S87: IR spectrum of the polyurea corresponding to Table 3, entry 3.

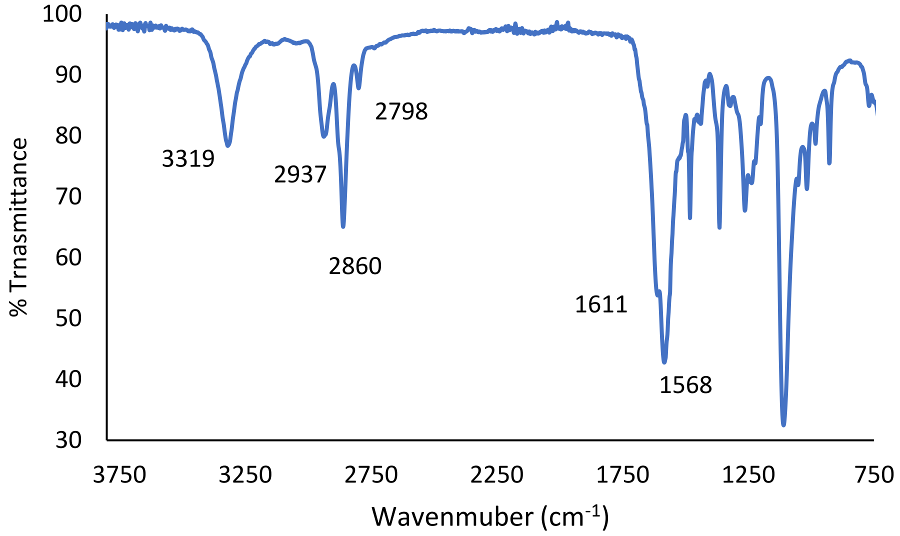

Figure S88: IR spectrum of the polyurea corresponding to Table 3, entry 4.

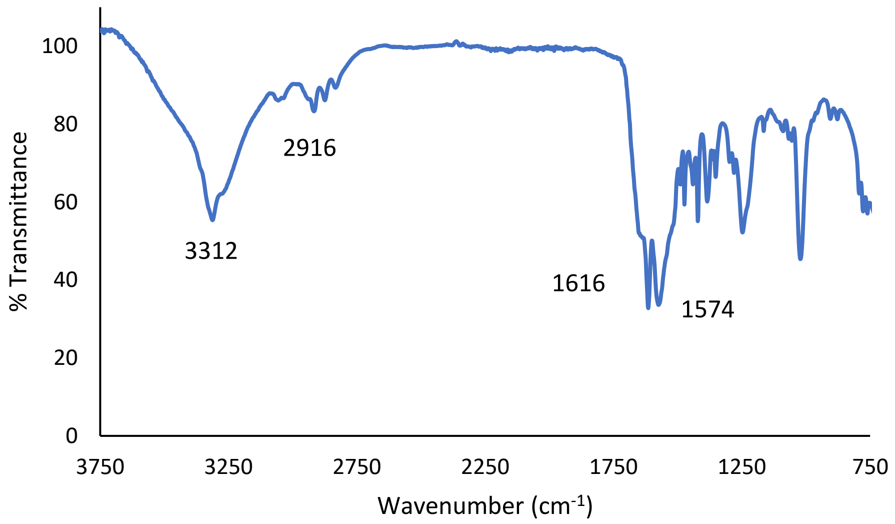

Figure S89: IR spectrum of the polyurea corresponding to Table 3, entry 5.

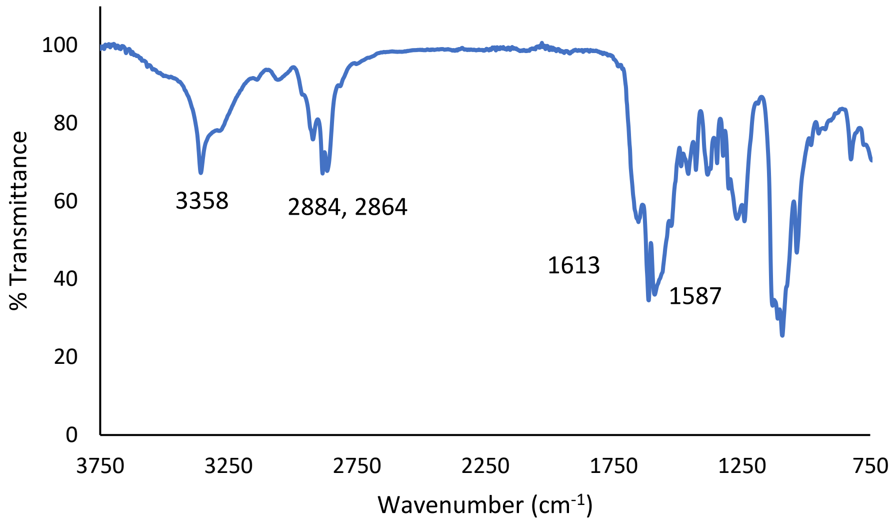

Figure S90: IR spectrum of the polyurea corresponding to Table 3, entry 6.

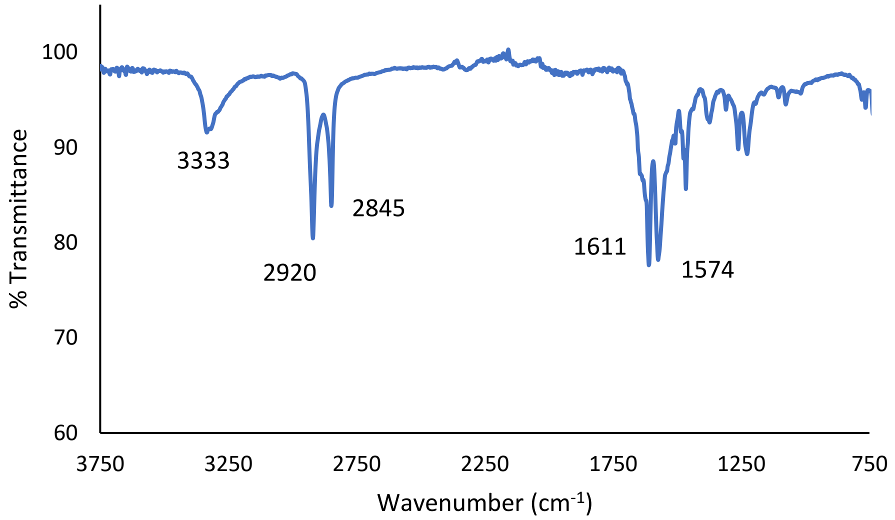

Figure S91: IR spectrum of the polyurea corresponding to Table 3, entry 7.

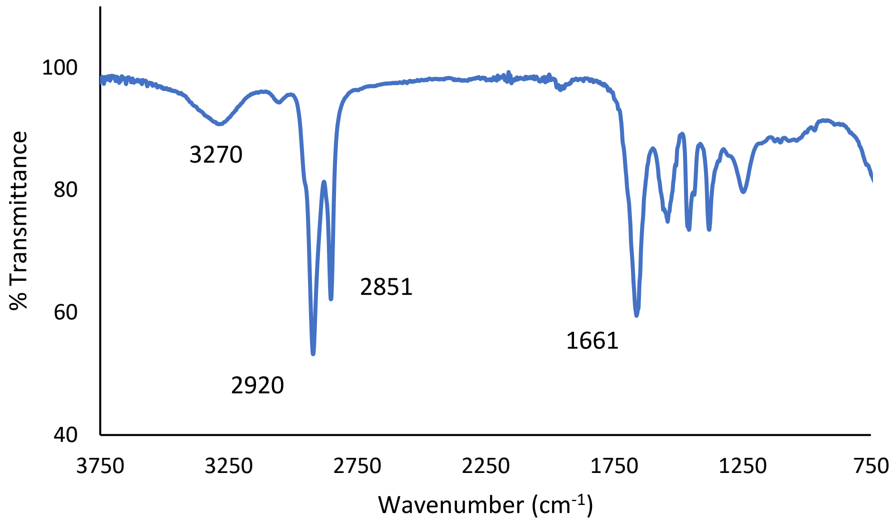

Figure S92: IR spectrum of the polyurea corresponding to Table 3, entry 8.

3.3.4. DSC spectra for polyurea samples

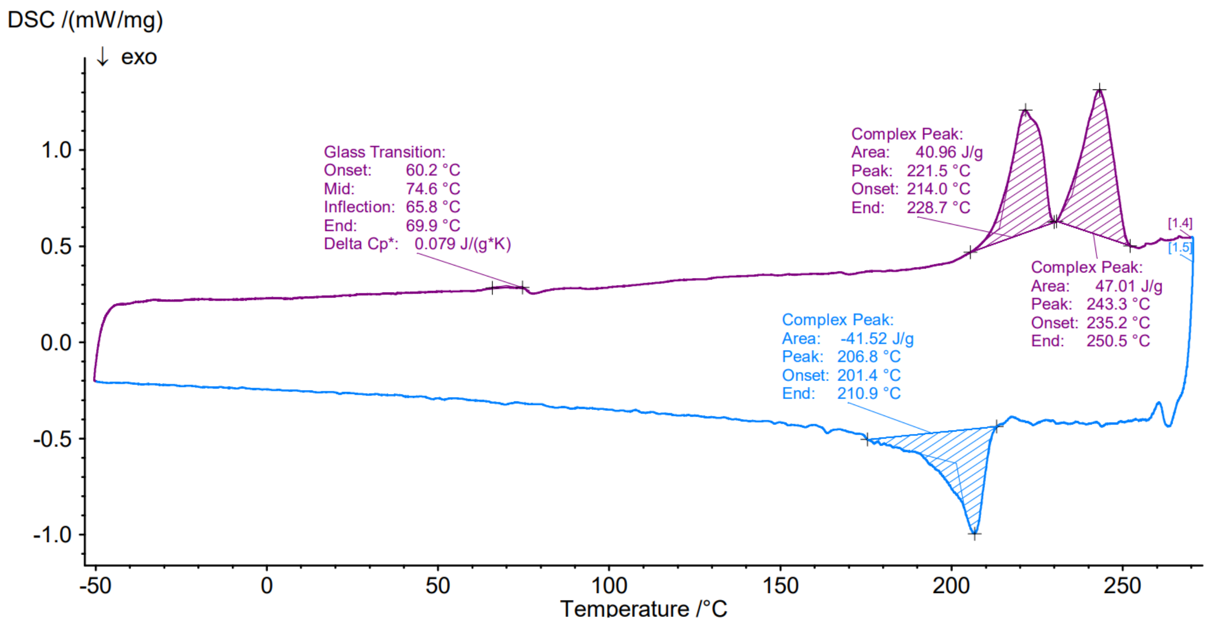

Figure S93: DSC trace corresponding to Table 3, entry 1.

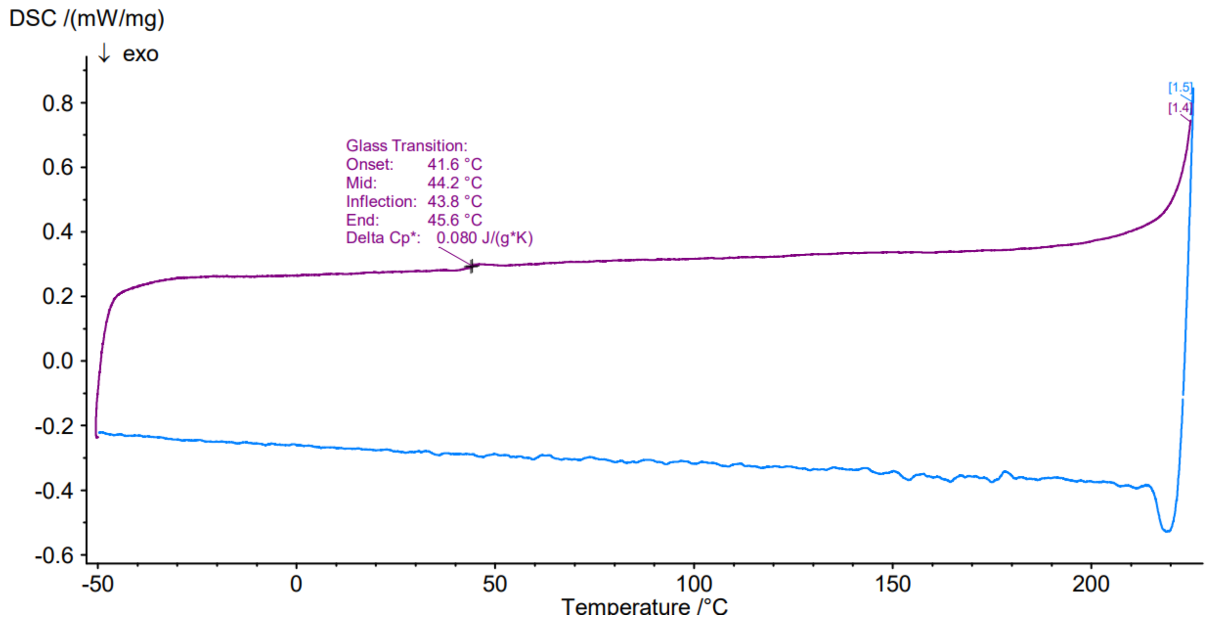

Figure S94: DSC trace corresponding to Table 3, entry 2.

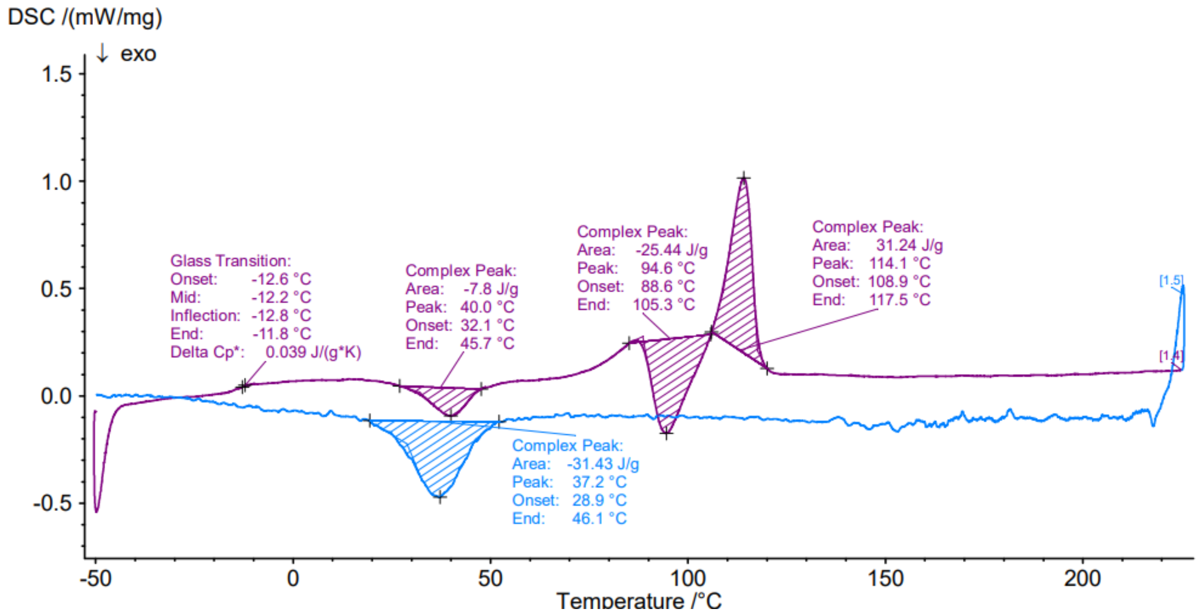

Figure S95: DSC trace corresponding to Table 3, entry 3.

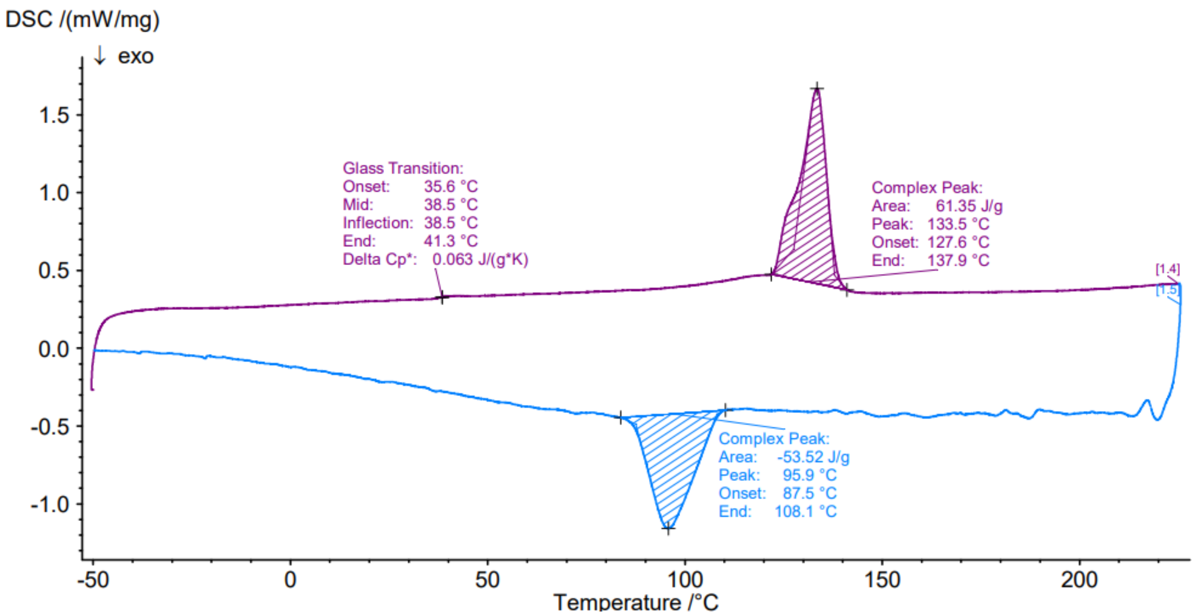

Figure S96: DSC trace corresponding to Table 3, entry 4.

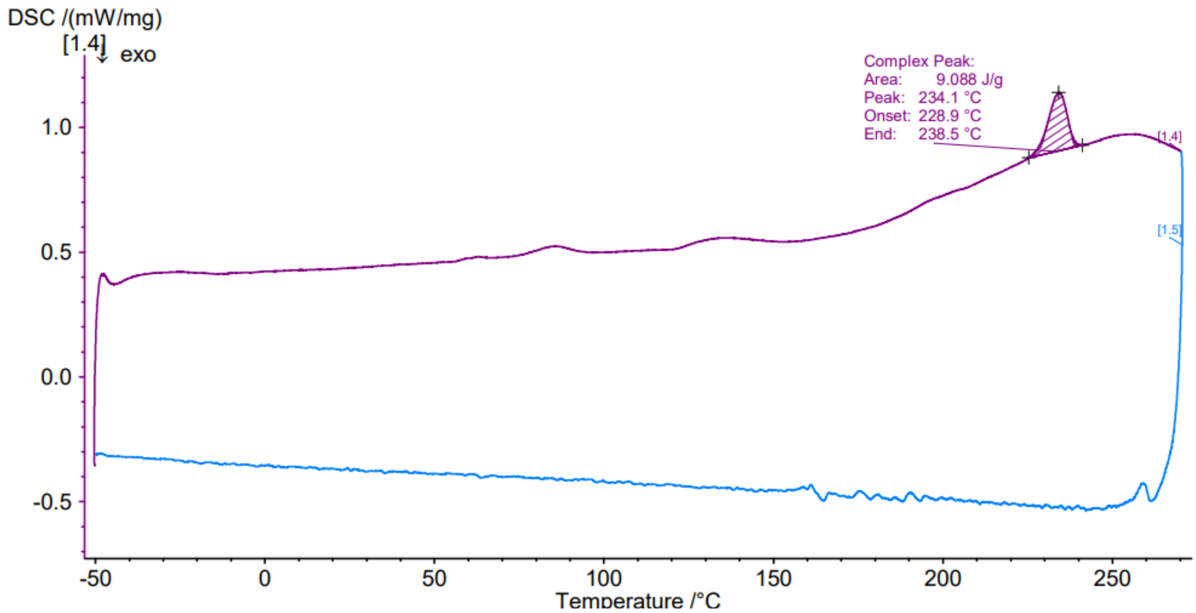

Figure S97: DSC trace corresponding to Table 3, entry 5.

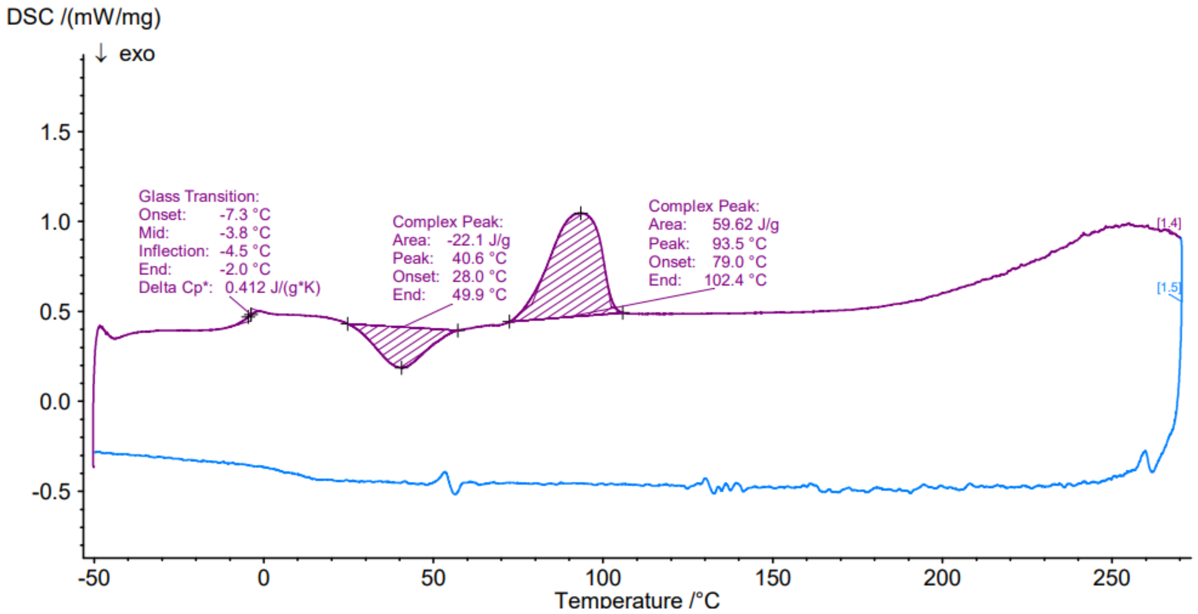

Figure S98: DSC trace corresponding to Table 3, entry 6.

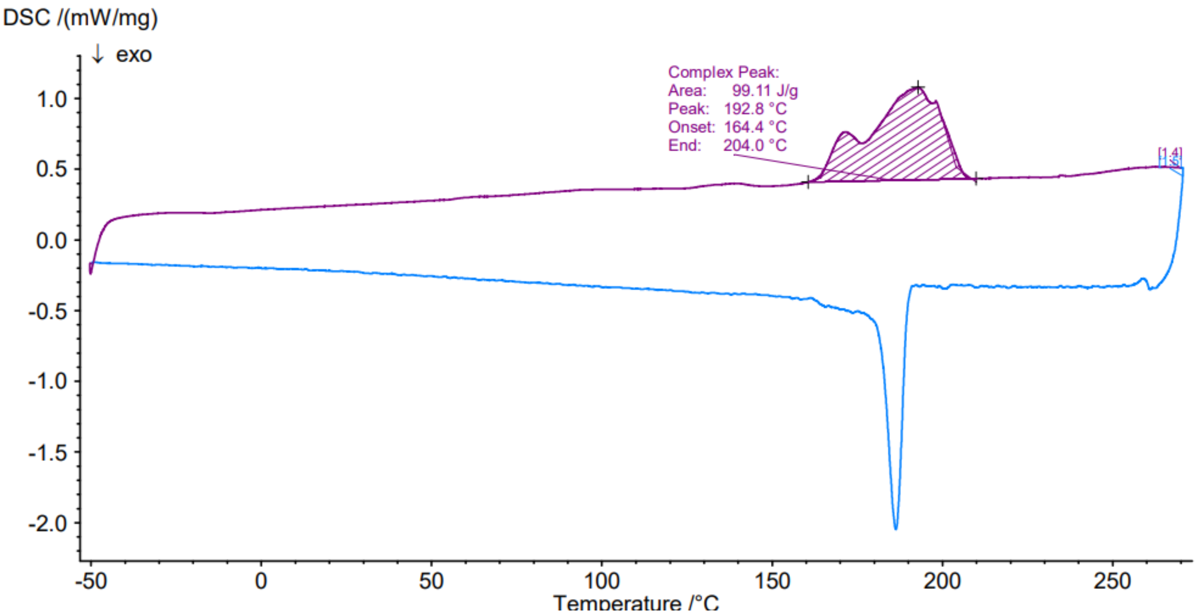

Figure S99: DSC trace corresponding to Table 3, entry 7.

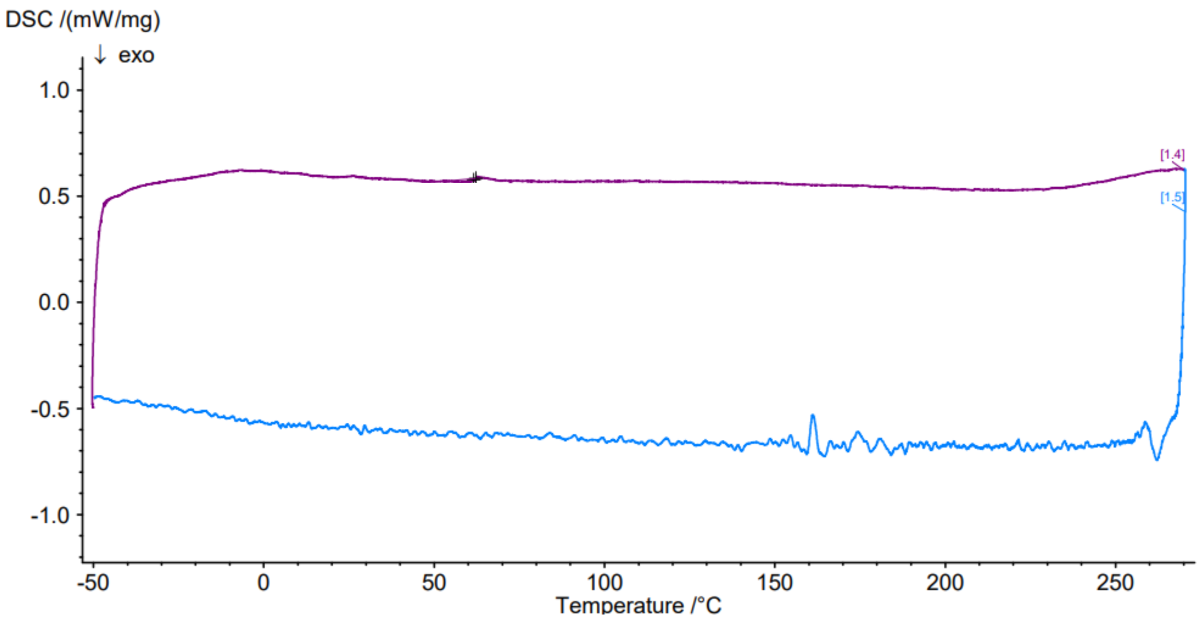

Figure S100: DSC trace corresponding to Table 3, entry 8.

3.3.5. TGA spectra for polyurea samples

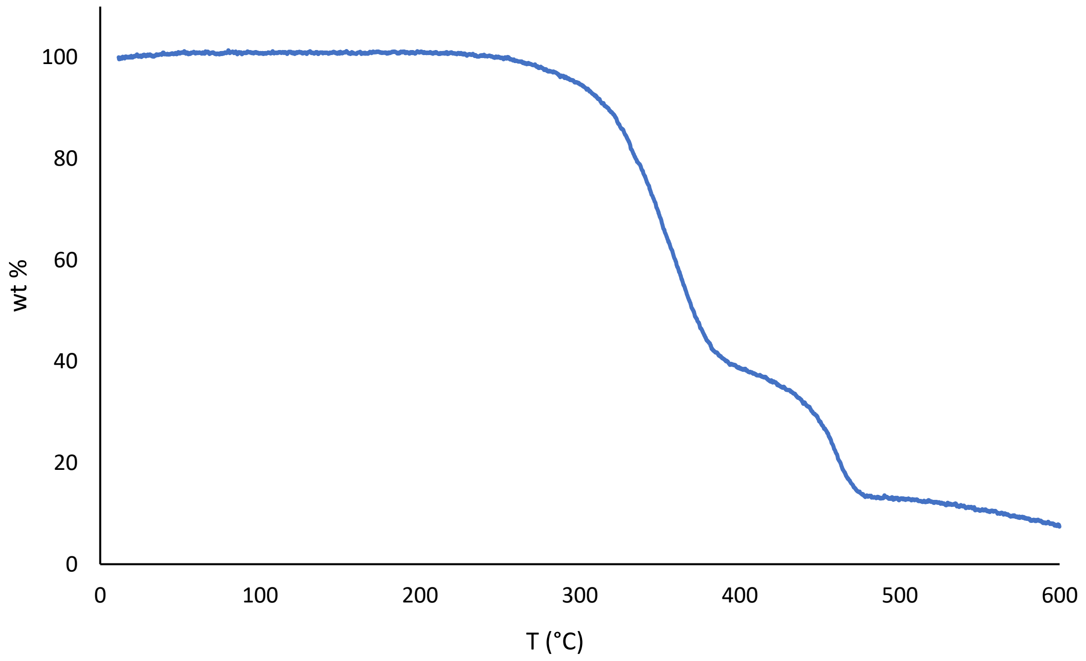

Figure S101: TGA data plotting mass loss as a function of data corresponding to Table 3, entry 1.

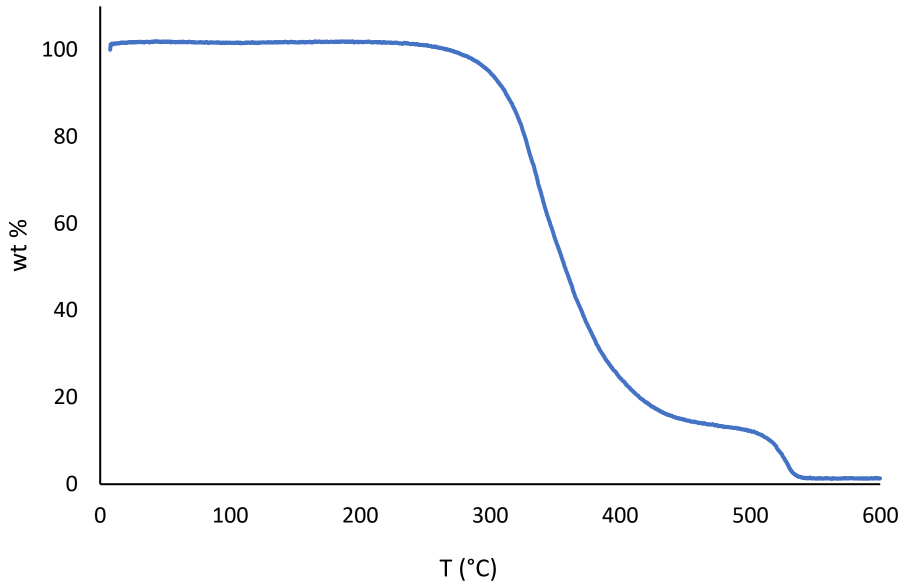

Figure S102: TGA data plotting mass loss as a function of data corresponding to Table 3, entry 2.

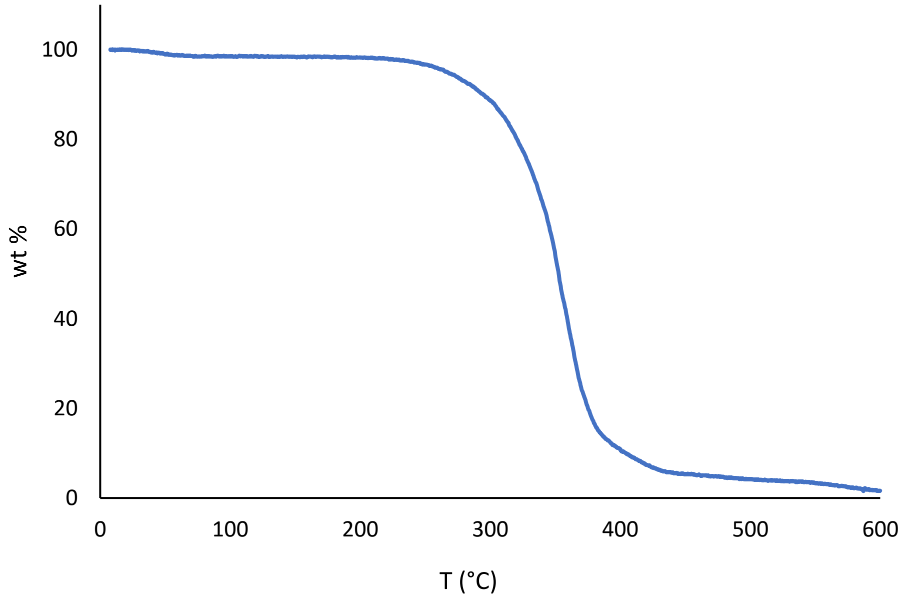

Figure S103: TGA data plotting mass loss as a function of data corresponding to Table 3, entry 3.

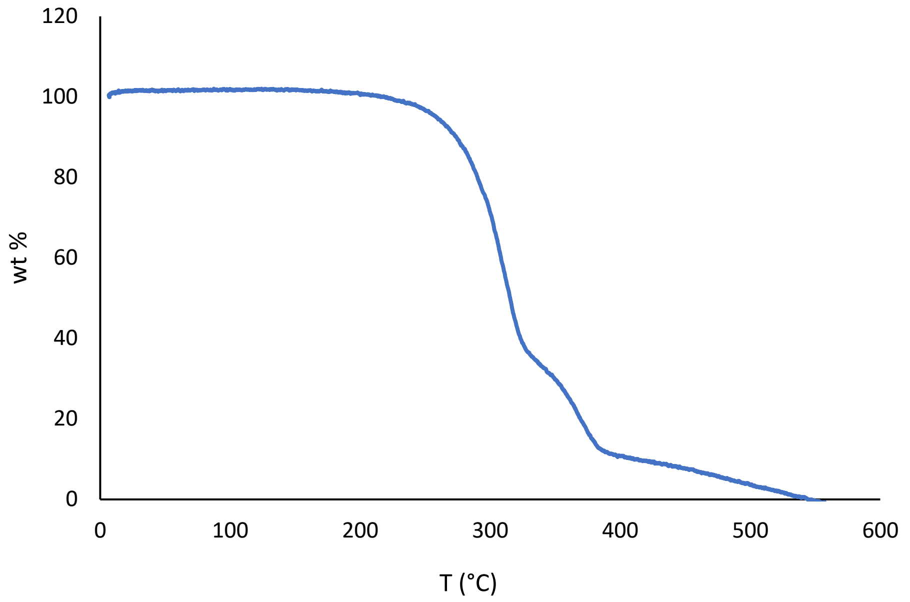

Figure S104: TGA data plotting mass loss as a function of data corresponding to Table 3, entry 4.

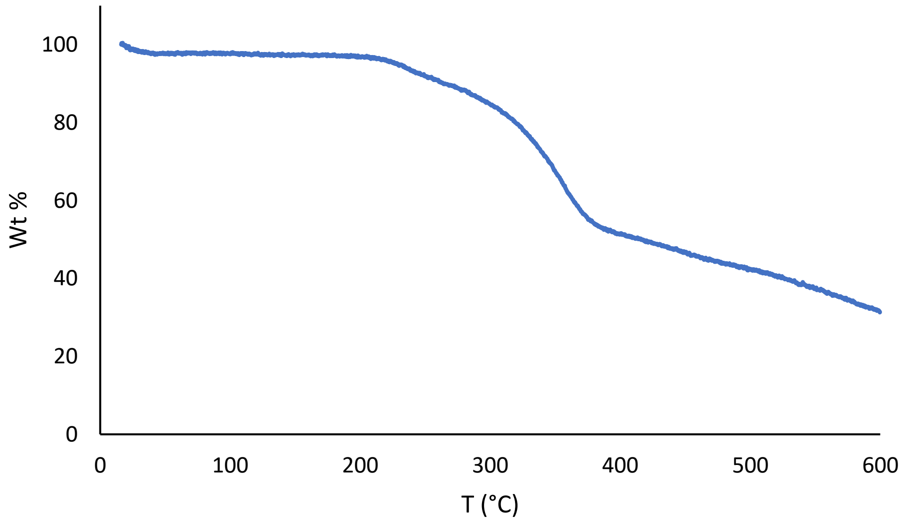

Figure S105: TGA data plotting mass loss as a function of data corresponding to Table 3, entry 5.

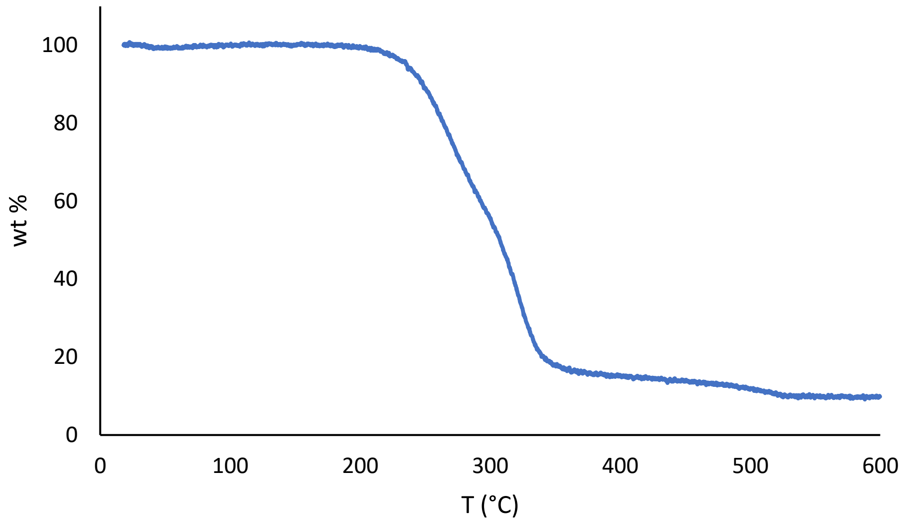

Figure S106: TGA data plotting mass loss as a function of data corresponding to Table 3, entry 6.

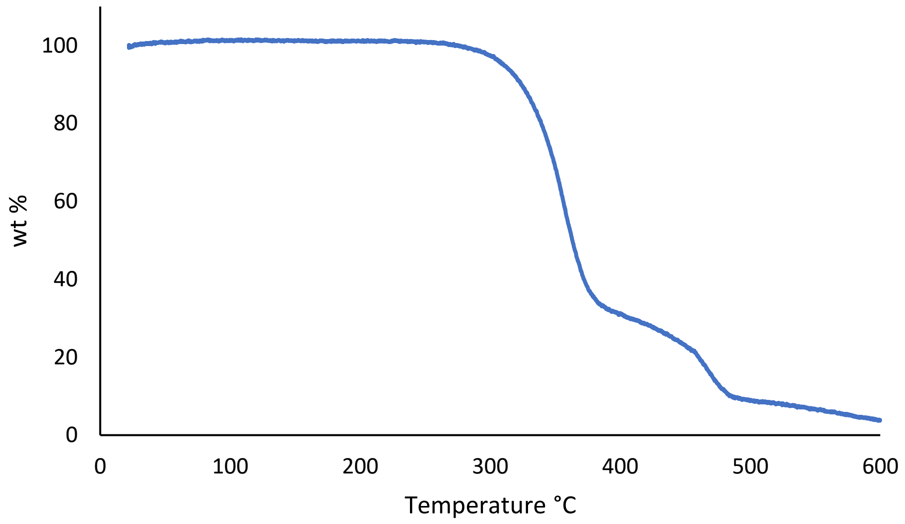

Figure S107: TGA data plotting mass loss as a function of data corresponding to Table 3, entry 7.

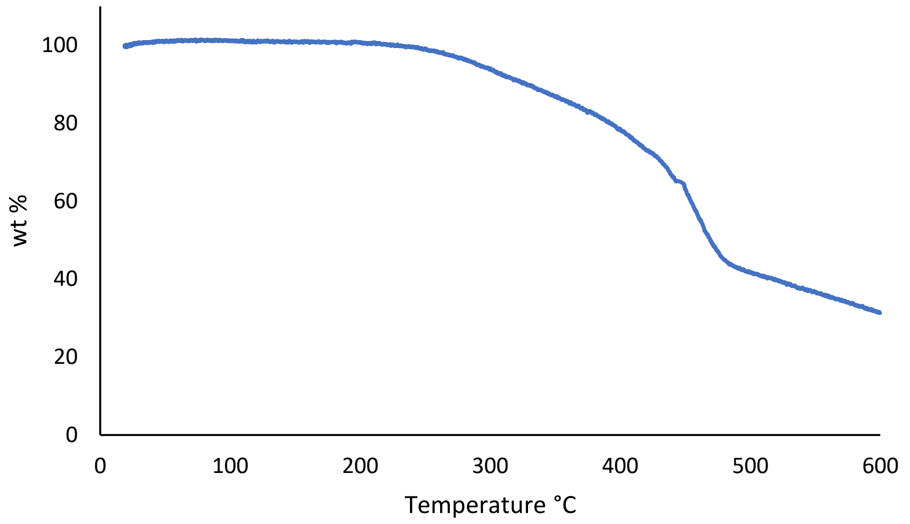

Figure S108: TGA data plotting mass loss as a function of data corresponding to Table 3, entry 8.

3.3.6. MALDI TOF spectra for polyurea samples

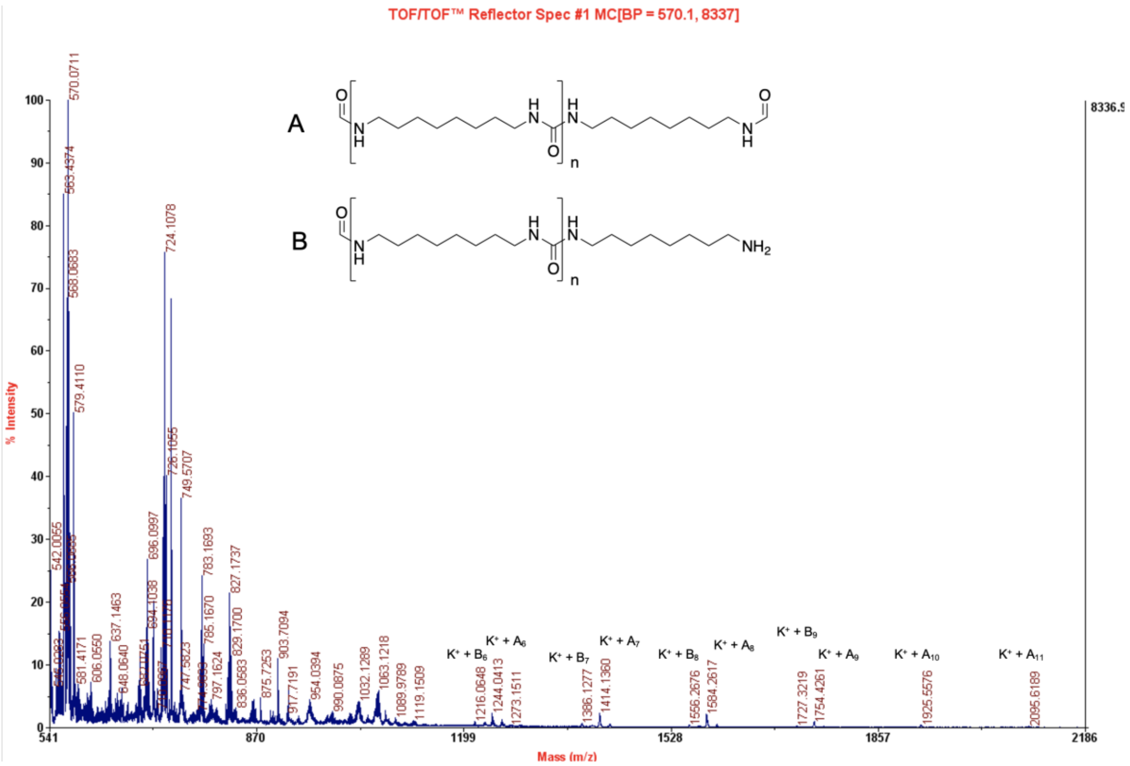

Figure S109: MALDI-TOF mass spectrum of polyurea corresponding to Table 3, entry 1, where the mass assignment is given in the form X_y, where “X” denotes polymer structure “A” or “B” and “y” denotes the value of repeating units “n”.

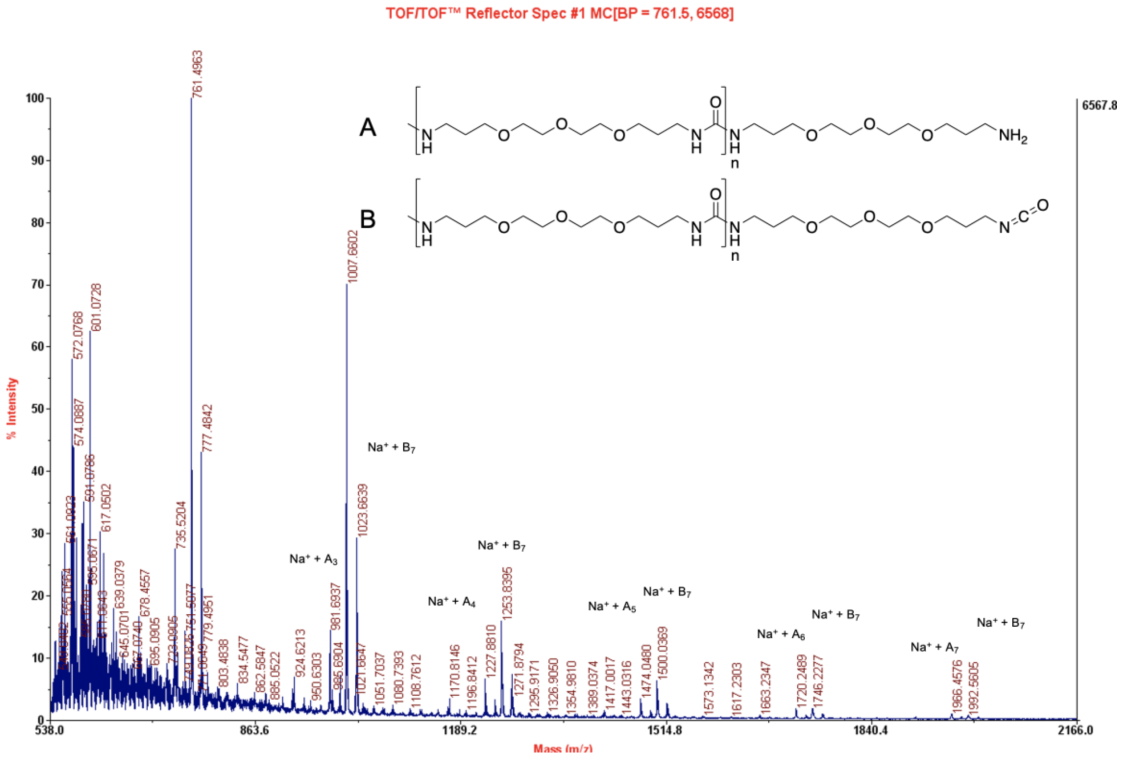

Figure S110: MALDI-TOF mass spectrum of polyurea corresponding to Table 3, entry 2, where the mass assignment is given in the form X_y, where “X” denotes polymer structure “A” or “B” and “y” denotes the value of repeating units “n”.

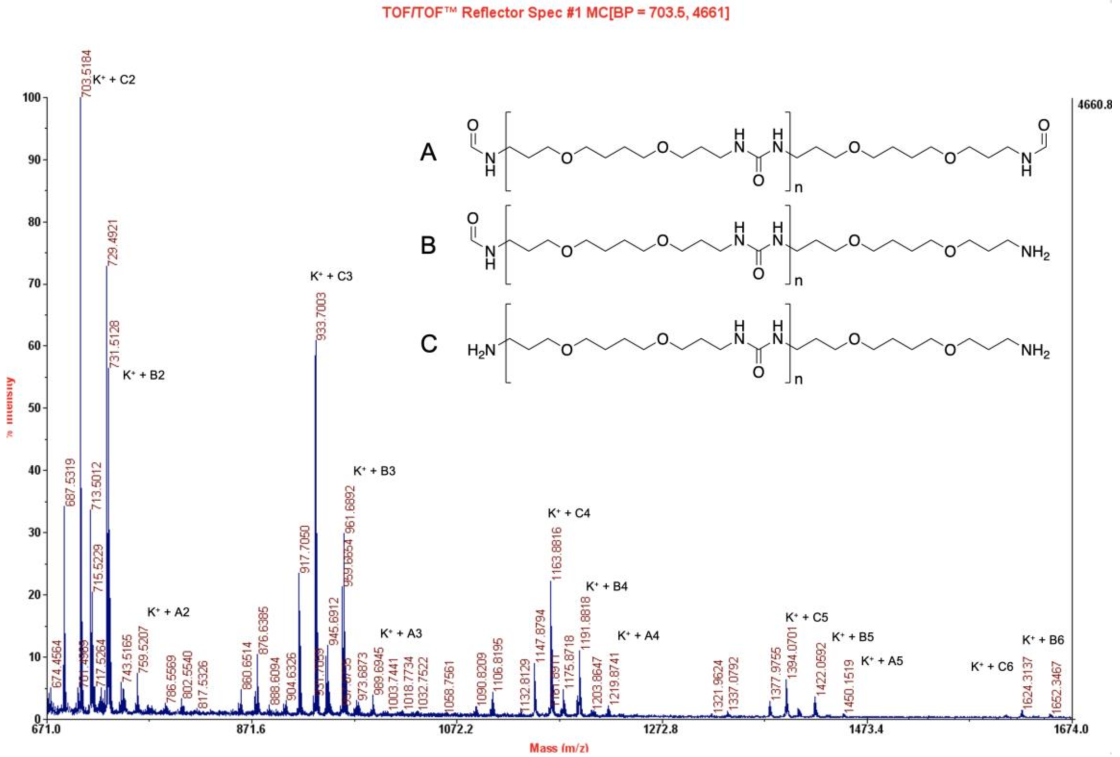

Figure S111: MALDI-TOF mass spectrum of polyurea corresponding to Table 3, entry 3, where the mass assignment is given in the form X_y, where “X” denotes polymer structure “A”, “B” or “C” and “y” denotes the value of repeating units “n”.

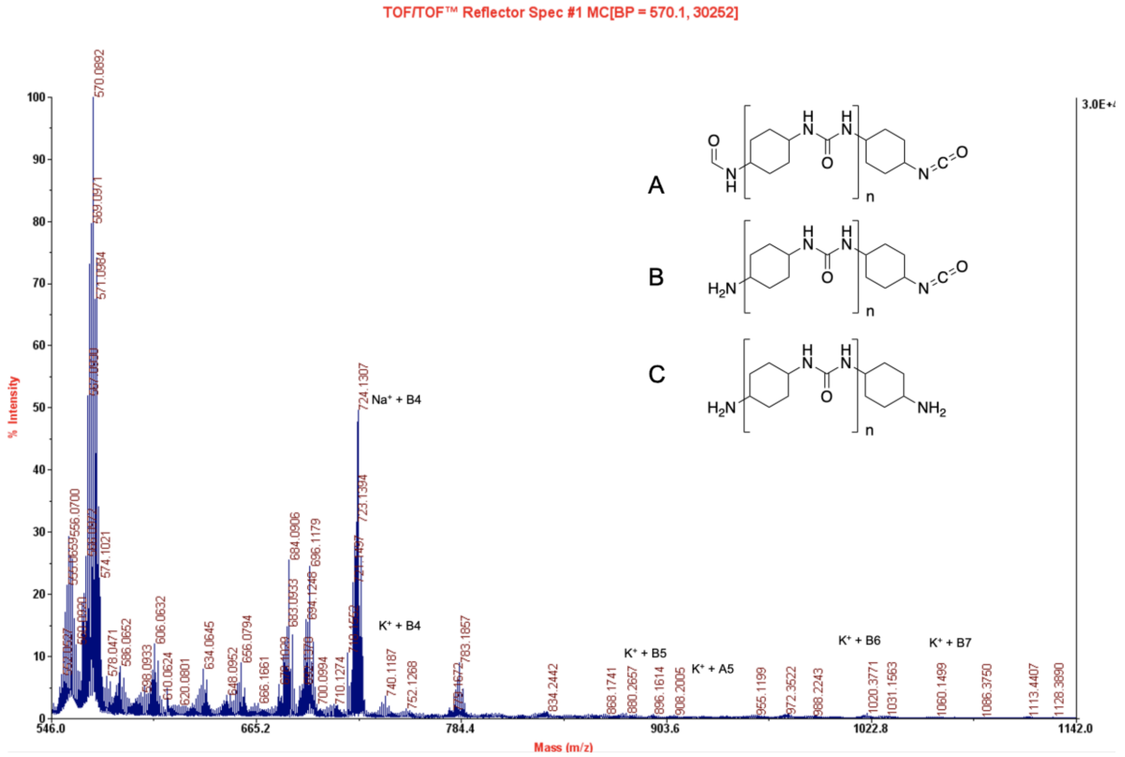

Figure S112: MALDI-TOF mass spectrum of polyurea corresponding to Table 3, entry 4, where the mass assignment is given in the form X_y, where “X” denotes polymer structure “A” or “B” and “y” denotes the value of repeating units “n”.

3.3.7. GPC data of the isolated polyurea

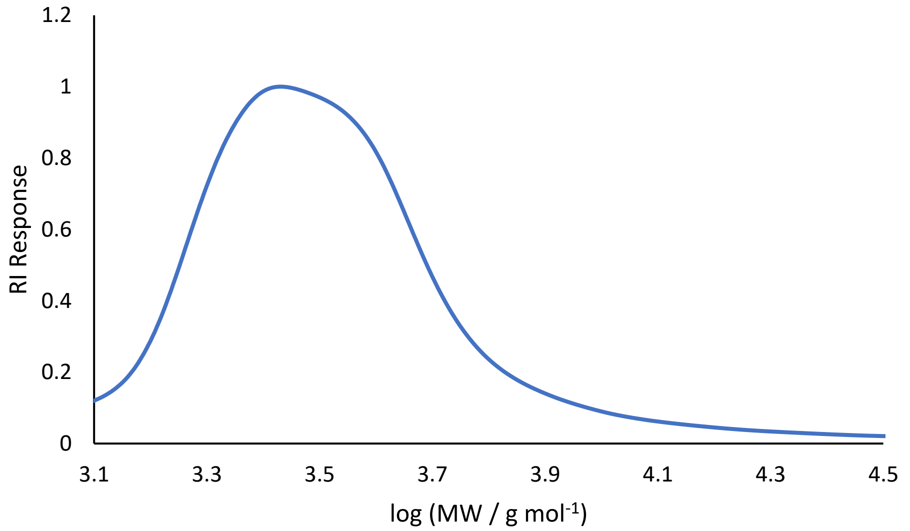

Figure S113: GPC chromatograph of polyurea corresponding to Table 3, entry 1.

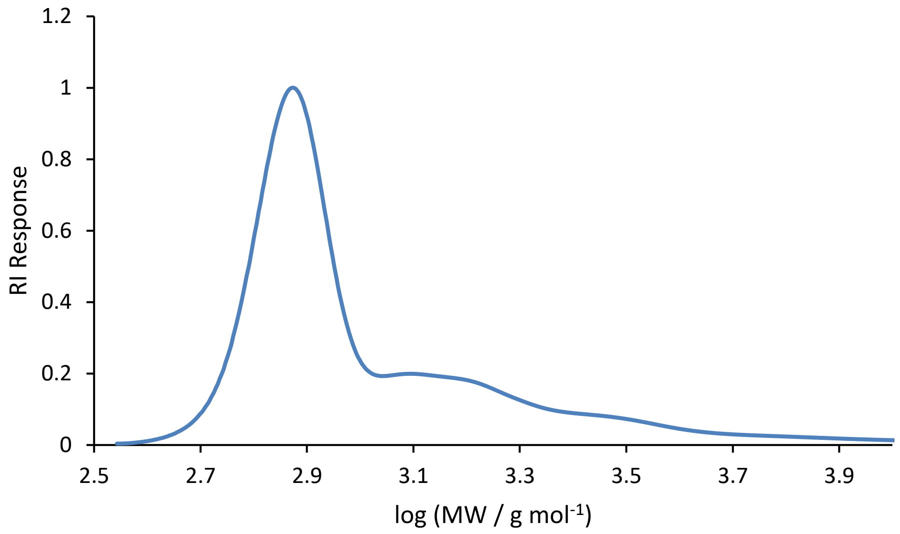

Figure S114: GPC chromatograph of polyurea corresponding to Table 3, entry 2.

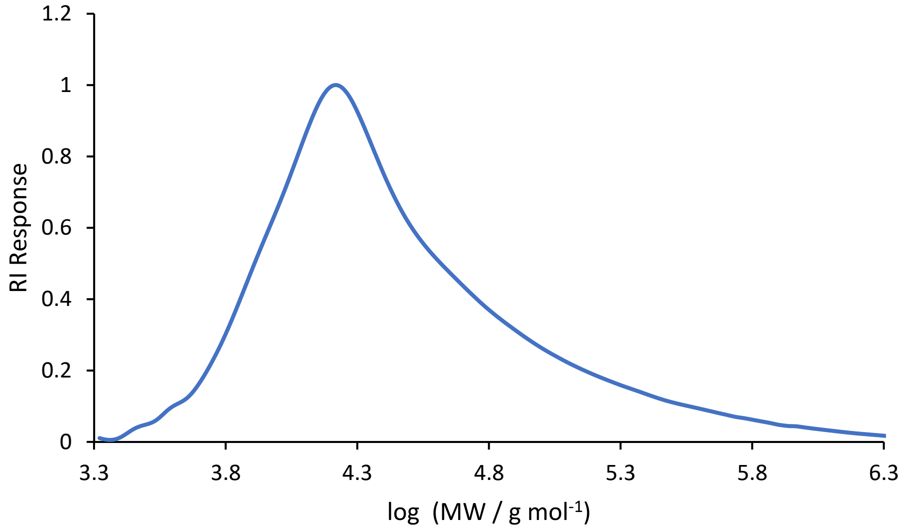

Figure S115: GPC chromatograph of polyurea corresponding to Table 3, entry 3.

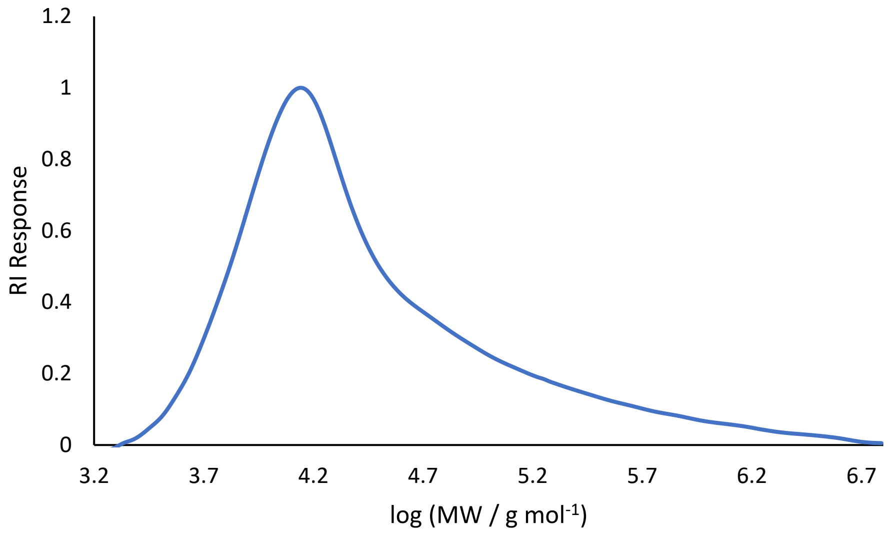

Figure S116: GPC chromatograph of polyurea corresponding to Table 3, entry 4.

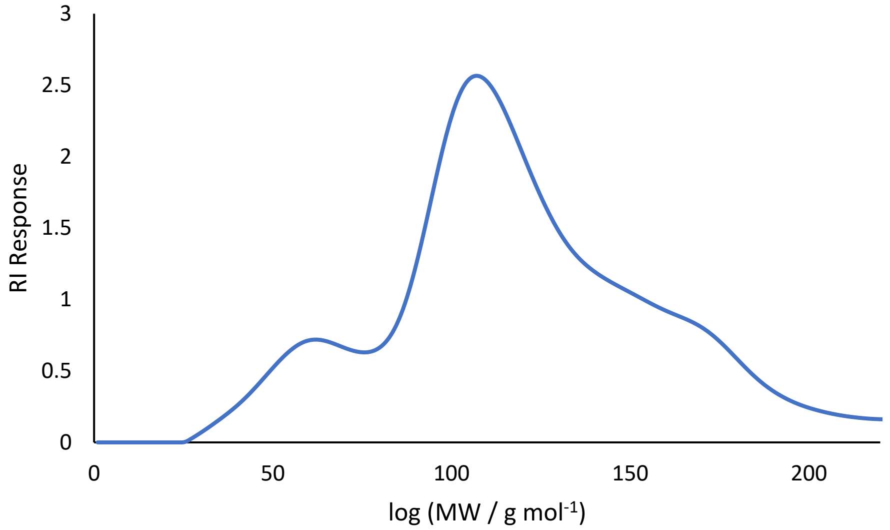

Figure S117: GPC chromatograph of polyurea corresponding to Table 3, entry 8.

3.4. Characterisation data for polyurea(urethane) synthesis

3.4.1. ^{1}H NMR and ^{13}C{^{1}H} NMR spectra for polyurea(urethane) synthesis

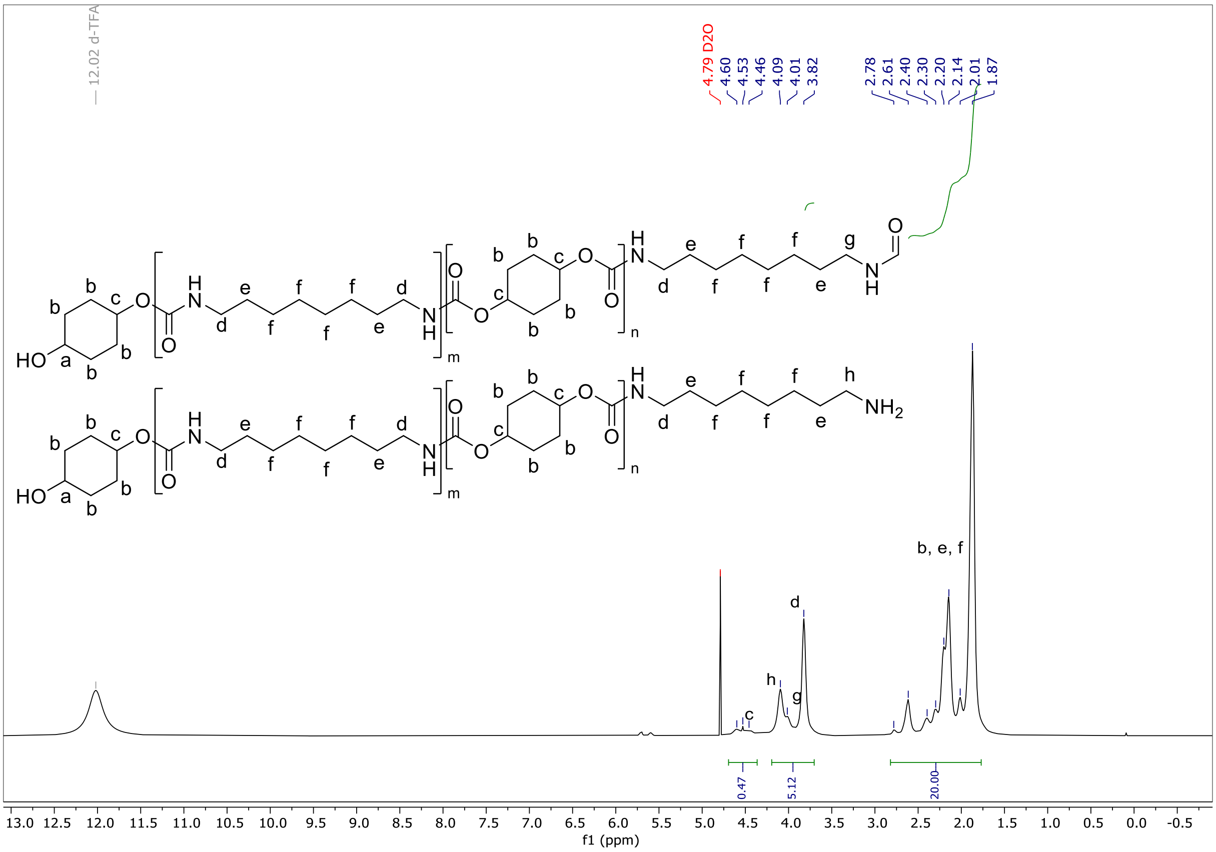

Figure S118: ^{1}H NMR spectrum (*d*-TFA, 500 MHz, 298 K) of Table 4, entry 1.

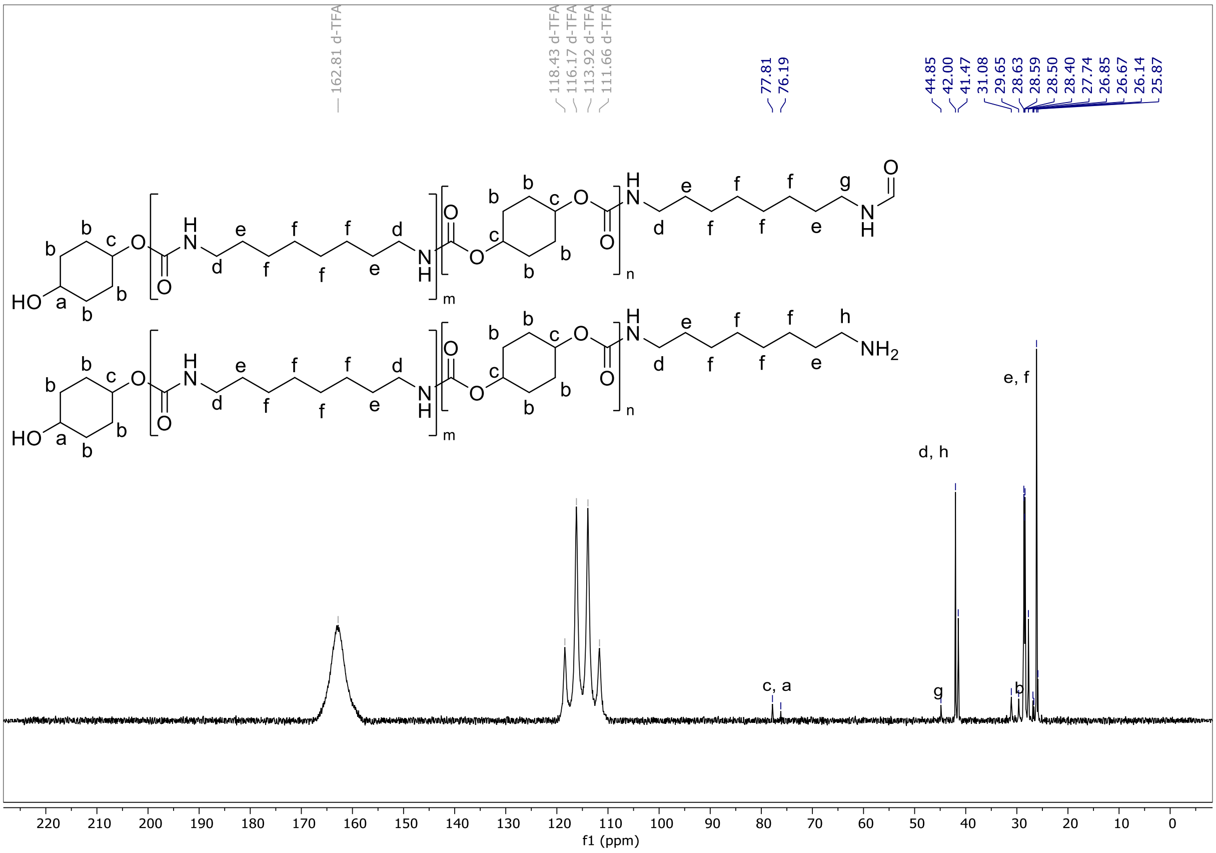

Figure S119: ^{13}C{^{1}H} NMR spectrum (*d*-TFA, 126 MHz, 298 K) of the Table 4, entry 1.

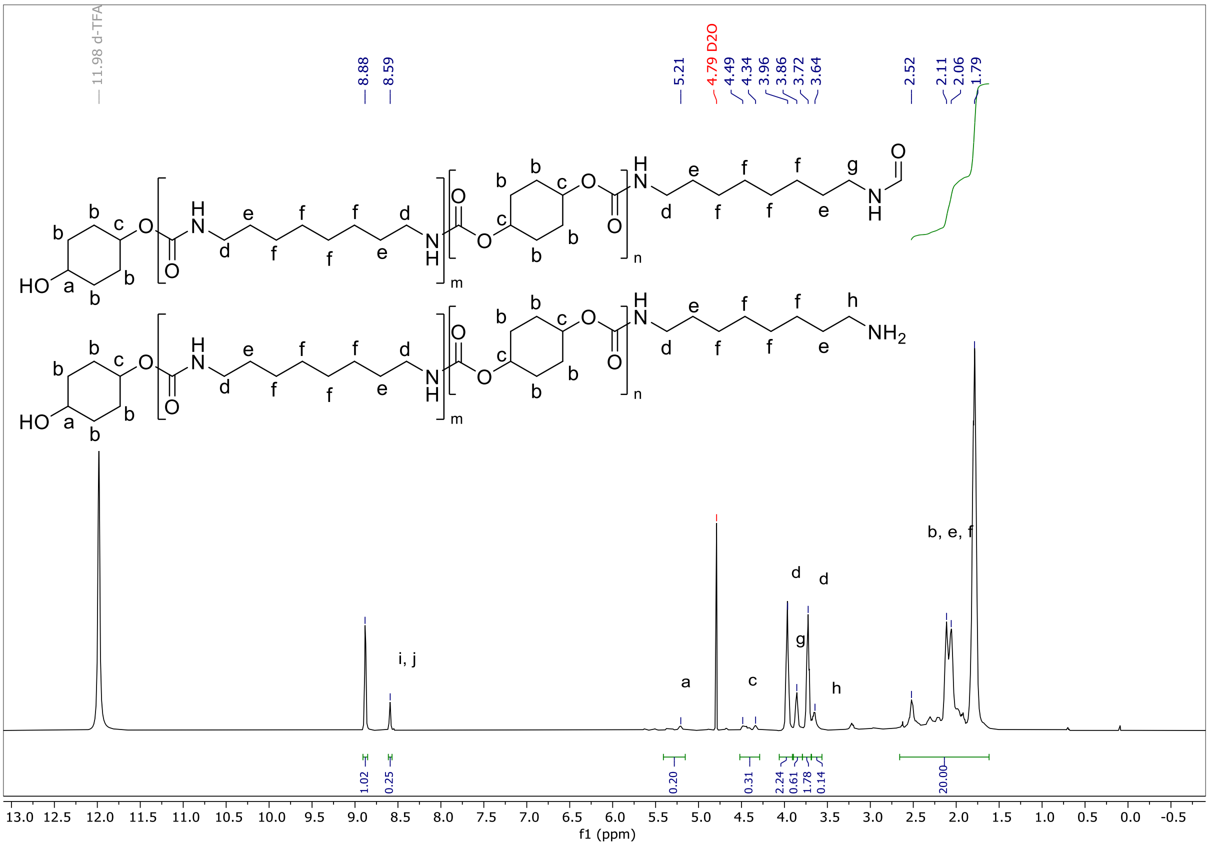

Figure S120: ^{1}H NMR spectrum (*d*-TFA, 500 MHz, 298 K) of Table 4, entry 2.

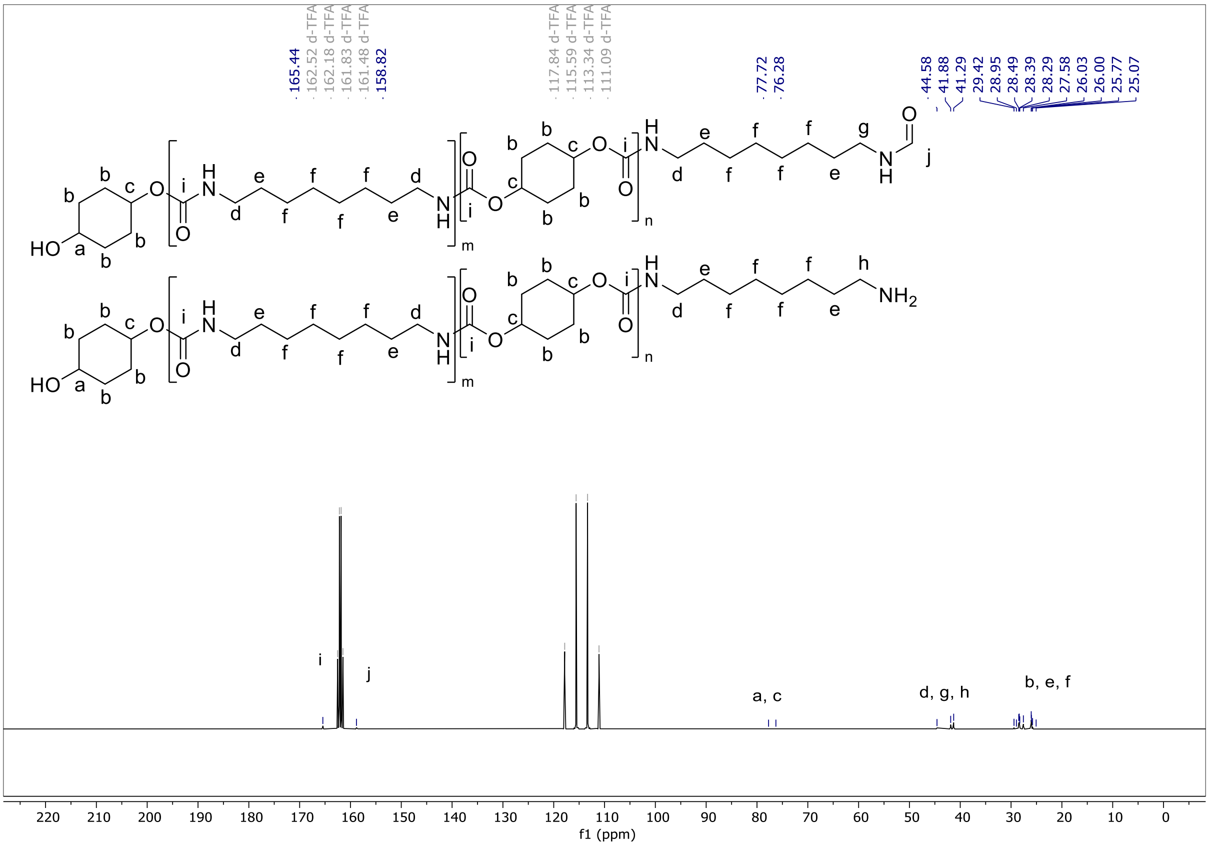

Figure S121: ^{13}C{^{1}H} NMR spectrum (*d*-TFA, 126 MHz, 298 K) of Table 4, entry 2.

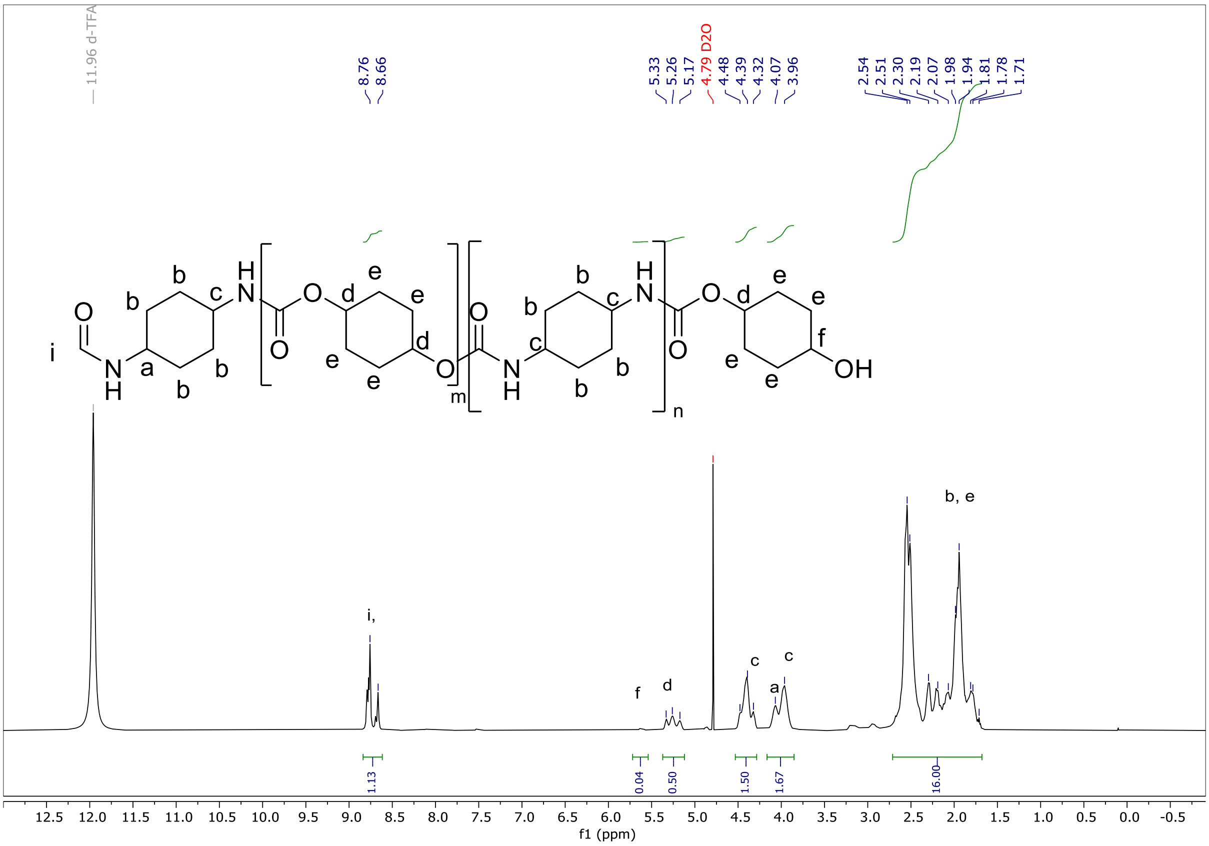

Figure S122: ^{1}H NMR spectrum (d-TFA, 500 MHz, 298 K) of Table 4, entry 3.

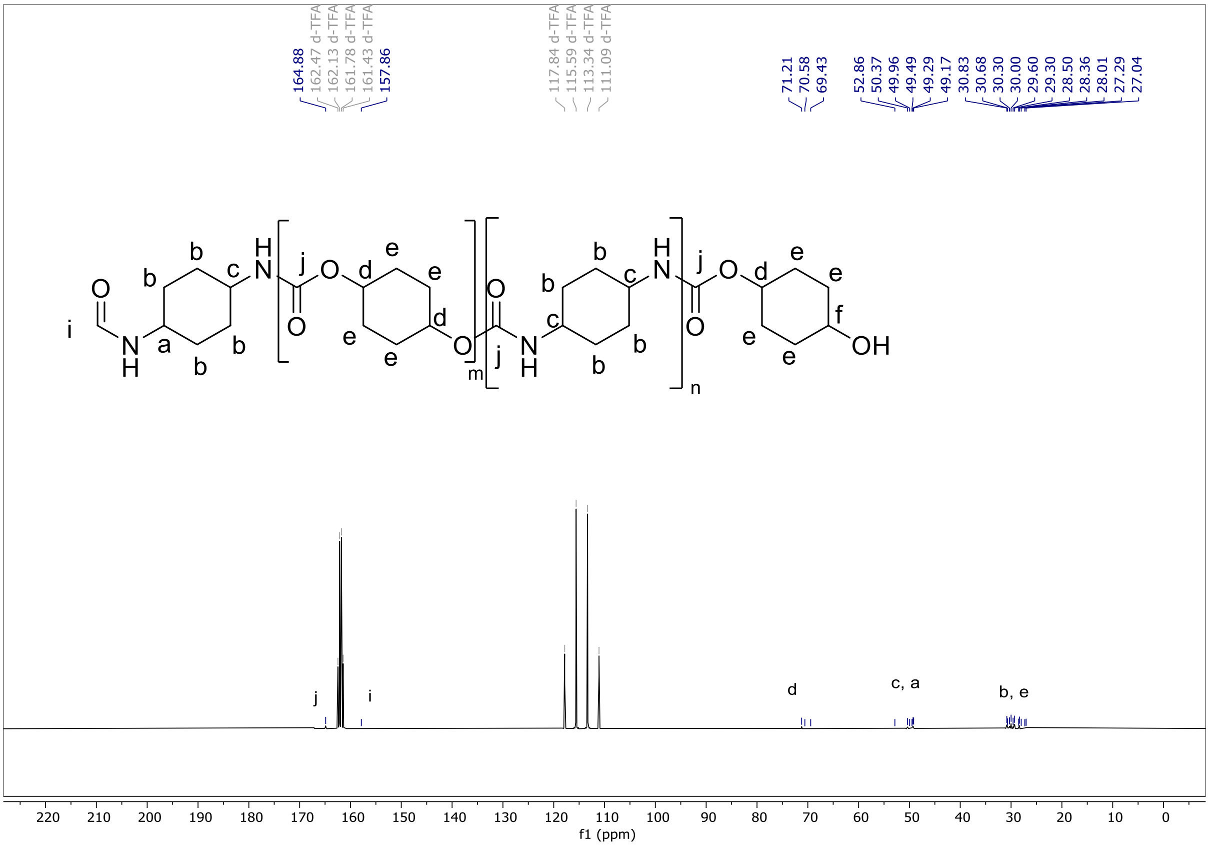

Figure S123: ^{13}C{^{1}H} NMR spectrum (d-TFA, 126 MHz, 298 K) of Table 4, entry 3.

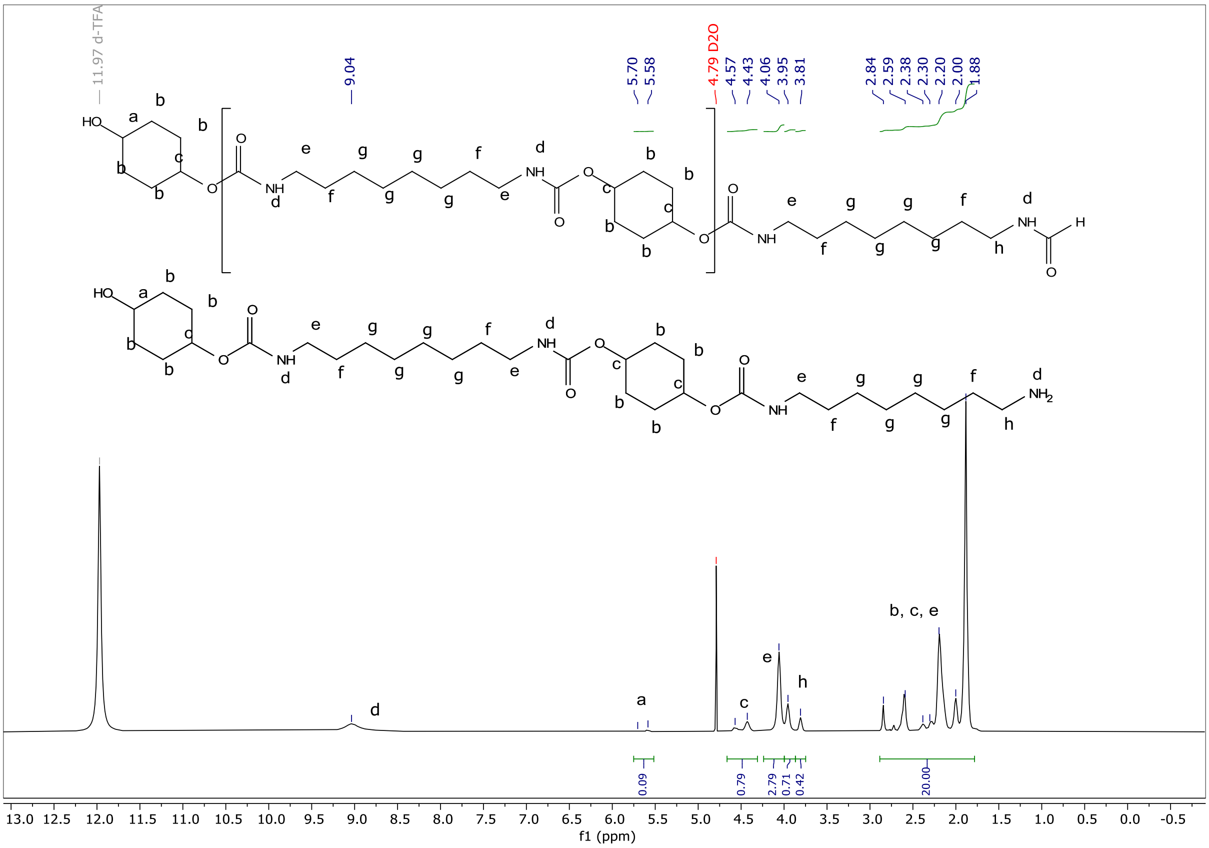

Figure S124: ^{1}H NMR spectrum (*d*-TFA, 500 MHz, 298 K) of Table 4, entry 4.

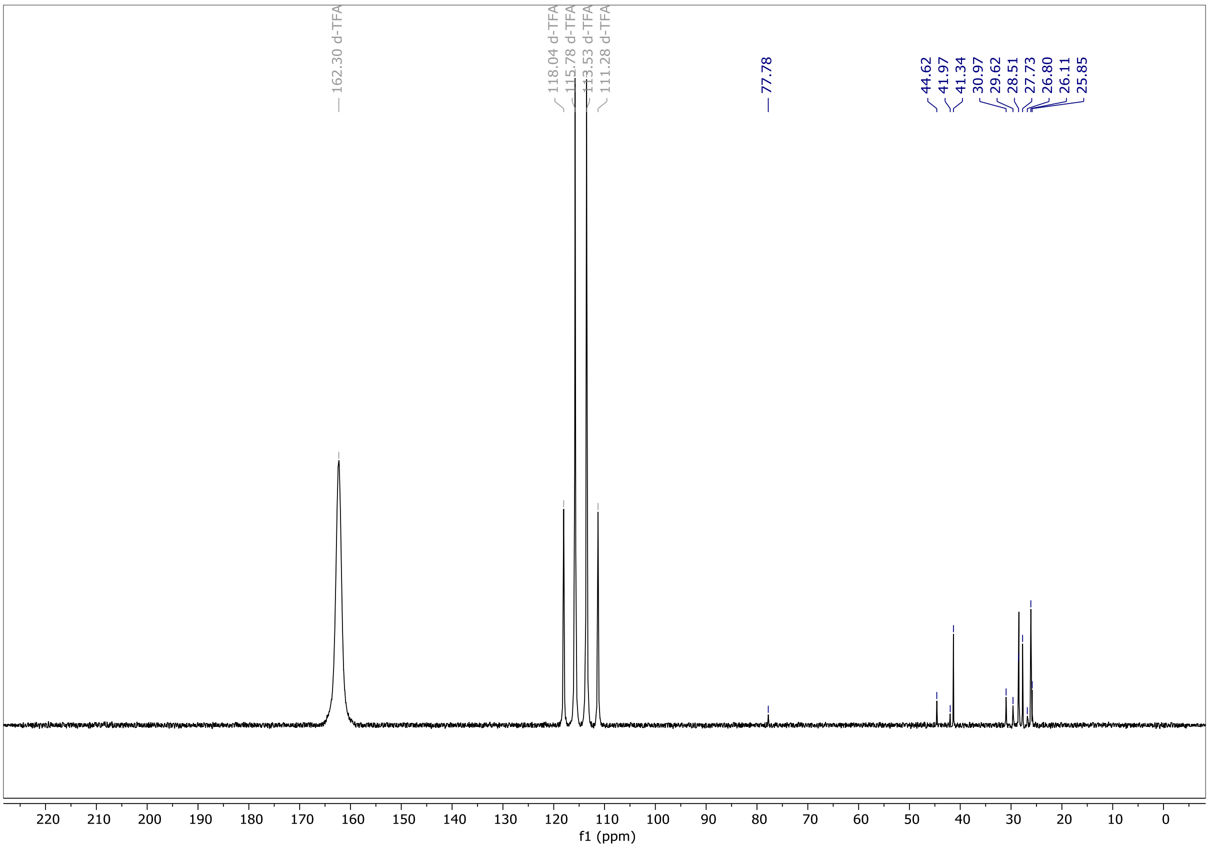

Figure S125: $^{13}C\{^{1}H\}$ NMR spectrum (*d*-TFA, 126 MHz, 298 K) of Table 4, entry 4.

3.4.2. IR spectra for polyurea(urethane) synthesis

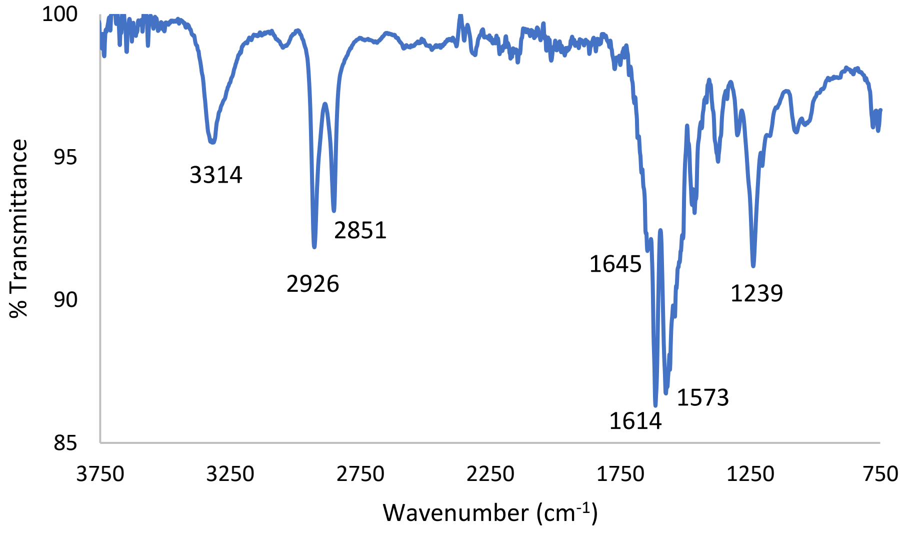

Figure S126: IR spectrum of polyurea(urethane) corresponding to Table 3, entry 1.

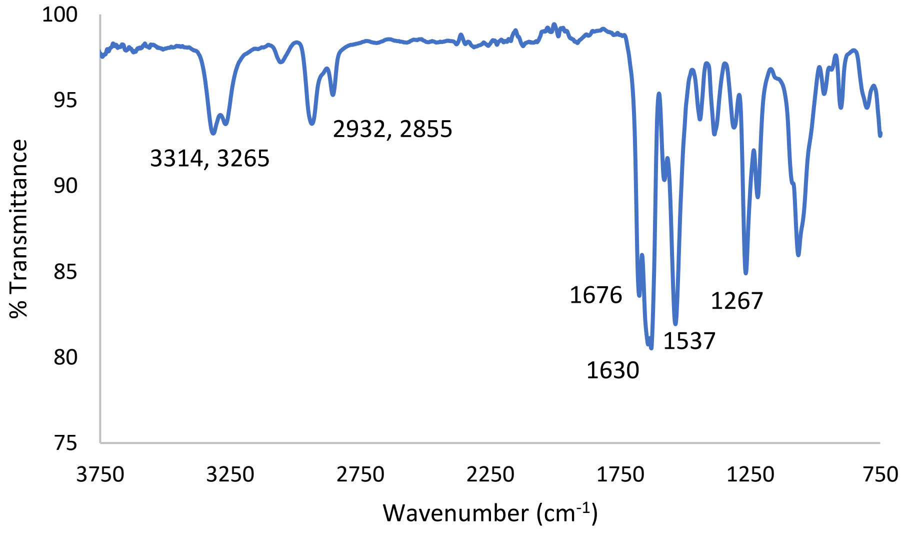

Figure S127: IR spectrum of polyurea(urethane) corresponding to Table 3, entry 2.

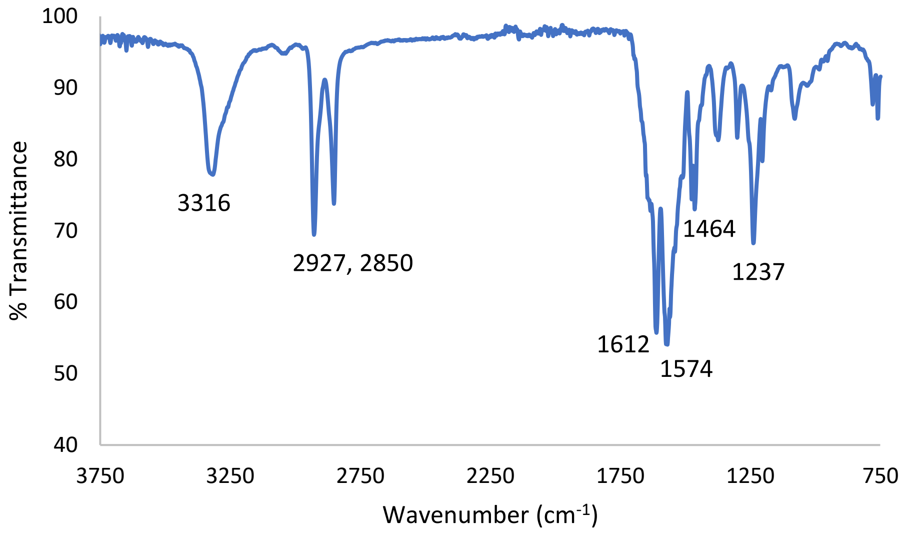

Figure S128: IR spectrum of polyurea(urethane) corresponding to Table 3, entry 3.

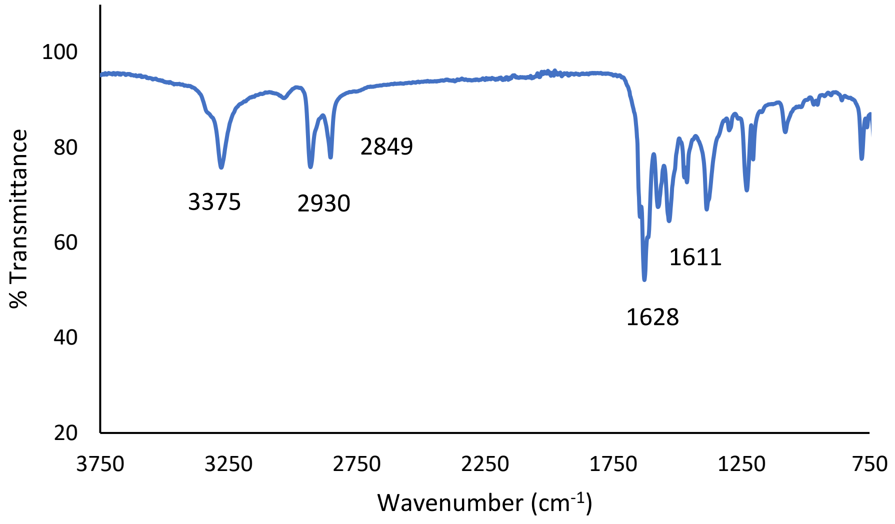

Figure S129: IR spectrum of polyurea(urethane) corresponding to Table 3, entry 4.

3.4.3. DSC data for polyurea(urethane)

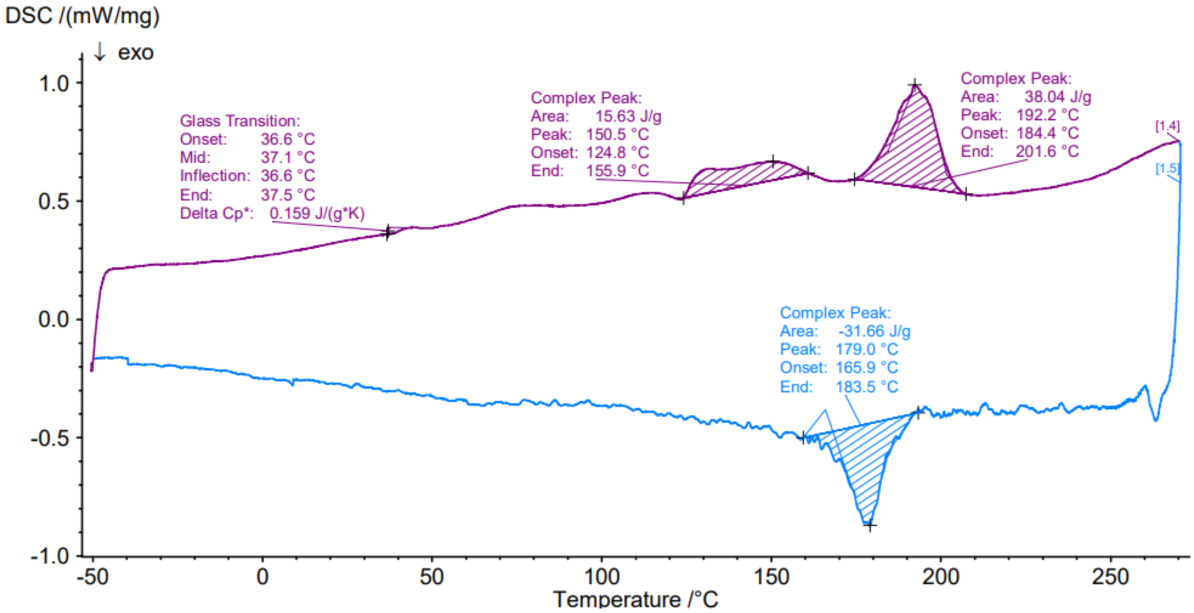

Figure S130: DSC trace corresponding to Table 4, entry 1.

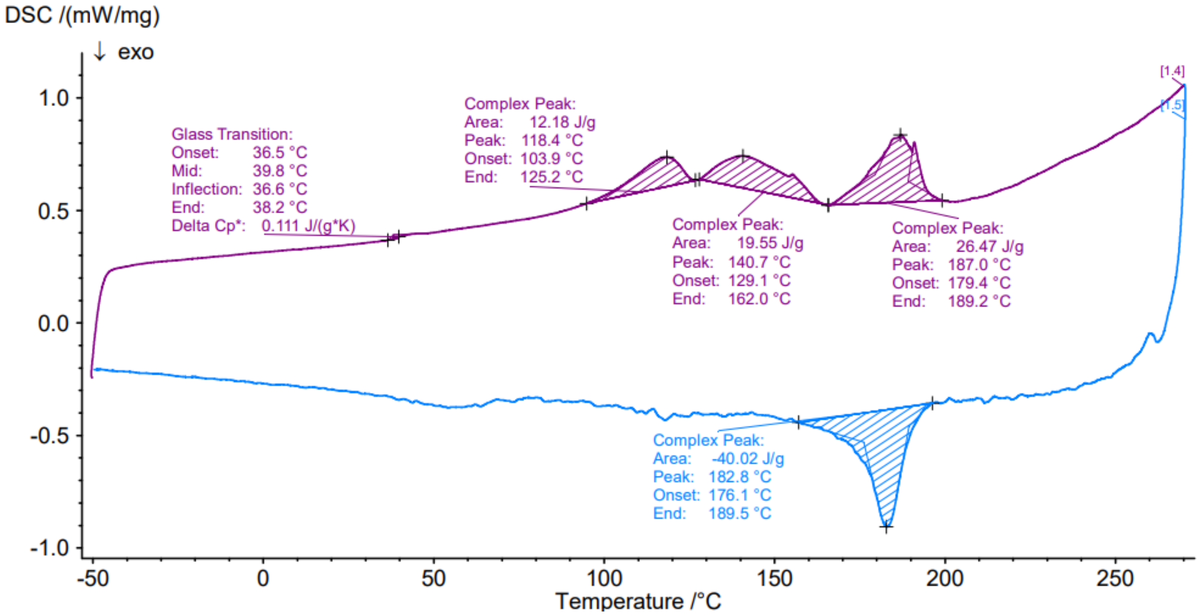

Figure S131: DSC trace corresponding to Table 4, entry 2.

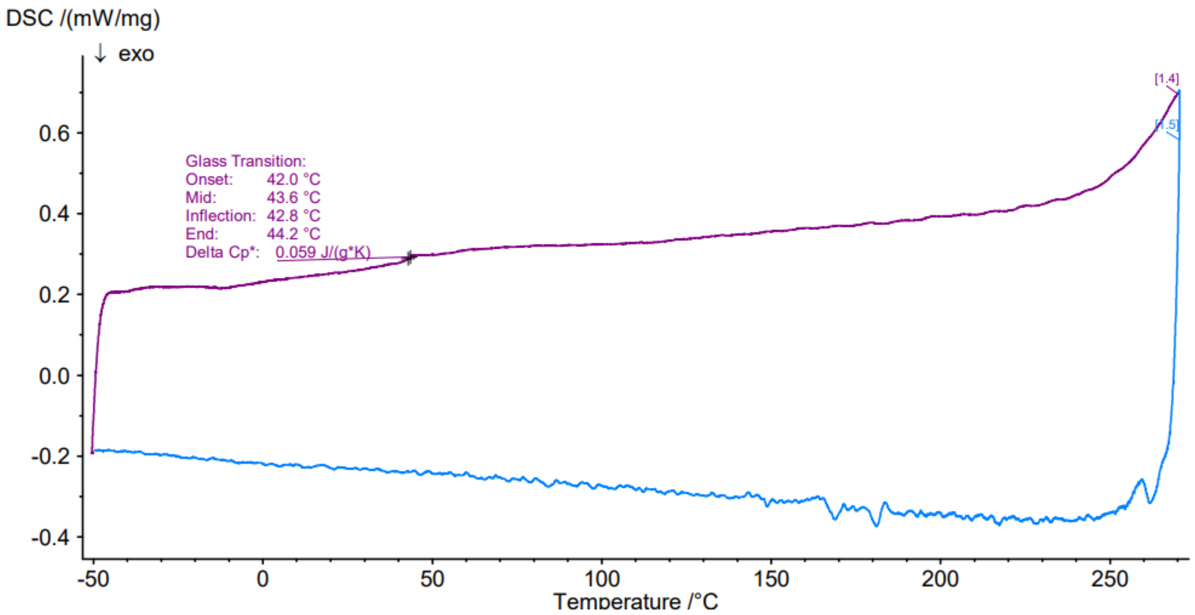

Figure S132: DSC trace corresponding to Table 4, entry 3.

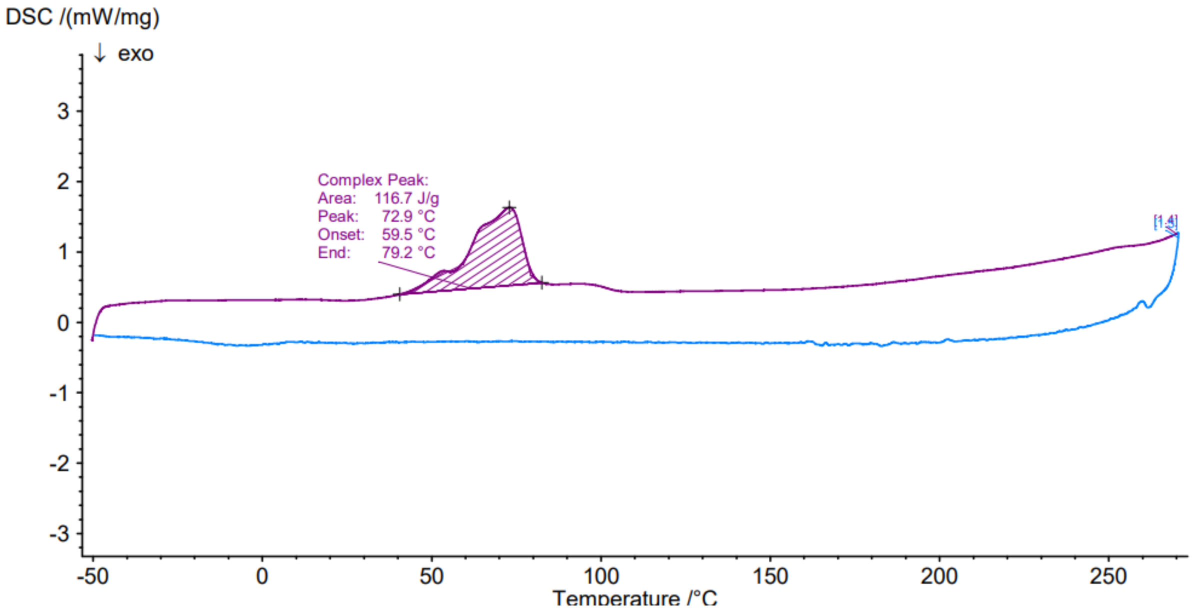

Figure S133: DSC trace corresponding to Table 4, entry 4.

3.4.4. TGA data for polyurea(urethane)

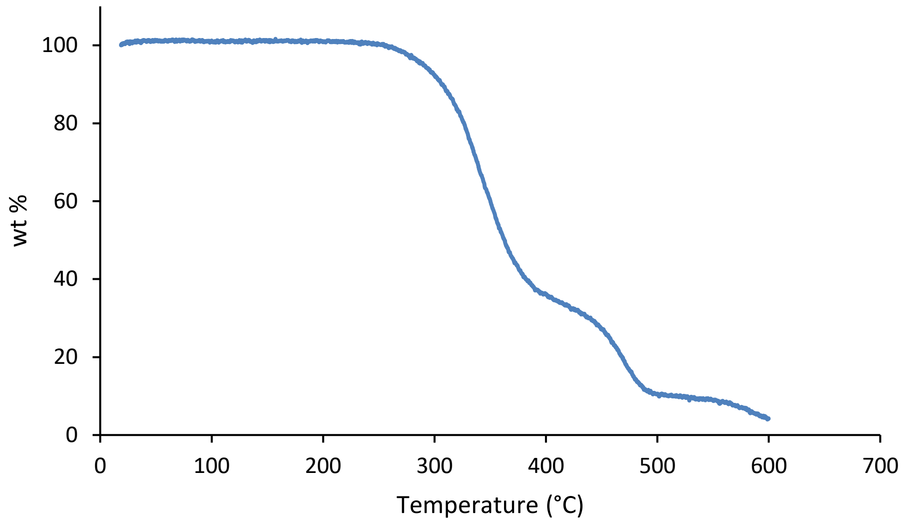

Figure S134: TGA data for polyurea(urethane) corresponding to Table 4, entry 1.

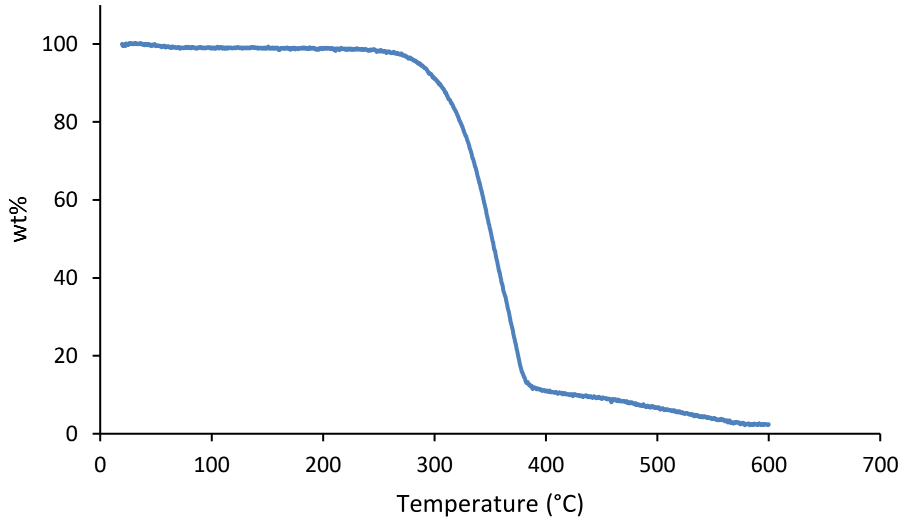

Figure S135: TGA data for polyurea(urethane) corresponding to Table 4, entry 2.

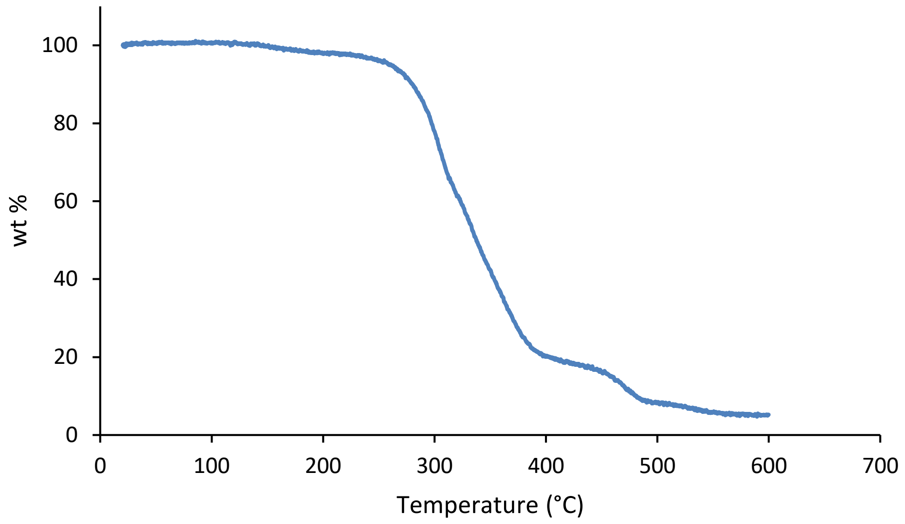

Figure S136: TGA data for polyurea(urethane) corresponding to Table 4, entry 3.

3.4.5. MALDI-TOF spectra for polyurea(urethane)

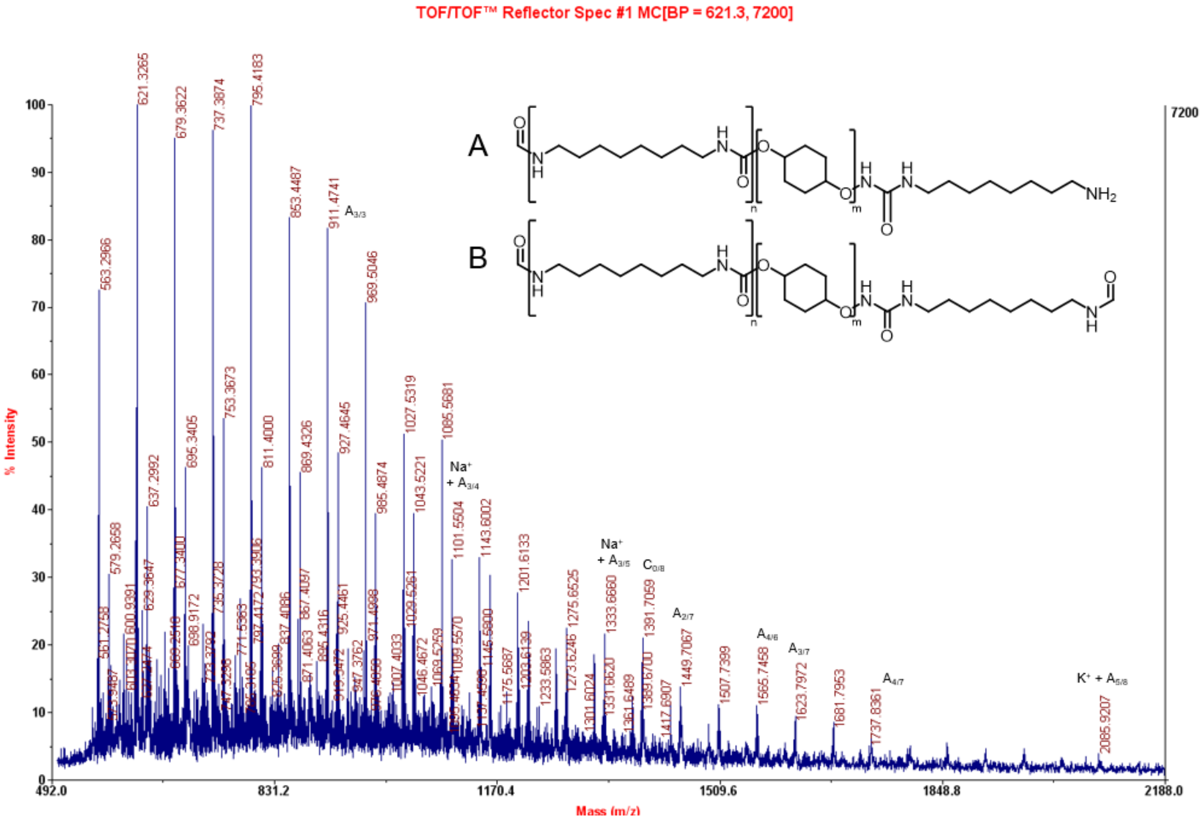

Figure S137: MALDI-TOF mass spectrum of polyurea corresponding to Table 4, entry 1, where the mass assignment is given in the form $X_{y/z}$, where “X” denotes polymer structure “A” or “B”, and “y” or “z” denote the value of repeating units “m” and “n”, respectively.

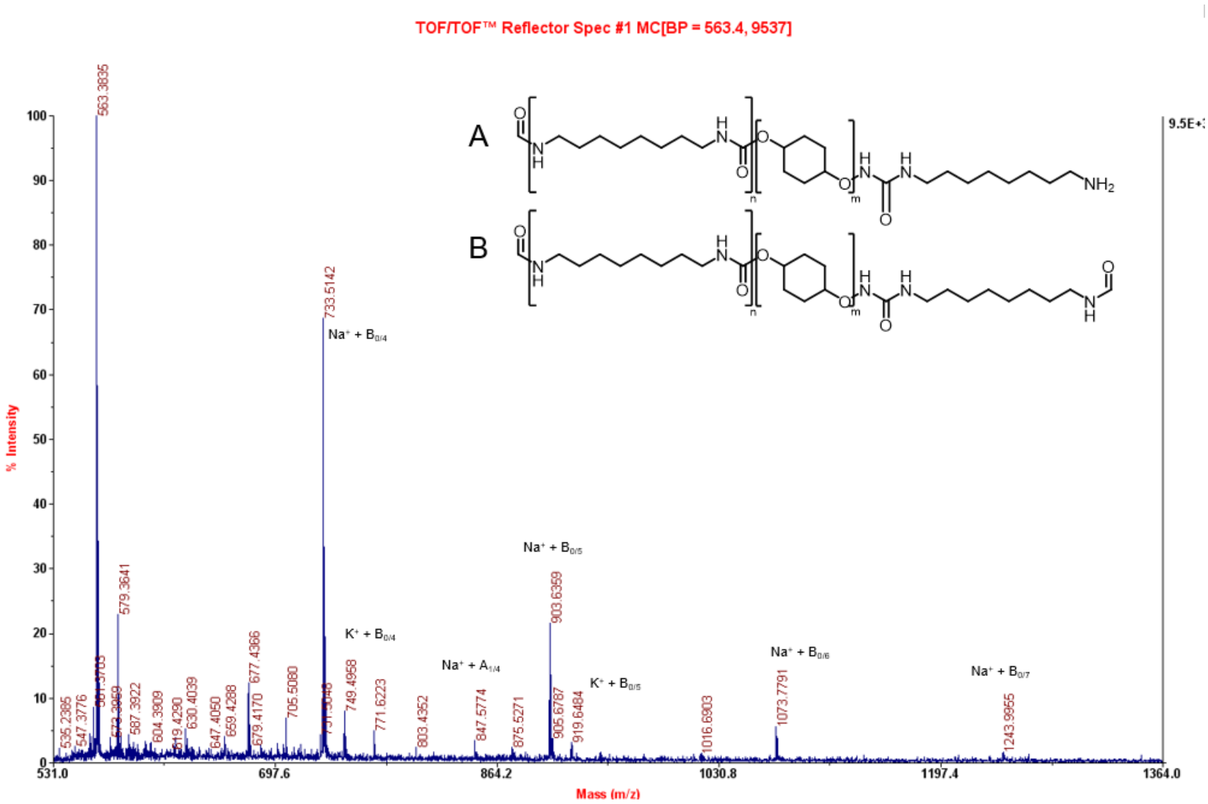

Figure S138: MALDI-TOF mass spectrum of polyurea corresponding to Table 4, entry 2, where the mass assignment is given in the form $X_{y/z}$, where "X" denotes polymer structure "A" or "B", and "y" or "z" denote the value of repeating units "m" and "n", respectively.

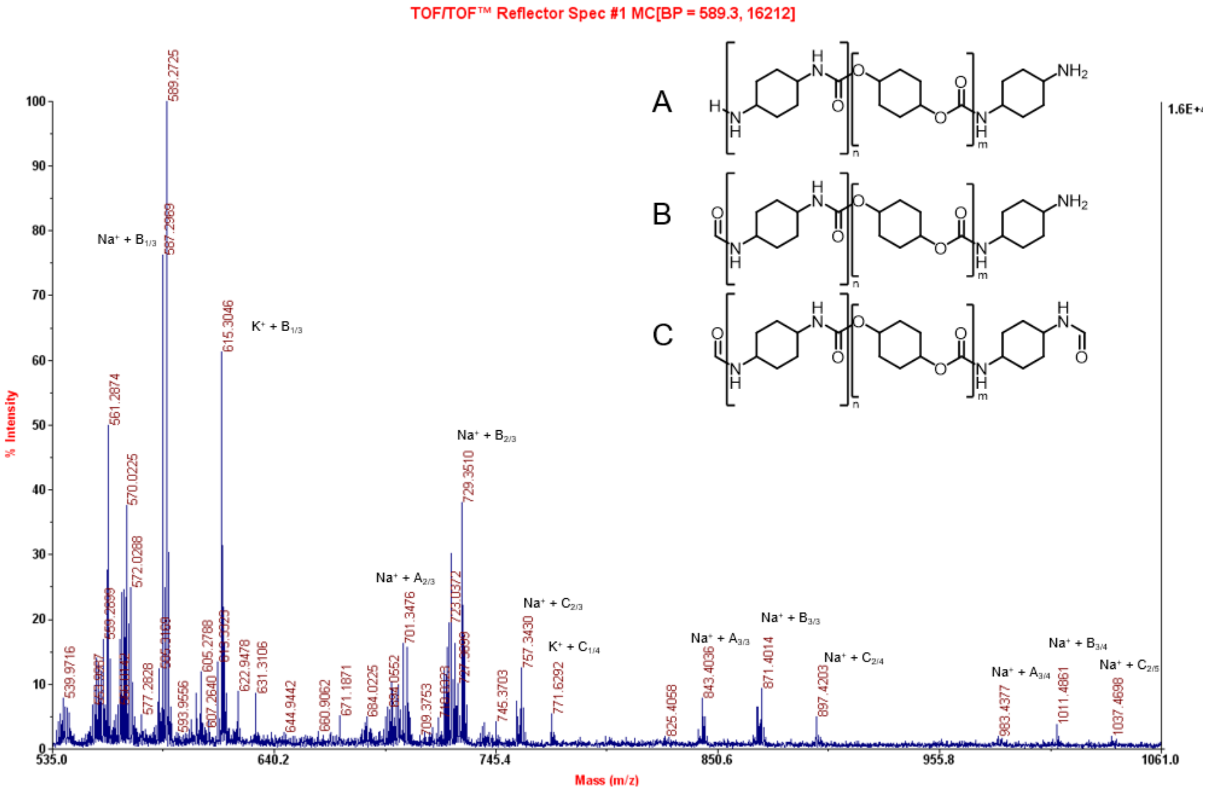

Figure S139: MALDI-TOF mass spectrum of polyurea corresponding to Table 4, entry 2, where the mass assignment is given in the form $X_{y/z}$, where "X" denotes polymer structure "A" or "B", and "y" or "z" denote the value of repeating units "m" and "n", respectively.

3.4.6. GPC traces of polyurea(urethane)

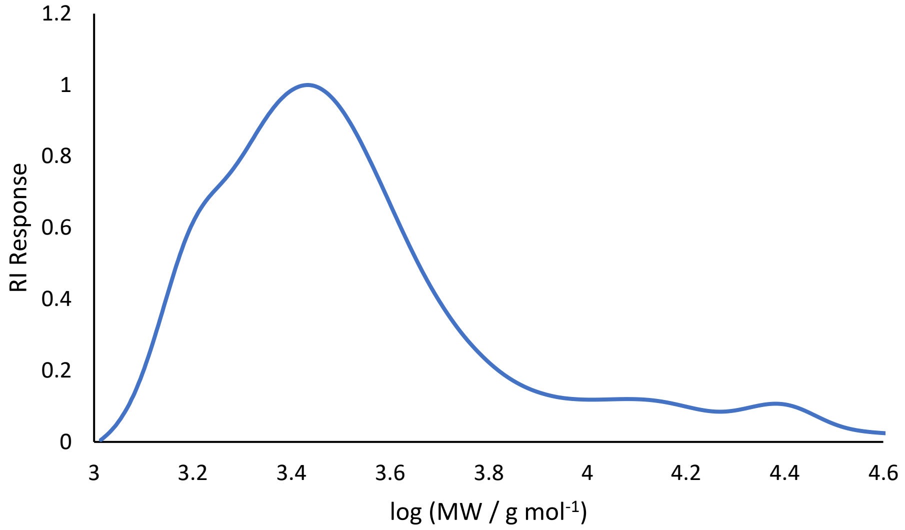

Figure S140: GPC trace of polyurea(urethane) corresponding to Table 4, entry 1.

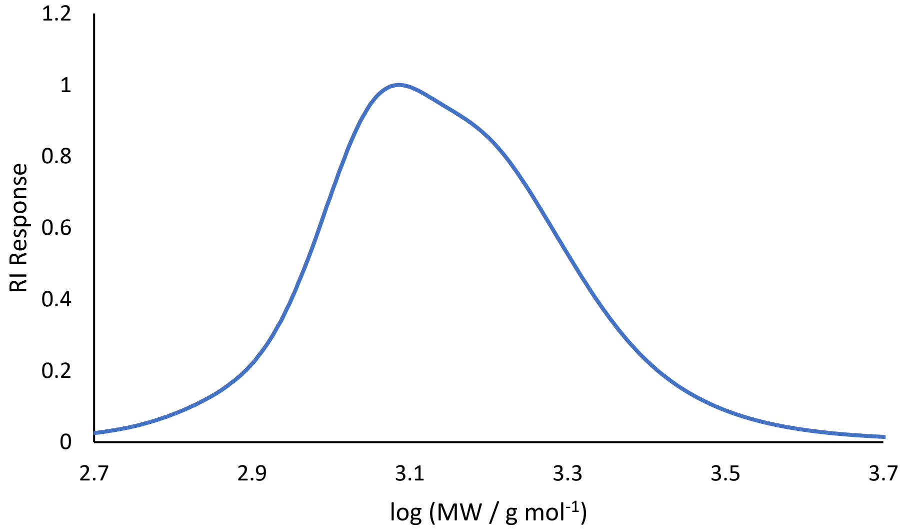

Figure S141: GPC trace of polyurea(urethane) corresponding to Table 4, entry 2.

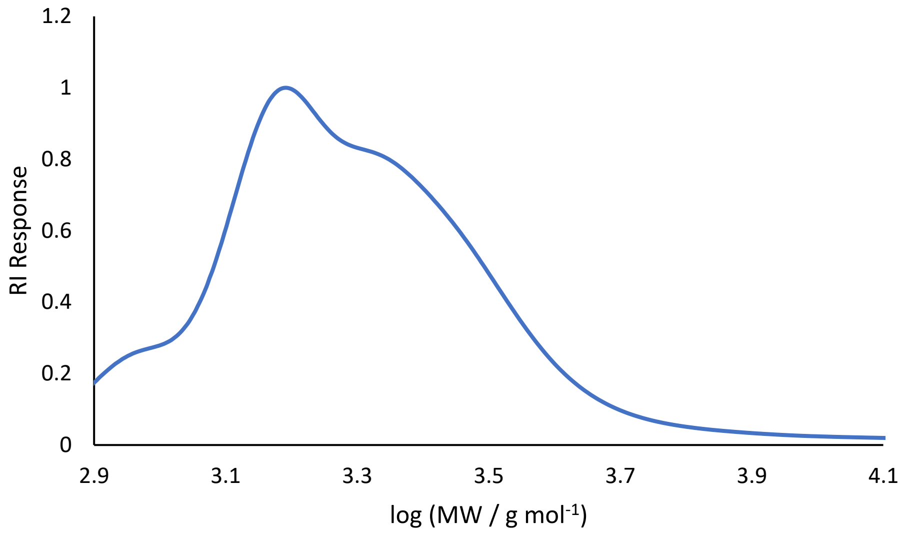

Figure S142: GPC trace of polyurea(urethane) corresponding to Table 4, entry 3.

3.5. Characterisation data for polyurea from recycling studies

3.5.1. ^{1}H NMR and ^{13}C{^{1}H} NMR data for polyurea from catalyst recycling studies

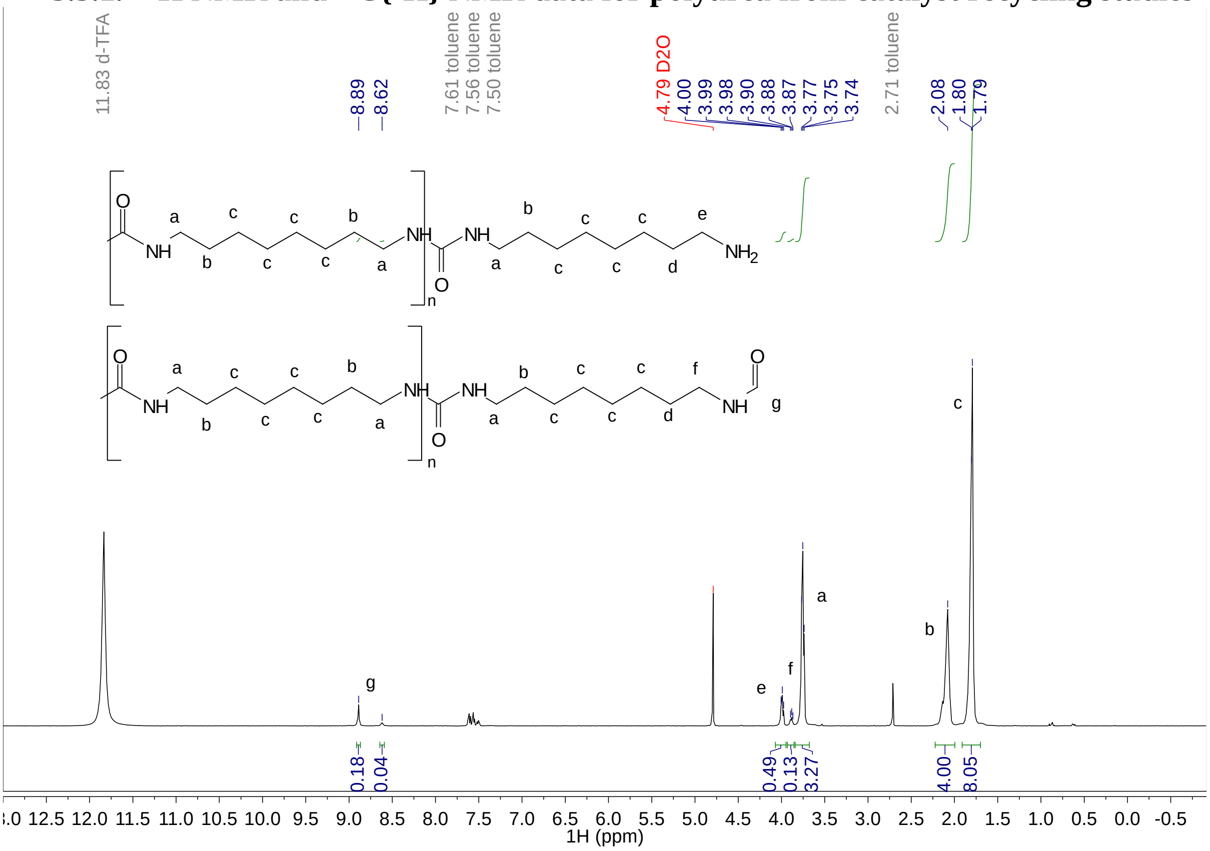

Figure S143: ^{1}H NMR spectrum (*d*-TFA, 500 MHz, 298 K) of Table 5, entry 1.

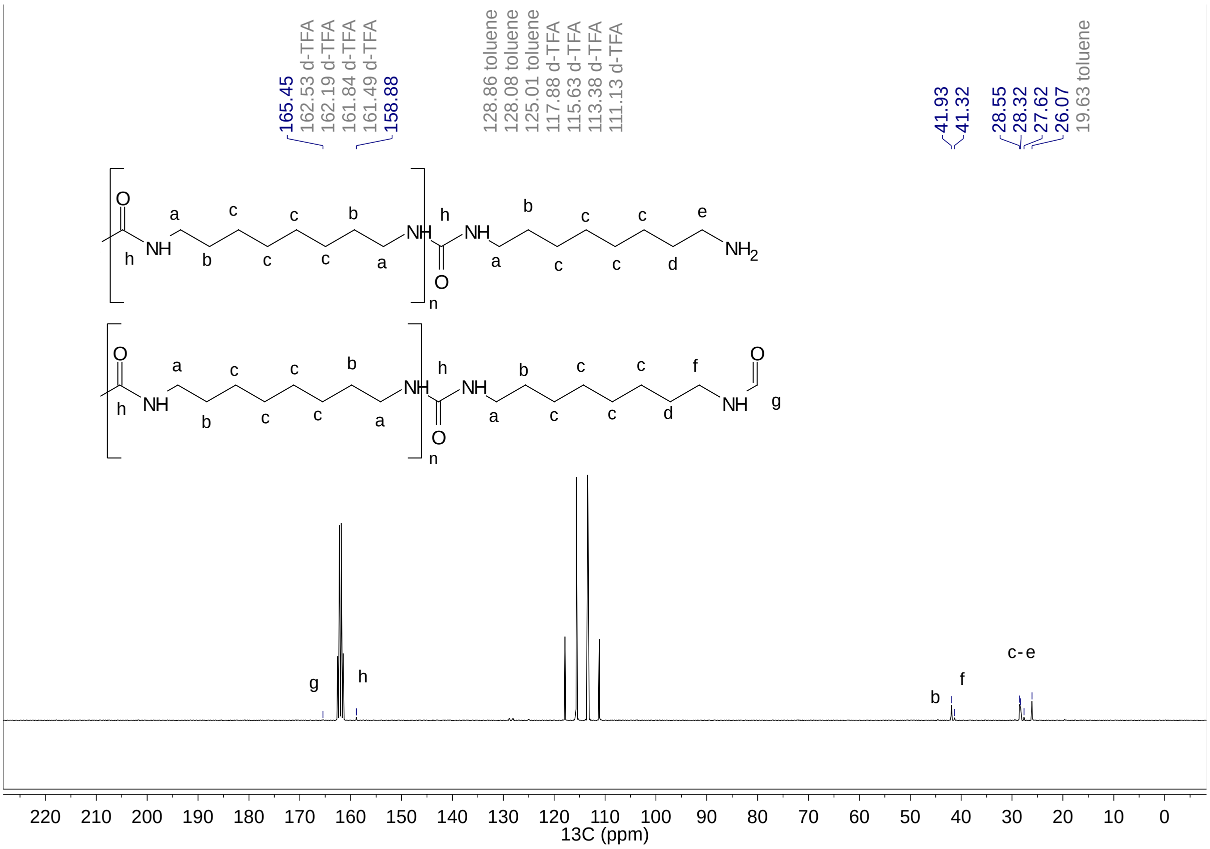

Figure S144: $^{13}C\{^{1}H\}$ NMR spectrum (*d*-TFA, 126 MHz, 298 K) of Table 5, entry 1.

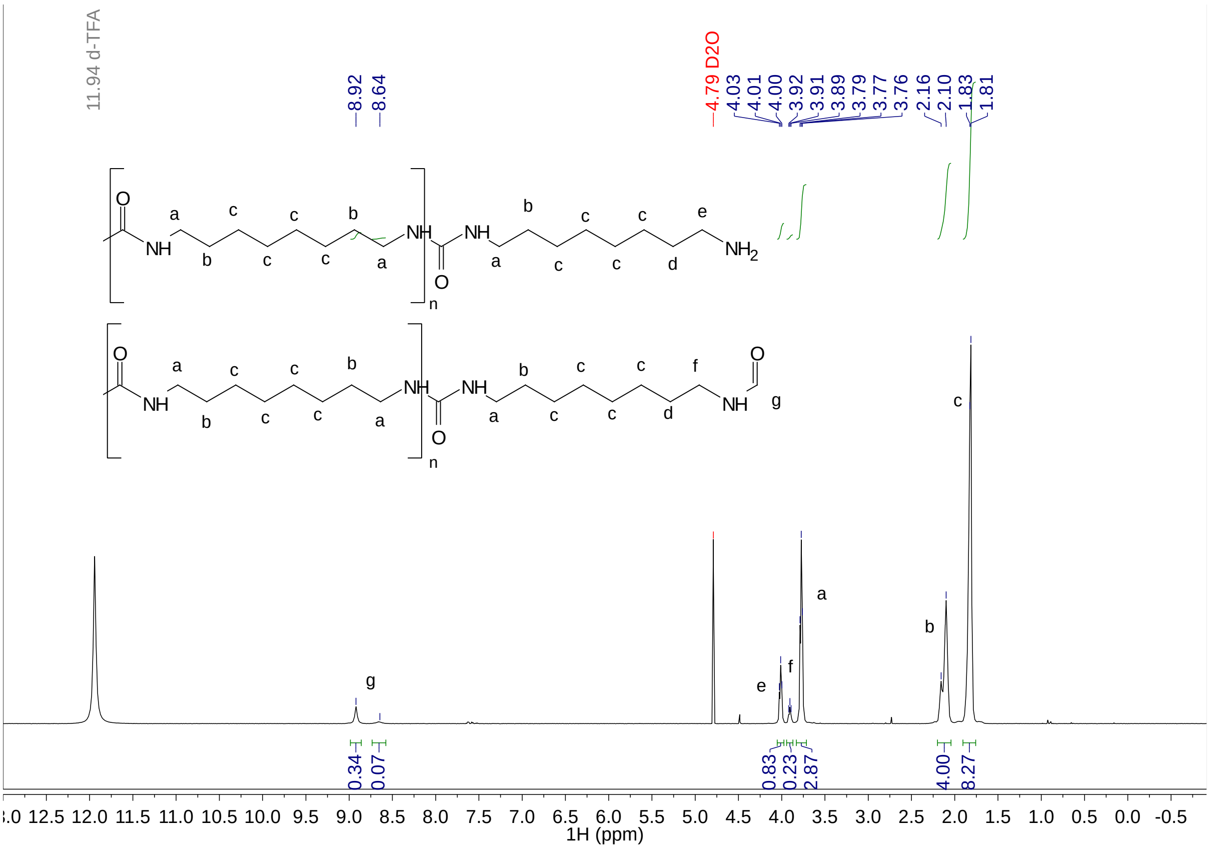

Figure S145: ^{1}H NMR spectrum (*d*-TFA, 500 MHz, 298 K) of Table 5, entry 2.

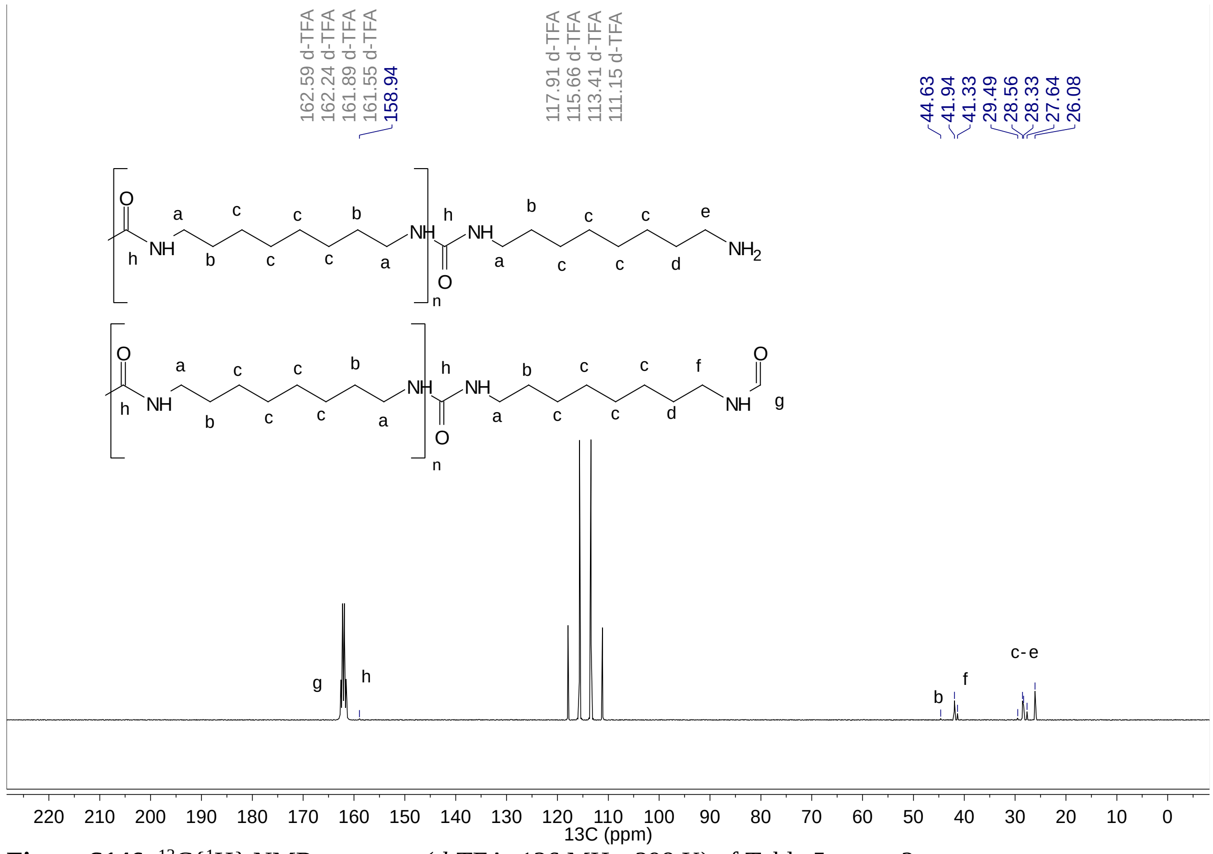

Figure S146: $^{13}C\{^1H\}$ NMR spectrum (*d*-TFA, 126 MHz, 298 K) of Table 5, entry 2.

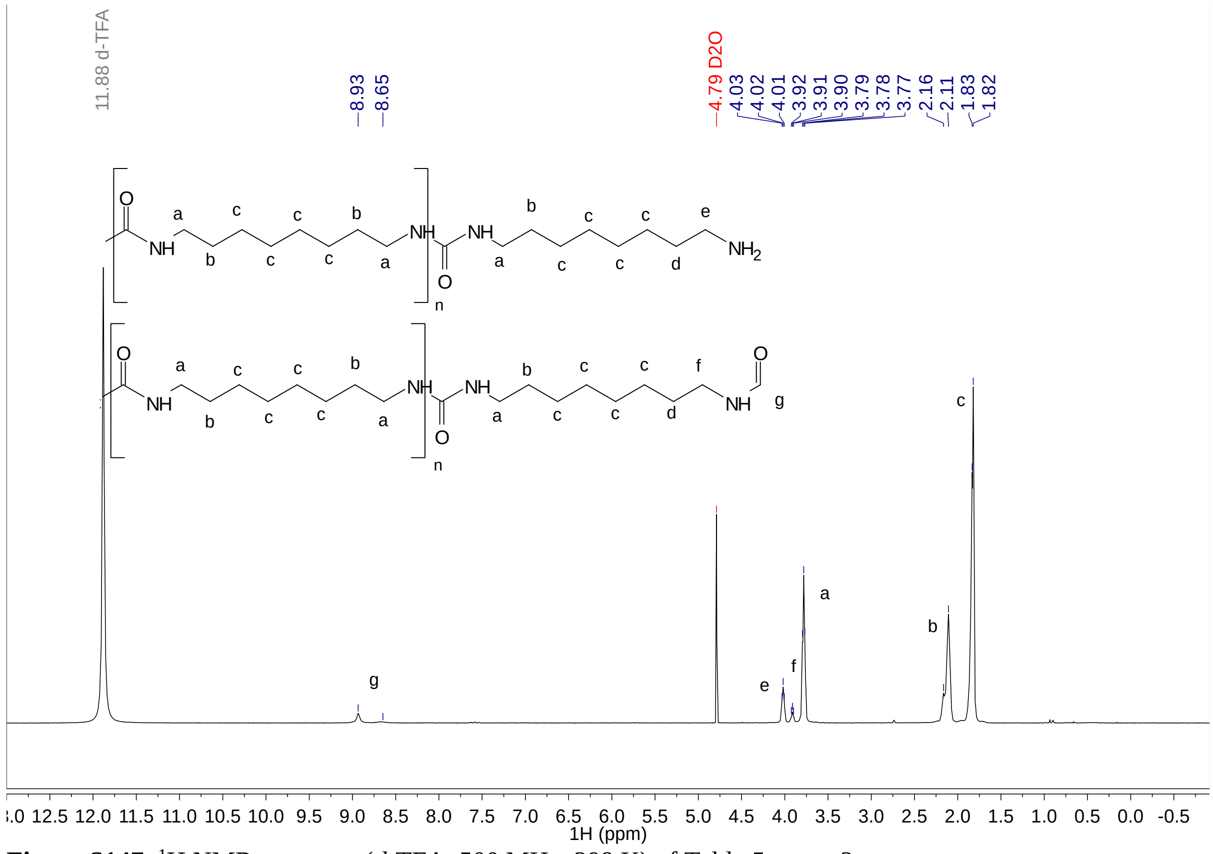

Figure S147: ^1H NMR spectrum (*d*-TFA, 500 MHz, 298 K) of Table 5, entry 3.

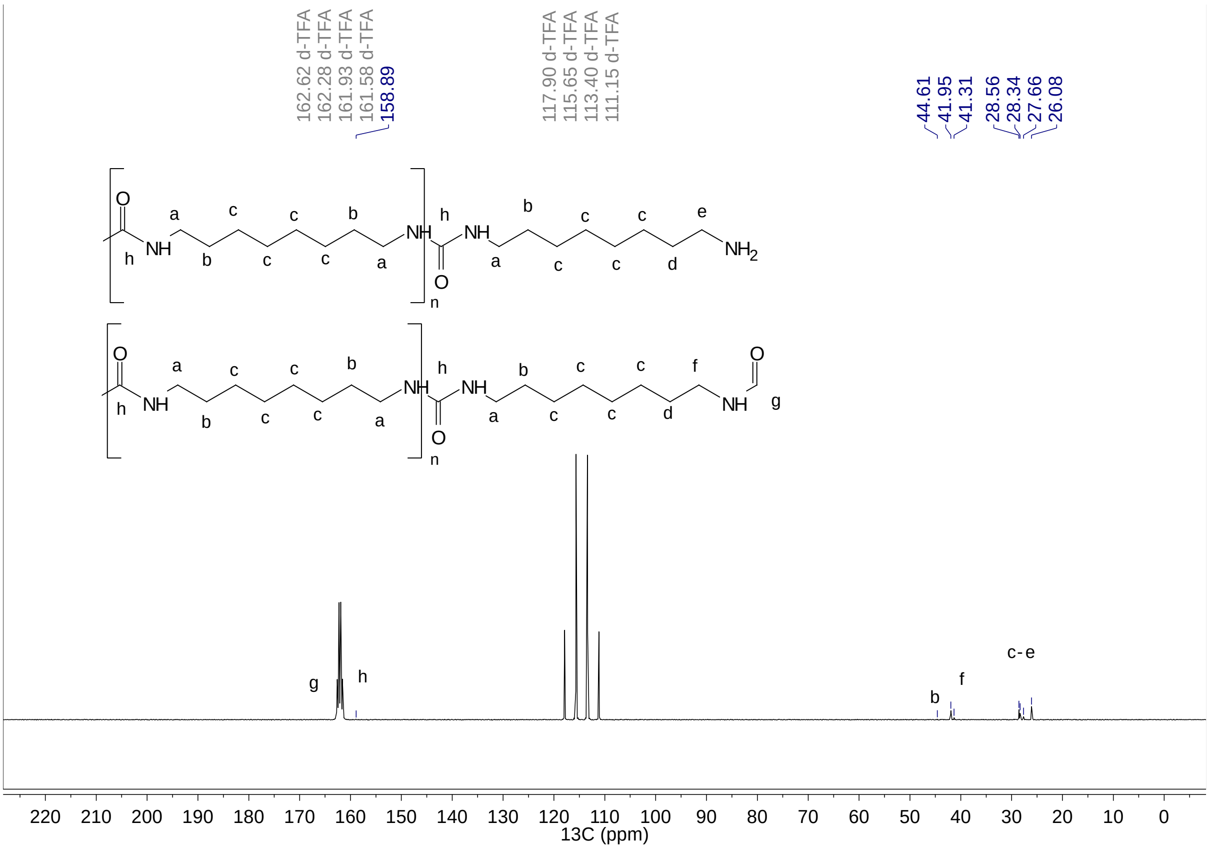

Figure S148: $^{13}C\{^{1}H\}$ NMR spectrum (*d*-TFA, 126 MHz, 298 K) of Table 5, entry 3.

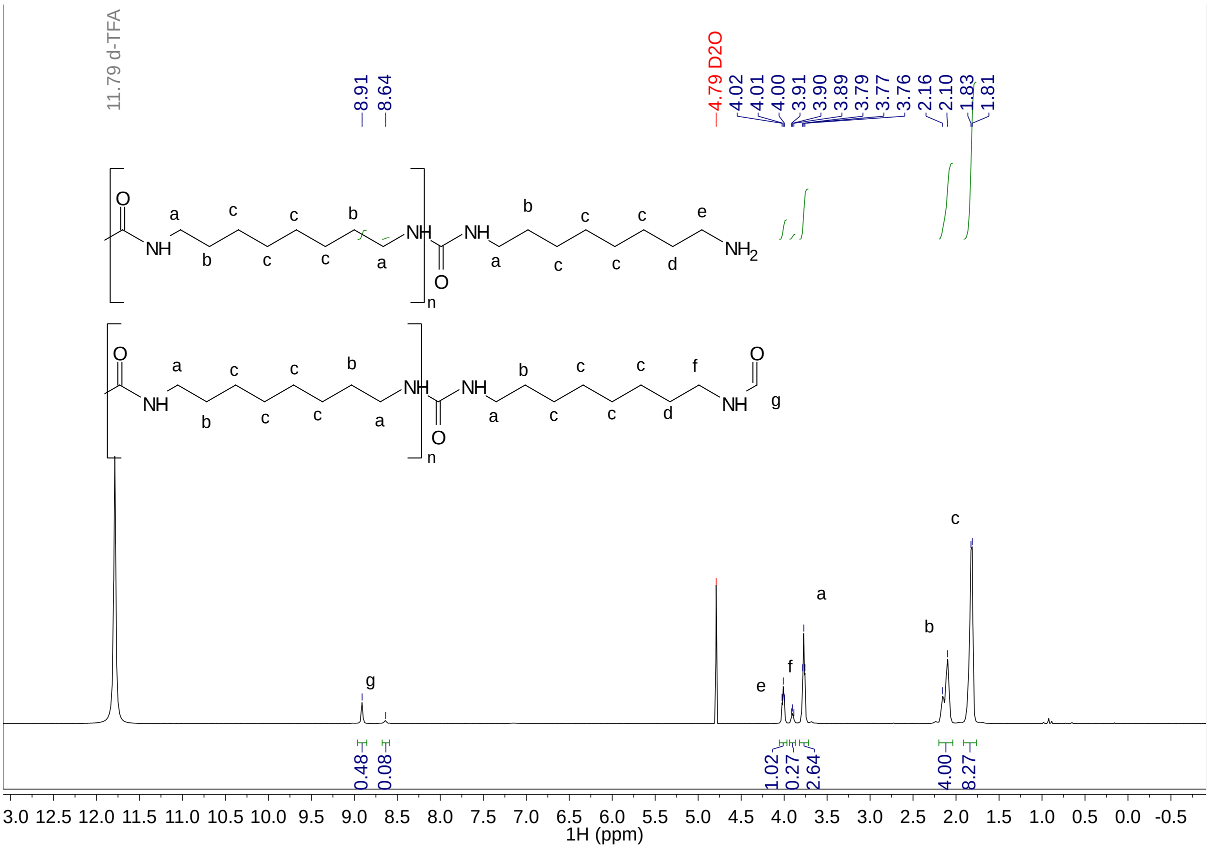

Figure S149: ^{1}H NMR spectrum (*d*-TFA, 500 MHz, 298 K) of Table 5, entry 4.

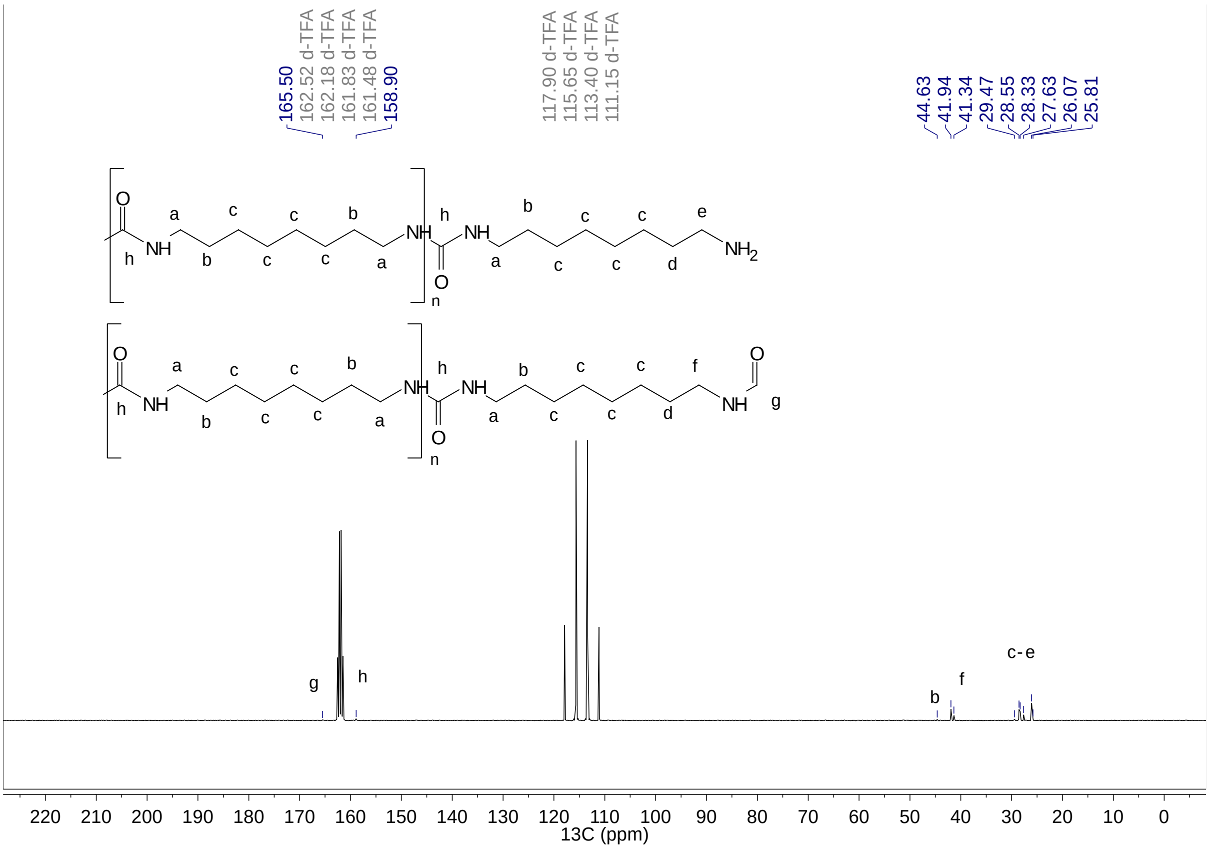

Figure S150: $^{13}C\{^{1}H\}$ NMR spectrum (*d*-TFA, 126 MHz, 298 K) of Table 5, entry 4.

3.5.2. IR data for polyurea from catalyst recycling studies

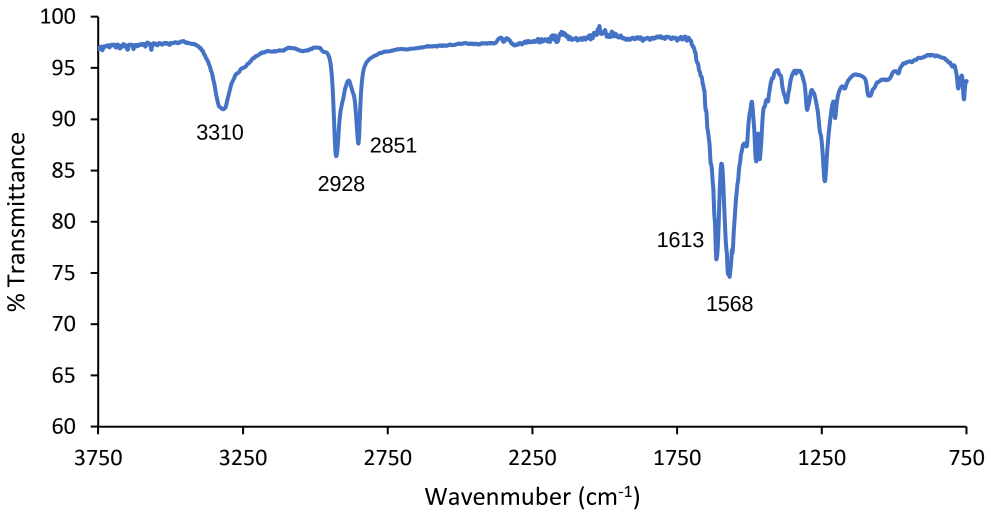

Figure S151: IR data corresponding to Table 5, entry 1.

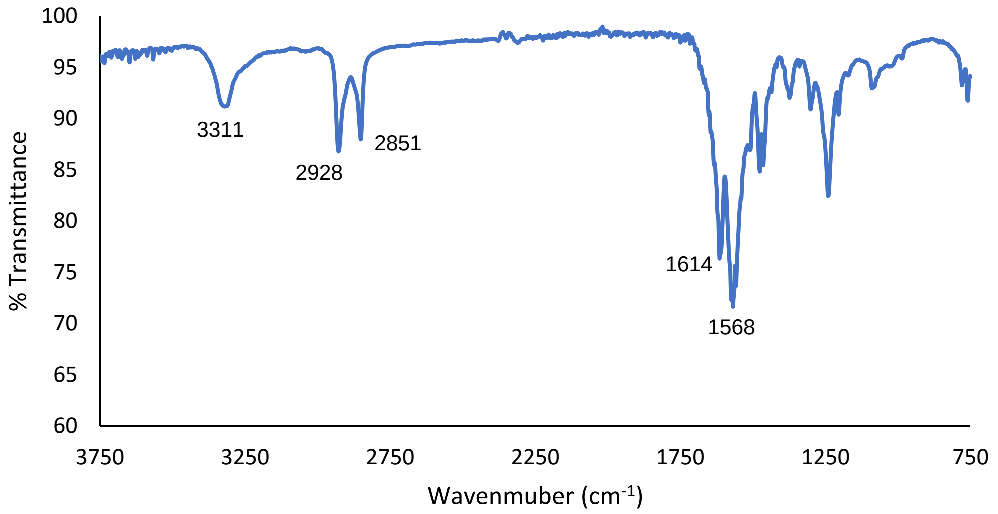

Figure S152: IR data corresponding to Table 5, entry 2.

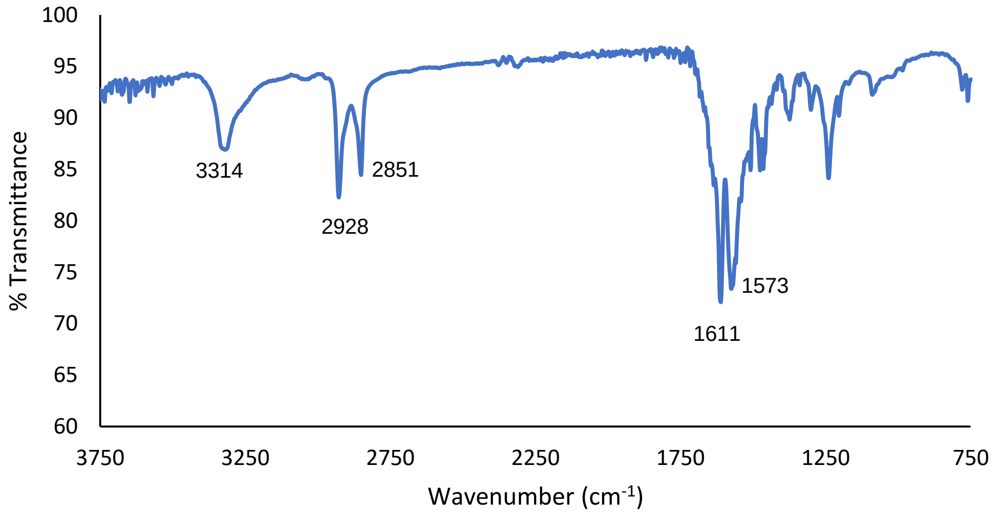

Figure S153: IR data corresponding to Table 5, entry 3.

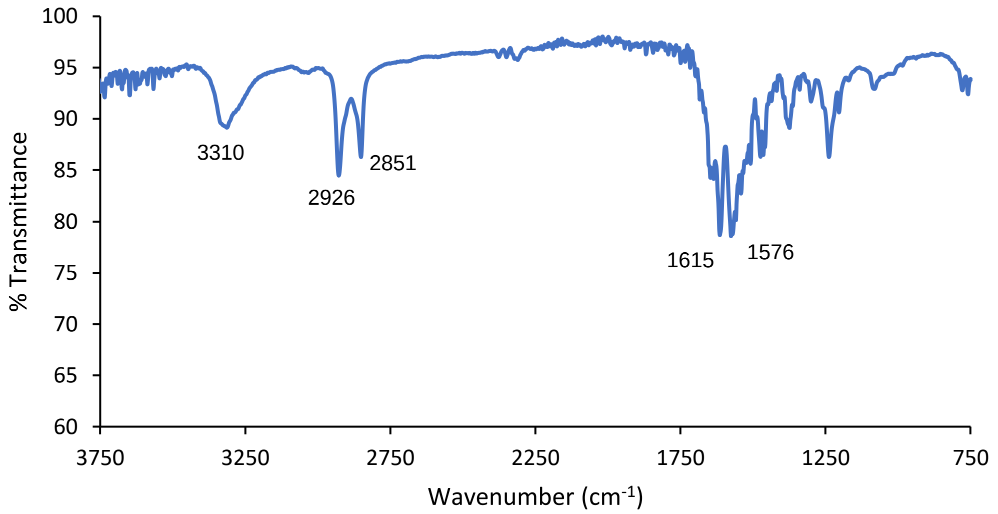

Figure S154: IR data corresponding to Table 5, entry 4.

4. Mechanistic Investigations

4.1. Stoichiometric reaction of complex 1 with base and formamides

General Procedure: A J-Young's NMR tube was charged with complex **1** (12.1 mg, 0.02 mmol), KO^{t}Bu (2.7 mg, 0.024 mmol) and formanilide or *N*-benzylformamide (0.024 mmol) before being vacuum cycled 3 times with argon. toluene-*d*$_8$ (*ca.* 0.5 mL) was added in a glovebox and the NMR tube was shaken vigorously. The NMR tube was then heated at 110 °C. NMR spectra taken after heating the NMR tube for 10 min at 110 °C showed the formation of complex **1a**. Continued heating of the tube at 110 °C led to the formation of mixture of species as shown in Table S1.

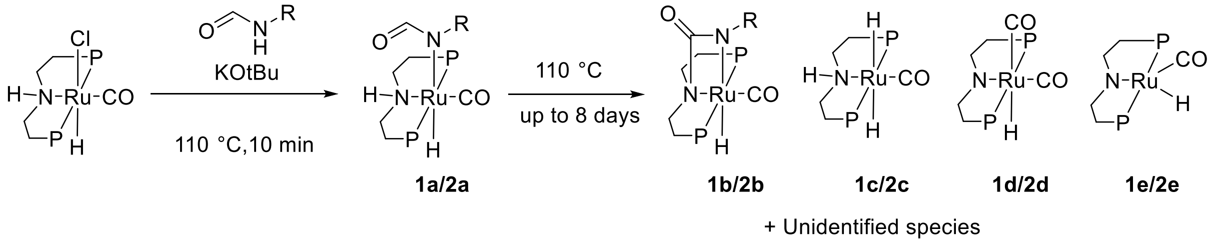

(where P is representative of PPh_2 or P^iPr_2 for complexes **1** and **2** for clarity; R = Ph or Bn)

Table S3: Complexes formed upon heating complex **1** or **2** with KO^{t}Bu and a formamide.[a,b]

		Yield (%) after given time (d)					
**R**	**Complex**	**10 min**	**1 day**	**2 days**	**4 days**	**7 days**	**8 days**
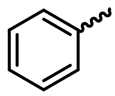	**1a**[c]	90% yield	0% yield	0% yield	0% yield	0% yield	-
	**1b**[c]	0% yield	30% yield	13% yield	5% yield	4% yield	-
	**1c**	10% yield	0% yield	0% yield	6% yield	9% yield	-
	**1d**	0% yield	11% yield	38% yield	40% yield	37% yield	-
	**1e**	0% yield	11% yield	11% yield	4% yield	4% yield	-
	**unidentified**[d]	0% yield	47% yield	38% yield	46% yield	46% yield	-
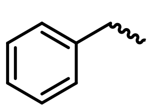	**1a'**	100% yield	100% yield	91% yield	76% yield	33% yield	25% yield
	**1b'**	0% yield	0% yield	3% yield	14% yield	42% yield	45% yield
	**1c**	0% yield	0% yield	6% yield	10% yield	17% yield	22% yield
	**1d**	0% yield	0% yield	0% yield	0% yield	4% yield	5% yield
	**1e**	0% yield	0% yield	0% yield	0% yield	0% yield	0% yield
	**unidentified**[d]	0% yield	0% yield	0% yield	0% yield	3% yield	3% yield
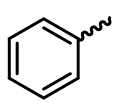	**2a**	100% yield	20% yield	9% yield	5%yield	6% yield	-
	**2b**	0% yield	80% yield	91% yield	81% yield	77% yield	-
	**2c**	0% yield	0% yield	0% yield	13% yield	14% yield	-
	**2d**	0% yield	0% yield	0% yield	1% yield	3% yield	-

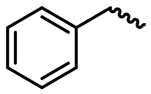	**2a'**	100% yield	46% yield	46% yield	44% yield	45% yield	-
	**2b'**	0% yield	53% yield	52% yield	47% yield	44% yield	-
	**2c**	0% yield	1% yield	2% yield	9% yield	10% yield	-

[a]Unless stated otherwise, the distribution given are acquired by the comparison of hydride shifts in the ^{1}H NMR spectrum. [b]The chemical shifts of species **1c** and **1d** were identified by comparison to literature values.[6,7] [c]The hydride chemical shifts of complexes **1a** and **1b** overlap, so the corresponding ^{31}P NMR chemical shifts were compared, and the overall integration of the hydride environment was divided accordingly. [d]unidentified species are proposed to be various intermediate species arising from the interaction of complex **1** and methanol as described by Leitner *et al.*.[8]

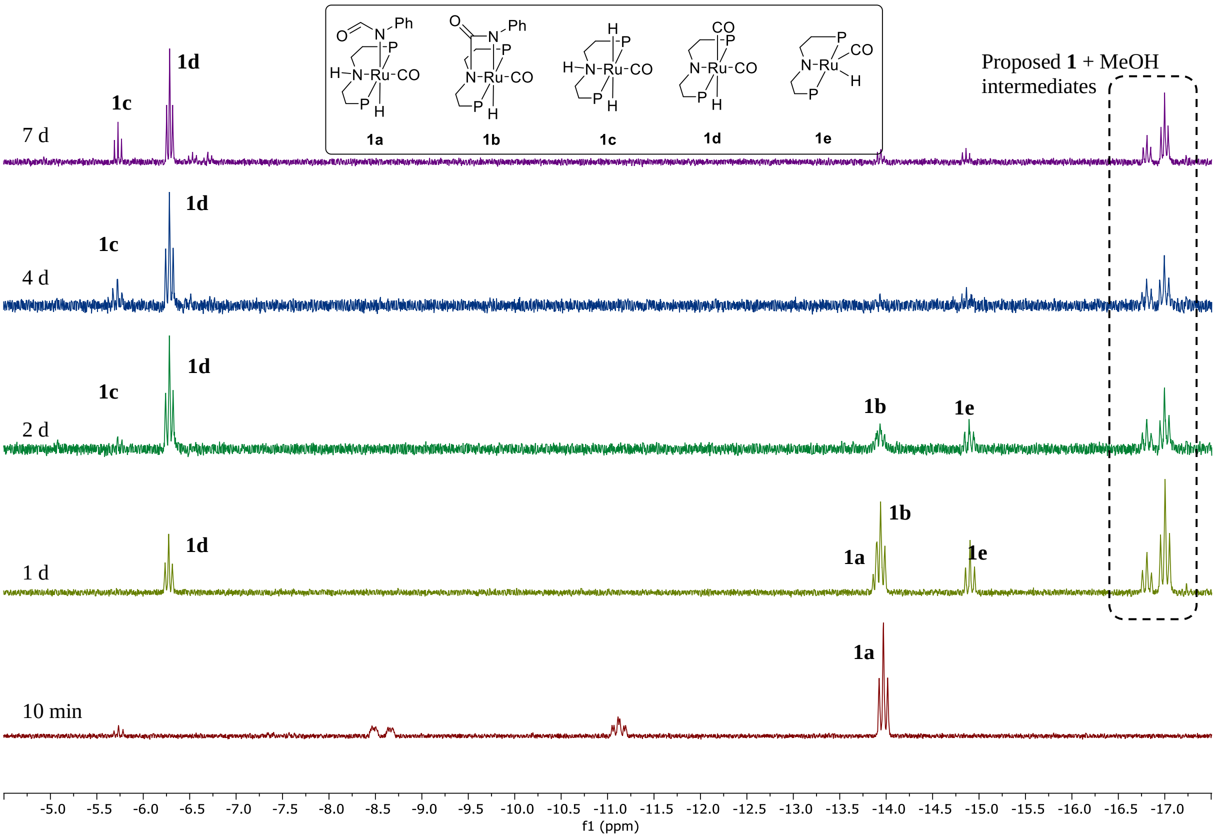

Figure S155: Selected region of the ^{1}H NMR spectra (toluene-d_8, 500 MHz, 298 K) showing temporal profile of the reaction of complex **1** + KO^{t}Bu with formanilide at 110 °C (Table S3, where R = Ph).

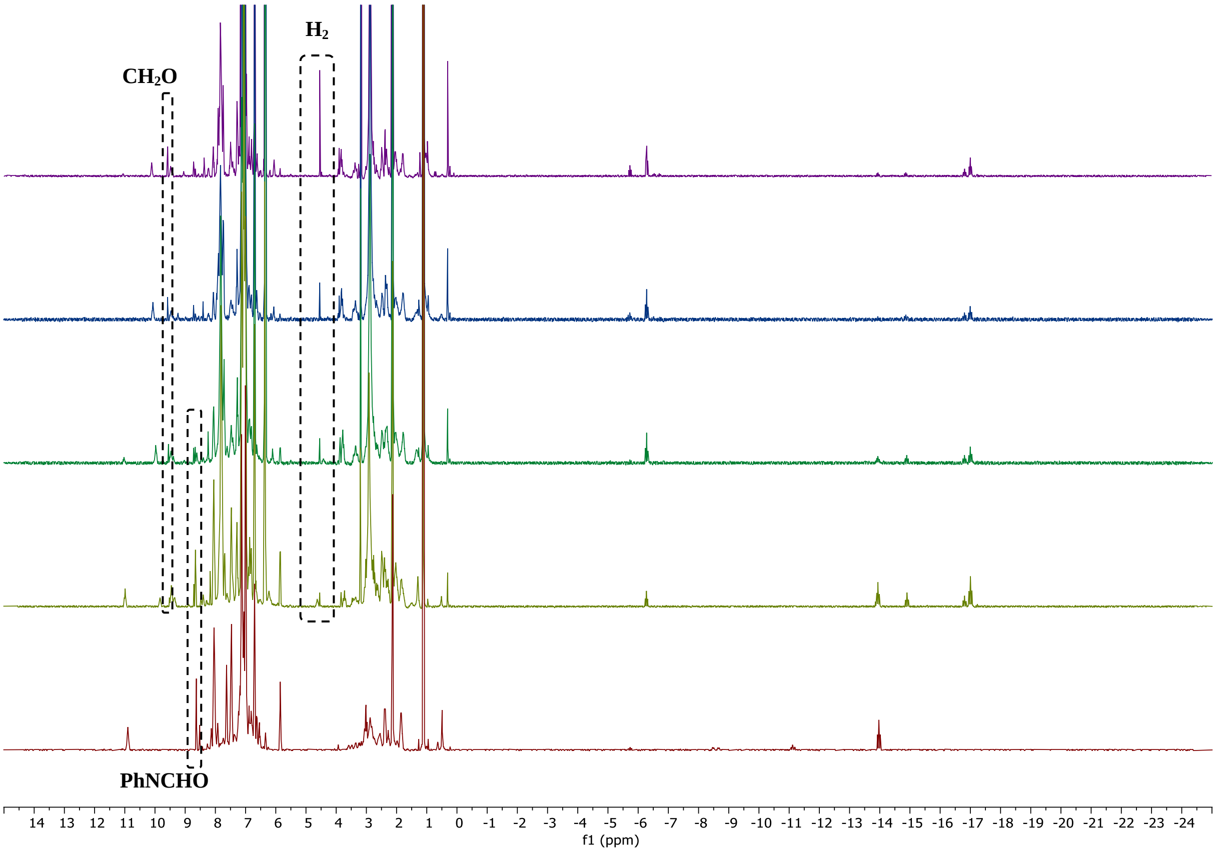

Figure S156: ^{1}H NMR spectra (toluene-d_8, 500 MHz, 298 K) showing temporal profile of the reaction of complex **1** + KO^{t}Bu with formanilide at 110 °C (Table S3, where R = Ph).

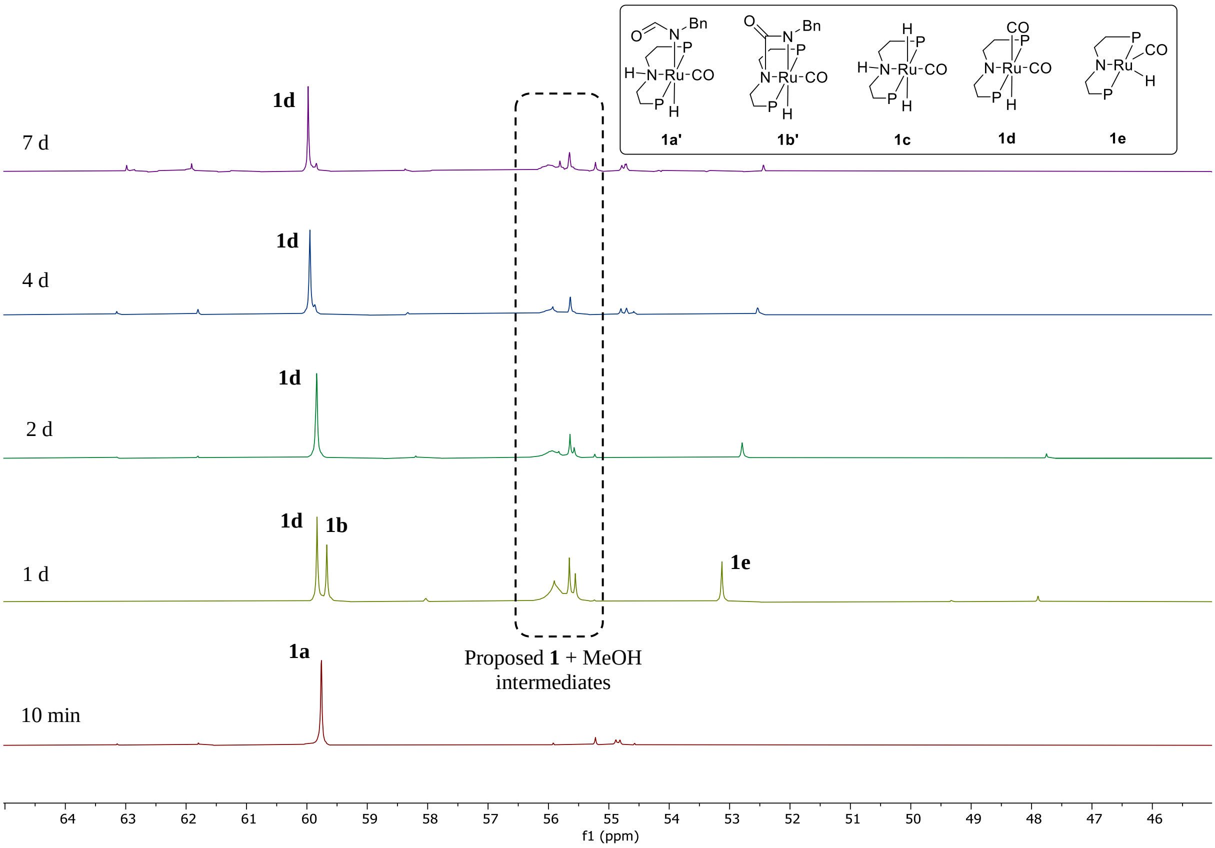

Figure S157: Selected region of the $^{31}P\{^{1}H\}$ NMR spectra (toluene-d_8, 162 MHz, 298 K) showing temporal profile of the reaction of complex **1** + KO^{t}Bu with formanilide at 110 °C (Table S3, where R = Ph).

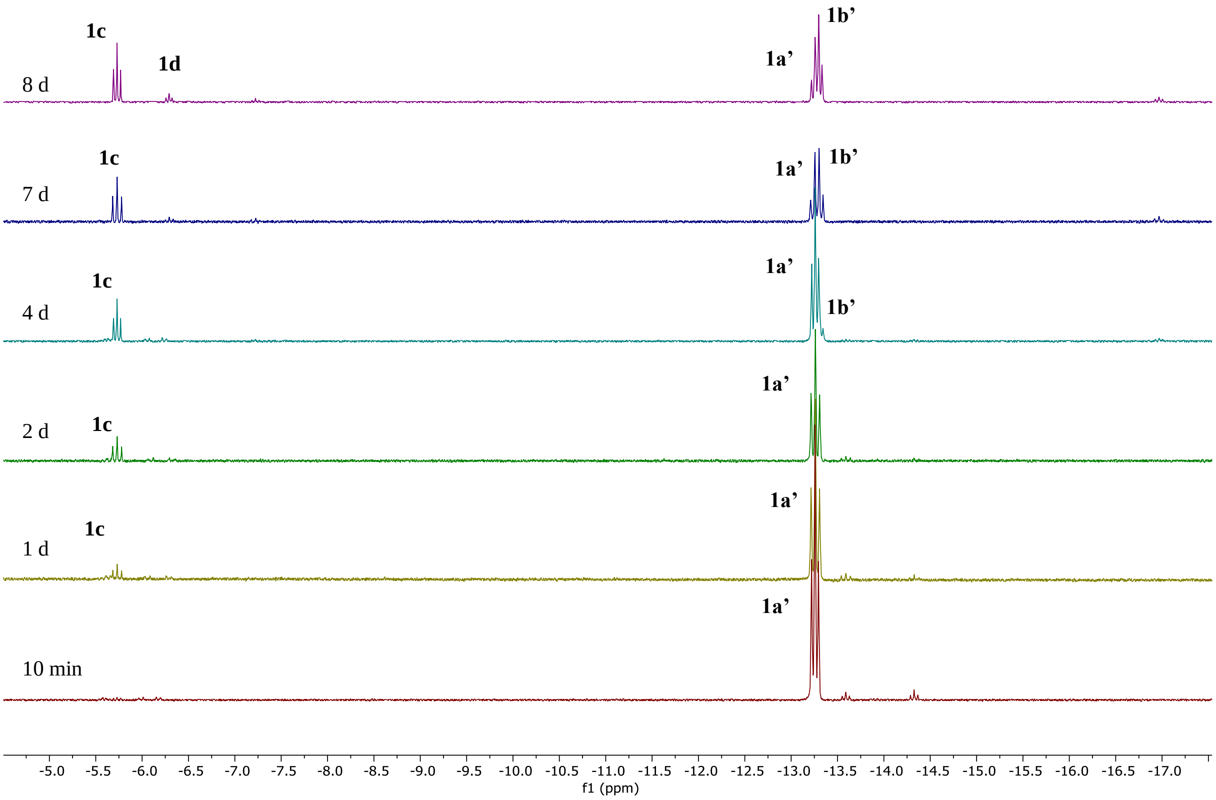

Figure S158: Selected region of the ^{1}H NMR spectra (toluene-d_8, 500 MHz, 298 K) showing temporal profile of the reaction of complex **1** + KO^{t}Bu with N-benzylformamide at 110 °C (Table S3, where R = Bn).

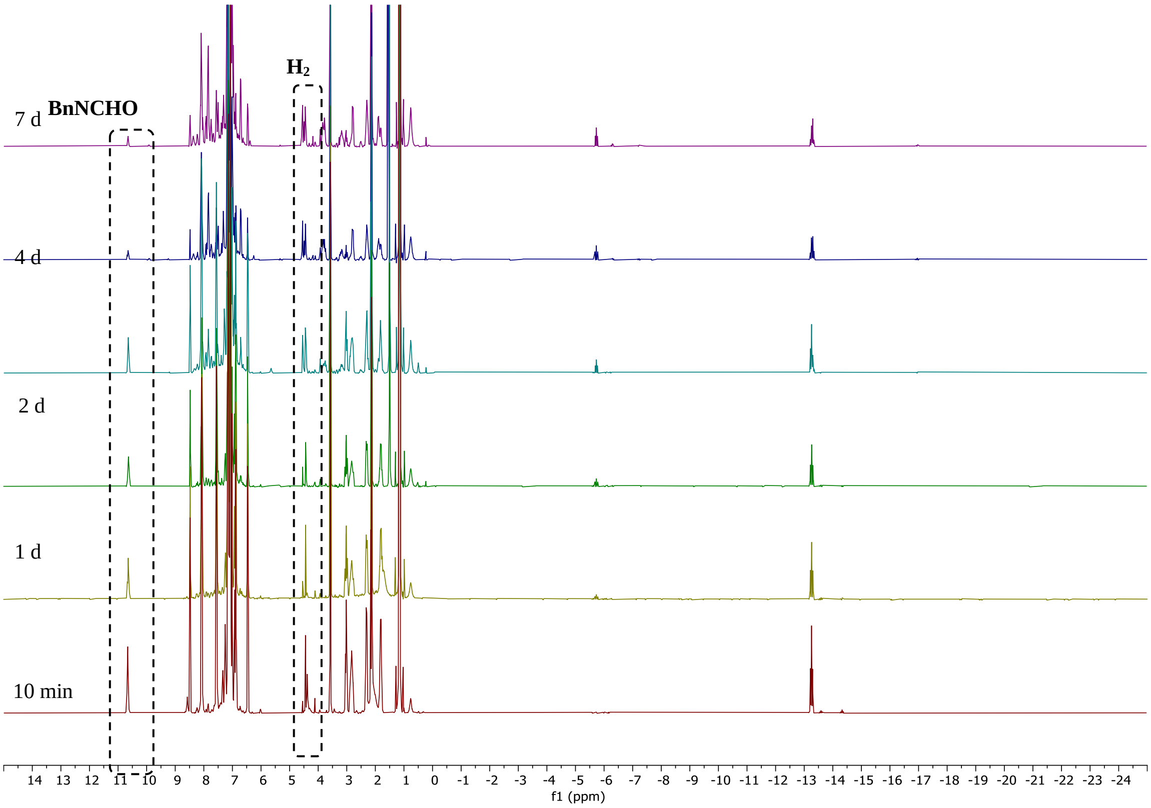

Figure S159: ^{1}H NMR spectra (toluene-d_8, 500 MHz, 298 K) showing temporal profile of the reaction of complex **1** + KO^{t}Bu with N-benzylformamide at 110 °C (Table S3, where R = Bn).

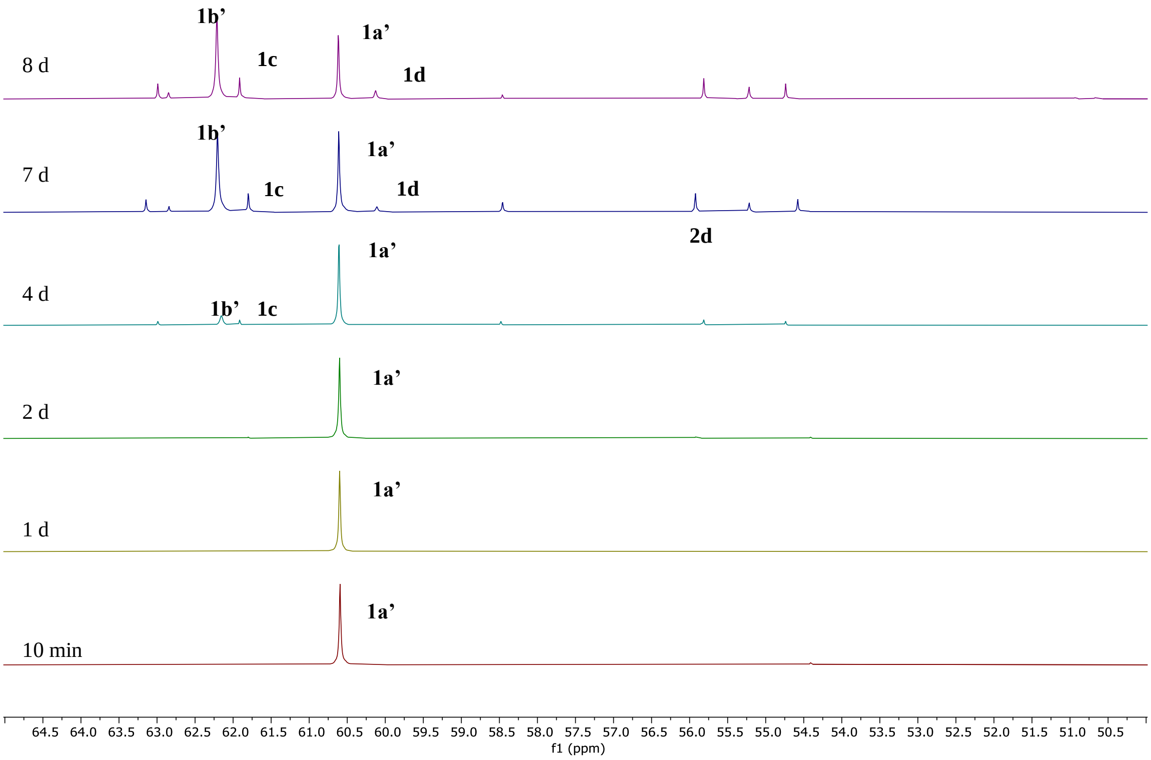

Figure S160: Selected region of the $^{31}P\{^{1}H\}$ NMR spectra (toluene-d_8, 162 MHz, 298 K) showing temporal profile of the reaction of complex **1** + KO^{t}Bu with N-benzylformamide at 110 °C (Table S3, where R = Bn).

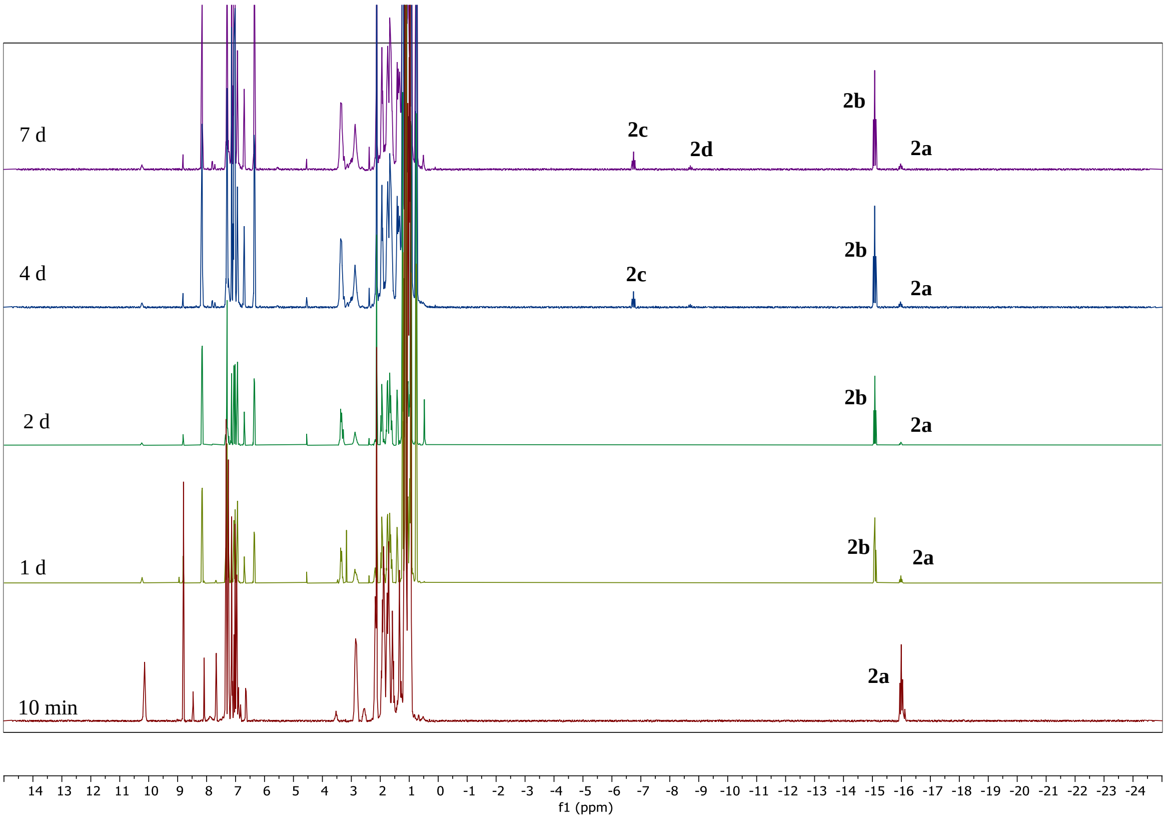

Figure S161: ^{1}H NMR spectra (toluene-d_8, 500 MHz, 298 K) showing temporal profile of the reaction of complex **2** + KO^{t}Bu with formanilide at 110 °C (Table S3, where R = Ph).

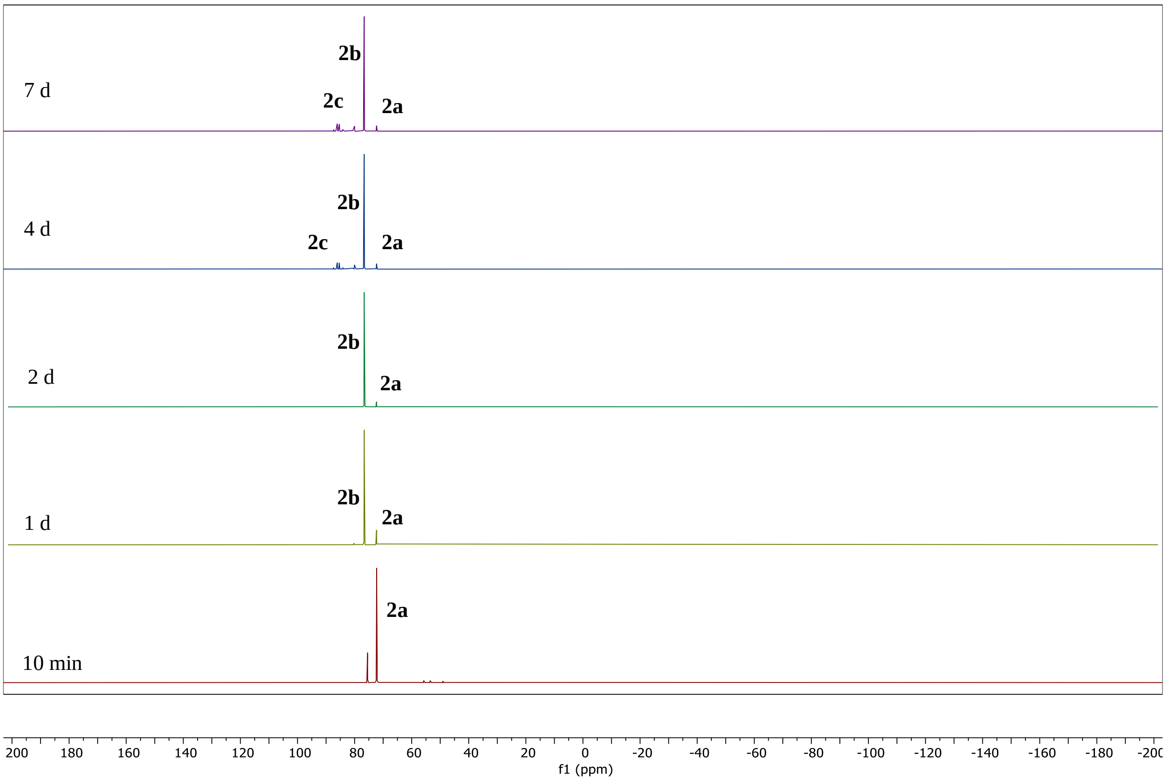

Figure S162: $^{31}P\{^{1}H\}$ NMR spectra (toluene-d_8, 500 MHz, 298 K) showing temporal profile of the reaction of complex **2** + KO^{t}Bu with formanilide at 110 °C (Table S3, where R = Ph).

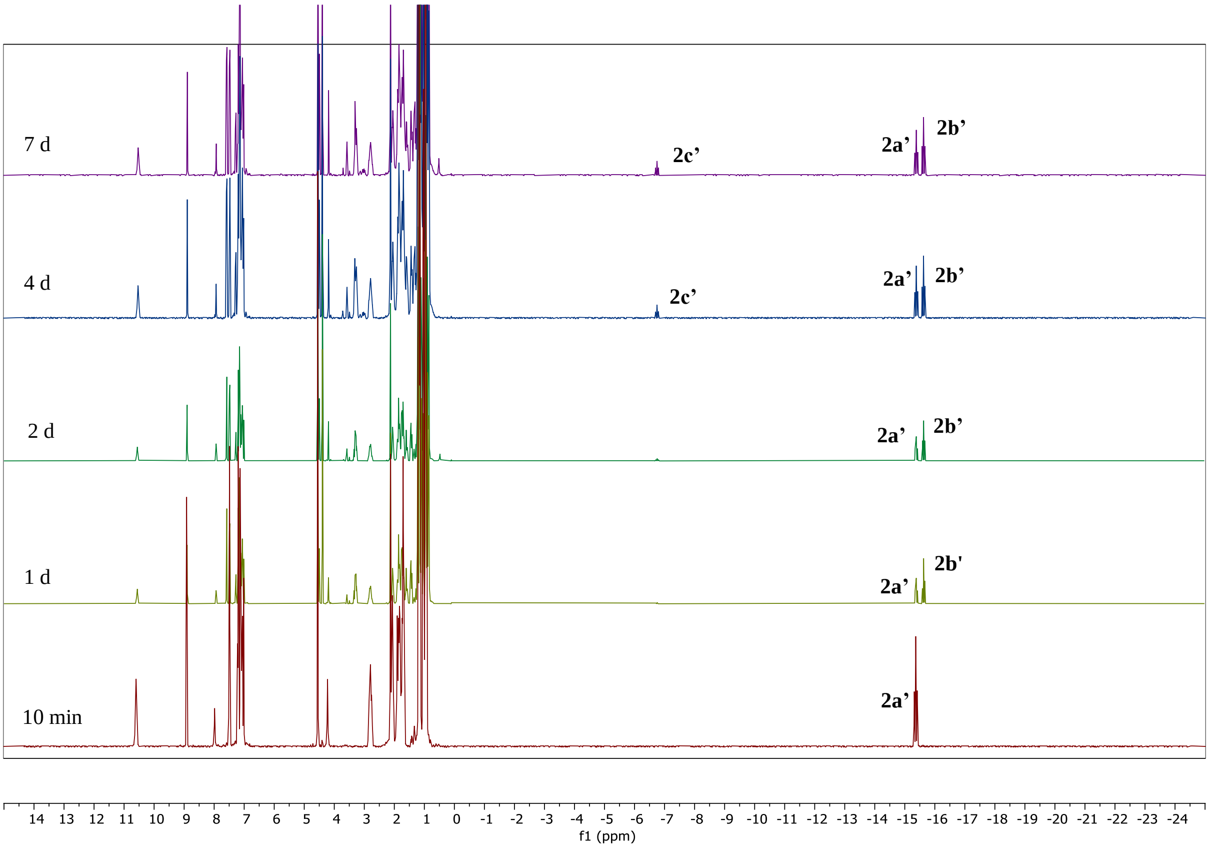

Figure S163: ^{1}H NMR spectra (toluene-d_8, 500 MHz, 298 K) showing temporal profile of the reaction of complex **2** + KO^{t}Bu with N-benzylformamide at 110 °C (Table S3, where R = Bn).

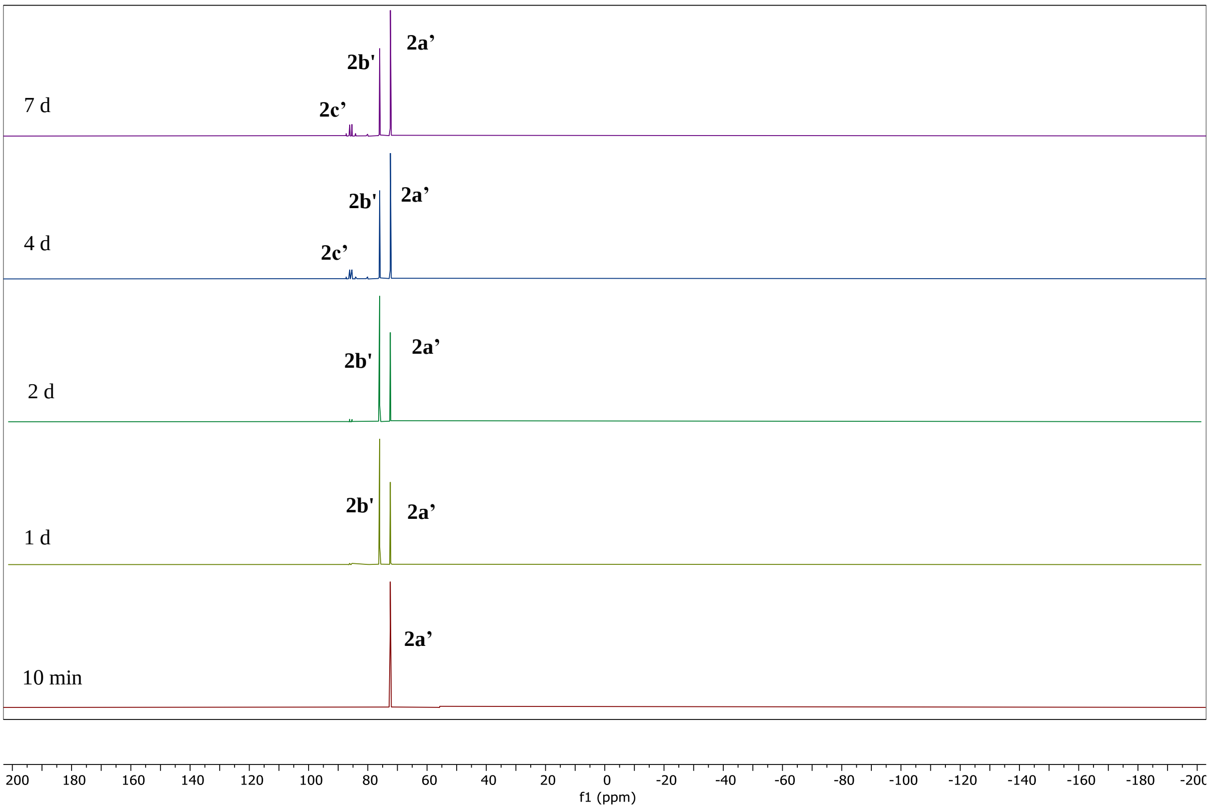

Figure S164: $^{31}P\{^{1}H\}$ NMR spectra (toluene-d_8, 500 MHz, 298 K) showing temporal profile of the reaction of complex **2** + KO^{t}Bu with N-benzylformamide at 110 °C (Table S3, where R = Bn).

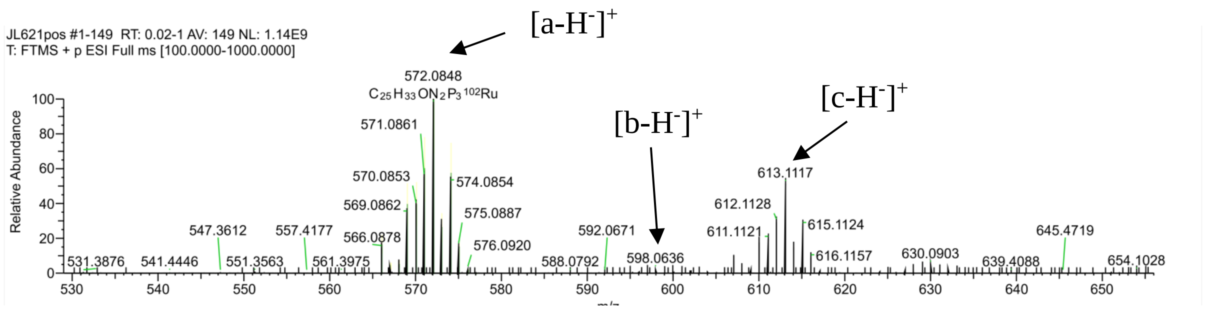

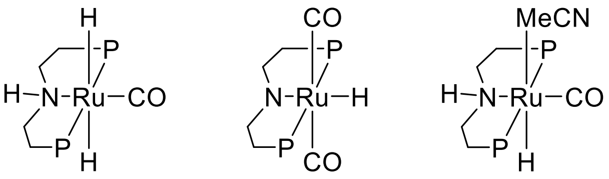

1c 1d 1f

Figure S165: HR-MS (ESI, MeCN, Da) spectrum from reaction mixture of formanilide, complex **1** and KO^{t}Bu in toluene-d_8 (110 °C, 48 h).

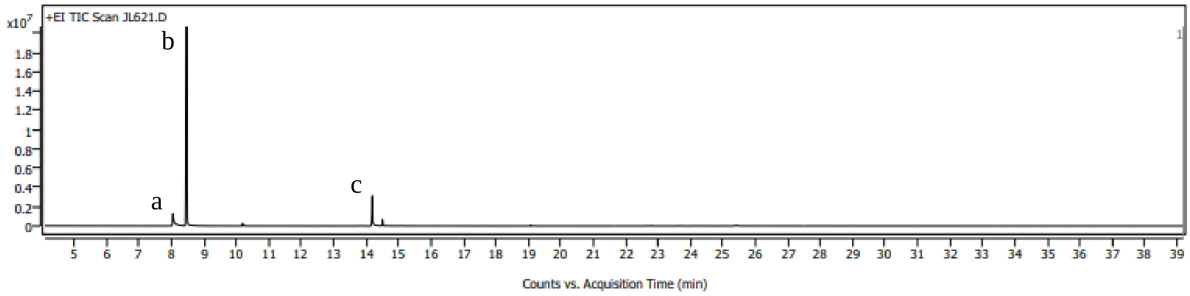

Figure S166: Gas chromatograph of the reaction mixture of complex **1** (0.02 mmol), KO^{t}Bu (0.024 mmol) and formanilide (0.024 mmol) in toluene after being heated at 110 °C for 48 h.

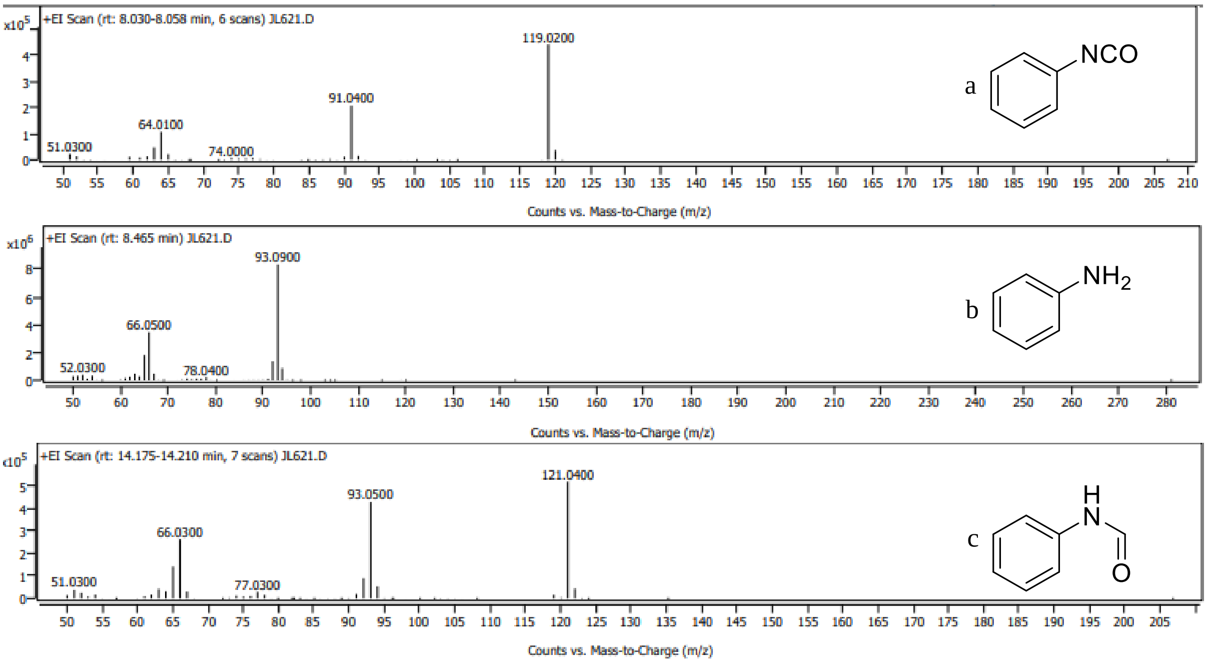

Figure S167: Mass spectra corresponding to gas chromatograph in Figure S142.

4.2. Synthesis of complexes 1a, 1b and 1d

General Procedures

*General Procedure for synthesis of complex **1a** and **1a'**:* A J-Young's NMR tube was charged with complex **1** (12.1 mg, 0.02 mmol), KO^{t}Bu (2.7 mg, 0.024 mmol) and the formanilide or *N*-benzylformamide (0.024 mmol) before being vacuum cycled 3 times with argon. toluene-d_8 (*ca.* 0.5 mL) was added in a glovebox and the NMR tube was shaken vigorously. The mixture was then heated at 60 °C for 24 h. The complexes were characterised by the crude NMR spectra which showed the formation of complexes **1a** and **1a'** in yields >95%. Crystals suitable for single crystal X-ray diffraction were obtained by vapour diffusion (toluene/hexane) under an argon atmosphere.

4.3. Characterisation data for complexes 1a, 1a' and 1d

4.3.1. NMR data

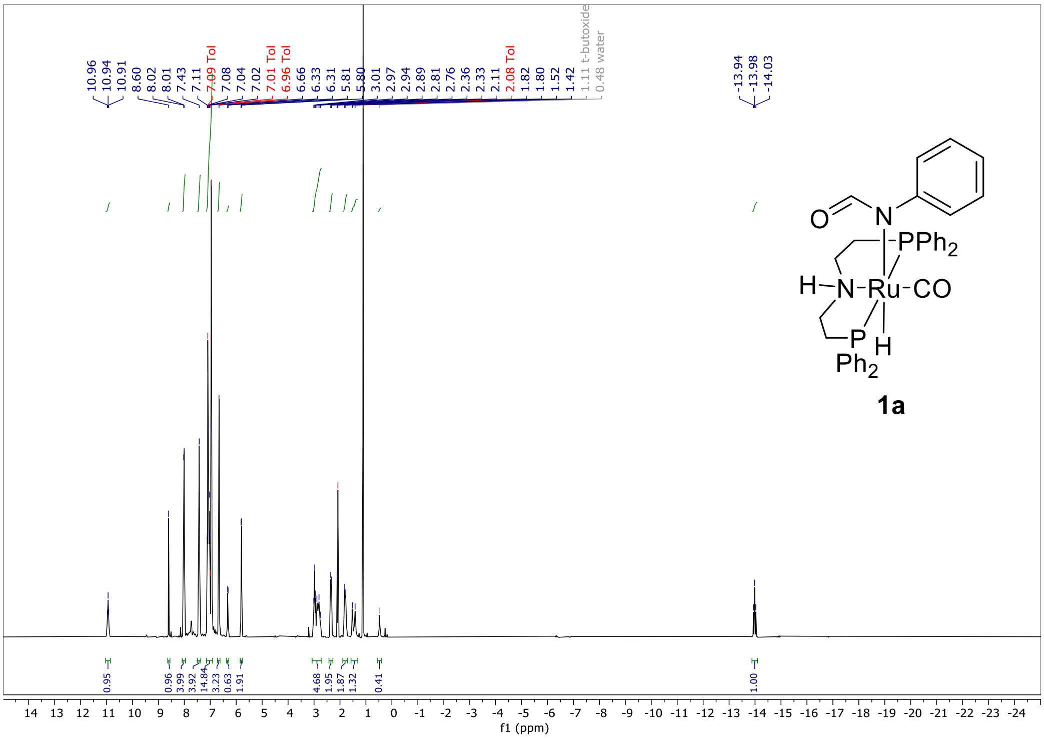

Figure S168: ^{1}H NMR spectrum (toluene-d_8, 500 MHz, 298 K) of complex **1a**.

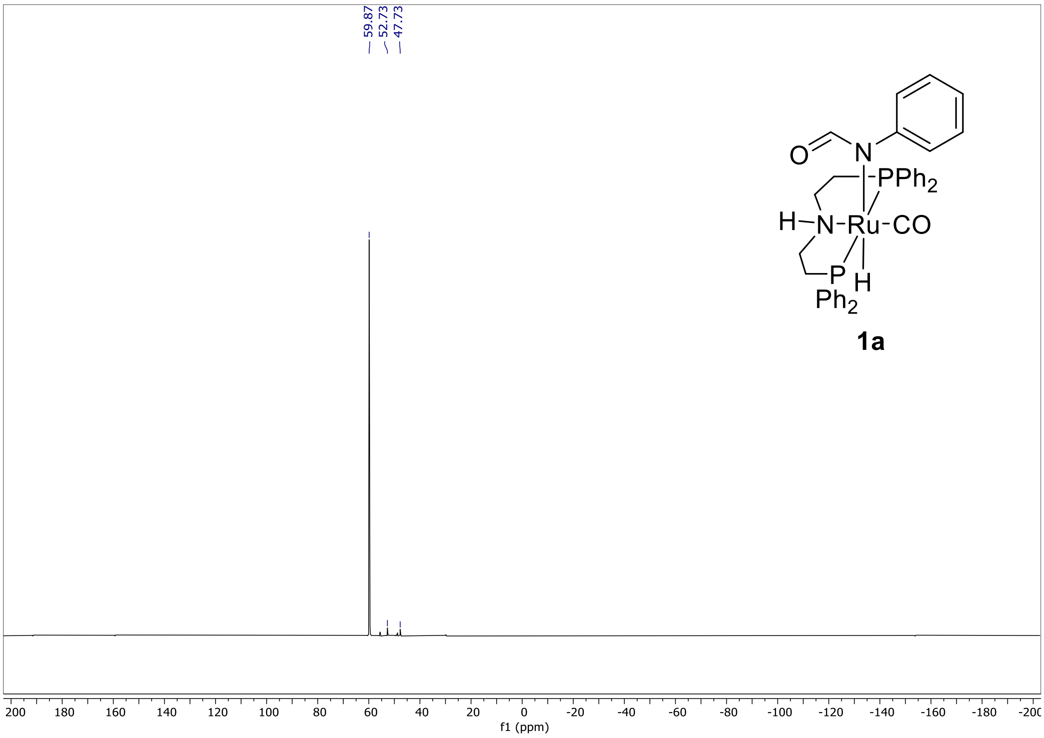

Figure S169: $^{31}P\{^{1}H\}$ NMR spectrum (toluene-d_8, 162 MHz, 298 K) of complex **1a**.

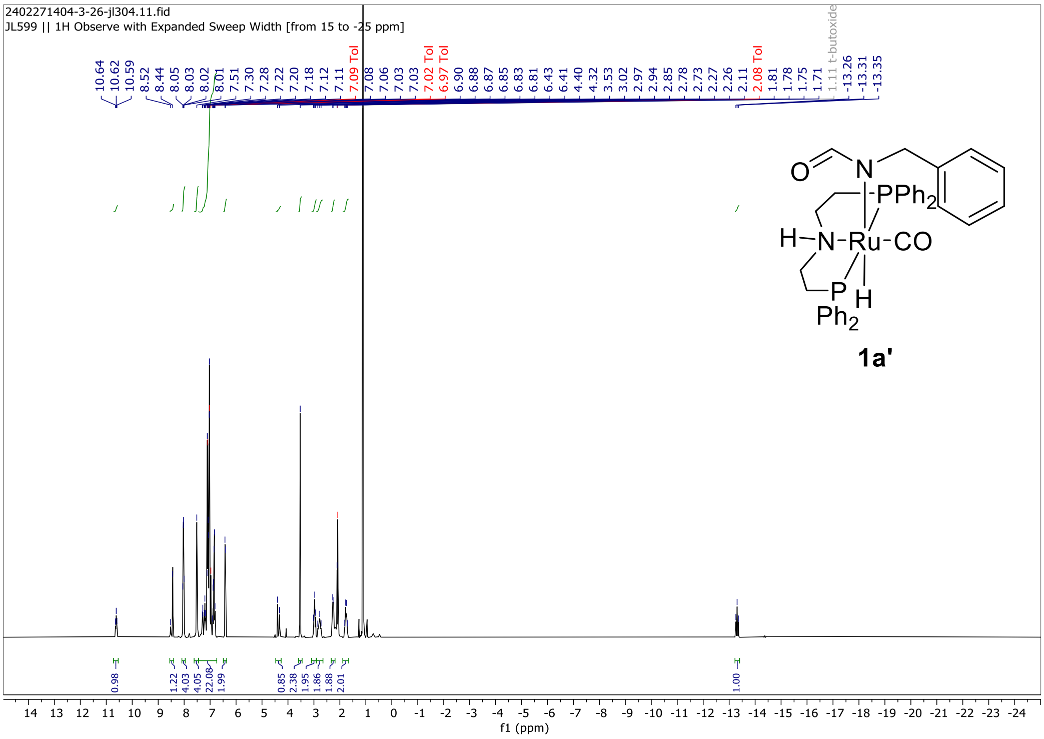

Figure S170: ^{1}H NMR spectrum (toluene-d_8, 500 MHz, 298 K) of complex **1a'**.

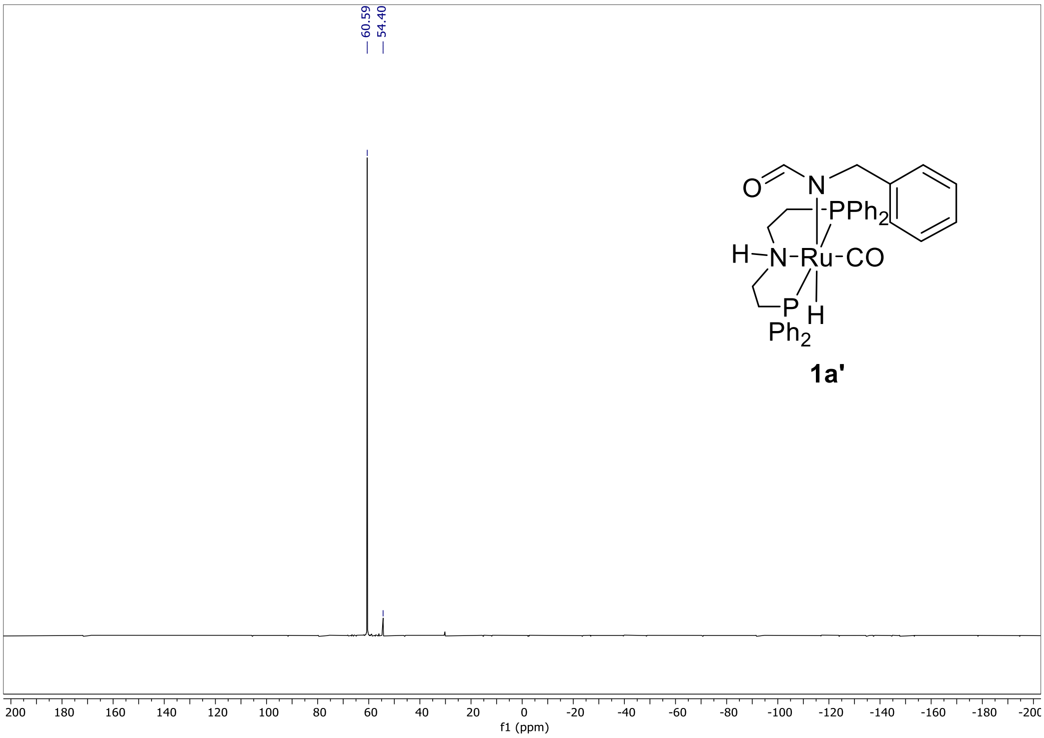

Figure S171: ^{31}P{^{1}H} NMR spectrum (toluene-d_8, 162 MHz, 298 K) of complex **1a'**.

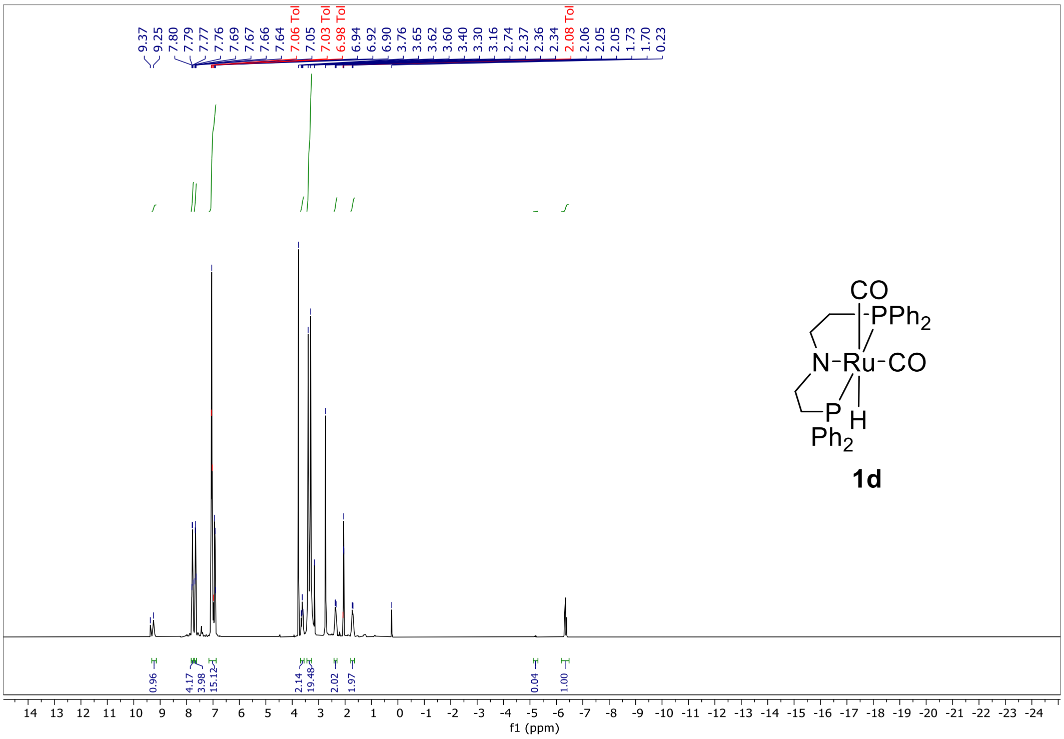

Figure S172: ^{1}H NMR spectrum (toluene-d_8, 500 MHz, 298 K) of complex **1d** corresponding to Figure 2B.

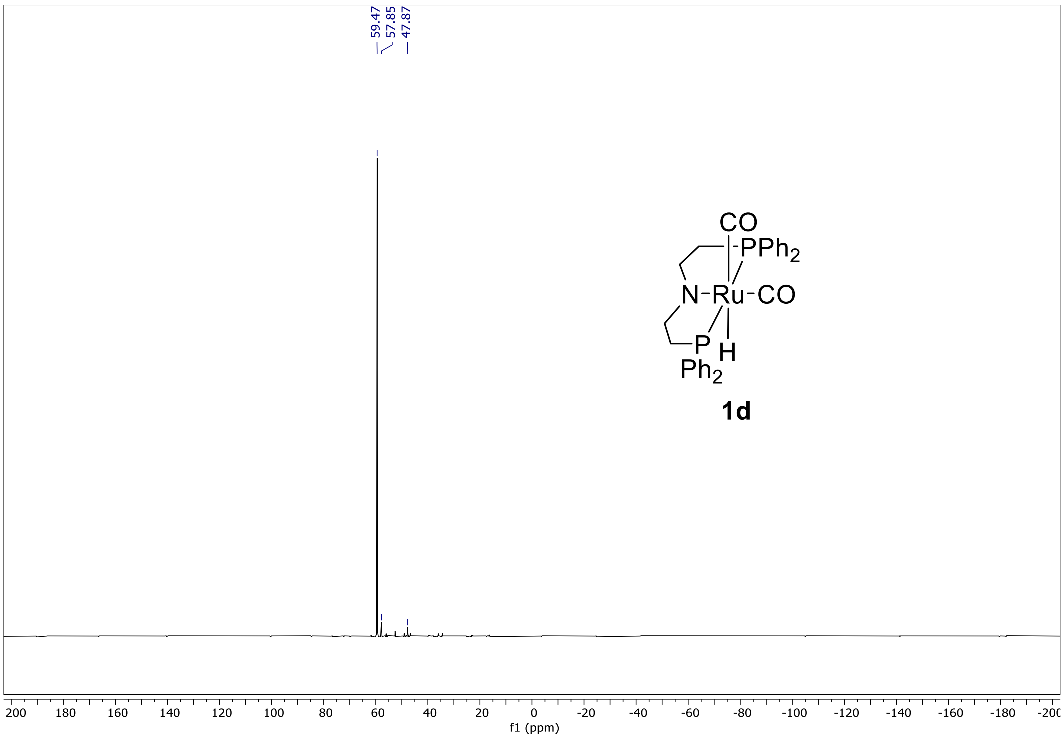

Figure S173: $^{31}P\{^{1}H\}$ NMR spectrum (toluene-d_8, 162 MHz, 298 K) of complex **1d** corresponding to Figure 2B.

4.3.2. Crystallographic data

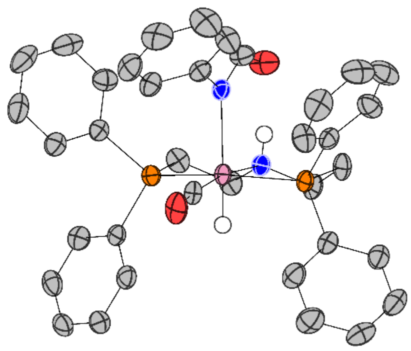

Figure S174: Single-crystal X-ray structure of complex **1a**. ORTEP thermal ellipsoid plots plotted at 50% probability level. Selected hydrogen atoms have been omitted for clarity.

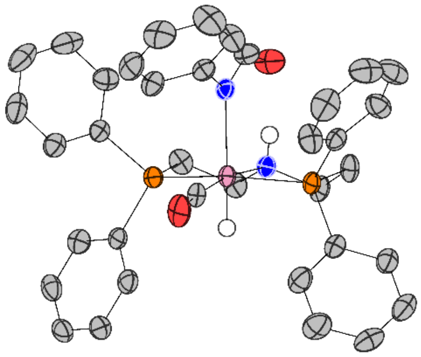

Figure S175: Single-crystal X-ray structure of complex **1a’**. ORTEP thermal ellipsoid plots plotted at 50% probability level. Selected hydrogen atoms have been omitted for clarity.

Table S4: Crystallographic data for complexes **1A** and **1A’**.

**Compound reference**	**1A**	**1A’**
Chemical formula	$C_{36}H_{36}N_2O_2P_2Ru$	$C_{45}H_{47}N_3O_3P_2Ru$
CCDC deposition number	2356150	2356158
Formula weight	691.68	840.86
Temperature (K)	173.15	173.15
Crystal system	Triclinic	Triclinic
Space group	P-1	P-1
a (Å)	9.3455(3)	10.3385(2)
b (Å)	12.3580(3)	12.9630(2)
c (Å)	14.8848(3)	15.3995(4)
α (°)	108.452(2)	85.247(2)
β (°)	92.030(2)	80.723(2)
γ (°)	97.599(2)	85.758(10)
Unit cell volume (Å^3)	1610.90(8)	2025.95(7)
Z	2	2
ρ_{calc}g (cm^3)	1.426	1.378
μ (mm^{-1})	0.621	0.510
F(000)	712.0	872.0
Radiation	Mo Kα (λ = 0.71073)	Mo Kα (λ = 0.71073)
2Θ range for data collection/°	3.514 to 59.034	3.158 to 58.912
Index ranges	$-12 \leq h \leq 12$, $-16 \leq k \leq 16$, $-18 \leq l \leq 19$	$-13 \leq h \leq 13$, $-17 \leq k \leq 16$, $-20 \leq l \leq 21$

Reflections collected	35066	44155
Independent reflections	7555 [R_{int} = 0.0565, R_{sigma} = 0.0781]	9459 [R_{int} = 0.0523, R_{sigma} = 0.0647]
Data/restraints/parameters	7555/0/392	9459/0/491
Goodness-of-fit on F^2	1.028	1.015
Final R indexes [I>=2σ (I)]	R_1 = 0.0523, wR_2 = 0.0942	R_1 = 0.0406, wR_2 = 0.0751
Final R indexes [all data]	R_1 = 0.0850, wR_2 = 0.1016	R_1 = 0.0652, wR_2 = 0.0801
Largest diff. peak/hole (e Å^{-3})	1.35/-0.57	0.71/-0.57

4.4. Control Experiments

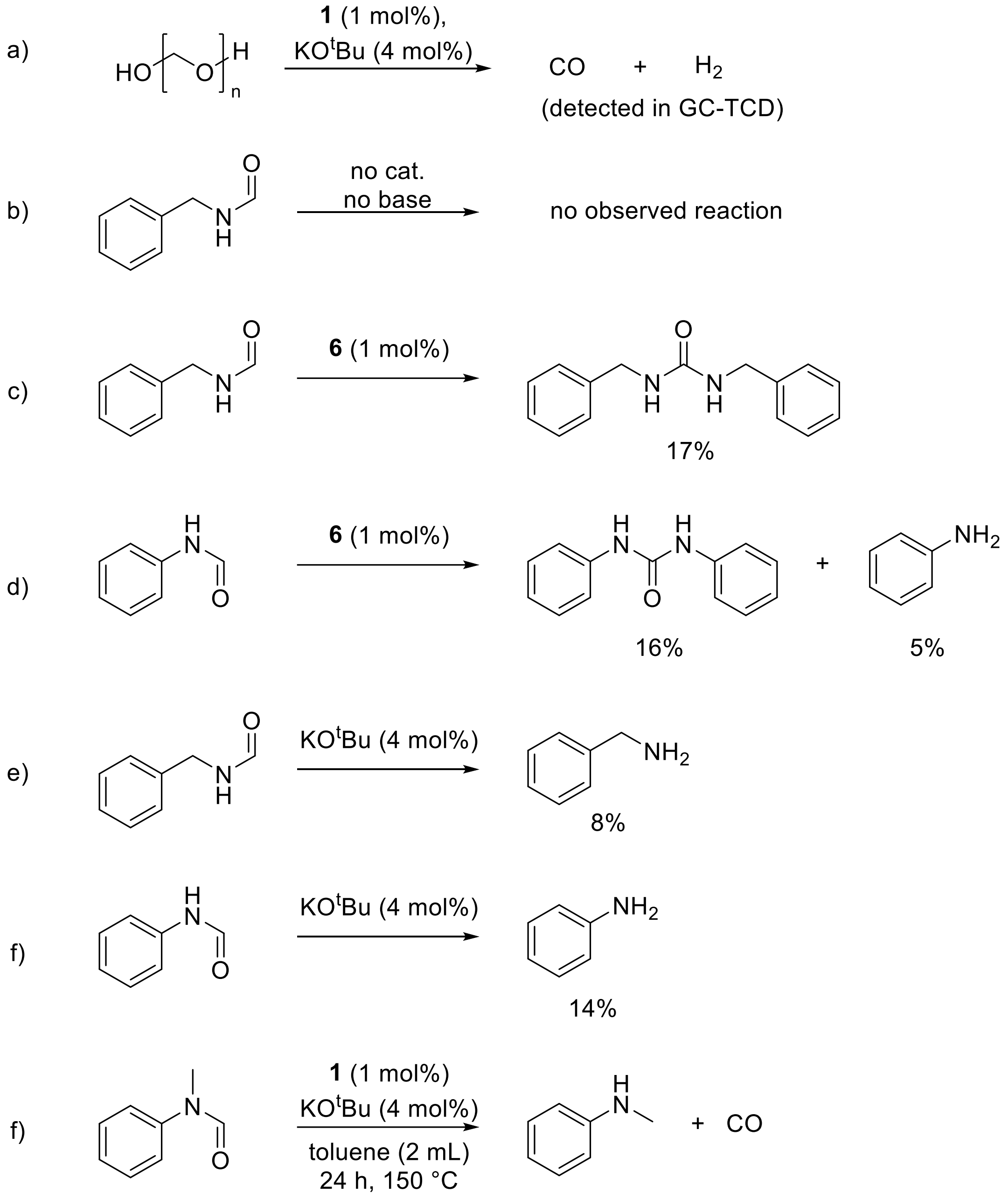

Figure S176: Control experiments (a-f) carried out to develop mechanistic understanding. All reactions were carried out at 150 °C for 24 h and in toluene (2 mL). Yields for reactions b-f were analysed by the addition of 1,3,5-trimethoxybenzene (0.33 mmol) to the reaction mixture as an internal standard. Complex **5** is Ru-MACHO-BH complex (see Table 1 in the manuscript).

4.5. Characterisation data for control experiments

4.5.1. ^{1}H NMR data

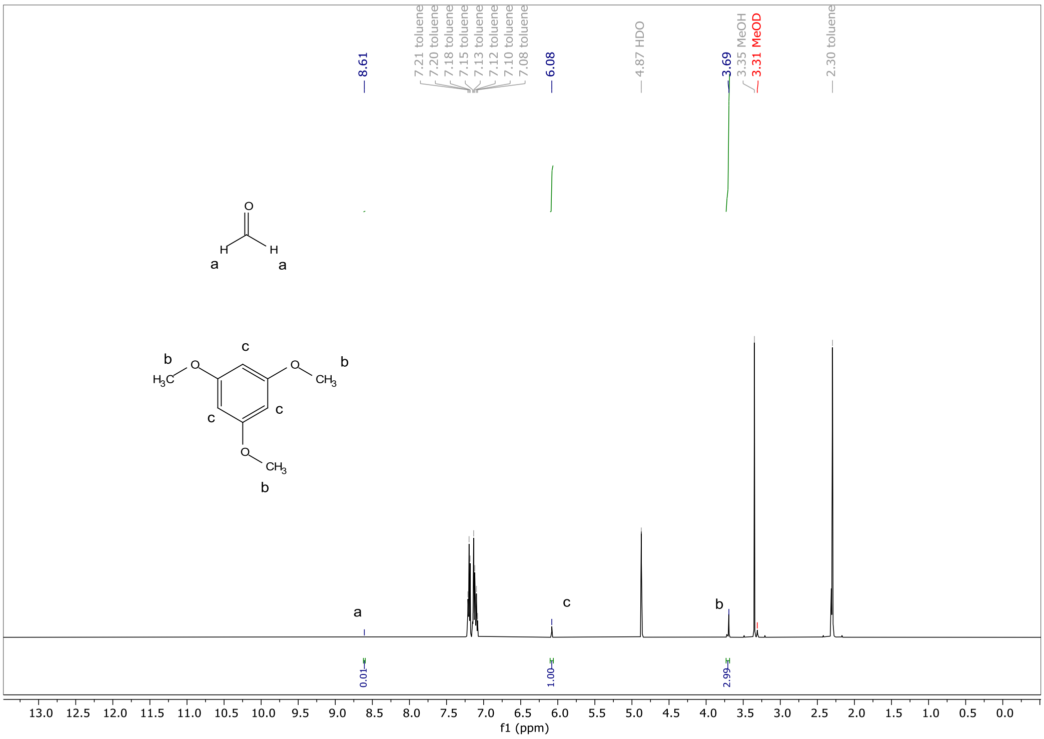

Figure S177: ^{1}H NMR (CD_3OD, 500 MHz, 298 K) of the reaction mixture from control experiment "a" (Section 4.4. a).

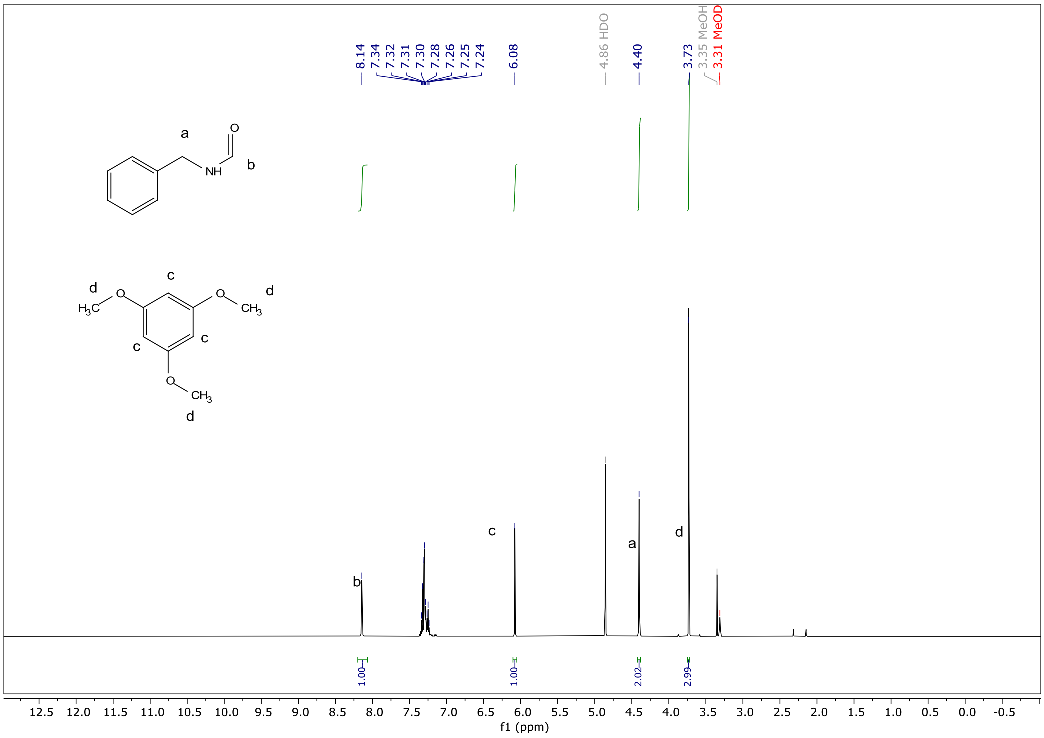

Figure S178: ^{1}H NMR (CD_3OD, 500 MHz, 298 K) of the reaction mixture from control experiment "b" (Section 4.4. b).

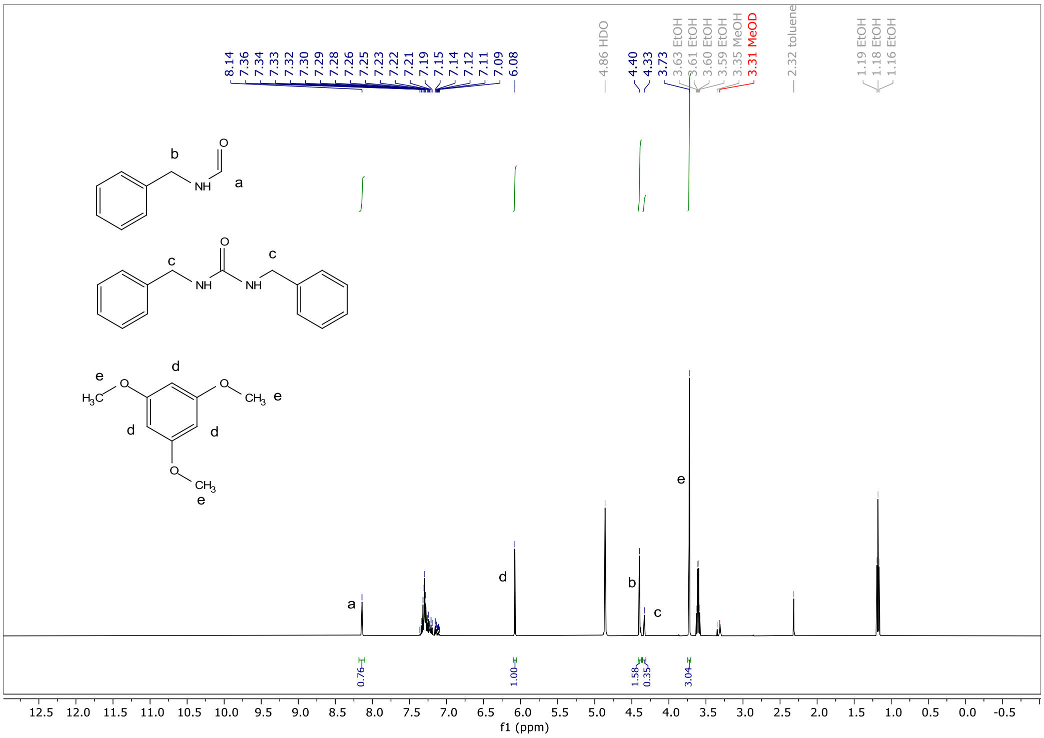

Figure S179: ^{1}H NMR (CD_3OD, 500 MHz, 298 K) of the reaction mixture from control experiment "c" (Section 4.4. c).

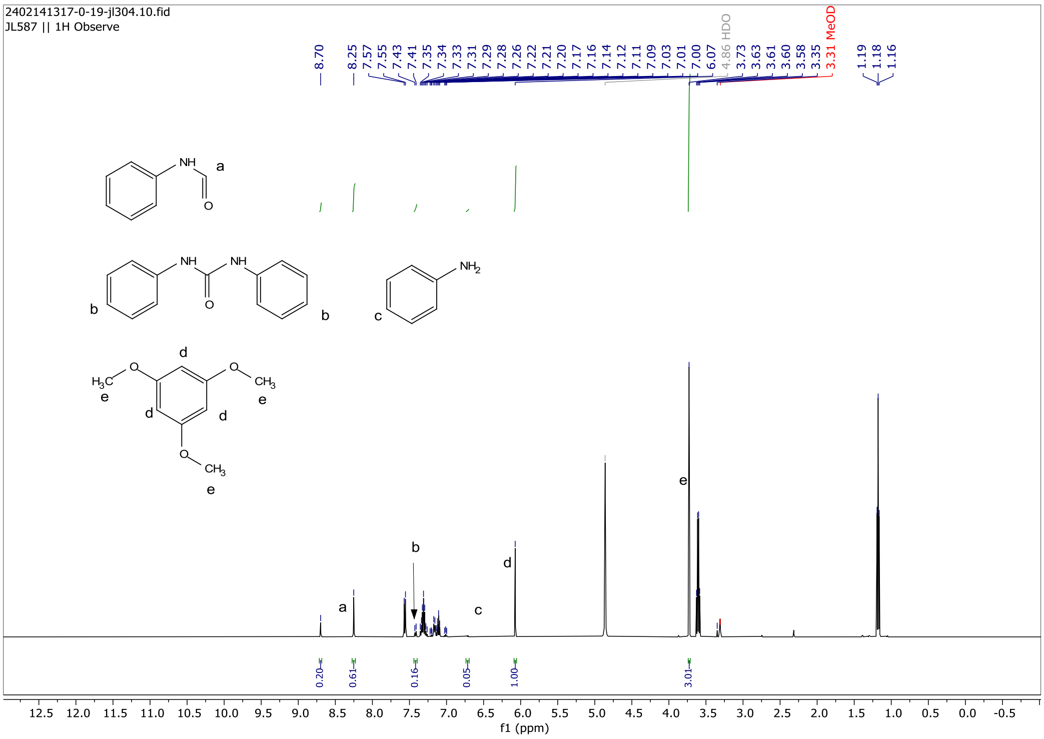

Figure S180: ^{1}H NMR (CD_3OD, 500 MHz, 298 K) of the reaction mixture from control experiment "d" (Section 4.4. d).

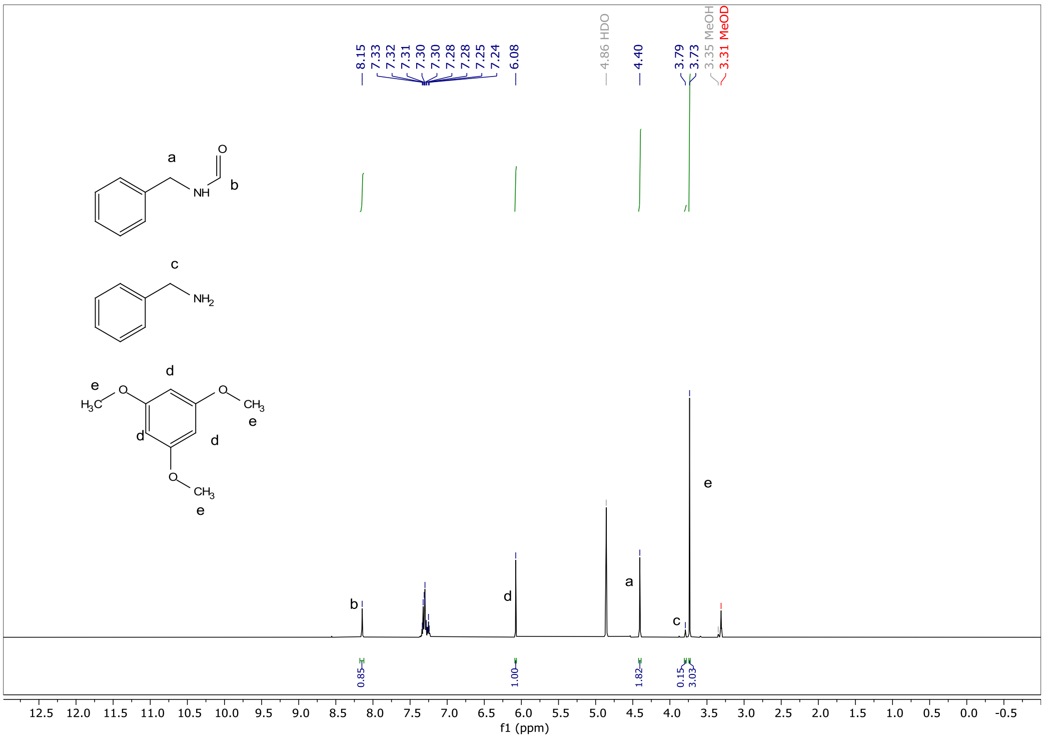

Figure S181: ^{1}H NMR (CD_3OD, 500 MHz, 298 K) of the reaction mixture from control experiment “e” (Section 4.4. e).

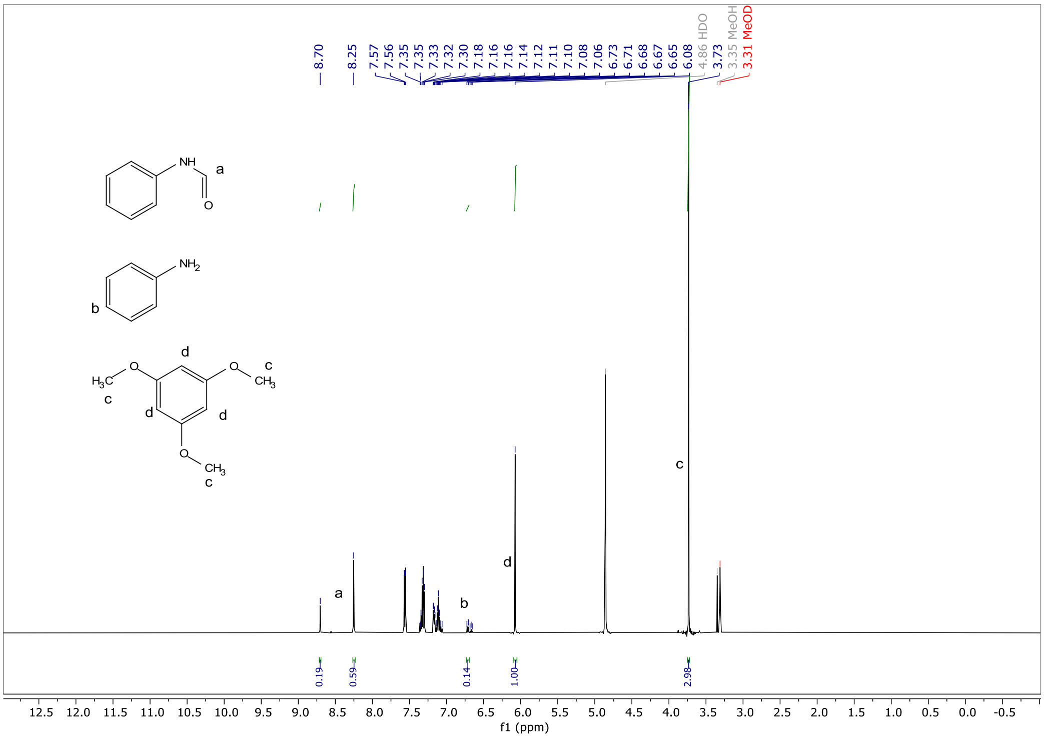

Figure S182: ^{1}H NMR (CD_3OD, 500 MHz, 298 K) of the reaction mixture from control experiment “f” (Section 4.4. f).

4.5.2. GCMS data

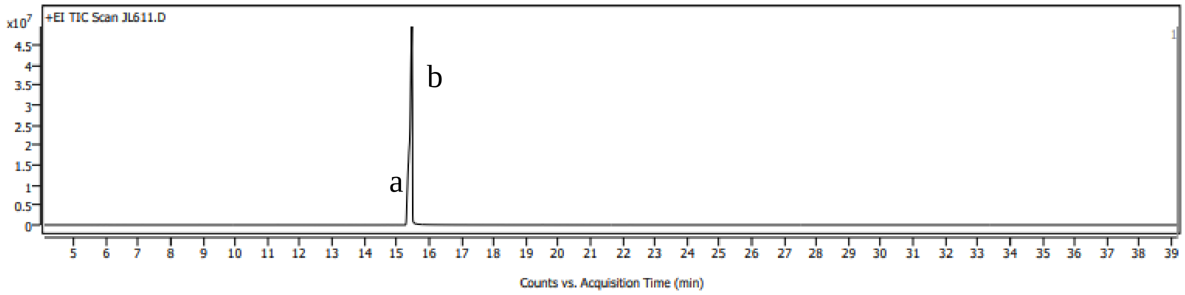

Figure S183: Gas chromatogram of reaction mixture from control reaction “b” (Section 4.4. b).

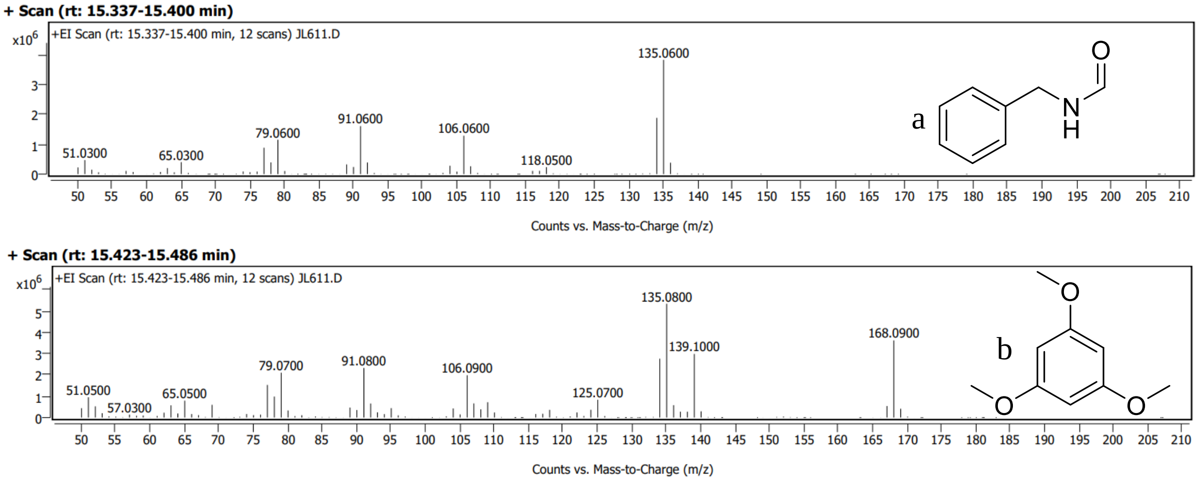

Figure S184: Mass spectra corresponding to gas chromatogram of reaction mixture from control reaction “b” (Section 4.4. b).

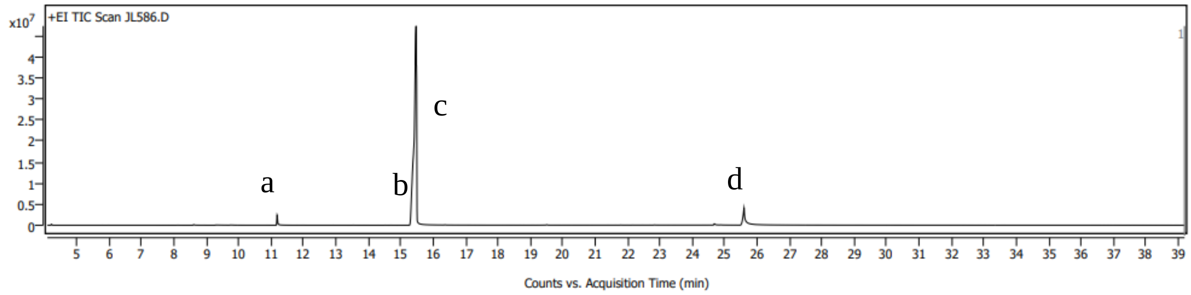

Figure S185: Gas chromatogram of reaction mixture from control experiment “c” (Section 4.4. c).

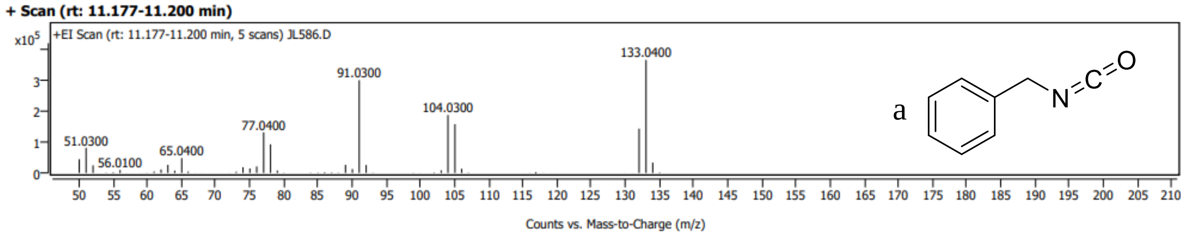

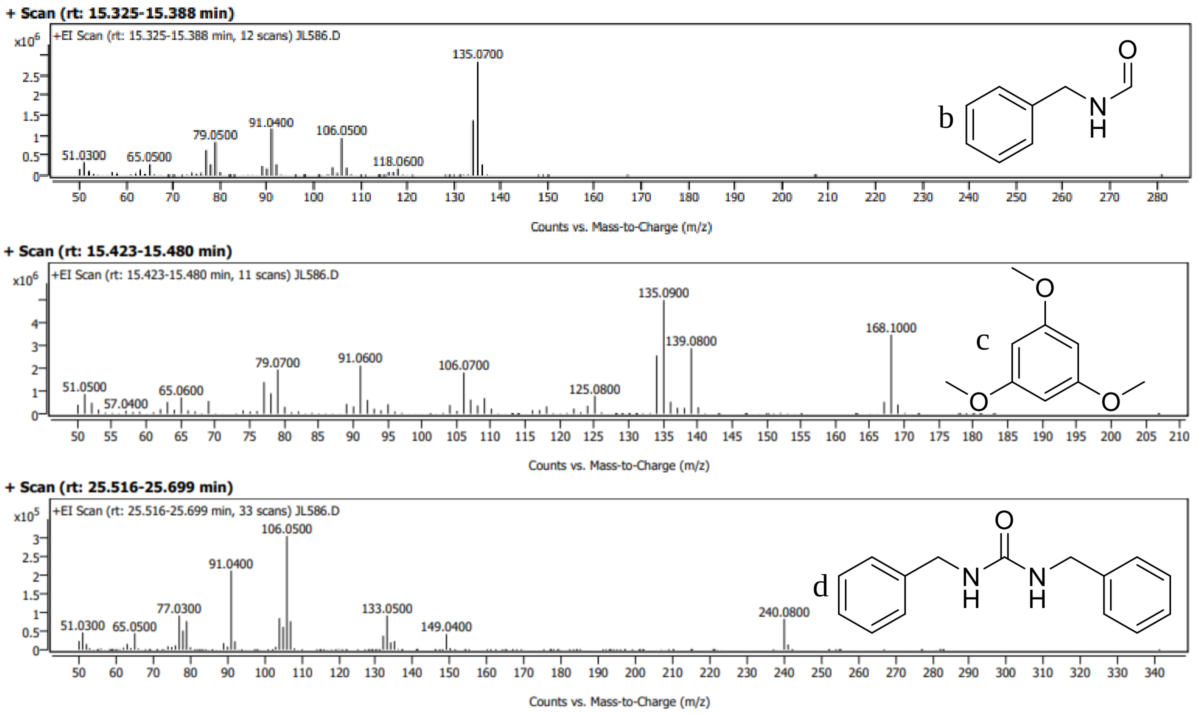

Figure S186: Mass spectra corresponding to gas chromatogram of reaction mixture from control experiment “c” (Section 4.4. c).

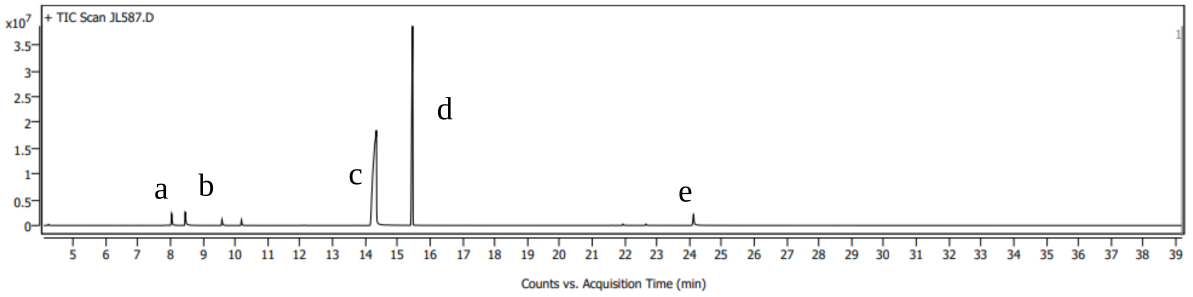

Figure S187: Gas chromatogram of reaction mixture from control experiment “d” (Section 4.4. d).

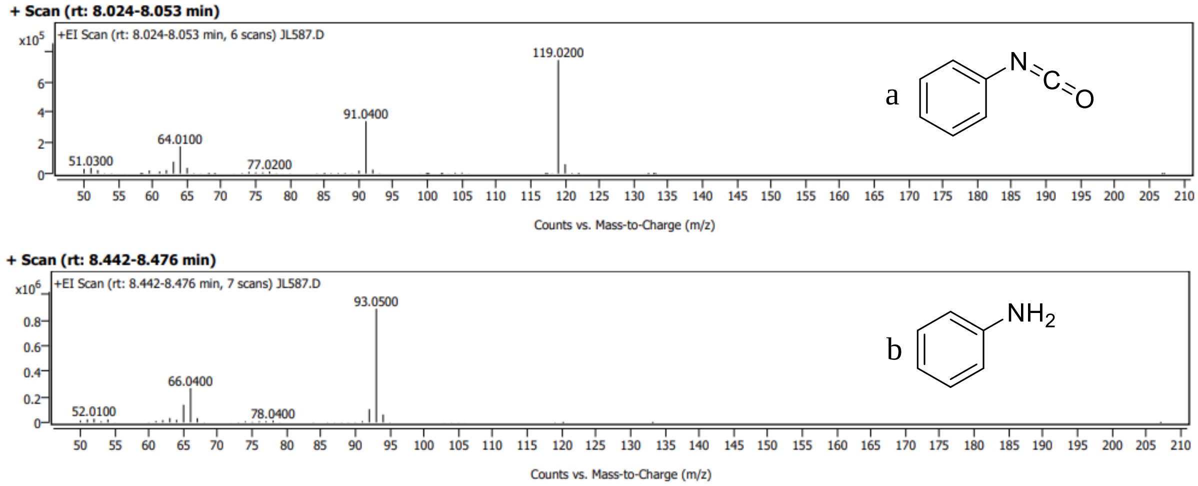

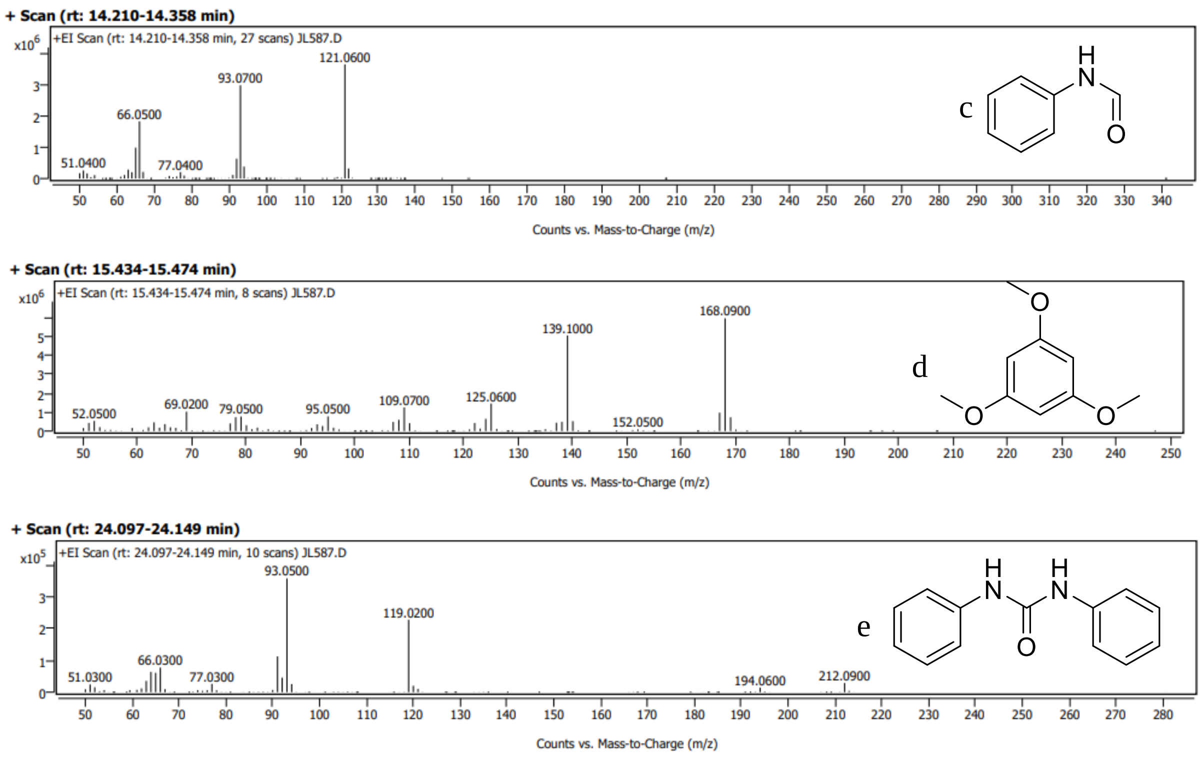

Figure S188: Mass spectra corresponding to gas chromatogram of reaction mixture from control experiment “d” (Section 4.4. d).

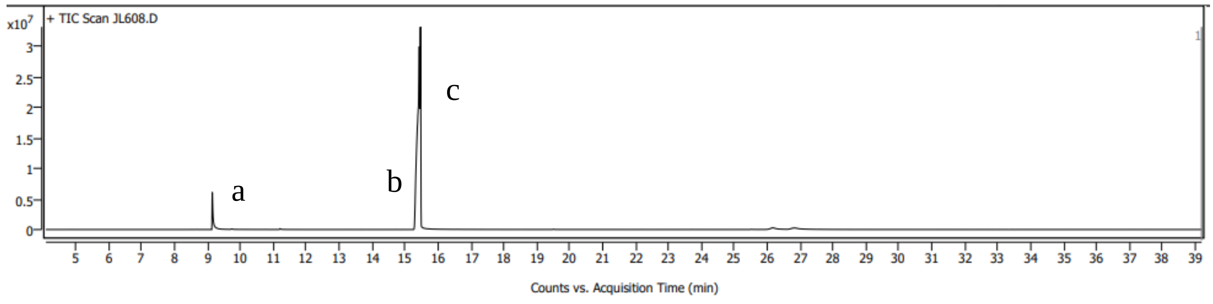

Figure S189: Gas chromatogram of reaction mixture from control experiment “e” (Section 4.4. e).

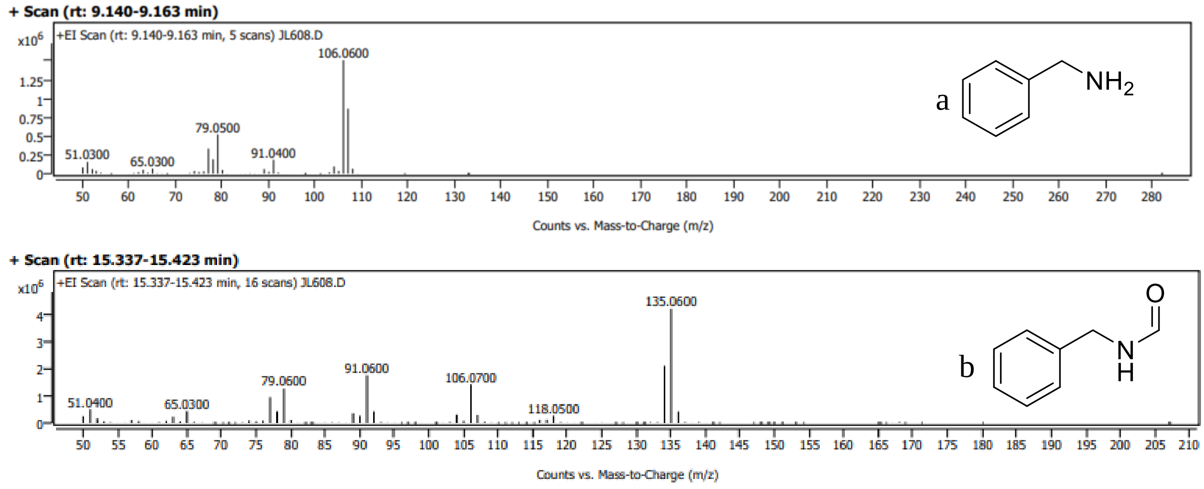

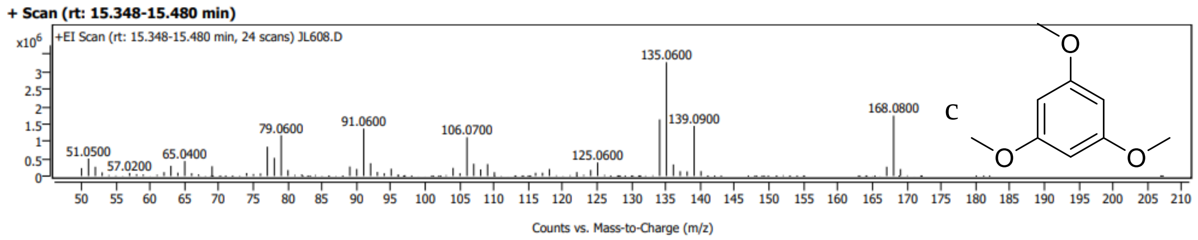

Figure S190: Mass spectra corresponding to gas chromatogram of reaction mixture from control experiment "e" (Section 4.4. e).

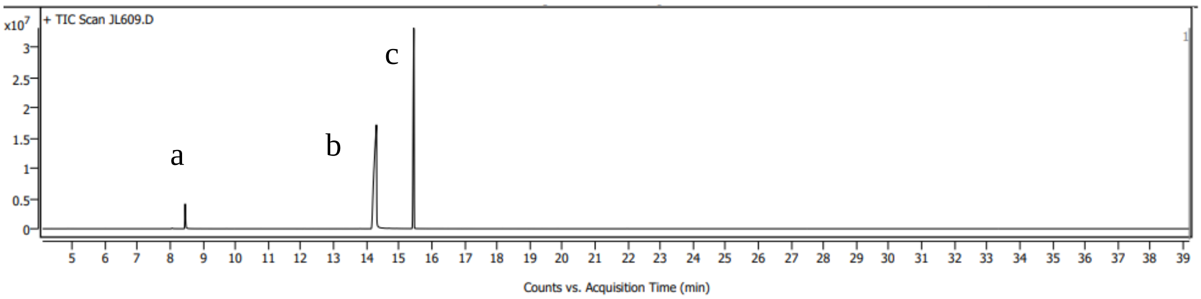

Figure S191: Gas chromatogram of reaction mixture from control experiment "f" (Section 4.4. f).

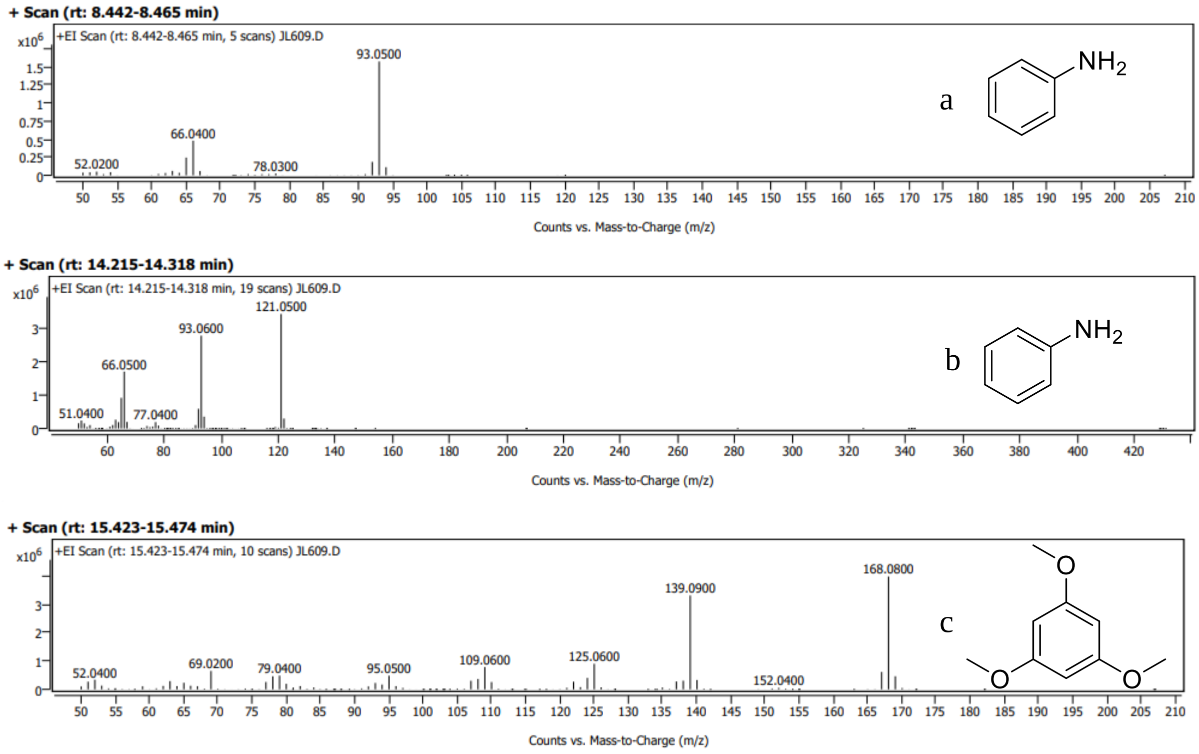

Figure S192: Mass spectra corresponding to gas chromatogram of reaction mixture from control experiment "f" (Section 4.4. f).

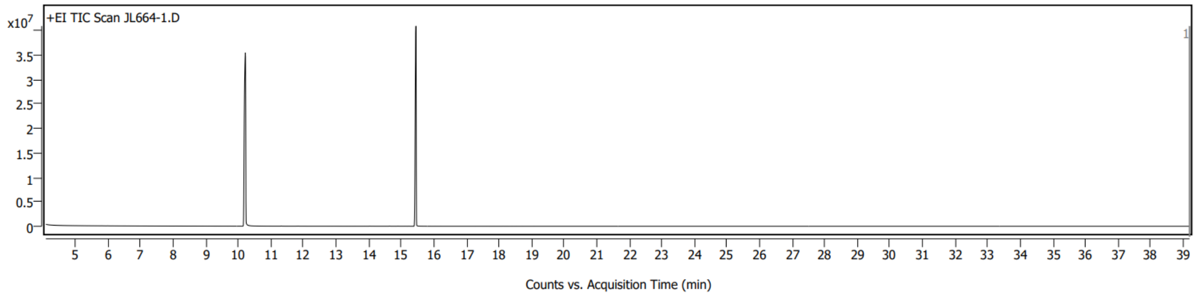

Figure S 193: Gas chromatogram of reaction mixture from control experiment "g" (Section 4.4. g and Scheme 2B in manuscript).

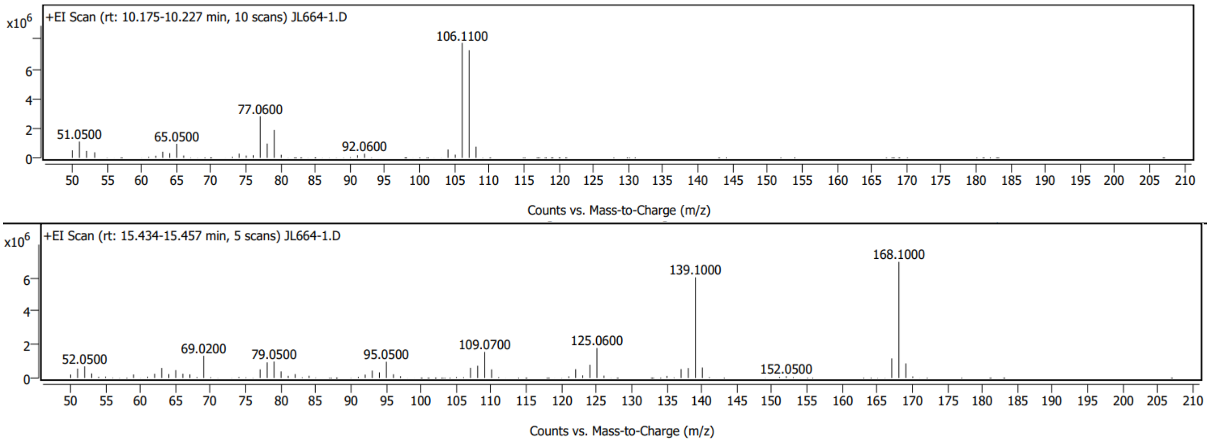

Figure S194: Mass spectra corresponding to gas chromatogram of reaction mixture from control experiment "g" (Section 4.4. g and Scheme 2B in manuscript).

4.5.3. GCTCD data

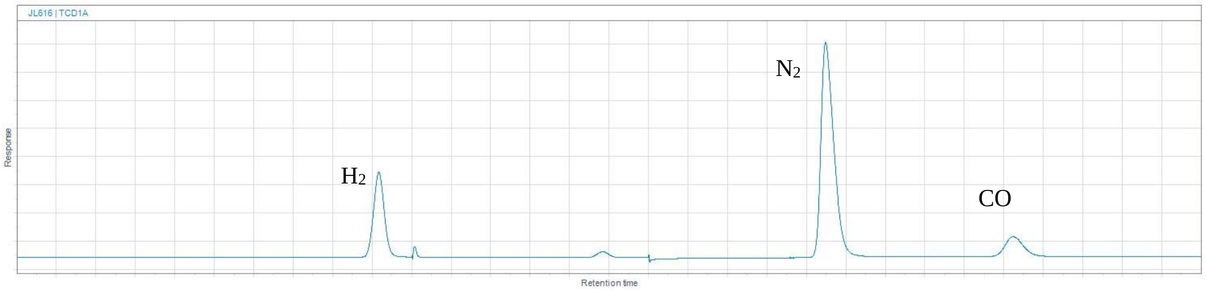

Figure S195: GC-TCD spectrum for the dehydrogenation of paraformaldehyde control reaction "a" (section 4.4. a).

5. Computational Details

5.1. General Considerations

DFT calculations were performed using the PBE0-D3(BJ)$_{PCM(THF)}$/def2-TZVP//RI-BP86$_{PCM(THF)}$/def2-SVP level of theory with Gaussian16, C.01.[9] This is the same level as used in previous work from the group,[10-14] benchmarked against a series of experimental 3d transition metal hydride bond strengths[15] and including Martin, Hay and Pratt empirical entropy corrections[16] (*i.e.* 5.09 kcal mol^{-1} per particle[10]). As noted previously, the choice of

solvent between THF (consistent with previous work) and toluene (used experimentally) has little impact upon the overall thermodynamics and for continuity we retain THF. Ru was described using the Stuttgart-Dresden relativistic small-core pseudopotential (SDD, as implemented in Gaussian 16)[17] both at the level of geometry optimisations and single point energy calculations. The stereochemistry and conformation of the metal fragment was taken as that observed in the X-ray structures, notably with the CO ligand of the [Ru](H)(CO) moiety trans to the MACHO-N atom, and the hydride trans to the bound substrates.

The good accord between the DFT and X-ray structures of **1A** are shown in an overlay of both (Figure S170) and the comparison of key distances (Table S3).

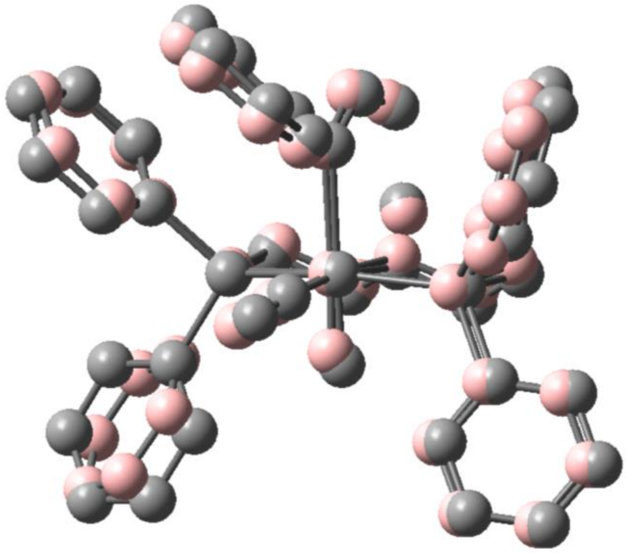

Figure S196: Overlay of structures of **1A** from X-ray crystallography (pink) and from BP86 optimisation (grey), selected H atoms omitted.

Table S5: Key metal-ligand distances from DFT optimisation (RI-BP86$_{PCM(THF)}$/def2-SVP level) and from X-ray crystallography of complex **1A** (in Å).

Distance	DFT	X-ray
Ru-C(O)	1.830	1.834(3)
Ru-N(macho)	2.229	2.188(2)
Ru-N(NPF)	2.267	2.256(3)
Ru-P	2.338/2.341	2.303(1)/2.322(1)
N(macho)-O(NPF)	2.684	2.720(4)
Ru-H	1.611	1.38(4)[a]

[a]Note that bond distances to H atoms tend to be underestimated in X-ray crystallography. NPF stands for N-phenyl formamide or formanilide.

5.2. Driving forces of catalysed reactions

Table S6: Computed free energies ΔG^{432K} (in parentheses: enthalpies ΔH^{432K}) for reactions under scrutiny (kcal mol^{-1}); all-syn conformations except where otherwise noted.

Reaction	R	ΔG^{432K}	(ΔH^{432K})
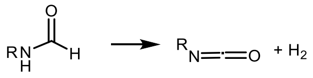 (1)	Me	16.4	(24.7)
	Ph	15.1	(23.2)
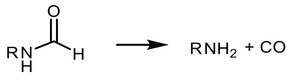 (2)	Me	17.0	(26.8)
	Ph	9.3	(20.2)
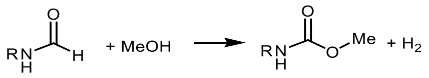 (3)	Me	2.6	(-1.8)
	Ph	1.6	(-2.6)
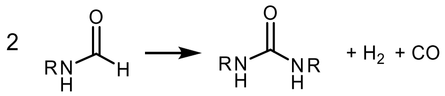 (4)	Me[a]	17.9	(23.9)
	Ph	16.1	(22.3)
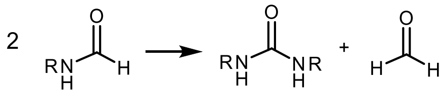 (5)	Me[a]	13.8	(14.4)
	Ph	12.0	(12.8)

[a]syn,anti conformation of N,N-dimethylurea

5.3. Direct decarbonylation of amide

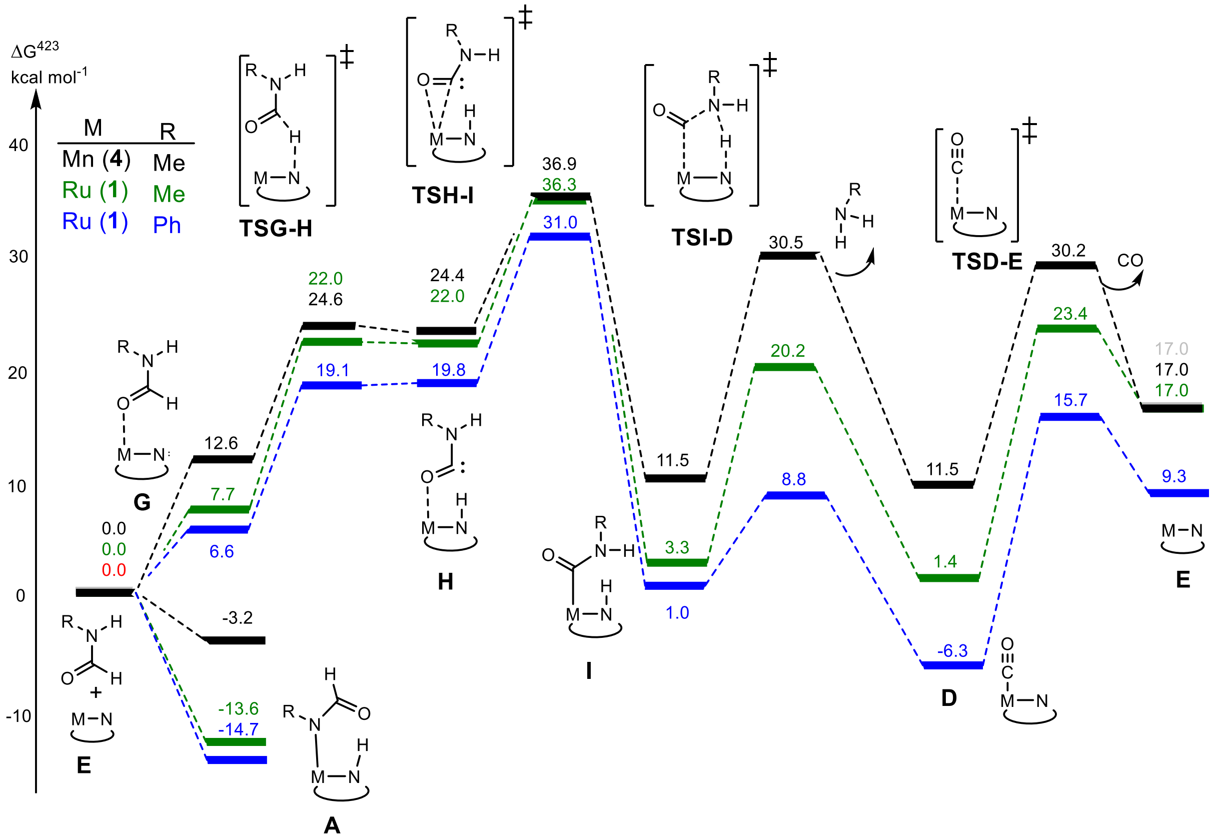

Figure S197: Direct decarbonylation pathway.

5.4. Production and decomposition of hemiaminals

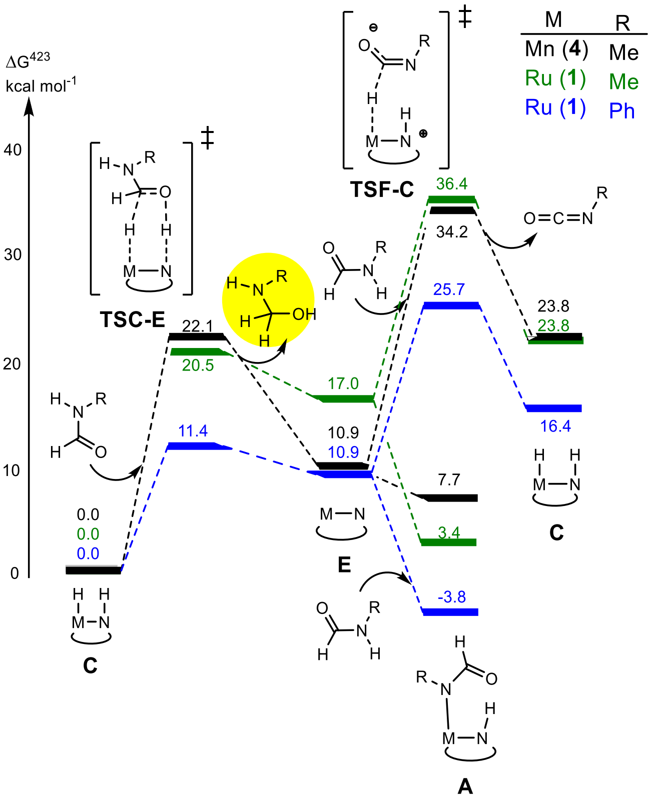

Figure S198: Catalytic formation of hemiaminal (highlighted). Regeneration of catalyst **C** is analogous to the first steps in Figure 4 in the main paper, (including off-cycle intermediate **A**) to illustrate that this reaction is also feasible under turnover conditions.

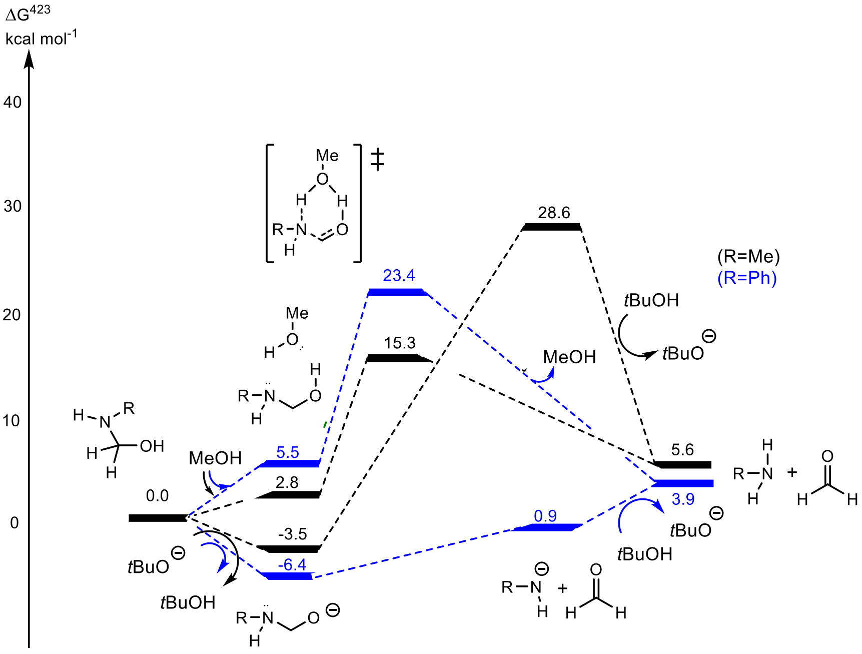

Figure S199: Decomposition of hemiaminal assisted through a protic solvent (upper branch) or through base (lower branch).

5.5. Computational raw data

1Aa_Ph
Frequencies, energies and thermodynamic properties:
Lowest Vibrational Mode (1/cm) = 12.1435
2nd Lowest Vibrational Mode (1/cm) = 17.9540
E(RB-P86) (a.u.) = -2430.01930784
Thermal correction to Enthalpy (a.u.) = 0.657786
Thermal correction to Gibbs Free Energy (a.u.) = 0.536432
Total Entropy (cal/Kmol) = 255.412
E(RPBE1PBE) (a.u.) = -2429.76683310
Optimised cartesian coordinates (Angstrom):

Ru	-0.079673	-0.328654	-0.019388
P	2.238190	-0.482649	0.275761
P	-2.379578	-0.431354	0.327941
N	-0.049126	0.150001	2.163633
O	-0.205057	-1.467900	-2.799763
C	3.046903	-2.154471	0.391073
C	-1.209966	-0.446229	2.888030
H	-1.241924	-0.065550	3.934317
H	-1.027151	-1.538556	2.940107
C	4.428999	-2.257761	0.676797
H	5.037636	-1.348986	0.813133
C	-3.596753	0.763553	-0.396668
C	3.443824	0.437673	-0.783603
C	-3.006479	-3.153228	0.873992
H	-2.395448	-3.036980	1.782825
C	3.425517	0.173663	-2.173186
H	2.701237	-0.546413	-2.586778
C	-3.219564	1.527456	-1.522029
H	-2.203849	1.417457	-1.930950
C	-2.536322	-0.151168	2.185133
H	-2.820424	0.913572	2.317699
H	-3.352346	-0.762380	2.621115
C	2.435179	0.240842	1.990854
H	3.388913	-0.061894	2.468976
H	2.436693	1.346557	1.882675
C	-3.204552	-2.060085	0.000062
C	1.239400	-0.203470	2.831904
H	1.249165	-1.305154	2.969090
H	1.282860	0.257573	3.844131
C	-4.127241	2.434351	-2.100367
H	-3.822451	3.026131	-2.977841
C	-4.894865	0.919452	0.144301
H	-5.213041	0.321812	1.013919
C	2.915570	-4.594040	0.286063
H	2.319174	-5.506214	0.124891
C	2.297308	-3.332920	0.193075
H	1.221809	-3.249722	-0.029902
C	-0.145637	-0.971920	-1.728597

C	4.375180	1.370201	-0.275493
H	4.416890	1.595540	0.800867
C	5.042333	-3.517382	0.776534
H	6.118546	-3.584285	1.002321
C	-3.577859	-4.405657	0.591235
H	-3.421153	-5.245305	1.287036
C	-5.795324	1.832076	-0.430267
H	-6.803132	1.949760	-0.001013
C	-3.973151	-2.250984	-1.169734
H	-4.139072	-1.411963	-1.863482
C	4.286422	-4.689158	0.580598
H	4.769873	-5.676367	0.653481
C	-5.413051	2.589679	-1.554283
H	-6.121472	3.303515	-2.003893
C	4.322704	0.825248	-3.034660
H	4.294795	0.610963	-4.114688
C	-4.341812	-4.587952	-0.576366
H	-4.785360	-5.571157	-0.799622
C	5.270685	2.024497	-1.142561
H	5.991423	2.750026	-0.732991
C	5.247073	1.754151	-2.521340
H	5.947058	2.269464	-3.197815
C	-4.534122	-3.508797	-1.456556
H	-5.128697	-3.642061	-2.374331
H	-0.166703	-1.853104	0.507738
H	0.501205	1.347359	-2.521896
H	-0.150986	1.184147	2.191513
N	0.165904	1.858472	-0.551747
C	0.501951	2.227664	-1.819948
O	0.750576	3.372286	-2.241182
C	0.197100	2.870777	0.437514
C	-0.939032	3.119544	1.251195
C	1.364214	3.644797	0.685882
C	-0.907590	4.080374	2.280662
H	-1.870919	2.577574	1.028303
C	1.390201	4.605775	1.708774
H	2.250118	3.482375	0.054817
C	0.258974	4.827232	2.520120
H	-1.810679	4.253735	2.888724
H	2.309155	5.191234	1.877804
H	0.285769	5.581031	3.322772

1Aa

Frequencies, energies and thermodynamic properties:

Lowest Vibrational Mode (1/cm) = 16.8989

2nd Lowest Vibrational Mode (1/cm) = 21.5726

E(RB-P86) (a.u.) = -2238.41040577

Thermal correction to Enthalpy (a.u.) = 0.602932

Thermal correction to Gibbs Free Energy (a.u.) = 0.490277

Total Entropy (cal/Kmol) = 237.103

E(RPBE1PBE) (a.u.) = -2238.16815725

Optimised cartesian coordinates (Angstrom):

Ru	0.030168	-0.014979	0.166979
P	-2.282400	0.164116	0.440220
P	2.333857	0.218305	0.431078
N	0.034455	0.168502	2.401305
O	0.054592	0.130066	-2.835490
C	-3.149888	1.757924	0.038502
C	1.220944	0.913753	2.912314
H	1.267700	0.844438	4.022995
H	1.065337	1.980452	2.652489
C	-4.526537	1.909424	0.331133
H	-5.094310	1.076142	0.776775
C	3.574381	-1.086361	-0.011303
C	-3.456689	-1.083408	-0.260550
C	3.184464	2.954546	0.409578
H	2.814551	3.026430	1.444024
C	-3.852922	-0.927860	-1.610591
H	-3.514926	-0.050989	-2.186461
C	3.149159	-2.227296	-0.724179
H	2.084661	-2.327935	-0.988886
C	2.517800	0.386020	2.295745
H	2.742411	-0.634958	2.671346
H	3.378374	1.028208	2.573474
C	-2.444574	0.028961	2.302894
H	-3.397379	0.464089	2.666710
H	-2.442715	-1.048761	2.568304
C	3.120292	1.732129	-0.294818
C	-1.241741	0.726229	2.937674
H	-1.251781	1.811484	2.704925
H	-1.270717	0.622100	4.045945
C	4.074286	-3.230272	-1.070799
H	3.733180	-4.117231	-1.627802
C	4.936310	-0.958723	0.350177
H	5.286661	-0.065830	0.893457
C	-3.114783	4.037302	-0.845057
H	-2.558645	4.866006	-1.311594
C	-2.451140	2.830608	-0.552986
H	-1.378779	2.709407	-0.774186
C	0.040927	0.039769	-1.656889
C	-3.899437	-2.218362	0.453095
H	-3.601831	-2.376967	1.500272
C	-5.184715	3.116901	0.046021
H	-6.255654	3.224341	0.281149
C	3.718708	4.103386	-0.201662
H	3.766917	5.047644	0.363839
C	5.854758	-1.965089	0.008731
H	6.913346	-1.860229	0.295651
C	3.595741	1.684603	-1.625619
H	3.557582	0.739792	-2.191406

C	-4.479705	4.183720	-0.544202
H	-4.998450	5.128561	-0.771939
C	5.424988	-3.101524	-0.704212
H	6.148195	-3.887356	-0.974770
C	-4.679275	-1.880911	-2.227287
H	-4.978803	-1.743885	-3.278465
C	4.188724	4.047695	-1.525724
H	4.605405	4.948311	-2.003766
C	-4.729425	-3.170668	-0.166969
H	-5.067694	-4.049291	0.404858
C	-5.121562	-3.005806	-1.506081
H	-5.768803	-3.754403	-1.989681
C	4.124287	2.834926	-2.236038
H	4.490711	2.780939	-3.273529
H	0.079724	1.611240	0.206645
H	-0.984654	-2.441469	-1.452208
H	0.108705	-0.808711	2.732248
N	-0.087315	-2.217642	0.388371
C	-0.588225	-3.026296	-0.575582
O	-0.635470	-4.277284	-0.579845
C	0.462240	-2.903175	1.550350
H	-0.114523	-2.697791	2.487237
H	0.429357	-4.002665	1.395575
H	1.520278	-2.609503	1.741156

1A_Ph

Frequencies, energies and thermodynamic properties:

Lowest Vibrational Mode (1/cm) = 15.3335
2nd Lowest Vibrational Mode (1/cm) = 23.7813
E(RB-P86) (a.u.) = -2430.03796399
Thermal correction to Enthalpy (a.u.) = 0.657451
Thermal correction to Gibbs Free Energy (a.u.) = 0.539141
Total Entropy (cal/Kmol) = 249.005
E(RPBE1PBE) (a.u.) = -2429.77744469

Optimised cartesian coordinates (Angstrom):

Ru	0.005895	-0.323450	0.228963
P	2.318462	-0.597076	0.468464
P	-2.301956	-0.641640	0.477350
N	0.014300	-1.001175	2.354002
O	0.036906	0.027027	-2.759364
C	3.063370	-2.068671	-0.386979
C	-1.205740	-1.739968	2.755748
H	-1.220726	-1.884371	3.861273
H	-1.179698	-2.751051	2.292578
C	3.556817	-3.199386	0.299521
H	3.529203	-3.249324	1.399374
C	-3.523628	0.701474	0.115852
C	3.605378	0.689427	0.112806
C	-4.478604	-2.398018	-0.025684
H	-5.069131	-1.711823	0.603103

C	4.928635	0.545742	0.590455
H	5.210022	-0.329107	1.197459
C	-3.764203	1.751494	1.030744
H	-3.289370	1.749896	2.023049
C	-2.452957	-0.973589	2.314953
H	-2.490125	0.005796	2.834318
H	-3.383016	-1.528358	2.551856
C	2.485559	-0.930757	2.298531
H	3.423038	-1.460908	2.562136
H	2.523604	0.068749	2.781025
C	-3.114149	-2.126140	-0.290096
C	1.250512	-1.700746	2.771395
H	1.237035	-2.726417	2.341975
H	1.273501	-1.809894	3.880910
C	-4.609550	2.820283	0.682987
H	-4.790660	3.627508	1.409888
C	-4.134331	0.755592	-1.159334
H	-3.954025	-0.048500	-1.890034
C	3.665797	-3.131619	-2.510540
H	3.707139	-3.097421	-3.611732
C	3.121004	-2.048732	-1.801605
H	2.735411	-1.178310	-2.357281
C	0.016791	-0.048830	-1.579936
C	3.277247	1.804507	-0.683935
H	2.249402	1.924120	-1.053005
C	4.096704	-4.287278	-0.413814
H	4.476549	-5.162384	0.137807
C	-5.095958	-3.536377	-0.568909
H	-6.158047	-3.736636	-0.354454
C	-4.975086	1.827183	-1.504655
H	-5.444396	1.851668	-2.500506
C	-2.385517	-3.011857	-1.111072
H	-1.321938	-2.804986	-1.309569
C	4.154815	-4.256317	-1.818200
H	4.580232	-5.106795	-2.374432
C	-5.217031	2.862437	-0.583784
H	-5.876552	3.702208	-0.854250
C	5.901572	1.511117	0.284748
H	6.930437	1.392185	0.667378
C	-4.360723	-4.415859	-1.387081
H	-4.846476	-5.307726	-1.814090
C	4.255781	2.767374	-0.995793
H	3.986323	3.635894	-1.613806
C	5.566456	2.623781	-0.511697
H	6.331912	3.378219	-0.753299
C	-3.007100	-4.150877	-1.657482
H	-2.426155	-4.833829	-2.297917
H	0.073877	-1.898791	-0.076119
H	-0.263926	3.371827	2.339404
H	-0.008293	-0.032061	2.810591

N	-0.100949	1.874754	0.895402
C	-0.147108	2.264433	2.178461
O	-0.100446	1.522799	3.203272
C	-0.079589	2.939442	-0.047235
C	-0.937256	2.939104	-1.174971
C	0.795006	4.047241	0.116478
C	-0.925585	4.001140	-2.093080
H	-1.634384	2.101200	-1.310264
C	0.796553	5.113508	-0.800352
H	1.503219	4.052428	0.959370
C	-0.062244	5.098996	-1.913328
H	-1.610176	3.973944	-2.956477
H	1.489851	5.956658	-0.647063
H	-0.055585	5.929794	-2.636590

1A

Frequencies, energies and thermodynamic properties:

Lowest Vibrational Mode (1/cm) = 13.3330
2nd Lowest Vibrational Mode (1/cm) = 20.1807
E(RB-P86) (a.u.) = -2238.43274329
Thermal correction to Enthalpy (a.u.) = 0.602561
Thermal correction to Gibbs Free Energy (a.u.) = 0.491598
Total Entropy (cal/Kmol) = 233.541
E(RPBE1PBE) (a.u.) = -2238.18631913

Optimised cartesian coordinates (Angstrom):

Ru	-0.005595	0.077723	0.087204
P	-2.314371	0.212634	0.387706
P	2.304912	0.148420	0.401185
N	-0.016632	0.141559	2.308715
O	0.014454	0.447721	-2.900726
C	-3.246895	1.730105	-0.132870
C	1.238604	0.662876	2.897198
H	1.245077	0.497924	4.000076
H	1.279399	1.761496	2.728360
C	-4.617403	1.873985	0.191054
H	-5.148027	1.070182	0.727243
C	3.378984	-1.178780	-0.315343
C	-3.399821	-1.166889	-0.205259
C	4.644881	1.767724	0.338896
H	5.160572	0.903963	0.789593
C	-4.126663	-1.038441	-1.412142
H	-4.124255	-0.081609	-1.957690
C	3.537446	-2.434811	0.313513
H	3.088439	-2.629184	1.298964
C	2.447505	-0.027435	2.262197
H	2.424180	-1.110321	2.501385
H	3.403853	0.387488	2.639334
C	-2.486037	0.199591	2.258543
H	-3.393619	0.749584	2.578605
H	-2.596392	-0.856333	2.577944

C	3.268051	1.688680	0.020301
C	-1.218976	0.801234	2.868837
H	-1.156716	1.890400	2.654192
H	-1.242132	0.682202	3.977535
C	4.270144	-3.458896	-0.313566
H	4.389911	-4.429154	0.194299
C	3.954993	-0.979082	-1.592877
H	3.837408	-0.010026	-2.103879
C	-3.298713	3.928724	-1.202518
H	-2.778023	4.730503	-1.749977
C	-2.594715	2.767802	-0.830886
H	-1.525034	2.660668	-1.068078
C	0.003766	0.259723	-1.732824
C	-3.405704	-2.412741	0.465252
H	-2.820535	-2.552550	1.387618
C	-5.316829	3.035489	-0.175732
H	-6.382676	3.136578	0.083991
C	5.368840	2.941969	0.075054
H	6.439439	2.992973	0.329859
C	4.683530	-2.005017	-2.217845
H	5.128326	-1.830160	-3.210425
C	2.634893	2.801450	-0.571307
H	1.561301	2.738144	-0.808846
C	-4.658549	4.065362	-0.875124
H	-5.208804	4.974846	-1.164448
C	4.845272	-3.248420	-1.579102
H	5.418182	-4.052253	-2.067946
C	-4.861325	-2.122700	-1.922928
H	-5.425990	-2.003237	-2.861391
C	4.729238	4.048558	-0.516436
H	5.299111	4.967857	-0.725696
C	-4.146853	-3.492754	-0.045809
H	-4.147655	-4.453600	0.493283
C	-4.878079	-3.351831	-1.239098
H	-5.457113	-4.200012	-1.637584
C	3.363670	3.975382	-0.840327
H	2.858309	4.836660	-1.305638
H	-0.003631	1.695469	0.216541
H	-0.014940	-4.025222	0.986922
H	-0.083896	-0.914030	2.488899
N	0.023409	-2.143090	0.115682
C	-0.062756	-2.915969	1.191218
O	-0.198298	-2.538626	2.400207
C	0.153085	-2.819059	-1.168438
H	-0.700519	-2.569676	-1.836656
H	1.082344	-2.504661	-1.692048
H	0.183978	-3.927578	-1.051094

1Ba_Ph

Frequencies, energies and thermodynamic properties:

Lowest Vibrational Mode (1/cm) = 8.6613
2nd Lowest Vibrational Mode (1/cm) = 17.3245
E(RB-P86) (a.u.) = -2428.82160651
Thermal correction to Enthalpy (a.u.) = 0.634748
Thermal correction to Gibbs Free Energy (a.u.) = 0.514391
Total Entropy (cal/Kmol) = 253.314
E(RPBE1PBE) (a.u.) = -2428.55444360
Optimised cartesian coordinates (Angstrom):

Ru	-0.008493	-0.587142	0.196982
P	-2.321487	-0.584309	-0.139292
P	2.303555	-0.643832	-0.138853
N	0.005868	0.502878	-1.725079
O	-0.023848	-1.814617	2.947172
C	-3.127500	-2.208313	-0.501716
C	1.234324	0.295721	-2.551659
H	1.261624	1.062996	-3.356745
H	1.144104	-0.705035	-3.023001
C	-3.609272	-2.565763	-1.779257
H	-3.556265	-1.857664	-2.620322
C	3.482082	0.128774	1.059729
C	-3.480097	0.212663	1.062638
C	3.545559	-2.665755	-1.765261
H	3.516417	-1.959827	-2.609244
C	-4.879703	0.035467	0.976990
H	-5.308347	-0.619922	0.201778
C	2.967435	0.983727	2.059309
H	1.880045	1.150230	2.120085
C	2.496085	0.393140	-1.690158
H	2.636377	1.434927	-1.332741
H	3.404692	0.131414	-2.268854
C	-2.486481	0.464098	-1.685887
H	-3.403332	0.231229	-2.263920
H	-2.595354	1.508199	-1.324226
C	3.066839	-2.290407	-0.491778
C	-1.229504	0.332630	-2.549853
H	-1.168844	-0.669212	-3.023531
H	-1.235976	1.102280	-3.353137
C	3.838554	1.617217	2.963737
H	3.429446	2.280968	3.741837
C	4.876255	-0.088720	0.978778
H	5.287837	-0.761743	0.209461
C	-3.772334	-4.417632	0.330899
H	-3.833327	-5.138810	1.161290
C	-3.209739	-3.150637	0.551165
H	-2.827475	-2.892970	1.552304
C	-0.017901	-1.331913	1.868906
C	-2.943860	1.045251	2.069658
H	-1.852394	1.180612	2.134027
C	-4.165758	-3.840513	-1.999111
H	-4.536435	-4.105526	-3.002054

C	4.068334	-3.956008	-1.977357
H	4.436993	-4.234848	-2.977276
C	5.742566	0.546393	1.885061
H	6.828155	0.371549	1.817080
C	3.117833	-3.230558	0.565028
H	2.737427	-2.958738	1.563092
C	-4.250795	-4.767162	-0.946626
H	-4.687727	-5.763223	-1.120223
C	5.225367	1.399612	2.878074
H	5.906350	1.893036	3.589662
C	-5.729853	0.688216	1.886020
H	-6.819799	0.544847	1.814263
C	4.122563	-4.880548	-0.921031
H	4.533143	-5.888786	-1.088550
C	-3.798823	1.696414	2.976956
H	-3.372899	2.342439	3.760925
C	-5.191033	1.518923	2.886614
H	-5.859451	2.026167	3.600395
C	3.646975	-4.513201	0.352548
H	3.683848	-5.232580	1.185899
H	-0.026477	-2.075426	-0.366602
N	0.043933	2.889004	-1.831737
C	0.025059	1.861401	-1.039827
O	0.018807	1.713030	0.237428
C	0.061821	4.224300	-1.411833
C	0.097209	5.211247	-2.436206
C	0.046066	4.674047	-0.059505
C	0.116211	6.579790	-2.132098
H	0.109176	4.864683	-3.481943
C	0.064518	6.047899	0.235619
H	0.019303	3.929284	0.747497
C	0.099955	7.010142	-0.790757
H	0.143822	7.319252	-2.949329
H	0.051394	6.371629	1.289831
H	0.114604	8.084804	-0.548717

1Ba

Frequencies, energies and thermodynamic properties:

Lowest Vibrational Mode (1/cm) = 14.8379
2nd Lowest Vibrational Mode (1/cm) = 16.0559
E(RB-P86) (a.u.) = -2237.20853369
Thermal correction to Enthalpy (a.u.) = 0.579978
Thermal correction to Gibbs Free Energy (a.u.) = 0.468016
Total Entropy (cal/Kmol) = 235.644
E(RPBE1PBE) (a.u.) = -2236.95819019

Optimised cartesian coordinates (Angstrom):

Ru	0.017262	-0.072129	0.000000
P	-0.100661	0.236991	2.312643
P	-0.100661	0.236991	-2.312643
N	0.451433	2.093090	0.000000

O	-0.280994	-3.069153	0.000000
C	-1.753738	0.054593	3.120705
C	0.014987	2.817440	-1.231713
H	0.489872	3.823544	-1.247353
H	-1.085083	2.949358	-1.162661
C	-2.477543	1.138292	3.662286
H	-2.059221	2.156189	3.651527
C	1.041644	-0.654917	-3.464106
C	1.041644	-0.654917	3.464106
C	-2.477543	1.138292	-3.662286
H	-2.059221	2.156189	-3.651527
C	0.818916	-0.698943	4.858812
H	-0.075048	-0.222182	5.291878
C	2.185756	-1.280968	-2.920987
H	2.354114	-1.241644	-1.832689
C	0.402827	2.035420	-2.489016
H	1.507380	2.020917	-2.600157
H	-0.004598	2.508029	-3.405268
C	0.402827	2.035420	2.489016
H	-0.004598	2.508029	3.405268
H	1.507380	2.020917	2.600157
C	-1.753738	0.054593	-3.120705
C	0.014987	2.817440	1.231713
H	-1.085083	2.949358	1.162661
H	0.489872	3.823544	1.247353
C	3.098228	-1.938393	-3.765358
H	3.987231	-2.425741	-3.334407
C	0.818916	-0.698943	-4.858812
H	-0.075048	-0.222182	-5.291878
C	-3.596708	-1.440922	3.719597
H	-4.030055	-2.453453	3.738583
C	-2.330035	-1.237912	3.149895
H	-1.784775	-2.093350	2.719103
C	-0.163206	-1.892959	0.000000
C	2.185756	-1.280968	2.920987
H	2.354114	-1.241644	1.832689
C	-3.752116	0.933279	4.224978
H	-4.305914	1.789521	4.641715
C	-3.752116	0.933279	-4.224978
H	-4.305914	1.789521	-4.641715
C	1.733462	-1.358432	-5.698290
H	1.552711	-1.390502	-6.784610
C	-2.330035	-1.237912	-3.149895
H	-1.784775	-2.093350	-2.719103
C	-4.312935	-0.354331	4.257650
H	-5.309307	-0.512982	4.699570
C	2.873477	-1.978565	-5.153374
H	3.586423	-2.498174	-5.813195
C	1.733462	-1.358432	5.698290
H	1.552711	-1.390502	6.784610

C	-4.312935	-0.354331	-4.257650
H	-5.309307	-0.512982	-4.699570
C	3.098228	-1.938393	3.765358
H	3.987231	-2.425741	3.334407
C	2.873477	-1.978565	5.153374
H	3.586423	-2.498174	5.813195
C	-3.596708	-1.440922	-3.719597
H	-4.030055	-2.453453	-3.738583
H	-1.575711	0.001514	0.000000
N	2.708449	2.901426	0.000000
C	1.953225	1.858103	0.000000
O	2.210437	0.589367	0.000000
C	4.146152	2.675663	0.000000
H	4.488997	2.100490	-0.893077
H	4.684159	3.646517	0.000000
H	4.488997	2.100490	0.893077

1B_Ph

Frequencies, energies and thermodynamic properties:

Lowest Vibrational Mode (1/cm) = 14.7855
2nd Lowest Vibrational Mode (1/cm) = 19.2142
E(RB-P86) (a.u.) = -2428.83455025
Thermal correction to Enthalpy (a.u.) = 0.635363
Thermal correction to Gibbs Free Energy (a.u.) = 0.518176
Total Entropy (cal/Kmol) = 246.640
E(RPBE1PBE) (a.u.) = -2428.57462834

Optimised cartesian coordinates (Angstrom):

Ru	-0.239714	-0.034261	0.000000
P	-0.519890	0.267750	2.307188
P	-0.519890	0.267750	-2.307188
N	-0.140323	2.173876	0.000000
O	-0.139610	-3.044922	0.000000
C	-2.227423	-0.049395	2.953399
C	-0.669970	2.841800	-1.228436
H	-0.318941	3.897814	-1.248931
H	-1.776254	2.849196	-1.144534
C	-3.062104	0.958192	3.482045
H	-2.715108	2.000395	3.550308
C	0.546271	-0.468003	-3.633890
C	0.546271	-0.468003	3.633890
C	-3.062104	0.958192	-3.482045
H	-2.715108	2.000395	-3.550308
C	0.168226	-0.411526	4.995394
H	-0.792567	0.043076	5.285352
C	1.773513	-1.068345	-3.281345
H	2.071368	-1.117271	-2.222796
C	-0.215780	2.107460	-2.491051
H	0.881525	2.210558	-2.626903
H	-0.688584	2.531953	-3.399348
C	-0.215780	2.107460	2.491051

H	-0.688584	2.531953	3.399348
H	0.881525	2.210558	2.626903
C	-2.227423	-0.049395	-2.953399
C	-0.669970	2.841800	1.228436
H	-1.776254	2.849196	1.144534
H	-0.318941	3.897814	1.248931
C	2.615592	-1.598483	-4.275669
H	3.571038	-2.064810	-3.987699
C	0.168226	-0.411526	-4.995394
H	-0.792567	0.043076	-5.285352
C	-4.004791	-1.687077	3.343422
H	-4.368253	-2.725379	3.286376
C	-2.715302	-1.376086	2.884524
H	-2.082415	-2.172919	2.461447
C	-0.173041	-1.862631	0.000000
C	1.773513	-1.068345	3.281345
H	2.071368	-1.117271	2.222796
C	-4.359951	0.645715	3.931823
H	-5.000802	1.444496	4.337813
C	-4.359951	0.645715	-3.931823
H	-5.000802	1.444496	-4.337813
C	1.011937	-0.942180	-5.985891
H	0.708771	-0.894011	-7.043977
C	-2.715302	-1.376086	-2.884524
H	-2.082415	-2.172919	-2.461447
C	-4.833055	-0.675187	3.866376
H	-5.847586	-0.918023	4.219952
C	2.236802	-1.536721	-5.628131
H	2.894424	-1.955945	-6.406182
C	1.011937	-0.942180	5.985891
H	0.708771	-0.894011	7.043977
C	-4.833055	-0.675187	-3.866376
H	-5.847586	-0.918023	-4.219952
C	2.615592	-1.598483	4.275669
H	3.571038	-2.064810	3.987699
C	2.236802	-1.536721	5.628131
H	2.894424	-1.955945	6.406182
C	-4.004791	-1.687077	-3.343422
H	-4.368253	-2.725379	-3.286376
H	-1.838871	-0.164130	0.000000
N	1.794432	0.933840	0.000000
C	1.393216	2.213069	0.000000
O	1.973765	3.303128	0.000000
C	3.137475	0.530268	0.000000
C	3.411192	-0.865069	0.000000
C	4.246528	1.424586	0.000000
C	4.728370	-1.349410	0.000000
H	2.558316	-1.562114	0.000000
C	5.559909	0.927350	0.000000
H	4.054303	2.505327	0.000000

C	5.816701	-0.456881	0.000000
H	4.903787	-2.437580	0.000000
H	6.400525	1.640979	0.000000
H	6.851117	-0.835064	0.000000

1B

Frequencies, energies and thermodynamic properties:

Lowest Vibrational Mode (1/cm) = 13.5038

2nd Lowest Vibrational Mode (1/cm) = 15.4721

E(RB-P86) (a.u.) = -2237.22168251

Thermal correction to Enthalpy (a.u.) = 0.580153

Thermal correction to Gibbs Free Energy (a.u.) = 0.468341

Total Entropy (cal/Kmol) = 235.327

E(RPBE1PBE) (a.u.) = -2236.97546119

Optimised cartesian coordinates (Angstrom):

Ru	0.000007	-0.106344	-0.012582
P	-2.306748	0.085139	0.286654
P	2.306891	0.084453	0.286513
N	0.000062	-0.206136	2.198022
O	-0.000263	-0.189392	-3.024620
C	-3.086878	1.719947	-0.100434
C	1.229990	0.316206	2.861751
H	1.247183	-0.025919	3.921103
H	1.160844	1.423750	2.859431
C	-3.473040	2.647451	0.890825
H	-3.361554	2.409494	1.959706
C	3.525746	-1.107760	-0.439464
C	-3.525923	-1.107190	-0.438591
C	3.476546	2.645130	0.891910
H	3.367221	2.405861	1.960722
C	-4.908221	-0.998594	-0.159757
H	-5.283202	-0.182622	0.479318
C	3.068805	-2.146067	-1.278405
H	1.993235	-2.223977	-1.501479
C	2.489793	-0.165932	2.136492
H	2.614436	-1.260258	2.274835
H	3.400535	0.315421	2.545831
C	-2.489662	-0.164167	2.136819
H	-3.399943	0.318280	2.545910
H	-2.615283	-1.258307	2.275749
C	3.087307	1.719346	-0.099749
C	-1.229401	0.317189	2.861813
H	-1.159420	1.424679	2.859321
H	-1.246716	-0.024765	3.921219
C	3.979173	-3.068206	-1.827713
H	3.612593	-3.872438	-2.485185
C	4.908239	-0.998966	-0.161701
H	5.283619	-0.182845	0.476947
C	-3.781582	3.314500	-1.823025
H	-3.900143	3.569854	-2.888026

C	-3.243243	2.069000	-1.462779
H	-2.939619	1.360770	-2.250995
C	-0.000150	-0.166324	-1.841203
C	-3.069508	-2.145309	-1.278041
H	-1.994100	-2.223103	-1.501921
C	-4.007508	3.898404	0.526947
H	-4.303882	4.611555	1.312595
C	4.011407	3.896075	0.528543
H	4.310142	4.607892	1.314506
C	5.813932	-1.924672	-0.705917
H	6.888812	-1.834428	-0.481872
C	3.241038	2.070108	-1.461961
H	2.934886	1.363280	-2.250458
C	-4.164355	4.234594	-0.828086
H	-4.583523	5.213162	-1.110846
C	5.350803	-2.960593	-1.539981
H	6.063566	-3.682272	-1.969724
C	-5.814229	-1.924300	-0.703443
H	-6.888946	-1.834219	-0.478556
C	4.165656	4.233932	-0.826365
H	4.585137	5.212484	-1.108719
C	-3.980201	-3.067437	-1.826839
H	-3.614032	-3.871519	-2.484723
C	-5.351621	-2.960023	-1.538046
H	-6.064629	-3.681713	-1.967362
C	3.779838	3.315543	-1.821710
H	3.896321	3.572218	-2.886622
H	0.000256	1.502793	-0.136305
N	-0.000458	-2.118543	0.949019
C	-0.000506	-1.746122	2.222196
O	-0.000887	-2.349748	3.303665
C	-0.000827	-3.527352	0.603476
H	-0.000952	-4.165473	1.513474
H	-0.895298	-3.788276	-0.004349
H	0.893452	-3.788708	-0.004444

1C

Frequencies, energies and thermodynamic properties:

Lowest Vibrational Mode (1/cm) = 13.2296
2nd Lowest Vibrational Mode (1/cm) = 21.5136
E(RB-P86) (a.u.) = -2030.53577943
Thermal correction to Enthalpy (a.u.) = 0.542191
Thermal correction to Gibbs Free Energy (a.u.) = 0.440343
Total Entropy (cal/Kmol) = 214.357
E(RPBE1PBE) (a.u.) = -2030.28094031

Optimised cartesian coordinates (Angstrom):

Ru	0.000000	-0.144756	0.171019
P	-2.297944	-0.053918	0.447102
P	2.297943	-0.053891	0.447107
O	0.000004	-0.371617	-2.830809

N	-0.000001	-0.017626	2.410960
C	-1.236803	0.611930	2.948526
H	-1.179281	1.693715	2.708526
H	-1.270007	0.512313	4.058031
C	1.236777	0.611983	2.948519
H	1.269982	0.512383	4.058026
H	1.179214	1.693761	2.708503
C	2.478417	-0.020827	2.319055
H	2.570555	-1.084275	2.627691
H	3.405837	0.491149	2.647613
C	-2.478418	-0.020915	2.319049
H	-3.405859	0.491011	2.647626
H	-2.570510	-1.084376	2.627654
C	0.000006	-0.271818	-1.649923
H	0.000021	-1.009175	2.696561
H	0.000001	-1.823418	0.384612
H	0.000000	1.541472	0.247023
C	-3.436374	-1.440493	-0.044544
C	-4.840131	-1.317365	0.078107
C	-2.888429	-2.652262	-0.514904
C	-5.677878	-2.395183	-0.255645
H	-5.284709	-0.372741	0.431349
C	-3.729992	-3.728199	-0.853893
H	-1.792207	-2.736366	-0.602967
C	-5.124282	-3.602411	-0.723604
H	-6.770034	-2.290927	-0.153584
H	-3.292009	-4.668754	-1.224946
H	-5.783141	-4.444253	-0.990318
C	-3.224329	1.423748	-0.187027
C	-3.601850	2.507752	0.634587
C	-3.490216	1.495676	-1.574957
C	-4.235209	3.637034	0.081982
H	-3.404396	2.487573	1.717482
C	-4.123516	2.622168	-2.124607
H	-3.198724	0.662280	-2.234966
C	-4.498314	3.697817	-1.297235
H	-4.524677	4.473437	0.738160
H	-4.324280	2.660600	-3.207187
H	-4.993719	4.582104	-1.728483
C	3.436385	-1.440472	-0.044494
C	4.840138	-1.317345	0.078201
C	2.888454	-2.652243	-0.514866
C	5.677894	-2.395166	-0.255517
H	5.284707	-0.372720	0.431452
C	3.730026	-3.728182	-0.853824
H	1.792234	-2.736347	-0.602961
C	5.124312	-3.602395	-0.723490
H	6.770047	-2.290911	-0.153421
H	3.292054	-4.668738	-1.224887
H	5.783178	-4.444239	-0.990178

C	3.224322	1.423759	-0.187070
C	3.601752	2.507828	0.634501
C	3.490291	1.495609	-1.574987
C	4.235103	3.637099	0.081865
H	3.404232	2.487709	1.717385
C	4.123580	2.622093	-2.124668
H	3.198873	0.662161	-2.234962
C	4.498288	3.697807	-1.297341
H	4.524500	4.473554	0.738009
H	4.324408	2.660466	-3.207238
H	4.993686	4.582086	-1.728613

1D

Frequencies, energies and thermodynamic properties:

Lowest Vibrational Mode (1/cm) =	6.0121
2nd Lowest Vibrational Mode (1/cm) =	12.2550
E(RB-P86) (a.u.) =	-2142.60151668
Thermal correction to Enthalpy (a.u.) =	0.531543
Thermal correction to Gibbs Free Energy (a.u.) =	0.423150
Total Entropy (cal/Kmol) =	228.131
E(RPBE1PBE) (a.u.) =	-2142.34909827

Optimised cartesian coordinates (Angstrom):

Ru	-0.000467	-0.290707	0.209559
P	-2.318005	0.006065	0.493102
P	2.318054	-0.000438	0.492050
O	-0.001663	-0.229528	-2.838912
N	0.000443	-0.016065	2.373623
C	-1.178129	0.651236	2.900377
H	-1.171803	1.761048	2.684445
H	-1.201089	0.572081	4.018612
C	1.183739	0.642335	2.901248
H	1.206649	0.560919	4.019319
H	1.184918	1.752542	2.687310
C	2.472456	0.029457	2.344559
H	2.556022	-1.035756	2.648478
H	3.385898	0.557825	2.686009
C	-2.471350	0.045913	2.345829
H	-3.380519	0.583428	2.684398
H	-2.563382	-1.017149	2.654816
C	-0.001285	-0.300807	-1.667051
H	0.001392	1.370477	0.173370
C	-3.531976	-1.268406	-0.079484
C	-4.875562	-1.211637	0.361102
C	-3.137661	-2.300134	-0.956800
C	-5.800627	-2.180822	-0.059844
H	-5.206416	-0.404416	1.034471
C	-4.069025	-3.265062	-1.384102
H	-2.095893	-2.347662	-1.309184
C	-5.399007	-3.209160	-0.934014
H	-6.842645	-2.131274	0.294049

H	-3.749647	-4.064316	-2.071435
H	-6.126862	-3.966545	-1.265648
C	-3.067041	1.565287	-0.163158
C	-3.285901	2.698458	0.650282
C	-3.379753	1.637256	-1.540516
C	-3.809602	3.880668	0.094907
H	-3.043976	2.672371	1.723626
C	-3.901215	2.819340	-2.091481
H	-3.219605	0.761631	-2.190319
C	-4.117554	3.945183	-1.274969
H	-3.977290	4.756282	0.742032
H	-4.141544	2.859954	-3.165752
H	-4.526920	4.871897	-1.707275
C	3.529690	-1.273660	-0.088380
C	4.876202	-1.214553	0.342857
C	3.130664	-2.306915	-0.961733
C	5.799600	-2.183089	-0.083161
H	5.210583	-0.405828	1.012684
C	4.060311	-3.271246	-1.394097
H	2.086645	-2.356011	-1.307153
C	5.393267	-3.213103	-0.953185
H	6.843994	-2.131688	0.263391
H	3.737287	-4.071725	-2.078295
H	6.119814	-3.969950	-1.288895
C	3.069060	1.561004	-0.156561
C	3.314823	2.681535	0.666473
C	3.357955	1.646688	-1.538406
C	3.840877	3.865125	0.116019
H	3.092627	2.644828	1.743720
C	3.882354	2.829719	-2.084279
H	3.176282	0.781041	-2.195903
C	4.125056	3.943241	-1.258197
H	4.029280	4.730946	0.770580
H	4.103938	2.881014	-3.162112
H	4.536400	4.870906	-1.686568
C	-0.002987	-2.199950	0.518538
O	-0.004802	-3.336861	0.781082

1E

Frequencies, energies and thermodynamic properties:

Lowest Vibrational Mode (1/cm) =	11.0341
2nd Lowest Vibrational Mode (1/cm) =	12.2184
E(RB-P86) (a.u.) =	-2029.33359612
Thermal correction to Enthalpy (a.u.) =	0.521915
Thermal correction to Gibbs Free Energy (a.u.) =	0.418245
Total Entropy (cal/Kmol) =	218.192
E(RPBE1PBE) (a.u.) =	-2029.07467588

Optimised cartesian coordinates (Angstrom):

Ru	0.000030	-0.222834	0.194221
P	-2.305999	-0.039716	0.454023

P	2.306032	-0.039626	0.454034
O	-0.000027	-1.842836	-2.371499
N	0.000014	0.199825	2.174974
C	-1.209133	0.601518	2.907603
H	-1.325592	1.716153	2.907849
H	-1.118233	0.310276	3.983301
C	1.209112	0.601634	2.907623
H	1.118242	0.310337	3.983309
H	1.325414	1.716286	2.907923
C	2.460116	-0.052317	2.315200
H	2.487580	-1.130521	2.580543
H	3.401881	0.403105	2.683532
C	-2.460050	-0.052606	2.315200
H	-3.401852	0.402679	2.683600
H	-2.487353	-1.130826	2.580496
C	0.000012	-1.190275	-1.382470
H	0.000083	1.025930	-0.772508
C	-3.475878	-1.375416	-0.083725
C	-4.804198	-1.114306	-0.485922
C	-3.014847	-2.711861	-0.054703
C	-5.657366	-2.173545	-0.845623
H	-5.176483	-0.078446	-0.521012
C	-3.871558	-3.768269	-0.407791
H	-1.972775	-2.920426	0.240502
C	-5.194886	-3.501062	-0.805265
H	-6.690982	-1.957594	-1.160128
H	-3.500300	-4.805156	-0.380791
H	-5.864412	-4.328589	-1.088986
C	-3.110480	1.514124	-0.156038
C	-3.944258	2.325332	0.644782
C	-2.863690	1.898999	-1.495380
C	-4.517350	3.498202	0.117147
H	-4.156709	2.051820	1.689541
C	-3.448011	3.061425	-2.023951
H	-2.199215	1.284029	-2.123495
C	-4.273866	3.866902	-1.216989
H	-5.159889	4.124674	0.756190
H	-3.250083	3.344880	-3.069932
H	-4.724617	4.783970	-1.628414
C	3.476125	-1.375189	-0.083574
C	4.804836	-1.113955	-0.484416
C	3.014975	-2.711608	-0.055658
C	5.658257	-2.173059	-0.843892
H	5.177215	-0.078097	-0.518641
C	3.871931	-3.767891	-0.408545
H	1.972634	-2.920238	0.238541
C	5.195636	-3.500566	-0.804670
H	6.692179	-1.957022	-1.157332
H	3.500576	-4.804766	-0.382437
H	5.865360	-4.327989	-1.088226

C	3.110274	1.514232	-0.156255
C	3.942185	2.326886	0.645026
C	2.864985	1.897738	-1.496275
C	4.514932	3.499836	0.117177
H	4.153421	2.054470	1.690316
C	3.448982	3.060232	-2.025032
H	2.201958	1.281639	-2.124820
C	4.272971	3.867168	-1.217606
H	5.156007	4.127443	0.756577
H	3.252260	3.342603	-3.071533
H	4.723457	4.784293	-1.629194

1F_Ph

Frequencies, energies and thermodynamic properties:

Lowest Vibrational Mode (1/cm) = 10.5994

2nd Lowest Vibrational Mode (1/cm) = 17.1535

E(RB-P86) (a.u.) = -2430.01310023

Thermal correction to Enthalpy (a.u.) = 0.658884

Thermal correction to Gibbs Free Energy (a.u.) = 0.537920

Total Entropy (cal/Kmol) = 254.590

E(RPBE1PBE) (a.u.) = -2429.74256519

Optimised cartesian coordinates (Angstrom):

Ru	0.545957	0.194114	0.000000
P	0.615425	-0.166949	2.313438
P	0.615425	-0.166949	-2.313438
N	-0.368893	-1.799010	0.000000
O	1.712878	2.977356	0.000000
C	2.292330	-0.511365	3.017613
C	-0.146137	-2.604826	-1.229292
H	-0.836040	-3.480329	-1.231346
H	0.889611	-3.007147	-1.206013
C	2.661168	-1.756601	3.570993
H	1.938837	-2.585389	3.627742
C	-0.136133	0.972823	-3.563842
C	-0.136133	0.972823	3.563842
C	2.661168	-1.756601	-3.570993
H	1.938837	-2.585389	-3.627742
C	0.160670	0.841300	4.940484
H	0.876457	0.076244	5.281949
C	-1.044488	1.968902	-3.142139
H	-1.284818	2.082570	-2.073502
C	-0.371949	-1.746837	-2.476224
H	-1.432090	-1.420783	-2.531645
H	-0.148383	-2.299594	-3.410974
C	-0.371949	-1.746837	2.476224
H	-0.148383	-2.299594	3.410974
H	-1.432090	-1.420783	2.531645
C	2.292330	-0.511365	-3.017613
C	-0.146137	-2.604826	1.229292
H	0.889611	-3.007147	1.206013

H	-0.836040	-3.480329	1.231346
C	-1.653105	2.815921	-4.086176
H	-2.359673	3.589634	-3.746082
C	0.160670	0.841300	-4.940484
H	0.876457	0.076244	-5.281949
C	4.548755	0.326454	3.463615
H	5.283504	1.146262	3.419230
C	3.252273	0.527823	2.964739
H	2.985212	1.502726	2.525389
C	1.254530	1.891829	0.000000
C	-1.044488	1.968902	3.142139
H	-1.284818	2.082570	2.073502
C	3.965816	-1.958760	4.061501
H	4.239956	-2.937038	4.487658
C	3.965816	-1.958760	-4.061501
H	4.239956	-2.937038	-4.487658
C	-0.449917	1.689730	-5.879392
H	-0.212637	1.579559	-6.949769
C	3.252273	0.527823	-2.964739
H	2.985212	1.502726	-2.525389
C	4.910347	-0.919715	4.011277
H	5.929687	-1.079432	4.396810
C	-1.357300	2.678510	-5.453991
H	-1.831512	3.345630	-6.191266
C	-0.449917	1.689730	5.879392
H	-0.212637	1.579559	6.949769
C	4.910347	-0.919715	-4.011277
H	5.929687	-1.079432	-4.396810
C	-1.653105	2.815921	4.086176
H	-2.359673	3.589634	3.746082
C	-1.357300	2.678510	5.453991
H	-1.831512	3.345630	6.191266
C	4.548755	0.326454	-3.463615
H	5.283504	1.146262	-3.419230
H	2.020173	-0.396777	0.000000
H	-1.270774	0.793183	0.000000
H	-1.409094	-1.471463	0.000000
N	-2.843963	-0.553853	0.000000
C	-2.467613	0.714633	0.000000
O	-3.032840	1.819542	0.000000
C	-4.189862	-0.955108	0.000000
C	-4.451694	-2.353552	0.000000
C	-5.308445	-0.073280	0.000000
C	-5.763058	-2.850881	0.000000
H	-3.595892	-3.047897	0.000000
C	-6.618384	-0.581626	0.000000
H	-5.125082	1.009709	0.000000
C	-6.860518	-1.968049	0.000000
H	-5.930323	-3.940674	0.000000
H	-7.466839	0.122996	0.000000

H -7.891261 -2.356732 0.000000

1F
Frequencies, energies and thermodynamic properties:
Lowest Vibrational Mode (1/cm) = 14.8258
2nd Lowest Vibrational Mode (1/cm) = 21.9248
E(RB-P86) (a.u.) = -2238.39904592
Thermal correction to Enthalpy (a.u.) = 0.599878
Thermal correction to Gibbs Free Energy (a.u.) = 0.483763
Total Entropy (cal/Kmol) = 244.385
E(RPBE1PBE) (a.u.) = -2238.14451989
Optimised cartesian coordinates (Angstrom):

Ru	-0.000130	-0.049292	-0.016307
P	-2.312061	0.138976	0.296685
P	2.311617	0.140386	0.296664
N	-0.000066	-0.222523	2.172019
O	0.000151	0.087576	-3.031190
C	-3.016239	1.833697	0.047421
C	1.230251	0.262992	2.848756
H	1.244246	-0.086041	3.907634
H	1.212234	1.374563	2.873077
C	-3.653229	2.568202	1.070764
H	-3.772102	2.143027	2.078907
C	3.564288	-0.956534	-0.516538
C	-3.563978	-0.957938	-0.517651
C	3.649474	2.571988	1.068993
H	3.767076	2.148533	2.078004
C	-4.930075	-0.597249	-0.571843
H	-5.264304	0.366479	-0.155871
C	3.152350	-2.190886	-1.066583
H	2.092399	-2.487502	-1.032824
C	2.475305	-0.246267	2.117975
H	2.524137	-1.354240	2.172662
H	3.411929	0.147912	2.561265
C	-2.475357	-0.249562	2.117612
H	-3.412623	0.142688	2.561266
H	-2.522319	-1.357672	2.171331
C	3.014891	1.835256	0.045760
C	-1.231109	0.260978	2.848864
H	-1.214628	1.372559	2.873804
H	-1.244778	-0.088672	3.907543
C	4.095879	-3.052934	-1.654231
H	3.762942	-4.012499	-2.080379
C	4.930150	-0.594970	-0.570869
H	5.263632	0.369435	-0.155863
C	-3.387787	3.703648	-1.488096
H	-3.280550	4.143138	-2.492431
C	-2.884171	2.418024	-1.234863
H	-2.376608	1.864752	-2.041215
C	0.000018	0.030740	-1.853449

C -3.151118 -2.191431 -1.068943
H -2.090962 -2.487297 -1.035310
C -4.147362 3.862425 0.817075
H -4.637769 4.425004 1.627326
C 4.142762 3.866296 0.814097
H 4.631293 4.430609 1.624277
C 5.868510 -1.459379 -1.160279
H 6.930209 -1.167673 -1.198178
C 2.884373 2.417460 -1.237651
H 2.378721 1.862444 -2.044007
C -4.018643 4.431445 -0.460848
H -4.406915 5.443243 -0.657704
C 5.453307 -2.689497 -1.703247
H 6.189462 -3.363711 -2.169164
C -5.867733 -1.461672 -1.162352
H -6.929618 -1.170635 -1.200164
C 4.015590 4.433204 -0.464917
H 4.403209 5.445069 -0.662714
C -4.093938 -3.053509 -1.657666
H -3.760272 -4.012395 -2.084771
C -5.451603 -2.690933 -1.706548
H -6.187213 -3.365145 -2.173326
C 3.387128 3.703188 -1.492059
H 3.281114 4.141004 -2.497255
H -0.000529 1.540820 0.039661
H -0.002113 -1.950083 0.029695
H 0.000824 -1.326170 2.216837
N 0.002423 -2.926830 1.862371
C 0.000387 -3.034552 0.554539
O 0.000699 -3.980358 -0.254266
C 0.004936 -4.172769 2.620973
H 0.032658 -3.952980 3.708174
H -0.901364 -4.792921 2.423708
H 0.884138 -4.816247 2.381021

1G_Ph
Frequencies, energies and thermodynamic properties:
Lowest Vibrational Mode (1/cm) = 10.4345
2nd Lowest Vibrational Mode (1/cm) = 13.4223
E(RB-P86) (a.u.) = -2430.00571915
Thermal correction to Enthalpy (a.u.) = 0.656347
Thermal correction to Gibbs Free Energy (a.u.) = 0.531688
Total Entropy (cal/Kmol) = 262.368
E(RPBE1PBE) (a.u.) = -2429.73343480
Optimised cartesian coordinates (Angstrom):
Ru -0.066560 -0.612839 0.000000
P -0.374815 -0.837245 2.294853
P -0.374815 -0.837245 -2.294853
N -2.193563 -0.664384 0.000000
O 2.965084 -0.511973 0.000000

C	0.028798	-2.493792	3.025222
C	-2.876240	-1.153414	-1.193652
H	-3.954248	-0.841836	-1.184501
H	-2.901463	-2.279471	-1.242462
C	-0.943007	-3.367794	3.558257
H	-2.004709	-3.078680	3.580273
C	0.345158	0.320062	-3.556085
C	0.345158	0.320062	3.556085
C	-0.943007	-3.367794	-3.558257
H	-2.004709	-3.078680	-3.580273
C	0.200599	0.084183	4.942876
H	-0.318266	-0.821046	5.298400
C	1.028404	1.475605	-3.119562
H	1.138901	1.657119	-2.038802
C	-2.218148	-0.609646	-2.467463
H	-2.363851	0.489442	-2.539305
H	-2.626370	-1.058344	-3.396480
C	-2.218148	-0.609646	2.467463
H	-2.626370	-1.058344	3.396480
H	-2.363851	0.489442	2.539305
C	0.028798	-2.493792	-3.025222
C	-2.876240	-1.153414	1.193652
H	-2.901463	-2.279471	1.242462
H	-3.954248	-0.841836	1.184501
C	1.554661	2.385122	-4.055993
H	2.087760	3.283112	-3.705021
C	0.200599	0.084183	-4.942876
H	-0.318266	-0.821046	-5.298400
C	1.755102	-4.158131	3.523929
H	2.813892	-4.462208	3.506779
C	1.382576	-2.906460	3.008796
H	2.152103	-2.242860	2.581807
C	1.777399	-0.517805	0.000000
C	1.028404	1.475605	3.119562
H	1.138901	1.657119	2.038802
C	-0.568679	-4.627574	4.065586
H	-1.340066	-5.299744	4.474404
C	-0.568679	-4.627574	-4.065586
H	-1.340066	-5.299744	-4.474404
C	0.725344	0.995086	-5.875414
H	0.608207	0.802407	-6.953983
C	1.382576	-2.906460	-3.008796
H	2.152103	-2.242860	-2.581807
C	0.778793	-5.024627	4.052759
H	1.069956	-6.009507	4.451233
C	1.403887	2.147137	-5.433463
H	1.818990	2.857711	-6.166021
C	0.725344	0.995086	5.875414
H	0.608207	0.802407	6.953983
C	0.778793	-5.024627	-4.052759

H	1.069956	-6.009507	-4.451233
C	1.554661	2.385122	4.055993
H	2.087760	3.283112	3.705021
C	1.403887	2.147137	5.433463
H	1.818990	2.857711	6.166021
C	1.755102	-4.158131	-3.523929
H	2.813892	-4.462208	-3.506779
H	0.009518	-2.190671	0.000000
H	-2.342323	1.626117	0.000000
N	-1.718331	3.632332	0.000000
C	-1.446203	2.298539	0.000000
O	-0.308838	1.793695	0.000000
C	-0.848287	4.748718	0.000000
C	-1.437712	6.035566	0.000000
C	0.561627	4.627450	0.000000
C	-0.631991	7.183237	0.000000
H	-2.535932	6.132277	0.000000
C	1.354367	5.788003	0.000000
H	1.019307	3.630829	0.000000
C	0.770485	7.067136	0.000000
H	-1.107381	8.176799	0.000000
H	2.451068	5.683413	0.000000
H	1.402981	7.968351	0.000000
H	-2.717609	3.859449	0.000000

1G

Frequencies, energies and thermodynamic properties:

Lowest Vibrational Mode (1/cm) = 12.4598
2nd Lowest Vibrational Mode (1/cm) = 18.5667
E(RB-P86) (a.u.) = -2238.40027056
Thermal correction to Enthalpy (a.u.) = 0.601640
Thermal correction to Gibbs Free Energy (a.u.) = 0.485115
Total Entropy (cal/Kmol) = 245.248
E(RPBE1PBE) (a.u.) = -2238.14352316

Optimised cartesian coordinates (Angstrom):

Ru	-0.001101	0.007267	0.058067
P	-2.298292	0.189778	0.364559
P	2.289332	0.241489	0.373676
N	-0.006474	0.028517	2.187271
O	-0.007287	-0.033508	-2.974819
C	-3.059445	1.841737	-0.001115
C	1.172818	0.537323	2.877638
H	1.167813	0.214901	3.952795
H	1.195992	1.664333	2.915974
C	-3.632744	2.671544	0.986465
H	-3.668529	2.348190	2.037848
C	3.549627	-0.921414	-0.338364
C	-3.533164	-0.974535	-0.390148
C	3.384257	2.842807	0.942112
H	3.273630	2.610350	2.012232

C	-4.925508	-0.761946	-0.265289
H	-5.303341	0.129098	0.262334
C	3.113558	-2.048462	-1.067921
H	2.032915	-2.207632	-1.211522
C	2.461714	0.027518	2.220155
H	2.562719	-1.068517	2.370680
H	3.377798	0.505142	2.625357
C	-2.471203	-0.084563	2.201411
H	-3.412289	0.330283	2.617835
H	-2.513471	-1.188226	2.321766
C	3.025981	1.892773	-0.038780
C	-1.212548	0.477285	2.873784
H	-1.289603	1.601712	2.918630
H	-1.199978	0.147570	3.946632
C	4.050305	-2.957352	-1.595412
H	3.701062	-3.831467	-2.168166
C	4.935369	-0.715300	-0.145168
H	5.290475	0.167703	0.411090
C	-3.564796	3.548357	-1.683973
H	-3.533932	3.886610	-2.732038
C	-3.026344	2.298161	-1.340541
H	-2.567111	1.670160	-2.121265
C	-0.001472	-0.052872	-1.787110
C	-3.067303	-2.111922	-1.085152
H	-1.981669	-2.275179	-1.178101
C	-4.163389	3.930200	0.641835
H	-4.603388	4.567511	1.425585
C	3.881884	4.105353	0.565346
H	4.155203	4.835957	1.343518
C	5.867683	-1.626685	-0.669220
H	6.945412	-1.457651	-0.513917
C	3.173759	2.233257	-1.404530
H	2.890245	1.507924	-2.184686
C	-4.133884	4.370343	-0.691922
H	-4.550432	5.354321	-0.959966
C	5.426478	-2.748989	-1.396652
H	6.159068	-3.459273	-1.812291
C	-5.835419	-1.677283	-0.821436
H	-6.918613	-1.502512	-0.719725
C	4.031013	4.431777	-0.793045
H	4.421513	5.419293	-1.086067
C	-3.981358	-3.025128	-1.643475
H	-3.608572	-3.907407	-2.188226
C	-5.364789	-2.810288	-1.512505
H	-6.079472	-3.523567	-1.953422
C	3.676323	3.489921	-1.778186
H	3.788382	3.737326	-2.845935
H	-0.014458	1.588625	0.012199
O	0.021840	-2.392363	0.252389
C	0.051328	-2.931516	1.378802

N	0.079172	-4.263877	1.584519
H	0.055400	-2.286484	2.293890
H	0.099758	-4.577943	2.558109
C	0.079099	-5.273308	0.534702
H	0.981402	-5.915605	0.603076
H	-0.819970	-5.920425	0.604530
H	0.076598	-4.760165	-0.444146

1H_Ph

Frequencies, energies and thermodynamic properties:

Lowest Vibrational Mode (1/cm) =	13.0145
2nd Lowest Vibrational Mode (1/cm) =	15.2997
E(RB-P86) (a.u.) =	-2429.98601700
Thermal correction to Enthalpy (a.u.) =	0.656255
Thermal correction to Gibbs Free Energy (a.u.) =	0.533973
Total Entropy (cal/Kmol) =	257.365
E(RPBE1PBE) (a.u.) =	-2429.71554077

Optimised cartesian coordinates (Angstrom):

Ru	0.584212	-0.041564	0.000000
P	0.800268	-0.342891	2.305762
P	0.800268	-0.342891	-2.305762
N	0.571619	-2.241790	0.000000
O	0.550598	2.974202	0.000000
C	2.463355	0.018113	3.037791
C	1.086792	-2.890420	-1.229810
H	0.792518	-3.966932	-1.244604
H	2.198598	-2.859338	-1.219625
C	3.272381	-0.961154	3.653219
H	2.927668	-2.002912	3.738816
C	-0.361716	0.385452	-3.553054
C	-0.361716	0.385452	3.553054
C	3.272381	-0.961154	-3.653219
H	2.927668	-2.002912	-3.738816
C	-0.047208	0.437504	4.930145
H	0.926043	0.066820	5.289839
C	-1.607997	0.878233	-3.105639
H	-1.851512	0.830785	-2.032051
C	0.530426	-2.185943	-2.470695
H	-0.573185	-2.307584	-2.512695
H	0.942233	-2.603645	-3.411716
C	0.530426	-2.185943	2.470695
H	0.942233	-2.603645	3.411716
H	-0.573185	-2.307584	2.512695
C	2.463355	0.018113	-3.037791
C	1.086792	-2.890420	1.229810
H	2.198598	-2.859338	1.219625
H	0.792518	-3.966932	1.244604
C	-2.529140	1.408986	-4.026887
H	-3.498965	1.789862	-3.669189
C	-0.047208	0.437504	-4.930145

H	0.926043	0.066820	-5.289839
C	4.203263	1.685554	3.471151
H	4.562568	2.724217	3.396207
C	2.945861	1.345535	2.947694
H	2.334189	2.119219	2.456253
C	0.556532	1.790624	0.000000
C	-1.607997	0.878233	3.105639
H	-1.851512	0.830785	2.032051
C	4.538607	-0.620372	4.167577
H	5.159996	-1.397420	4.640634
C	4.538607	-0.620372	-4.167577
H	5.159996	-1.397420	-4.640634
C	-0.970602	0.970722	-5.846141
H	-0.716719	1.009573	-6.917609
C	2.945861	1.345535	-2.947694
H	2.334189	2.119219	-2.456253
C	5.005519	0.701703	4.080796
H	5.995379	0.966895	4.484803
C	-2.212450	1.457313	-5.396368
H	-2.932830	1.878498	-6.115722
C	-0.970602	0.970722	5.846141
H	-0.716719	1.009573	6.917609
C	5.005519	0.701703	-4.080796
H	5.995379	0.966895	-4.484803
C	-2.529140	1.408986	4.026887
H	-3.498965	1.789862	3.669189
C	-2.212450	1.457313	5.396368
H	-2.932830	1.878498	6.115722
C	4.203263	1.685554	-3.471151
H	4.562568	2.724217	-3.396207
H	2.181887	-0.021538	0.000000
H	-0.540436	-2.334976	0.000000
N	-3.469311	-1.763131	0.000000
C	-2.095767	-1.576599	0.000000
O	-1.684533	-0.363268	0.000000
C	-4.575382	-0.881179	0.000000
C	-5.874012	-1.453855	0.000000
C	-4.456915	0.532919	0.000000
C	-7.018234	-0.643744	0.000000
H	-5.978252	-2.552102	0.000000
C	-5.613887	1.332265	0.000000
H	-3.458127	0.984446	0.000000
C	-6.898062	0.759141	0.000000
H	-8.014310	-1.115302	0.000000
H	-5.501892	2.428842	0.000000
H	-7.795965	1.396700	0.000000
H	-3.733624	-2.751777	0.000000

1H

Frequencies, energies and thermodynamic properties:

Lowest Vibrational Mode (1/cm) = 17.2418
2nd Lowest Vibrational Mode (1/cm) = 24.5148
E(RB-P86) (a.u.) = -2238.37768027
Thermal correction to Enthalpy (a.u.) = 0.600575
Thermal correction to Gibbs Free Energy (a.u.) = 0.485380
Total Entropy (cal/Kmol) = 242.448
E(RPBE1PBE) (a.u.) = -2238.12165027
Optimised cartesian coordinates (Angstrom):

Ru	-0.000066	0.006399	0.031377
P	-2.303238	0.195780	0.336334
P	2.303087	0.196305	0.336391
N	-0.000063	-0.049832	2.224909
O	0.000086	0.024471	-2.985795
C	-3.064216	1.850941	-0.002477
C	1.228011	0.445568	2.887210
H	1.239670	0.125989	3.957305
H	1.228721	1.558676	2.884711
C	-3.683653	2.641562	0.989199
H	-3.757219	2.284572	2.027761
C	3.524862	-0.975532	-0.419986
C	-3.524755	-0.976202	-0.420296
C	3.681834	2.643076	0.988906
H	3.754665	2.286744	2.027741
C	-4.910270	-0.698660	-0.454218
H	-5.294382	0.250068	-0.045988
C	3.044855	-2.189229	-0.962109
H	1.963672	-2.403138	-0.924226
C	2.468690	-0.103641	2.174627
H	2.504824	-1.209220	2.277661
H	3.411508	0.297033	2.599211
C	-2.468782	-0.104615	2.174487
H	-3.411793	0.295542	2.599126
H	-2.504410	-1.210229	2.277329
C	3.063684	1.851552	-0.002858
C	-1.228369	0.445028	2.887197
H	-1.229528	1.558137	2.884791
H	-1.239955	0.125347	3.957262
C	3.944077	-3.113508	-1.524927
H	3.563164	-4.056874	-1.948020
C	4.910444	-0.698186	-0.452748
H	5.294412	0.250275	-0.043750
C	-3.530152	3.603528	-1.648085
H	-3.465966	3.975280	-2.683114
C	-2.988384	2.349430	-1.324967
H	-2.493050	1.752695	-2.107888
C	-0.000005	0.008746	-1.801485
C	-3.044541	-2.190252	-0.961459
H	-1.963392	-2.404253	-0.922842
C	-4.216667	3.904586	0.665486
H	-4.692740	4.511391	1.452213

C 4.214507 3.906175 0.664927
H 4.689598 4.513665 1.451719
C 5.803940 -1.625516 -1.016500
H 6.882350 -1.400703 -1.040214
C 2.988770 2.349230 -1.325698
H 2.494465 1.751787 -2.108733
C -4.144313 4.386990 -0.651895
H -4.562797 5.374335 -0.903767
C 5.322274 -2.833951 -1.554149
H 6.023654 -3.556849 -2.000704
C -5.803499 -1.626160 -1.018118
H -6.881857 -1.401191 -1.042731
C 4.143066 4.387779 -0.652798
H 4.561264 5.375190 -0.904883
C -3.943503 -3.114702 -1.524408
H -3.562421 -4.058338 -1.946746
C -5.321641 -2.834964 -1.554758
H -6.022812 -3.557993 -2.001428
C 3.530166 3.603426 -1.649061
H 3.466702 3.974533 -2.684366
H -0.000253 1.606027 0.042608
O 0.000335 -2.271924 0.324009
C 0.000336 -2.676230 1.547694
N 0.001023 -4.025203 1.739737
H 0.000137 -1.193473 2.286576
H 0.000979 -4.332691 2.714199
C 0.001849 -5.070006 0.714905
H 0.901934 -5.717555 0.793065
H -0.896845 -5.719408 0.793583
H 0.001078 -4.580913 -0.276980

1I_Ph
Frequencies, energies and thermodynamic properties:
Lowest Vibrational Mode (1/cm) = 15.7437
2nd Lowest Vibrational Mode (1/cm) = 16.8924
E(RB-P86) (a.u.) = -2430.01313913
Thermal correction to Enthalpy (a.u.) = 0.657597
Thermal correction to Gibbs Free Energy (a.u.) = 0.536789
Total Entropy (cal/Kmol) = 254.260
E(RPBE1PBE) (a.u.) = -2429.74904552
Optimised cartesian coordinates (Angstrom):
Ru -0.255279 -0.297595 -0.014667
P 1.702431 -1.503549 0.286369
P -2.448246 0.476078 0.333506
N -0.099028 0.024868 2.207981
O -0.556671 -0.861713 -2.954172
C 1.647004 -3.332778 -0.015168
C -1.420604 0.175661 2.888222
H -1.273409 0.559556 3.923061
H -1.861836 -0.839355 2.968607

C	1.291136	-4.253360	0.994624
H	1.078274	-3.910647	2.019032
C	-3.344417	1.842775	-0.550413
C	3.331591	-1.067378	-0.490276
C	-4.577588	-1.129202	1.455795
H	-4.476490	-0.560950	2.393197
C	4.530665	-1.684669	-0.062245
H	4.511848	-2.444997	0.735551
C	-2.668492	2.617303	-1.517991
H	-1.601244	2.422367	-1.725234
C	-2.345316	1.096086	2.095184
H	-1.925482	2.122536	2.028934
H	-3.337880	1.185744	2.581867
C	2.031867	-1.293695	2.128323
H	2.556458	-2.170635	2.560110
H	2.732781	-0.436197	2.226066
C	-3.756757	-0.844074	0.343575
C	0.725681	-1.024848	2.877009
H	0.101772	-1.940847	2.904805
H	0.938311	-0.732675	3.930510
C	-3.349250	3.654473	-2.185201
H	-2.816354	4.254375	-2.940560
C	-4.703354	2.115145	-0.263541
H	-5.248975	1.505710	0.474833
C	1.795041	-5.184281	-1.609045
H	1.992947	-5.542010	-2.632010
C	1.898030	-3.812610	-1.321070
H	2.180786	-3.110660	-2.122087
C	-0.433722	-0.639371	-1.800934
C	3.377448	-0.113886	-1.528683
H	2.445808	0.372128	-1.857868
C	1.195387	-5.626591	0.705537
H	0.922046	-6.332659	1.505808
C	-5.544417	-2.151830	1.387515
H	-6.172112	-2.365296	2.267479
C	-5.372855	3.155759	-0.928178
H	-6.430270	3.361239	-0.696303
C	-3.927265	-1.599596	-0.840330
H	-3.285209	-1.402039	-1.713693
C	1.445181	-6.095966	-0.596275
H	1.367327	-7.171346	-0.821510
C	-4.696930	3.926392	-1.893736
H	-5.225281	4.737601	-2.419999
C	5.755434	-1.340961	-0.657959
H	6.684911	-1.822886	-0.314959
C	-5.710365	-2.891594	0.205214
H	-6.467982	-3.689473	0.152438
C	4.604966	0.220706	-2.131025
H	4.628702	0.964564	-2.942979
C	5.793911	-0.388760	-1.695047

H	6.754817	-0.123688	-2.164114
C	-4.899884	-2.609112	-0.911449
H	-5.020614	-3.184934	-1.842831
H	-0.940634	-1.756191	0.444498
O	0.417745	2.384522	-1.364732
C	0.651702	1.631667	-0.398335
N	1.602186	2.103359	0.561919
H	0.386790	0.933826	2.296410
H	1.973923	1.378187	1.179096
C	2.326910	3.311465	0.629170
C	3.393321	3.396139	1.563915
C	2.032750	4.451530	-0.163538
C	4.136844	4.576240	1.706608
H	3.637340	2.518449	2.186346
C	2.787459	5.627045	-0.008976
H	1.221035	4.386799	-0.896941
C	3.840779	5.705571	0.919819
H	4.958398	4.610819	2.440537
H	2.542080	6.500703	-0.635085
H	4.424882	6.632705	1.029585

1I

Frequencies, energies and thermodynamic properties:

Lowest Vibrational Mode (1/cm) = 16.1367
2nd Lowest Vibrational Mode (1/cm) = 20.2706
E(RB-P86) (a.u.) = -2238.40515582
Thermal correction to Enthalpy (a.u.) = 0.602689
Thermal correction to Gibbs Free Energy (a.u.) = 0.490048
Total Entropy (cal/Kmol) = 237.073
E(RPBE1PBE) (a.u.) = -2238.15717853

Optimised cartesian coordinates (Angstrom):

Ru	0.002224	-0.170727	0.085994
P	-2.246038	0.305162	0.367042
P	2.331973	-0.066133	0.361701
N	0.025741	-0.155889	2.327657
O	0.009343	-0.143450	-2.924437
C	-2.882986	1.938941	-0.239035
C	1.335992	0.223655	2.930845
H	1.352305	-0.051614	4.009986
H	1.416403	1.329186	2.870438
C	-2.767126	3.114358	0.535450
H	-2.335496	3.079092	1.547803
C	3.629525	-1.140327	-0.423976
C	-3.621478	-0.827681	-0.161779
C	3.773029	2.321563	1.133358
H	3.898742	1.888105	2.137468
C	-4.944157	-0.640581	0.304389
H	-5.181673	0.193442	0.984770
C	3.239100	-2.283004	-1.156134
H	2.166835	-2.545129	-1.225798

C	2.499285	-0.432916	2.189027
H	2.454695	-1.539320	2.276662
H	3.475809	-0.114584	2.607823
C	-2.428329	0.348522	2.243789
H	-3.197482	1.079680	2.566582
H	-2.804479	-0.652250	2.548127
C	3.087167	1.616168	0.121369
C	-1.083463	0.653432	2.907155
H	-0.810443	1.717261	2.752966
H	-1.152557	0.482373	4.005943
C	4.218616	-3.100922	-1.752207
H	3.907194	-3.990791	-2.323060
C	5.004233	-0.827297	-0.300002
H	5.322402	0.070747	0.253405
C	-3.838919	3.269099	-2.057568
H	-4.255786	3.322777	-3.075894
C	-3.422928	2.029017	-1.542026
H	-3.523243	1.123341	-2.161458
C	0.001700	-0.153697	-1.742358
C	-3.346479	-1.888525	-1.050360
H	-2.315931	-2.038162	-1.409148
C	-3.192569	4.351982	0.021321
H	-3.101390	5.258428	0.640809
C	4.311243	3.598321	0.876856
H	4.837380	4.137519	1.680868
C	5.974862	-1.651720	-0.893279
H	7.042621	-1.399768	-0.790082
C	2.958026	2.211938	-1.155463
H	2.412296	1.682942	-1.953396
C	-3.727718	4.433763	-1.276933
H	-4.057047	5.404591	-1.679840
C	5.583345	-2.789660	-1.624275
H	6.345245	-3.431074	-2.095882
C	-5.969250	-1.509970	-0.104340
H	-6.995238	-1.359315	0.267836
C	4.182454	4.178193	-0.395810
H	4.605455	5.175424	-0.595894
C	-4.377703	-2.751464	-1.467388
H	-4.152305	-3.573062	-2.165629
C	-5.687664	-2.566005	-0.992612
H	-6.493795	-3.243697	-1.315942
C	3.506805	3.477660	-1.414026
H	3.399200	3.924375	-2.415296
H	0.117949	1.497849	0.239931
O	0.358511	-3.178226	-0.560484
C	-0.161899	-2.327183	0.193191
N	-0.875840	-2.848255	1.302239
H	-0.138844	-1.160761	2.532201
H	-1.653473	-2.264818	1.622977
C	-1.103145	-4.285843	1.449252

H -0.143985 -4.823417 1.323584
H -1.503933 -4.497178 2.460769
H -1.813876 -4.692123 0.693010

1Ja
Frequencies, energies and thermodynamic properties:
Lowest Vibrational Mode (1/cm) = 16.4493
2nd Lowest Vibrational Mode (1/cm) = 16.7468
E(RB-P86) (a.u.) = -2143.78539299
Thermal correction to Enthalpy (a.u.) = 0.554385
Thermal correction to Gibbs Free Energy (a.u.) = 0.446732
Total Entropy (cal/Kmol) = 226.575
E(RPBE1PBE) (a.u.) = -2143.53606492
Optimised cartesian coordinates (Angstrom):

Ru	0.000000	-0.299708	0.208849
P	-2.303648	0.002984	0.448676
P	2.303648	0.002986	0.448676
N	0.000000	-0.003532	2.417516
O	0.000000	-0.762304	-2.768198
C	-3.023419	1.595694	-0.169937
C	1.236302	0.646856	2.941580
H	1.273510	0.555709	4.050969
H	1.171068	1.726596	2.696757
C	-3.562300	2.589396	0.674579
H	-3.593075	2.446841	1.765551
C	3.590386	-1.214711	-0.102501
C	-3.590385	-1.214712	-0.102504
C	3.562298	2.589399	0.674573
H	3.593071	2.446847	1.765545
C	-4.966493	-0.948586	0.090782
H	-5.289212	-0.000157	0.550286
C	3.197174	-2.423381	-0.716372
H	2.128019	-2.639481	-0.862863
C	2.481956	0.020964	2.312947
H	2.572418	-1.047080	2.607111
H	3.406841	0.527559	2.656024
C	-2.481957	0.020958	2.312947
H	-3.406843	0.527551	2.656026
H	-2.572416	-1.047086	2.607110
C	3.023418	1.595694	-0.169940
C	-1.236304	0.646852	2.941581
H	-1.171072	1.726593	2.696760
H	-1.273511	0.555704	4.050970
C	4.169772	-3.355804	-1.125144
H	3.853811	-4.295225	-1.606023
C	4.966493	-0.948585	0.090788
H	5.289212	-0.000155	0.550293
C	-3.516735	3.012165	-2.104460
H	-3.495520	3.171746	-3.194229
C	-3.003576	1.821536	-1.566749

H	-2.575529	1.061686	-2.240646
C	0.000000	-0.574652	-1.601332
C	-3.197173	-2.423382	-0.716374
H	-2.128017	-2.639483	-0.862863
C	-4.069146	3.786950	0.133340
H	-4.483272	4.554534	0.806445
C	4.069144	3.786952	0.133331
H	4.483269	4.554539	0.806435
C	5.932229	-1.885595	-0.312004
H	7.001376	-1.671142	-0.154518
C	3.003576	1.821533	-1.566753
H	2.575531	1.061681	-2.240649
C	-4.049676	4.000911	-1.254826
H	-4.447300	4.937599	-1.676476
C	5.535041	-3.090907	-0.922454
H	6.293883	-3.822090	-1.243844
C	-5.932228	-1.885595	-0.312013
H	-7.001375	-1.671142	-0.154529
C	4.049675	4.000910	-1.254836
H	4.447299	4.937596	-1.676488
C	-4.169770	-3.355805	-1.125149
H	-3.853808	-4.295226	-1.606028
C	-5.535039	-3.090908	-0.922462
H	-6.293881	-3.822090	-1.243854
C	3.516735	3.012161	-2.104467
H	3.495521	3.171739	-3.194236
H	0.000000	1.399673	0.171496
H	0.000001	-0.991127	2.753037
C	0.000001	-2.321253	0.690952
H	0.000002	-2.482889	1.859369
O	0.000000	-3.376178	0.049705

1J

Frequencies, energies and thermodynamic properties:

Lowest Vibrational Mode (1/cm) = 15.6014
2nd Lowest Vibrational Mode (1/cm) = 20.0047
E(RB-P86) (a.u.) = -2143.79413653
Thermal correction to Enthalpy (a.u.) = 0.555375
Thermal correction to Gibbs Free Energy (a.u.) = 0.448966
Total Entropy (cal/Kmol) = 223.956
E(RPBE1PBE) (a.u.) = -2143.54324679

Optimised cartesian coordinates (Angstrom):

Ru	0.000022	-0.237167	0.121654
P	-2.306065	0.003747	0.397232
P	2.306093	0.003596	0.397270
N	0.000024	-0.027000	2.338055
O	0.000144	-0.642071	-2.866586
C	-3.097029	1.584409	-0.159632
C	1.233965	0.573207	2.905378
H	1.261025	0.420213	4.009256

H	1.199002	1.668023	2.723562
C	-3.585303	2.565163	0.729798
H	-3.538590	2.412126	1.818800
C	3.543120	-1.251175	-0.184181
C	-3.543160	-1.251081	-0.183953
C	3.585460	2.564914	0.730227
H	3.538869	2.411633	1.819199
C	-4.924000	-1.074934	0.068240
H	-5.282090	-0.170345	0.586260
C	3.109351	-2.404587	-0.870439
H	2.036645	-2.541370	-1.075118
C	2.474151	-0.049479	2.260845
H	2.538964	-1.128755	2.515143
H	3.408113	0.426113	2.622348
C	-2.474075	-0.049056	2.260827
H	-3.407973	0.426712	2.622261
H	-2.539050	-1.128293	2.515253
C	3.097033	1.584367	-0.159353
C	-1.233795	0.573507	2.905288
H	-1.198640	1.668293	2.723330
H	-1.260919	0.420669	4.009189
C	4.039537	-3.372796	-1.292600
H	3.689310	-4.267567	-1.831217
C	4.924031	-1.074783	0.067447
H	5.282201	-0.170034	0.585130
C	-3.732272	3.014905	-2.041708
H	-3.786713	3.185141	-3.128714
C	-3.173719	1.823890	-1.551948
H	-2.788368	1.073398	-2.261411
C	0.000087	-0.475998	-1.694223
C	-3.109485	-2.404265	-0.870650
H	-2.036833	-2.540842	-1.075750
C	-4.139217	3.762312	0.236375
H	-4.514133	4.519170	0.943738
C	4.139361	3.762143	0.236990
H	4.514393	4.518839	0.944466
C	5.849730	-2.045939	-0.348474
H	6.922612	-1.901379	-0.144112
C	3.173565	1.824137	-1.551627
H	2.788074	1.073823	-2.261199
C	-4.215713	3.989837	-1.147792
H	-4.649908	4.926443	-1.531893
C	5.408927	-3.196820	-1.029817
H	6.136901	-3.954812	-1.360045
C	-5.849715	-2.046121	-0.347558
H	-6.922543	-1.901772	-0.142763
C	4.215705	3.989951	-1.147140
H	4.649901	4.926617	-1.531094
C	-4.039696	-3.372506	-1.292697
H	-3.689550	-4.267104	-1.831654

C	-5.409006	-3.196781	-1.029343
H	-6.137007	-3.954795	-1.359459
C	3.732116	3.015231	-2.041204
H	3.786428	3.185695	-3.128179
H	-0.000071	1.456262	0.060661
H	-0.000089	-1.063625	2.542516
C	-0.000093	-2.264832	0.635932
H	-0.000109	-3.088923	-0.152934
O	-0.000219	-2.696212	1.814045

1TSBBa_Ph

Frequencies, energies and thermodynamic properties:

Lowest Vibrational Mode (1/cm) = -56.9232
2nd Lowest Vibrational Mode (1/cm) = 14.3956
E(RB-P86) (a.u.) = -2428.79231693
Thermal correction to Enthalpy (a.u.) = 0.633894
Thermal correction to Gibbs Free Energy (a.u.) = 0.516911
Total Entropy (cal/Kmol) = 246.210
E(RPBE1PBE) (a.u.) = -2428.53135992

Optimised cartesian coordinates (Angstrom):

Ru	-0.388221	0.430540	0.063763
P	1.663473	1.517031	-0.204866
P	-2.492150	-0.536263	-0.290334
N	0.077892	-0.457223	-1.914543
O	-1.085395	1.740792	2.686603
C	1.602095	3.365513	-0.211463
C	-1.167136	-0.597326	-2.763737
H	-0.927870	-1.234655	-3.643904
H	-1.378454	0.421771	-3.142854
C	1.175803	4.065299	-1.363267
H	0.938526	3.522538	-2.291978
C	-3.033530	-1.995702	0.701620
C	3.095787	1.132284	0.895851
C	-4.180404	1.537299	-1.239089
H	-3.543245	1.544891	-2.138094
C	4.377728	1.672050	0.639665
H	4.531719	2.359941	-0.207566
C	-2.315743	-2.347134	1.864465
H	-1.444819	-1.744739	2.169605
C	-2.419324	-1.138840	-2.062786
H	-2.394904	-2.240923	-2.008351
H	-3.327720	-0.822422	-2.615884
C	2.219873	0.991009	-1.910494
H	2.662103	1.844088	-2.465311
H	2.989377	0.217840	-1.736606
C	-3.940788	0.611651	-0.197449
C	1.058027	0.383144	-2.701682
H	0.456283	1.197611	-3.153277
H	1.462440	-0.234419	-3.533154
C	-2.703986	-3.462922	2.628440

H	-2.135924	-3.730108	3.533444
C	-4.147311	-2.774222	0.311315
H	-4.722769	-2.509334	-0.590506
C	1.748621	5.491506	0.989689
H	1.974605	6.045049	1.914881
C	1.883641	4.092215	0.967589
H	2.215841	3.564036	1.875346
C	-0.800485	1.204577	1.677090
C	2.917168	0.259364	1.990214
H	1.920311	-0.165219	2.192749
C	1.044475	5.464830	-1.338310
H	0.716633	5.996998	-2.245395
C	-5.231914	2.464535	-1.139617
H	-5.410659	3.175404	-1.961916
C	-4.530311	-3.890176	1.073792
H	-5.397262	-4.493854	0.761613
C	-4.765034	0.641762	0.949681
H	-4.592172	-0.070307	1.771841
C	1.330210	6.181650	-0.162222
H	1.226708	7.278035	-0.143697
C	-3.809585	-4.235742	2.233109
H	-4.111761	-5.111586	2.828914
C	5.462270	1.338815	1.467854
H	6.458111	1.762077	1.261220
C	-6.051238	2.485756	0.004072
H	-6.874456	3.213415	0.081297
C	4.004848	-0.069673	2.819958
H	3.855599	-0.751906	3.671540
C	5.276906	0.468399	2.559340
H	6.128772	0.209518	3.207912
C	-5.813274	1.574233	1.047857
H	-6.449332	1.584184	1.947220
H	-1.000244	1.697721	-0.607661
N	1.938105	-1.845315	-1.365878
C	0.619896	-1.841368	-1.493273
O	-0.257864	-2.712319	-1.297520
C	2.648656	-2.996719	-1.021855
C	4.035965	-2.827477	-0.732322
C	2.133849	-4.328723	-0.967600
C	4.860075	-3.913173	-0.405935
H	4.449935	-1.806981	-0.772626
C	2.970501	-5.411555	-0.647500
H	1.068579	-4.487808	-1.179582
C	4.335439	-5.220611	-0.361670
H	5.926703	-3.738400	-0.186553
H	2.543044	-6.428458	-0.619109
H	4.981679	-6.076457	-0.108830

1TSBBa

Frequencies, energies and thermodynamic properties:

Lowest Vibrational Mode (1/cm) = -85.7325
2nd Lowest Vibrational Mode (1/cm) = 18.0837
E(RB-P86) (a.u.) = -2237.17698578
Thermal correction to Enthalpy (a.u.) = 0.578844
Thermal correction to Gibbs Free Energy (a.u.) = 0.469545
Total Entropy (cal/Kmol) = 230.038
E(RPBE1PBE) (a.u.) = -2236.93146243

Optimised cartesian coordinates (Angstrom):

Ru	0.009423	0.005269	0.007359
P	-2.309405	0.099593	0.289199
P	2.325798	0.105778	0.328437
N	0.007112	-0.670659	2.107297
O	0.035120	1.056392	-2.817653
C	-3.096099	1.758873	0.064718
C	1.188593	-0.135630	2.881057
H	1.259638	-0.682595	3.847911
H	0.935325	0.917134	3.117221
C	-3.018128	2.733486	1.085664
H	-2.545355	2.494576	2.051556
C	3.442972	-1.104397	-0.504658
C	-3.414111	-1.034551	-0.662014
C	2.834168	2.848565	0.806441
H	2.180643	2.729692	1.685860
C	-4.784529	-1.166558	-0.339345
H	-5.224149	-0.568763	0.475477
C	2.975794	-1.805730	-1.636708
H	1.950766	-1.627925	-2.000391
C	2.546315	-0.176934	2.167662
H	3.008307	-1.174158	2.267444
H	3.223280	0.590191	2.597361
C	-2.553926	-0.377649	2.079136
H	-3.343382	0.239240	2.556042
H	-2.859616	-1.439851	2.052622
C	3.120751	1.741281	-0.025711
C	-1.238068	-0.260982	2.854227
H	-1.076946	0.794859	3.153891
H	-1.305341	-0.869479	3.782653
C	3.807426	-2.731526	-2.292586
H	3.431775	-3.275496	-3.173616
C	4.756284	-1.339457	-0.037272
H	5.141349	-0.796021	0.840640
C	-4.215057	3.394332	-1.369933
H	-4.683241	3.647328	-2.334483
C	-3.697180	2.102993	-1.167222
H	-3.766747	1.356633	-1.974370
C	0.023248	0.611473	-1.725971
C	-2.872867	-1.803574	-1.714098
H	-1.804433	-1.700990	-1.964958
C	-3.539854	4.022789	0.880656
H	-3.477062	4.770133	1.687559

C 3.370517 4.114764 0.518324
H 3.142716 4.966355 1.178982
C 5.583222 -2.268190 -0.691324
H 6.603767 -2.448604 -0.317835
C 3.943152 1.931032 -1.158118
H 4.175749 1.081614 -1.819013
C -4.139167 4.357013 -0.347372
H -4.547117 5.367587 -0.506999
C 5.110439 -2.965281 -1.819475
H 5.760610 -3.693674 -2.329509
C -5.595920 -2.059452 -1.058824
H -6.662071 -2.159022 -0.800010
C 4.191762 4.295224 -0.610378
H 4.609958 5.288680 -0.837025
C -3.688790 -2.694114 -2.435868
H -3.257007 -3.289636 -3.255675
C -5.049501 -2.823806 -2.107883
H -5.688527 -3.522680 -2.670605
C 4.473510 3.201726 -1.447475
H 5.114225 3.333953 -2.333845
H 0.004056 1.503401 0.441139
N -1.021962 -2.814807 1.848278
C 0.135217 -2.190638 1.864926
O 1.310255 -2.605130 1.681582
C -0.921937 -4.241809 1.590876
H -0.237917 -4.768009 2.301565
H -1.924015 -4.714449 1.672972
H -0.528219 -4.473873 0.569943

1TSCE_Ph
Frequencies, energies and thermodynamic properties:
Lowest Vibrational Mode (1/cm) = -587.5489
2nd Lowest Vibrational Mode (1/cm) = 11.9068
E(RB-P86) (a.u.) = -2431.19391349
Thermal correction to Enthalpy (a.u.) = 0.673331
Thermal correction to Gibbs Free Energy (a.u.) = 0.549405
Total Entropy (cal/Kmol) = 260.824
E(RPBE1PBE) (a.u.) = -2430.92669241
Optimised cartesian coordinates (Angstrom):
Ru -0.572163 0.355589 0.021557
P 1.239069 1.809739 -0.278986
P -2.542985 -0.850772 -0.322309
N -0.406473 0.160384 -2.103207
O -0.759155 0.503539 3.039890
C 0.960085 3.644857 -0.148280
C -1.643831 -0.170792 -2.849279
H -1.383189 -0.509183 -3.880862
H -2.273800 0.741571 -2.966537
C 1.993288 4.545565 -0.499047
H 2.972930 4.166821 -0.833269

C	-2.900961	-2.462897	0.514460
C	2.831832	1.607047	0.647896
C	-4.929933	0.509693	-1.171998
H	-4.656700	0.264815	-2.209544
C	4.091991	1.552592	0.009185
H	4.169693	1.608517	-1.087079
C	-1.952623	-3.020170	1.398363
H	-1.019694	-2.473281	1.607733
C	-2.418465	-1.281384	-2.133486
H	-1.829415	-2.222202	-2.161130
H	-3.403974	-1.483370	-2.599565
C	1.668342	1.510099	-2.070985
H	2.212518	2.361923	-2.526042
H	2.328174	0.616586	-2.084617
C	-4.125279	0.082535	-0.093760
C	0.368454	1.201796	-2.817889
H	-0.244828	2.126117	-2.934251
H	0.611231	0.851076	-3.849543
C	-2.199002	-4.261866	2.013056
H	-1.453627	-4.686559	2.703914
C	-4.104008	-3.160745	0.257970
H	-4.861290	-2.730851	-0.417655
C	-0.477395	5.548715	0.390303
H	-1.446170	5.935918	0.744060
C	-0.274474	4.158881	0.300705
H	-1.078897	3.459176	0.577584
C	-0.671599	0.432292	1.863770
C	2.773305	1.519127	2.059367
H	1.798852	1.547774	2.571951
C	1.787124	5.932271	-0.413923
H	2.598892	6.622841	-0.692701
C	-6.098653	1.258513	-0.934282
H	-6.716658	1.585183	-1.785696
C	-4.345020	-4.402134	0.869519
H	-5.283700	-4.940299	0.662446
C	-4.510112	0.419527	1.225812
H	-3.889546	0.099732	2.078807
C	0.550339	6.437202	0.031072
H	0.391079	7.525000	0.100582
C	-3.392892	-4.954611	1.747749
H	-3.585799	-5.926480	2.229090
C	5.268428	1.420255	0.769707
H	6.243207	1.377365	0.258730
C	-6.476721	1.583986	0.379075
H	-7.392302	2.168115	0.562725
C	3.950835	1.390361	2.815322
H	3.889284	1.323707	3.913018
C	5.201578	1.341214	2.172061
H	6.124355	1.237252	2.764338
C	-5.679356	1.160417	1.459847

H	-5.968696	1.410920	2.492797
H	-1.511989	1.622038	-0.053015
H	0.509301	-1.327225	-0.033436
H	0.309298	-0.925695	-2.127644
O	0.908101	-1.965740	-2.004026
C	1.157123	-2.075223	-0.682621
H	0.842808	-3.070761	-0.270055
N	2.531005	-1.787188	-0.253739
H	2.646722	-1.064634	0.461204
C	3.543459	-2.733438	-0.338416
C	4.741387	-2.573733	0.413809
C	3.429758	-3.873056	-1.183517
C	5.776322	-3.514937	0.323345
H	4.847215	-1.696474	1.073418
C	4.473108	-4.810610	-1.255099
H	2.529583	-3.998442	-1.803053
C	5.654491	-4.646292	-0.508144
H	6.691999	-3.363767	0.918710
H	4.359533	-5.684116	-1.918473
H	6.468708	-5.384661	-0.574299

1TSCE

Frequencies, energies and thermodynamic properties:

Lowest Vibrational Mode (1/cm) = -592.0076
2nd Lowest Vibrational Mode (1/cm) = 8.5156
E(RB-P86) (a.u.) = -2239.57783858
Thermal correction to Enthalpy (a.u.) = 0.618535
Thermal correction to Gibbs Free Energy (a.u.) = 0.503319
Total Entropy (cal/Kmol) = 242.491
E(RPBE1PBE) (a.u.) = -2239.32524707

Optimised cartesian coordinates (Angstrom):

Ru	0.072251	-0.012331	-0.011092
P	-2.238262	0.208911	0.258629
P	2.370618	0.133853	0.354357
N	0.028031	-0.152008	2.132015
O	0.200398	0.048055	-3.034337
C	-2.912713	1.932007	0.129581
C	1.187942	0.417150	2.856703
H	1.169306	0.082116	3.922060
H	1.116434	1.529449	2.876749
C	-3.637410	2.558839	1.166496
H	-3.831248	2.032910	2.113886
C	3.573820	-1.112119	-0.300877
C	-3.495218	-0.768830	-0.691218
C	3.309603	2.793382	0.840351
H	2.984234	2.663383	1.883958
C	-4.866905	-0.703805	-0.355197
H	-5.207348	-0.056124	0.468989
C	3.113147	-2.166909	-1.116587
H	2.043761	-2.220602	-1.375520

C	2.501930	-0.037952	2.212534
H	2.645764	-1.124765	2.384573
H	3.382563	0.494909	2.624769
C	-2.442054	-0.324865	2.028230
H	-3.408029	-0.023342	2.481325
H	-2.408019	-1.436852	1.966666
C	3.178832	1.739088	-0.090312
C	-1.238403	0.198057	2.817779
H	-1.307150	1.303696	2.951437
H	-1.245716	-0.244598	3.843259
C	4.011175	-3.138478	-1.596711
H	3.641850	-3.955943	-2.236049
C	4.947747	-1.039285	0.027330
H	5.325397	-0.214273	0.653160
C	-3.174704	3.947258	-1.235314
H	-2.989320	4.486699	-2.177755
C	-2.680835	2.643164	-1.071492
H	-2.101820	2.172489	-1.882389
C	0.143805	0.005970	-1.853991
C	-3.087954	-1.586778	-1.766649
H	-2.021952	-1.631757	-2.040175
C	-4.121887	3.871186	1.004019
H	-4.681112	4.349507	1.823921
C	3.855825	4.028584	0.445163
H	3.954625	4.840222	1.183500
C	5.840979	-2.013520	-0.448117
H	6.909030	-1.951002	-0.185348
C	3.599401	1.946819	-1.424618
H	3.504124	1.136136	-2.165103
C	-3.894953	4.566416	-0.195690
H	-4.275651	5.592440	-0.321076
C	5.374069	-3.064248	-1.261477
H	6.077160	-3.825016	-1.636213
C	-5.809896	-1.457009	-1.074647
H	-6.875793	-1.402317	-0.801541
C	4.274901	4.225758	-0.882051
H	4.703117	5.192903	-1.189525
C	-4.034853	-2.334883	-2.490762
H	-3.705135	-2.966722	-3.330792
C	-5.395745	-2.274055	-2.143781
H	-6.137210	-2.860225	-2.709550
C	4.145085	3.180731	-1.815995
H	4.471826	3.326082	-2.857980
H	0.116070	1.571141	-0.026383
H	0.011449	-1.997308	0.175806
H	0.163764	-1.433627	2.234199
O	0.355248	-2.629981	2.166325
C	-0.021321	-2.939591	0.911831
H	0.712569	-3.632974	0.406958
N	-1.403458	-3.424207	0.802974

H	-1.672943	-3.444272	-0.190938
C	-1.591993	-4.749326	1.394584
H	-1.382417	-4.700748	2.482160
H	-2.644330	-5.075007	1.264453
H	-0.927924	-5.543114	0.962240

1TSDE

Frequencies, energies and thermodynamic properties:

Lowest Vibrational Mode (1/cm) = -27.9859
2nd Lowest Vibrational Mode (1/cm) = 16.5058
E(RB-P86) (a.u.) = -2142.55970102
Thermal correction to Enthalpy (a.u.) = 0.530702
Thermal correction to Gibbs Free Energy (a.u.) = 0.421126
Total Entropy (cal/Kmol) = 230.622
E(RPBE1PBE) (a.u.) = -2142.31147917

Optimised cartesian coordinates (Angstrom):

Ru	0.039765	-0.179037	0.174775
P	-2.262051	0.121510	0.409846
P	2.345626	0.037621	0.436215
N	0.034047	0.422920	2.114937
O	0.073972	-1.906005	-2.321239
C	-2.999114	1.750572	-0.109297
C	1.240016	0.866602	2.823833
H	1.138190	0.656587	3.917297
H	1.369240	1.977630	2.743888
C	-2.153194	2.852041	-0.364966
H	-1.060733	2.712382	-0.316807
C	3.575272	-1.272918	-0.020467
C	-3.505704	-1.100038	-0.200986
C	3.788265	2.531466	0.508788
H	3.937119	2.373175	1.587632
C	-4.375199	-1.810992	0.655903
H	-4.355053	-1.640063	1.742881
C	3.155157	-2.621861	-0.005087
H	2.106023	-2.858920	0.233473
C	2.489552	0.158910	2.291243
H	2.505255	-0.897308	2.634238
H	3.434420	0.632222	2.628412
C	-2.421461	0.154395	2.267890
H	-3.365541	0.631548	2.602350
H	-2.413876	-0.904316	2.601881
C	3.087738	1.582878	-0.267833
C	-1.179305	0.870746	2.809082
H	-1.312585	1.981007	2.721087
H	-1.088932	0.667780	3.904633
C	4.062555	-3.654128	-0.298968
H	3.721747	-4.701581	-0.284843
C	4.916976	-0.972978	-0.346181
H	5.258633	0.073776	-0.370074
C	-4.940784	3.181706	-0.519023

H	-6.033794	3.304654	-0.583463
C	-4.399942	1.926961	-0.189337
H	-5.075249	1.077546	0.001832
C	0.053568	-1.219202	-1.357646
C	-3.564568	-1.342759	-1.593979
H	-2.888767	-0.800218	-2.274733
C	-2.697087	4.108225	-0.689786
H	-2.025238	4.958628	-0.888015
C	4.307990	3.696849	-0.087253
H	4.846698	4.430761	0.533093
C	5.821530	-2.007546	-0.645174
H	6.864900	-1.762168	-0.900186
C	2.919922	1.822297	-1.652872
H	2.360743	1.098375	-2.267479
C	-4.090914	4.275432	-0.769000
H	-4.516580	5.257493	-1.029871
C	5.397621	-3.348657	-0.621073
H	6.107418	-4.156797	-0.859037
C	-5.284440	-2.747284	0.127962
H	-5.956594	-3.294915	0.807691
C	4.143818	3.921372	-1.464505
H	4.552924	4.832473	-1.929189
C	-4.476321	-2.273411	-2.117469
H	-4.510583	-2.451900	-3.203908
C	-5.337798	-2.980238	-1.256927
H	-6.049620	-3.713838	-1.667373
C	3.451222	2.977841	-2.247560
H	3.316978	3.146966	-3.327813
H	0.093976	1.003567	-0.865066
O	-0.804488	-4.036837	0.180909
C	-0.711809	-3.253213	1.014778

1TSEBa

Frequencies, energies and thermodynamic properties:

Lowest Vibrational Mode (1/cm) = -214.0951

2nd Lowest Vibrational Mode (1/cm) = 16.5706

E(RB-P86) (a.u.) = -2237.17064673

Thermal correction to Enthalpy (a.u.) = 0.576905

Thermal correction to Gibbs Free Energy (a.u.) = 0.460139

Total Entropy (cal/Kmol) = 245.754

E(RPBE1PBE) (a.u.) = -2236.91066600

Optimised cartesian coordinates (Angstrom):

Ru	-0.000493	-0.057207	-0.109032
P	-2.300592	0.084652	0.227730
P	2.299520	0.087626	0.226870
N	0.000068	-0.389010	1.975175
O	-0.001324	0.230818	-3.127920
C	-3.049146	1.776025	0.134984
C	1.198207	-0.112346	2.762139
H	1.169356	-0.700240	3.715979

H	1.247711	0.965664	3.076263
C	-3.470192	2.495052	1.274402
H	-3.400329	2.046708	2.277156
C	3.526393	-0.944779	-0.705303
C	-3.526858	-0.948321	-0.704617
C	3.466009	2.500441	1.271308
H	3.396285	2.053076	2.274510
C	-4.919535	-0.746929	-0.565939
H	-5.299659	0.064885	0.075308
C	3.058534	-1.980556	-1.542864
H	1.973488	-2.134654	-1.654203
C	2.472295	-0.487957	1.995087
H	2.562732	-1.592614	1.922594
H	3.396862	-0.112968	2.480279
C	-2.471938	-0.492402	1.995609
H	-3.397076	-0.119450	2.481281
H	-2.560564	-1.597168	1.922258
C	3.046236	1.779715	0.132494
C	-1.198367	-0.114934	2.762590
H	-1.249808	0.962823	3.077276
H	-1.168236	-0.703301	3.716108
C	3.969399	-2.805251	-2.228533
H	3.593799	-3.608186	-2.882718
C	4.918940	-0.742048	-0.567283
H	5.298577	0.070532	0.073280
C	-3.664985	3.688382	-1.264426
H	-3.738813	4.150627	-2.261688
C	-3.147383	2.389670	-1.136690
H	-2.812569	1.847025	-2.035885
C	-0.000959	0.093680	-1.951014
C	-3.058389	-1.983102	-1.543067
H	-1.973260	-2.136168	-1.654919
C	-3.981792	3.800750	1.144488
H	-4.304559	4.349823	2.043464
C	3.976133	3.806593	1.140198
H	4.297943	4.356990	2.038707
C	5.826186	-1.568305	-1.252143
H	6.909556	-1.402510	-1.138950
C	3.144188	2.392132	-1.139789
H	2.810349	1.848137	-2.038530
C	-4.082648	4.399218	-0.122611
H	-4.484396	5.420020	-0.222490
C	5.353053	-2.601214	-2.083828
H	6.065970	-3.244808	-2.623521
C	-5.826304	-1.573523	-1.251028
H	-6.909778	-1.408762	-1.137325
C	4.076743	4.403841	-0.127498
H	4.477342	5.425002	-0.228314
C	-3.968777	-2.808137	-2.228957
H	-3.592703	-3.610286	-2.883832

C	-5.352560	-2.605437	-2.083597
H	-6.065103	-3.249294	-2.623470
C	3.660304	3.691320	-1.268710
H	3.733943	4.152603	-2.266431
H	-0.001391	1.510714	-0.027021
N	0.011319	-3.241651	2.565294
C	0.003694	-2.750775	1.455709
O	-0.001107	-2.524266	0.261626
C	0.016438	-4.510503	3.247597
H	-0.966781	-4.688018	3.731761
H	0.227631	-5.355031	2.557610
H	0.783479	-4.507845	4.048676

1TSEB_Ph

Frequencies, energies and thermodynamic properties:

Lowest Vibrational Mode (1/cm) = -136.0568

2nd Lowest Vibrational Mode (1/cm) = 12.9254

E(RB-P86) (a.u.) = -2428.78166647

Thermal correction to Enthalpy (a.u.) = 0.631417

Thermal correction to Gibbs Free Energy (a.u.) = 0.510027

Total Entropy (cal/Kmol) = 255.487

E(RPBE1PBE) (a.u.) = -2428.51764980

Optimised cartesian coordinates (Angstrom):

Ru	0.078524	-0.465696	0.146978
P	2.387327	-0.465512	0.480093
P	-2.213322	-0.842565	0.387358
N	0.068980	-0.877323	2.226863
O	0.113584	-0.088970	-2.861044
C	3.411368	-1.897014	-0.117566
C	-1.069699	-1.616455	2.760772
H	-1.079642	-1.565876	3.880225
H	-1.017025	-2.715022	2.515370
C	4.780671	-1.981919	0.229936
H	5.247755	-1.190727	0.839213
C	-3.455582	0.452372	-0.086937
C	3.426756	0.973870	-0.056108
C	-4.346415	-2.669804	-0.103967
H	-4.968227	-1.983228	0.493569
C	3.758995	2.046660	0.800874
H	3.463853	2.029445	1.860844
C	-3.384202	0.992257	-1.392682
H	-2.594459	0.653908	-2.082197
C	-2.391672	-1.045201	2.236774
H	-2.552555	-0.030549	2.657992
H	-3.264136	-1.675459	2.504279
C	2.510271	-0.547304	2.341337
H	3.480433	-0.970047	2.672931
H	2.434493	0.490582	2.726802
C	-2.982788	-2.374412	-0.338140
C	1.306825	-1.361445	2.828554

H	1.486709	-2.452871	2.611436
H	1.254687	-1.289480	3.945687
C	-4.307143	1.962839	-1.815910
H	-4.237537	2.373068	-2.835832
C	-4.464362	0.914182	0.788586
H	-4.545810	0.514287	1.810996
C	3.625382	-3.996472	-1.354034
H	3.168847	-4.781219	-1.978101
C	2.842762	-2.911941	-0.914693
H	1.777128	-2.844526	-1.184158
C	0.092599	-0.166117	-1.677312
C	3.823540	1.039523	-1.413975
H	3.574326	0.213216	-2.099526
C	5.559086	-3.067205	-0.204399
H	6.623154	-3.123515	0.075607
C	-4.925134	-3.832785	-0.638421
H	-5.987652	-4.052036	-0.446480
C	-5.384184	1.891658	0.363814
H	-6.164945	2.241780	1.057937
C	-2.216471	-3.258628	-1.125960
H	-1.155624	-3.026812	-1.308989
C	4.982444	-4.077572	-0.998213
H	5.594623	-4.927061	-1.340654
C	-5.309566	2.416241	-0.938039
H	-6.030108	3.181202	-1.268379
C	4.476879	3.154711	0.312887
H	4.732131	3.979707	0.996971
C	-4.151910	-4.711843	-1.420836
H	-4.607624	-5.621884	-1.842528
C	4.538809	2.146650	-1.898574
H	4.841939	2.178819	-2.957243
C	4.868372	3.209183	-1.035727
H	5.429914	4.077485	-1.415164
C	-2.798764	-4.421205	-1.664960
H	-2.188569	-5.101918	-2.279870
H	0.212290	-2.004852	-0.163436
N	-0.149203	1.839787	0.977068
C	-0.293777	1.806035	2.213966
O	-0.423667	1.872570	3.382755
C	-0.336693	3.011267	0.172373
C	-1.257073	4.014962	0.546098
C	0.410513	3.145380	-1.014400
C	-1.420458	5.148994	-0.266271
H	-1.852063	3.902183	1.466033
C	0.238422	4.284596	-1.818429
H	1.128119	2.359459	-1.287482
C	-0.675402	5.289122	-1.451443
H	-2.142214	5.926257	0.030795
H	0.829116	4.384498	-2.742664
H	-0.808599	6.177842	-2.087825

1TSEB
Frequencies, energies and thermodynamic properties:
Lowest Vibrational Mode (1/cm) = -171.9837
2nd Lowest Vibrational Mode (1/cm) = 11.9050
E(RB-P86) (a.u.) = -2237.17886820
Thermal correction to Enthalpy (a.u.) = 0.576839
Thermal correction to Gibbs Free Energy (a.u.) = 0.462258
Total Entropy (cal/Kmol) = 241.155
E(RPBE1PBE) (a.u.) = -2236.92598411
Optimised cartesian coordinates (Angstrom):

Ru	-0.001220	0.059231	0.064114
P	-2.309364	0.157097	0.384428
P	2.307585	0.178135	0.376614
N	0.002414	0.115009	2.196719
O	-0.011688	0.124016	-2.966703
C	-3.259425	1.700230	-0.034859
C	1.192686	0.660718	2.836635
H	1.182201	0.446130	3.936713
H	1.244326	1.784054	2.754277
C	-4.648814	1.775589	0.221671
H	-5.180579	0.910825	0.651371
C	3.415439	-1.145251	-0.308067
C	-3.410427	-1.155587	-0.329330
C	4.639660	1.803287	0.153770
H	5.182344	0.947265	0.587156
C	-4.204438	-2.020922	0.455815
H	-4.213540	-1.932712	1.552847
C	3.376027	-1.380674	-1.703444
H	2.701206	-0.786042	-2.339952
C	2.463497	0.050743	2.234743
H	2.505660	-1.030614	2.483330
H	3.390161	0.532200	2.608040
C	-2.456486	-0.007480	2.239421
H	-3.394221	0.442365	2.624474
H	-2.467821	-1.093405	2.470744
C	3.246879	1.717854	-0.079986
C	-1.199004	0.625873	2.845107
H	-1.278043	1.748447	2.774180
H	-1.180316	0.400832	3.942967
C	4.186063	-2.369449	-2.285974
H	4.143963	-2.538151	-3.373847
C	4.280567	-1.927267	0.489720
H	4.338089	-1.767443	1.577264
C	-3.325387	3.984135	-0.906604
H	-2.802879	4.845540	-1.352535
C	-2.605685	2.812948	-0.603845
H	-1.523983	2.749636	-0.802202
C	-0.006472	0.044644	-1.782237
C	-3.432458	-1.300120	-1.737692

H	-2.814183	-0.638931	-2.366202
C	-5.364815	2.946510	-0.077160
H	-6.446120	2.993584	0.128891
C	5.345620	2.972802	-0.173322
H	6.429818	3.027309	0.014948
C	5.086820	-2.922370	-0.095700
H	5.755331	-3.523979	0.540755
C	2.579508	2.819386	-0.655187
H	1.495305	2.748454	-0.836425
C	-4.704060	4.054113	-0.642655
H	-5.267312	4.970708	-0.879985
C	5.042899	-3.145595	-1.482470
H	5.674621	-3.924496	-1.938152
C	-5.001469	-3.008676	-0.154446
H	-5.614919	-3.675372	0.472541
C	4.671290	4.069131	-0.744563
H	5.226651	4.984488	-1.004210
C	-4.232723	-2.282006	-2.344258
H	-4.239232	-2.379210	-3.441610
C	-5.018707	-3.141770	-1.553194
H	-5.643266	-3.914859	-2.028219
C	3.289014	3.989185	-0.985939
H	2.755688	4.841570	-1.436380
H	-0.006456	1.647601	0.009514
N	0.002433	-2.227673	0.494154
C	0.032970	-2.509034	1.705735
O	0.065206	-2.903523	2.819125
C	-0.020857	-3.219921	-0.595528
H	-0.026294	-4.259559	-0.212595
H	-0.924321	-3.047017	-1.211979
H	0.870149	-3.065313	-1.234783

1TSEJ

Frequencies, energies and thermodynamic properties:

Lowest Vibrational Mode (1/cm) =	-650.2468
2nd Lowest Vibrational Mode (1/cm) =	18.2357
E(RB-P86) (a.u.) =	-2259.39444266
Thermal correction to Enthalpy (a.u.) =	0.605564
Thermal correction to Gibbs Free Energy (a.u.) =	0.490963
Total Entropy (cal/Kmol) =	241.197
E(RPBE1PBE) (a.u.) =	-2259.14346232

Optimised cartesian coordinates (Angstrom):

Ru	0.002247	-0.028578	0.035531
P	-2.322570	0.110163	0.350089
P	2.322019	0.202371	0.346877
N	0.008132	-0.077168	2.254842
O	0.086879	0.365277	-2.954661
C	-3.201985	1.728693	0.102944
C	1.199707	0.584361	2.850301
H	1.224385	0.400290	3.949425

H	1.105307	1.681719	2.700427
C	-4.578459	1.832562	0.414823
H	-5.132597	0.955409	0.786885
C	3.505479	-1.041485	-0.344001
C	-3.491431	-1.073750	-0.463990
C	4.419701	2.114369	0.431317
H	4.972881	1.364370	1.019666
C	-4.383293	-1.905042	0.249715
H	-4.416682	-1.883972	1.349552
C	4.413850	-0.710625	-1.374963
H	4.472012	0.321086	-1.754512
C	2.480130	0.049549	2.207781
H	2.585257	-1.032283	2.426205
H	3.376842	0.581788	2.582213
C	-2.464192	-0.236389	2.183141
H	-3.415020	0.143722	2.608607
H	-2.450712	-1.340550	2.305640
C	3.117545	1.830430	-0.046337
C	-1.253861	0.383370	2.883073
H	-1.294198	1.492357	2.818964
H	-1.262926	0.116525	3.964978
C	5.254961	-1.696974	-1.920875
H	5.960721	-1.426478	-2.722483
C	3.430089	-2.378777	0.114321
H	2.677121	-2.655012	0.876759
C	-3.208147	4.076097	-0.580324
H	-2.667705	4.950540	-0.976276
C	-2.524547	2.859214	-0.399393
H	-1.453390	2.778053	-0.641503
C	0.048313	0.182298	-1.793054
C	-3.478753	-1.132563	-1.877635
H	-2.781282	-0.499504	-2.449365
C	-5.256430	3.050039	0.240564
H	-6.327123	3.118824	0.490235
C	5.027330	3.349609	0.151936
H	6.039515	3.558465	0.533113
C	4.282264	-3.354843	-0.430081
H	4.221847	-4.392302	-0.063820
C	2.442974	2.804120	-0.812660
H	1.426233	2.593794	-1.176292
C	-4.572331	4.175134	-0.258143
H	-5.106506	5.128178	-0.398871
C	5.197533	-3.018639	-1.444823
H	5.860171	-3.788930	-1.870697
C	-5.247508	-2.776058	-0.440146
H	-5.936785	-3.420147	0.128601
C	4.346435	4.314616	-0.614252
H	4.824330	5.282482	-0.834044
C	-4.344876	-2.000177	-2.562059
H	-4.322246	-2.036608	-3.662600

C	-5.231109	-2.825365	-1.844502
H	-5.906491	-3.509951	-2.381445
C	3.056140	4.038357	-1.097704
H	2.518013	4.787891	-1.699376
H	-0.057167	1.582335	0.170375
H	0.344973	-1.956322	0.653108
C	-0.358330	-2.290850	-0.362262
H	-1.370931	-2.645869	0.004451
O	0.168538	-2.897486	-1.298574
H	0.134917	-1.137238	2.388595
O	0.732960	-2.613915	1.958718
C	0.301438	-3.911557	2.241612
H	0.436286	-4.614588	1.377092
H	0.877188	-4.360670	3.093437
H	-0.782592	-3.977150	2.531377

1TSFC_Ph

Frequencies, energies and thermodynamic properties:

Lowest Vibrational Mode (1/cm) = -216.7745

2nd Lowest Vibrational Mode (1/cm) = 2.9448

E(RB-P86) (a.u.) = -2429.98625550

Thermal correction to Enthalpy (a.u.) = 0.651357

Thermal correction to Gibbs Free Energy (a.u.) = 0.527655

Total Entropy (cal/Kmol) = 260.353

E(RPBE1PBE) (a.u.) = -2429.71666286

Optimised cartesian coordinates (Angstrom):

Ru	0.728128	0.122738	0.000000
P	0.668078	-0.172721	2.300974
P	0.668078	-0.172721	-2.300974
N	-0.444035	-1.764100	0.000000
O	2.217190	2.742292	0.000000
C	2.247486	-0.566657	3.191095
C	-0.270603	-2.573026	-1.235650
H	-1.014594	-3.403174	-1.262276
H	0.738619	-3.033597	-1.195311
C	2.600369	-1.870104	3.602271
H	1.910432	-2.714458	3.450935
C	-0.113117	1.047422	-3.463489
C	-0.113117	1.047422	3.463489
C	2.600369	-1.870104	-3.602271
H	1.910432	-2.714458	-3.450935
C	-0.080805	0.847776	4.863474
H	0.443386	-0.024577	5.286593
C	-0.773082	2.180127	-2.942232
H	-0.798366	2.329004	-1.850924
C	-0.421125	-1.690961	-2.476668
H	-1.459115	-1.302645	-2.549252
H	-0.219361	-2.257267	-3.408494
C	-0.421125	-1.690961	2.476668
H	-0.219361	-2.257267	3.408494

H	-1.459115	-1.302645	2.549252
C	2.247486	-0.566657	-3.191095
C	-0.270603	-2.573026	1.235650
H	0.738619	-3.033597	1.195311
H	-1.014594	-3.403174	1.262276
C	-1.396013	3.098988	-3.807577
H	-1.905136	3.982338	-3.390148
C	-0.080805	0.847776	-4.863474
H	0.443386	-0.024577	-5.286593
C	4.407811	0.242683	4.013583
H	5.111469	1.075469	4.171768
C	3.167932	0.487275	3.401734
H	2.913844	1.511531	3.083088
C	1.640024	1.708197	0.000000
C	-0.773082	2.180127	2.942232
H	-0.798366	2.329004	1.850924
C	3.845604	-2.114381	4.212497
H	4.104586	-3.137766	4.527972
C	3.845604	-2.114381	-4.212497
H	4.104586	-3.137766	-4.527972
C	-0.708978	1.763450	-5.724317
H	-0.681294	1.597869	-6.813273
C	3.167932	0.487275	-3.401734
H	2.913844	1.511531	-3.083088
C	4.751617	-1.060706	4.420455
H	5.725322	-1.253252	4.898127
C	-1.366620	2.891840	-5.197711
H	-1.853264	3.612089	-5.874669
C	-0.708978	1.763450	5.724317
H	-0.681294	1.597869	6.813273
C	4.751617	-1.060706	-4.420455
H	5.725322	-1.253252	-4.898127
C	-1.396013	3.098988	3.807577
H	-1.905136	3.982338	3.390148
C	-1.366620	2.891840	5.197711
H	-1.853264	3.612089	5.874669
C	4.407811	0.242683	-4.013583
H	5.111469	1.075469	-4.171768
H	2.097717	-0.839392	0.000000
H	-0.814855	0.864652	0.000000
H	-1.415248	-1.370856	0.000000
N	-3.056740	-0.297534	0.000000
C	-2.825268	0.928140	0.000000
O	-2.905805	2.114365	0.000000
C	-4.338626	-0.909334	0.000000
C	-4.406988	-2.320084	0.000000
C	-5.537105	-0.156520	0.000000
C	-5.653649	-2.967028	0.000000
H	-3.475244	-2.905726	0.000000
C	-6.778056	-0.812812	0.000000

H	-5.494293	0.943912	0.000000
C	-6.844701	-2.218829	0.000000
H	-5.691647	-4.067956	0.000000
H	-7.703730	-0.215433	0.000000
H	-7.820902	-2.728093	0.000000

1TSFC

Frequencies, energies and thermodynamic properties:

Lowest Vibrational Mode (1/cm) = -273.0870
2nd Lowest Vibrational Mode (1/cm) = 18.4273
E(RB-P86) (a.u.) = -2238.37708168
Thermal correction to Enthalpy (a.u.) = 0.597089
Thermal correction to Gibbs Free Energy (a.u.) = 0.481836
Total Entropy (cal/Kmol) = 242.572
E(RPBE1PBE) (a.u.) = -2238.12209017

Optimised cartesian coordinates (Angstrom):

Ru	0.000007	0.097494	-0.025300
P	-2.299205	0.190659	0.278698
P	2.299219	0.190633	0.278712
N	-0.000003	-0.209493	2.172526
O	0.000018	0.340431	-3.029035
C	-3.146637	1.823774	0.039337
C	1.234733	0.262287	2.851442
H	1.256853	-0.101571	3.905341
H	1.204455	1.371961	2.884870
C	-3.685441	2.587760	1.096616
H	-3.656178	2.215097	2.131962
C	3.492570	-0.975173	-0.539574
C	-3.492563	-0.975135	-0.539595
C	3.685490	2.587713	1.096635
H	3.656231	2.215044	2.131979
C	-4.890486	-0.776827	-0.458767
H	-5.294975	0.103459	0.066556
C	2.993120	-2.098714	-1.232264
H	1.903383	-2.249381	-1.293127
C	2.473156	-0.228439	2.098451
H	2.531698	-1.336818	2.136679
H	3.409694	0.162743	2.545091
C	-2.473160	-0.228408	2.098436
H	-3.409696	0.162787	2.545069
H	-2.531718	-1.336786	2.136664
C	3.146668	1.823740	0.039356
C	-1.234736	0.262301	2.851435
H	-1.204445	1.371975	2.884868
H	-1.256868	-0.101560	3.905333
C	3.878497	-3.014837	-1.829905
H	3.477449	-3.886801	-2.370882
C	4.890494	-0.776865	-0.458762
H	5.294989	0.103429	0.066544
C	-3.792554	3.593620	-1.525850

H	-3.830653	3.982092	-2.556054
C	-3.203095	2.344449	-1.275469
H	-2.776070	1.768639	-2.112789
C	0.000014	0.257771	-1.847424
C	-2.993119	-2.098664	-1.232309
H	-1.903382	-2.249330	-1.293183
C	-4.268833	3.844882	0.845161
H	-4.682318	4.429262	1.682696
C	4.268896	3.844829	0.845182
H	4.682394	4.429200	1.682717
C	5.771991	-1.695296	-1.054171
H	6.859585	-1.532775	-0.984806
C	3.203122	2.344421	-1.275448
H	2.776083	1.768622	-2.112767
C	-4.326267	4.349934	-0.464470
H	-4.783828	5.332725	-0.659801
C	5.267563	-2.815651	-1.741697
H	5.960078	-3.531229	-2.213137
C	-5.771988	-1.695250	-1.054181
H	-6.859581	-1.532730	-0.984803
C	4.326327	4.349888	-0.464447
H	4.783898	5.332673	-0.659776
C	-3.878502	-3.014779	-1.829955
H	-3.477459	-3.886734	-2.370950
C	-5.267566	-2.815594	-1.741730
H	-5.960086	-3.531165	-2.213174
C	3.792595	3.593585	-1.525827
H	3.830691	3.982062	-2.556028
H	0.000015	1.739615	0.273513
H	0.000007	-1.620075	-0.070446
H	-0.000014	-1.264097	2.192488
N	-0.000062	-3.099236	1.892797
C	-0.000035	-3.400822	0.685161
O	0.000032	-4.006802	-0.344909
C	-0.000227	-4.072602	2.990902
H	0.894297	-3.914785	3.628096
H	-0.896216	-3.916419	3.626455
H	0.001045	-5.123583	2.633521

1TSGH

Frequencies, energies and thermodynamic properties:

Lowest Vibrational Mode (1/cm) = -166.6686
2nd Lowest Vibrational Mode (1/cm) = 15.0612
E(RB-P86) (a.u.) = -2238.37764496
Thermal correction to Enthalpy (a.u.) = 0.598358
Thermal correction to Gibbs Free Energy (a.u.) = 0.484037
Total Entropy (cal/Kmol) = 240.608
E(RPBE1PBE) (a.u.) = -2238.12065228

Optimised cartesian coordinates (Angstrom):

Ru	0.000510	0.005032	0.025206

P	-2.301409	0.194645	0.332369
P	2.302548	0.192334	0.332690
N	0.000266	-0.070410	2.212671
O	0.000921	0.031520	-2.993415
C	-3.060773	1.852770	0.004088
C	1.225129	0.415573	2.883708
H	1.235465	0.079994	3.949623
H	1.230569	1.529555	2.901344
C	-3.672548	2.641126	1.002265
H	-3.741178	2.280075	2.039760
C	3.524700	-0.973390	-0.433202
C	-3.524671	-0.970056	-0.433309
C	3.677064	2.636693	1.003355
H	3.746473	2.274565	2.040423
C	-4.910747	-0.694268	-0.457714
H	-5.294142	0.249584	-0.037614
C	3.045009	-2.182509	-0.985591
H	1.963654	-2.395587	-0.952112
C	2.469325	-0.122620	2.168091
H	2.511300	-1.228795	2.261384
H	3.410699	0.278286	2.595786
C	-2.468803	-0.119466	2.167853
H	-3.409732	0.282756	2.595291
H	-2.512124	-1.225560	2.261536
C	3.063321	1.849946	0.005114
C	-1.224015	0.417380	2.883464
H	-1.228006	1.531372	2.900801
H	-1.234895	0.082093	3.949466
C	3.944328	-3.103322	-1.553909
H	3.563349	-4.043059	-1.984953
C	4.910584	-0.697061	-0.461487
H	5.294549	0.247836	-0.044288
C	-3.530563	3.613334	-1.631942
H	-3.470911	3.989081	-2.665809
C	-2.990617	2.356440	-1.316769
H	-2.501160	1.761386	-2.104696
C	0.000731	0.011990	-1.808884
C	-3.045788	-2.177726	-0.989462
H	-1.964284	-2.390357	-0.958994
C	-4.203813	3.906922	0.686485
H	-4.673945	4.511894	1.478191
C	4.209294	3.902240	0.688205
H	4.680915	4.505973	1.479972
C	5.804372	-1.620681	-1.030868
H	6.882999	-1.396519	-1.050988
C	2.992236	2.354958	-1.315185
H	2.501203	1.761211	-2.103111
C	-4.137243	4.394445	-0.629319
H	-4.554372	5.383931	-0.874987
C	5.322795	-2.824652	-1.578549

H	6.024401	-3.544669	-2.029379
C	-5.805478	-1.617062	-1.026923
H	-6.884254	-1.393364	-1.043979
C	4.141775	4.391094	-0.627058
H	4.559642	5.380391	-0.872235
C	-3.946029	-3.097732	-1.557649
H	-3.565657	-4.036361	-1.991632
C	-5.324664	-2.819643	-1.578354
H	-6.027029	-3.539017	-2.029027
C	3.533179	3.611574	-1.629757
H	3.472762	3.988381	-2.663194
H	0.001202	1.603046	0.042565
O	-0.000411	-2.284800	0.316864
C	-0.002400	-2.662700	1.545929
N	-0.007787	-4.002326	1.782440
H	-0.000773	-1.258373	2.259040
H	-0.005835	-4.279620	2.766018
C	-0.010256	-5.076761	0.788966
H	-0.895688	-5.737363	0.908858
H	-0.041287	-4.618022	-0.216817
H	0.902541	-5.706443	0.865904

1TSHI_Ph

Frequencies, energies and thermodynamic properties:

Lowest Vibrational Mode (1/cm) = -215.6893

2nd Lowest Vibrational Mode (1/cm) = 12.5737

E(RB-P86) (a.u.) = -2429.96344310

Thermal correction to Enthalpy (a.u.) = 0.655213

Thermal correction to Gibbs Free Energy (a.u.) = 0.532758

Total Entropy (cal/Kmol) = 257.728

E(RPBE1PBE) (a.u.) = -2429.69647266

Optimised cartesian coordinates (Angstrom):

Ru	0.487129	0.250356	0.012904
P	-1.154937	1.881029	0.305925
P	2.431806	-1.017042	0.378174
N	0.445477	0.235810	2.244362
O	0.639517	0.358402	-2.991846
C	-0.618158	3.624344	-0.024388
C	1.690339	-0.176296	2.925821
H	1.482672	-0.436833	3.990207
H	2.389428	0.689089	2.936061
C	-0.502886	4.607346	0.981815
H	-0.755010	4.375043	2.027687
C	2.784822	-2.672458	-0.372983
C	-2.833022	1.828769	-0.475268
C	4.986634	0.087289	1.133114
H	4.835320	-0.333146	2.138971
C	-3.721482	2.924758	-0.377219
H	-3.414383	3.842634	0.149884
C	1.695442	-3.438456	-0.846923

H	0.674539	-3.020851	-0.781531
C	2.326167	-1.372892	2.211664
H	1.671607	-2.266565	2.289455
H	3.305291	-1.645244	2.655339
C	-1.497600	1.774994	2.142776
H	-1.948253	2.701766	2.552147
H	-2.256092	0.970165	2.240148
C	4.018600	-0.093786	0.121524
C	-0.212055	1.394411	2.885795
H	0.504597	2.245150	2.889593
H	-0.446715	1.166525	3.952208
C	1.923710	-4.714831	-1.393628
H	1.071914	-5.308106	-1.763335
C	4.097182	-3.190607	-0.461047
H	4.953930	-2.596162	-0.105880
C	0.149695	5.263607	-1.672951
H	0.403569	5.514365	-2.715131
C	-0.282296	3.966043	-1.356058
H	-0.355562	3.208649	-2.153214
C	0.566447	0.303725	-1.814315
C	-3.232847	0.667155	-1.171417
H	-2.543216	-0.189834	-1.241551
C	-0.060867	5.906102	0.663041
H	0.026089	6.661175	1.460506
C	6.166009	0.811190	0.871404
H	6.908745	0.948027	1.673428
C	4.315684	-4.465936	-1.010356
H	5.341338	-4.862945	-1.077225
C	4.255751	0.462288	-1.158102
H	3.504940	0.343520	-1.955455
C	0.263786	6.238081	-0.662735
H	0.607936	7.254659	-0.910536
C	3.229794	-5.230123	-1.477193
H	3.404383	-6.227626	-1.911407
C	-4.996963	2.853887	-0.962088
H	-5.683796	3.711628	-0.881582
C	6.395843	1.352718	-0.404392
H	7.319360	1.917560	-0.607867
C	-4.510659	0.603761	-1.757823
H	-4.815421	-0.306112	-2.298445
C	-5.392827	1.693447	-1.654398
H	-6.391401	1.641677	-2.116971
C	5.437861	1.172987	-1.420484
H	5.608353	1.596055	-2.423181
H	1.472361	1.474324	0.073165
O	-1.060166	-1.908503	-0.407648
C	-1.287992	-1.622762	0.811643
N	-2.541701	-2.011930	1.337446
H	-0.237754	-0.589466	2.118725
H	-2.652412	-1.759477	2.323876

C	-3.634674	-2.730456	0.807533
C	-4.753294	-2.967202	1.650576
C	-3.667271	-3.240369	-0.518001
C	-5.865840	-3.684775	1.188777
H	-4.741372	-2.577690	2.682792
C	-4.789571	-3.958957	-0.965504
H	-2.803676	-3.062593	-1.169460
C	-5.894668	-4.188394	-0.125921
H	-6.719640	-3.852270	1.865533
H	-4.794430	-4.348184	-1.997062
H	-6.767970	-4.752366	-0.489591

1TSHI

Frequencies, energies and thermodynamic properties:

Lowest Vibrational Mode (1/cm) = -242.1206
2nd Lowest Vibrational Mode (1/cm) = 15.8735
E(RB-P86) (a.u.) = -2238.35478472
Thermal correction to Enthalpy (a.u.) = 0.600413
Thermal correction to Gibbs Free Energy (a.u.) = 0.487334
Total Entropy (cal/Kmol) = 237.996
E(RPBE1PBE) (a.u.) = -2238.10170128

Optimised cartesian coordinates (Angstrom):

Ru	0.053555	-0.092367	0.045199
P	-2.226824	0.275624	0.354291
P	2.376220	0.017329	0.377914
N	0.058298	-0.021468	2.283364
O	0.067783	0.004935	-2.962419
C	-2.829568	1.988921	-0.021378
C	1.311017	0.427315	2.921678
H	1.313540	0.162237	4.005363
H	1.361370	1.537275	2.861225
C	-3.390620	2.849083	0.947055
H	-3.508144	2.521243	1.991137
C	3.610175	-1.186064	-0.299925
C	-3.556166	-0.782028	-0.385314
C	3.794539	2.463629	0.950643
H	3.885402	2.129799	1.995494
C	-4.920352	-0.419958	-0.298848
H	-5.215152	0.518121	0.198888
C	3.148932	-2.460640	-0.701090
H	2.068771	-2.688134	-0.627437
C	2.522752	-0.205643	2.230290
H	2.524545	-1.305781	2.381337
H	3.479324	0.182067	2.635913
C	-2.419147	0.044756	2.201037
H	-3.332152	0.524973	2.607349
H	-2.534397	-1.051492	2.333094
C	3.143939	1.659429	-0.009874
C	-1.154405	0.541373	2.911398
H	-1.091316	1.651293	2.864425

H	-1.197130	0.267320	3.992078
C	4.064862	-3.413294	-1.185349
H	3.702356	-4.405499	-1.499344
C	4.985716	-0.874198	-0.396478
H	5.351810	0.120847	-0.096969
C	-3.124988	3.741947	-1.705443
H	-3.017523	4.085767	-2.746491
C	-2.696973	2.452479	-1.351967
H	-2.248569	1.799996	-2.118308
C	0.060269	-0.054755	-1.781838
C	-3.191482	-1.976131	-1.045659
H	-2.127011	-2.260391	-1.103868
C	-3.810111	4.146115	0.592501
H	-4.242310	4.806367	1.361258
C	4.338831	3.710835	0.587315
H	4.839024	4.328402	1.350290
C	5.892471	-1.832145	-0.882322
H	6.963244	-1.582753	-0.955877
C	3.049974	2.128060	-1.342068
H	2.532243	1.520887	-2.101791
C	-3.681067	4.594620	-0.732474
H	-4.010621	5.608845	-1.008315
C	5.433703	-3.103066	-1.277259
H	6.146061	-3.850615	-1.661839
C	-5.907742	-1.247599	-0.859860
H	-6.968557	-0.958348	-0.789333
C	4.247631	4.163606	-0.739454
H	4.675156	5.138571	-1.022363
C	-4.185514	-2.798475	-1.608892
H	-3.894537	-3.726548	-2.126427
C	-5.541476	-2.437522	-1.517291
H	-6.316395	-3.081439	-1.963180
C	3.603534	3.366211	-1.704910
H	3.524910	3.714181	-2.747150
H	0.142444	1.481442	0.058914
O	0.023056	-2.723798	-0.283437
C	-0.281763	-2.575338	0.954829
N	-1.098162	-3.547684	1.509050
H	0.005152	-1.097211	2.197468
H	-1.308311	-3.443504	2.504384
C	-1.588210	-4.752025	0.839578
H	-1.297897	-5.672231	1.391061
H	-2.694392	-4.750410	0.723275
H	-1.128563	-4.777462	-0.167956

1TSID_Ph

Frequencies, energies and thermodynamic properties:

Lowest Vibrational Mode (1/cm) = -108.7553

2nd Lowest Vibrational Mode (1/cm) = 9.5626

E(RB-P86) (a.u.) = -2430.00031272

Thermal correction to Enthalpy (a.u.) = 0.653877
Thermal correction to Gibbs Free Energy (a.u.) = 0.531901
Total Entropy (cal/Kmol) = 256.719
E(RPBE1PBE) (a.u.) = -2429.73115125

Optimised cartesian coordinates (Angstrom):

Ru	-0.106885	-0.434701	0.086499
P	-2.420160	-0.341227	-0.315944
P	2.184038	-0.857266	-0.219378
N	-0.005470	0.125437	-2.041741
O	-0.256944	-1.449412	2.927941
C	-3.433482	-1.897836	-0.263780
C	1.220115	-0.303000	-2.760851
H	1.321140	0.270831	-3.711256
H	1.105380	-1.373286	-3.040016
C	-2.824337	-3.152691	-0.054712
H	-1.733534	-3.199174	0.092196
C	3.520101	-0.141478	0.842907
C	-3.479483	0.852589	0.620352
C	3.142793	-3.239211	-1.520909
H	3.224661	-2.658669	-2.452301
C	-4.347878	1.774188	-0.007151
H	-4.430640	1.814969	-1.103993
C	3.194752	0.675194	1.946016
H	2.141034	0.883236	2.181264
C	2.471382	-0.109005	-1.903018
H	2.650207	0.968877	-1.708197
H	3.378351	-0.505400	-2.402909
C	-2.488062	0.225755	-2.100013
H	-3.398798	-0.148442	-2.609023
H	-2.529470	1.333958	-2.093005
C	2.695718	-2.633669	-0.327191
C	-1.219538	-0.240670	-2.815252
H	-1.231232	-1.343602	-2.953295
H	-1.176359	0.217671	-3.830808
C	4.214977	1.228854	2.741946
H	3.949677	1.864269	3.601536
C	4.879231	-0.404969	0.550402
H	5.150178	-1.057465	-0.295417
C	-5.605826	-3.007447	-0.445042
H	-6.695340	-2.944746	-0.595139
C	-4.834489	-1.833672	-0.453239
H	-5.332205	-0.861597	-0.600026
C	-0.198272	-1.033814	1.828553
C	-3.407003	0.830520	2.032944
H	-2.727724	0.126346	2.538724
C	-3.600855	-4.326311	-0.042419
H	-3.112850	-5.299708	0.124693
C	3.493042	-4.603215	-1.538767
H	3.838677	-5.062412	-2.478538
C	5.894089	0.155163	1.343073

H	6.949652	-0.052002	1.105557
C	2.608533	-3.416082	0.848516
H	2.257578	-2.961262	1.789079
C	-4.990590	-4.256942	-0.239020
H	-5.597902	-5.175923	-0.227621
C	5.563392	0.972809	2.440850
H	6.360807	1.407600	3.064152
C	-5.126464	2.656290	0.765568
H	-5.797088	3.371064	0.262869
C	3.405885	-5.372538	-0.366664
H	3.681518	-6.438798	-0.382594
C	-4.189933	1.707968	2.800407
H	-4.120598	1.680205	3.899259
C	-5.050438	2.624921	2.168384
H	-5.659137	3.317678	2.770587
C	2.964433	-4.773769	0.829061
H	2.894100	-5.368982	1.753256
H	-0.222611	-1.994103	-0.454734
O	0.127444	2.331675	1.482207
C	0.025745	1.529827	0.622710
N	-0.235643	2.691017	-1.203535
H	0.000882	1.214802	-1.885794
H	-1.227813	2.858996	-0.971851
C	0.445402	3.886067	-1.127662
C	1.833940	3.958583	-1.469560
C	-0.164469	5.107801	-0.695840
C	2.547490	5.162137	-1.397859
H	2.341051	3.040295	-1.807743
C	0.555032	6.310475	-0.634851
H	-1.231467	5.091674	-0.413371
C	1.919651	6.356006	-0.983942
H	3.615423	5.171558	-1.674829
H	0.041738	7.228974	-0.302525
H	2.483790	7.300525	-0.932215

1TSID

Frequencies, energies and thermodynamic properties:

Lowest Vibrational Mode (1/cm) = -102.9698

2nd Lowest Vibrational Mode (1/cm) = 14.3646

E(RB-P86) (a.u.) = -2238.38391667

Thermal correction to Enthalpy (a.u.) = 0.597915

Thermal correction to Gibbs Free Energy (a.u.) = 0.487274

Total Entropy (cal/Kmol) = 232.864

E(RPBE1PBE) (a.u.) = -2238.12830421

Optimised cartesian coordinates (Angstrom):

Ru	-0.006353	-0.094028	0.058838
P	-2.308664	0.164619	0.367619
P	2.299133	0.112997	0.374607
N	-0.012711	-0.159057	2.259999
O	0.034607	0.172743	-2.954970

C	-3.138055	1.781121	-0.045052
C	1.195438	0.379993	2.892784
H	1.225009	0.108110	3.979117
H	1.189853	1.501776	2.863190
C	-2.382210	2.876780	-0.510653
H	-1.293151	2.754632	-0.629699
C	3.555609	-1.039108	-0.358372
C	-3.500808	-1.058962	-0.354383
C	3.409053	2.699877	0.976273
H	3.266513	2.469522	2.043059
C	-4.509902	-1.697040	0.402420
H	-4.629259	-1.480916	1.475363
C	3.148858	-2.073493	-1.227378
H	2.082525	-2.190360	-1.472091
C	2.470745	-0.132578	2.217647
H	2.577234	-1.229321	2.358066
H	3.387338	0.339281	2.627514
C	-2.490654	-0.006399	2.223179
H	-3.372235	0.550827	2.600138
H	-2.653678	-1.082981	2.440569
C	3.061676	1.757894	-0.015736
C	-1.187970	0.477667	2.866703
H	-1.127365	1.594862	2.779058
H	-1.228677	0.262546	3.965275
C	4.100954	-2.956754	-1.771738
H	3.773022	-3.758727	-2.452160
C	4.928379	-0.896378	-0.046056
H	5.264478	-0.081755	0.616106
C	-5.159731	3.154345	-0.169860
H	-6.248636	3.256833	-0.036835
C	-4.535911	1.928911	0.117752
H	-5.148422	1.080460	0.463266
C	0.015544	0.043244	-1.780174
C	-3.383699	-1.364056	-1.730614
H	-2.593284	-0.887533	-2.331550
C	-3.010223	4.101860	-0.803682
H	-2.407719	4.948711	-1.169513
C	3.937211	3.954100	0.614884
H	4.203414	4.678279	1.401393
C	5.874182	-1.784049	-0.584742
H	6.940302	-1.666763	-0.332593
C	3.252912	2.095609	-1.376179
H	2.984114	1.374736	-2.165542
C	-4.397909	4.244330	-0.632481
H	-4.889211	5.203179	-0.862779
C	5.461726	-2.815887	-1.450195
H	6.205387	-3.507857	-1.876708
C	-5.382288	-2.619310	-0.206158
H	-6.162828	-3.109493	0.397468
C	4.125210	4.280860	-0.738809

H	4.538622	5.262491	-1.019676
C	-4.259903	-2.279306	-2.336803
H	-4.153746	-2.506549	-3.409429
C	-5.260930	-2.911338	-1.575586
H	-5.943841	-3.633912	-2.049843
C	3.781731	3.346207	-1.734317
H	3.925630	3.592818	-2.798373
H	0.075230	1.562731	0.251447
O	-0.137460	-3.092096	-0.701365
C	-0.120630	-2.161944	0.070214
N	-0.242388	-2.723778	1.707057
H	-0.106481	-1.703180	2.206863
H	-1.219135	-3.039819	1.811524
C	0.690887	-3.807497	2.028775
H	1.728359	-3.418463	2.007780
H	0.493180	-4.230910	3.034949
H	0.612723	-4.617778	1.273675

1TSJaD

Frequencies, energies and thermodynamic properties:

Lowest Vibrational Mode (1/cm) = -851.8693
2nd Lowest Vibrational Mode (1/cm) = 17.2349
E(RB-P86) (a.u.) = -2143.75652127
Thermal correction to Enthalpy (a.u.) = 0.545643
Thermal correction to Gibbs Free Energy (a.u.) = 0.439226
Total Entropy (cal/Kmol) = 223.972
E(RPBE1PBE) (a.u.) = -2143.49991090

Optimised cartesian coordinates (Angstrom):

Ru	0.000000	-0.352833	0.204712
P	-2.308193	-0.003910	0.458248
P	2.308193	-0.003913	0.458248
N	0.000001	0.040214	2.330932
O	0.000001	-0.772038	-2.797928
C	-2.981612	1.604067	-0.165267
C	1.213995	0.652187	2.908899
H	1.216365	0.505653	4.016058
H	1.210916	1.754668	2.738678
C	-3.487048	2.610009	0.685638
H	-3.511612	2.465620	1.776343
C	3.593268	-1.207012	-0.115696
C	-3.593268	-1.207009	-0.115693
C	3.487054	2.610003	0.685643
H	3.511620	2.465611	1.776347
C	-4.960076	-0.976091	0.167807
H	-5.269631	-0.067531	0.709447
C	3.221779	-2.364074	-0.831834
H	2.161756	-2.547980	-1.060596
C	2.473449	0.017579	2.312421
H	2.540151	-1.051798	2.603831
H	3.401170	0.515893	2.659424

C	-2.473448	0.017586	2.312422
H	-3.401168	0.515904	2.659424
H	-2.540154	-1.051791	2.603833
C	2.981614	1.604064	-0.165264
C	-1.213992	0.652191	2.908899
H	-1.210910	1.754672	2.738676
H	-1.216362	0.505657	4.016058
C	4.201597	-3.282902	-1.252124
H	3.900316	-4.182059	-1.812406
C	4.960076	-0.976089	0.167795
H	5.269633	-0.067524	0.709426
C	-3.452062	3.034276	-2.094286
H	-3.435827	3.194575	-3.183968
C	-2.965612	1.830140	-1.561801
H	-2.565051	1.059500	-2.240207
C	0.000000	-0.625844	-1.630763
C	-3.221781	-2.364065	-0.831841
H	-2.161759	-2.547968	-1.060609
C	-3.967203	3.820385	0.148994
H	-4.355939	4.597774	0.825807
C	3.967211	3.820379	0.149001
H	4.355950	4.597765	0.825816
C	5.934318	-1.898613	-0.246963
H	6.995638	-1.712399	-0.017867
C	2.965612	1.830141	-1.561798
H	2.565048	1.059504	-2.240205
C	-3.952965	4.034828	-1.239158
H	-4.330053	4.981684	-1.656868
C	5.556519	-3.053805	-0.958167
H	6.322531	-3.774124	-1.286480
C	-5.934318	-1.898614	-0.246951
H	-6.995637	-1.712405	-0.017848
C	3.952971	4.034826	-1.239151
H	4.330060	4.981683	-1.656859
C	-4.201600	-3.282893	-1.252131
H	-3.900319	-4.182046	-1.812420
C	-5.556520	-3.053801	-0.958165
H	-6.322533	-3.774120	-1.286479
C	3.452064	3.034278	-2.094280
H	3.435827	3.194580	-3.183962
H	0.000000	1.276956	-0.042927
H	0.000000	-1.230633	2.642706
C	-0.000006	-2.332972	0.628596
H	-0.000003	-2.246456	2.608626
O	-0.000013	-3.480122	0.359078

4Aa

Frequencies, energies and thermodynamic properties:

Lowest Vibrational Mode (1/cm) = 35.2381

2nd Lowest Vibrational Mode (1/cm) = 44.0088

E(RB-P86) (a.u.) = -2954.92723024
Thermal correction to Enthalpy (a.u.) = 0.615543
Thermal correction to Gibbs Free Energy (a.u.) = 0.512423
Total Entropy (cal/Kmol) = 217.033
E(RPBE1PBE) (a.u.) = -2954.55692121

Optimised cartesian coordinates (Angstrom):

P	-2.289209	-0.140481	0.181114
C	-2.466907	-0.486065	2.022191
C	-1.237939	-1.256701	2.504588
H	-3.402323	-1.025108	2.274781
H	-2.519491	0.499782	2.533369
H	-1.240145	-1.345743	3.615889
H	-1.227980	-2.287513	2.093755
N	0.000110	-0.573914	2.042755
C	1.238231	-1.256646	2.504475
C	2.467120	-0.485951	2.021976
H	1.228284	-2.287456	2.093638
H	1.240540	-1.345694	3.615776
H	2.519703	0.499901	2.533146
H	3.402582	-1.024952	2.274487
P	2.289255	-0.140381	0.180914
Mn	0.000005	-0.145938	-0.084612
C	0.000017	-1.861148	-0.452397
O	-0.000195	-3.014967	-0.740085
C	-0.000084	0.164860	-1.819235
O	-0.000300	0.284213	-2.997371
C	-3.260069	1.473389	-0.013454
C	-4.637797	1.478886	0.669876
C	-3.361621	1.917487	-1.482899
H	-2.594701	2.192971	0.511765
H	-4.585392	1.242119	1.751538
H	-5.095600	2.487433	0.576980
H	-5.341519	0.761468	0.196119
H	-2.382590	1.882457	-2.000616
H	-4.077948	1.288815	-2.052561
H	-3.732276	2.963293	-1.534779
C	-3.391126	-1.428230	-0.676914
C	-3.401919	-2.805410	0.010476
C	-3.024762	-1.568329	-2.166770
H	-4.417273	-1.002025	-0.604343
H	-3.755409	-2.763197	1.060149
H	-4.094100	-3.481793	-0.535953
H	-2.399854	-3.279435	-0.007315
H	-2.983110	-0.596239	-2.694898
H	-2.038912	-2.059607	-2.290559
H	-3.781688	-2.199299	-2.680513
C	3.260026	1.473539	-0.013706
C	3.361753	1.917493	-1.483182
C	4.637657	1.479209	0.669817
H	2.594537	2.193127	0.511353

H	2.382814	1.882259	-2.001059
H	3.732261	2.963348	-1.535114
H	4.078268	1.288868	-2.052658
H	4.585118	1.242520	1.751490
H	5.341510	0.761815	0.196216
H	5.095387	2.487788	0.576907
C	3.391194	-1.428070	-0.677177
C	3.024405	-1.568590	-2.166889
C	3.402532	-2.805087	0.010531
H	4.417265	-1.001613	-0.605014
H	2.982351	-0.596631	-2.695223
H	3.781330	-2.199490	-2.680722
H	2.038639	-2.060141	-2.290266
H	3.756318	-2.762537	1.060091
H	2.400585	-3.279371	-0.006855
H	4.094727	-3.481423	-0.535940
H	-0.000841	2.491246	-1.568338
C	-0.000467	2.907371	-0.524384
H	0.000108	0.368896	2.463547
N	-0.000018	1.930054	0.418831
O	-0.000543	4.145567	-0.350792
C	0.000426	2.442595	1.785763
H	-0.897969	2.125351	2.369044
H	0.899315	2.125537	2.368386
H	0.000309	3.552535	1.767926

4A

Frequencies, energies and thermodynamic properties:

Lowest Vibrational Mode (1/cm) = 39.0480
2nd Lowest Vibrational Mode (1/cm) = 57.1078
E(RB-P86) (a.u.) = -2954.94331151
Thermal correction to Enthalpy (a.u.) = 0.615476
Thermal correction to Gibbs Free Energy (a.u.) = 0.513962
Total Entropy (cal/Kmol) = 213.653
E(RPBE1PBE) (a.u.) = -2954.56883176

Optimised cartesian coordinates (Angstrom):

P	2.291772	0.046117	0.130155
C	2.444554	-0.115746	1.991803
C	1.233222	0.557183	2.640571
H	3.401785	0.279455	2.388044
H	2.411949	-1.204859	2.209709
H	1.228157	0.359415	3.739345
H	1.268718	1.660881	2.511375
N	0.000003	0.033906	2.018328
C	-1.232979	0.557797	2.640492
C	-2.444587	-0.114850	1.991948
H	-1.268096	1.661479	2.511009
H	-1.227956	0.360317	3.739320
H	-2.412476	-1.203899	2.210236
H	-3.401666	0.280870	2.388040

P	-2.291742	0.046323	0.130223
Mn	0.000004	0.108456	-0.142872
C	-0.000005	1.859306	-0.091378
O	-0.000040	3.047745	-0.083008
C	-0.000192	0.209303	-1.902460
O	-0.000438	0.345301	-3.078694
C	3.301808	-1.433767	-0.490799
C	4.628589	-1.653990	0.256240
C	3.527060	-1.397398	-2.013187
H	2.619265	-2.280132	-0.257710
H	4.490242	-1.795703	1.346341
H	5.128892	-2.567192	-0.132902
H	5.335925	-0.810176	0.106190
H	2.598542	-1.175986	-2.577230
H	4.286477	-0.637989	-2.295392
H	3.907520	-2.381184	-2.362002
C	3.383879	1.527307	-0.345300
C	3.386298	2.665558	0.690819
C	3.024763	2.065862	-1.743286
H	4.412863	1.102738	-0.384972
H	3.728986	2.336932	1.692306
H	4.082597	3.464410	0.355552
H	2.383698	3.127225	0.793740
H	3.007671	1.276631	-2.519159
H	2.030029	2.554197	-1.739462
H	3.772170	2.827317	-2.054470
C	-3.301637	-1.433910	-0.490111
C	-3.526578	-1.398349	-2.012566
C	-4.628575	-1.653751	0.256779
H	-2.619116	-2.280118	-0.256418
H	-2.597965	-1.177185	-2.576597
H	-3.906960	-2.382313	-2.360965
H	-4.285957	-0.639088	-2.295290
H	-4.490482	-1.794823	1.346995
H	-5.335905	-0.810051	0.106063
H	-5.128756	-2.567197	-0.131948
C	-3.383857	1.527230	-0.345943
C	-3.024523	2.065262	-1.744077
C	-3.386506	2.665882	0.689748
H	-4.412812	1.102598	-0.385616
H	-3.007117	1.275706	-2.519615
H	-3.771969	2.826478	-2.055742
H	-2.029856	2.553740	-1.740223
H	-3.729356	2.337607	1.691295
H	-2.383942	3.127620	0.792683
H	-4.082791	3.464568	0.354060
H	-0.000147	-3.997857	0.580244
C	-0.000249	-2.904969	0.870613
H	-0.000181	-1.027274	2.156681
N	0.000003	-2.057141	-0.162157

O	-0.000366	-2.630225	2.107935
C	0.000134	-2.701926	-1.473493
H	-0.889186	-2.411698	-2.071680
H	0.889879	-2.412202	-2.071280
H	-0.000239	-3.813385	-1.385945

4Ba

Frequencies, energies and thermodynamic properties:

Lowest Vibrational Mode (1/cm) = 35.5348
2nd Lowest Vibrational Mode (1/cm) = 40.8576
E(RB-P86) (a.u.) = -2953.73396399
Thermal correction to Enthalpy (a.u.) = 0.592661
Thermal correction to Gibbs Free Energy (a.u.) = 0.489831
Total Entropy (cal/Kmol) = 216.426
E(RPBE1PBE) (a.u.) = -2953.35573331

Optimised cartesian coordinates (Angstrom):

P	2.285210	-0.095834	-0.017065
C	2.483033	1.140302	-1.422866
C	1.231071	1.121122	-2.301929
H	3.396020	0.962450	-2.025511
H	2.589696	2.143556	-0.960199
H	1.210398	2.019153	-2.960030
H	1.202521	0.217427	-2.946249
N	-0.000023	1.098185	-1.460908
C	-1.231146	1.121332	-2.301874
C	-2.483058	1.140518	-1.422731
H	-1.202714	0.217722	-2.946315
H	-1.210409	2.019461	-2.959837
H	-2.589626	2.143745	-0.959985
H	-3.396098	0.962756	-2.025324
P	-2.285216	-0.095732	-0.017032
Mn	-0.000022	-0.337795	0.101688
C	-0.000047	-1.815412	-0.830902
O	-0.000052	-2.814259	-1.474026
C	-0.000013	-1.255636	1.607910
O	-0.000286	-1.888870	2.607697
C	3.131066	0.823802	1.406604
C	4.534056	1.357182	1.070293
C	3.137374	0.039529	2.729998
H	2.432127	1.682490	1.522769
H	4.553778	1.978790	0.152680
H	4.902638	1.991972	1.904860
H	5.269223	0.534120	0.942626
H	2.143237	-0.377458	2.982802
H	3.867869	-0.796500	2.709085
H	3.442072	0.713057	3.559497
C	3.434505	-1.539676	-0.432882
C	3.395193	-1.934672	-1.920149
C	3.140136	-2.757085	0.464255
H	4.456196	-1.162684	-0.200294

H	3.689428	-1.105524	-2.594445
H	4.109020	-2.767408	-2.098744
H	2.389761	-2.292980	-2.220459
H	3.141520	-2.507878	1.543120
H	2.153223	-3.200176	0.221787
H	3.910606	-3.540450	0.298661
C	-3.130993	0.823817	1.406750
C	-3.136919	0.039605	2.730181
C	-4.534145	1.356929	1.070687
H	-2.432179	1.682631	1.522748
H	-2.142672	-0.377233	2.982801
H	-3.441538	0.713129	3.559712
H	-3.867304	-0.796525	2.709455
H	-4.554158	1.978524	0.153074
H	-5.269176	0.533724	0.943165
H	-4.902696	1.991657	1.905316
C	-3.434579	-1.539486	-0.432968
C	-3.140147	-2.757060	0.463920
C	-3.395421	-1.934218	-1.920309
H	-4.456238	-1.162503	-0.200218
H	-3.141474	-2.508059	1.542833
H	-3.910618	-3.540402	0.298222
H	-2.153244	-3.200098	0.221311
H	-3.689681	-1.104937	-2.594431
H	-2.390034	-2.292517	-2.220778
H	-4.109303	-2.766890	-2.098987
C	0.000075	2.201719	-0.438836
N	0.000071	3.419753	-0.853523
O	0.000113	1.679244	0.752088
C	0.000175	4.466935	0.157464
H	-0.000455	5.464703	-0.328855
H	-0.891563	4.417531	0.826804
H	0.892670	4.418209	0.825838

4B

Frequencies, energies and thermodynamic properties:

Lowest Vibrational Mode (1/cm) =	34.3704
2nd Lowest Vibrational Mode (1/cm) =	40.2436
E(RB-P86) (a.u.) =	-2953.74731998
Thermal correction to Enthalpy (a.u.) =	0.592993
Thermal correction to Gibbs Free Energy (a.u.) =	0.490217
Total Entropy (cal/Kmol) =	216.310
E(RPBE1PBE) (a.u.) =	-2953.37280171

Optimised cartesian coordinates (Angstrom):

P	2.292319	0.045924	0.060760
C	2.483741	-0.815887	1.722933
C	1.226961	-0.590661	2.564860
H	3.390789	-0.489920	2.270304
H	2.603801	-1.900042	1.516548
H	1.207772	-1.298734	3.425013

H	1.194562	0.441007	2.973118
N	0.000006	-0.776407	1.740675
C	-1.226936	-0.590665	2.564879
C	-2.483724	-0.815899	1.722968
H	-1.194535	0.441004	2.973134
H	-1.207728	-1.298737	3.425032
H	-2.603786	-1.900055	1.516588
H	-3.390765	-0.489928	2.270348
P	-2.292326	0.045915	0.060792
Mn	-0.000007	0.208308	-0.151227
C	-0.000023	1.888819	0.353381
O	0.000011	3.028894	0.689274
C	-0.000011	0.666128	-1.850987
O	0.000032	0.978680	-2.993616
C	3.247575	-1.149072	-1.062234
C	4.678079	-1.462600	-0.590384
C	3.225119	-0.751447	-2.547861
H	2.633657	-2.069276	-0.945377
H	4.722510	-1.800176	0.464739
H	5.106670	-2.276831	-1.213776
H	5.351494	-0.586227	-0.699612
H	2.208436	-0.494795	-2.903898
H	3.887704	0.116303	-2.749524
H	3.600646	-1.594132	-3.167219
C	3.401691	1.578293	0.163832
C	3.297395	2.317917	1.509795
C	3.137584	2.536086	-1.013633
H	4.436872	1.179962	0.070983
H	3.578792	1.681233	2.372591
H	3.991841	3.185675	1.507502
H	2.276665	2.714181	1.681800
H	3.196972	2.033070	-1.998688
H	2.135404	3.002142	-0.931351
H	3.890152	3.353772	-1.008849
C	-3.247605	-1.149073	-1.062195
C	-3.225159	-0.751450	-2.547823
C	-4.678108	-1.462587	-0.590334
H	-2.633693	-2.069281	-0.945340
H	-2.208479	-0.494795	-2.903868
H	-3.600688	-1.594136	-3.167178
H	-3.887748	0.116297	-2.749483
H	-4.722535	-1.800171	0.464787
H	-5.351513	-0.586205	-0.699550
H	-5.106714	-2.276809	-1.213728
C	-3.401691	1.578290	0.163883
C	-3.137711	2.535998	-1.013680
C	-3.297238	2.318011	1.509777
H	-4.436883	1.179956	0.071171
H	-3.197209	2.032912	-1.998692
H	-3.890277	3.353686	-1.008874

H	-2.135521	3.002058	-0.931542
H	-3.578556	1.681396	2.372651
H	-2.276478	2.714263	1.681641
H	-3.991664	3.185786	1.507494
C	-0.000012	-2.148338	1.070357
N	-0.000002	-1.874869	-0.231938
O	0.000043	-3.192845	1.733573
C	0.000003	-2.940097	-1.214886
H	-0.890136	-2.876296	-1.879702
H	0.890285	-2.876461	-1.879531
H	-0.000129	-3.936481	-0.723182

4C

Frequencies, energies and thermodynamic properties:

Lowest Vibrational Mode (1/cm) = 32.0446
2nd Lowest Vibrational Mode (1/cm) = 38.1007
E(RB-P86) (a.u.) = -2747.05386515
Thermal correction to Enthalpy (a.u.) = 0.555146
Thermal correction to Gibbs Free Energy (a.u.) = 0.461618
Total Entropy (cal/Kmol) = 196.847
E(RPBE1PBE) (a.u.) = -2746.66880808

Optimised cartesian coordinates (Angstrom):

P	2.226191	0.065118	-0.311783
C	2.447454	-1.089199	-1.788423
C	1.242382	-2.027688	-1.850778
H	3.400715	-1.654890	-1.761919
H	2.474410	-0.453930	-2.701066
H	1.236314	-2.602783	-2.807121
H	1.264917	-2.762623	-1.019142
N	0.000033	-1.230590	-1.710926
C	-1.242419	-2.027489	-1.850985
C	-2.447366	-1.088835	-1.788669
H	-1.265147	-2.762502	-1.019423
H	-1.236329	-2.602497	-2.807381
H	-2.474090	-0.453419	-2.701217
H	-3.400713	-1.654393	-1.762392
P	-2.226147	0.065228	-0.311835
Mn	0.000013	0.036190	0.050281
C	-0.000009	-1.286419	1.238568
O	-0.000216	-2.113518	2.095569
C	0.000043	1.291836	1.268933
O	-0.000373	2.166270	2.073518
C	3.041980	1.644850	-0.981737
C	4.469550	1.461049	-1.523246
C	2.963136	2.830749	-0.006060
H	2.360153	1.865667	-1.834641
H	4.549023	0.640664	-2.265385
H	4.805985	2.393545	-2.026435
H	5.196949	1.256122	-0.709080
H	1.948278	2.956224	0.419148

H	3.676969	2.714244	0.836625
H	3.234569	3.772049	-0.531009
C	3.459723	-0.575224	0.983464
C	3.458343	-2.109105	1.105497
C	3.188504	0.073466	2.353384
H	4.463872	-0.254515	0.625390
H	3.739680	-2.617986	0.161341
H	4.197709	-2.421545	1.874684
H	2.467357	-2.488572	1.427661
H	3.180311	1.180360	2.311593
H	2.207342	-0.253023	2.754567
H	3.972123	-0.230982	3.080700
C	-3.041912	1.645089	-0.981514
C	-2.962880	2.830892	-0.005735
C	-4.469559	1.461437	-1.522876
H	-2.360170	1.865952	-1.834473
H	-1.947965	2.956269	0.419371
H	-3.234305	3.772256	-0.530573
H	-3.676629	2.714367	0.837017
H	-4.549163	0.641168	-2.265127
H	-5.196870	1.256427	-0.708653
H	-4.806006	2.394025	-2.025887
C	-3.459705	-0.575373	0.983253
C	-3.188299	0.072789	2.353388
C	-3.458557	-2.109295	1.104749
H	-4.463825	-0.254386	0.625343
H	-3.179921	1.179696	2.311989
H	-3.971926	-0.231783	3.080644
H	-2.207170	-0.254010	2.754400
H	-3.740024	-2.617799	0.160428
H	-2.467614	-2.489031	1.426731
H	-4.197931	-2.421889	1.873866
H	0.000012	1.240765	-1.032352
H	0.000147	-0.526217	-2.464469

4D

Frequencies, energies and thermodynamic properties:

Lowest Vibrational Mode (1/cm) = 34.5536

2nd Lowest Vibrational Mode (1/cm) = 38.4223

E(RB-P86) (a.u.) = -2859.11323612

Thermal correction to Enthalpy (a.u.) = 0.544459

Thermal correction to Gibbs Free Energy (a.u.) = 0.445854

Total Entropy (cal/Kmol) = 207.531

E(RPBE1PBE) (a.u.) = -2858.73248100

Optimised cartesian coordinates (Angstrom):

P	-2.280753	0.044157	0.225589
C	-2.444771	-0.576815	1.979865
C	-1.199273	-1.412676	2.299744
H	-3.390828	-1.128333	2.154863
H	-2.453594	0.330960	2.620929

H	-1.195546	-1.642444	3.398265
H	-1.291887	-2.420735	1.796953
N	0.000024	-0.683876	1.941629
C	1.199342	-1.412657	2.299712
C	2.444817	-0.576768	1.979811
H	1.291963	-2.420711	1.796912
H	1.195646	-1.642431	3.398232
H	2.453628	0.331008	2.620874
H	3.390891	-1.128264	2.154792
P	2.280761	0.044197	0.225536
Mn	0.000001	0.098976	-0.055958
C	0.000032	-1.642359	-0.574018
O	0.000141	-2.781189	-0.855363
C	-0.000046	0.767831	-1.700693
O	0.000007	1.194390	-2.802394
C	-3.207302	1.695395	0.280531
C	-4.636200	1.593105	0.841545
C	-3.176586	2.450609	-1.058830
H	-2.593515	2.268663	1.010255
H	-4.673120	1.096630	1.832125
H	-5.060517	2.612476	0.966651
H	-5.315945	1.044143	0.155856
H	-2.151515	2.534731	-1.471320
H	-3.815192	1.958488	-1.822309
H	-3.571333	3.479728	-0.919941
C	-3.382597	-1.044715	-0.872529
C	-3.483574	-2.508371	-0.408154
C	-2.958987	-0.959148	-2.350219
H	-4.392219	-0.587510	-0.772365
H	-3.878415	-2.604498	0.622605
H	-4.180143	-3.056746	-1.078522
H	-2.505409	-3.027188	-0.454375
H	-2.864060	0.083729	-2.710984
H	-1.983704	-1.459187	-2.519319
H	-3.712564	-1.470538	-2.986901
C	3.207280	1.695463	0.280450
C	3.176425	2.450742	-1.058871
C	4.636228	1.593206	0.841347
H	2.593541	2.268689	1.010246
H	2.151322	2.534810	-1.471295
H	3.571102	3.479884	-0.919952
H	3.815023	1.958713	-1.822414
H	4.673249	1.096688	1.831900
H	5.315944	1.044309	0.155578
H	5.060510	2.612590	0.966467
C	3.382567	-1.044685	-0.872597
C	2.958638	-0.959438	-2.350211
C	3.483797	-2.508253	-0.408005
H	4.392146	-0.587328	-0.772682
H	2.863503	0.083360	-2.711147

H	3.712140	-1.470853	-2.986961
H	1.983377	-1.459629	-2.519009
H	3.878812	-2.604163	0.622706
H	2.505685	-3.027195	-0.453987
H	4.180326	-3.056650	-1.078396
C	0.000017	1.711796	0.789696
O	0.000046	2.722880	1.378367

4E

Frequencies, energies and thermodynamic properties:

Lowest Vibrational Mode (1/cm) = 26.4314

2nd Lowest Vibrational Mode (1/cm) = 33.8029

E(RB-P86) (a.u.) = -2745.85675886

Thermal correction to Enthalpy (a.u.) = 0.534071

Thermal correction to Gibbs Free Energy (a.u.) = 0.438805

Total Entropy (cal/Kmol) = 200.504

E(RPBE1PBE) (a.u.) = -2745.47148106

Optimised cartesian coordinates (Angstrom):

P	2.254234	-0.122734	-0.230244
C	2.468350	-1.209919	-1.730164
C	1.194650	-2.046209	-1.889433
H	3.381446	-1.840217	-1.686652
H	2.577393	-0.518600	-2.592959
H	1.044958	-2.289796	-2.970505
H	1.314440	-3.038614	-1.386890
N	-0.000188	-1.359479	-1.363623
C	-1.195128	-2.044860	-1.890946
C	-2.468515	-1.208146	-1.731302
H	-1.315732	-3.037867	-1.389776
H	-1.044909	-2.287143	-2.972243
H	-2.577043	-0.515986	-2.593488
H	-3.381896	-1.838091	-1.688618
P	-2.254287	-0.122377	-0.230405
Mn	-0.000062	0.129390	-0.180899
C	0.000009	0.735528	1.480836
O	-0.000277	1.128932	2.601899
C	-0.000164	1.738703	-0.856453
O	-0.000243	2.788429	-1.411860
C	3.377939	1.346524	-0.627335
C	4.861135	0.974529	-0.792806
C	3.184178	2.550356	0.311320
H	2.976102	1.640394	-1.624333
H	5.013155	0.132672	-1.499192
H	5.428565	1.844500	-1.188347
H	5.326583	0.696904	0.176223
H	2.115256	2.812966	0.431864
H	3.607708	2.366853	1.319451
H	3.705827	3.437819	-0.107046
C	3.128374	-1.098980	1.140214
C	2.401961	-2.433813	1.383997

C 3.282175 -0.307920 2.450546
H 4.141930 -1.318730 0.734494
H 2.405522 -3.088629 0.489768
H 2.900095 -2.993014 2.204992
H 1.344909 -2.265482 1.678866
H 3.919859 0.590216 2.331906
H 2.301679 0.020349 2.850913
H 3.761939 -0.951063 3.219712
C -3.377940 1.347320 -0.625948
C -3.183817 2.550310 0.313713
C -4.861212 0.975627 -0.791413
H -2.976305 1.642030 -1.622780
H -2.114835 2.812711 0.434198
H -3.705499 3.438188 -0.103727
H -3.607084 2.365960 1.321800
H -5.013468 0.134394 -1.498493
H -5.326477 0.697214 0.177477
H -5.428638 1.845997 -1.186080
C -3.128176 -1.099835 1.139339
C -3.280754 -0.310153 2.450647
C -2.402302 -2.435321 1.381146
H -4.142090 -1.318573 0.733969
H -3.917943 0.588514 2.333369
H -3.760470 -0.953855 3.219374
H -2.299829 0.017088 2.850815
H -2.407375 -3.089366 0.486363
H -1.344772 -2.267897 1.674818
H -2.899791 -2.994904 2.202269

4F
Frequencies, energies and thermodynamic properties:
Lowest Vibrational Mode (1/cm) = 32.7089
2nd Lowest Vibrational Mode (1/cm) = 35.8506
E(RB-P86) (a.u.) = -2954.91266074
Thermal correction to Enthalpy (a.u.) = 0.612591
Thermal correction to Gibbs Free Energy (a.u.) = 0.505325
Total Entropy (cal/Kmol) = 225.761
E(RPBE1PBE) (a.u.) = -2954.53720090
Optimised cartesian coordinates (Angstrom):
P 2.303608 -0.130168 -0.103902
C 2.500266 0.400790 -1.893446
C 1.248227 -0.021122 -2.664673
H 3.424239 0.010940 -2.365230
H 2.568844 1.508891 -1.879603
H 1.230518 0.471937 -3.665390
H 1.237527 -1.118116 -2.845027
N 0.037790 0.350081 -1.891408
C -1.208672 0.096684 -2.654934
C -2.423173 0.535591 -1.834669
H -1.263214 -0.985785 -2.901329

H	-1.181458	0.647088	-3.625090
H	-2.409541	1.639976	-1.716798
H	-3.376028	0.260841	-2.330838
P	-2.259261	-0.163197	-0.103360
Mn	0.024299	-0.342801	0.102486
C	0.046611	-2.044148	-0.275566
O	0.062970	-3.205548	-0.516037
C	0.016458	-0.702858	1.832185
O	0.008757	-0.964749	2.984312
C	3.166409	1.295590	0.801290
C	4.630140	1.511178	0.380677
C	3.016660	1.232973	2.329777
H	2.567387	2.158145	0.431561
H	4.757685	1.576085	-0.719096
H	5.004950	2.463784	0.812889
H	5.293470	0.703986	0.756958
H	1.963349	1.095473	2.642946
H	3.619006	0.410928	2.770276
H	3.380799	2.180836	2.780230
C	3.446813	-1.619907	0.139525
C	3.453797	-2.589714	-1.055485
C	3.121592	-2.358466	1.451055
H	4.463256	-1.175751	0.225941
H	3.775047	-2.105813	-1.999509
H	4.171324	-3.413758	-0.852877
H	2.459470	-3.051735	-1.216970
H	3.088796	-1.683189	2.328659
H	2.142285	-2.874561	1.387590
H	3.896081	-3.130168	1.648882
C	-3.194796	1.113661	0.939710
C	-3.259148	0.747083	2.432476
C	-4.585166	1.471699	0.387393
H	-2.535377	2.004918	0.853857
H	-2.268737	0.466652	2.841277
H	-3.625406	1.621250	3.011742
H	-3.964995	-0.088110	2.624368
H	-4.556444	1.813441	-0.666720
H	-5.293164	0.618165	0.453371
H	-5.020480	2.298672	0.988711
C	-3.364262	-1.701978	-0.050911
C	-3.051613	-2.579833	1.176012
C	-3.318363	-2.532566	-1.345730
H	-4.394302	-1.293820	0.058652
H	-3.057832	-2.011852	2.126199
H	-3.811960	-3.385644	1.261293
H	-2.059772	-3.064668	1.079486
H	-3.631930	-1.955593	-2.238615
H	-2.307519	-2.948753	-1.530970
H	-4.015362	-3.393177	-1.253748
H	0.114072	1.417977	0.578784

C	-0.099595	2.593706	0.371702
H	0.091716	1.420797	-1.678852
N	0.103960	2.887398	-0.889379
O	-0.428823	3.220251	1.392213
C	-0.068973	4.282236	-1.277816
H	0.883028	4.856583	-1.184864
H	-0.380767	4.347132	-2.341901
H	-0.825974	4.810908	-0.653308

4G

Frequencies, energies and thermodynamic properties:

Lowest Vibrational Mode (1/cm) = 27.3327
2nd Lowest Vibrational Mode (1/cm) = 30.3443
E(RB-P86) (a.u.) = -2954.91393160
Thermal correction to Enthalpy (a.u.) = 0.613626
Thermal correction to Gibbs Free Energy (a.u.) = 0.506243
Total Entropy (cal/Kmol) = 226.008
E(RPBE1PBE) (a.u.) = -2954.53343599

Optimised cartesian coordinates (Angstrom):

P	-2.273932	-0.204790	0.148088
C	-2.452288	0.022450	1.997838
C	-1.193098	-0.558201	2.652900
H	-3.389816	-0.410143	2.404813
H	-2.490234	1.120092	2.170510
H	-1.155841	-0.224621	3.724853
H	-1.283778	-1.680657	2.708158
N	-0.000055	-0.118664	1.941137
C	1.193100	-0.557770	2.652977
C	2.452156	0.023061	1.997824
H	1.284058	-1.680194	2.708438
H	1.155754	-0.224010	3.724872
H	2.489816	1.120754	2.170230
H	3.389776	-0.409205	2.404940
P	2.273943	-0.204657	0.148122
Mn	0.000004	-0.311472	-0.105900
C	0.000007	-2.040988	-0.076712
O	0.000002	-3.232335	-0.044789
C	0.000037	-0.297419	-1.881861
O	0.000172	-0.359445	-3.068140
C	-3.214578	1.301831	-0.526336
C	-4.624141	1.485195	0.061637
C	-3.234010	1.367688	-2.062761
H	-2.572068	2.133451	-0.162780
H	-4.628807	1.500733	1.170323
H	-5.056048	2.449540	-0.284599
H	-5.319112	0.684797	-0.271055
H	-2.229538	1.207618	-2.501487
H	-3.922963	0.612015	-2.495858
H	-3.598132	2.363844	-2.395435
C	-3.391106	-1.666312	-0.316680

C	-3.376188	-2.803044	0.721294
C	-3.051112	-2.205523	-1.719236
H	-4.418919	-1.238773	-0.342043
H	-3.703745	-2.470631	1.726617
H	-4.073018	-3.607051	0.398449
H	-2.368215	-3.254920	0.813344
H	-3.033464	-1.413238	-2.492610
H	-2.058910	-2.699596	-1.724422
H	-3.806839	-2.960872	-2.026161
C	3.214590	1.301800	-0.526650
C	3.233935	1.367359	-2.063086
C	4.624205	1.485218	0.061187
H	2.572098	2.133488	-0.163224
H	2.229440	1.207189	-2.501728
H	3.598021	2.363457	-2.395972
H	3.922877	0.611615	-2.496079
H	4.628955	1.501069	1.169866
H	5.319088	0.684671	-0.271333
H	5.056164	2.449432	-0.285349
C	3.391175	-1.666281	-0.316167
C	3.051366	-2.205797	-1.718653
C	3.376109	-2.802784	0.722054
H	4.418996	-1.238754	-0.341483
H	3.033856	-1.413685	-2.492207
H	3.807116	-2.961234	-2.025305
H	2.059152	-2.699845	-1.723870
H	3.703533	-2.470147	1.727346
H	2.368121	-3.254635	0.814068
H	4.072977	-3.606867	0.399480
O	0.000170	1.949927	-0.157147
C	-0.000292	2.635160	0.888495
H	-0.000655	2.107765	1.877299
N	-0.000356	3.982704	0.908307
C	0.000142	4.832380	-0.275189
H	0.900957	5.480268	-0.299095
H	-0.000057	4.182220	-1.168952
H	-0.900102	5.481044	-0.299227
H	-0.000700	4.430889	1.828277

4H

Frequencies, energies and thermodynamic properties:

Lowest Vibrational Mode (1/cm) = 20.6977
2nd Lowest Vibrational Mode (1/cm) = 35.1860
E(RB-P86) (a.u.) = -2954.89809346
Thermal correction to Enthalpy (a.u.) = 0.613882
Thermal correction to Gibbs Free Energy (a.u.) = 0.507180
Total Entropy (cal/Kmol) = 224.573
E(RPBE1PBE) (a.u.) = -2954.51580017

Optimised cartesian coordinates (Angstrom):

P	-2.280904	-0.168996	0.128967

C	-2.457454	0.064242	1.983196
C	-1.233696	-0.542905	2.676108
H	-3.405513	-0.343651	2.389017
H	-2.466025	1.162425	2.153385
H	-1.215695	-0.238317	3.751003
H	-1.274534	-1.654707	2.660397
N	-0.002460	-0.089540	1.995435
C	1.225355	-0.552546	2.675829
C	2.453764	0.045402	1.983173
H	1.257726	-1.664620	2.659599
H	1.209784	-0.248335	3.750866
H	2.471246	1.143356	2.154232
H	3.398600	-0.370249	2.388617
P	2.275398	-0.184995	0.128680
Mn	-0.003306	-0.303258	-0.120960
C	-0.009352	-2.047615	-0.065981
O	-0.013544	-3.237416	-0.032724
C	-0.003439	-0.343672	-1.886738
O	-0.003582	-0.406208	-3.069536
C	-3.174897	1.362068	-0.537062
C	-4.587448	1.573342	0.032988
C	-3.167973	1.440300	-2.072964
H	-2.509860	2.167644	-0.156080
H	-4.608566	1.580983	1.141596
H	-4.988598	2.551833	-0.310022
H	-5.298345	0.794544	-0.317249
H	-2.157841	1.270768	-2.494481
H	-3.861170	0.700711	-2.526748
H	-3.509111	2.445985	-2.400587
C	-3.420606	-1.613714	-0.323181
C	-3.412470	-2.749316	0.715960
C	-3.095373	-2.156445	-1.727842
H	-4.442235	-1.171385	-0.341502
H	-3.731664	-2.413185	1.722950
H	-4.121168	-3.544719	0.398745
H	-2.410372	-3.216042	0.801260
H	-3.074175	-1.363796	-2.500917
H	-2.108881	-2.662227	-1.739924
H	-3.861330	-2.902829	-2.030350
C	3.181209	1.340117	-0.535472
C	3.174451	1.420653	-2.071255
C	4.595693	1.538926	0.034291
H	2.523190	2.150610	-0.152752
H	2.162988	1.259643	-2.492956
H	3.523549	2.424038	-2.397569
H	3.861676	0.676248	-2.526239
H	4.617325	1.544026	1.142898
H	5.300207	0.755245	-0.317929
H	5.004450	2.514918	-0.306850
C	3.404565	-1.637415	-0.325392

C 3.074165 -2.176513 -1.730237
C 3.389298 -2.773707 0.712874
H 4.429279 -1.202346 -0.344314
H 3.058395 -1.383073 -2.502632
H 3.834201 -2.928434 -2.033966
H 2.083819 -2.674686 -1.741974
H 3.711847 -2.440600 1.719800
H 2.383973 -3.233297 0.798820
H 4.091994 -3.573911 0.394388
O 0.005127 1.827697 -0.099763
C 0.009523 2.452150 1.030380
H 0.001669 1.019965 1.985525
N 0.031925 3.813751 0.945488
C 0.050692 4.625454 -0.272778
H -0.871018 5.239510 -0.374907
H 0.923969 5.312605 -0.289383
H 0.117109 3.938628 -1.137048
H 0.028045 4.313147 1.836952

4I
Frequencies, energies and thermodynamic properties:
Lowest Vibrational Mode (1/cm) = 36.4316
2nd Lowest Vibrational Mode (1/cm) = 45.1599
E(RB-P86) (a.u.) = -2954.91851220
Thermal correction to Enthalpy (a.u.) = 0.615875
Thermal correction to Gibbs Free Energy (a.u.) = 0.511611
Total Entropy (cal/Kmol) = 219.442
E(RPBE1PBE) (a.u.) = -2954.54189833
Optimised cartesian coordinates (Angstrom):
P -2.280513 -0.071044 0.164325
C -2.463458 0.024879 2.034612
C -1.236281 -0.612699 2.684276
H -3.401476 -0.432705 2.408670
H -2.503907 1.106137 2.292126
H -1.237026 -0.437421 3.785994
H -1.224695 -1.710977 2.525194
N -0.005596 -0.049904 2.070840
C 1.235742 -0.587214 2.687382
C 2.478193 -0.012189 2.002702
H 1.208912 -1.690644 2.571513
H 1.254649 -0.372646 3.781717
H 2.602249 1.063933 2.258975
H 3.396638 -0.516327 2.366426
P 2.262454 -0.136536 0.130959
Mn -0.000869 -0.183252 -0.096036
C -0.028753 -1.963003 -0.050205
O -0.043266 -3.151406 -0.087533
C -0.014155 -0.204797 -1.854777
O -0.026030 -0.236265 -3.037398
C -3.300057 1.432705 -0.385344

C	-4.698503	1.525776	0.248408
C	-3.357620	1.567246	-1.916048
H	-2.678502	2.272082	-0.005823
H	-4.676978	1.495688	1.356764
H	-5.175607	2.486451	-0.044097
H	-5.373085	0.715247	-0.101527
H	-2.339526	1.554140	-2.350118
H	-3.972448	0.767659	-2.380965
H	-3.828551	2.537060	-2.187568
C	-3.381329	-1.526037	-0.381232
C	-3.416171	-2.701103	0.611664
C	-2.994977	-2.011817	-1.791160
H	-4.405507	-1.093227	-0.429497
H	-3.786265	-2.407557	1.614638
H	-4.105534	-3.485278	0.229748
H	-2.418855	-3.170978	0.726398
H	-2.928292	-1.186045	-2.526005
H	-2.014328	-2.527721	-1.780347
H	-3.753514	-2.735282	-2.161196
C	3.284929	1.350099	-0.469659
C	3.239303	1.509793	-1.999003
C	4.724654	1.412759	0.067140
H	2.712822	2.196879	-0.031553
H	2.203174	1.466071	-2.388318
H	3.669813	2.492546	-2.288226
H	3.839763	0.729569	-2.512469
H	4.777921	1.338663	1.172645
H	5.364308	0.611250	-0.359459
H	5.189995	2.380457	-0.220354
C	3.336378	-1.617314	-0.393821
C	2.952117	-2.098559	-1.805786
C	3.324750	-2.787884	0.605018
H	4.371891	-1.211855	-0.430297
H	2.933151	-1.277994	-2.549263
H	3.685266	-2.856406	-2.157214
H	1.950409	-2.572480	-1.806081
H	3.691803	-2.500750	1.611002
H	2.313044	-3.227975	0.709697
H	3.996185	-3.592673	0.234646
O	-0.440587	2.593779	-1.153957
C	-0.020799	1.923350	-0.190743
H	-0.044059	0.972612	2.232803
N	0.367981	2.679450	0.948148
C	0.386790	4.141249	0.908040
H	-0.585480	4.511676	0.529555
H	0.550812	4.538209	1.929812
H	1.175944	4.550361	0.234911
H	1.095344	2.268109	1.538201

4Ja

Frequencies, energies and thermodynamic properties:

Lowest Vibrational Mode (1/cm) =	35.1283
2nd Lowest Vibrational Mode (1/cm) =	46.9614
E(RB-P86) (a.u.) =	-2860.30406773
Thermal correction to Enthalpy (a.u.) =	0.567567
Thermal correction to Gibbs Free Energy (a.u.) =	0.469493
Total Entropy (cal/Kmol) =	206.416
E(RPBE1PBE) (a.u.) =	-2859.92499714

Optimised cartesian coordinates (Angstrom):

P	2.264528	0.006436	-0.240326
C	2.462204	-0.732026	-1.960466
C	1.239010	-1.593343	-2.275957
H	3.401503	-1.308097	-2.083300
H	2.512220	0.123403	-2.669840
H	1.237438	-1.895731	-3.349406
H	1.239449	-2.524181	-1.672215
N	-0.000012	-0.839080	-1.947908
C	-1.239032	-1.593357	-2.275938
C	-2.462232	-0.732050	-1.960442
H	-1.239456	-2.524188	-1.672185
H	-1.237467	-1.895756	-3.349383
H	-2.512273	0.123366	-2.669829
H	-3.401525	-1.308136	-2.083251
P	-2.264537	0.006429	-0.240308
Mn	-0.000004	0.006483	0.033351
C	0.000007	-1.608314	0.825631
O	0.000123	-2.628862	1.429167
C	0.000016	0.836635	1.582677
O	-0.000059	1.392336	2.627724
C	3.201414	1.644780	-0.439072
C	4.653046	1.502640	-0.927382
C	3.101932	2.541637	0.805943
H	2.608876	2.131024	-1.246364
H	4.744356	0.877271	-1.839000
H	5.064048	2.505479	-1.173841
H	5.314005	1.068700	-0.147449
H	2.050832	2.688599	1.121088
H	3.676820	2.126920	1.660446
H	3.531241	3.541951	0.581385
C	3.394223	-1.018921	0.890826
C	3.401192	-2.521769	0.560440
C	3.044704	-0.782169	2.371955
H	4.416429	-0.622903	0.700904
H	3.747337	-2.733827	-0.471182
H	4.097805	-3.046426	1.249650
H	2.400354	-2.978079	0.697227
H	3.013317	0.292405	2.639087
H	2.056124	-1.216908	2.622088
H	3.803180	-1.270222	3.021313
C	-3.201392	1.644789	-0.439081

C	-3.101866	2.541687	0.805901
C	-4.653035	1.502667	-0.927361
H	-2.608855	2.130988	-1.246401
H	-2.050752	2.688684	1.120981
H	-3.531210	3.541983	0.581330
H	-3.676701	2.126986	1.660447
H	-4.744382	0.877231	-1.838929
H	-5.313996	1.068809	-0.147383
H	-5.064005	2.505500	-1.173889
C	-3.394250	-1.018915	0.890835
C	-3.044722	-0.782189	2.371964
C	-3.401254	-2.521761	0.560430
H	-4.416448	-0.622874	0.700917
H	-3.013383	0.292380	2.639124
H	-3.803169	-1.270291	3.021319
H	-2.056120	-1.216895	2.622071
H	-3.747394	-2.733798	-0.471197
H	-2.400433	-2.978103	0.697224
H	-4.097893	-3.046404	1.249624
O	0.000026	2.957459	-0.547351
C	-0.000003	1.782475	-0.913251
H	-0.000016	1.658145	-2.091932
H	-0.000021	0.019086	-2.540377

4J
Frequencies, energies and thermodynamic properties:

Lowest Vibrational Mode (1/cm) =	36.3908
2nd Lowest Vibrational Mode (1/cm) =	47.6776
E(RB-P86) (a.u.) =	-2860.31214061
Thermal correction to Enthalpy (a.u.) =	0.568572
Thermal correction to Gibbs Free Energy (a.u.) =	0.471015
Total Entropy (cal/Kmol) =	205.327
E(RPBE1PBE) (a.u.) =	-2859.93240650

Optimised cartesian coordinates (Angstrom):

P	2.264590	-0.077792	0.175304
C	2.454635	0.203530	2.024147
C	1.235338	0.972946	2.534865
H	3.401034	0.716070	2.290010
H	2.482979	-0.803132	2.494238
H	1.229310	0.991081	3.650902
H	1.256742	2.029097	2.191508
N	-0.000082	0.339793	2.020532
C	-1.235333	0.973364	2.534734
C	-2.454822	0.204207	2.024062
H	-1.256482	2.029491	2.191277
H	-1.229341	0.991647	3.650772
H	-2.483636	-0.802299	2.494463
H	-3.401090	0.717165	2.289563
P	-2.264614	-0.077718	0.175268
Mn	-0.000013	-0.010638	-0.104183

C	0.000032	1.736926	-0.526429
O	-0.000356	2.873812	-0.860503
C	-0.000113	-0.481733	-1.796774
O	0.000030	-0.810024	-2.936735
C	3.192893	-1.720378	-0.040292
C	4.626709	-1.729075	0.516172
C	3.145289	-2.255632	-1.481468
H	2.572060	-2.392365	0.593698
H	4.682605	-1.386240	1.569246
H	5.034708	-2.762512	0.484633
H	5.310809	-1.095599	-0.087489
H	2.123088	-2.227926	-1.907807
H	3.812612	-1.677659	-2.154956
H	3.496066	-3.309740	-1.504839
C	3.406840	1.191886	-0.654439
C	3.390584	2.571120	0.027899
C	3.089915	1.316808	-2.156377
H	4.429193	0.766611	-0.542138
H	3.710503	2.529604	1.088524
H	4.096641	3.250176	-0.497366
H	2.387859	3.041207	-0.018987
H	3.090943	0.339600	-2.677984
H	2.095487	1.779923	-2.316743
H	3.847533	1.963174	-2.649754
C	-3.193061	-1.720312	-0.039787
C	-3.145485	-2.256030	-1.480790
C	-4.626867	-1.728739	0.516673
H	-2.572260	-2.392144	0.594409
H	-2.123257	-2.228639	-1.907088
H	-3.496466	-3.310076	-1.503896
H	-3.812641	-1.678113	-2.154492
H	-4.682807	-1.385476	1.569603
H	-5.310903	-1.095483	-0.087290
H	-5.034903	-2.762174	0.485534
C	-3.406621	1.191838	-0.654997
C	-3.089321	1.316310	-2.156891
C	-3.390291	2.571262	0.026922
H	-4.429051	0.766720	-0.542788
H	-3.089863	0.338914	-2.678140
H	-3.846971	1.962281	-2.650739
H	-2.094970	1.779638	-2.317130
H	-3.710563	2.530132	1.087454
H	-2.387444	3.041120	-0.019770
H	-4.096032	3.250314	-0.498776
O	0.000147	-2.353957	1.668898
C	-0.000014	-1.943479	0.484686
H	-0.000359	-2.777356	-0.294096
H	-0.000265	-0.674546	2.303922

4TSBBa

Frequencies, energies and thermodynamic properties:
Lowest Vibrational Mode (1/cm) = -80.2554
2nd Lowest Vibrational Mode (1/cm) = 15.8993
E(RB-P86) (a.u.) = -2953.68860746
Thermal correction to Enthalpy (a.u.) = 0.591047
Thermal correction to Gibbs Free Energy (a.u.) = 0.488243
Total Entropy (cal/Kmol) = 216.370
E(RPBE1PBE) (a.u.) = -2953.31701068

Optimised cartesian coordinates (Angstrom):

P	2.326398	-0.012574	-0.037788
C	2.536635	1.012780	-1.590872
C	1.187682	1.119574	-2.310920
H	3.286703	0.565018	-2.273553
H	2.885863	2.011214	-1.277296
H	1.185913	2.005629	-2.986062
H	1.043378	0.224021	-2.948455
N	-0.031363	1.184010	-1.420900
C	-1.233996	1.024501	-2.318375
C	-2.566283	0.770094	-1.606728
H	-1.003047	0.164481	-2.980397
H	-1.317269	1.929817	-2.960147
H	-2.993757	1.732253	-1.273678
H	-3.272369	0.265110	-2.296403
P	-2.260888	-0.247464	-0.068056
Mn	0.037884	-0.333562	0.076306
C	0.127145	-1.750471	-0.899584
O	0.192051	-2.727897	-1.568485
C	0.076693	-1.343945	1.527097
O	0.101842	-2.021887	2.492126
C	3.101195	1.084124	1.298811
C	4.566756	1.458242	1.021399
C	2.915120	0.541685	2.724784
H	2.469538	1.991068	1.169268
H	4.722033	1.860293	-0.000550
H	4.889518	2.246240	1.735616
H	5.253130	0.595789	1.160562
H	1.862615	0.269619	2.939823
H	3.545060	-0.352071	2.919077
H	3.218625	1.317238	3.460376
C	3.498531	-1.495931	-0.194894
C	3.601079	-2.070593	-1.619128
C	3.141511	-2.600610	0.816749
H	4.493751	-1.079464	0.077655
H	3.985206	-1.335404	-2.353892
H	4.311350	-2.925211	-1.615920
H	2.626144	-2.452300	-1.982755
H	3.021612	-2.219092	1.849394
H	2.197570	-3.110474	0.536242
H	3.944800	-3.368008	0.831719
C	-3.134021	0.786064	1.259230

C	-2.775735	0.392968	2.700388
C	-4.652704	0.905876	1.055306
H	-2.660367	1.768559	1.030480
H	-1.680096	0.352561	2.862068
H	-3.191978	1.143718	3.406050
H	-3.198198	-0.592539	2.988714
H	-4.924335	1.186978	0.016877
H	-5.184291	-0.036131	1.308065
H	-5.056968	1.695051	1.725420
C	-3.296933	-1.822088	-0.251282
C	-2.938349	-2.857198	0.829810
C	-3.238955	-2.448077	-1.655968
H	-4.339304	-1.475206	-0.075461
H	-2.963745	-2.436487	1.854365
H	-3.659988	-3.701227	0.795750
H	-1.925194	-3.276550	0.663265
H	-3.582333	-1.755413	-2.450066
H	-2.218733	-2.794574	-1.913325
H	-3.909649	-3.333544	-1.686727
C	-0.038883	2.504394	-0.610673
N	-1.241897	2.990638	-0.400298
O	1.089291	2.929233	-0.250531
C	-1.244303	4.238765	0.345038
H	-0.734560	5.068510	-0.204733
H	-2.288212	4.563316	0.544094
H	-0.713933	4.167773	1.326921

4TSCE

Frequencies, energies and thermodynamic properties:

Lowest Vibrational Mode (1/cm) = -523.4275
2nd Lowest Vibrational Mode (1/cm) = 34.5749
E(RB-P86) (a.u.) = -2956.09094397
Thermal correction to Enthalpy (a.u.) = 0.631385
Thermal correction to Gibbs Free Energy (a.u.) = 0.526344
Total Entropy (cal/Kmol) = 221.078
E(RPBE1PBE) (a.u.) = -2955.71317976

Optimised cartesian coordinates (Angstrom):

P	2.361563	0.012335	-0.091266
C	2.474772	0.579536	-1.870607
C	1.280621	-0.004347	-2.627410
H	3.442823	0.332241	-2.351597
H	2.373187	1.685530	-1.844390
H	1.181161	0.503245	-3.617883
H	1.434602	-1.086491	-2.846168
N	0.041135	0.184874	-1.837746
C	-1.138307	-0.225757	-2.632743
C	-2.419556	0.113581	-1.871677
H	-1.085508	-1.315030	-2.863821
H	-1.134400	0.302367	-3.617584
H	-2.529112	1.218363	-1.823932

H	-3.324318	-0.302942	-2.359367
P	-2.207103	-0.446267	-0.100250
Mn	0.092937	-0.340787	0.124504
C	0.268800	-2.059620	0.014677
O	0.392360	-3.237592	-0.079061
C	0.095455	-0.394566	1.894158
O	0.101819	-0.453070	3.077495
C	3.193480	1.449291	0.829986
C	4.560398	1.854949	0.252119
C	3.263015	1.256288	2.354584
H	2.476198	2.276538	0.627927
H	4.520763	2.071041	-0.834253
H	4.920422	2.776309	0.758778
H	5.330988	1.072906	0.420180
H	2.296631	0.931507	2.787487
H	4.035800	0.511864	2.639005
H	3.546587	2.215220	2.839074
C	3.600082	-1.411852	0.080597
C	3.598376	-2.368470	-1.125407
C	3.406223	-2.184934	1.399184
H	4.590474	-0.904238	0.111665
H	3.836721	-1.856329	-2.078979
H	4.372705	-3.151136	-0.973360
H	2.624866	-2.886550	-1.236617
H	3.385223	-1.524136	2.287289
H	2.464213	-2.767943	1.387400
H	4.243124	-2.903209	1.536007
C	-3.310540	0.777293	0.826530
C	-3.340102	0.566026	2.349957
C	-4.733127	0.894679	0.251993
H	-2.769951	1.733421	0.618215
H	-2.328688	0.435222	2.782380
H	-3.804148	1.449908	2.838982
H	-3.952001	-0.315568	2.636504
H	-4.741758	1.124788	-0.832505
H	-5.331894	-0.027674	0.412944
H	-5.272183	1.720722	0.764941
C	-3.123468	-2.100787	0.029777
C	-2.802594	-2.831845	1.347203
C	-2.903103	-3.021575	-1.183566
H	-4.197315	-1.806989	0.043827
H	-2.945056	-2.191359	2.239112
H	-3.470968	-3.712975	1.457741
H	-1.758318	-3.201831	1.357396
H	-3.221470	-2.555499	-2.137551
H	-1.842648	-3.329759	-1.278394
H	-3.505015	-3.947390	-1.056499
H	-0.056926	1.687472	0.322426
C	-0.101718	2.755315	-0.191898
H	-0.062676	1.500918	-1.736848

O	-0.096352	2.663027	-1.537573
H	0.831514	3.259346	0.207216
N	-1.303395	3.386757	0.359389
C	-1.484092	4.764986	-0.100915
H	-0.627401	5.445023	0.145510
H	-1.600036	4.765718	-1.202590
H	-2.404147	5.194333	0.345251
H	-1.236870	3.372215	1.387574

4TSDE

Frequencies, energies and thermodynamic properties:

Lowest Vibrational Mode (1/cm) = -99.2399
2nd Lowest Vibrational Mode (1/cm) = 26.8826
E(RB-P86) (a.u.) = -2859.07484352
Thermal correction to Enthalpy (a.u.) = 0.542975
Thermal correction to Gibbs Free Energy (a.u.) = 0.441716
Total Entropy (cal/Kmol) = 213.117
E(RPBE1PBE) (a.u.) = -2858.69742147

Optimised cartesian coordinates (Angstrom):

P	2.285816	-0.066470	0.131179
C	2.451410	-0.566099	1.924051
C	1.197316	-0.097845	2.664020
H	3.385915	-0.193692	2.392573
H	2.496717	-1.675642	1.932048
H	1.076469	-0.700882	3.599068
H	1.326246	0.959289	3.015332
N	-0.000013	-0.227705	1.827584
C	-1.197338	-0.097907	2.664029
C	-2.451449	-0.566103	1.924051
H	-1.326262	0.959203	3.015421
H	-1.076479	-0.701012	3.599032
H	-2.496820	-1.675643	1.932050
H	-3.385933	-0.193642	2.392572
P	-2.285843	-0.066485	0.131176
Mn	-0.000007	0.086630	-0.084180
C	-0.000014	1.798160	-0.327645
O	-0.000014	2.983313	-0.429939
C	0.000037	-0.192795	-1.838009
O	0.000094	-0.347345	-3.014074
C	3.229301	-1.476444	-0.730275
C	4.624286	-1.748016	-0.139702
C	3.293767	-1.365627	-2.263289
H	2.569275	-2.340061	-0.483813
H	4.608791	-1.908601	0.956846
H	5.052649	-2.664165	-0.600715
H	5.330856	-0.918672	-0.355694
H	2.313581	-1.125957	-2.717306
H	4.024277	-0.594584	-2.585497
H	3.638331	-2.331773	-2.690688
C	3.407305	1.446942	-0.062153

C	3.189203	2.478547	1.059750
C	3.265233	2.104820	-1.447477
H	4.439800	1.041553	0.036320
H	3.382271	2.061896	2.068604
H	3.887696	3.331483	0.918503
H	2.159246	2.888426	1.040745
H	3.426916	1.394273	-2.280746
H	2.262255	2.558135	-1.574568
H	4.015582	2.918066	-1.552118
C	-3.229338	-1.476467	-0.730248
C	-3.293636	-1.365801	-2.263279
C	-4.624399	-1.747892	-0.139788
H	-2.569394	-2.340100	-0.483621
H	-2.313388	-1.126230	-2.717216
H	-3.638209	-2.331970	-2.690619
H	-4.024068	-0.594749	-2.585641
H	-4.609037	-1.908269	0.956795
H	-5.330922	-0.918571	-0.356021
H	-5.052732	-2.664118	-0.600675
C	-3.407320	1.446937	-0.062142
C	-3.265321	2.104768	-1.447495
C	-3.189131	2.478567	1.059721
H	-4.439816	1.041569	0.036402
H	-3.427110	1.394202	-2.280728
H	-4.015635	2.918050	-1.552104
H	-2.262328	2.558023	-1.574681
H	-3.382191	2.061957	2.068593
H	-2.159155	2.888393	1.040674
H	-3.887586	3.331534	0.918468
C	0.000007	-2.709019	0.335853
O	0.000091	-3.469415	1.201346

4TSEB

Frequencies, energies and thermodynamic properties:

Lowest Vibrational Mode (1/cm) = -186.5482
2nd Lowest Vibrational Mode (1/cm) = 32.8789
E(RB-P86) (a.u.) = -2953.68873198
Thermal correction to Enthalpy (a.u.) = 0.589421
Thermal correction to Gibbs Free Energy (a.u.) = 0.484202
Total Entropy (cal/Kmol) = 221.452
E(RPBE1PBE) (a.u.) = -2953.31210796

Optimised cartesian coordinates (Angstrom):

P	2.287588	0.083429	0.135800
C	2.453205	-0.245031	1.968574
C	1.192085	0.298155	2.647261
H	3.388027	0.166780	2.402084
H	2.490190	-1.349826	2.084258
H	1.133894	-0.117463	3.687387
H	1.289667	1.411265	2.787786
N	0.000007	-0.067297	1.892298

C	-1.192064	0.298189	2.647259
C	-2.453197	-0.244980	1.968579
H	-1.289622	1.411302	2.787773
H	-1.133880	-0.117421	3.687388
H	-2.490205	-1.349771	2.084283
H	-3.388010	0.166859	2.402084
P	-2.287582	0.083446	0.135798
Mn	0.000007	0.168520	-0.116643
C	0.000011	1.895736	-0.123924
O	0.000017	3.085650	-0.108616
C	-0.000007	0.117666	-1.892017
O	-0.000024	0.156449	-3.079691
C	3.280851	-1.354586	-0.612591
C	4.666852	-1.565376	0.021613
C	3.375775	-1.297758	-2.147481
H	2.642595	-2.223559	-0.337072
H	4.621407	-1.690564	1.121896
H	5.135306	-2.482868	-0.396231
H	5.356248	-0.722946	-0.199686
H	2.400781	-1.080344	-2.627779
H	4.097220	-0.522478	-2.480770
H	3.743306	-2.269645	-2.541656
C	3.376908	1.593442	-0.228940
C	3.311904	2.672402	0.867189
C	3.068491	2.198530	-1.612107
H	4.413348	1.186693	-0.245868
H	3.624413	2.293305	1.860544
H	3.998309	3.506177	0.603557
H	2.293309	3.099356	0.959860
H	3.088572	1.448266	-2.426022
H	2.071395	2.681317	-1.622516
H	3.822244	2.978357	-1.855588
C	-3.280877	-1.354575	-0.612547
C	-3.375854	-1.297791	-2.147442
C	-4.666862	-1.565338	0.021702
H	-2.642622	-2.223542	-0.337021
H	-2.400885	-1.080341	-2.627791
H	-3.743354	-2.269708	-2.541572
H	-4.097346	-0.522553	-2.480729
H	-4.621391	-1.690494	1.121987
H	-5.356259	-0.722910	-0.199605
H	-5.135333	-2.482839	-0.396104
C	-3.376888	1.593459	-0.228972
C	-3.068470	2.198500	-1.612159
C	-3.311869	2.672450	0.867126
H	-4.413332	1.186721	-0.245884
H	-3.088567	1.448209	-2.426050
H	-3.822211	2.978331	-1.855661
H	-2.071366	2.681272	-1.622589
H	-3.624376	2.293382	1.860493

H -2.293269 3.099397 0.959782
H -3.998267 3.506223 0.603475
C -0.000012 -2.660449 1.086326
N -0.000013 -2.255665 -0.082564
O -0.000032 -3.060568 2.196199
C 0.000024 -3.073276 -1.301623
H 0.893099 -2.825276 -1.906740
H -0.000682 -4.158310 -1.078418
H -0.892284 -2.824212 -1.907417

4TSEJ
Frequencies, energies and thermodynamic properties:
Lowest Vibrational Mode (1/cm) = -933.6928
2nd Lowest Vibrational Mode (1/cm) = 34.1623
E(RB-P86) (a.u.) = -2860.24366002
Thermal correction to Enthalpy (a.u.) = 0.561841
Thermal correction to Gibbs Free Energy (a.u.) = 0.463171
Total Entropy (cal/Kmol) = 207.667
E(RPBE1PBE) (a.u.) = -2859.85919565
Optimised cartesian coordinates (Angstrom):
P -2.274306 -0.029453 -0.173128
C -2.418131 0.059808 -2.033588
C -1.193544 0.800987 -2.576732
H -3.371835 0.510940 -2.376528
H -2.384810 -0.998885 -2.369637
H -1.173540 0.698280 -3.692888
H -1.302107 1.902235 -2.380573
N 0.035997 0.275287 -2.003048
C 1.214632 0.943470 -2.528443
C 2.492402 0.245990 -2.049588
H 1.247764 2.029772 -2.237487
H 1.197403 0.934292 -3.648801
H 2.569243 -0.756364 -2.523659
H 3.408423 0.811712 -2.314195
P 2.328216 -0.055425 -0.210397
Mn 0.033687 -0.059517 0.073190
C 0.044749 1.630864 0.594519
O 0.068124 2.766295 0.920327
C 0.060637 -0.626494 1.754430
O 0.081951 -0.972362 2.885221
C -3.318942 -1.566047 0.227125
C -4.754467 -1.505594 -0.323422
C -3.300723 -1.964662 1.712284
H -2.770489 -2.345517 -0.342269
H -4.789999 -1.258943 -1.403777
H -5.240880 -2.496892 -0.196594
H -5.383514 -0.768606 0.219603
H -2.275863 -1.992350 2.131769
H -3.907697 -1.274239 2.334735
H -3.741861 -2.977533 1.831760

C -3.316973 1.398237 0.513635
C -3.178557 2.708722 -0.280856
C -3.066626 1.624808 2.016538
H -4.362119 1.038555 0.385889
H -3.453470 2.590334 -1.347972
H -3.862578 3.471078 0.151067
H -2.151374 3.120673 -0.228164
H -3.166360 0.697237 2.613140
H -2.056116 2.039281 2.201113
H -3.804020 2.356722 2.411048
C 3.277184 -1.688683 -0.009039
C 3.280124 -2.231479 1.429996
C 4.698167 -1.663575 -0.598715
H 2.661003 -2.374211 -0.634979
H 2.271994 -2.236053 1.889616
H 3.661674 -3.274742 1.436796
H 3.950060 -1.638034 2.086693
H 4.721173 -1.322748 -1.653112
H 5.378841 -1.012045 -0.011114
H 5.129877 -2.687163 -0.571982
C 3.429875 1.220026 0.654299
C 3.114814 1.312906 2.159294
C 3.400168 2.609533 -0.007261
H 4.457312 0.809159 0.535181
H 3.106483 0.324965 2.659703
H 3.882583 1.938452 2.663144
H 2.129287 1.788221 2.335473
H 3.717400 2.585392 -1.068787
H 2.394512 3.071477 0.046720
H 4.102890 3.283498 0.528466
O -0.841642 -2.862461 -1.367593
C -0.102520 -2.204719 -0.631156
H 0.532952 -2.754805 0.121717
H 0.133847 -1.075412 -1.280245

4TSFC
Frequencies, energies and thermodynamic properties:
Lowest Vibrational Mode (1/cm) = -266.9207
2nd Lowest Vibrational Mode (1/cm) = 7.3093
E(RB-P86) (a.u.) = -2954.89310775
Thermal correction to Enthalpy (a.u.) = 0.609617
Thermal correction to Gibbs Free Energy (a.u.) = 0.500306
Total Entropy (cal/Kmol) = 230.064
E(RPBE1PBE) (a.u.) = -2954.50968348
Optimised cartesian coordinates (Angstrom):
P 2.243768 -0.170018 -0.079521
C 2.454935 0.555364 -1.803902
C 1.235503 0.166828 -2.641826
H 3.401071 0.251241 -2.295837
H 2.486092 1.659519 -1.681298

H	1.223246	0.737337	-3.601396
H	1.257957	-0.912648	-2.902663
N	-0.000348	0.429757	-1.866048
C	-1.236015	0.165662	-2.641733
C	-2.455689	0.553702	-1.803928
H	-1.257726	-0.913937	-2.902131
H	-1.224166	0.735796	-3.601528
H	-2.487741	1.657900	-1.681983
H	-3.401620	0.248526	-2.295603
P	-2.243779	-0.170577	-0.079153
Mn	0.000035	-0.440256	0.088617
C	0.000197	-2.131010	-0.431774
O	0.000345	-3.286573	-0.717560
C	0.000232	-0.870041	1.789811
O	0.000371	-1.151213	2.942726
C	3.122920	1.147918	0.968596
C	4.572390	1.444407	0.548291
C	3.013638	0.900476	2.481920
H	2.500449	2.039307	0.731230
H	4.672723	1.657693	-0.535593
H	4.948527	2.335650	1.095932
H	5.255529	0.604406	0.795853
H	1.974430	0.679846	2.793840
H	3.658519	0.057363	2.808072
H	3.352938	1.801526	3.036824
C	3.425595	-1.655049	-0.026152
C	3.394322	-2.498741	-1.312690
C	3.143791	-2.532197	1.207691
H	4.443084	-1.215250	0.073187
H	3.684886	-1.920928	-2.213308
H	4.114981	-3.340219	-1.220942
H	2.392506	-2.940683	-1.486064
H	3.159883	-1.958255	2.155136
H	2.150784	-3.019333	1.127994
H	3.909699	-3.333887	1.286558
C	-3.122748	1.147975	0.968350
C	-3.011792	0.902309	2.481832
C	-4.572779	1.443168	0.549092
H	-2.501013	2.039433	0.729276
H	-1.972116	0.682657	2.792875
H	-3.351069	1.803765	3.036093
H	-3.655798	0.059149	2.809590
H	-4.674297	1.654836	-0.535003
H	-5.255249	0.603189	0.798556
H	-4.948802	2.335017	1.095826
C	-3.425314	-1.655777	-0.024483
C	-3.143158	-2.531882	1.210014
C	-3.394056	-2.500475	-1.310360
H	-4.442871	-1.216096	0.074643
H	-3.159369	-1.957205	2.157015

H	-3.908800	-3.333759	1.289559
H	-2.149989	-3.018750	1.130629
H	-3.684668	-1.923371	-2.211416
H	-2.392240	-2.942521	-1.483440
H	-4.114701	-3.341889	-1.217923
H	-0.000102	1.138389	0.577109
C	0.000099	3.047214	0.435551
H	-0.000831	1.449045	-1.606388
N	-0.001497	3.143897	-0.805749
O	0.001715	3.298345	1.603668
C	-0.002540	4.409596	-1.545481
H	-0.898291	4.459166	-2.198675
H	-0.001228	5.297762	-0.879169
H	0.890972	4.458818	-2.201751

4TSGH

Frequencies, energies and thermodynamic properties:

Lowest Vibrational Mode (1/cm) = -797.5927
2nd Lowest Vibrational Mode (1/cm) = 26.8831
E(RB-P86) (a.u.) = -2954.89696819
Thermal correction to Enthalpy (a.u.) = 0.609765
Thermal correction to Gibbs Free Energy (a.u.) = 0.503577
Total Entropy (cal/Kmol) = 223.491
E(RPBE1PBE) (a.u.) = -2954.51208206

Optimised cartesian coordinates (Angstrom):

P	-2.276992	-0.170372	0.115739
C	-2.453823	0.198793	1.946648
C	-1.220110	-0.349061	2.674114
H	-3.399454	-0.183820	2.382141
H	-2.473149	1.306384	2.036001
H	-1.198340	0.045840	3.721262
H	-1.276236	-1.460049	2.764075
N	-0.000275	0.050175	1.953258
C	1.219002	-0.350846	2.674072
C	2.453500	0.195379	1.946719
H	1.273588	-1.461926	2.763858
H	1.197768	0.043919	3.721281
H	2.474475	1.302921	2.036335
H	3.398570	-0.188722	2.382112
P	2.276148	-0.173117	0.115712
Mn	-0.000537	-0.305714	-0.126633
C	-0.001550	-2.039476	0.033368
O	-0.002241	-3.224912	0.143660
C	-0.000679	-0.432723	-1.890321
O	-0.000827	-0.556034	-3.069492
C	-3.185765	1.295874	-0.669467
C	-4.586867	1.561053	-0.092974
C	-3.213235	1.233293	-2.206088
H	-2.514533	2.133847	-0.379731
H	-4.584532	1.678692	1.009548

H	-4.999392	2.499700	-0.522998
H	-5.301740	0.749667	-0.348524
H	-2.214497	1.019554	-2.634858
H	-3.919749	0.459251	-2.573517
H	-3.558627	2.206510	-2.616918
C	-3.407854	-1.653193	-0.225798
C	-3.392888	-2.704695	0.898310
C	-3.080222	-2.303002	-1.583742
H	-4.432265	-1.219611	-0.277645
H	-3.714027	-2.293130	1.876090
H	-4.096175	-3.527056	0.643779
H	-2.387548	-3.156225	1.019599
H	-3.060030	-1.573934	-2.417030
H	-2.092971	-2.806244	-1.554451
H	-3.844325	-3.072702	-1.827023
C	3.186710	1.292312	-0.668960
C	3.213115	1.230883	-2.205638
C	4.588546	1.554982	-0.093097
H	2.516963	2.131080	-0.378127
H	2.213756	1.019037	-2.633885
H	3.559786	2.203862	-2.615959
H	3.918156	0.456001	-2.574123
H	4.587053	1.671645	1.009535
H	5.302123	0.742833	-0.349824
H	5.002120	2.493437	-0.522535
C	3.405243	-1.657152	-0.226327
C	3.076787	-2.306047	-1.584506
C	3.389089	-2.709027	0.897413
H	4.430163	-1.224778	-0.278056
H	3.057540	-1.576629	-2.417515
H	3.839896	-3.076638	-1.828081
H	2.088877	-2.808008	-1.555419
H	3.710718	-2.298163	1.875328
H	2.383242	-3.159450	1.018569
H	4.091441	-3.532095	0.642584
O	0.000863	1.864209	-0.211742
C	0.001683	2.455753	0.925835
H	0.000530	1.296510	1.825972
N	0.005451	3.812088	0.936868
C	0.008502	4.694375	-0.230558
H	-0.896174	5.339231	-0.255829
H	0.903039	5.353379	-0.238666
H	0.021694	4.060542	-1.136346
H	0.004650	4.253731	1.858725

4TSHI

Frequencies, energies and thermodynamic properties:

Lowest Vibrational Mode (1/cm) = -277.3147

2nd Lowest Vibrational Mode (1/cm) = 20.0443

E(RB-P86) (a.u.) = -2954.86944262

Thermal correction to Enthalpy (a.u.) = 0.612383
Thermal correction to Gibbs Free Energy (a.u.) = 0.505222
Total Entropy (cal/Kmol) = 225.541
E(RPBE1PBE) (a.u.) = -2954.49384224
Optimised cartesian coordinates (Angstrom):

P	-2.318265	-0.029127	0.172489
C	-2.480794	0.068737	2.042132
C	-1.301029	-0.658678	2.697224
H	-3.450375	-0.318058	2.416705
H	-2.435926	1.150973	2.290636
H	-1.277544	-0.436866	3.791742
H	-1.415162	-1.761156	2.599354
N	-0.041750	-0.254985	2.053563
C	1.170783	-0.799314	2.684929
C	2.422767	-0.218257	2.017197
H	1.154063	-1.907784	2.589980
H	1.184514	-0.574442	3.779030
H	2.515622	0.860174	2.267785
H	3.341179	-0.723879	2.379072
P	2.230448	-0.290962	0.148494
Mn	-0.046805	-0.263029	-0.141377
C	-0.147503	-1.987997	-0.146692
O	-0.220095	-3.177296	-0.168237
C	-0.057540	-0.256057	-1.902555
O	-0.071760	-0.294826	-3.082902
C	-3.167632	1.575540	-0.365368
C	-4.588889	1.760626	0.191492
C	-3.112852	1.794170	-1.885213
H	-2.494211	2.326747	0.101498
H	-4.640467	1.647192	1.293544
H	-4.953676	2.782314	-0.051206
H	-5.311325	1.047533	-0.259920
H	-2.079897	1.683371	-2.266528
H	-3.778913	1.092746	-2.430793
H	-3.457961	2.822628	-2.127544
C	-3.518048	-1.394651	-0.370183
C	-3.563839	-2.598976	0.587386
C	-3.222959	-1.850976	-1.811592
H	-4.518348	-0.906931	-0.352355
H	-3.865297	-2.318435	1.616620
H	-4.312936	-3.332922	0.218896
H	-2.587314	-3.121420	0.635462
H	-3.160117	-1.006347	-2.525343
H	-2.265204	-2.406758	-1.865611
H	-4.028150	-2.531490	-2.163485
C	3.258305	1.207877	-0.391522
C	3.114737	1.519595	-1.889549
C	4.730254	1.164442	0.052609
H	2.743715	2.017497	0.171887
H	2.049242	1.608925	-2.177193

H	3.613085	2.486090	-2.120148
H	3.597258	0.747225	-2.524987
H	4.849001	0.949822	1.134672
H	5.316064	0.408537	-0.512304
H	5.207394	2.149453	-0.141782
C	3.257650	-1.787346	-0.405784
C	2.912848	-2.187492	-1.852196
C	3.154857	-2.997194	0.540248
H	4.309464	-1.425837	-0.381267
H	2.968137	-1.335139	-2.557779
H	3.621652	-2.966203	-2.207726
H	1.889548	-2.609226	-1.915858
H	3.487021	-2.764490	1.572021
H	2.122735	-3.398518	0.583993
H	3.810722	-3.812583	0.165198
O	-0.053625	2.175420	-0.741314
C	0.184150	2.180948	0.514368
H	0.018418	0.801909	1.908856
N	0.664376	3.368652	1.048841
C	0.876236	4.608934	0.305002
H	0.235104	5.436501	0.680903
H	1.934980	4.947557	0.344840
H	0.607316	4.399028	-0.749285
H	0.820399	3.383685	2.059304

4TSID

Frequencies, energies and thermodynamic properties:

Lowest Vibrational Mode (1/cm) = -97.0777
2nd Lowest Vibrational Mode (1/cm) = 45.1534
E(RB-P86) (a.u.) = -2954.89306678
Thermal correction to Enthalpy (a.u.) = 0.610557
Thermal correction to Gibbs Free Energy (a.u.) = 0.507405
Total Entropy (cal/Kmol) = 217.102
E(RPBE1PBE) (a.u.) = -2954.50779274

Optimised cartesian coordinates (Angstrom):

P	-2.249862	-0.142108	0.120517
C	-2.453466	0.325873	1.921219
C	-1.183653	-0.106229	2.662009
H	-3.374660	-0.092718	2.375428
H	-2.547517	1.433137	1.947084
H	-1.204526	0.317636	3.700615
H	-1.197727	-1.222081	2.801361
N	0.005433	0.345116	1.946566
C	1.207915	-0.053568	2.673436
C	2.466374	0.440652	1.949525
H	1.272366	-1.167962	2.809269
H	1.198009	0.366932	3.713701
H	2.523178	1.549310	1.997783
H	3.398769	0.050050	2.405613
P	2.299444	-0.015231	0.140003

Mn	0.031141	-0.177684	-0.131338
C	0.077288	-1.915796	0.294935
O	0.109238	-3.073179	0.529485
C	0.045005	-0.571269	-1.854201
O	0.053346	-0.862276	-3.001934
C	-3.269350	1.189903	-0.765840
C	-4.697328	1.366887	-0.221467
C	-3.264381	1.029456	-2.295384
H	-2.690811	2.108007	-0.521997
H	-4.722350	1.540639	0.873387
H	-5.175742	2.245210	-0.706327
H	-5.340266	0.488784	-0.443515
H	-2.241761	0.893860	-2.698414
H	-3.883114	0.165672	-2.617726
H	-3.698819	1.935004	-2.770744
C	-3.289926	-1.711440	-0.145768
C	-3.307621	-2.667724	1.060069
C	-2.872263	-2.452164	-1.429993
H	-4.324919	-1.327154	-0.286388
H	-3.701075	-2.191301	1.980042
H	-3.968371	-3.532120	0.832025
H	-2.300140	-3.072956	1.280853
H	-2.828638	-1.788123	-2.315082
H	-1.874436	-2.921098	-1.316637
H	-3.600981	-3.262006	-1.650063
C	3.237610	1.387237	-0.729771
C	3.250121	1.238360	-2.260638
C	4.648132	1.652979	-0.176466
H	2.599429	2.265583	-0.484245
H	2.241787	1.032924	-2.670864
H	3.622684	2.175004	-2.728011
H	3.928825	0.421863	-2.585123
H	4.656281	1.817048	0.920134
H	5.346808	0.819829	-0.402949
H	5.071398	2.564644	-0.650840
C	3.435060	-1.515060	-0.136664
C	3.069868	-2.264597	-1.431675
C	3.498735	-2.483177	1.058112
H	4.446543	-1.068589	-0.264519
H	2.989483	-1.594818	-2.309743
H	3.847883	-3.025731	-1.656593
H	2.102213	-2.795147	-1.328655
H	3.860487	-1.997707	1.986257
H	2.513556	-2.945336	1.267291
H	4.206993	-3.307247	0.823786
O	-0.153894	2.487284	-1.542576
C	-0.035836	1.722886	-0.639211
H	0.021994	1.956644	1.649406
N	0.135160	2.848052	1.016848
C	-0.822487	3.938759	1.063630

H	-0.551659	4.728749	1.799451
H	-0.917855	4.422083	0.063104
H	-1.824723	3.552978	1.343210
H	1.106713	3.184078	1.015451

4TSJaD

Frequencies, energies and thermodynamic properties:

Lowest Vibrational Mode (1/cm) = -812.0502

2nd Lowest Vibrational Mode (1/cm) = 37.5654

E(RB-P86) (a.u.) = -2860.27245697

Thermal correction to Enthalpy (a.u.) = 0.558335

Thermal correction to Gibbs Free Energy (a.u.) = 0.460695

Total Entropy (cal/Kmol) = 205.501

E(RPBE1PBE) (a.u.) = -2859.88711495

Optimised cartesian coordinates (Angstrom):

P	-2.284120	0.007549	0.241835
C	-2.455531	-0.690104	1.967188
C	-1.215257	-1.531951	2.280470
H	-3.395053	-1.260511	2.111282
H	-2.487915	0.188764	2.645487
H	-1.184746	-1.755254	3.375152
H	-1.268555	-2.522745	1.768155
N	0.000000	-0.798347	1.886943
C	1.215256	-1.531954	2.280471
C	2.455533	-0.690110	1.967189
H	1.268553	-2.522748	1.768156
H	1.184744	-1.755256	3.375153
H	2.487926	0.188755	2.645492
H	3.395052	-1.260523	2.111279
P	2.284122	0.007547	0.241836
Mn	0.000002	0.059723	-0.031233
C	-0.000001	-1.585277	-0.749413
O	-0.000006	-2.654460	-1.241733
C	0.000006	0.815398	-1.632111
O	0.000011	1.304715	-2.706614
C	-3.220616	1.647472	0.382532
C	-4.649394	1.510237	0.936413
C	-3.194964	2.473671	-0.914345
H	-2.605280	2.180674	1.141311
H	-4.687720	0.958764	1.897367
H	-5.074625	2.520378	1.119260
H	-5.327773	1.000939	0.219550
H	-2.173153	2.579393	-1.328573
H	-3.841398	2.026056	-1.698150
H	-3.585021	3.494672	-0.715809
C	-3.382245	-1.046292	-0.889791
C	-3.417424	-2.537039	-0.509200
C	-3.004546	-0.857889	-2.370828
H	-4.403447	-0.632517	-0.733555
H	-3.784632	-2.707445	0.522413

H -4.109666 -3.072851 -1.193819
H -2.422705 -3.015140 -0.609827
H -2.956144 0.207407 -2.669911
H -2.019125 -1.312734 -2.596795
H -3.759302 -1.355790 -3.016572
C 3.220617 1.647471 0.382539
C 3.194975 2.473668 -0.914340
C 4.649390 1.510240 0.936432
H 2.605273 2.180674 1.141311
H 2.173168 2.579386 -1.328579
H 3.585027 3.494671 -0.715801
H 3.841420 2.026055 -1.698138
H 4.687707 0.958777 1.897392
H 5.327775 1.000935 0.219580
H 5.074621 2.520382 1.119273
C 3.382247 -1.046292 -0.889791
C 3.004555 -0.857879 -2.370828
C 3.417418 -2.537041 -0.509208
H 4.403451 -0.632523 -0.733549
H 2.956161 0.207418 -2.669908
H 3.759309 -1.355783 -3.016572
H 2.019131 -1.312716 -2.596801
H 3.784621 -2.707454 0.522405
H 2.422697 -3.015137 -0.609841
H 4.109659 -3.072853 -1.193828
O -0.000018 2.998298 0.697345
C -0.000006 1.822025 0.712724
H -0.000090 1.338353 2.770032
H -0.000009 0.367876 2.504535

CO

Frequencies, energies and thermodynamic properties:
Lowest Vibrational Mode (1/cm) = 2149.2038
2nd Lowest Vibrational Mode (1/cm) =
E(RB-P86) (a.u.) = -113.225123535
Thermal correction to Enthalpy (a.u.) = 0.008201
Thermal correction to Gibbs Free Energy (a.u.) = -0.014249
Total Entropy (cal/Kmol) = 47.251
E(RPBE1PBE) (a.u.) = -113.231031939

Optimised cartesian coordinates (Angstrom):
C 0.000000 0.000000 -0.652672
O 0.000000 0.000000 0.489504

H2

Frequencies, energies and thermodynamic properties:
Lowest Vibrational Mode (1/cm) = 4271.1430
2nd Lowest Vibrational Mode (1/cm) =
E(RB-P86) (a.u.) = -1.17253812887
Thermal correction to Enthalpy (a.u.) = 0.013035
Thermal correction to Gibbs Free Energy (a.u.) = -0.001819

Total Entropy (cal/Kmol) = 31.263
E(RPBE1PBE) (a.u.) = -1.16811951249
Optimised cartesian coordinates (Angstrom):
H 0.000000 0.000000 0.383856
H 0.000000 0.000000 -0.383856

HCHO
Frequencies, energies and thermodynamic properties:
Lowest Vibrational Mode (1/cm) = 1153.3722
2nd Lowest Vibrational Mode (1/cm) = 1224.0562
E(RB-P86) (a.u.) = -114.419675639
Thermal correction to Enthalpy (a.u.) = 0.029499
Thermal correction to Gibbs Free Energy (a.u.) = 0.003985
Total Entropy (cal/Kmol) = 53.698
E(RPBE1PBE) (a.u.) = -114.422572597
Optimised cartesian coordinates (Angstrom):
C -0.530702 -0.000023 -0.000046
O 0.682771 -0.000073 0.000012
H -1.138378 0.952762 0.000092
H -1.139578 -0.952041 0.000092

MeNCO
Frequencies, energies and thermodynamic properties:
Lowest Vibrational Mode (1/cm) = 49.4982
2nd Lowest Vibrational Mode (1/cm) = 151.3578
E(RB-P86) (a.u.) = -207.846761028
Thermal correction to Enthalpy (a.u.) = 0.054656
Thermal correction to Gibbs Free Energy (a.u.) = 0.021495
Total Entropy (cal/Kmol) = 69.792
E(RPBE1PBE) (a.u.) = -207.841159117
Optimised cartesian coordinates (Angstrom):
C -0.752697 -0.036634 0.000026
N 0.417817 -0.334176 -0.000007
O -1.934663 0.119256 -0.000011
C 1.772274 0.137721 0.000003
H 2.468367 -0.724077 -0.001415
H 1.982982 0.752513 -0.900632
H 1.983767 0.750229 0.902006

MeNH2
Frequencies, energies and thermodynamic properties:
Lowest Vibrational Mode (1/cm) = 322.3283
2nd Lowest Vibrational Mode (1/cm) = 840.0892
E(RB-P86) (a.u.) = -95.7846538999
Thermal correction to Enthalpy (a.u.) = 0.066188
Thermal correction to Gibbs Free Energy (a.u.) = 0.038887
Total Entropy (cal/Kmol) = 57.461
E(RPBE1PBE) (a.u.) = -95.7815728994
Optimised cartesian coordinates (Angstrom):
C -0.703538 0.000000 0.017638

H -1.133970 0.891252 -0.488068
H -1.133970 -0.891252 -0.488069
H -1.093240 0.000000 1.068052
N 0.756044 0.000000 -0.132793
H 1.145050 0.813606 0.365907
H 1.145050 -0.813606 0.365907

MeNHCH2OH+MeOH
Frequencies, energies and thermodynamic properties:
Lowest Vibrational Mode (1/cm) = 74.0705
2nd Lowest Vibrational Mode (1/cm) = 81.1230
E(RB-P86) (a.u.) = -325.885317230
Thermal correction to Enthalpy (a.u.) = 0.157039
Thermal correction to Gibbs Free Energy (a.u.) = 0.113142
Total Entropy (cal/Kmol) = 92.390
E(RPBE1PBE) (a.u.) = -325.892661517
Optimised cartesian coordinates (Angstrom):
C 0.963002 0.926872 0.604079
O 0.478771 1.512099 -0.575329
H 0.367409 1.204123 1.510455
H 2.001814 1.312597 0.752009
N 0.928530 -0.547115 0.521915
C 1.956598 -1.117757 -0.358034
H 1.820893 -2.214330 -0.442683
H 1.841858 -0.674764 -1.366953
H 2.997503 -0.913420 -0.009276
H 0.999489 -0.946694 1.468110
H -0.411976 1.087214 -0.729310
O -1.533435 -0.323489 -0.622794
H -0.704511 -0.672105 -0.150226
C -2.614193 -0.305350 0.297305
H -2.934214 -1.332130 0.595881
H -2.389217 0.263211 1.232041
H -3.483895 0.184629 -0.188563

MeNHCH2OH
Frequencies, energies and thermodynamic properties:
Lowest Vibrational Mode (1/cm) = 118.8537
2nd Lowest Vibrational Mode (1/cm) = 168.8564
E(RB-P86) (a.u.) = -210.229185874
Thermal correction to Enthalpy (a.u.) = 0.100331
Thermal correction to Gibbs Free Energy (a.u.) = 0.065596
Total Entropy (cal/Kmol) = 73.105
E(RPBE1PBE) (a.u.) = -210.235200492
Optimised cartesian coordinates (Angstrom):
C 0.706821 0.542989 0.280442
O 1.389295 -0.617258 -0.229862
H 1.260508 1.473813 0.003700
H 2.208511 -0.729580 0.291108
H 0.650750 0.498730 1.396469

N	-0.624199	0.650397	-0.232993
H	-0.597480	0.786390	-1.252087
C	-1.525278	-0.448754	0.112894
H	-2.529235	-0.258528	-0.319052
H	-1.184163	-1.455541	-0.227995
H	-1.643122	-0.495401	1.217683

MeNHCH2O-

Frequencies, energies and thermodynamic properties:

Lowest Vibrational Mode (1/cm) = 166.3582
2nd Lowest Vibrational Mode (1/cm) = 258.9750
E(RB-P86) (a.u.) = -209.694335808
Thermal correction to Enthalpy (a.u.) = 0.084982
Thermal correction to Gibbs Free Energy (a.u.) = 0.052101
Total Entropy (cal/Kmol) = 69.205
E(RPBE1PBE) (a.u.) = -209.706239048

Optimised cartesian coordinates (Angstrom):

C	-0.853016	-0.409169	0.261972
O	-1.418658	0.678016	-0.187026
H	-1.371973	-1.419422	0.003424
H	-0.708336	-0.497466	1.418687
N	0.589079	-0.713995	-0.212900
H	0.498926	-0.713417	-1.245560
C	1.464967	0.415122	0.101505
H	2.461000	0.305309	-0.383558
H	1.032328	1.407809	-0.190282
H	1.642064	0.455305	1.202936

MeNHCONHMe

Frequencies, energies and thermodynamic properties:

Lowest Vibrational Mode (1/cm) = 72.3214
2nd Lowest Vibrational Mode (1/cm) = 95.5320
E(RB-P86) (a.u.) = -303.671874431
Thermal correction to Enthalpy (a.u.) = 0.124212
Thermal correction to Gibbs Free Energy (a.u.) = 0.082944
Total Entropy (cal/Kmol) = 86.856
E(RPBE1PBE) (a.u.) = -303.669901376

Optimised cartesian coordinates (Angstrom):

C	-0.037944	0.537193	-0.010250
O	-0.606714	1.641100	0.030924
N	1.340965	0.435723	-0.078711
N	-0.720157	-0.663979	-0.000657
H	1.814186	1.332736	0.038311
H	-0.201391	-1.539846	-0.073202
C	-2.168474	-0.724524	0.000519
C	2.122788	-0.781611	0.036371
H	-2.543056	0.313266	0.086319
H	-2.571720	-1.169304	-0.936483
H	-2.554933	-1.315727	0.859050
H	3.196496	-0.530643	-0.055024

H 1.980085 -1.306715 1.010352
H 1.890177 -1.501127 -0.780979

MeNHCOOMe
Frequencies, energies and thermodynamic properties:
Lowest Vibrational Mode (1/cm) = 78.2357
2nd Lowest Vibrational Mode (1/cm) = 87.7482
E(RB-P86) (a.u.) = -323.522648640
Thermal correction to Enthalpy (a.u.) = 0.112269
Thermal correction to Gibbs Free Energy (a.u.) = 0.071934
Total Entropy (cal/Kmol) = 84.891
E(RPBE1PBE) (a.u.) = -323.528826568
Optimised cartesian coordinates (Angstrom):
N -1.134193 -0.616889 0.000016
H -1.041470 -1.634573 -0.000006
C 0.016645 0.117456 0.000006
O 0.095110 1.343600 0.000007
O 1.102947 -0.719916 0.000004
C 2.377741 -0.066063 -0.000010
H 2.505692 0.569965 0.900420
H 3.133693 -0.872968 0.000191
H 2.505837 0.569637 -0.900653
C -2.452934 -0.014860 -0.000013
H -2.324442 1.083945 0.000034
H -3.036524 -0.303188 -0.900934
H -3.036590 -0.303259 0.900842

MeNH-
Frequencies, energies and thermodynamic properties:
Lowest Vibrational Mode (1/cm) = 420.0845
2nd Lowest Vibrational Mode (1/cm) = 1010.0291
E(RB-P86) (a.u.) = -95.1978002556
Thermal correction to Enthalpy (a.u.) = 0.048338
Thermal correction to Gibbs Free Energy (a.u.) = 0.021562
Total Entropy (cal/Kmol) = 56.355
E(RPBE1PBE) (a.u.) = -95.2059626340
Optimised cartesian coordinates (Angstrom):
C 0.587792 -0.010727 -0.000005
H 1.107760 0.993559 -0.000100
H 1.132062 -0.547778 -0.892177
H 1.132111 -0.547612 0.892275
N -0.815455 0.142895 0.000005
H -1.190495 -0.834069 0.000000

MeOH
Frequencies, energies and thermodynamic properties:
Lowest Vibrational Mode (1/cm) = 328.9952
2nd Lowest Vibrational Mode (1/cm) = 1029.4984
E(RB-P86) (a.u.) = -115.633780797
Thermal correction to Enthalpy (a.u.) = 0.053745

Thermal correction to Gibbs Free Energy (a.u.) = 0.026709
Total Entropy (cal/Kmol) = 56.901
E(RPBE1PBE) (a.u.) = -115.641714677
Optimised cartesian coordinates (Angstrom):

C	0.658881	-0.019023	0.000000
H	1.099499	0.999699	-0.000001
H	1.050517	-0.550407	-0.901771
H	1.050518	-0.550404	0.901773
O	-0.752661	0.123802	0.000000
H	-1.132532	-0.775166	0.000000

NMF
Frequencies, energies and thermodynamic properties:
Lowest Vibrational Mode (1/cm) = 102.9880
2nd Lowest Vibrational Mode (1/cm) = 283.0339
E(RB-P86) (a.u.) = -209.059955320
Thermal correction to Enthalpy (a.u.) = 0.077762
Thermal correction to Gibbs Free Energy (a.u.) = 0.044705
Total Entropy (cal/Kmol) = 69.575
E(RPBE1PBE) (a.u.) = -209.058638253
Optimised cartesian coordinates (Angstrom):

H	-1.432450	1.408279	-0.000054
C	-0.866120	0.434389	-0.000007
H	0.811277	1.607892	0.000071
N	0.480924	0.639717	0.000016
O	-1.427290	-0.662281	0.000005
C	1.458602	-0.436119	-0.000016
H	2.107361	-0.395772	-0.900612
H	2.106818	-0.396395	0.901019
H	0.903948	-1.393400	-0.000442

NPF
Frequencies, energies and thermodynamic properties:
Lowest Vibrational Mode (1/cm) = 68.4318
2nd Lowest Vibrational Mode (1/cm) = 175.1274
E(RB-P86) (a.u.) = -400.667732728
Thermal correction to Enthalpy (a.u.) = 0.132527
Thermal correction to Gibbs Free Energy (a.u.) = 0.091849
Total Entropy (cal/Kmol) = 85.615
E(RPBE1PBE) (a.u.) = -400.647469526
Optimised cartesian coordinates (Angstrom):

N	1.419013	-0.920476	0.000106
H	1.490224	-1.942710	0.000135
C	2.621852	-0.253377	0.000042
H	3.478884	-0.982425	-0.000079
C	0.106002	-0.400638	0.000057
C	-0.168482	0.988649	0.000113
C	-0.969663	-1.321641	-0.000042
C	-1.502416	1.430076	0.000038
H	0.669018	1.697099	0.000175

C -2.295274 -0.863572 -0.000066
H -0.759662 -2.404044 -0.000084
C -2.571633 0.516383 -0.000038
H -1.704568 2.513292 0.000066
H -3.119155 -1.594868 -0.000143
H -3.612562 0.875447 -0.000081
O 2.787803 0.963283 -0.000169

PhNCO
Frequencies, energies and thermodynamic properties:
Lowest Vibrational Mode (1/cm) = 67.6457
2nd Lowest Vibrational Mode (1/cm) = 85.3832
E(RB-P86) (a.u.) = -399.455919766
Thermal correction to Enthalpy (a.u.) = 0.109166
Thermal correction to Gibbs Free Energy (a.u.) = 0.068457
Total Entropy (cal/Kmol) = 85.680
E(RPBE1PBE) (a.u.) = -399.431883115
Optimised cartesian coordinates (Angstrom):
N 1.151747 -1.035099 0.000000
C 2.366144 -1.024919 0.000000
C 0.000000 -0.247449 0.000000
C 0.070591 1.166551 0.000000
C -1.259337 -0.888936 0.000000
C -1.112407 1.921749 0.000000
H 1.052602 1.664205 0.000000
C -2.434464 -0.120325 0.000000
H -1.298715 -1.988466 0.000000
C -2.367903 1.284800 0.000000
H -1.050501 3.021556 0.000000
H -3.412451 -0.627068 0.000000
H -3.292074 1.883406 0.000000
O 3.545395 -1.157096 0.000000

PhNH2
Frequencies, energies and thermodynamic properties:
Lowest Vibrational Mode (1/cm) = 213.6125
2nd Lowest Vibrational Mode (1/cm) = 359.4426
E(RB-P86) (a.u.) = -287.403537874
Thermal correction to Enthalpy (a.u.) = 0.120518
Thermal correction to Gibbs Free Energy (a.u.) = 0.084411
Total Entropy (cal/Kmol) = 75.995
E(RPBE1PBE) (a.u.) = -287.380493117
Optimised cartesian coordinates (Angstrom):
N -2.342554 0.000080 -0.070239
H -2.815577 0.851618 0.239874
H -2.815647 -0.851880 0.238696
C -0.955411 -0.000113 -0.006876
C -0.223398 -1.218535 -0.001960
C -0.223421 1.218467 -0.002098
C 1.179831 -1.211315 0.002680

H	-0.770412	-2.176033	-0.002939
C	1.179707	1.211418	0.002820
H	-0.770620	2.175863	-0.003618
C	1.897842	0.000026	0.004410
H	1.720255	-2.172282	0.007983
H	1.720135	2.172379	0.008244
H	2.998843	0.000082	0.009581

PhNHCH2OH+MeOH

Frequencies, energies and thermodynamic properties:

Lowest Vibrational Mode (1/cm) = 41.1862
2nd Lowest Vibrational Mode (1/cm) = 53.0327
E(RB-P86) (a.u.) = -517.495711960
Thermal correction to Enthalpy (a.u.) = 0.211780
Thermal correction to Gibbs Free Energy (a.u.) = 0.159452
Total Entropy (cal/Kmol) = 110.134
E(RPBE1PBE) (a.u.) = -517.485725384

Optimised cartesian coordinates (Angstrom):

C	1.250324	-1.189814	1.147136
O	1.744164	-1.786474	-0.021234
H	2.053252	-0.962390	1.890492
H	0.550114	-1.923371	1.610168
N	0.567519	0.097640	0.896551
H	0.698463	0.756421	1.672914
H	2.272715	-1.081325	-0.492161
O	2.596191	0.585050	-1.018668
H	1.814272	0.752332	-0.422287
C	3.715593	1.310734	-0.518745
H	4.600854	1.036897	-1.127667
H	3.571449	2.412377	-0.605752
H	3.948467	1.075672	0.546391
C	-0.761937	0.107583	0.409741
C	-1.235912	-0.918593	-0.444655
C	-1.631022	1.173900	0.747404
C	-2.552781	-0.874098	-0.932608
H	-0.551070	-1.728405	-0.735950
C	-2.941250	1.210253	0.245680
H	-1.272488	1.976855	1.413024
C	-3.414734	0.185351	-0.595394
H	-2.905428	-1.681355	-1.595047
H	-3.601667	2.047713	0.523237
H	-4.444095	0.214607	-0.985370

PhNHCH2OH

Frequencies, energies and thermodynamic properties:

Lowest Vibrational Mode (1/cm) = 49.4478
2nd Lowest Vibrational Mode (1/cm) = 106.3868
E(RB-P86) (a.u.) = -401.846830376
Thermal correction to Enthalpy (a.u.) = 0.155135
Thermal correction to Gibbs Free Energy (a.u.) = 0.112072

Total Entropy (cal/Kmol) = 90.635
E(RPBE1PBE) (a.u.) = -401.832686722
Optimised cartesian coordinates (Angstrom):

C	-2.452066	-0.186692	0.470127
O	-2.907385	0.787160	-0.460722
H	-3.245728	-0.929440	0.717581
H	-2.221441	0.393994	1.392246
N	-1.301212	-0.937389	0.034068
H	-1.400155	-1.951665	-0.022429
H	-3.075276	0.317325	-1.302811
C	-0.004936	-0.433642	0.015324
C	0.251094	0.961990	0.103711
C	1.102680	-1.316666	-0.102762
C	1.571693	1.440295	0.098952
H	-0.593738	1.665136	0.134348
C	2.414442	-0.821402	-0.116517
H	0.921638	-2.402219	-0.175689
C	2.664817	0.560804	-0.009049
H	1.745031	2.526877	0.168435
H	3.254574	-1.529524	-0.205900
H	3.696323	0.945841	-0.017195

PhNHCH2O-

Frequencies, energies and thermodynamic properties:
Lowest Vibrational Mode (1/cm) = 82.4338
2nd Lowest Vibrational Mode (1/cm) = 119.6576
E(RB-P86) (a.u.) = -401.318632051
Thermal correction to Enthalpy (a.u.) = 0.139328
Thermal correction to Gibbs Free Energy (a.u.) = 0.097420
Total Entropy (cal/Kmol) = 88.203
E(RPBE1PBE) (a.u.) = -401.306932356
Optimised cartesian coordinates (Angstrom):

C	-2.568877	-0.153273	0.357634
O	-2.878027	0.887440	-0.352020
H	-3.346503	-1.011876	0.423000
H	-2.290045	0.034498	1.471283
N	-1.329521	-0.981852	-0.140020
H	-1.395151	-1.995025	0.013167
C	-0.056158	-0.478137	-0.067214
C	0.168457	0.936669	0.003179
C	1.098365	-1.324146	-0.074344
C	1.470796	1.450607	0.061120
H	-0.728770	1.577561	-0.031637
C	2.393433	-0.787891	-0.019631
H	0.957728	-2.418416	-0.121459
C	2.601495	0.605140	0.049889
H	1.609685	2.544971	0.112290
H	3.258248	-1.473921	-0.026085
H	3.620612	1.021852	0.091935

PhNHCONHPh
Frequencies, energies and thermodynamic properties:
Lowest Vibrational Mode (1/cm) = 20.6971
2nd Lowest Vibrational Mode (1/cm) = 25.5311
E(RB-P86) (a.u.) = -686.891089284
Thermal correction to Enthalpy (a.u.) = 0.233655
Thermal correction to Gibbs Free Energy (a.u.) = 0.176963
Total Entropy (cal/Kmol) = 119.317
E(RPBE1PBE) (a.u.) = -686.850097799
Optimised cartesian coordinates (Angstrom):

C	0.000000	0.000000	0.031408
O	0.000000	0.000000	1.264907
N	-0.015496	-1.157602	-0.746272
N	0.015496	1.157602	-0.746272
H	-0.041231	-1.044792	-1.762682
H	0.041231	1.044792	-1.762682
C	0.010669	2.499278	-0.317132
C	-0.022223	2.892640	1.043705
C	0.038429	3.501390	-1.320614
C	-0.027397	4.259087	1.372155
H	-0.042021	2.120515	1.821643
C	0.033014	4.859968	-0.974503
H	0.064104	3.206108	-2.382998
C	0.000000	5.251772	0.376797
H	-0.053331	4.547748	2.435493
H	0.054762	5.618803	-1.772995
H	-0.004342	6.318618	0.649148
C	-0.010669	-2.499278	-0.317132
C	0.022223	-2.892640	1.043705
C	-0.038429	-3.501390	-1.320614
C	0.027397	-4.259087	1.372155
H	0.042021	-2.120515	1.821643
C	-0.033014	-4.859968	-0.974503
H	-0.064104	-3.206108	-2.382998
C	0.000000	-5.251772	0.376797
H	0.053331	-4.547748	2.435493
H	-0.054762	-5.618803	-1.772995
H	0.004342	-6.318618	0.649148

PhNHCOOMe
Frequencies, energies and thermodynamic properties:
Lowest Vibrational Mode (1/cm) = 46.2135
2nd Lowest Vibrational Mode (1/cm) = 61.7372
E(RB-P86) (a.u.) = -515.131621344
Thermal correction to Enthalpy (a.u.) = 0.166892
Thermal correction to Gibbs Free Energy (a.u.) = 0.118782
Total Entropy (cal/Kmol) = 101.256
E(RPBE1PBE) (a.u.) = -515.118858719
Optimised cartesian coordinates (Angstrom):

N	0.601671	-0.702469	0.000105

H 0.787622 -1.708971 0.000069
C 1.725771 0.097124 0.000319
C -0.751259 -0.313254 -0.000121
C -1.169557 1.039450 -0.000065
C -1.729092 -1.338863 -0.000410
C -2.541889 1.341610 -0.000296
H -0.412459 1.833557 0.000157
C -3.094546 -1.019037 -0.000636
H -1.409943 -2.394238 -0.000458
C -3.513078 0.324598 -0.000582
H -2.852809 2.398899 -0.000249
H -3.837844 -1.832264 -0.000859
H -4.585289 0.575027 -0.000760
O 1.761933 1.321416 0.000383
O 2.830403 -0.705560 0.000422
C 4.090518 -0.017501 0.000614
H 4.196942 0.619857 0.902088
H 4.864816 -0.805773 0.000907
H 4.197362 0.619581 -0.901007

PhNH-
Frequencies, energies and thermodynamic properties:
Lowest Vibrational Mode (1/cm) = 159.0034
2nd Lowest Vibrational Mode (1/cm) = 398.2737
E(RB-P86) (a.u.) = -286.869277693
Thermal correction to Enthalpy (a.u.) = 0.106147
Thermal correction to Gibbs Free Energy (a.u.) = 0.070449
Total Entropy (cal/Kmol) = 75.133
E(RPBE1PBE) (a.u.) = -286.849844348
Optimised cartesian coordinates (Angstrom):
N 2.410246 -0.136859 -0.000212
H 2.784399 0.833046 -0.000161
C 1.074327 -0.034701 0.000027
C 0.242598 -1.234134 0.000220
C 0.300841 1.204411 0.000132
C -1.153298 -1.191104 -0.000080
H 0.772290 -2.203856 0.000468
C -1.098869 1.227034 0.000057
H 0.861409 2.159175 0.000232
C -1.864228 0.036328 -0.000159
H -1.715706 -2.143956 -0.000013
H -1.616400 2.205276 0.000080
H -2.965939 0.061325 -0.000298
----
tBuOH
Frequencies, energies and thermodynamic properties:
Lowest Vibrational Mode (1/cm) = 206.1782
2nd Lowest Vibrational Mode (1/cm) = 257.3741
E(RB-P86) (a.u.) = -233.497745962
Thermal correction to Enthalpy (a.u.) = 0.138606

Thermal correction to Gibbs Free Energy (a.u.) = 0.101748
Total Entropy (cal/Kmol) = 77.574
E(RPBE1PBE) (a.u.) = -233.497077979
Optimised cartesian coordinates (Angstrom):

C	0.001185	0.018044	0.000000
C	1.531459	0.143230	0.000000
H	1.872843	0.698285	0.898004
H	2.013198	-0.855293	0.000000
H	1.872843	0.698285	-0.898004
C	-0.488700	-0.707958	-1.268435
H	-0.094984	-1.744344	-1.326587
H	-1.598589	-0.769409	-1.281368
H	-0.164410	-0.158662	-2.176243
C	-0.488700	-0.707958	1.268435
H	-0.164410	-0.158662	2.176243
H	-1.598589	-0.769409	1.281368
H	-0.094984	-1.744344	1.326587
O	-0.488700	1.376148	0.000000
H	-1.464774	1.322219	0.000000

----
tBuO-
Frequencies, energies and thermodynamic properties:
Lowest Vibrational Mode (1/cm) = 197.4656
2nd Lowest Vibrational Mode (1/cm) = 265.3926
E(RB-P86) (a.u.) = -232.952483085
Thermal correction to Enthalpy (a.u.) = 0.123014
Thermal correction to Gibbs Free Energy (a.u.) = 0.086532
Total Entropy (cal/Kmol) = 76.783
E(RPBE1PBE) (a.u.) = -232.960271220
Optimised cartesian coordinates (Angstrom):

C	-0.000082	0.002245	0.161376
C	0.009972	-1.467548	-0.423231
H	-0.881008	-2.013560	-0.038300
H	0.010999	-1.542326	-1.538339
H	0.907743	-2.001619	-0.037335
C	1.263199	0.732034	-0.448594
H	1.326023	0.747021	-1.564460
H	1.277008	1.783878	-0.083419
H	2.181106	0.241009	-0.052929
C	-1.272433	0.715207	-0.449471
H	-2.183894	0.212834	-0.053250
H	-1.299805	1.767096	-0.085269
H	-1.335382	0.728302	-1.565357
O	-0.000841	0.023217	1.497273

TS-MeNHCH2OH+MeOH
Frequencies, energies and thermodynamic properties:
Lowest Vibrational Mode (1/cm) = -642.4449
2nd Lowest Vibrational Mode (1/cm) = 50.1491
E(RB-P86) (a.u.) = -325.866297348

Thermal correction to Enthalpy (a.u.) = 0.151178
Thermal correction to Gibbs Free Energy (a.u.) = 0.108686
Total Entropy (cal/Kmol) = 89.434
E(RPBE1PBE) (a.u.) = -325.868821294
Optimised cartesian coordinates (Angstrom):

C	-0.795169	-1.100426	0.488936
O	-0.148053	-1.383164	-0.651450
H	-0.253011	-1.328258	1.451063
H	-1.820797	-1.545989	0.561114
N	-0.998404	0.459016	0.589960
C	-2.007459	0.980649	-0.358776
H	-1.944822	2.083625	-0.418942
H	-1.782438	0.540612	-1.348274
H	-3.026382	0.680931	-0.044251
H	-1.194856	0.755061	1.559680
H	0.738842	-0.517743	-0.672247
O	1.319195	0.528461	-0.484462
H	0.033787	0.779657	0.276738
C	2.510260	0.338183	0.241395
H	3.296549	-0.169668	-0.371004
H	2.942469	1.315351	0.564697
H	2.384561	-0.279495	1.169676

TS-PhNHCH2OH+MeOH
Frequencies, energies and thermodynamic properties:
Lowest Vibrational Mode (1/cm) = -745.8771
2nd Lowest Vibrational Mode (1/cm) = 30.1338
E(RB-P86) (a.u.) = -517.468169358
Thermal correction to Enthalpy (a.u.) = 0.204906
Thermal correction to Gibbs Free Energy (a.u.) = 0.153730
Total Entropy (cal/Kmol) = 107.708
E(RPBE1PBE) (a.u.) = -517.451934535
Optimised cartesian coordinates (Angstrom):

C	1.268623	1.409980	0.904452
O	1.755993	0.376221	1.582316
H	2.010128	2.145627	0.480363
H	0.456077	1.978544	1.422910
N	0.572993	0.882051	-0.473168
H	0.578812	1.638827	-1.174520
H	2.256441	-0.346644	0.693964
O	2.382477	-0.885279	-0.366732
H	1.349417	0.078728	-0.727920
C	3.680948	-0.765829	-0.909061
H	4.415907	-1.395699	-0.352637
H	3.696655	-1.109396	-1.969234
H	4.076220	0.281715	-0.897045
C	-0.752761	0.329166	-0.309816
C	-0.893948	-0.932519	0.302391
C	-1.887078	1.059391	-0.713901
C	-2.181117	-1.456217	0.510365

H	0.005360	-1.491862	0.595817
C	-3.168978	0.520872	-0.509771
H	-1.767937	2.046083	-1.190308
C	-3.319552	-0.735001	0.106269
H	-2.292641	-2.442808	0.987100
H	-4.054707	1.089820	-0.833312
H	-4.325272	-1.153885	0.266756

1TSGH_Ph

Frequencies, energies and thermodynamic properties:

Lowest Vibrational Mode (1/cm) =	-586.2774
2nd Lowest Vibrational Mode (1/cm) =	8.6701
E(RB-P86) (a.u.) =	-2429.98542021
Thermal correction to Enthalpy (a.u.) =	0.652379
Thermal correction to Gibbs Free Energy (a.u.) =	0.529635
Total Entropy (cal/Kmol) =	258.335
E(RPBE1PBE) (a.u.) =	-2429.71227314

Optimised cartesian coordinates (Angstrom):

Ru	0.573030	0.096198	0.005567
P	0.391006	2.399842	0.321834
P	1.167937	-2.138032	0.317704
N	0.451657	0.073767	2.187017
O	0.642715	0.111271	-3.013471
C	1.927009	3.398650	0.044697
C	1.116951	-1.054417	2.871751
H	0.755402	-1.128168	3.926967
H	2.215945	-0.874628	2.926984
C	2.552923	4.160849	1.054410
H	2.131689	4.201436	2.070464
C	0.281820	-3.556938	-0.484254
C	-0.922060	3.444891	-0.467974
C	3.765490	-3.150964	1.052059
H	3.355785	-3.385291	2.046406
C	-0.825564	4.853373	-0.535468
H	0.064877	5.366365	-0.138079
C	-1.018422	-3.332569	-0.990885
H	-1.460587	-2.326112	-0.913269
C	0.822484	-2.368717	2.140538
H	-0.262108	-2.602790	2.195849
H	1.363832	-3.229579	2.582642
C	0.005169	2.503781	2.148165
H	0.242226	3.494033	2.587400
H	-1.096272	2.373763	2.212816
C	2.952033	-2.567274	0.056830
C	0.715218	1.355573	2.873871
H	1.812994	1.543276	2.925569
H	0.353169	1.304826	3.930258
C	-1.739792	-4.387196	-1.578882
H	-2.753109	-4.202735	-1.969867
C	0.851896	-4.846317	-0.583725

H	1.869050	-5.032179	-0.203520
C	3.670769	4.100488	-1.524599
H	4.104357	4.072102	-2.536923
C	2.503105	3.371910	-1.247684
H	2.036815	2.767805	-2.042510
C	0.606134	0.103792	-1.829678
C	-2.064194	2.801453	-0.995537
H	-2.136718	1.703283	-0.939920
C	3.731121	4.881543	0.777822
H	4.210489	5.467312	1.578349
C	5.118281	-3.441387	0.788741
H	5.741319	-3.891299	1.578144
C	0.127940	-5.896272	-1.175614
H	0.581706	-6.897503	-1.251053
C	3.519099	-2.280261	-1.207480
H	2.902463	-1.807406	-1.988448
C	4.290549	4.856029	-0.510576
H	5.211200	5.421316	-0.725701
C	-1.168558	-5.669135	-1.673607
H	-1.732349	-6.492551	-2.140454
C	-1.860512	5.606008	-1.117645
H	-1.775704	6.703412	-1.168326
C	5.669648	-3.161464	-0.472793
H	6.727476	-3.390476	-0.677717
C	-3.098289	3.559269	-1.574335
H	-3.985338	3.048966	-1.982264
C	-2.998161	4.960837	-1.637699
H	-3.806057	5.552536	-2.097073
C	4.864477	-2.582190	-1.472283
H	5.289538	-2.355885	-2.463091
H	2.142206	0.365522	0.041554
H	-0.760585	-0.128220	2.208280
N	-3.403816	-0.573567	1.770027
C	-2.073934	-0.350192	1.489610
O	-1.708618	-0.293155	0.269500
C	-4.543517	-0.768046	0.952422
C	-5.789170	-0.973944	1.598121
C	-4.499644	-0.766441	-0.464739
C	-6.959304	-1.172806	0.851695
H	-5.832398	-0.976486	2.700282
C	-5.681563	-0.967314	-1.199338
H	-3.539479	-0.607441	-0.969423
C	-6.915003	-1.171136	-0.555443
H	-7.915180	-1.330559	1.376706
H	-5.630609	-0.963217	-2.300299
H	-7.833395	-1.327213	-1.142667
H	-3.604324	-0.603209	2.773420

1TSAbB_Ph
Frequencies, energies and thermodynamic properties:

Lowest Vibrational Mode (1/cm) = -538.9976
2nd Lowest Vibrational Mode (1/cm) = 13.1593
E(RB-P86) (a.u.) = -2429.93331183
Thermal correction to Enthalpy (a.u.) = 0.647554
Thermal correction to Gibbs Free Energy (a.u.) = 0.524166
Total Entropy (cal/Kmol) = 259.691
E(RPBE1PBE) (a.u.) = -2429.66587575

Optimised cartesian coordinates (Angstrom):

Ru	0.088446	-0.416336	0.276198
P	2.410316	-0.536508	0.559618
P	-2.200966	-0.812313	0.503363
N	0.110417	-1.094678	2.301383
O	0.052539	0.082959	-2.708290
C	3.345356	-1.891386	-0.302177
C	-1.073148	-1.804661	2.795174
H	-1.060689	-1.847647	3.913794
H	-1.081813	-2.873703	2.453159
C	4.686611	-2.167365	0.056075
H	5.188644	-1.567066	0.832374
C	-3.473556	0.466792	0.077841
C	3.526258	0.923126	0.296407
C	-4.301255	-2.653710	-0.027216
H	-4.922871	-1.996825	0.603164
C	4.208898	1.076774	-0.933786
H	4.144752	0.288568	-1.700503
C	-3.699163	0.737351	-1.293282
H	-3.140289	0.180362	-2.062749
C	-2.357100	-1.104877	2.339492
H	-2.429800	-0.110410	2.826503
H	-3.269589	-1.681432	2.593837
C	2.570744	-0.951081	2.374955
H	3.513717	-1.494609	2.585617
H	2.598780	0.008524	2.929803
C	-2.945668	-2.329473	-0.272083
C	1.329912	-1.750104	2.780744
H	1.409000	-2.800979	2.395020
H	1.307232	-1.840352	3.896299
C	-4.633262	1.708560	-1.686249
H	-4.798739	1.903718	-2.757625
C	-4.197032	1.198742	1.045269
H	-4.047595	1.012656	2.119307
C	3.449138	-3.694876	-1.952431
H	2.960136	-4.287898	-2.741576
C	2.735520	-2.663870	-1.312669
H	1.691960	-2.454416	-1.591817
C	0.068508	-0.025505	-1.529612
C	3.618411	1.956876	1.257751
H	3.080230	1.880387	2.215151
C	5.395747	-3.200470	-0.578338
H	6.437846	-3.406976	-0.286666

C -4.869883 -3.807371 -0.591179
H -5.925525 -4.050393 -0.390426
C -5.129398 2.176795 0.648151
H -5.688171 2.736494 1.415088
C -2.177908 -3.174321 -1.100110
H -1.123942 -2.920743 -1.292556
C 4.777884 -3.967089 -1.585017
H 5.335416 -4.776068 -2.083291
C -5.351836 2.432563 -0.715636
H -6.084399 3.195359 -1.023550
C 4.975555 2.227299 -1.188134
H 5.503034 2.327628 -2.150104
C -4.095097 -4.647237 -1.414298
H -4.543088 -5.550034 -1.859120
C 4.390941 3.103683 1.002719
H 4.456776 3.894831 1.766444
C 5.072430 3.242981 -0.219620
H 5.676008 4.142692 -0.418101
C -2.750734 -4.327269 -1.669315
H -2.140046 -4.977040 -2.316234
H 0.216333 -1.936195 -0.103676
H 0.101021 1.205333 3.303122
H 0.102097 0.415347 3.010516
N -0.213699 2.033749 0.905547
C -0.679943 2.463834 1.990958
O -1.242940 3.106415 2.817301
C -0.300338 2.918359 -0.235987
C 0.818881 3.052231 -1.080881
C -1.478330 3.645497 -0.507527
C 0.758770 3.916603 -2.186491
H 1.730200 2.479972 -0.860335
C -1.529050 4.504853 -1.619121
H -2.359386 3.528633 0.142182
C -0.412410 4.646386 -2.461769
H 1.640754 4.018401 -2.838529
H -2.455113 5.064190 -1.826551
H -0.455292 5.320438 -3.331670

1Ab_Ph
Frequencies, energies and thermodynamic properties:
Lowest Vibrational Mode (1/cm) = 12.1435
2nd Lowest Vibrational Mode (1/cm) = 17.9540
E(RB-P86) (a.u.) = -2430.01930784
Thermal correction to Enthalpy (a.u.) = 0.657786
Thermal correction to Gibbs Free Energy (a.u.) = 0.536432
Total Entropy (cal/Kmol) = 255.412
E(RPBE1PBE) (a.u.) = -2429.76683310
Optimised cartesian coordinates (Angstrom):
Ru -0.079673 -0.328654 -0.019388
P 2.238190 -0.482649 0.275761

P	-2.379578	-0.431354	0.327941
N	-0.049126	0.150001	2.163633
O	-0.205057	-1.467900	-2.799763
C	3.046903	-2.154471	0.391073
C	-1.209966	-0.446229	2.888030
H	-1.241924	-0.065550	3.934317
H	-1.027151	-1.538556	2.940107
C	4.428999	-2.257761	0.676797
H	5.037636	-1.348986	0.813133
C	-3.596753	0.763553	-0.396668
C	3.443824	0.437673	-0.783603
C	-3.006479	-3.153228	0.873992
H	-2.395448	-3.036980	1.782825
C	3.425517	0.173663	-2.173186
H	2.701237	-0.546413	-2.586778
C	-3.219564	1.527456	-1.522029
H	-2.203849	1.417457	-1.930950
C	-2.536322	-0.151168	2.185133
H	-2.820424	0.913572	2.317699
H	-3.352346	-0.762380	2.621115
C	2.435179	0.240842	1.990854
H	3.388913	-0.061894	2.468976
H	2.436693	1.346557	1.882675
C	-3.204552	-2.060085	0.000062
C	1.239400	-0.203470	2.831904
H	1.249165	-1.305154	2.969090
H	1.282860	0.257573	3.844131
C	-4.127241	2.434351	-2.100367
H	-3.822451	3.026131	-2.977841
C	-4.894865	0.919452	0.144301
H	-5.213041	0.321812	1.013919
C	2.915570	-4.594040	0.286063
H	2.319174	-5.506214	0.124891
C	2.297308	-3.332920	0.193075
H	1.221809	-3.249722	-0.029902
C	-0.145637	-0.971920	-1.728597
C	4.375180	1.370201	-0.275493
H	4.416890	1.595540	0.800867
C	5.042333	-3.517382	0.776534
H	6.118546	-3.584285	1.002321
C	-3.577859	-4.405657	0.591235
H	-3.421153	-5.245305	1.287036
C	-5.795324	1.832076	-0.430267
H	-6.803132	1.949760	-0.001013
C	-3.973151	-2.250984	-1.169734
H	-4.139072	-1.411963	-1.863482
C	4.286422	-4.689158	0.580598
H	4.769873	-5.676367	0.653481
C	-5.413051	2.589679	-1.554283
H	-6.121472	3.303515	-2.003893

C	4.322704	0.825248	-3.034660
H	4.294795	0.610963	-4.114688
C	-4.341812	-4.587952	-0.576366
H	-4.785360	-5.571157	-0.799622
C	5.270685	2.024497	-1.142561
H	5.991423	2.750026	-0.732991
C	5.247073	1.754151	-2.521340
H	5.947058	2.269464	-3.197815
C	-4.534122	-3.508797	-1.456556
H	-5.128697	-3.642061	-2.374331
H	-0.166703	-1.853104	0.507738
H	0.501205	1.347359	-2.521896
H	-0.150986	1.184147	2.191513
N	0.165904	1.858472	-0.551747
C	0.501951	2.227664	-1.819948
O	0.750576	3.372286	-2.241182
C	0.197100	2.870777	0.437514
C	-0.939032	3.119544	1.251195
C	1.364214	3.644797	0.685882
C	-0.907590	4.080374	2.280662
H	-1.870919	2.577574	1.028303
C	1.390201	4.605775	1.708774
H	2.250118	3.482375	0.054817
C	0.258974	4.827232	2.520120
H	-1.810679	4.253735	2.888724
H	2.309155	5.191234	1.877804
H	0.285769	5.581031	3.322772

----

6. References

1. K. P. Dhake, P. J. Tambade, R. S. Singhal, B. M. Bhanage, *Green Chem. Lett. Rev.*, 2011, **4**, 151.
2. Z. Han, L. Rong, J. Wu, L. Zhang, Z. Wang and K. Ding, *Angew. Chem. Int. Ed.*, 2012, **51**, 13041-13045.
3. X. Liu and T. Werner, *Adv. Synth. Catal.*, 2021, **363**, 1096-1104.
4. G. M. Sheldrick, *Acta Crystallogr. A*, 2015, **71**, 3-8.
5. G. M. Sheldrick, *Acta Crystallogr. C*, 2015, **71**, 3-8.
6. L. Zhang, G. Raffa, D. H. Nguyen, Y. Swesi. L. Corbel-Demailly, F. Capet, X. Trivelli, S. Desset, S. Paul, J. Paul, P. Fongarland, F. Dumeignil and R. M. Gauvin, *J. Catal.*, 2016, **340**, 331-343.
7. A. Kaithal, B. Chatterjee, C. Werlé and W. Leitner, *Angew. Chem. Int. Ed.*, 2021, **60**, 26500-26505.
8. A. Kaithal, N. Schmitz, M. Hölscher and W. Leitner, *ChemCatChem*, 2019, **12**,781-787.
9. M. J. Frisch, G. W. Trucks, H. B. Schlegel, G. E. Scuseria, M. A. Robb, J. R. Cheeseman, G. Scalmani, V. Barone, G. A. Petersson, H. Nakatsuji, X. Li, M. Caricato, A. V. Marenich, J. Bloino, B. G. Janesko, R. Gomperts, B. Mennucci, H. P. Hratchian, J. V. Ortiz, A. F. Izmaylov, J. L. Sonnenberg, D. Williams-Young, F. Ding, F. Lipparini, F. Egidi, J. Goings, B. Peng, A. Petrone, T. Henderson, D. Ranasinghe, V. G. Zakrzewski, J. Gao, N. Rega, G. Zheng, W. Liang, M. Hada, M. Ehara, K. Toyota, R. Fukuda, J. Hasegawa, M. Ishida, T. Nakajima, Y. Honda, O. Kitao, H. Nakai, T. Vreven, K. Throssell, J. A. Montgomery, Jr., J. E. Peralta, F. Ogliaro, M. J. Bearpark, J. J. Heyd, E. N. Brothers, K. N. Kudin, V. N. Staroverov, T. A. Keith, R. Kobayashi, J. Normand, K. Raghavachari, A. P. Rendell, J. C. Burant, S. S. Iyengar, J. Tomasi, M. Cossi, J. M. Millam, M. Klene, C. Adamo, R. Cammi, J. W. Ochterski, R. L. Martin, K. Morokuma, O. Farkas, J. B. Foresman and D. J. Fox, Gaussian 16, Revision C.01, Gaussian Inc., Wallingford CT, 2019. C. N. Brodie, A. E. Owen, J. S. Kolb, M. Bühl and A. Kumar, *Angew. Chem. Int. Ed.*, 2023, **62**, e202306655.
10. A. E. Owen, A. Preiss, A. McLuskie, C. Gao, G. Peters, M. Bühl and A. Kumar, *ACS Catal.*, 2022, **12**, 6923-6933.
11. A. E. Owen, A. Preiss, A. McLuskie, C. Gao, G. Peters, M. Bühl and A. Kumar, *ACS Catal.*, 2023, **13**, 10796-10797.
12. C. L. Oates, A. S. Goodfellow, M. Bühl and M. Clarke, *Angew. Chem. Int. Ed.*, 2023, **62**, e202212479.
13. C. L. Oates, A. S. Goodfellow, M. Bühl and M. Clarke, *Green Chem.*, 2023, **25**, 3864-3868.
14. A. S. Goodfellow and M. Bühl, *Molecules*, 2021, **26**, 4072.
15. R. L. Martin, P. J. Hays and L. R. Pratt, *J. Phys. Chem. A*, 1998, **102**, 3565-3573.
16. D. Andrae, U. Haeussermann, M. Dolg, H. Stoll, and H. Preuss, *Theor. Chem. Acc.*, 1990, **17**, 123-41.
